# De‐identification procedures for magnetic resonance images and the impact on structural brain measures at different ages

**DOI:** 10.1002/hbm.25459

**Published:** 2021-05-11

**Authors:** Elizabeth E. L. Buimer, Hugo G. Schnack, Yaron Caspi, Neeltje E. M. van Haren, Mikhail Milchenko, Pascal Pas, Alzheimer's Disease Neuroimaging Initiative, Hilleke E. Hulshoff Pol, Rachel M. Brouwer

**Affiliations:** ^1^ UMC Utrecht Brain Center University Medical Center Utrecht, Utrecht University Utrecht Netherlands; ^2^ Department of Child and Adolescent Psychiatry/Psychology Erasmus MC Rotterdam Netherlands; ^3^ Department of Radiology, Washington University School of Medicine Mallinckrodt Institute of Radiology Saint Louis Missouri USA

**Keywords:** aged, brain, child, data anonymization, humans, longitudinal studies, magnetic resonance imaging, privacy, reproducibility of results, young adult

## Abstract

Surface rendering of MRI brain scans may lead to identification of the participant through facial characteristics. In this study, we evaluate three methods that overwrite voxels containing privacy‐sensitive information: Face Masking, FreeSurfer defacing, and FSL defacing. We included structural T1‐weighted MRI scans of children, young adults and older adults. For the young adults, test–retest data were included with a 1‐week interval. The effects of the de‐identification methods were quantified using different statistics to capture random variation and systematic noise in measures obtained through the FreeSurfer processing pipeline. Face Masking and FSL defacing impacted brain voxels in some scans especially in younger participants. FreeSurfer defacing left brain tissue intact in all cases. FSL defacing and FreeSurfer defacing preserved identifiable characteristics around the eyes or mouth in some scans. For all de‐identification methods regional brain measures of subcortical volume, cortical volume, cortical surface area, and cortical thickness were on average highly replicable when derived from original versus de‐identified scans with average regional correlations >.90 for children, young adults, and older adults. Small systematic biases were found that incidentally resulted in significantly different brain measures after de‐identification, depending on the studied subsample, de‐identification method, and brain metric. In young adults, test–retest intraclass correlation coefficients (ICCs) were comparable for original scans and de‐identified scans with average regional ICCs >.90 for (sub)cortical volume and cortical surface area and ICCs >.80 for cortical thickness. We conclude that apparent visual differences between de‐identification methods minimally impact reliability of brain measures, although small systematic biases can occur.

## Graphical abstract

De‐identification methods remove or blur facial characteristics on anatomical MRI scans, but also have a small effect on FreeSurfer brain measures. Furthermore, some methods are too invasive in younger participants or do not always succeed to remove all facial characteristics.

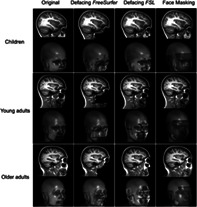

## 1 INTRODUCTION

Advances in magnetic resonance imaging (MRI) technology enable researchers to collect good quality structural MRI scans of the brain. However, brain scans also contain privacy‐sensitive facial characteristics. The participants' faces can be reconstructed with 3D rendering software that is part of most MRI viewers. Recently, this topic received attention from the scientific community as well as popular journals after the release of a study in which face recognition software was used to identify participants based on their MRI scan (Schwarz et al., 2019). This study complements an earlier study that shows how participants can be identified through their 3D renders by humans (Prior et al., 2009). In light of the increasing number of world‐wide (public) neuroimaging collaborations (Poline et al., 2012) and technical improvements, the question is whether sharing raw anatomical MRI images is still in line with privacy regulations (White, Blok, & Calhoun, 2020). From an ethical viewpoint sharing identifiable data may compromise the confidentiality participants consented to. For these reasons, more and more open‐access datasets contain MRI scans that were subjected to some type of de‐identification method.

Different efforts can be made to de‐identify MRI scans (listed here: open‐brain‐consent.readthedocs.io/en/stable/anon_tools.html). First, brain extraction or skull‐stripping removes nonbrain tissue. Second, defacing algorithms remove facial and dental characteristics. Examples of defacing methods are: fsl_deface (Alfaro‐Almagro et al., 2018; fsl.fmrib.ox.ac.uk/fsl/fslwiki/FSL), mri_deface (Bischoff‐Grethe et al., 2007; freesurfer.net/fswiki/mri_deface), pydeface (github.com/poldracklab/pydeface), QuickShear (github.com/nipy/quickshear/), mridefacer (github.com/mih/mridefacer/). Third, Face Masking (Milchenko & Marcus, 2013, nrg.wustl.edu/software/face‐masking) masks the face and, optional, the ear region and thus preserves more anatomical landmarks than defacing methods. An advantage of the latter toolbox is that it can be applied to raw DICOM images, which limits the number of intermediate processing steps. In a recent study, a new method is introduced, mri_reface, to replace voxels in the face and ear region with a population average (Schwarz et al., 2021). In this article, we will focus on defacing with FSL, a method developed by and used in the UK biobank study (Alfaro‐Almagro et al., 2018), defacing with FreeSurfer, for example, used in the BRAINS (Job et al., 2017), CamCAN, (Taylor et al., 2017), ATLAS (Liew et al., 2018) and HID study (Ozyurt et al., 2010) and Face Masking which is implemented in XNAT (Marcus, Olsen, Ramaratnam, & Buckner, 2007), used in the repositories of OASIS (Marcus et al., 2007), HCP (Marcus et al., 2013), and GSP (Holmes et al., 2015).

De‐identification methods are typically optimized for healthy adults, but different results can be expected in other populations for example, related to the amount of atrophy, that is, the closeness of the brain to the skull. A recent study investigated the effects of different de‐identification methods (QuickShear, Face Masking, and FreeSurfer defacing) in patients with multiple sclerosis, Alzheimer's Disease (AD), and glioblastoma (de Sitter et al., 2020). In this study, we focus on age‐related effects by including children, young adults, and older adults (with and without AD). From an ethical perspective, children are an extra sensitive population. Possible age‐related effects of de‐identification procedures on brain measures are also relevant in the light of longitudinal studies investigating brain development or aging.

An optimal de‐identification method (a) prevents participant identification, (b) leaves brain tissue intact, and (c) has a negligible effect on brain measures, that is, the de‐identified scan should approximately generate the same results as an unmasked scan. Previous research shows that these criteria are not always met and even after de‐identification some participants can still be identified with facial recognition software (Abramian & Eklund, 2019; Schwarz et al., 2021). Furthermore, defacing can overwrite a small amount of brain voxels in some cases (Alfaro‐Almagro et al., 2018). Last, de‐identification procedures impact subsequent processing and outcome measures (Holmes et al., 2015; de Sitter et al., 2020; Schwarz et al., 2021). In this study, we focus on the effect of the three de‐identification procedures on brain measures, but we also describe visual aspects of the methods.

To evaluate the de‐identification techniques, we started with a visual check to rate the invasiveness, that is, whether brain voxels are preserved, and to rate the presence of eyes and mouth characteristics after de‐identification. Second, we assessed whether de‐identification altered brain measures differently in children, young adults, and older adults. To this end, we extracted regional subcortical and cortical volumes, cortical surface area, and cortical thickness of original scans and de‐identified scans. Last, we quantified how the effects of de‐identification techniques on brain measures compared to test–retest reliability in young adults.

## 2 MATERIALS AND METHODS

### 2.1 Samples

#### 2.1.1 Sample of children

The children's data consisted of 25 children from the general population (8 male) with a mean age of 9.5 (0.9) years within a range from 8 to 11 years. These data were collected as part of the first wave in a large longitudinal study on brain development in Utrecht, the Netherlands: The YOUth cohort study (Onland‐Moret et al., 2020). The subset included in this study is labeled as pilot data. The children's parents or guardians gave written consent. Included MR images were of good quality after exclusion of scans with poor contrast or major motion artifacts such as ringing.

#### 2.1.2 Sample of young adults

The sample of young adults consisted of 16 volunteers from the general population (6 male) with a mean age of 23.6 (3.3) years within a range from 19 to 31 years. To assess the test–retest reliability of the YOUth MRI protocol, the adults were scanned twice using the same acquisition parameters. The scan–rescan interval was between 6 and 8 days. The adult dataset was acquired in the context of protocol development. The adults signed written informed consent. All available scans were of good quality without major motion artifacts or other artifacts.

#### 2.1.3 Sample of older adults

The elderly sample was selected from the large Alzheimer's Disease Neuroimaging Initiative (ADNI, adni.loni.usc.edu). ADNI was launched in 2003 as a public‐private partnership, led by Principal Investigator Michael W. Weiner, MD. The primary goal of ADNI has been to test whether serial MRI, positron emission tomography (PET), other biological markers, and clinical and neuropsychological assessment can be combined to measure the progression of mild cognitive impairment (MCI) and early AD. Selected scans were of good quality without major motion or dental artifacts based on visual quality control in addition to provided quality assessment codes. Furthermore, the subset was created to ensure a heterogenous sample with regard to age, sex and patient‐control status. The older adults sample consisted of 43 elderly participants: 22 participants (7 male) with AD with a mean age of 72.6 (7.5) years ranging from 56 to 86 and 21 age‐matched participants (9 male) without cognitive impairment with a mean age of 74.6 (5.9) years ranging from 65 to 85.

### 2.2 Acquisition parameters

The ADNI subset was selected with uniform acquisition parameters, that resembled the parameters of the YOUth MRI protocol, used for the acquisition of the child and adult data. All acquisition parameters can be found in Table 1.

**TABLE 1:** Acquisition parameters

	Children and young adults	Older adults *ADNI 2*
Parameters	*YOUth MRI protocol*	*(Philips: MPRAGE)*
Multicenter	No, a single MR scanner	Yes
Type of MR scanner	Philips Ingenia	Philips Achieva
Field strength (T)	3.0	3.0
Head‐coil	32‐channel SENSE head‐coil	8‐channel SENSE head‐coil
Scan	Structural T1W 3D GRE	Structural T1W 3D GRE
Scan orientation	Sagittal	Sagittal
TR (ms)	10	6.8
TE (ms)	4.6	3.1
Flip angle (degrees)	8	9
Field of view (mm)	240 × 240 × 200	256 × 240 × 204
Acquisition matrix	304 × 304	256 × 240
Reconstructed voxel size (mm^3^)	0.75 × 0.75 × 0.80	1.00 × 1.00 × 1.20

Abbreviations: GRE, gradient echo; T1W, T1‐weighted; T, Tesla; TE, echo time; TR, repetition time.

### 2.3 De‐identification methods

All scans were subjected to three de‐identification methods. FreeSurfer defacing was applied using version 1.22 of the mri_deface function (Bischoff‐Grethe et al., 2007; https://surfer.nmr.mgh.harvard.edu/fswiki/mri_deface) in FreeSurfer version 6.0 (Fischl et al., 2002). FSL defacing was applied using version 1.0.0 of the fsl_deface function (Alfaro‐Almagro et al., 2018) in FSL 6.0.1 (Jenkinson, Beckmann, Behrens, Woolrich, & Smith, 2012; https://fsl.fmrib.ox.ac.uk/fsl/fslwiki/). The Face Masking toolbox was applied with default coarseness and an ear mask using version December 26, 2017 of the mask_face function (Milchenko & Marcus, 2013; https://nrg.wustl.edu/software/face-masking/). The toolbox is implemented in neuroinformatics platform XNAT (Marcus, Olsen, et al., 2007), but in this article we used the offline toolbox.

For face masking, we additionally compared different mask settings in children and young adults by varying the coarseness of the mask. Furthermore, we investigated the effect of switching the ear mask option on or off (−e flag). The coarseness of the mask was varied between 0.1 and 1.2 in steps of 0.1 (default value is 1) by adjusting the grid step variable (−s flag). The coarseness variable applied to both the face and ear mask.

### 2.4 Visual inspection

A visual inspection of the de‐identified scans was performed with MRIcroGL (version July 14, 2017, www.mccauslandcenter.sc.edu/mricrogl/). A 3D rendering was created to inspect the effect of de‐identification on the face and ear characteristics. Two raters rated the fraction of participants in each age group where the eyes or the mouth were preserved after de‐identification. Next, all 2D axial brain slices were checked and coded by three independent raters. The raters rated whether brain tissue was left intact or whether more than a few brain voxels were removed or blurred by the de‐identification method. For FSL defacing and Face Masking, the invasiveness of the method in the ear area was assessed separately. FreeSurfer defacing currently does not provide an option to remove ears.

Additionally, one rater assessed the invasiveness of different coarseness settings in the child sample and the adult sample (first time point). The mask setting that resulted in no overlap with brain tissue in any participant (child or adult) was labeled “the noninvasive mask.”

### 2.5 Visualization of de‐identification

**FIGURE 1:**
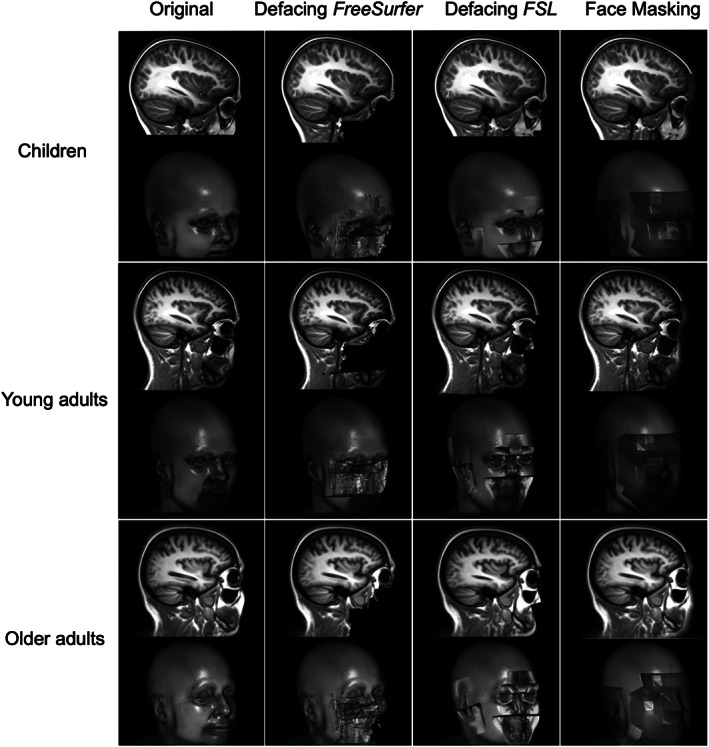
Visual appearance of scans before and after de‐identification. The first column shows the original scan before de‐identification and the other columns show the visual appearance of the scans after different de‐identification options. Each row shows the de‐identified average brain of a specific age group. Within each row a sagittal slice is shown on top and the 3D render below. To prevent identification, the face renderings shown here are renders of the average scans for each sample

To show the effects of de‐identification while preventing participant identification, we created average brains (Caspi et al., 2020; Peper et al., 2009). In short, the individual scans were registered to Talairach space and corrected for nonuniformity followed by a series of linear and nonlinear warpings of the scans (Collins, Holmes, Peters, & Evans, 1995). The average child brain was created by averaging the scans of all 25 children. The average young adult brain was created using the first scan of each young adult, 16 in total. The average older brain was created by averaging the scan of all older participants, 43 in total. To visualize the effect of de‐identification on the brain (Figure 1), the average brains were used as input to the masking and defacing tools.

### 2.6 MRI processing

Face Masking was applied to the raw DICOMs, after which they were converted from DICOM to NIfTI format (dcm2niix, https://github.com/rordenlab/dcm2niix) together with the original DICOM files. Face Masking could not be applied to NIfTI format. Both defacing tools accept only NIfTI input and were therefore applied to the original scans after conversion to NIfTI. FreeSurfer version 6.0 was used for automatic brain segmentation and parcellation (Fischl et al., 2002; freesurfer.net). Global and regional brain measures of subcortical volume, cortical volume, cortical surface area, and cortical thickness were extracted. The Desikan–Killiany atlas was used for cortical parcellation (Desikan et al., 2006). No additional quality check procedures were performed on the segmentations and parcellations. Besides atlas‐based measures of cortical thickness, vertex‐wise cortical thickness was extracted. For the vertex‐wise analysis, cortical thickness of each scan was resampled to the average brain. After resampling, the cortical surfaces were smoothed with a 3D Gaussian kernel (FWHM = 10 mm).

### 2.7 Statistical analysis

We computed several measures to assess the impact of de‐identification on brain measures: the intraclass correlation coefficient (ICC) of absolute agreement (Bartko & Carpenter Jr., 1976; McGraw & Wong, 1996; Shrout & Fleiss, 1979; Koo & Li, 2016) is traditionally used in test–retest context and captures random variation as well as systematic biases. To disentangle these two types of variation and get a better understanding of the effects of de‐identification on brain measures, we computed Pearson correlation coefficients (Pearson's *r*) for coherence, and paired *t*‐tests and signed percentage differences (PD) for assessment of systematic bias. As an indication of the variance introduced by de‐identification we also computed the coefficient of variation (CoV) and absolute PDs. All these statistics were computed for the comparisons described below.

First, we investigated the differences between the original and the de‐identified scans in children, young adults (first time point), and older adults. The ADNI data were analyzed as a whole and in two separate groups based on diagnosis of AD. Second, the scan–rescan adult data were used to calculate the test–retest reliability of brain measures from de‐identified scans and compare this to the test–retest reliability of the original scans. Last, to separate the effect of de‐identification from test–retest reliability, we calculated the agreement between brain measures from the first scan session and the second scan session, masking, or defacing only the second scan. The test–retest reliability of the original scans in this study was also reported elsewhere (Buimer et al., 2020).

Additionally, the coarseness setting of the noninvasive mask was defined based on the visual inspection described above. To compare the mask with default coarseness and the noninvasive mask, both with and without ear mask, we computed ICCs between brain measures derived from the original scans versus one of the mask settings. This additional analysis was done in the child sample and the adult sample (first time point), because in older adults invasiveness was less of an issue likely due to an increased distance between the skull and the brain.

All statistics were computed in R (version 3.5.0, April 23, 2018). ICCs and their 95% confidence intervals were calculated with the “irr package” (version 0.84). based on a single measure, absolute‐agreement, 2‐way model. Local ICCs were visualized and average ICCs are reported over all regions or vertices. When averaging Pearson correlations or ICCs, the average was computed after Fisher's *Z* transformation of the individual values and then transformed back.

### 2.8 Visualization of reliability

For visualization of local reliability, ICCs were color‐coded using colormap “jet” in MATLAB_R2017b. Next, region‐ or vertex‐wise color‐coded cortical ICCs were overlaid on the cortical surface of the average brain using FreeSurfer's tksurfer.

## 3 RESULTS

### 3.1 Visual inspection

Figure 1 shows the effect of the different de‐identification methods from an outer and sagittal view. The first column shows the original average scans for each age category. The 3D renders show facial and ear characteristics in great detail. The second column shows that defacing with FreeSurfer removes facial characteristics in a confined part of the face only, preserving ear characteristics, and in some participants sensitive information in the area of the eyes or the mouth. Full preservation of the eyes occurred in up to 10% of the participants and full preservation of the mouth in up to 27% of the participants, but the inter‐rater reliability for this assessment was low (Table 2). The third column shows that defacing with FSL removes most facial and ear characteristics, but some characteristics around the eyes remain. Full preservation of the eyes occurred often in children (18%) and only incidentally in young adults and older adults (Table 2). The fourth column shows that Face Masking results in blurring of the full face and ear area.

**TABLE 2:** Presence of eyes and mouth characteristics after de‐identification

	Defacing FreeSurfer	Defacing FSL	Face masking
Children—eyes	8%	18%	0%
Young adults—eyes	0%	6%	0%
Older adults—eyes	10%	5%	0%
Children—mouth	4%	4%	0%
Young adults—mouth	13%	0%	0%
Older adults—mouth	27%	0%	0%

*Note*: For each sample, the percentage of individuals is given for whom the eyes or mouth was preserved in the 3D render after de‐identification. The percentages are the average of the percentages given by two raters. The inter‐rater variability was 0.48 based on a one‐way model of absolute agreement.

Defacing with FreeSurfer was not invasive. With this method no brain tissue was removed across all scans. Defacing with FSL resulted in the removal of some brain tissue in the majority of children and in some young adults. Face Masking resulted in blurring of some brain tissue in all children and some young and older adults. Both FSL defacing and Face Masking were more invasive in younger participants compared to older participants. Furthermore, invasiveness was higher in proximity to the ear compared to the face region. Table 3 lists the percentages of scans in which brain tissue was affected by de‐identification averaged over three ratings.

**TABLE 3:** Invasiveness of each de‐identification method

	Defacing FreeSurfer^a^	Defacing FSL	Face masking
Children—face	0%	85%	100%
Young adults—face	0%	34%	10%
Older adults—face	0%	0%	6%
Children—ears	NA	64%	100%
Young adults—ears	NA	12%	56%
Older adults—ears	NA	0%	24%

*Note*: For each sample, the percentage of individuals is given for whom the procedure was too invasive, that is, brain tissue was blurred or removed due to de‐identification of the face or ears. The percentages are the average of the percentages given by three raters. The inter‐rater variability was 0.63 based on a one‐way model of absolute agreement.

a Currently, FreeSurfer does not provide an option to de‐identify ears.

### 3.2 The effects of de‐identification on brain measures

**FIGURE 2:**
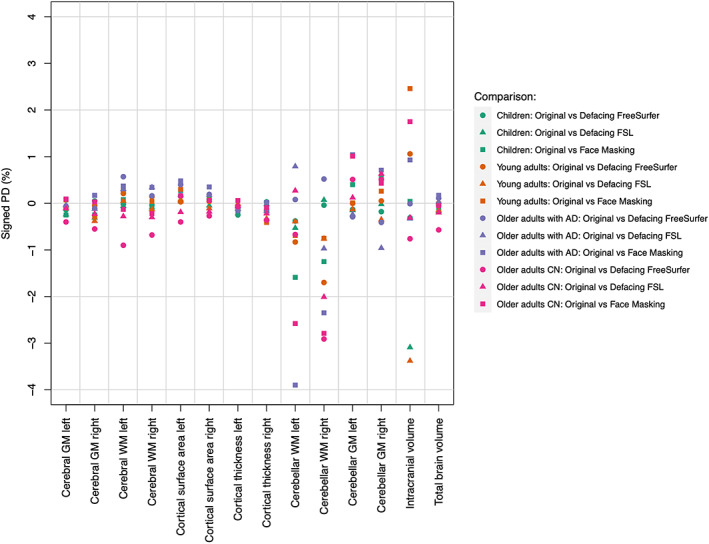
Average signed percentage differences for global brain measures after de‐identification. AD, Alzheimer's Disease; CN, no cognitive impairment; GM, Gray matter; WM, White matter

**FIGURE 3:**
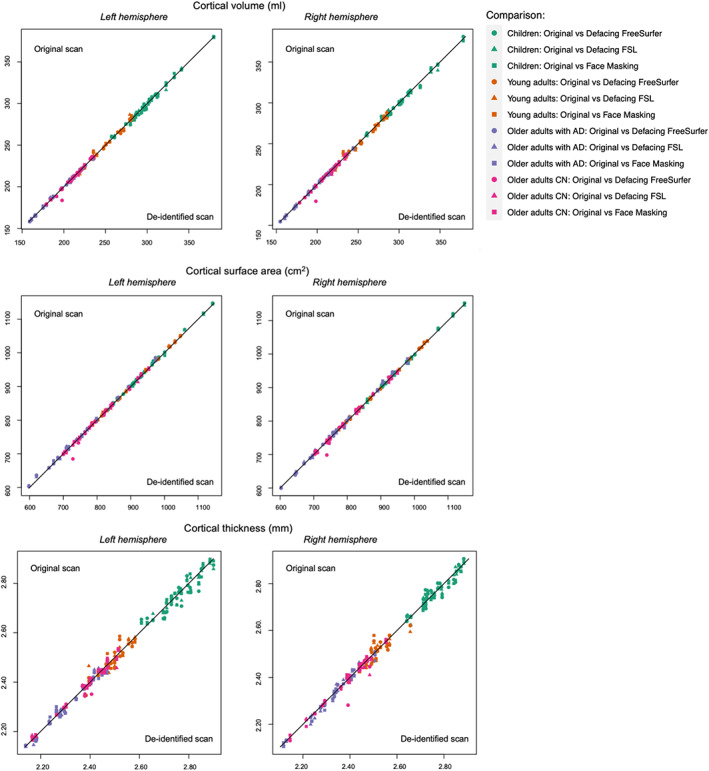
Individual global brain measures derived from original versus de‐identified scans. AD, Alzheimer's Disease; CN, no cognitive impairment

All original and de‐identified scans were successfully processed using FreeSurfer. Brain measures were altered by de‐identification procedures. However, the effect of de‐identification on brain measures was small, that is, absolute PDs were on average <5% for any age category or de‐identification method. Means, standard deviations, and corresponding CoVs were comparable before and after de‐identification. Figure 2 shows the signed PDs for global brain measures. For most brain measures the signed PDs averaged out to around zero, but for cerebellar white matter volume and intracranial volume larger systematic biases were found depending on the age category and de‐identification method. Systematic biases for average cortical thickness, total cortical surface area, and total cortical volume were very small, as suggested by the signed PDs (Figure 2) and scatterplots (Figure 3).

In children, all correlations were above .90 except for the ICC of intracranial volume with FSL defacing (Pearson's *r* = .95; ICC = .87). In young adults, all correlations for global brain measures were above .90 except right hemisphere cortical thickness for all de‐identification techniques and left hemisphere cortical thickness with FSL defacing and Face Masking (Pearson's *r* >.8; ICCs >.8). In older adults, only the correlations for the cerebellar white matter were below .90 in the left hemisphere for all methods (Pearson's *r* >.5; ICCs >.5) and in the right hemisphere only for Face Masking (Pearson's *r* >.8; ICCs >.8). In addition to the lower correlations in the cerebellar white matter in the full group of older adults, lower correlations were found for right hemisphere cerebellar gray matter in older adults without AD (Pearson's *r* >.5; ICCs >.5).

**FIGURE 4:**
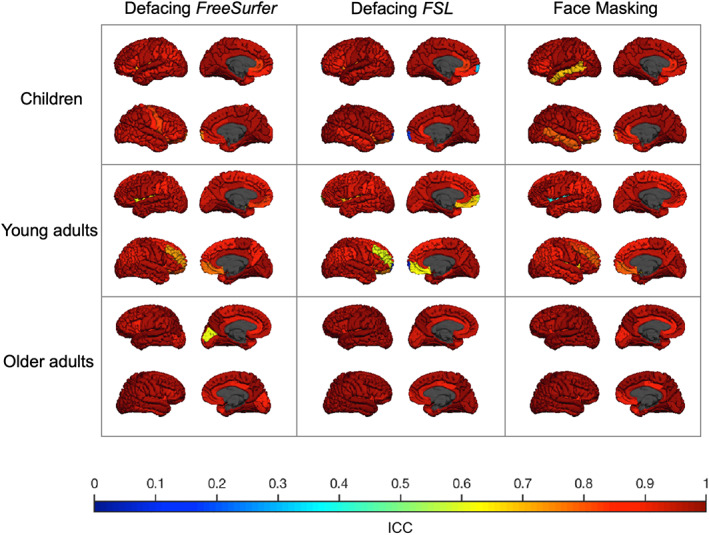
ICC of cortical thickness derived from original versus de‐identified scans. The ICC for each sample (children, young adults, and older adults) is plotted on the corresponding average scan. Each column shows a different de‐identification technique. Within each square, the left hemisphere (top) and the right hemisphere (bottom) are shown from an outer and medial view. The lowest ICC (.07) was found in the right frontal pole in young adults using FSL defacing

**FIGURE 5:**
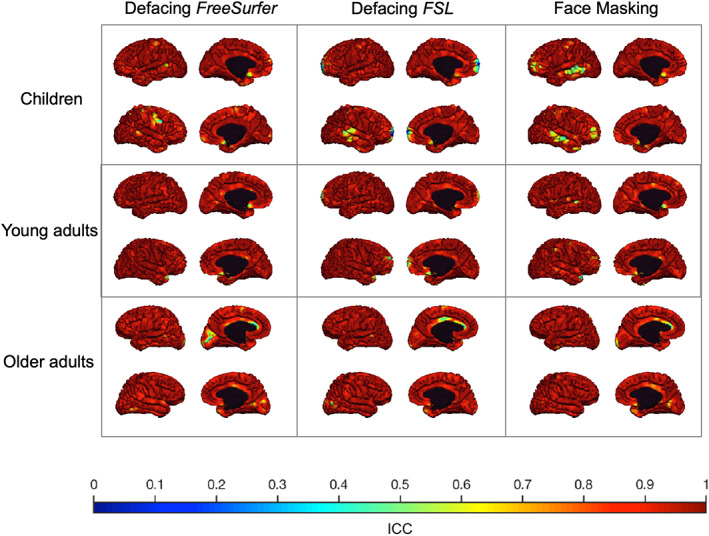
ICC of vertex‐wise cortical thickness derived from original versus de‐identified scans. The ICC for each sample (children, young adults, and older adults) is plotted on the corresponding average scan. Each column shows a different de‐identification technique. Within each square, the left hemisphere (top) and the right hemisphere (bottom) are shown from an outer and medial view

The correlation between regional brain measures derived from original scans and de‐identified scans was on average higher than .90 using Pearson's *r* and ICCs. In general, similar correlations were found using Pearson's *r* or ICCs. Regional ICCs for cortical volume were high in children, young adults, and older adults (Figure S1). Average regional ICCs for cortical surface area in children, young adults, and older adults were high for each method in each age category (Figure S2). Average regional ICCs for cortical thickness in children, young adults, and older adults were on average also high (ICCs >.93) for each method in each age category (Figure 4, see Figure 5 for the vertex‐wise ICCs). Despite these high ICCs, cortical volumes in children were significantly different in more regions than expected by chance after Face Masking or FSL defacing, with in general smaller volumes after de‐identification. In older adults, cortical surface area was significantly different in more regions than expected by chance after Face Masking and in the AD subsample after both Face Masking and FreeSurfer defacing, with generally larger surface areas after de‐identification.

All statistics comparing regional and global brain measures of original versus de‐identified can be found in Tables S1–S5.

### 3.3 Face masking: Varying the coarseness of the mask in children and young adults

In order to better understand the effect of mask invasiveness on reliability of brain measures we varied the coarseness settings of the face and ear masks in children and young adults. In Figure S3, we define settings for a noninvasive mask based on visual inspection. The noninvasive mask had a coarseness setting (grid step value) of 0.6. At this value the brain tissue of all participants was untouched by both the face and the ear mask. Figures S4 and S5 show the ICCs for brain measures in cortical regions for the noninvasive and default mask (with and without ear mask) compared to the original scans in children and young adults, respectively. We show that different coarseness settings generate similar reliability of brain measures. Furthermore, adding or removing the ear mask has minimal effect on reliability.

### 3.4 Effect of de‐identification on test–retest reliability of brain measures

**FIGURE 6:**
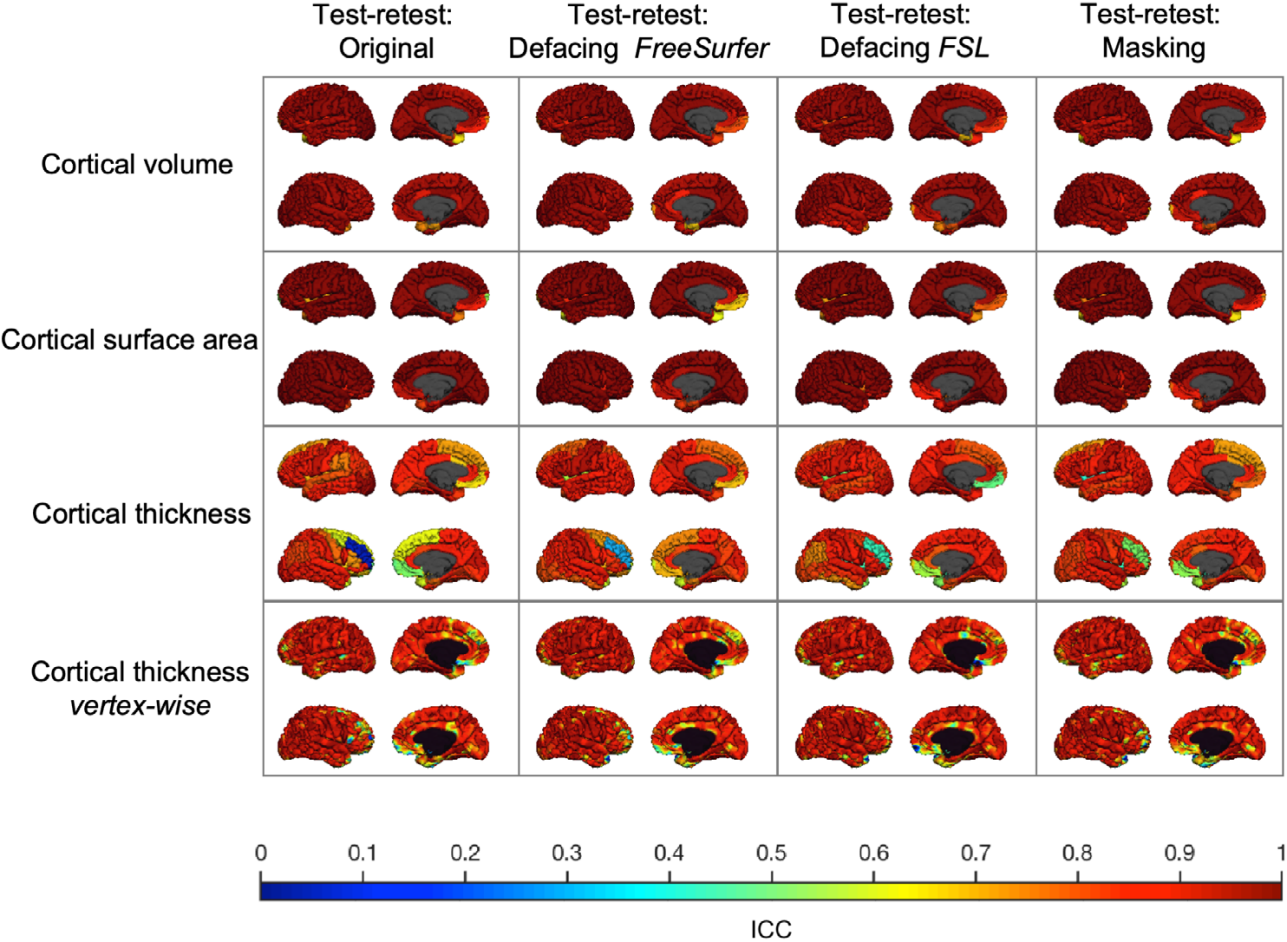
Test–retest reliability in young adults using different de‐identification techniques. The test–retest ICC for each type of brain measure is plotted on the average adult scan. The first column shows the test–retest reliability of the original scans. The other columns show the test–retest reliability if both scans are subject to a de‐identification technique. Within each square, the left hemisphere (top) and the right hemisphere (bottom) are shown from an outer and medial view. Lowest reliability was found for cortical thickness in the rostral middle frontal gyrus of the right hemisphere independent of de‐identification procedures. The poor reliability in this region was not related to parcellation or segmentation errors and could not be explained by a single outlier

**FIGURE 7:**
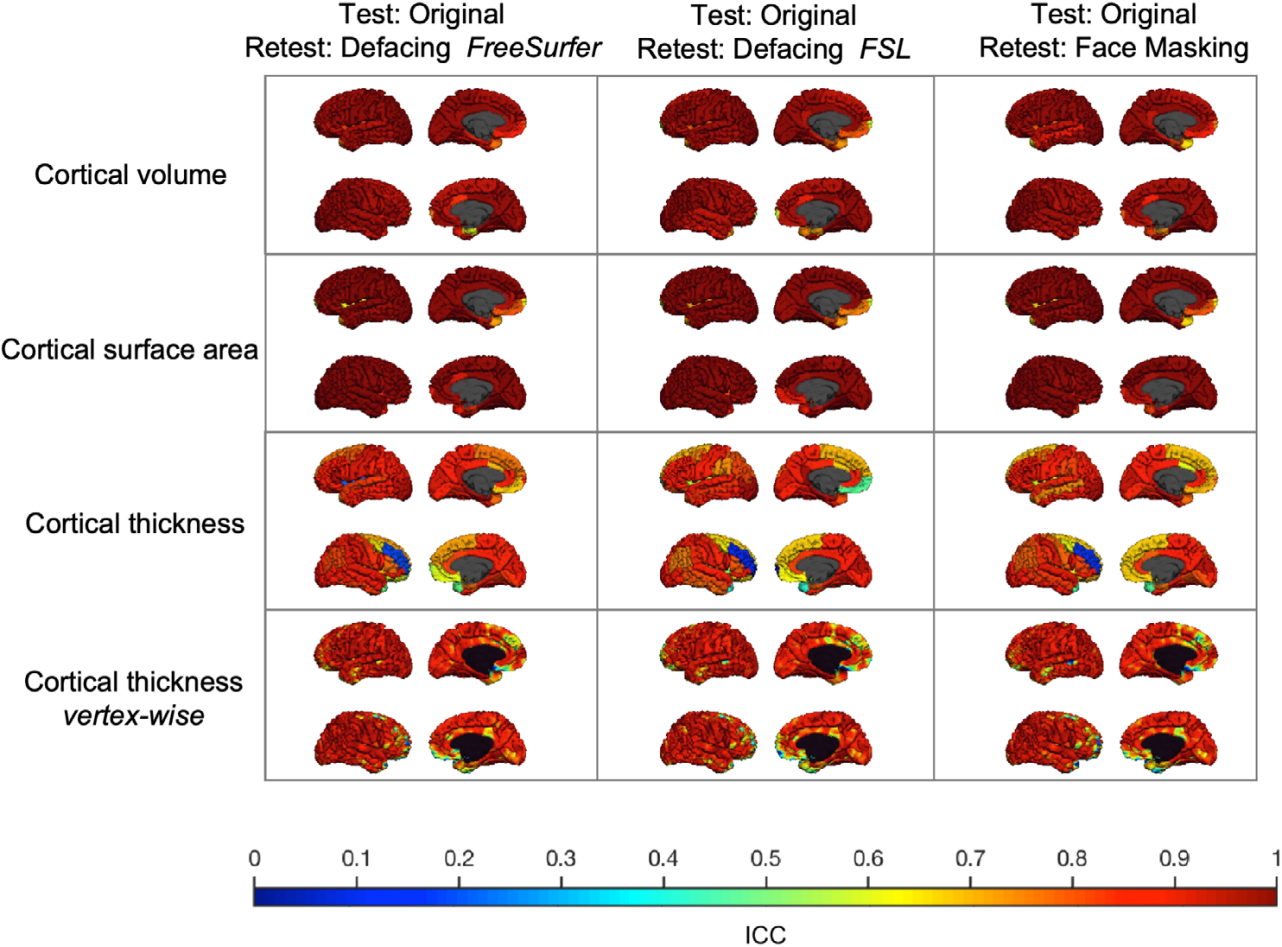
Test–retest reliability in young adults de‐identifying only the second scan. The test–retest ICC for each type of brain measure is plotted on the average adult scan. Each column shows the test–retest reliability of the original scan compared to a de‐identified second scan using different techniques. Within each square, the left hemisphere (top) and the right hemisphere (bottom) are shown from an outer and medial view

Independent of de‐identification procedures, global brain measures were highly reliable, although reliability for cortical thickness was lower than for the other measures. The test–retest reliability for cortical thickness in the original scans was .89 for the left hemisphere and .74 for the right hemisphere. The other global brain measures had test–retest ICCs above .90. Test–retest reliability was similar for scans with or without de‐identification for regional brain measures. Furthermore, de‐identifying only the second scan did on average not result in different regional test–retest ICCs. Figure 6 shows the regional test–retest ICCs for cortical brain measures with or without de‐identification. Figure 7 shows similar cortical test–retest ICCs when de‐identifying only the second scan.

All test–retest statistics can be found in Tables S6–S8.

## 4 DISCUSSION

In this study, we evaluated three de‐identification methods for structural MRI scans: Defacing in FreeSurfer, defacing in FSL, and the Face Masking toolbox. Our main goal was to assess the effect of these methods on the reliability of global and regional brain measures. In addition, we aimed to determine the utility of these methods in cohort studies investigating development or aging. To our knowledge this is the first study into the effects of de‐identification procedures that includes neuroimaging data from children.

We show that using Face Masking and FSL defacing, voxels in the brain are overwritten, especially in children and especially in proximity to the ears. Face Masking provides the option to decrease the coarseness of the mask. This prevents the blurring of brain tissue and results in similar reliability measures. However, reducing coarseness may come at the expense of the full covering of facial features. The age‐dependent effect of these de‐identification methods could be related to the distance of the skull to the brain, which increases with aging due to atrophy. FreeSurfer defacing did not remove voxels in the brain. Furthermore, FreeSurfer defacing and FSL defacing did not succeed in fully removing facial characteristics in all participants.

The obvious visual differences between the methods do not translate to differences in the reliability of brain measure estimates. In general, high correlations were found between brain measures derived from original scans versus de‐identified scans but in some regions lower correlations were found independent of the used method. Pearson's correlations were in most cases similar to ICCs. We found some evidence for small systematic biases and significantly different brain measures after application of de‐identification methods. These biases were not equally distributed over the age groups, disease‐status groups for the ADNI data, and de‐identification methods. This suggests age‐specific biases. Still, these effects were very small.

**FIGURE 8:**
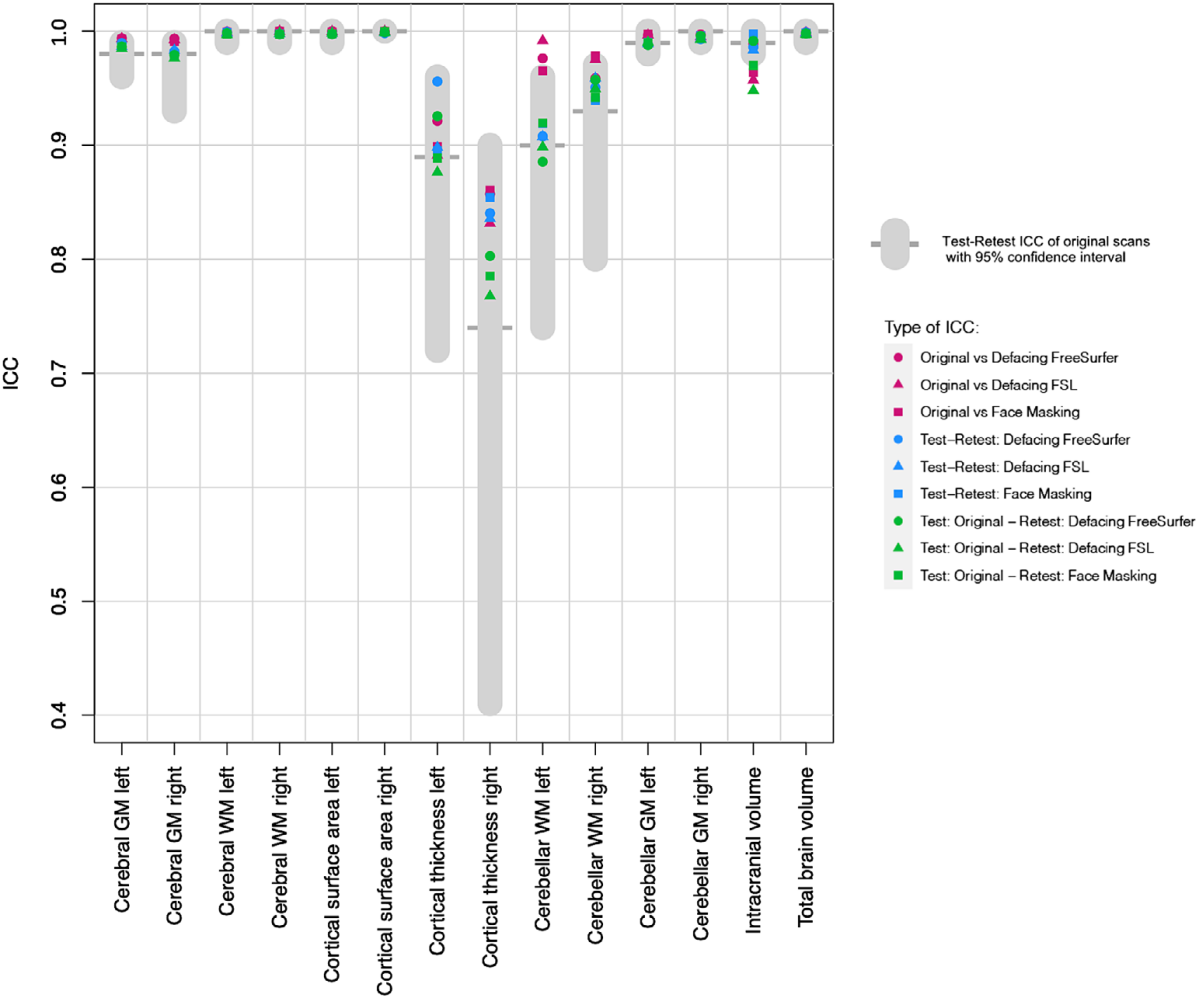
Test–retest ICCs of global brain measures compared to de‐identification ICCs. This figure allows for direct comparison of the ICCs for global brain measures in young adults reported in our study. In gray, test–retest ICC and 95% confidence interval of the original scans, that is, without any de‐identification. In purple, ICCs comparing original adult scans to de‐identified adult scans. In blue, test–retest ICCs when both scans are de‐identified. In green, test–retest ICCs when only the second scan is de‐identified. The type of de‐identification applied is indicated by the shape of the data point. GM, Gray matter; WM, white matter

The ICCs found in this study are comparable to test–retest ICCs of the original scans in young adults, for a direct comparison of ICCs see Figure 8. Theoretically, the amount of noise added by defacing or Face Masking should be far less than the amount of noise introduced in the test–retest procedure. Given that brain deviations found in imaging studies are generally small, introducing an error that is similar in size to test–retest differences is undesirable. However, we argue that the amount of added noise is limited based on the finding that ICCs between original first session scans and de‐identified second session scans were comparable to test–retest ICCs, suggesting no additional effect of de‐identification on reliability. In a previous study, we modeled how these test–retest ICCs relate to power and effect of interest (Buimer et al., 2020). Based on the ICCs found in the current study, it can be hypothesized that as long as all scans are uniformly processed, the sample size needed to detect an effect of interest is the same for original and de‐identified scans. However, sample sizes may be decreased after the necessary visual quality control, which could lead to more exclusions than usual. Scans might need to be removed when privacy‐sensitive features are not fully de‐identified or when brain tissue is impacted.

Regional test–retest ICCs of unmasked scans had an average of .95 for subcortical volume, .96 for cortical volume, .98 for cortical surface area, and .84 for cortical thickness. Lower reliability for cortical thickness measures compared to cortical volume or cortical surface area has been reported before (Iscan et al., 2015; Liem et al., 2015; Wonderlick et al., 2009). Brain measures with a high between‐subject variability naturally generate higher ICCs. Surface area and subcortical volume have a higher between‐subject variability than cortical thickness. This may explain why cortical thickness test–retest ICCs are lower on average, but it does not explain the region‐specific lower ICCs when comparing atlas‐based results with vertex‐wise results. In our study, the lower average ICC for atlas‐based cortical thickness was mostly driven by poor reliability in specific frontal regions in the right hemisphere. Vertex‐wise cortical thickness in these regions was more reliable. Furthermore, lower test–retest ICCs were found in areas that are known to be unreliable, such as the frontal pole (Desikan et al., 2006).

Our study complements previous work from de Sitter et al. (2020) and Schwarz et al. (2021). de Sitter et al. (2020) compared three de‐identification methods: QuickShear (Schimke & Hale, 2011), Face Masking (Milchenko & Marcus, 2013), and FreeSurfer defacing (Bischoff‐Grethe et al., 2007). Brain measures of interest and corresponding processing pipelines in this study were tailored to the patient groups (e.g., BraTumIA for segmentation of glioblastoma) and are therefore hard to compare to our populations, except for an analysis using SIENAX on the ADNI data. Schwarz et al. (2021) compared four different de‐identification methods: FreeSurfer defacing (Bischoff‐Grethe et al., 2007), FSL defacing (Alfaro‐Almagro et al., 2018), pydeface (Gulban et al., n.d.), and the newly developed method mri_reface (Schwarz et al., 2021). We add to this literature by including neuroimaging data from children to directly compare the effects of defacing on brain images of individuals at different ages and by adding a test–retest dataset of subjects scanned 1 week apart. Both earlier studies show that all de‐identification methods impacted subsequent image processing and highlighted the possible role of altered image registration (de Sitter et al., 2020; Schwarz et al., 2021). Accordingly, we noted that de‐identification has a small effect on the Talairach transformation at the start of the FreeSurfer segmentation pipeline, which resulted in altered registration for all de‐identification methods used. Small effects on registration can have large effects on brain measures in areas that are difficult to parcellate. In addition, scan–rescan variability can affect registration, resulting in different segmentations, and parcellations. Here, we show that in general, the effects of repeated scanning (test–retest) were higher than the effects of de‐identification on brain measures. Similar results were found for within‐scanning‐session test–retest reliability in ADNI data (Schwarz et al., 2021). de Sitter et al. (2020) and Schwarz et al. (2021) both describe a systematic bias toward lower brain volumes. We also found some systematic biases, but these biases were not consistent across all age groups, all methods, and all regions. The small systematic effects did not translate in lower ICCs, which suggests that most study results will not be impacted by such a bias because between‐subject variation will be similar if all scans are processed using the same de‐identification method. These biases may be more important when studying longitudinal trajectories. Whether these biases would influence results and whether within‐person effects of de‐identification are stable over time remains an open question at this point.

Neuroimaging results are impacted by methodological choices such as scanner make and model, type of acquisition, study sample, processing pipeline, and atlas (Vijayakumar, Mills, Alexander‐Bloch, Tamnes, & Whittle, 2018). In this study, we only used high quality data obtained from T1‐weighted scans acquired on a 3T Philips scanner, and all scans were processed with FreeSurfer. Therefore, the reported effects of de‐identification could be study‐specific. Other scanners or different quality data may generate different results. With regard to pipeline‐specific effects, previous studies show the effect of de‐identifying ADNI data on brain measures using other processing pipelines (de Sitter et al., 2020; Schwarz et al., 2021). Furthermore, based on this study we cannot draw conclusions about brain measures derived with other software or other types of image acquisitions such as T2‐weighted scans. Face Masking can be applied on CT scans and T1‐ and T2‐weighted MRI scans. Defacing with FSL can be applied on T1‐ and T2‐weighted MRI scans. Defacing with FreeSurfer is based on a T1‐weighted face mask, but in principle it would be possible to use this method on T2‐weighted MRI scans as well. Another limitation of the current study is that only adult test–retest data were included. No ethical approval was granted to collect test–retest data in the YOUth cohort. For the ADNI cohort test–retest data (without repositioning the participant in the scanner) is available. These test–retest data were compared to the effects of de‐identification before (Schwarz et al., 2021). Last, an important limitation is that we were not able to extensively study whether the de‐identification methods were successful, because we did not have access to photographs of the participants. Automated facial recognition methods are probably the best tool to test if de‐identification was successful. A recent study using automated facial recognition showed that defacing data with FreeSurfer reduces the probability of identifying a participant from 97 to 10%. Using FSL defacing the probability was reduced even further to 3% (Schwarz et al., 2021). Face Masking was not included in this study because preliminary evidence suggests that this de‐identification technique could be reversible (Abramian & Eklund, 2019).

In conclusion, de‐identification methods impact recognizable facial characteristics, but the side effect is that brain measures are impacted as well. We show that if de‐identification is a necessity, masking, or defacing can be considered, as global brain measures can be estimated reliably and in general local brain measures are minimally affected. We also observe that, these methods do not de‐identify all participants beyond recognition, which may lead to exclusions of scans. Thus, the perfect de‐identification method, that is, one that does not impact brain measures and does not result in additional exclusion of scans, does not exist yet. This article highlights the importance to further develop de‐identification methods, especially for neuroimaging data from children.

## Supporting information


**Appendix** **S1.** Supporting Information.

## Data Availability

Processed data and scripts that support the findings of this study are available on request from the corresponding author. The raw and processed MRI scans are not publicly available due to privacy or ethical restrictions. Requests for the child MRI scans can be submitted here: https://www.uu.nl/en/research/youth‐cohort‐study/data‐access. Requests for MRI scans of the ADNI data can be submitted here: http://adni.loni.usc.edu/data‐samples/access‐data/.
