# Supplementary material for: De‐identification procedures for magnetic resonance images and the impact on structural brain measures at different ages
Source: Hum Brain Mapp. 2021 May 11;42(11):3643–55. doi: 10.1002/hbm.25459 (PMC8249889; doi:10.1002/hbm.25459)
Supplement: Supplementary file 1 — Appendix S1. Supporting Information. [file HBM-42-3643-s001.pdf]

De-identification procedures for magnetic resonance images and the impact on structural brain measures at different ages

Supplementary Tables and figures

Elizabeth E.L. Buimer[1*], Hugo G. Schnack[1], Yaron Caspi[1], Neeltje E.M. van Haren[1,2], Mikhail Milchenko[3], Pascal Pas[1], Alzheimer's Disease Neuroimaging Initiative[4], Hilleke E. Hulshoff Pol[1], Rachel M. Brouwer[1]

**Corresponding author: Heidelberglaan 100 (Room A01.161), 3584CX Utrecht, The Netherlands. 0031-887556365, e.e.l.buimer@umcutrecht.nl*

Table of Contents

Figure S1. ICC of cortical volume derived from original versus de-identified scans. 2

Figure S2. ICC of cortical surface area derived from original versus de-identified scans. 2

Table S1. Effects of de-identification on brain measures in children. 3

Table S2. Effects of de-identification on brain measures in young adults. 10

Table S3. Effects of de-identification on brain measures in older adults. 17

Table S4. Effects of de-identification on brain measures in older adults diagnosed with Alzheimer's disease. 24

Table S5. Effects of de-identification on brain measures in older adults without cognitive impairment. 31

Figure S3. De-identification properties and invasiveness of masks with varying coarseness. 38

Figure S4. ICC between brain measures in children derived from original scans versus scans subjected to different mask settings. 39

Figure S5. ICC between brain measures in adults derived from original scans versus scans subjected to different mask settings. 39

Table S6. Effects of de-identification on test-retest reliability in young adults: Means, coefficients of variation and t-tests 40

Table S7. Effects of de-identification on test-retest reliability in young adults: Average signed and absolute percentage differences 47

Table S8. Effects of de-identification on test-retest reliability in young adults: Pearson's correlation coefficients and intra-class correlation coefficients 53

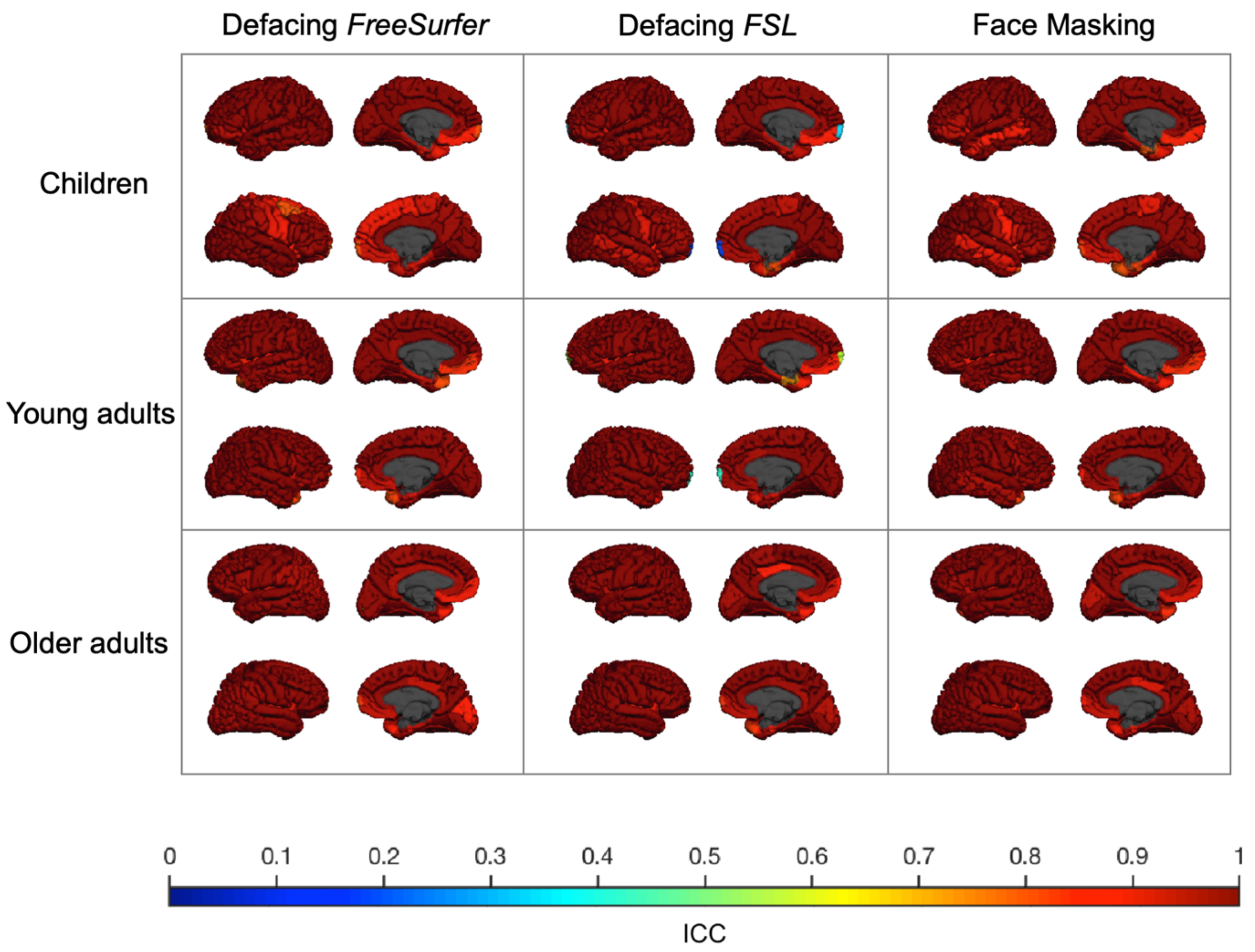

Figure S1. ICC of cortical volume derived from original versus de-identified scans.
The ICC for each sample (children, young adults and older adults) is plotted on the corresponding average scan. Each column shows a different de-identification technique. Within each square, the left hemisphere (top) and the right hemisphere (bottom) are shown from an outer and medial view. The lowest ICC (0.21) was found in the right frontal pole in children using FSL defacing.

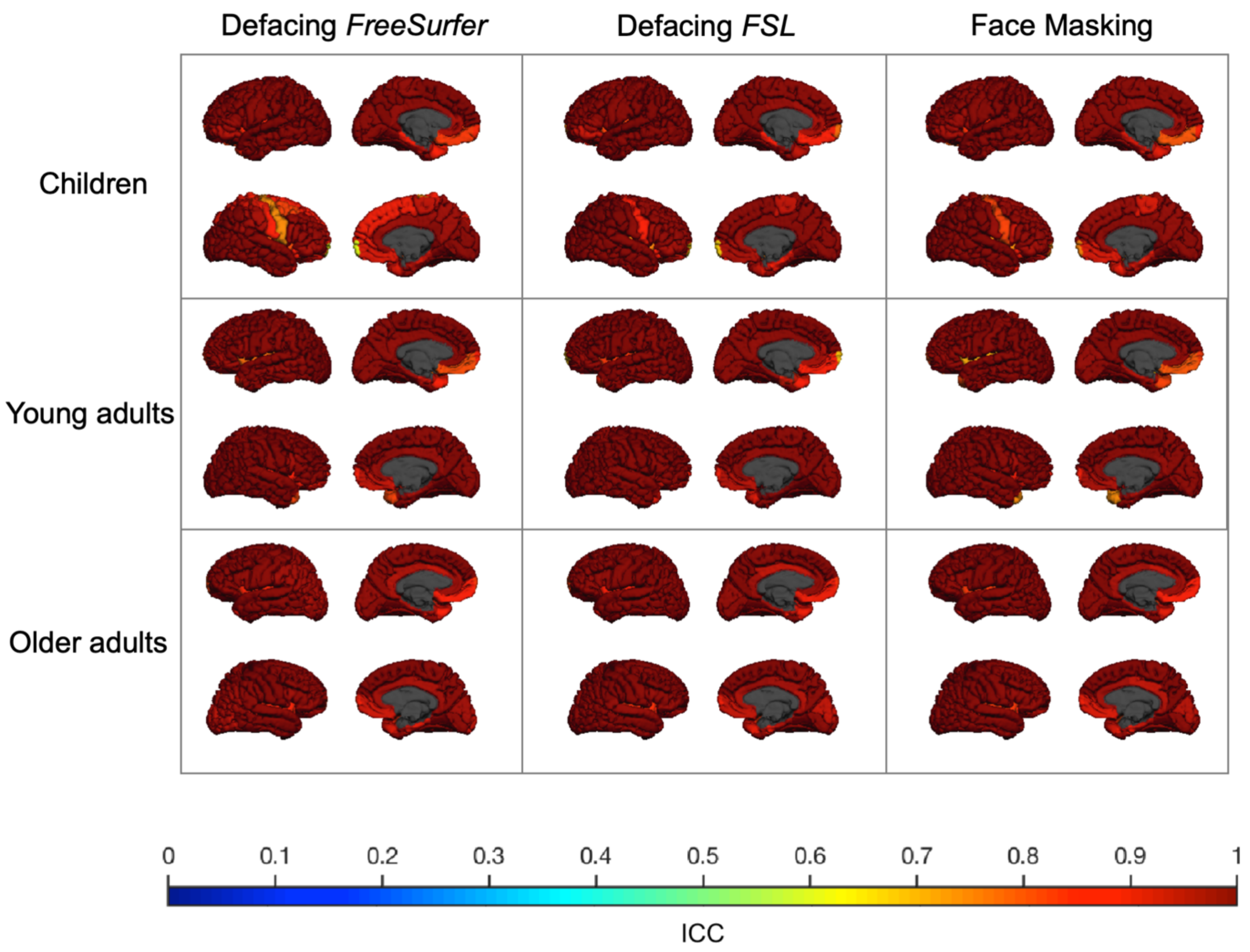

Figure S2. ICC of cortical surface area derived from original versus de-identified scans.
The ICC for each sample (children, young adults and older adults) is plotted on the corresponding average scan. Each column shows a different de-identification technique. Within each square, the left hemisphere (top) and the right hemisphere (bottom) are shown from an outer and medial view. The lowest ICC (0.61) was found in the right frontal pole in children using FreeSurfer defacing.

Table S1. Effects of de-identification on brain measures in children.

Brain metric	Mean (SD)				Coefficient of Variation				Paired t-test t(*p*)			Signed PD in %			Absolute PD (%)			Pearson's r			ICC (95% CI)		
	Orig	FS df	FSL df	FM	Orig	FS df	FSL df	FM	FS df	FSL df	FM	FS df	FSL df	FM	FS df	FSL df	FM	FS df	FSL df	FM	FS df	FSL df	FM
Subcortical volume (ml)																							
Left thalamus	7.43 (0.62)	7.46 (0.61)	7.44 (0.62)	7.43 (0.64)	8.37	8.18	8.29	8.60	-1.06 (0.30)	-0.39 (0.70)	-0.18 (0.86)	0.40 (1.80)	0.16 (1.96)	0.04 (1.53)	1.48 (1.06)	1.35 (1.40)	1.31 (0.73)	0.98	0.97	0.98	0.98 (0.95-0.99)	0.97 (0.94-0.99)	0.98 (0.96-0.99)
Left caudatus	3.98 (0.46)	3.98 (0.45)	3.98 (0.44)	3.97 (0.45)	11.46	11.22	11.10	11.24	-0.09 (0.93)	-0.10 (0.92)	0.38 (0.71)	0.06 (1.80)	0.08 (1.85)	-0.12 (1.91)	1.28 (1.24)	1.28 (1.31)	1.45 (1.22)	0.99	0.99	0.99	0.99 (0.97-1.00)	0.99 (0.97-0.99)	0.99 (0.97-0.99)
Left putamen	5.33 (0.50)	5.35 (0.54)	5.36 (0.51)	5.34 (0.52)	9.37	10.07	9.51	9.75	-1.06 (0.30)	-2.22 (0.04)	-0.63 (0.53)	0.37 (2.25)	0.70 (1.58)	0.20 (1.87)	1.67 (1.52)	1.36 (1.04)	1.28 (1.36)	0.98	0.99	0.98	0.98 (0.95-0.99)	0.98 (0.96-0.99)	0.98 (0.96-0.99)
Left pallidum	1.79 (0.18)	1.77 (0.15)	1.77 (0.16)	1.77 (0.17)	10.10	8.33	8.79	9.79	0.90 (0.38)	1.34 (0.19)	1.34 (0.19)	-0.92 (6.03)	-0.89 (3.49)	-1.04 (3.81)	4.65 (3.84)	2.45 (2.60)	2.89 (2.64)	0.80	0.93	0.92	0.79 (0.58-0.90)	0.92 (0.83-0.96)	0.92 (0.82-0.96)
Left hippocampus	3.95 (0.32)	3.97 (0.31)	3.95 (0.33)	3.93 (0.31)	8.22	7.93	8.34	7.98	-1.30 (0.20)	-0.13 (0.89)	1.24 (0.23)	0.41 (1.42)	0.05 (2.00)	-0.46 (1.98)	1.27 (0.71)	1.58 (1.18)	1.53 (1.30)	0.98	0.97	0.97	0.98 (0.96-0.99)	0.97 (0.94-0.99)	0.97 (0.93-0.99)
Left amygdala	1.61 (0.20)	1.61 (0.18)	1.60 (0.19)	1.58 (0.18)	12.47	11.47	11.93	11.48	0.73 (0.47)	1.12 (0.27)	2.69 (0.01)	-0.43 (4.00)	-0.80 (3.89)	-2.21 (4.36)	3.23 (2.31)	3.19 (2.28)	4.04 (2.66)	0.95	0.95	0.94	0.95 (0.89-0.98)	0.95 (0.89-0.98)	0.92 (0.79-0.97)
Left accumbens	0.70 (0.13)	0.69 (0.12)	0.70 (0.12)	0.71 (0.11)	18.05	17.82	17.41	16.19	1.51 (0.14)	-0.49 (0.63)	-1.24 (0.23)	-1.69 (6.27)	0.90 (8.46)	2.11 (7.58)	5.30 (3.62)	6.84 (4.86)	6.38 (4.45)	0.95	0.88	0.91	0.95 (0.88-0.98)	0.88 (0.75-0.95)	0.91 (0.80-0.96)
Right thalamus	7.23 (0.60)	7.24 (0.61)	7.26 (0.61)	7.26 (0.64)	8.25	8.40	8.44	8.83	-0.34 (0.74)	-1.80 (0.08)	-1.27 (0.22)	0.09 (1.48)	0.43 (1.26)	0.31 (1.40)	1.21 (0.82)	1.06 (0.78)	1.08 (0.92)	0.98	0.99	0.99	0.98 (0.96-0.99)	0.99 (0.97-0.99)	0.99 (0.97-0.99)
Right caudatus	4.13 (0.46)	4.14 (0.46)	4.14 (0.46)	4.13 (0.47)	11.24	11.07	11.21	11.41	-1.41 (0.17)	-1.29 (0.21)	-0.56 (0.58)	0.33 (1.16)	0.31 (1.21)	0.13 (1.87)	0.94 (0.74)	1.05 (0.64)	1.13 (1.49)	1.00	0.99	0.99	0.99 (0.99-1.00)	0.99 (0.99-1.00)	0.99 (0.98-1.00)
Right putamen	5.29 (0.53)	5.32 (0.53)	5.33 (0.51)	5.32 (0.53)	9.99	9.97	9.60	9.98	-1.91 (0.07)	-2.30 (0.03)	-1.91 (0.07)	0.59 (1.59)	0.85 (1.95)	0.59 (1.57)	1.16 (1.21)	1.32 (1.66)	1.29 (1.04)	0.99	0.98	0.99	0.99 (0.97-0.99)	0.98 (0.95-0.99)	0.99 (0.97-0.99)
Right pallidum	1.70 (0.14)	1.71 (0.15)	1.70 (0.14)	1.69 (0.15)	8.48	8.86	8.27	8.66	-1.83 (0.08)	0.08 (0.93)	0.86 (0.40)	0.88 (2.48)	-0.03 (2.34)	-0.34 (1.87)	1.93 (1.76)	1.78 (1.48)	1.50 (1.13)	0.96	0.96	0.98	0.95 (0.90-0.98)	0.96 (0.91-0.98)	0.98 (0.95-0.99)
Right hippocampus	4.01 (0.30)	4.01 (0.31)	4.01 (0.32)	3.99 (0.31)	7.57	7.67	8.02	7.82	0.00 (1.00)	-0.19 (0.85)	1.09 (0.28)	0.00 (2.08)	0.04 (2.10)	-0.47 (2.04)	1.53 (1.37)	1.50 (1.44)	1.62 (1.28)	0.96	0.97	0.96	0.96 (0.92-0.98)	0.96 (0.92-0.98)	0.96 (0.92-0.98)
Right amygdala	1.72 (0.20)	1.69 (0.21)	1.71 (0.19)	1.68 (0.19)	11.86	12.16	11.29	11.49	2.47 (0.02)	0.83 (0.41)	3.12 (0.00)	-2.07 (3.86)	-0.53 (3.37)	-2.37 (3.76)	3.26 (2.88)	2.92 (1.67)	3.41 (2.81)	0.94	0.96	0.95	0.93 (0.83-0.97)	0.96 (0.90-0.98)	0.93 (0.78-0.97)
Right accumbens	0.66 (0.10)	0.66 (0.10)	0.68 (0.11)	0.67 (0.11)	14.85	15.21	16.13	16.41	-0.10 (0.92)	-2.01 (0.06)	-0.81 (0.42)	0.12 (7.85)	2.08 (5.56)	0.93 (6.84)	6.03 (4.88)	4.83 (3.33)	5.79 (3.57)	0.87	0.94	0.90	0.87 (0.73-0.94)	0.93 (0.83-0.97)	0.90 (0.79-0.95)
Ventricular volume (ml)																							
Third ventricle	0.83 (0.27)	0.83 (0.26)	0.83 (0.27)	0.83 (0.26)	32.48	31.80	32.72	31.97	-0.47 (0.64)	0.22 (0.83)	0.06 (0.95)	0.38 (2.98)	-0.23 (3.30)	0.04 (3.57)	2.11 (2.10)	2.29 (2.33)	2.70 (2.27)	1.00	1.00	0.99	1.00 (0.99-1.00)	1.00 (0.99-1.00)	1.00 (0.99-1.00)
Left lateral ventricle	6.63 (3.84)	6.65 (3.85)	6.66 (3.85)	6.64 (3.85)	57.88	57.88	57.84	57.91	-1.31 (0.20)	-2.03 (0.05)	-0.82 (0.42)	0.33 (1.80)	0.58 (1.75)	0.27 (2.51)	1.21 (1.35)	1.10 (1.46)	1.29 (2.15)	1.00	1.00	1.00	1.00 (1.00-1.00)	1.00 (1.00-1.00)	1.00 (1.00-1.00)
Left inferior lateral ventricle	0.42 (0.18)	0.41 (0.16)	0.40 (0.17)	0.40 (0.15)	41.40	40.39	42.40	37.19	2.16 (0.04)	3.34 (0.00)	2.22 (0.04)	-3.81 (11.60)	-7.18 (12.34)	-3.68 (11.75)	8.00 (9.11)	11.19 (8.70)	9.03 (8.21)	0.98	0.97	0.97	0.98 (0.94-0.99)	0.96 (0.87-0.99)	0.96 (0.89-0.98)
Right lateral ventricle	6.05 (2.63)	6.06 (2.61)	6.06 (2.62)	6.04 (2.61)	43.41	43.14	43.29	43.21	-0.79 (0.44)	-0.90 (0.37)	0.64 (0.53)	0.36 (1.66)	0.23 (0.97)	0.00 (1.27)	1.01 (1.35)	0.75 (0.64)	0.91 (0.87)	1.00	1.00	1.00	1.00 (1.00-1.00)	1.00 (1.00-1.00)	1.00 (1.00-1.00)
Right inferior lateral ventricle	0.41 (0.11)	0.41 (0.11)	0.41 (0.13)	0.40 (0.12)	27.55	26.60	31.59	28.62	0.01 (0.99)	-0.13 (0.90)	0.40 (0.69)	0.29 (5.70)	-1.17 (12.65)	-1.03 (8.27)	4.26 (3.69)	7.58 (10.08)	5.10 (6.51)	0.98	0.94	0.97	0.98 (0.96-0.99)	0.93 (0.86-0.97)	0.97 (0.93-0.99)
Local cortical volume (ml)																							
Left banks superior temporal sulcus	3.00 (0.41)	3.01 (0.48)	3.01 (0.42)	2.96 (0.41)	13.74	16.07	14.09	13.92	-0.37 (0.71)	-0.66 (0.52)	1.36 (0.19)	0.05 (5.08)	0.37 (3.43)	-1.27 (4.52)	3.82 (3.26)	2.67 (2.11)	3.83 (2.61)	0.95	0.97	0.94	0.94 (0.88-0.98)	0.97 (0.93-0.99)	0.94 (0.87-0.97)
Left caudal anterior cingulate cortex	2.14 (0.47)	2.14 (0.50)	2.16 (0.48)	2.16 (0.49)	22.01	23.39	22.40	22.81	0.27 (0.79)	-0.99 (0.33)	-1.23 (0.23)	-0.46 (2.94)	0.84 (5.23)	0.58 (3.15)	2.24 (1.91)	3.19 (4.18)	2.24 (2.25)	0.99	0.97	0.99	0.99 (0.98-1.00)	0.97 (0.94-0.99)	0.99 (0.98-1.00)
Left caudal middle frontal gyrus	8.23 (1.50)	8.18 (1.55)	8.22 (1.61)	8.29 (1.54)	18.24	18.92	19.59	18.62	0.88 (0.39)	0.11 (0.91)	-1.36 (0.19)	-0.64 (3.05)	-0.24 (2.98)	0.73 (3.03)	2.31 (2.04)	2.31 (1.84)	2.34 (2.01)	0.99	0.99	0.99	0.99 (0.97-0.99)	0.99 (0.97-0.99)	0.99 (0.97-0.99)
Left cuneus	4.21 (0.65)	4.19 (0.64)	4.19 (0.64)	4.20 (0.65)	15.41	15.18	15.25	15.48	0.69 (0.50)	1.13 (0.27)	0.25 (0.81)	-0.30 (2.35)	-0.40 (1.93)	-0.10 (2.20)	1.85 (1.43)	1.48 (1.26)	1.80 (1.21)	0.99	0.99	0.99	0.99 (0.97-0.99)	0.99 (0.98-1.00)	0.99 (0.98-1.00)
Left entorhinal cortex	2.11 (0.34)	2.09 (0.37)	2.12 (0.41)	2.13 (0.45)	16.11	17.68	19.23	20.91	0.49 (0.63)	-0.15 (0.88)	-0.37 (0.71)	-1.04 (9.12)	-0.04 (8.85)	0.16 (11.73)	6.38 (6.47)	6.94 (5.32)	8.30 (8.12)	0.85	0.87	0.81	0.85 (0.70-0.93)	0.86 (0.71-0.94)	0.78 (0.57-0.90)
Left fusiform gyrus	11.89 (1.10)	11.79 (1.05)	11.76 (1.13)	11.79 (1.12)	9.29	8.89	9.65	9.50	1.69 (0.10)	2.17 (0.04)	1.61 (0.12)	-0.82 (2.38)	-1.14 (2.42)	-0.89 (2.59)	1.68 (1.84)	1.89 (1.86)	1.93 (1.91)	0.96	0.96	0.96	0.96 (0.91-0.98)	0.96 (0.90-0.98)	0.95 (0.90-0.98)
Left inferior parietal cortex	16.52 (2.04)	16.51 (2.08)	16.47 (1.98)	16.53 (1.99)	12.36	12.62	12.04	12.04	0.30 (0.77)	0.99 (0.33)	-0.14 (0.89)	-0.15 (1.93)	-0.28 (1.58)	0.10 (1.93)	1.50 (1.19)	1.22 (1.00)	1.38 (1.32)	0.99	0.99	0.99	0.99 (0.97-0.99)	0.99 (0.98-1.00)	0.99 (0.97-0.99)
Left inferior temporal gyrus	13.44 (1.94)	13.39 (2.00)	13.41 (1.93)	13.28 (1.85)	14.45	14.94	14.40	13.89	1.03 (0.31)	0.53 (0.60)	2.70 (0.01)	-0.45 (2.08)	-0.18 (1.74)	-1.09 (2.27)	1.38 (1.60)	1.39 (1.03)	2.11 (1.32)	0.99	0.99	0.99	0.99 (0.98-1.00)	0.99 (0.98-1.00)	0.99 (0.96-0.99)
Left isthmus cingulate cortex	3.34 (0.63)	3.35 (0.64)	3.36 (0.63)	3.35 (0.65)	18.81	18.95	18.77	19.45	-0.55 (0.59)	-0.92 (0.37)	-0.22 (0.83)	0.27 (3.21)	0.44 (2.55)	0.04 (3.09)	2.58 (1.86)	2.06 (1.51)	2.32 (1.99)	0.99	0.99	0.99	0.99 (0.97-0.99)	0.99 (0.98-1.00)	0.99 (0.97-0.99)
Left lateral occipital cortex	15.90 (2.16)	15.83 (2.24)	15.91 (2.14)	15.92 (2.23)	13.58	14.14	13.48	14.02	1.40 (0.17)	-0.08 (0.94)	-0.36 (0.72)	-0.52 (1.71)	0.04 (1.50)	0.06 (1.64)	1.37 (1.12)	1.22 (0.83)	1.30 (0.97)	0.99	0.99	0.99	0.99 (0.98-1.00)	0.99 (0.99-1.00)	0.99 (0.98-1.00)
Left lateral orbitofrontal cortex	9.49 (0.91)	9.45 (0.92)	9.40 (0.93)	9.49 (0.97)	9.61	9.75	9.92	10.23	0.96 (0.35)	1.92 (0.07)	-0.23 (0.82)	-0.42 (2.12)	-0.94 (2.41)	0.01 (1.45)	1.81 (1.12)	2.11 (1.44)	1.08 (0.95)	0.98	0.97	0.99	0.98 (0.95-0.99)	0.97 (0.92-0.99)	0.99 (0.98-1.00)
Left lingual gyrus	8.59 (1.19)	8.57 (1.15)	8.61 (1.21)	8.63 (1.21)	13.83	13.38	14.08	13.96	0.36 (0.72)	-0.53 (0.60)	-1.20 (0.24)	-0.09 (1.81)	0.21 (2.16)	0.51 (2.10)	1.37 (1.15)	1.49 (1.55)	1.37 (1.65)	0.99	0.99	0.99	0.99 (0.98-1.00)	0.99 (0.97-0.99)	0.99 (0.97-0.99)
Left medial orbitofrontal cortex	6.12 (0.67)	6.35 (0.81)	6.21 (0.79)	6.26 (0.88)	11.02	12.74	12.72	14.05	-3.52 (0.00)	-1.20 (0.24)	-1.51 (0.14)	3.45 (5.19)	1.19 (5.90)	1.87 (7.18)	5.00 (3.65)	4.27 (4.16)	5.61 (4.73)	0.92	0.90	0.86	0.87 (0.59-0.95)	0.88 (0.76-0.95)	0.83 (0.65-0.92)

Left middle temporal gyrus	13.86 (1.51)	13.83 (1.47)	13.70 (1.40)	13.31 (1.52)	10.88	10.65	10.19	11.39	0.40 (0.69)	2.58 (0.02)	4.77 (0.00)	-0.16 (2.19)	-1.09 (2.08)	-4.10 (4.39)	1.82 (1.16)	1.73 (1.56)	5.00 (3.28)	0.98	0.98	0.93	0.98 (0.95-0.99)	0.97 (0.93-0.99)	0.87 (0.40-0.96)
Left parahippocampal gyrus	2.47 (0.32)	2.50 (0.34)	2.48 (0.34)	2.51 (0.33)	12.95	13.59	13.61	13.19	-1.21 (0.24)	-0.38 (0.71)	-1.75 (0.09)	0.95 (4.04)	0.30 (4.55)	1.48 (4.01)	3.20 (2.56)	3.54 (2.78)	3.50 (2.37)	0.95	0.94	0.95	0.95 (0.88-0.98)	0.94 (0.87-0.97)	0.94 (0.87-0.97)
Left paracentral lobule	4.29 (0.57)	4.28 (0.58)	4.30 (0.56)	4.30 (0.57)	13.23	13.48	13.08	13.29	0.22 (0.83)	-0.62 (0.54)	-0.77 (0.45)	-0.15 (2.50)	0.33 (2.45)	0.39 (2.57)	1.97 (1.49)	2.08 (1.27)	2.03 (1.58)	0.98	0.98	0.98	0.98 (0.96-0.99)	0.98 (0.96-0.99)	0.98 (0.96-0.99)
Left pars opercularis	6.13 (0.74)	6.11 (0.74)	6.09 (0.76)	6.12 (0.73)	12.15	12.18	12.46	11.88	0.54 (0.60)	1.31 (0.20)	0.21 (0.84)	-0.30 (2.78)	-0.66 (2.35)	-0.10 (3.07)	2.18 (1.70)	2.02 (1.31)	1.97 (2.33)	0.97	0.98	0.96	0.98 (0.94-0.99)	0.98 (0.96-0.99)	0.96 (0.92-0.98)
Left pars orbitalis	3.19 (0.41)	3.17 (0.45)	3.20 (0.42)	3.11 (0.44)	12.93	14.07	13.14	14.27	1.03 (0.31)	-0.34 (0.74)	3.19 (0.00)	-1.00 (4.29)	0.23 (3.57)	-2.80 (4.23)	3.60 (2.44)	2.89 (2.02)	4.11 (2.91)	0.95	0.96	0.96	0.95 (0.90-0.98)	0.96 (0.92-0.98)	0.94 (0.80-0.98)
Left pars triangularis	4.71 (0.74)	4.73 (0.74)	4.72 (0.72)	4.60 (0.75)	15.73	15.65	15.14	16.30	-0.86 (0.40)	-0.58 (0.57)	2.04 (0.05)	0.67 (3.69)	0.48 (3.35)	-2.31 (5.69)	2.92 (2.28)	2.57 (2.15)	4.55 (4.04)	0.98	0.98	0.94	0.98 (0.95-0.99)	0.98 (0.96-0.99)	0.94 (0.85-0.97)
Left pericalcarine cortex	2.66 (0.49)	2.66 (0.49)	2.66 (0.50)	2.67 (0.49)	18.30	18.29	18.76	18.40	-0.08 (0.94)	-0.16 (0.88)	-0.54 (0.60)	0.03 (2.41)	-0.03 (1.92)	0.30 (3.58)	1.95 (1.36)	1.42 (1.26)	2.80 (2.19)	0.99	0.99	0.98	0.99 (0.98-1.00)	0.99 (0.99-1.00)	0.98 (0.96-0.99)
Left postcentral gyrus	12.09 (1.38)	12.07 (1.37)	12.10 (1.38)	12.10 (1.43)	11.38	11.37	11.37	11.79	0.47 (0.64)	-0.34 (0.74)	-0.23 (0.82)	-0.11 (1.36)	0.12 (1.65)	0.03 (1.68)	0.95 (0.96)	1.30 (0.99)	1.32 (1.01)	0.99	0.99	0.99	0.99 (0.99-1.00)	0.99 (0.98-1.00)	0.99 (0.98-1.00)
Left posterior cingulate cortex	3.89 (0.69)	3.89 (0.69)	3.91 (0.70)	3.91 (0.71)	17.78	17.81	17.95	18.23	-0.14 (0.89)	-0.58 (0.56)	-0.95 (0.35)	0.07 (2.48)	0.36 (3.03)	0.50 (2.69)	2.04 (1.35)	2.36 (1.87)	2.16 (1.63)	0.99	0.98	0.99	0.99 (0.98-1.00)	0.98 (0.97-0.99)	0.99 (0.97-0.99)
Left precentral gyrus	15.50 (1.48)	15.45 (1.52)	15.47 (1.49)	15.46 (1.55)	9.55	9.82	9.64	10.06	1.36 (0.19)	0.71 (0.48)	1.01 (0.32)	-0.35 (1.16)	-0.18 (1.23)	-0.32 (1.38)	1.00 (0.66)	0.95 (0.77)	1.15 (0.80)	0.99	0.99	0.99	0.99 (0.98-1.00)	0.99 (0.98-1.00)	0.99 (0.98-1.00)
Left precuneus	12.93 (1.62)	12.91 (1.59)	12.90 (1.62)	12.96 (1.58)	12.53	12.31	12.52	12.21	0.64 (0.53)	0.95 (0.35)	-0.85 (0.41)	-0.11 (1.12)	-0.23 (1.28)	0.27 (1.39)	0.81 (0.76)	1.12 (0.62)	1.17 (0.76)	1.00	1.00	0.99	1.00 (0.99-1.00)	1.00 (0.99-1.00)	0.99 (0.99-1.00)
Left rostral anterior cingulate cortex	3.18 (0.55)	3.19 (0.58)	3.24 (0.59)	3.18 (0.54)	17.34	18.22	18.21	16.99	-0.38 (0.71)	-2.47 (0.02)	-0.21 (0.84)	0.17 (4.50)	1.71 (3.96)	0.19 (4.07)	3.52 (2.72)	3.77 (1.98)	3.25 (2.37)	0.97	0.98	0.98	0.97 (0.93-0.99)	0.97 (0.93-0.99)	0.98 (0.95-0.99)
Left rostral middle frontal gyrus	20.88 (2.94)	20.94 (3.03)	20.70 (2.83)	20.51 (2.82)	14.09	14.46	13.65	13.74	-0.76 (0.45)	1.70 (0.10)	3.73 (0.00)	0.24 (1.94)	-0.80 (2.47)	-1.72 (2.35)	1.54 (1.16)	2.00 (1.62)	2.25 (1.83)	0.99	0.98	0.99	0.99 (0.98-1.00)	0.98 (0.96-0.99)	0.98 (0.91-0.99)
Left superior frontal gyrus	28.06 (3.45)	28.01 (3.45)	27.78 (3.59)	28.13 (3.59)	12.30	12.31	12.93	12.76	0.61 (0.55)	2.54 (0.02)	-0.80 (0.43)	-0.17 (1.51)	-1.07 (2.01)	0.22 (1.55)	1.19 (0.92)	1.64 (1.55)	1.13 (1.05)	0.99	0.99	0.99	0.99 (0.98-1.00)	0.99 (0.96-0.99)	0.99 (0.98-1.00)
Left superior parietal cortex	18.05 (2.33)	17.96 (2.40)	18.09 (2.39)	18.02 (2.35)	12.89	13.35	13.20	13.07	1.94 (0.06)	-0.62 (0.54)	0.66 (0.51)	-0.58 (1.42)	0.18 (1.73)	-0.24 (1.59)	1.10 (1.05)	1.40 (1.00)	1.27 (0.94)	1.00	0.99	0.99	0.99 (0.99-1.00)	0.99 (0.98-1.00)	0.99 (0.98-1.00)
Left superior temporal gyrus	15.21 (1.98)	15.23 (1.93)	15.20 (1.93)	15.20 (1.86)	13.03	12.65	12.70	12.21	-0.39 (0.70)	0.22 (0.83)	0.14 (0.89)	0.19 (1.98)	-0.03 (1.60)	0.03 (2.36)	1.46 (1.32)	1.18 (1.06)	1.84 (1.42)	0.99	0.99	0.98	0.99 (0.97-0.99)	0.99 (0.98-1.00)	0.98 (0.96-0.99)
Left supramarginal gyrus	15.45 (2.95)	15.40 (2.98)	15.49 (3.02)	15.52 (3.09)	19.13	19.32	19.49	19.91	0.69 (0.49)	-0.74 (0.47)	-1.17 (0.25)	-0.27 (1.96)	0.21 (1.71)	0.33 (1.90)	1.59 (1.14)	1.23 (1.18)	1.48 (1.20)	0.99	1.00	1.00	0.99 (0.99-1.00)	1.00 (0.99-1.00)	0.99 (0.99-1.00)
Left frontal pole	1.34 (0.20)	1.30 (0.18)	1.13 (0.23)	1.31 (0.20)	14.95	13.62	20.18	15.32	1.66 (0.11)	4.67 (0.00)	1.53 (0.14)	-2.80 (9.76)	-17.47 (21.22)	-2.24 (7.38)	7.51 (6.68)	18.33 (20.45)	5.67 (5.12)	0.81	0.49	0.88	0.79 (0.58-0.90)	0.34 (-0.07-0.65)	0.88 (0.75-0.94)
Left temporal pole	2.78 (0.54)	2.84 (0.47)	2.85 (0.50)	2.83 (0.46)	19.51	16.55	17.72	16.18	-1.22 (0.24)	-1.81 (0.08)	-1.12 (0.27)	2.43 (8.89)	2.55 (7.10)	2.31 (8.22)	5.81 (7.07)	5.21 (5.38)	6.08 (5.89)	0.91	0.95	0.92	0.90 (0.79-0.95)	0.94 (0.87-0.97)	0.91 (0.80-0.96)
Left transverse temporal gyrus	1.51 (0.31)	1.52 (0.32)	1.52 (0.30)	1.53 (0.34)	20.70	21.32	19.55	21.93	-0.83 (0.42)	-1.09 (0.29)	-1.60 (0.12)	0.53 (3.75)	0.91 (3.36)	1.00 (3.68)	2.79 (2.50)	2.89 (1.86)	3.04 (2.22)	0.98	0.99	0.99	0.98 (0.96-0.99)	0.99 (0.97-0.99)	0.98 (0.96-0.99)
Left insula	8.09 (0.87)	8.00 (0.81)	8.09 (0.85)	8.14 (0.86)	10.77	10.15	10.52	10.58	1.49 (0.15)	0.01 (0.99)	-0.63 (0.53)	-1.05 (3.69)	0.02 (4.24)	0.58 (4.38)	2.73 (2.65)	3.13 (2.78)	3.38 (2.76)	0.94	0.92	0.92	0.93 (0.85-0.97)	0.92 (0.83-0.96)	0.92 (0.82-0.96)
Right banks superior temporal sulcus	2.68 (0.42)	2.66 (0.46)	2.69 (0.44)	2.65 (0.42)	15.61	17.44	16.53	15.81	0.44 (0.66)	-0.37 (0.71)	0.92 (0.37)	-0.75 (5.11)	0.20 (4.25)	-0.94 (4.87)	4.08 (3.06)	3.20 (2.73)	3.99 (2.84)	0.95	0.96	0.95	0.94 (0.88-0.97)	0.96 (0.91-0.98)	0.95 (0.89-0.98)
Right caudal anterior cingulate cortex	2.39 (0.50)	2.39 (0.50)	2.40 (0.50)	2.39 (0.51)	20.85	20.93	20.68	21.28	-0.39 (0.70)	-1.18 (0.25)	-0.57 (0.57)	0.23 (3.46)	0.79 (2.94)	0.22 (2.70)	2.48 (2.37)	2.49 (1.68)	1.95 (1.83)	0.99	0.99	0.99	0.99 (0.97-0.99)	0.99 (0.97-0.99)	0.99 (0.98-1.00)
Right caudal middle frontal gyrus	8.11 (1.03)	8.00 (1.00)	8.09 (1.06)	8.17 (1.06)	12.70	12.50	13.09	12.97	0.75 (0.46)	0.53 (0.60)	-1.60 (0.12)	-1.23 (8.50)	-0.27 (2.13)	0.75 (2.43)	3.32 (7.90)	1.58 (1.42)	1.85 (1.71)	0.77	0.99	0.98	0.78 (0.56-0.89)	0.99 (0.97-0.99)	0.98 (0.96-0.99)
Right cuneus	4.59 (1.04)	4.60 (1.03)	4.59 (1.02)	4.59 (1.07)	22.60	22.49	22.28	23.22	-0.28 (0.78)	0.20 (0.85)	0.02 (0.98)	0.16 (2.48)	-0.02 (2.18)	-0.13 (2.50)	1.95 (1.49)	1.73 (1.28)	1.97 (1.49)	0.99	1.00	0.99	0.99 (0.98-1.00)	1.00 (0.99-1.00)	0.99 (0.99-1.00)
Right entorhinal cortex	2.09 (0.32)	2.12 (0.34)	2.16 (0.31)	2.16 (0.32)	15.37	16.18	14.45	14.88	-1.16 (0.26)	-1.59 (0.12)	-1.83 (0.08)	1.52 (6.93)	3.20 (10.00)	3.44 (9.87)	5.27 (4.63)	7.12 (7.60)	7.24 (7.43)	0.91	0.80	0.81	0.91 (0.80-0.96)	0.79 (0.58-0.90)	0.80 (0.60-0.91)
Right fusiform gyrus	11.33 (1.43)	11.31 (1.51)	11.29 (1.46)	11.29 (1.49)	12.65	13.38	12.90	13.16	0.40 (0.69)	0.75 (0.46)	1.04 (0.31)	-0.26 (2.24)	-0.44 (2.84)	-0.45 (1.90)	1.79 (1.31)	1.87 (2.15)	1.58 (1.11)	0.99	0.98	0.99	0.98 (0.97-0.99)	0.98 (0.95-0.99)	0.99 (0.98-1.00)
Right inferior parietal cortex	20.01 (3.05)	20.08 (3.07)	20.11 (3.08)	20.14 (3.05)	15.22	15.26	15.32	15.14	-0.64 (0.53)	-1.31 (0.20)	-1.49 (0.15)	0.36 (2.62)	0.49 (1.83)	0.64 (2.06)	1.74 (1.95)	1.27 (1.40)	1.64 (1.37)	0.98	0.99	0.99	0.98 (0.97-0.99)	0.99 (0.98-1.00)	0.99 (0.98-1.00)
Right inferior temporal gyrus	12.39 (1.97)	12.46 (1.95)	12.45 (1.94)	12.43 (1.95)	15.92	15.63	15.60	15.65	-1.34 (0.19)	-1.30 (0.21)	-0.76 (0.45)	0.58 (2.01)	0.49 (1.64)	0.38 (2.31)	1.72 (1.15)	1.34 (1.03)	1.88 (1.33)	0.99	0.99	0.99	0.99 (0.98-1.00)	0.99 (0.99-1.00)	0.99 (0.98-1.00)
Right isthmus cingulate cortex	3.33 (0.63)	3.36 (0.63)	3.33 (0.62)	3.33 (0.64)	18.82	18.64	18.55	19.09	-1.76 (0.09)	-0.14 (0.89)	-0.06 (0.95)	0.79 (2.50)	0.08 (2.54)	-0.02 (2.53)	1.99 (1.66)	1.92 (1.62)	1.92 (1.59)	0.99	0.99	0.99	0.99 (0.98-1.00)	0.99 (0.98-1.00)	0.99 (0.98-1.00)
Right lateral occipital cortex	16.05 (2.09)	16.10 (2.21)	16.11 (2.14)	16.11 (2.15)	13.04	13.75	13.27	13.36	-0.60 (0.56)	-1.35 (0.19)	-1.03 (0.32)	0.19 (1.94)	0.36 (1.26)	0.30 (1.55)	1.36 (1.37)	1.00 (0.81)	1.18 (1.02)	0.99	0.99	0.99	0.99 (0.97-0.99)	0.99 (0.99-1.00)	0.99 (0.98-1.00)
Right lateral orbitofrontal cortex	9.25 (1.21)	9.18 (1.05)	9.10 (1.07)	9.06 (1.06)	13.07	11.42	11.71	11.71	0.73 (0.47)	1.60 (0.12)	2.70 (0.01)	-0.57 (4.84)	-1.54 (4.87)	-1.98 (3.62)	3.95 (2.75)	3.89 (3.22)	3.32 (2.38)	0.92	0.92	0.96	0.91 (0.81-0.96)	0.91 (0.80-0.96)	0.94 (0.83-0.97)
Right lingual gyrus	9.13 (1.58)	9.14 (1.59)	9.11 (1.56)	9.10 (1.56)	17.34	17.34	17.17	17.09	-0.47 (0.64)	0.82 (0.42)	0.95 (0.35)	0.12 (1.36)	-0.20 (1.25)	-0.25 (1.37)	1.14 (0.73)	1.06 (0.67)	1.10 (0.83)	1.00	1.00	1.00	1.00 (0.99-1.00)	1.00 (0.99-1.00)	1.00 (0.99-1.00)
Right medial orbitofrontal cortex	6.65 (0.76)	6.70 (0.81)	6.61 (0.73)	6.68 (0.80)	11.38	12.14	11.04	12.04	-0.92 (0.37)	0.97 (0.34)	-0.50 (0.62)	0.72 (4.27)	-0.57 (3.03)	0.39 (4.41)	3.14 (2.92)	2.37 (1.90)	3.41 (2.73)	0.93	0.96	0.93	0.93 (0.85-0.97)	0.96 (0.91-0.98)	0.93 (0.85-0.97)
Right middle temporal gyrus	15.49 (1.74)	15.55 (1.65)	15.29 (1.61)	14.95 (1.67)	11.22	10.61	10.56	11.14	-1.16 (0.26)	1.57 (0.13)	4.56 (0.00)	0.48 (1.88)	-1.17 (3.83)	-3.48 (3.77)	1.51 (1.19)	2.40 (3.18)	3.96 (3.24)	0.99	0.94	0.94	0.99 (0.97-0.99)	0.93 (0.84-0.97)	0.90 (0.51-0.97)
Right parahippocampal gyrus	2.21 (0.24)	2.20 (0.27)	2.23 (0.21)	2.22 (0.23)	10.64	12.19	9.36	10.44	0.73 (0.48)	-0.87 (0.39)	-0.32 (0.75)	-0.91 (5.13)	0.88 (4.40)	0.36 (5.46)	4.28 (2.85)	3.59 (2.58)	4.30 (3.27)	0.91	0.91	0.87	0.91 (0.80-0.96)	0.91 (0.80-0.96)	0.87 (0.74-0.94)
Right paracentral lobule	4.64 (0.50)	4.64 (0.56)	4.64 (0.56)	4.58 (0.59)	10.77	12.00	12.10	12.80	0.03 (0.98)	0.16 (0.87)	1.32 (0.20)	-0.17 (4.77)	-0.28 (4.31)	-1.53 (5.44)	2.45 (4.07)	2.45 (3.53)	3.19 (4.63)	0.93	0.95	0.93	0.93 (0.85-0.97)	0.94 (0.88-0.97)	0.91 (0.81-0.96)
Right pars opercularis	5.26 (0.55)	5.27 (0.58)	5.27 (0.58)	5.29 (0.60)	10.51	10.93	11.03	11.42	-0.31 (0.76)	-0.60 (0.55)	-0.78 (0.44)	0.13 (3.11)	0.23 (2.45)	0.58 (4.57)	2.19 (2.17)	1.93 (1.47)	3.46 (2.96)	0.96	0.98	0.92	0.96 (0.92-0.98)	0.98 (0.95-0.99)	0.92 (0.83-0.96)
Right pars orbitalis	3.66 (0.44)	3.65 (0.48)	3.68 (0.47)	3.59 (0.48)	11.89	13.07	12.82	13.50	0.73 (0.47)	-0.72 (0.48)	2.74 (0.01)	-0.53 (2.73)	0.33 (3.07)	-2.19 (3.75)	2.15 (1.72)	2.42 (1.85)	3.57 (2.41)	0.98	0.97	0.96	0.97 (0.94-0.99)	0.97 (0.93-0.99)	0.95 (0.86-0.98)
Right pars triangularis	5.68 (0.90)	5.64 (0.84)	5.70 (0.88)	5.61 (0.91)	15.79	14.94	15.48	16.27	1.18 (0.25)	-0.76 (0.45)	1.79 (0.09)	-0.58 (3.26)	0.47 (2.93)	-1.35 (3.86)	2.35 (2.27)	2.26 (1.87)	2.84 (2.90)	0.98	0.98	0.98	0.98 (0.96-0.99)	0.98 (0.96-0.99)	0.97 (0.94-0.99)

Right pericalcarine cortex	2.92 (0.48)	2.94 (0.49)	2.91 (0.47)	2.92 (0.48)	16.27	16.82	16.08	16.38	-0.78 (0.44)	0.99 (0.33)	0.13 (0.90)	0.34 (2.71)	-0.45 (2.49)	-0.12 (3.40)	2.29 (1.42)	2.01 (1.48)	2.57 (2.17)	0.99	0.99	0.98	0.99 (0.97-0.99)	0.99 (0.98-1.00)	0.98 (0.95-0.99)
Right postcentral gyrus	11.30 (1.33)	11.45 (1.20)	11.31 (1.30)	11.24 (1.50)	11.81	10.47	11.45	13.35	-1.47 (0.16)	-0.18 (0.86)	0.72 (0.48)	1.50 (5.22)	0.12 (1.85)	-0.76 (4.68)	2.21 (4.95)	1.52 (1.01)	2.29 (4.12)	0.92	0.99	0.97	0.91 (0.81-0.96)	0.99 (0.97-0.99)	0.96 (0.92-0.98)
Right posterior cingulate cortex	4.11 (0.72)	4.09 (0.70)	4.11 (0.70)	4.11 (0.70)	17.49	17.17	16.93	17.10	0.76 (0.45)	0.13 (0.90)	0.17 (0.86)	-0.47 (3.38)	0.02 (2.56)	-0.02 (3.11)	2.13 (2.63)	1.89 (1.68)	2.36 (1.95)	0.98	0.99	0.99	0.98 (0.95-0.99)	0.99 (0.97-0.99)	0.99 (0.97-0.99)
Right precentral gyrus	15.61 (1.46)	15.42 (1.32)	15.45 (1.29)	15.45 (1.33)	9.38	8.55	8.37	8.58	1.60 (0.12)	1.49 (0.15)	1.16 (0.26)	-1.17 (3.75)	-0.93 (3.23)	-0.96 (4.28)	1.99 (3.37)	1.73 (2.87)	2.01 (3.88)	0.91	0.93	0.88	0.90 (0.78-0.95)	0.92 (0.83-0.97)	0.88 (0.74-0.94)
Right precuneus	13.64 (1.64)	13.60 (1.62)	13.62 (1.64)	13.61 (1.54)	11.99	11.92	12.06	11.35	0.49 (0.63)	0.32 (0.75)	0.33 (0.74)	-0.26 (2.91)	-0.11 (1.72)	-0.10 (2.59)	1.59 (2.43)	1.28 (1.12)	1.79 (1.84)	0.97	0.99	0.98	0.97 (0.94-0.99)	0.99 (0.98-1.00)	0.98 (0.95-0.99)
Right rostral anterior cingulate cortex	2.55 (0.53)	2.51 (0.52)	2.55 (0.57)	2.59 (0.55)	20.62	20.85	22.24	21.15	2.60 (0.02)	0.32 (0.75)	-1.52 (0.14)	-1.88 (3.54)	-0.65 (4.03)	1.30 (5.08)	3.09 (2.50)	3.08 (2.60)	4.13 (3.12)	0.99	0.99	0.98	0.98 (0.95-0.99)	0.99 (0.97-0.99)	0.97 (0.94-0.99)
Right rostral middle frontal gyrus	21.69 (2.64)	21.64 (2.61)	21.40 (2.46)	21.49 (2.58)	12.16	12.05	11.50	12.01	0.50 (0.62)	2.05 (0.05)	1.90 (0.07)	-0.21 (2.35)	-1.27 (3.26)	-0.89 (2.38)	1.62 (1.68)	2.67 (2.21)	1.98 (1.55)	0.98	0.96	0.98	0.98 (0.96-0.99)	0.96 (0.90-0.98)	0.98 (0.95-0.99)
Right superior frontal gyrus	28.07 (3.46)	27.94 (3.51)	27.92 (3.59)	28.18 (3.47)	12.34	12.55	12.87	12.31	0.38 (0.70)	0.89 (0.38)	-0.66 (0.52)	-0.45 (5.60)	-0.59 (2.92)	0.40 (2.93)	2.52 (4.99)	1.90 (2.26)	1.68 (2.41)	0.89	0.97	0.97	0.90 (0.78-0.95)	0.97 (0.94-0.99)	0.97 (0.93-0.99)
Right superior parietal cortex	17.42 (2.52)	17.58 (2.42)	17.45 (2.43)	17.46 (2.46)	14.47	13.76	13.94	14.10	-1.00 (0.33)	-0.50 (0.62)	-0.35 (0.73)	0.97 (4.65)	0.22 (1.36)	0.25 (2.83)	1.97 (4.31)	1.04 (0.89)	1.86 (2.11)	0.95	1.00	0.98	0.95 (0.89-0.98)	1.00 (0.99-1.00)	0.98 (0.96-0.99)
Right superior temporal gyrus	14.49 (1.69)	14.48 (1.59)	14.34 (1.50)	14.44 (1.52)	11.63	11.01	10.45	10.53	0.42 (0.67)	2.40 (0.02)	0.93 (0.36)	-0.05 (1.50)	-0.97 (2.12)	-0.29 (2.00)	1.21 (0.86)	1.72 (1.54)	1.44 (1.39)	0.99	0.99	0.99	0.99 (0.98-1.00)	0.98 (0.94-0.99)	0.98 (0.96-0.99)
Right supramarginal gyrus	13.40 (2.42)	13.49 (2.36)	13.35 (2.35)	13.38 (2.43)	18.06	17.52	17.57	18.17	-0.63 (0.54)	0.92 (0.37)	0.35 (0.73)	0.73 (5.64)	-0.27 (1.81)	-0.13 (1.63)	2.39 (5.14)	1.45 (1.08)	1.28 (0.99)	0.95	1.00	1.00	0.96 (0.90-0.98)	0.99 (0.99-1.00)	1.00 (0.99-1.00)
Right frontal pole	1.54 (0.21)	1.58 (0.27)	1.29 (0.22)	1.52 (0.22)	13.86	16.85	17.09	14.41	-1.27 (0.22)	5.03 (0.00)	0.96 (0.34)	2.04 (9.91)	-18.09 (17.87)	-1.77 (9.16)	7.44 (6.70)	20.10 (15.48)	7.78 (4.91)	0.81	0.34	0.79	0.79 (0.59-0.90)	0.21 (-0.10-0.52)	0.80 (0.59-0.90)
Right temporal pole	2.72 (0.40)	2.69 (0.43)	2.71 (0.49)	2.64 (0.50)	14.87	15.90	18.22	18.93	0.83 (0.41)	0.13 (0.90)	1.40 (0.17)	-1.20 (6.18)	-0.83 (8.14)	-3.86 (11.97)	4.55 (4.25)	6.37 (4.97)	9.05 (8.58)	0.93	0.92	0.81	0.93 (0.84-0.97)	0.91 (0.80-0.96)	0.78 (0.57-0.90)
Right transverse temporal gyrus	1.35 (0.28)	1.35 (0.26)	1.34 (0.27)	1.34 (0.28)	20.52	19.54	19.81	20.71	0.09 (0.93)	0.67 (0.51)	1.18 (0.25)	0.07 (4.63)	-0.48 (4.88)	-0.76 (3.45)	3.79 (2.56)	3.90 (2.87)	2.49 (2.46)	0.98	0.98	0.99	0.98 (0.94-0.99)	0.98 (0.94-0.99)	0.99 (0.97-0.99)
Right insula	7.81 (0.77)	7.94 (0.90)	7.99 (0.96)	8.06 (0.79)	9.85	11.34	12.02	9.74	-1.67 (0.11)	-2.08 (0.05)	-2.93 (0.01)	1.58 (5.01)	2.09 (5.00)	3.18 (5.36)	3.45 (3.92)	3.42 (4.16)	4.36 (4.41)	0.89	0.90	0.85	0.87 (0.73-0.94)	0.86 (0.69-0.94)	0.81 (0.54-0.92)
Local cortical surface (cm²)																							
Left banks superior temporal sulcus	10.77 (1.29)	10.84 (1.47)	10.78 (1.32)	10.67 (1.33)	12.01	13.54	12.23	12.42	-0.90 (0.38)	-0.24 (0.82)	1.27 (0.22)	0.46 (3.71)	0.10 (3.10)	-0.94 (3.47)	2.93 (2.25)	2.60 (1.60)	3.15 (1.63)	0.97	0.97	0.96	0.96 (0.91-0.98)	0.97 (0.93-0.99)	0.96 (0.91-0.98)
Left caudal anterior cingulate cortex	6.64 (1.28)	6.66 (1.35)	6.69 (1.29)	6.63 (1.26)	19.21	20.32	19.22	19.05	-0.29 (0.78)	-0.78 (0.44)	0.18 (0.86)	-0.04 (3.74)	0.65 (4.33)	-0.13 (3.19)	2.97 (2.20)	3.34 (2.75)	2.30 (2.17)	0.99	0.97	0.99	0.98 (0.96-0.99)	0.97 (0.94-0.99)	0.99 (0.97-0.99)
Left caudal middle frontal gyrus	26.06 (4.29)	26.02 (4.34)	26.04 (4.39)	26.27 (4.28)	16.48	16.68	16.84	16.29	0.36 (0.72)	0.18 (0.86)	-1.61 (0.12)	-0.20 (2.27)	-0.12 (2.16)	0.79 (2.41)	1.81 (1.33)	1.64 (1.37)	1.80 (1.76)	0.99	0.99	0.99	0.99 (0.98-1.00)	0.99 (0.98-1.00)	0.99 (0.97-0.99)
Left cuneus	17.01 (2.22)	16.98 (2.17)	17.06 (2.14)	16.98 (2.14)	13.05	12.76	12.56	12.60	0.47 (0.64)	-0.82 (0.42)	0.48 (0.63)	-0.16 (1.96)	0.34 (1.75)	-0.13 (1.92)	1.54 (1.19)	1.41 (1.06)	1.43 (1.25)	0.99	0.99	0.99	0.99 (0.97-0.99)	0.99 (0.98-1.00)	0.99 (0.97-0.99)
Left entorhinal cortex	4.66 (0.92)	4.64 (0.93)	4.66 (0.97)	4.63 (1.04)	19.86	20.03	20.87	22.39	0.20 (0.84)	-0.06 (0.96)	0.32 (0.75)	-0.42 (7.48)	-0.07 (5.95)	-1.29 (9.72)	5.61 (4.83)	4.70 (3.53)	7.50 (6.14)	0.93	0.96	0.90	0.93 (0.85-0.97)	0.96 (0.90-0.98)	0.90 (0.79-0.95)
Left fusiform gyrus	34.73 (2.99)	34.52 (3.04)	34.58 (3.18)	34.49 (3.11)	8.60	8.80	9.20	9.02	2.22 (0.04)	1.17 (0.25)	1.61 (0.12)	-0.62 (1.38)	-0.46 (1.75)	-0.72 (2.10)	1.07 (1.05)	1.43 (1.07)	1.66 (1.44)	0.99	0.98	0.97	0.99 (0.96-0.99)	0.98 (0.95-0.99)	0.97 (0.93-0.99)
Left inferior parietal cortex	50.56 (5.95)	50.68 (5.89)	50.35 (5.74)	50.62 (5.62)	11.76	11.63	11.39	11.11	-0.80 (0.43)	1.62 (0.12)	-0.29 (0.77)	0.25 (1.42)	-0.39 (1.31)	0.19 (1.93)	1.13 (0.87)	1.05 (0.85)	1.39 (1.33)	0.99	0.99	0.99	0.99 (0.98-1.00)	0.99 (0.98-1.00)	0.99 (0.97-0.99)
Left inferior temporal gyrus	37.43 (4.86)	37.29 (4.91)	37.43 (4.95)	37.52 (4.90)	12.99	13.17	13.23	13.06	1.11 (0.28)	-0.01 (0.99)	-0.77 (0.45)	-0.39 (1.67)	-0.03 (1.30)	0.23 (1.60)	1.23 (1.17)	1.12 (0.63)	1.10 (1.16)	0.99	1.00	0.99	0.99 (0.98-1.00)	1.00 (0.99-1.00)	0.99 (0.99-1.00)
Left isthmus cingulate cortex	11.16 (2.03)	11.24 (2.06)	11.27 (2.01)	11.23 (2.05)	18.17	18.35	17.82	18.30	-1.03 (0.32)	-1.67 (0.11)	-0.96 (0.35)	0.59 (3.19)	0.97 (2.67)	0.49 (3.00)	2.48 (2.03)	2.41 (1.44)	2.12 (2.13)	0.98	0.99	0.99	0.98 (0.97-0.99)	0.99 (0.97-0.99)	0.99 (0.97-0.99)
Left lateral occipital cortex	56.75 (7.50)	56.75 (7.70)	56.81 (7.58)	57.01 (7.79)	13.23	13.58	13.35	13.66	-0.02 (0.98)	-0.35 (0.73)	-1.43 (0.17)	-0.03 (1.05)	0.10 (1.42)	0.42 (1.52)	0.86 (0.57)	1.09 (0.89)	1.16 (1.04)	1.00	0.99	0.99	1.00 (0.99-1.00)	0.99 (0.98-1.00)	0.99 (0.98-1.00)
Left lateral orbitofrontal cortex	27.74 (3.01)	27.67 (3.13)	27.56 (3.11)	27.82 (3.18)	10.86	11.30	11.28	11.43	0.79 (0.44)	1.45 (0.16)	-0.73 (0.47)	-0.33 (1.79)	-0.72 (2.30)	0.20 (1.82)	1.52 (0.96)	2.04 (1.22)	1.48 (1.04)	0.99	0.98	0.99	0.99 (0.97-0.99)	0.98 (0.95-0.99)	0.99 (0.97-0.99)
Left lingual gyrus	33.36 (4.25)	33.49 (4.45)	33.56 (4.47)	33.44 (4.42)	12.74	13.30	13.33	13.22	-1.16 (0.26)	-1.62 (0.12)	-0.62 (0.54)	0.33 (1.67)	0.55 (1.80)	0.21 (1.98)	1.19 (1.19)	1.25 (1.39)	1.45 (1.34)	0.99	0.99	0.99	0.99 (0.98-1.00)	0.99 (0.97-0.99)	0.99 (0.97-0.99)
Left medial orbitofrontal cortex	19.05 (2.07)	19.79 (2.53)	19.30 (2.59)	19.49 (2.72)	10.89	12.77	13.40	13.96	-2.95 (0.01)	-0.98 (0.33)	-1.39 (0.18)	3.65 (6.47)	1.03 (6.60)	1.96 (8.03)	6.10 (4.11)	5.04 (4.28)	5.99 (5.58)	0.87	0.87	0.81	0.81 (0.54-0.92)	0.85 (0.69-0.93)	0.78 (0.56-0.89)
Left middle temporal gyrus	35.06 (3.40)	35.26 (3.35)	34.99 (3.30)	35.25 (3.43)	9.69	9.51	9.44	9.73	-2.16 (0.04)	0.73 (0.47)	-1.96 (0.06)	0.60 (1.37)	-0.18 (1.31)	0.52 (1.36)	1.25 (0.79)	1.00 (0.85)	1.13 (0.90)	0.99	0.99	0.99	0.99 (0.97-1.00)	0.99 (0.98-1.00)	0.99 (0.97-1.00)
Left parahippocampal gyrus	6.77 (0.62)	6.82 (0.76)	6.78 (0.73)	6.81 (0.73)	9.09	11.09	10.75	10.68	-0.77 (0.45)	-0.14 (0.89)	-0.81 (0.43)	0.58 (4.59)	-0.01 (4.39)	0.52 (3.80)	3.43 (3.04)	3.15 (2.99)	2.83 (2.52)	0.90	0.90	0.93	0.88 (0.76-0.95)	0.89 (0.77-0.95)	0.92 (0.82-0.96)
Left paracentral lobule	14.19 (1.55)	14.22 (1.62)	14.24 (1.60)	14.23 (1.61)	10.95	11.38	11.21	11.30	-0.45 (0.66)	-1.04 (0.31)	-0.77 (0.45)	0.14 (2.09)	0.36 (1.81)	0.26 (1.94)	1.60 (1.31)	1.47 (1.08)	1.58 (1.12)	0.98	0.99	0.99	0.98 (0.96-0.99)	0.99 (0.97-0.99)	0.99 (0.97-0.99)
Left pars opercularis	18.23 (2.09)	18.25 (2.09)	18.14 (2.13)	18.21 (2.03)	11.48	11.43	11.73	11.17	-0.34 (0.74)	1.42 (0.17)	0.23 (0.82)	0.14 (1.98)	-0.50 (1.63)	-0.09 (2.46)	1.65 (1.06)	1.35 (1.02)	1.65 (1.80)	0.98	0.99	0.97	0.99 (0.97-0.99)	0.99 (0.97-1.00)	0.97 (0.94-0.99)
Left pars orbitalis	7.73 (0.88)	7.68 (1.05)	7.74 (0.98)	7.70 (0.99)	11.34	13.65	12.64	12.80	0.75 (0.46)	-0.11 (0.91)	0.60 (0.56)	-0.90 (4.08)	-0.03 (4.22)	-0.56 (3.35)	3.32 (2.45)	3.28 (2.57)	2.56 (2.18)	0.96	0.94	0.97	0.95 (0.88-0.98)	0.93 (0.85-0.97)	0.96 (0.92-0.98)
Left pars triangularis	14.37 (1.76)	14.46 (1.82)	14.45 (1.76)	14.45 (1.76)	12.28	12.61	12.20	12.20	-0.98 (0.34)	-0.93 (0.36)	-1.00 (0.33)	0.63 (3.23)	0.57 (2.97)	0.62 (3.02)	2.62 (1.92)	2.51 (1.61)	2.49 (1.75)	0.97	0.97	0.97	0.97 (0.92-0.98)	0.97 (0.94-0.99)	0.97 (0.93-0.99)
Left pericalcarine cortex	15.91 (2.52)	15.92 (2.54)	16.03 (2.67)	15.99 (2.60)	15.84	15.96	16.67	16.26	-0.14 (0.89)	-1.43 (0.17)	-0.87 (0.39)	0.05 (2.11)	0.64 (2.58)	0.48 (2.90)	1.80 (1.06)	2.15 (1.50)	2.17 (1.94)	0.99	0.99	0.98	0.99 (0.98-1.00)	0.99 (0.97-0.99)	0.98 (0.96-0.99)
Left postcentral gyrus	43.69 (3.88)	43.87 (3.73)	43.79 (3.91)	43.85 (3.85)	8.87	8.51	8.93	8.79	-1.53 (0.14)	-1.01 (0.32)	-1.23 (0.23)	0.46 (1.46)	0.24 (1.24)	0.37 (1.55)	0.88 (1.25)	0.97 (0.79)	0.96 (1.26)	0.99	0.99	0.99	0.99 (0.97-0.99)	0.99 (0.98-1.00)	0.99 (0.97-0.99)
Left posterior cingulate cortex	12.62 (2.18)	12.69 (2.14)	12.64 (2.21)	12.71 (2.16)	17.27	16.86	17.50	17.00	-1.06 (0.30)	-0.21 (0.84)	-1.14 (0.27)	0.59 (2.41)	0.12 (2.99)	0.80 (2.92)	2.06 (1.33)	2.41 (1.70)	2.50 (1.63)	0.99	0.98	0.98	0.99 (0.98-1.00)	0.98 (0.96-0.99)	0.98 (0.96-0.99)
Left precentral gyrus	49.99 (4.71)	49.99 (4.85)	50.01 (4.69)	49.99 (4.69)	9.41	9.69	9.39	9.37	-0.07 (0.94)	-0.18 (0.86)	-0.04 (0.97)	-0.01 (1.02)	0.04 (1.09)	0.02 (1.36)	0.81 (0.61)	0.76 (0.76)	0.95 (0.94)	0.99	0.99	0.99	0.99 (0.99-1.00)	0.99 (0.98-1.00)	0.99 (0.98-1.00)
Left precuneus	42.86 (4.81)	42.91 (4.91)	42.76 (4.95)	43.01 (4.80)	11.23	11.45	11.58	11.16	-0.57 (0.57)	1.08 (0.29)	-1.43 (0.16)	0.10 (1.03)	-0.28 (1.11)	0.33 (1.12)	0.77 (0.67)	0.91 (0.68)	0.93 (0.69)	1.00	1.00	0.99	1.00 (0.99-1.00)	1.00 (0.99-1.00)	0.99 (0.99-1.00)

Left rostral anterior cingulate cortex	8.74 (1.50)	8.85 (1.68)	8.91 (1.66)	8.74 (1.49)	17.16	18.96	18.61	17.08	-1.25 (0.22)	-2.08 (0.05)	0.04 (0.97)	0.97 (4.99)	1.74 (4.89)	-0.03 (4.09)	3.68 (3.44)	4.03 (3.19)	3.24 (2.39)	0.97	0.97	0.97	0.96 (0.92-0.98)	0.96 (0.91-0.98)	0.97 (0.93-0.99)
Left rostral middle frontal gyrus	65.63 (7.64)	65.90 (7.78)	65.70 (7.62)	66.02 (7.68)	11.65	11.80	11.61	11.63	-1.48 (0.15)	-0.30 (0.77)	-1.76 (0.09)	0.40 (1.40)	0.11 (1.78)	0.59 (1.72)	1.17 (0.84)	1.37 (1.11)	1.41 (1.12)	0.99	0.99	0.99	0.99 (0.98-1.00)	0.99 (0.97-0.99)	0.99 (0.97-1.00)
Left superior frontal gyrus	78.98 (9.75)	78.98 (9.59)	78.73 (9.86)	79.17 (9.92)	12.35	12.15	12.53	12.53	-0.04 (0.97)	0.99 (0.33)	-0.94 (0.36)	0.03 (1.19)	-0.33 (1.56)	0.22 (1.27)	0.91 (0.75)	1.24 (0.97)	0.99 (0.80)	1.00	0.99	0.99	1.00 (0.99-1.00)	0.99 (0.98-1.00)	0.99 (0.99-1.00)
Left superior parietal cortex	61.63 (7.94)	61.52 (8.11)	61.69 (8.02)	61.42 (7.80)	12.88	13.17	12.99	12.70	0.80 (0.43)	-0.54 (0.59)	1.48 (0.15)	-0.22 (1.19)	0.07 (0.86)	-0.34 (1.14)	0.78 (0.91)	0.65 (0.54)	0.98 (0.64)	1.00	1.00	1.00	1.00 (0.99-1.00)	1.00 (1.00-1.00)	1.00 (0.99-1.00)
Left superior temporal gyrus	42.42 (5.41)	42.55 (5.44)	42.38 (5.46)	42.50 (5.32)	12.74	12.80	12.89	12.52	-1.21 (0.24)	0.44 (0.66)	-0.63 (0.53)	0.28 (1.21)	-0.11 (0.96)	0.20 (1.44)	0.94 (0.78)	0.76 (0.57)	1.08 (0.95)	1.00	1.00	0.99	1.00 (0.99-1.00)	1.00 (0.99-1.00)	0.99 (0.98-1.00)
Left supramarginal gyrus	46.83 (8.81)	46.96 (9.13)	47.01 (9.09)	47.04 (9.17)	18.82	19.45	19.33	19.49	-0.75 (0.46)	-1.40 (0.17)	-1.53 (0.14)	0.20 (1.59)	0.32 (1.25)	0.33 (1.42)	1.25 (0.97)	1.11 (0.61)	1.14 (0.88)	1.00	1.00	1.00	1.00 (0.99-1.00)	1.00 (0.99-1.00)	1.00 (0.99-1.00)
Left frontal pole	2.88 (0.32)	2.83 (0.31)	2.71 (0.38)	2.87 (0.33)	11.18	10.89	13.88	11.51	1.51 (0.14)	4.22 (0.00)	0.24 (0.81)	-1.84 (6.55)	-6.26 (7.67)	-0.34 (6.06)	5.89 (3.21)	7.70 (6.16)	4.14 (4.36)	0.84	0.85	0.86	0.83 (0.66-0.92)	0.76 (0.28-0.91)	0.87 (0.72-0.94)
Left temporal pole	4.90 (0.64)	4.97 (0.56)	4.94 (0.65)	4.96 (0.62)	13.16	11.24	13.24	12.40	-1.18 (0.25)	-1.05 (0.30)	-1.24 (0.23)	1.80 (6.97)	0.89 (4.53)	1.44 (5.69)	5.29 (4.77)	3.20 (3.27)	4.51 (3.66)	0.85	0.94	0.91	0.84 (0.68-0.93)	0.94 (0.88-0.97)	0.91 (0.80-0.96)
Left transverse temporal gyrus	4.58 (0.80)	4.60 (0.84)	4.63 (0.78)	4.65 (0.87)	17.51	18.21	16.95	18.68	-0.40 (0.69)	-1.29 (0.21)	-2.01 (0.06)	0.19 (4.30)	1.05 (3.95)	1.34 (3.93)	3.30 (2.68)	3.22 (2.44)	3.17 (2.60)	0.97	0.98	0.98	0.97 (0.94-0.99)	0.98 (0.94-0.99)	0.98 (0.94-0.99)
Left insula	23.89 (2.30)	23.47 (2.49)	23.73 (2.28)	23.88 (2.68)	9.64	10.59	9.62	11.23	1.56 (0.13)	0.56 (0.58)	0.04 (0.97)	-1.87 (5.80)	-0.71 (6.31)	-0.20 (5.99)	4.45 (4.09)	4.47 (4.42)	4.26 (4.12)	0.84	0.79	0.82	0.83 (0.65-0.92)	0.80 (0.59-0.90)	0.82 (0.63-0.92)
Right banks superior temporal sulcus	9.66 (1.10)	9.62 (1.26)	9.65 (1.20)	9.56 (1.17)	11.34	13.10	12.39	12.22	0.39 (0.70)	0.07 (0.95)	1.27 (0.21)	-0.57 (4.53)	-0.17 (3.19)	-1.11 (3.97)	3.60 (2.71)	2.26 (2.20)	3.13 (2.62)	0.93	0.97	0.95	0.93 (0.84-0.97)	0.97 (0.93-0.99)	0.94 (0.87-0.97)
Right caudal anterior cingulate cortex	7.28 (1.57)	7.28 (1.48)	7.34 (1.55)	7.27 (1.51)	21.60	20.35	21.12	20.75	-0.03 (0.97)	-1.45 (0.16)	0.26 (0.80)	0.21 (4.65)	0.95 (3.29)	-0.03 (2.85)	3.29 (3.21)	2.89 (1.74)	2.34 (1.54)	0.97	0.99	0.99	0.97 (0.94-0.99)	0.99 (0.98-1.00)	0.99 (0.98-1.00)
Right caudal middle frontal gyrus	24.93 (2.93)	24.66 (2.82)	24.88 (3.03)	25.05 (3.01)	11.74	11.45	12.17	12.01	0.74 (0.47)	0.36 (0.72)	-1.17 (0.25)	-1.03 (7.11)	-0.21 (2.35)	0.47 (2.04)	2.83 (6.59)	1.66 (1.65)	1.65 (1.25)	0.80	0.98	0.99	0.80 (0.61-0.91)	0.98 (0.95-0.99)	0.98 (0.97-0.99)
Right cuneus	18.12 (3.12)	18.16 (3.14)	18.24 (3.14)	18.15 (3.23)	17.23	17.27	17.19	17.80	-0.46 (0.65)	-1.65 (0.11)	-0.41 (0.69)	0.24 (2.49)	0.72 (2.10)	0.13 (2.12)	1.86 (1.63)	1.74 (1.33)	1.66 (1.28)	0.99	0.99	0.99	0.99 (0.97-0.99)	0.99 (0.98-1.00)	0.99 (0.98-1.00)
Right entorhinal cortex	4.29 (0.77)	4.37 (0.81)	4.42 (0.78)	4.36 (0.82)	18.02	18.51	17.55	18.74	-1.77 (0.09)	-1.90 (0.07)	-1.18 (0.25)	1.75 (5.44)	3.05 (8.45)	1.50 (7.23)	4.24 (3.75)	7.19 (5.22)	5.66 (4.61)	0.96	0.90	0.93	0.95 (0.90-0.98)	0.89 (0.76-0.95)	0.93 (0.84-0.97)
Right fusiform gyrus	33.09 (4.17)	33.02 (4.27)	32.93 (4.15)	33.08 (4.20)	12.60	12.92	12.59	12.70	0.80 (0.43)	1.50 (0.15)	0.11 (0.91)	-0.25 (1.39)	-0.49 (1.74)	-0.04 (1.54)	1.14 (0.80)	1.27 (1.26)	1.25 (0.86)	0.99	0.99	0.99	0.99 (0.99-1.00)	0.99 (0.98-1.00)	0.99 (0.98-1.00)
Right inferior parietal cortex	60.29 (8.28)	60.58 (8.33)	60.43 (8.36)	60.62 (8.06)	13.73	13.76	13.83	13.30	-1.04 (0.31)	-0.88 (0.39)	-1.58 (0.13)	0.47 (2.14)	0.25 (1.43)	0.60 (1.72)	1.30 (1.74)	1.00 (1.03)	1.29 (1.26)	0.99	1.00	0.99	0.99 (0.97-0.99)	1.00 (0.99-1.00)	0.99 (0.98-1.00)
Right inferior temporal gyrus	35.51 (4.84)	35.71 (4.82)	35.52 (4.82)	35.80 (4.92)	13.64	13.50	13.58	13.74	-1.81 (0.08)	-0.14 (0.89)	-2.59 (0.02)	0.60 (1.63)	0.04 (1.51)	0.80 (1.63)	1.29 (1.14)	1.13 (0.97)	1.53 (0.94)	0.99	0.99	0.99	0.99 (0.98-1.00)	0.99 (0.99-1.00)	0.99 (0.98-1.00)
Right isthmus cingulate cortex	10.30 (1.91)	10.35 (1.90)	10.27 (1.86)	10.27 (1.87)	18.59	18.37	18.14	18.20	-0.94 (0.36)	0.45 (0.66)	0.56 (0.58)	0.51 (2.93)	-0.23 (2.93)	-0.31 (2.88)	2.40 (1.69)	2.25 (1.83)	2.37 (1.61)	0.99	0.99	0.99	0.99 (0.98-1.00)	0.99 (0.97-0.99)	0.99 (0.97-0.99)
Right lateral occipital cortex	56.82 (6.91)	57.05 (7.34)	56.88 (7.36)	56.99 (7.47)	12.16	12.87	12.94	13.11	-1.16 (0.26)	-0.37 (0.71)	-0.92 (0.37)	0.33 (1.65)	0.04 (1.39)	0.20 (1.52)	1.17 (1.19)	1.09 (0.83)	1.16 (0.98)	0.99	0.99	0.99	0.99 (0.98-1.00)	0.99 (0.98-1.00)	0.99 (0.98-1.00)
Right lateral orbitofrontal cortex	28.61 (4.00)	28.27 (3.59)	27.95 (3.35)	27.81 (3.41)	13.98	12.71	11.99	12.26	0.94 (0.36)	1.85 (0.08)	2.63 (0.01)	-1.06 (6.15)	-2.11 (6.17)	-2.66 (5.17)	4.97 (3.65)	4.66 (4.48)	4.44 (3.69)	0.89	0.90	0.93	0.88 (0.76-0.95)	0.87 (0.73-0.94)	0.90 (0.74-0.96)
Right lingual gyrus	34.59 (4.92)	34.68 (4.98)	34.54 (4.79)	34.53 (4.85)	14.21	14.35	13.86	14.04	-0.85 (0.41)	0.37 (0.72)	0.49 (0.63)	0.26 (1.63)	-0.10 (1.80)	-0.14 (1.62)	1.21 (1.10)	1.38 (1.12)	1.37 (0.81)	0.99	0.99	0.99	0.99 (0.99-1.00)	0.99 (0.98-1.00)	0.99 (0.99-1.00)
Right medial orbitofrontal cortex	20.22 (2.26)	20.34 (2.47)	20.14 (2.25)	20.36 (2.36)	11.17	12.15	11.17	11.58	-0.64 (0.53)	0.56 (0.58)	-0.78 (0.44)	0.49 (4.67)	-0.38 (3.40)	0.68 (4.55)	3.33 (3.24)	2.62 (2.13)	3.53 (2.86)	0.92	0.95	0.92	0.92 (0.82-0.96)	0.96 (0.90-0.98)	0.92 (0.83-0.96)
Right middle temporal gyrus	39.06 (3.82)	39.27 (3.79)	39.17 (3.78)	39.04 (3.90)	9.78	9.64	9.66	9.98	-1.99 (0.06)	-1.05 (0.31)	0.16 (0.88)	0.55 (1.34)	0.29 (1.29)	-0.06 (1.44)	1.15 (0.85)	1.15 (0.61)	1.17 (0.81)	0.99	0.99	0.99	0.99 (0.97-1.00)	0.99 (0.98-1.00)	0.99 (0.98-1.00)
Right parahippocampal gyrus	6.31 (0.57)	6.31 (0.55)	6.38 (0.53)	6.32 (0.58)	9.10	8.79	8.24	9.14	0.13 (0.90)	-1.46 (0.16)	-0.16 (0.88)	-0.07 (3.71)	1.15 (3.75)	0.12 (4.29)	3.09 (1.95)	3.12 (2.29)	3.45 (2.45)	0.92	0.91	0.89	0.92 (0.83-0.96)	0.90 (0.80-0.96)	0.90 (0.78-0.95)
Right paracentral lobule	15.56 (1.65)	15.57 (1.78)	15.52 (1.86)	15.42 (1.87)	10.63	11.42	12.01	12.12	-0.05 (0.96)	0.39 (0.70)	1.03 (0.31)	-0.05 (3.99)	-0.44 (4.00)	-1.10 (4.87)	2.19 (3.31)	2.26 (3.29)	2.74 (4.15)	0.94	0.95	0.93	0.94 (0.87-0.97)	0.94 (0.88-0.98)	0.92 (0.83-0.96)
Right pars opercularis	15.69 (1.49)	15.79 (1.59)	15.77 (1.60)	15.79 (1.58)	9.48	10.08	10.16	10.00	-1.24 (0.23)	-0.98 (0.34)	-0.86 (0.40)	0.59 (2.70)	0.40 (2.27)	0.57 (3.54)	1.89 (1.98)	1.77 (1.44)	2.59 (2.43)	0.97	0.97	0.93	0.96 (0.92-0.98)	0.97 (0.94-0.99)	0.93 (0.86-0.97)
Right pars orbitalis	9.15 (1.08)	9.22 (1.17)	9.23 (1.14)	9.17 (1.21)	11.81	12.71	12.33	13.17	-0.99 (0.33)	-1.36 (0.19)	-0.25 (0.81)	0.63 (3.44)	0.80 (3.10)	0.03 (3.34)	2.68 (2.17)	2.61 (1.78)	2.69 (1.91)	0.96	0.97	0.97	0.96 (0.91-0.98)	0.96 (0.92-0.98)	0.96 (0.92-0.98)
Right pars triangularis	16.77 (2.29)	16.74 (2.21)	16.96 (2.28)	16.63 (2.16)	13.68	13.21	13.45	13.02	0.35 (0.73)	-2.10 (0.05)	1.61 (0.12)	-0.13 (3.08)	1.10 (2.53)	-0.77 (2.53)	2.24 (2.07)	2.17 (1.66)	2.09 (1.57)	0.98	0.98	0.98	0.98 (0.95-0.99)	0.98 (0.95-0.99)	0.98 (0.95-0.99)
Right pericalcarine cortex	16.90 (2.62)	16.85 (2.63)	16.91 (2.60)	16.88 (2.64)	15.48	15.63	15.39	15.62	0.53 (0.60)	-0.21 (0.83)	0.21 (0.83)	-0.26 (2.28)	0.11 (2.68)	-0.13 (2.76)	1.90 (1.23)	2.07 (1.66)	2.25 (1.53)	0.99	0.99	0.98	0.99 (0.98-1.00)	0.99 (0.97-0.99)	0.98 (0.97-0.99)
Right postcentral gyrus	41.25 (4.56)	41.92 (4.00)	41.33 (4.32)	41.18 (5.17)	11.05	9.55	10.45	12.54	-1.38 (0.18)	-0.59 (0.56)	0.31 (0.76)	1.77 (6.68)	0.26 (1.69)	-0.40 (3.64)	2.16 (6.56)	1.25 (1.14)	1.61 (3.27)	0.85	0.99	0.98	0.84 (0.67-0.92)	0.99 (0.98-1.00)	0.98 (0.95-0.99)
Right posterior cingulate cortex	13.04 (2.22)	12.97 (2.25)	13.03 (2.19)	13.06 (2.25)	17.01	17.37	16.82	17.20	0.82 (0.42)	0.05 (0.96)	-0.33 (0.75)	-0.57 (3.16)	-0.01 (1.85)	0.14 (2.36)	2.22 (2.27)	1.46 (1.10)	1.70 (1.60)	0.98	0.99	0.99	0.98 (0.96-0.99)	0.99 (0.99-1.00)	0.99 (0.98-1.00)
Right precentral gyrus	51.25 (6.16)	50.14 (4.14)	50.65 (4.25)	50.41 (4.00)	12.02	8.25	8.39	7.93	1.49 (0.15)	1.16 (0.26)	1.30 (0.21)	-1.88 (6.45)	-0.89 (4.12)	-1.33 (5.22)	2.58 (6.20)	1.89 (3.76)	2.14 (4.94)	0.80	0.94	0.88	0.73 (0.49-0.87)	0.88 (0.74-0.94)	0.80 (0.60-0.91)
Right precuneus	45.42 (4.95)	45.32 (4.87)	45.32 (5.06)	45.32 (4.67)	10.89	10.75	11.16	10.30	0.39 (0.70)	0.65 (0.52)	0.49 (0.63)	-0.20 (2.81)	-0.26 (1.78)	-0.17 (2.35)	1.56 (2.33)	1.17 (1.35)	1.55 (1.74)	0.97	0.99	0.98	0.97 (0.93-0.99)	0.99 (0.97-0.99)	0.98 (0.95-0.99)
Right rostral anterior cingulate cortex	6.56 (1.36)	6.46 (1.32)	6.53 (1.42)	6.66 (1.39)	20.75	20.40	21.77	20.87	1.85 (0.08)	0.47 (0.64)	-1.81 (0.08)	-1.43 (3.72)	-0.74 (5.11)	1.47 (4.36)	3.18 (2.34)	3.62 (3.61)	3.66 (2.70)	0.98	0.97	0.98	0.98 (0.95-0.99)	0.97 (0.94-0.99)	0.98 (0.95-0.99)
Right rostral middle frontal gyrus	66.90 (7.69)	66.69 (7.66)	66.61 (7.44)	67.46 (7.60)	11.49	11.49	11.18	11.26	0.65 (0.52)	0.97 (0.34)	-2.06 (0.05)	-0.32 (2.52)	-0.42 (2.34)	0.86 (2.15)	1.58 (1.96)	1.54 (1.79)	1.61 (1.64)	0.98	0.98	0.98	0.98 (0.95-0.99)	0.98 (0.95-0.99)	0.98 (0.96-0.99)
Right superior frontal gyrus	77.65 (9.89)	77.38 (9.77)	77.65 (9.82)	78.03 (9.72)	12.74	12.63	12.65	12.45	0.30 (0.77)	-0.01 (0.99)	-1.01 (0.32)	-0.32 (5.65)	0.01 (2.02)	0.52 (2.29)	2.24 (5.18)	1.22 (1.59)	1.34 (1.91)	0.89	0.99	0.98	0.90 (0.78-0.95)	0.99 (0.97-0.99)	0.98 (0.96-0.99)
Right superior parietal cortex	60.21 (7.65)	60.78 (7.21)	60.37 (7.33)	60.66 (7.47)	12.71	11.86	12.14	12.32	-0.99 (0.33)	-1.40 (0.18)	-1.50 (0.15)	1.02 (4.95)	0.32 (0.93)	0.78 (2.69)	1.74 (4.73)	0.76 (0.62)	1.18 (2.54)	0.93	1.00	0.98	0.93 (0.84-0.97)	1.00 (0.99-1.00)	0.98 (0.96-0.99)
Right superior temporal gyrus	40.13 (4.52)	40.11 (4.54)	40.08 (4.57)	40.15 (4.31)	11.26	11.32	11.40	10.73	0.20 (0.84)	0.50 (0.62)	-0.26 (0.79)	-0.06 (1.35)	-0.14 (1.08)	0.11 (1.08)	1.03 (0.85)	0.91 (0.57)	0.78 (0.73)	0.99	0.99	1.00	0.99 (0.98-1.00)	0.99 (0.99-1.00)	1.00 (0.99-1.00)
Right supramarginal gyrus	40.22 (7.03)	40.53 (6.89)	40.08 (7.05)	40.10 (7.09)	17.49	17.01	17.60	17.67	-0.79 (0.44)	1.39 (0.18)	1.58 (0.13)	0.81 (5.07)	-0.39 (1.43)	-0.33 (0.93)	1.85 (4.78)	1.08 (0.99)	0.72 (0.66)	0.96	1.00	1.00	0.96 (0.92-0.98)	1.00 (0.99-1.00)	1.00 (1.00-1.00)

Right frontal pole	3.42 (0.34)	3.52 (0.55)	3.27 (0.43)	3.39 (0.38)	9.83	15.72	13.11	11.16	-1.26 (0.22)	2.62 (0.02)	0.55 (0.59)	2.21 (10.79)	-4.99 (8.94)	-0.98 (7.76)	8.72 (6.50)	7.74 (6.59)	6.31 (4.45)	0.69	0.73	0.74	0.61 (0.30-0.80)	0.67 (0.34-0.84)	0.74 (0.49-0.88)
Right temporal pole	4.74 (0.61)	4.65 (0.69)	4.73 (0.67)	4.74 (0.64)	12.84	14.89	14.09	13.55	1.58 (0.13)	0.17 (0.87)	-0.03 (0.98)	-2.12 (5.93)	-0.40 (5.36)	-0.10 (7.01)	4.88 (3.88)	4.31 (3.09)	5.42 (4.30)	0.92	0.94	0.85	0.91 (0.80-0.96)	0.94 (0.86-0.97)	0.85 (0.69-0.93)
Right transverse temporal gyrus	3.85 (0.68)	3.83 (0.62)	3.81 (0.67)	3.83 (0.71)	17.72	16.28	17.67	18.43	0.43 (0.67)	1.15 (0.26)	0.88 (0.39)	-0.26 (4.58)	-1.04 (4.71)	-0.75 (3.95)	3.90 (2.28)	3.56 (3.18)	2.69 (2.95)	0.97	0.97	0.98	0.96 (0.92-0.98)	0.97 (0.93-0.99)	0.98 (0.96-0.99)
Right insula	22.99 (2.14)	23.49 (2.59)	23.72 (3.18)	24.00 (2.42)	9.32	11.01	13.42	10.09	-1.67 (0.11)	-1.95 (0.06)	-3.51 (0.00)	1.95 (6.10)	2.70 (7.42)	4.22 (6.03)	3.86 (5.06)	5.31 (5.77)	5.74 (4.55)	0.82	0.82	0.81	0.79 (0.59-0.90)	0.74 (0.49-0.88)	0.73 (0.34-0.89)
Local cortical thickness (mm)																							
Left banks superior temporal sulcus	2.86 (0.15)	2.86 (0.15)	2.87 (0.15)	2.86 (0.15)	5.36	5.09	5.13	5.11	0.18 (0.86)	-0.85 (0.40)	0.28 (0.78)	-0.05 (1.69)	0.24 (1.31)	-0.10 (1.95)	1.21 (1.15)	1.01 (0.85)	1.48 (1.24)	0.95	0.97	0.93	0.95 (0.89-0.98)	0.97 (0.93-0.99)	0.93 (0.85-0.97)
Left caudal anterior cingulate cortex	2.94 (0.24)	2.94 (0.25)	2.96 (0.24)	2.94 (0.23)	8.17	8.37	8.15	7.74	-0.10 (0.92)	-1.14 (0.26)	-0.09 (0.93)	0.03 (2.34)	0.75 (3.31)	0.08 (2.60)	1.73 (1.54)	2.65 (2.07)	2.16 (1.38)	0.96	0.92	0.95	0.96 (0.92-0.98)	0.92 (0.83-0.96)	0.95 (0.89-0.98)
Left caudal middle frontal gyrus	2.75 (0.13)	2.73 (0.14)	2.75 (0.14)	2.74 (0.15)	4.72	5.14	4.92	5.40	1.21 (0.24)	0.13 (0.89)	0.99 (0.33)	-0.48 (1.95)	-0.05 (1.60)	-0.35 (1.65)	1.60 (1.17)	1.25 (0.97)	1.29 (1.06)	0.93	0.95	0.96	0.93 (0.84-0.97)	0.95 (0.89-0.98)	0.95 (0.89-0.98)
Left cuneus	2.27 (0.13)	2.26 (0.13)	2.26 (0.14)	2.27 (0.13)	5.81	5.65	6.16	5.74	1.75 (0.09)	1.35 (0.19)	-0.13 (0.90)	-0.34 (0.99)	-0.38 (1.37)	0.04 (1.43)	0.86 (0.58)	1.10 (0.87)	1.06 (0.93)	0.99	0.98	0.97	0.98 (0.96-0.99)	0.97 (0.94-0.99)	0.97 (0.94-0.99)
Left entorhinal cortex	3.37 (0.32)	3.37 (0.32)	3.38 (0.35)	3.39 (0.32)	9.37	9.46	10.32	9.38	-0.17 (0.87)	-0.56 (0.58)	-0.85 (0.40)	0.11 (3.39)	0.27 (3.39)	0.54 (3.42)	2.75 (1.90)	2.79 (1.86)	2.34 (2.50)	0.94	0.95	0.94	0.94 (0.87-0.97)	0.95 (0.88-0.98)	0.94 (0.88-0.97)
Left fusiform gyrus	2.98 (0.13)	2.98 (0.13)	2.98 (0.13)	2.99 (0.13)	4.49	4.49	4.36	4.32	0.37 (0.72)	0.18 (0.86)	-0.90 (0.38)	-0.08 (1.04)	-0.03 (0.91)	0.13 (0.68)	0.79 (0.66)	0.72 (0.53)	0.55 (0.41)	0.97	0.98	0.99	0.97 (0.94-0.99)	0.98 (0.95-0.99)	0.99 (0.97-0.99)
Left inferior parietal cortex	2.81 (0.09)	2.81 (0.09)	2.82 (0.09)	2.82 (0.09)	3.10	3.16	3.02	3.14	0.81 (0.43)	-0.55 (0.59)	-0.53 (0.60)	-0.26 (1.59)	0.14 (1.29)	0.13 (1.24)	1.30 (0.92)	1.05 (0.73)	1.02 (0.68)	0.87	0.91	0.92	0.87 (0.74-0.94)	0.91 (0.82-0.96)	0.92 (0.83-0.97)
Left inferior temporal gyrus	2.93 (0.13)	2.92 (0.13)	2.92 (0.13)	2.91 (0.12)	4.52	4.52	4.59	4.25	0.84 (0.41)	1.14 (0.26)	1.98 (0.06)	-0.19 (1.14)	-0.24 (1.07)	-0.57 (1.45)	0.86 (0.75)	0.85 (0.68)	1.27 (0.89)	0.97	0.97	0.95	0.97 (0.94-0.99)	0.97 (0.94-0.99)	0.94 (0.86-0.97)
Left isthmus cingulate cortex	2.63 (0.19)	2.63 (0.16)	2.63 (0.18)	2.61 (0.17)	7.05	6.17	6.73	6.57	0.24 (0.81)	0.23 (0.82)	1.49 (0.15)	-0.06 (2.01)	-0.08 (2.19)	-0.56 (1.92)	1.53 (1.28)	1.78 (1.22)	1.65 (1.08)	0.96	0.95	0.96	0.95 (0.89-0.98)	0.95 (0.89-0.98)	0.96 (0.90-0.98)
Left lateral occipital cortex	2.47 (0.09)	2.46 (0.09)	2.47 (0.09)	2.47 (0.08)	3.58	3.76	3.52	3.44	2.58 (0.02)	0.54 (0.59)	2.22 (0.04)	-0.53 (1.06)	-0.09 (0.89)	-0.29 (0.67)	0.80 (0.87)	0.70 (0.54)	0.55 (0.49)	0.96	0.97	0.98	0.95 (0.87-0.98)	0.97 (0.93-0.99)	0.98 (0.95-0.99)
Left lateral orbitofrontal cortex	2.97 (0.12)	2.95 (0.10)	2.96 (0.11)	2.96 (0.12)	4.19	3.45	3.60	3.89	1.34 (0.19)	0.63 (0.54)	0.26 (0.80)	-0.44 (1.68)	-0.19 (1.64)	-0.07 (1.65)	1.18 (1.26)	1.14 (1.17)	1.36 (0.90)	0.92	0.92	0.92	0.90 (0.78-0.95)	0.91 (0.81-0.96)	0.92 (0.83-0.96)
Left lingual gyrus	2.42 (0.13)	2.41 (0.13)	2.41 (0.13)	2.43 (0.14)	5.31	5.37	5.46	5.59	0.79 (0.44)	0.57 (0.58)	-1.42 (0.17)	-0.18 (1.13)	-0.15 (1.30)	0.31 (1.17)	0.86 (0.73)	1.13 (0.62)	1.03 (0.61)	0.98	0.97	0.98	0.98 (0.95-0.99)	0.97 (0.94-0.99)	0.98 (0.95-0.99)
Left medial orbitofrontal cortex	2.74 (0.15)	2.73 (0.14)	2.74 (0.14)	2.73 (0.16)	5.51	5.11	4.98	5.80	1.05 (0.30)	0.17 (0.87)	1.03 (0.31)	-0.54 (2.58)	-0.06 (2.43)	-0.47 (2.12)	2.11 (1.53)	1.81 (1.58)	1.64 (1.39)	0.88	0.89	0.93	0.88 (0.74-0.94)	0.89 (0.77-0.95)	0.92 (0.84-0.97)
Left middle temporal gyrus	3.08 (0.12)	3.06 (0.11)	3.06 (0.10)	3.00 (0.12)	3.79	3.59	3.41	4.00	1.66 (0.11)	2.10 (0.05)	5.57 (0.00)	-0.51 (1.56)	-0.52 (1.25)	-2.56 (2.29)	1.24 (1.05)	1.02 (0.87)	2.86 (1.90)	0.91	0.94	0.83	0.91 (0.80-0.96)	0.93 (0.84-0.97)	0.68 (0.02-0.89)
Left parahippocampal gyrus	3.15 (0.24)	3.15 (0.25)	3.16 (0.24)	3.18 (0.24)	7.52	7.84	7.62	7.57	-0.81 (0.43)	-1.02 (0.32)	-3.15 (0.00)	0.26 (1.72)	0.50 (2.47)	1.04 (1.67)	1.34 (1.08)	1.89 (1.63)	1.46 (1.29)	0.97	0.94	0.98	0.97 (0.94-0.99)	0.94 (0.88-0.97)	0.97 (0.89-0.99)
Left paracentral lobule	2.69 (0.14)	2.69 (0.15)	2.70 (0.14)	2.70 (0.15)	5.36	5.68	5.09	5.38	0.49 (0.63)	-0.99 (0.33)	-1.17 (0.26)	-0.15 (1.38)	0.29 (1.40)	0.25 (1.09)	1.12 (0.78)	1.07 (0.92)	0.93 (0.59)	0.97	0.97	0.98	0.97 (0.93-0.99)	0.96 (0.92-0.98)	0.98 (0.96-0.99)
Left pars opercularis	2.87 (0.09)	2.86 (0.10)	2.87 (0.10)	2.87 (0.10)	3.26	3.44	3.46	3.56	1.09 (0.29)	-0.34 (0.73)	0.22 (0.83)	-0.33 (1.48)	0.10 (1.51)	-0.07 (1.30)	1.10 (1.01)	1.22 (0.87)	0.99 (0.82)	0.91	0.90	0.93	0.90 (0.80-0.96)	0.90 (0.79-0.96)	0.93 (0.85-0.97)
Left pars orbitalis	2.97 (0.15)	2.96 (0.12)	2.98 (0.15)	2.95 (0.14)	4.95	4.21	4.90	4.77	0.43 (0.67)	-0.88 (0.39)	1.42 (0.17)	-0.16 (2.27)	0.33 (1.86)	-0.65 (2.29)	1.43 (1.74)	1.58 (0.99)	1.80 (1.52)	0.89	0.93	0.89	0.88 (0.75-0.94)	0.93 (0.84-0.97)	0.88 (0.75-0.95)
Left pars triangularis	2.77 (0.14)	2.77 (0.12)	2.78 (0.13)	2.73 (0.13)	4.90	4.35	4.78	4.73	-0.98 (0.34)	-0.82 (0.42)	3.08 (0.01)	0.35 (1.71)	0.38 (2.35)	-1.24 (2.05)	1.26 (1.19)	1.92 (1.37)	1.89 (1.43)	0.94	0.89	0.91	0.93 (0.86-0.97)	0.89 (0.76-0.95)	0.89 (0.68-0.95)
Left pericalcarine cortex	1.90 (0.14)	1.89 (0.13)	1.89 (0.15)	1.89 (0.14)	7.26	7.11	7.84	7.16	0.55 (0.59)	1.19 (0.25)	0.84 (0.41)	-0.22 (2.07)	-0.46 (1.75)	-0.30 (1.86)	1.58 (1.31)	1.46 (1.04)	1.49 (1.11)	0.96	0.97	0.97	0.96 (0.91-0.98)	0.97 (0.94-0.99)	0.97 (0.93-0.99)
Left postcentral gyrus	2.38 (0.14)	2.37 (0.14)	2.38 (0.13)	2.38 (0.13)	5.76	5.71	5.62	5.53	1.62 (0.12)	0.14 (0.89)	0.62 (0.54)	-0.51 (1.56)	-0.03 (1.16)	-0.14 (1.20)	1.10 (1.20)	0.90 (0.72)	0.79 (0.90)	0.96	0.98	0.98	0.96 (0.91-0.98)	0.98 (0.95-0.99)	0.98 (0.95-0.99)
Left posterior cingulate cortex	2.77 (0.15)	2.76 (0.14)	2.77 (0.14)	2.76 (0.14)	5.35	5.20	5.20	5.10	1.06 (0.30)	-0.96 (0.35)	0.29 (0.78)	-0.32 (1.51)	0.26 (1.31)	-0.07 (1.40)	1.21 (0.93)	1.05 (0.81)	1.07 (0.88)	0.96	0.97	0.96	0.96 (0.91-0.98)	0.97 (0.93-0.99)	0.96 (0.92-0.98)
Left precentral gyrus	2.74 (0.13)	2.73 (0.13)	2.73 (0.13)	2.74 (0.14)	4.93	4.90	4.69	4.99	1.97 (0.06)	0.59 (0.56)	0.62 (0.54)	-0.53 (1.33)	-0.17 (1.54)	-0.16 (1.23)	1.05 (0.96)	1.13 (1.04)	1.00 (0.70)	0.96	0.95	0.97	0.96 (0.90-0.98)	0.95 (0.89-0.98)	0.97 (0.94-0.99)
Left precuneus	2.76 (0.11)	2.75 (0.10)	2.76 (0.10)	2.76 (0.10)	3.89	3.75	3.59	3.47	1.33 (0.20)	-0.60 (0.55)	-0.31 (0.76)	-0.24 (0.91)	0.13 (0.97)	0.08 (1.08)	0.73 (0.58)	0.82 (0.51)	0.88 (0.61)	0.97	0.97	0.96	0.97 (0.94-0.99)	0.97 (0.93-0.99)	0.96 (0.91-0.98)
Left rostral anterior cingulate cortex	3.12 (0.15)	3.09 (0.13)	3.12 (0.12)	3.11 (0.14)	4.66	4.27	3.94	4.55	1.58 (0.13)	-0.22 (0.83)	0.44 (0.66)	-0.84 (2.72)	0.15 (2.67)	-0.23 (2.76)	2.22 (1.73)	2.02 (1.70)	2.06 (1.79)	0.82	0.81	0.82	0.81 (0.62-0.91)	0.81 (0.61-0.91)	0.83 (0.65-0.92)
Left rostral middle frontal gyrus	2.59 (0.12)	2.60 (0.12)	2.59 (0.11)	2.58 (0.12)	4.47	4.44	4.26	4.62	-0.35 (0.73)	0.71 (0.48)	2.06 (0.05)	0.10 (1.35)	-0.26 (1.89)	-0.63 (1.53)	1.05 (0.83)	1.51 (1.12)	1.28 (1.02)	0.95	0.91	0.94	0.96 (0.90-0.98)	0.91 (0.80-0.96)	0.94 (0.86-0.97)
Left superior frontal gyrus	2.98 (0.13)	2.97 (0.12)	2.97 (0.12)	2.98 (0.13)	4.22	3.99	3.88	4.28	0.74 (0.47)	2.09 (0.05)	-0.33 (0.75)	-0.21 (1.50)	-0.48 (1.19)	0.08 (1.32)	1.18 (0.91)	0.99 (0.80)	1.03 (0.80)	0.94	0.96	0.95	0.94 (0.86-0.97)	0.95 (0.89-0.98)	0.95 (0.90-0.98)
Left superior parietal cortex	2.50 (0.11)	2.49 (0.12)	2.51 (0.11)	2.51 (0.11)	4.43	4.72	4.44	4.55	1.52 (0.14)	-0.46 (0.65)	-0.37 (0.71)	-0.39 (1.26)	0.10 (1.06)	0.08 (1.13)	0.89 (0.95)	0.87 (0.57)	0.83 (0.76)	0.96	0.97	0.97	0.96 (0.91-0.98)	0.97 (0.94-0.99)	0.97 (0.93-0.99)
Left superior temporal gyrus	3.06 (0.16)	3.06 (0.16)	3.06 (0.16)	3.07 (0.16)	5.26	5.36	5.27	5.26	0.55 (0.59)	0.46 (0.65)	-0.29 (0.77)	-0.14 (1.21)	-0.08 (0.90)	0.07 (1.17)	0.94 (0.76)	0.68 (0.58)	0.97 (0.64)	0.97	0.99	0.98	0.97 (0.94-0.99)	0.99 (0.97-0.99)	0.98 (0.95-0.99)
Left supramarginal gyrus	2.85 (0.11)	2.84 (0.11)	2.85 (0.11)	2.86 (0.11)	3.82	3.92	3.75	3.81	0.92 (0.37)	0.31 (0.76)	-0.65 (0.52)	-0.31 (1.68)	-0.07 (1.23)	0.16 (1.25)	1.41 (0.92)	0.92 (0.80)	0.95 (0.81)	0.91	0.95	0.95	0.91 (0.80-0.96)	0.95 (0.89-0.98)	0.95 (0.89-0.98)
Left frontal pole	3.08 (0.33)	3.07 (0.31)	2.80 (0.34)	3.07 (0.31)	10.71	10.08	12.12	10.15	0.54 (0.60)	4.01 (0.00)	0.55 (0.58)	-0.33 (3.58)	-9.78 (12.42)	-0.41 (3.96)	2.54 (2.48)	10.48 (11.81)	2.91 (2.66)	0.94	0.44	0.92	0.94 (0.87-0.97)	0.33 (-0.05-0.63)	0.92 (0.84-0.97)
Left temporal pole	3.74 (0.27)	3.74 (0.31)	3.76 (0.24)	3.74 (0.24)	7.29	8.19	6.44	6.53	-0.02 (0.99)	-0.81 (0.43)	0.12 (0.91)	-0.07 (3.14)	0.49 (2.79)	-0.03 (3.63)	2.40 (1.97)	1.96 (2.01)	2.68 (2.38)	0.93	0.93	0.88	0.92 (0.83-0.97)	0.93 (0.84-0.97)	0.88 (0.74-0.94)
Left transverse temporal gyrus	2.95 (0.21)	2.94 (0.21)	2.95 (0.23)	2.94 (0.23)	7.25	7.19	7.67	7.70	0.84 (0.41)	-0.42 (0.68)	0.66 (0.52)	-0.24 (1.42)	0.09 (1.49)	-0.23 (1.49)	1.17 (0.80)	1.21 (0.84)	1.20 (0.88)	0.98	0.98	0.98	0.98 (0.96-0.99)	0.98 (0.96-0.99)	0.98 (0.96-0.99)
Left insula	3.28 (0.20)	3.32 (0.16)	3.31 (0.17)	3.30 (0.17)	5.99	4.91	5.05	5.17	-1.61 (0.12)	-1.12 (0.27)	-1.03 (0.31)	1.35 (4.14)	0.93 (4.06)	0.73 (3.46)	3.01 (3.11)	2.58 (3.24)	2.58 (2.36)	0.75	0.76	0.83	0.72 (0.47-0.87)	0.75 (0.51-0.88)	0.82 (0.64-0.92)
Right banks superior temporal sulcus	2.86 (0.21)	2.85 (0.22)	2.86 (0.20)	2.87 (0.20)	7.23	7.60	7.04	6.85	0.02 (0.99)	-0.91 (0.37)	-1.59 (0.12)	-0.03 (1.89)	0.33 (1.78)	0.62 (1.90)	1.56 (1.03)	1.24 (1.30)	1.48 (1.31)	0.97	0.97	0.97	0.97 (0.93-0.99)	0.97 (0.93-0.99)	0.96 (0.92-0.98)

Right caudal anterior cingulate cortex	2.77 (0.20)	2.77 (0.18)	2.76 (0.20)	2.77 (0.19)	7.05	6.62	7.14	6.89	-0.43 (0.67)	0.76 (0.46)	0.13 (0.89)	0.27 (2.77)	-0.34 (2.27)	-0.04 (2.20)	2.25 (1.57)	1.84 (1.33)	1.84 (1.15)	0.92	0.95	0.95	0.92 (0.83-0.96)	0.95 (0.89-0.98)	0.95 (0.89-0.98)
Right caudal middle frontal gyrus	2.80 (0.12)	2.80 (0.12)	2.81 (0.12)	2.80 (0.12)	4.37	4.13	4.21	4.23	0.13 (0.90)	-0.33 (0.74)	-0.26 (0.80)	-0.02 (1.40)	0.08 (1.12)	0.07 (1.31)	1.13 (0.80)	0.88 (0.67)	1.10 (0.68)	0.95	0.97	0.95	0.95 (0.88-0.98)	0.97 (0.93-0.99)	0.95 (0.90-0.98)
Right cuneus	2.25 (0.13)	2.25 (0.13)	2.24 (0.14)	2.25 (0.14)	5.61	5.85	6.07	6.23	0.92 (0.37)	1.72 (0.10)	-0.01 (0.99)	-0.21 (1.07)	-0.51 (1.36)	-0.03 (1.27)	0.91 (0.57)	1.19 (0.81)	1.04 (0.69)	0.98	0.97	0.98	0.98 (0.96-0.99)	0.97 (0.93-0.99)	0.98 (0.95-0.99)
Right entorhinal cortex	3.48 (0.31)	3.46 (0.30)	3.45 (0.32)	3.48 (0.29)	8.97	8.65	9.24	8.42	0.56 (0.58)	0.67 (0.51)	-0.02 (0.98)	-0.40 (4.00)	-0.66 (5.06)	0.06 (3.74)	2.90 (2.73)	3.29 (3.84)	2.50 (2.73)	0.90	0.86	0.91	0.91 (0.80-0.96)	0.86 (0.71-0.94)	0.91 (0.81-0.96)
Right fusiform gyrus	2.94 (0.10)	2.94 (0.11)	2.94 (0.11)	2.93 (0.10)	3.27	3.69	3.68	3.45	-0.45 (0.65)	-1.11 (0.28)	0.42 (0.68)	0.11 (1.35)	0.20 (0.96)	-0.08 (0.92)	1.08 (0.78)	0.77 (0.59)	0.73 (0.54)	0.93	0.97	0.96	0.93 (0.84-0.97)	0.96 (0.91-0.98)	0.96 (0.92-0.98)
Right inferior parietal cortex	2.74 (0.12)	2.74 (0.12)	2.75 (0.12)	2.74 (0.12)	4.36	4.50	4.21	4.35	-0.22 (0.83)	-1.49 (0.15)	-0.96 (0.35)	0.06 (1.53)	0.39 (1.31)	0.24 (1.25)	1.20 (0.92)	0.99 (0.92)	0.93 (0.85)	0.94	0.96	0.96	0.94 (0.88-0.98)	0.95 (0.90-0.98)	0.96 (0.91-0.98)
Right inferior temporal gyrus	2.87 (0.12)	2.87 (0.12)	2.88 (0.13)	2.87 (0.12)	4.34	4.05	4.39	4.09	0.00 (1.00)	-1.99 (0.06)	0.11 (0.91)	0.01 (1.85)	0.46 (1.15)	-0.02 (1.35)	1.38 (1.20)	0.95 (0.77)	1.08 (0.78)	0.91	0.97	0.95	0.91 (0.80-0.96)	0.96 (0.91-0.98)	0.95 (0.89-0.98)
Right isthmus cingulate cortex	2.72 (0.24)	2.72 (0.22)	2.72 (0.24)	2.71 (0.23)	8.96	8.01	8.74	8.42	-0.06 (0.95)	-0.17 (0.87)	1.04 (0.31)	0.10 (2.11)	0.10 (2.08)	-0.25 (1.39)	1.68 (1.23)	1.51 (1.40)	1.23 (0.63)	0.98	0.97	0.99	0.97 (0.93-0.99)	0.97 (0.93-0.99)	0.99 (0.97-0.99)
Right lateral occipital cortex	2.46 (0.08)	2.46 (0.08)	2.47 (0.08)	2.46 (0.07)	3.16	3.23	3.21	2.94	0.12 (0.90)	-2.23 (0.04)	-0.35 (0.73)	-0.04 (1.37)	0.32 (0.71)	0.08 (1.09)	1.06 (0.83)	0.63 (0.45)	0.88 (0.61)	0.91	0.97	0.94	0.91 (0.81-0.96)	0.97 (0.93-0.99)	0.94 (0.86-0.97)
Right lateral orbitofrontal cortex	2.86 (0.13)	2.88 (0.12)	2.88 (0.10)	2.88 (0.11)	4.43	4.20	3.49	3.79	-1.42 (0.17)	-1.65 (0.11)	-1.52 (0.14)	0.77 (2.72)	0.90 (2.65)	0.91 (2.91)	2.07 (1.89)	1.97 (1.95)	2.26 (2.00)	0.81	0.81	0.76	0.80 (0.60-0.91)	0.77 (0.55-0.89)	0.74 (0.50-0.88)
Right lingual gyrus	2.42 (0.14)	2.42 (0.14)	2.42 (0.14)	2.42 (0.13)	5.60	5.69	5.93	5.53	0.05 (0.96)	0.54 (0.60)	0.34 (0.74)	-0.02 (1.22)	-0.13 (1.04)	-0.06 (0.86)	0.90 (0.80)	0.85 (0.59)	0.69 (0.50)	0.98	0.98	0.99	0.98 (0.95-0.99)	0.98 (0.96-0.99)	0.99 (0.97-0.99)
Right medial orbitofrontal cortex	2.82 (0.13)	2.82 (0.14)	2.82 (0.14)	2.82 (0.14)	4.62	4.94	4.93	4.86	-0.24 (0.81)	0.26 (0.79)	-0.12 (0.90)	0.08 (1.99)	-0.10 (1.68)	0.04 (1.92)	1.61 (1.13)	1.34 (0.98)	1.56 (1.08)	0.92	0.94	0.92	0.92 (0.82-0.96)	0.94 (0.86-0.97)	0.92 (0.83-0.96)
Right middle temporal gyrus	3.12 (0.12)	3.12 (0.11)	3.10 (0.14)	3.06 (0.11)	3.83	3.60	4.67	3.63	-0.14 (0.89)	1.42 (0.17)	4.44 (0.00)	0.05 (1.42)	-0.69 (2.33)	-1.76 (2.00)	1.03 (0.95)	1.56 (1.84)	2.08 (1.64)	0.93	0.87	0.86	0.93 (0.85-0.97)	0.85 (0.69-0.93)	0.77 (0.26-0.92)
Right parahippocampal gyrus	3.02 (0.19)	3.02 (0.19)	3.02 (0.18)	3.02 (0.18)	6.38	6.30	5.99	5.99	0.03 (0.98)	-0.29 (0.78)	-0.46 (0.65)	-0.01 (1.93)	0.12 (1.69)	0.19 (1.76)	1.54 (1.12)	1.33 (1.01)	1.32 (1.16)	0.95	0.96	0.96	0.96 (0.90-0.98)	0.96 (0.92-0.98)	0.96 (0.91-0.98)
Right paracentral lobule	2.70 (0.15)	2.71 (0.15)	2.71 (0.15)	2.70 (0.15)	5.49	5.66	5.64	5.56	-0.77 (0.45)	-1.29 (0.21)	-0.63 (0.53)	0.22 (1.47)	0.28 (1.10)	0.12 (1.03)	0.98 (1.10)	0.81 (0.78)	0.79 (0.64)	0.97	0.98	0.98	0.97 (0.92-0.98)	0.98 (0.95-0.99)	0.98 (0.96-0.99)
Right pars opercularis	2.91 (0.11)	2.89 (0.12)	2.90 (0.10)	2.91 (0.11)	3.66	4.04	3.53	3.93	1.74 (0.10)	0.65 (0.52)	0.20 (0.85)	-0.71 (2.01)	-0.15 (1.18)	-0.07 (1.61)	1.59 (1.39)	0.93 (0.72)	1.10 (1.16)	0.87	0.95	0.91	0.86 (0.70-0.93)	0.95 (0.88-0.98)	0.91 (0.81-0.96)
Right pars orbitalis	3.04 (0.16)	3.02 (0.15)	3.03 (0.15)	3.00 (0.15)	5.22	5.10	4.90	5.06	1.49 (0.15)	0.56 (0.58)	3.18 (0.00)	-0.69 (2.33)	-0.17 (1.68)	-1.24 (1.95)	1.79 (1.60)	1.29 (1.05)	1.89 (1.29)	0.90	0.95	0.93	0.89 (0.77-0.95)	0.95 (0.89-0.98)	0.90 (0.71-0.96)
Right pars triangularis	2.85 (0.12)	2.84 (0.13)	2.84 (0.13)	2.83 (0.14)	4.39	4.54	4.65	4.86	0.93 (0.36)	1.18 (0.25)	1.54 (0.14)	-0.34 (1.76)	-0.35 (1.40)	-0.58 (1.82)	1.31 (1.20)	1.22 (0.73)	1.51 (1.14)	0.92	0.95	0.93	0.92 (0.83-0.96)	0.95 (0.89-0.98)	0.92 (0.83-0.96)
Right pericalcarine cortex	1.96 (0.17)	1.96 (0.17)	1.96 (0.18)	1.96 (0.18)	8.87	8.85	9.16	9.02	-0.01 (0.99)	0.62 (0.54)	0.57 (0.57)	0.01 (1.69)	-0.29 (2.20)	-0.24 (2.02)	1.27 (1.09)	1.61 (1.49)	1.56 (1.28)	0.98	0.97	0.98	0.98 (0.96-0.99)	0.97 (0.94-0.99)	0.98 (0.95-0.99)
Right postcentral gyrus	2.36 (0.15)	2.35 (0.14)	2.36 (0.15)	2.36 (0.14)	6.18	6.09	6.55	6.13	0.89 (0.38)	-0.29 (0.77)	-0.06 (0.95)	-0.52 (2.85)	0.05 (1.22)	0.02 (1.25)	1.34 (2.55)	1.00 (0.66)	1.05 (0.65)	0.89	0.98	0.98	0.89 (0.76-0.95)	0.98 (0.96-0.99)	0.98 (0.96-0.99)
Right posterior cingulate cortex	2.74 (0.14)	2.75 (0.16)	2.75 (0.15)	2.75 (0.15)	5.23	5.78	5.34	5.29	-0.77 (0.45)	-1.04 (0.31)	-0.68 (0.50)	0.18 (1.32)	0.41 (1.98)	0.18 (1.34)	1.05 (0.80)	1.39 (1.44)	0.95 (0.94)	0.97	0.93	0.97	0.97 (0.93-0.99)	0.93 (0.84-0.97)	0.97 (0.93-0.99)
Right precentral gyrus	2.70 (0.15)	2.72 (0.12)	2.71 (0.14)	2.71 (0.14)	5.38	4.51	5.02	5.07	-1.03 (0.31)	-0.28 (0.78)	-1.31 (0.20)	0.70 (3.29)	0.08 (1.13)	0.32 (1.18)	1.57 (2.97)	0.85 (0.73)	0.97 (0.72)	0.81	0.98	0.98	0.80 (0.60-0.90)	0.98 (0.95-0.99)	0.98 (0.95-0.99)
Right precuneus	2.74 (0.11)	2.74 (0.11)	2.74 (0.11)	2.74 (0.10)	4.11	3.91	3.97	3.81	0.55 (0.59)	0.04 (0.97)	0.15 (0.88)	-0.12 (1.17)	0.00 (0.75)	-0.02 (0.92)	0.87 (0.77)	0.58 (0.47)	0.71 (0.57)	0.96	0.98	0.98	0.96 (0.91-0.98)	0.98 (0.96-0.99)	0.97 (0.94-0.99)
Right rostral anterior cingulate cortex	3.24 (0.19)	3.22 (0.17)	3.24 (0.14)	3.21 (0.16)	5.85	5.19	4.33	4.93	1.13 (0.27)	0.19 (0.85)	1.75 (0.09)	-0.42 (2.01)	-0.03 (2.63)	-1.01 (2.97)	1.75 (1.02)	2.25 (1.28)	2.42 (1.95)	0.94	0.91	0.86	0.93 (0.86-0.97)	0.87 (0.73-0.94)	0.83 (0.66-0.92)
Right rostral middle frontal gyrus	2.68 (0.10)	2.69 (0.09)	2.68 (0.09)	2.67 (0.08)	3.64	3.32	3.51	3.09	-0.34 (0.74)	1.31 (0.20)	1.48 (0.15)	0.10 (1.35)	-0.33 (1.29)	-0.40 (1.37)	0.98 (0.91)	1.11 (0.71)	0.96 (1.05)	0.93	0.94	0.93	0.93 (0.84-0.97)	0.93 (0.86-0.97)	0.91 (0.80-0.96)
Right superior frontal gyrus	3.06 (0.11)	3.06 (0.11)	3.05 (0.11)	3.06 (0.11)	3.61	3.65	3.68	3.50	0.41 (0.68)	2.07 (0.05)	0.28 (0.78)	-0.09 (1.13)	-0.40 (0.97)	-0.05 (1.03)	0.95 (0.59)	0.90 (0.53)	0.86 (0.53)	0.95	0.97	0.96	0.95 (0.90-0.98)	0.96 (0.91-0.98)	0.96 (0.91-0.98)
Right superior parietal cortex	2.48 (0.11)	2.48 (0.11)	2.49 (0.11)	2.48 (0.10)	4.32	4.35	4.38	4.18	0.14 (0.89)	-1.46 (0.16)	0.35 (0.73)	-0.03 (1.18)	0.25 (0.87)	-0.07 (1.03)	1.00 (0.59)	0.67 (0.59)	0.79 (0.65)	0.96	0.98	0.97	0.96 (0.92-0.98)	0.98 (0.95-0.99)	0.97 (0.94-0.99)
Right superior temporal gyrus	3.09 (0.14)	3.09 (0.13)	3.08 (0.14)	3.09 (0.13)	4.61	4.31	4.62	4.35	-0.17 (0.86)	1.41 (0.17)	0.74 (0.46)	0.06 (1.29)	-0.35 (1.24)	-0.20 (1.45)	1.03 (0.76)	0.90 (0.92)	1.01 (1.05)	0.96	0.96	0.95	0.96 (0.91-0.98)	0.96 (0.92-0.98)	0.95 (0.89-0.98)
Right supramarginal gyrus	2.86 (0.10)	2.86 (0.11)	2.86 (0.10)	2.86 (0.10)	3.63	3.83	3.47	3.54	0.24 (0.81)	-0.82 (0.42)	-0.71 (0.48)	-0.06 (1.17)	0.18 (1.03)	0.16 (1.13)	0.94 (0.66)	0.83 (0.62)	0.99 (0.53)	0.95	0.96	0.95	0.95 (0.90-0.98)	0.96 (0.91-0.98)	0.95 (0.89-0.98)
Right frontal pole	3.01 (0.23)	3.03 (0.21)	2.75 (0.28)	3.02 (0.24)	7.73	6.96	10.17	8.10	-0.68 (0.51)	4.16 (0.00)	-0.46 (0.65)	0.79 (5.39)	-9.05 (10.92)	0.49 (5.67)	4.36 (3.15)	10.97 (8.90)	4.41 (3.49)	0.73	0.29	0.74	0.73 (0.49-0.87)	0.20 (-0.10-0.50)	0.75 (0.50-0.88)
Right temporal pole	3.72 (0.33)	3.73 (0.36)	3.71 (0.35)	3.66 (0.42)	8.92	9.72	9.34	11.34	-0.61 (0.55)	0.10 (0.92)	1.34 (0.19)	0.38 (3.76)	-0.11 (3.08)	-1.75 (6.25)	2.85 (2.41)	2.32 (1.97)	4.05 (5.02)	0.92	0.95	0.87	0.92 (0.83-0.97)	0.95 (0.89-0.98)	0.85 (0.68-0.93)
Right transverse temporal gyrus	2.94 (0.21)	2.95 (0.20)	2.96 (0.19)	2.94 (0.20)	7.08	6.65	6.58	6.67	-0.65 (0.52)	-1.63 (0.12)	-0.34 (0.74)	0.23 (1.56)	0.65 (1.90)	0.13 (1.54)	1.12 (1.09)	1.53 (1.27)	1.11 (1.05)	0.98	0.96	0.98	0.97 (0.94-0.99)	0.96 (0.91-0.98)	0.98 (0.94-0.99)
Right insula	3.27 (0.13)	3.27 (0.12)	3.26 (0.15)	3.25 (0.15)	3.84	3.75	4.67	4.56	-0.11 (0.91)	0.88 (0.39)	2.06 (0.05)	0.05 (1.97)	-0.53 (2.85)	-0.87 (2.07)	1.33 (1.43)	2.19 (1.85)	1.62 (1.53)	0.87	0.80	0.89	0.88 (0.74-0.94)	0.79 (0.58-0.90)	0.87 (0.72-0.94)
Global brain measures																							
Left total cortical volume (ml)	300.22 (25.53)	299.82 (25.75)	299.47 (25.28)	299.50 (25.95)	8.50	8.59	8.44	8.66	0.83 (0.41)	1.62 (0.12)	1.55 (0.13)	-0.14 (0.84)	-0.24 (0.77)	-0.25 (0.78)	0.63 (0.55)	0.64 (0.47)	0.67 (0.44)	1.00	1.00	1.00	1.00 (0.99-1.00)	1.00 (0.99-1.00)	1.00 (0.99-1.00)
Right total cortical volume (ml)	302.42 (25.83)	302.55 (25.85)	301.41 (24.84)	301.64 (25.48)	8.54	8.54	8.24	8.45	-0.30 (0.76)	2.10 (0.05)	1.68 (0.11)	0.05 (0.74)	-0.31 (0.76)	-0.25 (0.74)	0.58 (0.45)	0.60 (0.54)	0.59 (0.50)	1.00	1.00	1.00	1.00 (0.99-1.00)	0.99 (0.99-1.00)	1.00 (0.99-1.00)
Left total cortical white matter (ml)	206.04 (18.70)	206.02 (18.92)	205.93 (18.89)	206.21 (18.87)	9.08	9.18	9.17	9.15	0.11 (0.92)	0.70 (0.49)	-1.06 (0.30)	-0.02 (0.39)	-0.06 (0.35)	0.08 (0.40)	0.32 (0.21)	0.28 (0.21)	0.31 (0.26)	1.00	1.00	1.00	1.00 (1.00-1.00)	1.00 (1.00-1.00)	1.00 (1.00-1.00)
Right total cortical white matter (ml)	205.74 (18.70)	205.67 (18.94)	205.58 (18.89)	205.64	9.09	9.21	9.19	9.17	0.41 (0.68)	1.00 (0.33)	0.55 (0.58)	-0.04 (0.43)	-0.09 (0.40)	-0.06 (0.44)	0.34 (0.25)	0.30 (0.27)	0.30 (0.32)	1.00	1.00	1.00	1.00 (1.00-1.00)	1.00 (1.00-1.00)	1.00 (1.00-1.00)

				(18.87)																			
Left total cortical surface area (cm²)	937.46 (76.21)	939.09 (77.59)	937.91 (77.02)	940.04 (76.51)	8.13	8.26	8.21	8.14	-2.57 (0.02)	-0.80 (0.43)	-3.76 (0.00)	0.16 (0.33)	0.04 (0.29)	0.27 (0.36)	0.28 (0.24)	0.22 (0.19)	0.38 (0.23)	1.00	1.00	1.00	1.00 (1.00-1.00)	1.00 (1.00-1.00)	1.00 (0.99-1.00)
Right total cortical surface area (cm²)	940.55 (78.12)	941.38 (79.21)	940.13 (78.56)	941.83 (77.18)	8.31	8.41	8.36	8.19	-1.36 (0.19)	0.63 (0.53)	-1.63 (0.12)	0.08 (0.32)	-0.05 (0.36)	0.14 (0.42)	0.27 (0.17)	0.28 (0.23)	0.35 (0.26)	1.00	1.00	1.00	1.00 (1.00-1.00)	1.00 (1.00-1.00)	1.00 (1.00-1.00)
Left mean cortical thickness (mm)	2.76 (0.08)	2.76 (0.07)	2.76 (0.07)	2.76 (0.08)	2.75	2.65	2.54	2.73	1.19 (0.25)	0.71 (0.48)	1.03 (0.31)	-0.25 (1.05)	-0.12 (0.86)	-0.19 (0.92)	0.82 (0.69)	0.71 (0.49)	0.78 (0.50)	0.93	0.95	0.94	0.92 (0.84-0.97)	0.95 (0.89-0.98)	0.94 (0.88-0.97)
Right mean cortical tickness (mm)	2.76 (0.07)	2.76 (0.07)	2.76 (0.07)	2.76 (0.06)	2.54	2.48	2.56	2.29	-0.14 (0.89)	0.13 (0.90)	0.54 (0.59)	0.03 (0.89)	-0.02 (0.66)	-0.08 (0.77)	0.70 (0.53)	0.51 (0.41)	0.62 (0.45)	0.94	0.97	0.95	0.94 (0.87-0.97)	0.97 (0.93-0.99)	0.95 (0.89-0.98)
Left cerebellar white matter (ml)	14.01 (1.53)	13.94 (1.36)	13.93 (1.49)	13.78 (1.43)	10.95	9.74	10.71	10.37	0.92 (0.36)	1.18 (0.25)	3.10 (0.00)	-0.38 (2.52)	-0.53 (2.31)	-1.59 (2.67)	2.04 (1.48)	1.95 (1.28)	2.35 (2.00)	0.97	0.98	0.97	0.97 (0.93-0.99)	0.98 (0.95-0.99)	0.96 (0.87-0.98)
Right cerebellar white matter (ml)	13.49 (1.51)	13.47 (1.42)	13.49 (1.48)	13.31 (1.43)	11.23	10.52	10.97	10.73	0.27 (0.79)	-0.05 (0.96)	2.60 (0.02)	-0.04 (2.15)	0.07 (2.80)	-1.25 (2.48)	1.70 (1.27)	2.22 (1.64)	2.13 (1.75)	0.98	0.97	0.98	0.98 (0.96-0.99)	0.97 (0.93-0.99)	0.97 (0.91-0.99)
Left cerebellar gray matter (ml)	56.08 (3.50)	56.00 (3.46)	56.00 (3.56)	56.30 (3.48)	6.25	6.18	6.36	6.18	0.80 (0.43)	1.42 (0.17)	-3.40 (0.00)	-0.13 (0.85)	-0.15 (0.52)	0.40 (0.59)	0.53 (0.66)	0.42 (0.33)	0.56 (0.43)	0.99	1.00	1.00	0.99 (0.98-1.00)	1.00 (0.99-1.00)	0.99 (0.98-1.00)
Right cerebellar gray matter (ml)	55.94 (3.88)	55.84 (3.86)	55.92 (3.82)	56.24 (3.72)	6.93	6.91	6.84	6.62	1.20 (0.24)	0.18 (0.86)	-3.96 (0.00)	-0.18 (0.80)	-0.02 (0.83)	0.55 (0.69)	0.55 (0.61)	0.63 (0.53)	0.73 (0.49)	0.99	0.99	1.00	0.99 (0.99-1.00)	0.99 (0.98-1.00)	0.99 (0.96-1.00)
Intracranial volume (ml)	1505.60 (102.22)	1501.25 (107.35)	1460.16 (104.25)	1506.29 (102.87)	6.79	7.15	7.14	6.83	1.16 (0.26)	7.01 (0.00)	-0.74 (0.47)	-0.31 (1.36)	-3.09 (2.32)	0.04 (0.30)	0.39 (1.34)	3.09 (2.32)	0.25 (0.18)	0.99	0.95	1.00	0.98 (0.96-0.99)	0.87 (0.08-0.96)	1.00 (1.00-1.00)
Total brain volume without ventricles (ml)	1212.24 (90.79)	1211.72 (91.63)	1210.12 (89.85)	1210.77 (90.94)	7.49	7.56	7.42	7.51	0.61 (0.55)	2.31 (0.03)	1.84 (0.08)	-0.05 (0.36)	-0.17 (0.38)	-0.12 (0.33)	0.26 (0.24)	0.33 (0.24)	0.30 (0.18)	1.00	1.00	1.00	1.00 (1.00-1.00)	1.00 (1.00-1.00)	1.00 (1.00-1.00)

Abbreviations: SD = standard deviation; Orig = original scan; FS df = FreeSurfer defacing; FSL df = FSL defacing; FM = Face Masking; t = t-statistic; *p*=p-value; PD = Average Percentage Difference; CI = Confidence Interval.

Table S2. Effects of de-identification on brain measures in young adults.

Brain metric	Mean (SD)				Coefficient of Variation				Paired t-test t(*p*)			Signed PD in %			Absolute PD (%)			Pearson's r			ICC (95% CI)		
	Orig	FS df	FSL df	FM	Orig	FS df	FSL df	FM	FS df	FSL df	FM	FS df	FSL df	FM	FS df	FSL df	FM	FS df	FSL df	FM	FS df	FSL df	FM
Subcortical volume (ml)																							
Left thalamus	7.58 (0.91)	7.56 (0.95)	7.59 (0.88)	7.58 (0.83)	12.06	12.55	11.61	10.95	0.53 (0.61)	-0.12 (0.91)	-0.10 (0.92)	-0.35 (2.18)	0.13 (2.67)	0.18 (2.41)	1.75 (1.28)	2.18 (1.45)	1.61 (1.75)	0.98	0.98	0.99	0.98 (0.96-0.99)	0.98 (0.93-0.99)	0.98 (0.95-0.99)
Left caudatus	3.85 (0.40)	3.85 (0.41)	3.84 (0.39)	3.82 (0.39)	10.28	10.62	10.24	10.26	0.65 (0.53)	1.09 (0.29)	2.96 (0.01)	-0.22 (1.10)	-0.30 (1.08)	-0.79 (1.04)	0.90 (0.63)	0.92 (0.60)	1.09 (0.70)	0.99	0.99	0.99	0.99 (0.98-1.00)	0.99 (0.98-1.00)	0.99 (0.96-1.00)
Left putamen	5.26 (0.74)	5.28 (0.70)	5.27 (0.72)	5.27 (0.69)	14.01	13.32	13.65	13.07	-0.61 (0.55)	-0.49 (0.63)	-0.43 (0.67)	0.54 (3.34)	0.24 (1.64)	0.41 (2.99)	1.78 (2.84)	1.35 (0.89)	1.45 (2.63)	0.98	0.99	0.98	0.98 (0.94-0.99)	0.99 (0.98-1.00)	0.98 (0.95-0.99)
Left pallidum	1.92 (0.24)	1.96 (0.24)	1.94 (0.23)	1.93 (0.22)	12.57	12.36	11.83	11.60	-1.88 (0.08)	-1.54 (0.14)	-0.43 (0.67)	2.13 (4.55)	1.11 (2.66)	0.47 (3.30)	3.67 (3.34)	2.14 (1.88)	2.57 (2.03)	0.94	0.98	0.97	0.93 (0.79-0.97)	0.97 (0.93-0.99)	0.96 (0.90-0.99)
Left hippocampus	4.11 (0.34)	4.12 (0.35)	4.11 (0.34)	4.13 (0.36)	8.32	8.49	8.24	8.80	-0.41 (0.69)	0.20 (0.84)	-1.21 (0.25)	0.23 (2.42)	-0.12 (2.38)	0.53 (1.87)	2.06 (1.18)	1.77 (1.53)	1.37 (1.34)	0.96	0.96	0.98	0.96 (0.89-0.99)	0.96 (0.89-0.99)	0.98 (0.93-0.99)
Left amygdala	1.73 (0.23)	1.74 (0.24)	1.74 (0.24)	1.72 (0.25)	13.41	13.48	13.93	14.55	-1.09 (0.29)	-0.60 (0.56)	0.30 (0.77)	0.99 (3.54)	0.55 (3.95)	-0.43 (3.53)	3.00 (1.99)	3.26 (2.15)	2.82 (2.05)	0.96	0.96	0.97	0.96 (0.90-0.99)	0.96 (0.89-0.99)	0.97 (0.92-0.99)
Left accumbens	0.62 (0.14)	0.64 (0.14)	0.64 (0.15)	0.65 (0.16)	22.05	22.74	23.46	24.21	-1.26 (0.23)	-1.38 (0.19)	-2.10 (0.05)	1.68 (6.98)	1.60 (5.32)	4.16 (8.29)	4.53 (5.46)	4.58 (2.95)	7.83 (4.67)	0.96	0.97	0.94	0.96 (0.89-0.99)	0.97 (0.91-0.99)	0.92 (0.75-0.97)
Right thalamus	7.21 (0.85)	7.19 (0.84)	7.23 (0.83)	7.23 (0.85)	11.81	11.71	11.48	11.71	0.49 (0.63)	-0.42 (0.68)	-0.46 (0.65)	-0.26 (1.97)	0.28 (2.23)	0.25 (2.03)	1.59 (1.13)	1.75 (1.34)	1.71 (1.03)	0.98	0.98	0.99	0.99 (0.96-0.99)	0.98 (0.95-0.99)	0.99 (0.96-1.00)
Right caudatus	3.92 (0.37)	3.89 (0.37)	3.91 (0.37)	3.93 (0.39)	9.53	9.40	9.46	9.90	1.97 (0.07)	0.22 (0.83)	-0.81 (0.43)	-0.75 (1.56)	-0.11 (2.01)	0.30 (1.57)	1.32 (1.09)	1.30 (1.50)	1.15 (1.08)	0.99	0.97	0.99	0.98 (0.95-0.99)	0.98 (0.93-0.99)	0.99 (0.96-1.00)
Right putamen	5.29 (0.66)	5.27 (0.68)	5.31 (0.67)	5.27 (0.66)	12.48	12.97	12.55	12.46	1.60 (0.13)	-1.62 (0.13)	1.65 (0.12)	-0.39 (0.81)	0.30 (0.77)	-0.46 (1.21)	0.79 (0.39)	0.69 (0.42)	0.99 (0.81)	1.00	1.00	1.00	1.00 (0.99-1.00)	1.00 (0.99-1.00)	1.00 (0.99-1.00)
Right pallidum	1.89 (0.20)	1.90 (0.22)	1.89 (0.22)	1.87 (0.21)	10.85	11.53	11.91	11.27	-1.09 (0.29)	-0.27 (0.79)	0.96 (0.35)	0.53 (2.22)	0.06 (2.69)	-0.94 (3.70)	1.75 (1.40)	2.07 (1.63)	2.53 (2.80)	0.98	0.98	0.95	0.98 (0.95-0.99)	0.98 (0.93-0.99)	0.95 (0.85-0.98)
Right hippocampus	4.25 (0.34)	4.27 (0.35)	4.27 (0.35)	4.26 (0.34)	7.94	8.25	8.12	8.00	-0.83 (0.42)	-1.69 (0.11)	-0.55 (0.59)	0.30 (1.58)	0.45 (1.14)	0.14 (1.03)	1.23 (0.99)	1.02 (0.63)	0.88 (0.50)	0.98	0.99	0.99	0.98 (0.95-0.99)	0.99 (0.97-1.00)	0.99 (0.98-1.00)
Right amygdala	1.90 (0.24)	1.87 (0.25)	1.87 (0.25)	1.85 (0.24)	12.73	13.33	13.20	12.96	2.40 (0.03)	1.76 (0.10)	2.06 (0.06)	-1.92 (3.19)	-1.88 (4.05)	-2.99 (5.78)	2.45 (2.77)	3.28 (2.95)	4.97 (4.09)	0.97	0.95	0.90	0.96 (0.87-0.99)	0.94 (0.84-0.98)	0.88 (0.68-0.96)
Right accumbens	0.63 (0.12)	0.63 (0.13)	0.63 (0.12)	0.63 (0.13)	19.31	20.35	19.27	20.51	-0.16 (0.87)	-0.19 (0.85)	0.00 (1.00)	-0.04 (6.95)	0.24 (4.85)	-0.24 (6.78)	4.86 (4.81)	3.61 (3.11)	5.37 (3.92)	0.95	0.97	0.94	0.95 (0.86-0.98)	0.97 (0.92-0.99)	0.94 (0.83-0.98)
Ventricular volume (ml)																							
Third ventricle	0.86 (0.38)	0.86 (0.39)	0.85 (0.39)	0.86 (0.39)	44.70	45.12	45.64	45.09	0.64 (0.53)	1.79 (0.09)	0.77 (0.45)	-0.54 (2.96)	-1.16 (2.54)	-0.39 (1.70)	2.24 (1.93)	2.12 (1.76)	1.23 (1.20)	1.00	1.00	1.00	1.00 (0.99-1.00)	1.00 (1.00-1.00)	1.00 (1.00-1.00)
Left lateral ventricle	8.45 (5.15)	8.45 (5.15)	8.46 (5.14)	8.44 (5.14)	60.95	61.00	60.72	60.90	-0.04 (0.97)	-1.64 (0.12)	0.37 (0.72)	-0.03 (1.04)	0.41 (1.17)	-0.03 (0.67)	0.61 (0.83)	0.64 (1.05)	0.52 (0.40)	1.00	1.00	1.00	1.00 (1.00-1.00)	1.00 (1.00-1.00)	1.00 (1.00-1.00)
Left inferior lateral ventricle	0.41 (0.23)	0.41 (0.21)	0.43 (0.21)	0.41 (0.23)	55.57	50.20	49.35	55.77	-0.11 (0.92)	-1.41 (0.18)	-0.03 (0.98)	1.01 (20.68)	5.76 (13.68)	-0.28 (13.42)	16.03 (12.43)	9.88 (10.88)	10.10 (8.45)	0.95	0.98	0.98	0.95 (0.85-0.98)	0.98 (0.94-0.99)	0.98 (0.94-0.99)
Right lateral ventricle	7.02 (3.69)	6.99 (3.68)	6.99 (3.70)	7.02 (3.68)	52.60	52.72	52.94	52.39	2.19 (0.04)	2.17 (0.05)	-0.02 (0.98)	-0.42 (0.92)	-0.37 (0.64)	0.04 (0.56)	0.74 (0.68)	0.57 (0.46)	0.39 (0.39)	1.00	1.00	1.00	1.00 (1.00-1.00)	1.00 (1.00-1.00)	1.00 (1.00-1.00)
Right inferior lateral ventricle	0.38 (0.18)	0.38 (0.19)	0.38 (0.18)	0.37 (0.19)	47.35	49.60	46.72	51.17	-0.16 (0.88)	0.73 (0.48)	0.92 (0.37)	-0.54 (6.82)	-0.41 (3.53)	-3.38 (12.60)	4.44 (5.08)	2.64 (2.28)	6.07 (11.48)	0.99	1.00	0.99	0.99 (0.98-1.00)	1.00 (0.99-1.00)	0.99 (0.97-1.00)
Local cortical volume (ml)																							
Left banks superior temporal sulcus	2.74 (0.53)	2.71 (0.50)	2.73 (0.54)	2.68 (0.49)	19.45	18.30	19.86	18.13	0.95 (0.36)	0.43 (0.67)	2.47 (0.03)	-0.80 (3.76)	-0.57 (4.00)	-2.05 (2.99)	3.30 (1.79)	3.01 (2.58)	3.05 (1.86)	0.98	0.97	0.99	0.98 (0.94-0.99)	0.97 (0.93-0.99)	0.98 (0.91-0.99)
Left caudal anterior cingulate cortex	1.96 (0.57)	1.94 (0.57)	1.95 (0.56)	1.95 (0.57)	28.91	29.45	28.44	28.95	1.50 (0.15)	0.12 (0.91)	0.25 (0.81)	-1.16 (2.85)	0.08 (3.43)	-0.16 (2.86)	2.15 (2.15)	2.87 (1.73)	2.34 (1.54)	1.00	0.99	1.00	1.00 (0.99-1.00)	0.99 (0.98-1.00)	1.00 (0.99-1.00)
Left caudal middle frontal gyrus	6.53 (0.89)	6.50 (0.87)	6.54 (0.90)	6.55 (0.89)	13.57	13.32	13.82	13.66	0.79 (0.44)	-0.29 (0.77)	-0.51 (0.62)	-0.45 (2.28)	0.09 (1.74)	0.30 (2.65)	1.72 (1.51)	1.30 (1.12)	2.05 (1.63)	0.98	0.99	0.98	0.98 (0.96-0.99)	0.99 (0.98-1.00)	0.98 (0.95-0.99)
Left cuneus	3.39 (0.63)	3.36 (0.60)	3.38 (0.62)	3.39 (0.64)	18.67	17.82	18.27	18.84	1.13 (0.28)	0.03 (0.97)	-0.24 (0.81)	-0.67 (2.63)	0.03 (2.71)	0.15 (2.85)	2.25 (1.42)	2.22 (1.46)	2.21 (1.70)	0.99	0.99	0.99	0.99 (0.97-1.00)	0.99 (0.97-1.00)	0.99 (0.96-1.00)
Left entorhinal cortex	2.03 (0.34)	2.00 (0.32)	2.04 (0.31)	2.04 (0.38)	16.96	16.11	14.99	18.80	0.81 (0.43)	-0.16 (0.87)	-0.28 (0.78)	-1.42 (7.83)	0.74 (12.09)	0.35 (9.55)	6.08 (4.91)	7.49 (9.32)	6.93 (6.34)	0.89	0.71	0.86	0.89 (0.72-0.96)	0.72 (0.36-0.89)	0.86 (0.64-0.95)
Left fusiform gyrus	10.75 (1.15)	10.69 (1.14)	10.66 (1.23)	10.70 (1.21)	10.74	10.69	11.50	11.27	1.06 (0.31)	1.61 (0.13)	1.44 (0.17)	-0.56 (2.16)	-0.86 (2.02)	-0.49 (1.17)	1.79 (1.26)	1.68 (1.36)	1.04 (0.70)	0.98	0.99	0.99	0.98 (0.95-0.99)	0.98 (0.95-0.99)	0.99 (0.98-1.00)
Left inferior parietal cortex	13.36 (2.06)	13.31 (2.09)	13.39 (2.18)	13.43 (2.07)	15.41	15.73	16.30	15.43	1.36 (0.19)	-0.33 (0.74)	-1.24 (0.23)	-0.45 (1.23)	0.02 (2.02)	0.48 (1.52)	1.07 (0.70)	1.47 (1.33)	1.25 (0.95)	1.00	0.99	0.99	1.00 (0.99-1.00)	0.99 (0.98-1.00)	0.99 (0.98-1.00)
Left inferior temporal gyrus	11.50 (1.31)	11.46 (1.42)	11.53 (1.46)	11.45 (1.41)	11.42	12.36	12.66	12.34	0.60 (0.55)	-0.52 (0.61)	0.61 (0.55)	-0.46 (2.40)	0.12 (2.19)	-0.55 (2.93)	1.73 (1.67)	1.76 (1.24)	2.42 (1.64)	0.99	0.99	0.97	0.98 (0.95-0.99)	0.99 (0.96-0.99)	0.97 (0.92-0.99)
Left isthmus cingulate cortex	2.91 (0.43)	2.93 (0.45)	2.94 (0.48)	2.94 (0.43)	14.86	15.51	16.28	14.67	-0.95 (0.36)	-1.53 (0.15)	-1.42 (0.18)	0.70 (3.67)	1.01 (2.89)	0.96 (2.62)	2.79 (2.39)	2.29 (1.96)	2.22 (1.61)	0.98	0.98	0.98	0.97 (0.93-0.99)	0.98 (0.94-0.99)	0.98 (0.95-0.99)
Left lateral occipital cortex	12.84 (2.13)	12.85 (2.06)	12.85 (2.17)	12.81 (2.08)	16.56	16.00	16.85	16.26	-0.08 (0.94)	-0.20 (0.84)	0.61 (0.55)	0.11 (2.10)	0.06 (1.91)	-0.21 (1.59)	1.55 (1.36)	1.58 (0.99)	1.16 (1.08)	0.99	0.99	1.00	0.99 (0.98-1.00)	0.99 (0.98-1.00)	1.00 (0.99-1.00)
Left lateral orbitofrontal cortex	8.01 (0.82)	8.04 (0.76)	8.02 (0.85)	8.04 (0.81)	10.23	9.45	10.60	10.04	-0.77 (0.45)	-0.26 (0.80)	-1.05 (0.31)	0.45 (2.02)	0.09 (2.16)	0.38 (1.44)	1.57 (1.29)	1.63 (1.36)	1.18 (0.86)	0.98	0.98	0.99	0.98 (0.95-0.99)	0.98 (0.95-0.99)	0.99 (0.97-1.00)
Left lingual gyrus	6.98 (1.22)	6.99 (1.22)	7.00 (1.24)	6.98 (1.24)	17.50	17.51	17.67	17.79	-0.26 (0.80)	-0.80 (0.43)	-0.27 (0.79)	0.07 (1.17)	0.26 (1.47)	0.03 (1.03)	0.92 (0.68)	1.12 (0.94)	0.87 (0.52)	1.00	1.00	1.00	1.00 (0.99-1.00)	1.00 (0.99-1.00)	1.00 (1.00-1.00)
Left medial orbitofrontal cortex	5.22 (0.86)	5.06 (0.62)	5.11 (0.79)	5.21 (0.78)	16.49	12.22	15.50	14.97	1.55 (0.14)	1.20 (0.25)	0.12 (0.91)	-2.65 (8.16)	-2.05 (7.35)	-0.06 (9.81)	7.41 (3.94)	6.22 (4.16)	8.34 (4.69)	0.88	0.90	0.82	0.82 (0.57-0.93)	0.89 (0.72-0.96)	0.82 (0.56-0.93)
Left middle temporal gyrus	11.22 (1.29)	11.25 (1.22)	11.24 (1.29)	11.20 (1.27)	11.52	10.81	11.46	11.32	-0.50 (0.62)	-0.39 (0.70)	0.35 (0.73)	0.43 (2.90)	0.22 (2.27)	-0.12 (1.79)	2.28 (1.75)	1.50 (1.68)	1.36 (1.12)	0.97	0.98	0.99	0.97 (0.92-0.99)	0.98 (0.95-0.99)	0.99 (0.97-1.00)

Left parahippocampal gyrus	2.39 (0.28)	2.38 (0.26)	2.39 (0.28)	2.40 (0.27)	11.72	11.07	11.79	11.05	0.37 (0.71)	0.11 (0.92)	-0.36 (0.72)	-0.34 (4.25)	-0.12 (4.55)	0.56 (5.23)	3.18 (2.72)	3.34 (2.96)	3.78 (3.52)	0.93	0.93	0.89	0.93 (0.82-0.98)	0.93 (0.82-0.98)	0.89 (0.72-0.96)
Left paracentral lobule	3.85 (0.60)	3.86 (0.57)	3.88 (0.59)	3.86 (0.55)	15.51	14.76	15.33	14.36	-0.40 (0.70)	-0.86 (0.40)	-0.16 (0.88)	0.33 (2.71)	0.58 (2.66)	0.19 (1.82)	2.11 (1.65)	1.91 (1.88)	1.44 (1.06)	0.99	0.98	0.99	0.99 (0.96-1.00)	0.98 (0.96-0.99)	0.99 (0.98-1.00)
Left pars opercularis	4.93 (0.73)	4.84 (0.71)	4.86 (0.78)	4.85 (0.70)	14.78	14.75	16.13	14.53	3.38 (0.00)	1.94 (0.07)	2.20 (0.04)	-1.87 (2.21)	-1.56 (2.85)	-1.62 (2.86)	2.48 (1.44)	2.57 (1.92)	2.41 (2.20)	0.99	0.99	0.98	0.98 (0.88-0.99)	0.98 (0.94-0.99)	0.97 (0.91-0.99)
Left pars orbitalis	2.53 (0.34)	2.54 (0.32)	2.54 (0.34)	2.53 (0.35)	13.49	12.58	13.38	13.81	-0.38 (0.71)	-0.36 (0.73)	-0.06 (0.95)	0.44 (3.61)	0.29 (3.18)	0.01 (4.10)	2.69 (2.35)	2.39 (2.04)	3.19 (2.45)	0.96	0.97	0.95	0.96 (0.89-0.99)	0.97 (0.92-0.99)	0.96 (0.88-0.98)
Left pars triangularis	3.89 (0.73)	3.91 (0.73)	3.89 (0.74)	3.93 (0.74)	18.87	18.65	19.04	18.90	-0.81 (0.43)	0.20 (0.84)	-1.70 (0.11)	0.60 (3.05)	-0.20 (2.99)	1.02 (2.52)	2.22 (2.11)	2.13 (2.04)	2.18 (1.54)	0.99	0.99	0.99	0.99 (0.97-1.00)	0.99 (0.97-1.00)	0.99 (0.97-1.00)
Left pericalcarine cortex	2.19 (0.47)	2.19 (0.48)	2.20 (0.46)	2.17 (0.47)	21.62	22.00	20.89	21.76	0.12 (0.91)	-0.19 (0.85)	0.83 (0.42)	-0.15 (4.03)	0.31 (4.26)	-0.94 (3.86)	3.21 (2.30)	3.34 (2.51)	3.01 (2.48)	0.98	0.98	0.98	0.98 (0.95-0.99)	0.98 (0.95-0.99)	0.98 (0.95-0.99)
Left postcentral gyrus	10.23 (1.45)	10.21 (1.44)	10.23 (1.46)	10.20 (1.43)	14.21	14.09	14.22	14.02	0.49 (0.63)	0.08 (0.94)	0.49 (0.63)	-0.26 (1.91)	-0.05 (1.36)	-0.29 (2.16)	1.31 (1.38)	1.02 (0.88)	1.59 (1.43)	0.99	0.99	0.99	0.99 (0.97-1.00)	0.99 (0.98-1.00)	0.99 (0.96-1.00)
Left posterior cingulate cortex	3.49 (0.60)	3.51 (0.57)	3.51 (0.60)	3.50 (0.61)	17.04	16.12	17.08	17.51	-1.28 (0.22)	-1.15 (0.27)	-0.39 (0.70)	0.69 (1.87)	0.42 (1.58)	0.19 (2.53)	1.42 (1.37)	1.24 (1.02)	1.99 (1.48)	1.00	1.00	0.99	1.00 (0.99-1.00)	1.00 (0.99-1.00)	0.99 (0.97-1.00)
Left precentral gyrus	13.69 (1.36)	13.69 (1.30)	13.72 (1.32)	13.73 (1.39)	9.96	9.46	9.63	10.11	-0.08 (0.93)	-0.52 (0.61)	-0.87 (0.40)	0.08 (1.75)	0.28 (1.94)	0.28 (1.29)	1.56 (0.69)	1.37 (1.37)	1.00 (0.82)	0.99	0.98	0.99	0.99 (0.96-0.99)	0.98 (0.95-0.99)	0.99 (0.98-1.00)
Left precuneus	10.50 (1.19)	10.41 (1.25)	10.49 (1.30)	10.48 (1.22)	11.31	11.96	12.38	11.61	2.04 (0.06)	0.21 (0.83)	0.60 (0.56)	-0.89 (1.68)	-0.19 (1.50)	-0.22 (1.35)	1.56 (1.03)	1.17 (0.90)	1.04 (0.85)	0.99	1.00	0.99	0.99 (0.96-1.00)	0.99 (0.98-1.00)	0.99 (0.98-1.00)
Left rostral anterior cingulate cortex	3.06 (0.50)	3.05 (0.52)	3.09 (0.51)	3.09 (0.51)	16.48	16.90	16.39	16.65	0.31 (0.76)	-1.00 (0.33)	-1.20 (0.25)	-0.30 (2.53)	0.94 (3.60)	0.77 (2.71)	1.81 (1.73)	2.87 (2.26)	2.26 (1.59)	0.99	0.97	0.99	0.99 (0.96-1.00)	0.97 (0.93-0.99)	0.99 (0.96-1.00)
Left rostral middle frontal gyrus	15.34 (2.11)	15.34 (2.04)	15.32 (2.10)	15.38 (2.00)	13.77	13.28	13.73	12.99	-0.07 (0.94)	0.32 (0.76)	-0.51 (0.62)	0.09 (1.97)	-0.14 (1.93)	0.35 (2.05)	1.60 (1.08)	1.52 (1.13)	1.68 (1.14)	0.99	0.99	0.99	0.99 (0.97-1.00)	0.99 (0.98-1.00)	0.99 (0.97-1.00)
Left superior frontal gyrus	23.51 (2.34)	23.55 (2.33)	23.37 (2.44)	23.57 (2.40)	9.94	9.89	10.44	10.20	-0.42 (0.68)	1.28 (0.22)	-0.57 (0.58)	0.17 (1.58)	-0.64 (1.91)	0.25 (1.98)	1.08 (1.13)	1.31 (1.50)	1.39 (1.39)	0.99	0.98	0.98	0.99 (0.97-1.00)	0.98 (0.95-0.99)	0.98 (0.95-0.99)
Left superior parietal cortex	14.57 (1.57)	14.56 (1.59)	14.58 (1.65)	14.58 (1.70)	10.74	10.94	11.34	11.63	0.15 (0.88)	-0.25 (0.81)	-0.16 (0.87)	-0.07 (1.34)	0.03 (1.50)	-0.01 (1.87)	0.93 (0.94)	1.08 (1.00)	1.50 (1.04)	0.99	0.99	0.99	0.99 (0.98-1.00)	0.99 (0.97-1.00)	0.99 (0.96-1.00)
Left superior temporal gyrus	12.23 (1.27)	12.28 (1.28)	12.27 (1.33)	12.31 (1.26)	10.35	10.39	10.84	10.24	-0.86 (0.40)	-0.71 (0.49)	-1.21 (0.25)	0.38 (1.81)	0.31 (2.03)	0.63 (2.12)	1.32 (1.25)	1.53 (1.32)	1.76 (1.27)	0.98	0.98	0.98	0.98 (0.96-0.99)	0.98 (0.95-0.99)	0.98 (0.94-0.99)
Left supramarginal gyrus	11.97 (1.60)	11.97 (1.58)	11.97 (1.66)	11.98 (1.69)	13.39	13.24	13.87	14.10	-0.02 (0.98)	-0.04 (0.97)	-0.21 (0.84)	0.03 (2.58)	-0.04 (2.19)	0.02 (1.97)	2.06 (1.45)	1.71 (1.30)	1.46 (1.27)	0.98	0.99	0.99	0.98 (0.94-0.99)	0.99 (0.96-1.00)	0.99 (0.97-1.00)
Left frontal pole	1.04 (0.14)	1.03 (0.14)	1.00 (0.11)	1.04 (0.15)	13.60	13.72	11.33	13.96	0.41 (0.69)	1.30 (0.21)	-0.44 (0.67)	-0.42 (4.36)	-3.55 (10.44)	0.60 (6.56)	2.67 (3.41)	7.14 (8.25)	5.12 (3.92)	0.95	0.59	0.90	0.96 (0.88-0.98)	0.57 (0.14-0.82)	0.90 (0.75-0.97)
Left temporal pole	2.63 (0.23)	2.66 (0.22)	2.65 (0.25)	2.65 (0.24)	8.66	8.29	9.61	9.25	-0.82 (0.43)	-0.53 (0.61)	-0.51 (0.62)	1.15 (5.83)	0.57 (5.02)	0.58 (4.92)	4.51 (3.71)	4.04 (2.86)	3.79 (3.03)	0.79	0.86	0.85	0.79 (0.51-0.92)	0.86 (0.64-0.95)	0.85 (0.63-0.95)
Left transverse temporal gyrus	1.33 (0.19)	1.33 (0.20)	1.33 (0.22)	1.33 (0.20)	14.70	15.39	16.64	14.76	-0.16 (0.88)	-0.69 (0.50)	-0.46 (0.65)	0.09 (4.11)	0.45 (3.91)	0.42 (3.76)	2.93 (2.78)	3.16 (2.21)	2.91 (2.29)	0.97	0.98	0.97	0.97 (0.91-0.99)	0.97 (0.92-0.99)	0.97 (0.92-0.99)
Left insula	7.80 (0.81)	7.89 (1.08)	7.99 (0.94)	7.87 (0.90)	10.40	13.73	11.80	11.40	-0.68 (0.50)	-1.88 (0.08)	-0.51 (0.62)	0.77 (6.27)	2.22 (4.78)	0.66 (6.01)	4.55 (4.24)	3.55 (3.83)	5.07 (3.03)	0.89	0.91	0.84	0.85 (0.64-0.95)	0.88 (0.68-0.96)	0.85 (0.62-0.94)
Right banks superior temporal sulcus	2.31 (0.32)	2.31 (0.34)	2.36 (0.35)	2.32 (0.40)	13.91	14.73	15.01	17.05	-0.12 (0.91)	-2.16 (0.05)	-0.47 (0.64)	0.01 (3.39)	1.81 (3.55)	0.14 (4.87)	2.98 (1.44)	3.30 (2.11)	3.70 (3.02)	0.97	0.97	0.97	0.97 (0.93-0.99)	0.96 (0.88-0.99)	0.95 (0.86-0.98)
Right caudal anterior cingulate cortex	2.45 (0.63)	2.45 (0.62)	2.42 (0.58)	2.45 (0.63)	25.86	25.43	23.93	25.54	-0.48 (0.64)	0.54 (0.59)	-0.28 (0.78)	0.46 (2.59)	-0.60 (5.98)	0.30 (3.68)	1.98 (1.66)	3.88 (4.48)	2.84 (2.26)	0.99	0.96	0.99	0.99 (0.98-1.00)	0.96 (0.89-0.99)	0.99 (0.97-1.00)
Right caudal middle frontal gyrus	6.49 (0.84)	6.50 (0.88)	6.49 (0.83)	6.45 (0.82)	12.89	13.51	12.75	12.65	-0.23 (0.82)	0.02 (0.98)	0.61 (0.55)	0.09 (2.98)	0.01 (2.73)	-0.60 (4.05)	2.36 (1.71)	2.18 (1.54)	2.84 (2.86)	0.98	0.98	0.95	0.98 (0.93-0.99)	0.98 (0.94-0.99)	0.95 (0.87-0.98)
Right cuneus	3.77 (0.74)	3.77 (0.76)	3.75 (0.69)	3.74 (0.72)	19.54	20.10	18.39	19.12	0.11 (0.91)	0.62 (0.55)	1.68 (0.11)	-0.18 (1.95)	-0.30 (2.89)	-0.75 (1.80)	1.66 (0.94)	2.39 (1.54)	1.57 (1.10)	1.00	0.99	1.00	0.99 (0.99-1.00)	0.99 (0.97-1.00)	0.99 (0.98-1.00)
Right entorhinal cortex	2.01 (0.33)	1.95 (0.46)	2.00 (0.38)	2.01 (0.41)	16.22	23.65	18.80	20.36	1.34 (0.20)	0.20 (0.84)	-0.06 (0.95)	-4.30 (9.16)	-0.76 (7.04)	-0.48 (8.92)	7.68 (6.37)	5.59 (4.10)	7.70 (4.07)	0.96	0.90	0.88	0.90 (0.74-0.96)	0.90 (0.74-0.96)	0.87 (0.66-0.95)
Right fusiform gyrus	9.91 (1.62)	9.86 (1.52)	9.94 (1.45)	9.94 (1.52)	16.39	15.46	14.56	15.34	0.80 (0.44)	-0.56 (0.58)	-0.57 (0.58)	-0.29 (2.24)	0.59 (2.47)	0.46 (2.36)	1.64 (1.50)	1.83 (1.70)	1.63 (1.72)	0.99	0.99	0.99	0.99 (0.98-1.00)	0.99 (0.97-1.00)	0.99 (0.97-1.00)
Right inferior parietal cortex	15.28 (2.33)	15.24 (2.36)	15.21 (2.39)	15.16 (2.39)	15.22	15.51	15.71	15.76	0.63 (0.54)	1.34 (0.20)	1.44 (0.17)	-0.28 (1.49)	-0.52 (1.41)	-0.86 (2.18)	1.22 (0.85)	1.15 (0.94)	1.73 (1.53)	1.00	1.00	0.99	1.00 (0.99-1.00)	1.00 (0.99-1.00)	0.99 (0.97-1.00)
Right inferior temporal gyrus	10.78 (1.13)	10.81 (1.14)	10.76 (1.11)	10.69 (1.13)	10.49	10.57	10.32	10.58	-0.66 (0.52)	0.36 (0.73)	1.42 (0.18)	0.30 (1.91)	-0.17 (2.12)	-0.78 (2.22)	1.51 (1.15)	1.69 (1.21)	1.67 (1.61)	0.98	0.98	0.98	0.98 (0.96-0.99)	0.98 (0.95-0.99)	0.98 (0.94-0.99)
Right isthmus cingulate cortex	2.84 (0.55)	2.85 (0.59)	2.86 (0.56)	2.86 (0.58)	19.35	20.73	19.55	20.09	-0.32 (0.76)	-1.05 (0.31)	-1.49 (0.16)	0.02 (2.61)	0.49 (2.28)	0.71 (2.09)	2.17 (1.33)	1.92 (1.23)	1.59 (1.49)	0.99	0.99	0.99	0.99 (0.98-1.00)	0.99 (0.98-1.00)	0.99 (0.98-1.00)
Right lateral occipital cortex	13.27 (2.52)	13.21 (2.47)	13.23 (2.55)	13.18 (2.49)	18.96	18.68	19.25	18.87	0.87 (0.40)	0.70 (0.49)	1.53 (0.15)	-0.42 (2.19)	-0.36 (1.65)	-0.65 (1.81)	1.82 (1.19)	1.21 (1.13)	1.64 (0.93)	0.99	1.00	1.00	0.99 (0.98-1.00)	1.00 (0.99-1.00)	1.00 (0.99-1.00)
Right lateral orbitofrontal cortex	8.06 (0.98)	8.22 (1.06)	8.01 (0.98)	8.09 (0.84)	12.20	12.89	12.23	10.36	-1.72 (0.11)	0.87 (0.40)	-0.47 (0.65)	1.88 (4.38)	-0.62 (2.78)	0.63 (3.67)	3.38 (3.28)	1.90 (2.07)	2.70 (2.47)	0.94	0.97	0.96	0.93 (0.80-0.97)	0.97 (0.93-0.99)	0.95 (0.85-0.98)
Right lingual gyrus	7.62 (1.12)	7.60 (1.13)	7.60 (1.15)	7.61 (1.10)	14.75	14.87	15.16	14.52	0.91 (0.38)	0.89 (0.39)	0.38 (0.71)	-0.26 (1.11)	-0.34 (1.25)	-0.10 (1.31)	0.83 (0.75)	1.03 (0.75)	1.08 (0.69)	1.00	1.00	1.00	1.00 (0.99-1.00)	1.00 (0.99-1.00)	1.00 (0.99-1.00)
Right medial orbitofrontal cortex	6.01 (0.74)	5.87 (0.75)	5.95 (0.73)	6.02 (0.78)	12.37	12.74	12.36	12.90	2.48 (0.03)	1.41 (0.18)	-0.23 (0.82)	-2.39 (3.84)	-1.06 (3.20)	0.10 (3.54)	3.24 (3.10)	2.48 (2.21)	2.77 (2.08)	0.95	0.97	0.97	0.94 (0.79-0.98)	0.97 (0.91-0.99)	0.97 (0.91-0.99)
Right middle temporal gyrus	12.16 (1.20)	12.16 (1.23)	12.04 (1.30)	12.01 (1.19)	9.85	10.11	10.77	9.94	0.10 (0.92)	2.40 (0.03)	1.89 (0.08)	-0.06 (1.28)	-1.16 (1.92)	-1.32 (2.68)	1.04 (0.71)	1.63 (1.51)	2.36 (1.76)	0.99	0.99	0.96	0.99 (0.98-1.00)	0.98 (0.93-0.99)	0.95 (0.87-0.98)
Right parahippocampal gyrus	2.20 (0.40)	2.22 (0.35)	2.23 (0.42)	2.24 (0.37)	18.36	15.66	18.65	16.39	-0.67 (0.51)	-1.89 (0.08)	-1.27 (0.22)	1.15 (4.33)	1.29 (3.10)	1.71 (4.11)	3.48 (2.69)	2.77 (1.78)	3.49 (2.65)	0.98	0.99	0.97	0.97 (0.91-0.99)	0.99 (0.96-1.00)	0.97 (0.91-0.99)
Right paracentral lobule	4.31 (0.57)	4.31 (0.62)	4.33 (0.57)	4.30 (0.55)	13.12	14.33	13.13	12.74	0.09 (0.93)	-0.69 (0.50)	0.24 (0.81)	-0.21 (2.40)	0.43 (2.39)	-0.08 (2.19)	1.74 (1.61)	2.02 (1.26)	1.65 (1.38)	0.99	0.98	0.99	0.98 (0.96-0.99)	0.98 (0.95-0.99)	0.99 (0.96-1.00)
Right pars opercularis	4.48 (0.54)	4.50 (0.51)	4.42 (0.53)	4.47 (0.49)	12.00	11.33	11.95	10.95	-0.45 (0.66)	3.41 (0.00)	0.24 (0.81)	0.39 (2.83)	-1.46 (1.84)	-0.11 (3.49)	2.49 (1.26)	2.02 (1.14)	2.71 (2.10)	0.97	0.99	0.96	0.97 (0.92-0.99)	0.98 (0.89-1.00)	0.96 (0.88-0.98)
Right pars orbitalis	3.00 (0.40)	3.01 (0.37)	3.00 (0.40)	2.99 (0.41)	13.16	12.28	13.23	13.85	-0.14 (0.89)	0.09 (0.93)	0.38 (0.71)	0.21 (3.28)	-0.06 (2.61)	-0.42 (3.48)	2.59 (1.92)	1.91 (1.71)	2.82 (1.95)	0.97	0.98	0.97	0.97 (0.91-0.99)	0.98 (0.94-0.99)	0.97 (0.91-0.99)
Right pars triangularis	4.72 (0.72)	4.71 (0.73)	4.72 (0.73)	4.73 (0.73)	15.20	15.57	15.39	15.51	0.10 (0.92)	-0.04 (0.97)	-0.48 (0.64)	-0.11 (2.26)	0.00 (1.81)	0.27 (2.51)	1.72 (1.39)	1.43 (1.04)	1.72 (1.79)	0.99	0.99	0.99	0.99 (0.97-1.00)	0.99 (0.98-1.00)	0.99 (0.97-1.00)
Right pericalcarine cortex	2.52 (0.54)	2.52 (0.52)	2.51 (0.56)	2.53 (0.53)	21.41	20.84	22.18	20.87	0.20 (0.84)	0.51 (0.62)	-0.83 (0.42)	-0.03 (2.60)	-0.46 (2.25)	0.62 (2.41)	2.06 (1.50)	1.80 (1.35)	2.05 (1.31)	0.99	0.99	0.99	0.99 (0.98-1.00)	0.99 (0.98-1.00)	0.99 (0.98-1.00)

Right postcentral gyrus	9.48 (0.82)	9.50 (0.82)	9.48 (0.85)	9.44 (0.80)	8.60	8.62	8.94	8.45	-0.50 (0.62)	0.04 (0.97)	1.01 (0.33)	0.18 (1.40)	-0.03 (1.37)	-0.39 (1.56)	1.21 (0.66)	1.14 (0.69)	1.13 (1.11)	0.99	0.99	0.98	0.99 (0.96-1.00)	0.99 (0.96-1.00)	0.98 (0.95-0.99)
Right posterior cingulate cortex	3.66 (0.58)	3.69 (0.59)	3.67 (0.59)	3.68 (0.58)	15.82	15.96	15.95	15.84	-1.14 (0.27)	-0.52 (0.61)	-0.92 (0.37)	0.67 (2.37)	0.29 (2.14)	0.46 (1.85)	1.91 (1.49)	1.77 (1.15)	1.49 (1.12)	0.99	0.99	0.99	0.99 (0.97-1.00)	0.99 (0.97-1.00)	0.99 (0.98-1.00)
Right precentral gyrus	14.04 (1.49)	14.07 (1.53)	14.03 (1.49)	14.02 (1.45)	10.57	10.89	10.59	10.31	-0.53 (0.60)	0.28 (0.78)	0.48 (0.64)	0.12 (1.27)	-0.12 (1.63)	-0.16 (1.39)	0.95 (0.81)	1.22 (1.04)	0.99 (0.95)	0.99	0.99	0.99	0.99 (0.98-1.00)	0.99 (0.96-1.00)	0.99 (0.97-1.00)
Right precuneus	10.80 (1.14)	10.71 (1.16)	10.76 (1.12)	10.70 (1.08)	10.52	10.84	10.44	10.08	1.81 (0.09)	1.22 (0.24)	2.34 (0.03)	-0.84 (1.83)	-0.31 (1.10)	-0.85 (1.53)	1.44 (1.38)	0.91 (0.66)	1.36 (1.06)	0.99	1.00	0.99	0.98 (0.95-0.99)	0.99 (0.99-1.00)	0.99 (0.95-1.00)
Right rostral anterior cingulate cortex	2.38 (0.45)	2.39 (0.50)	2.37 (0.44)	2.37 (0.47)	19.07	20.85	18.47	19.81	-0.57 (0.58)	0.49 (0.63)	0.80 (0.43)	0.29 (3.33)	-0.33 (3.76)	-0.59 (2.37)	2.51 (2.10)	2.86 (2.34)	1.86 (1.51)	0.98	0.98	0.99	0.98 (0.95-0.99)	0.98 (0.94-0.99)	0.99 (0.98-1.00)
Right rostral middle frontal gyrus	16.08 (2.07)	16.08 (1.98)	15.92 (2.10)	16.01 (2.01)	12.86	12.30	13.21	12.54	0.04 (0.97)	2.45 (0.03)	1.05 (0.31)	0.06 (1.65)	-1.06 (1.86)	-0.39 (1.67)	1.31 (0.95)	1.60 (1.40)	1.33 (1.03)	0.99	0.99	0.99	0.99 (0.98-1.00)	0.99 (0.96-1.00)	0.99 (0.98-1.00)
Right superior frontal gyrus	23.13 (2.42)	23.15 (2.42)	22.95 (2.36)	23.00 (2.39)	10.45	10.44	10.27	10.39	-0.20 (0.85)	1.57 (0.14)	1.33 (0.20)	0.08 (1.53)	-0.76 (1.93)	-0.58 (1.84)	1.11 (1.02)	1.54 (1.35)	1.43 (1.25)	0.99	0.98	0.99	0.99 (0.97-1.00)	0.98 (0.94-0.99)	0.98 (0.96-0.99)
Right superior parietal cortex	13.49 (1.59)	13.51 (1.57)	13.51 (1.48)	13.54 (1.51)	11.75	11.61	10.93	11.13	-0.20 (0.84)	-0.26 (0.80)	-0.71 (0.49)	0.12 (2.21)	0.20 (1.80)	0.42 (2.22)	1.69 (1.37)	1.33 (1.18)	1.52 (1.62)	0.98	0.99	0.99	0.99 (0.96-0.99)	0.99 (0.97-1.00)	0.98 (0.96-0.99)
Right superior temporal gyrus	12.33 (1.25)	12.38 (1.28)	12.29 (1.22)	12.35 (1.25)	10.16	10.37	9.92	10.14	-1.03 (0.32)	0.82 (0.42)	-0.50 (0.63)	0.37 (1.64)	-0.31 (1.72)	0.22 (1.83)	1.29 (1.03)	1.36 (1.06)	1.41 (1.12)	0.99	0.99	0.98	0.99 (0.97-1.00)	0.99 (0.97-1.00)	0.99 (0.96-0.99)
Right supramarginal gyrus	10.77 (1.58)	10.71 (1.57)	10.72 (1.61)	10.70 (1.54)	14.70	14.65	15.00	14.37	1.78 (0.09)	0.90 (0.38)	1.29 (0.22)	-0.51 (1.17)	-0.45 (1.92)	-0.56 (1.89)	1.05 (0.68)	1.56 (1.14)	1.63 (1.05)	1.00	0.99	0.99	1.00 (0.99-1.00)	0.99 (0.98-1.00)	0.99 (0.98-1.00)
Right frontal pole	1.24 (0.12)	1.27 (0.15)	1.18 (0.15)	1.23 (0.11)	9.52	12.12	13.05	9.14	-1.61 (0.13)	1.48 (0.16)	0.29 (0.78)	2.20 (6.13)	-4.91 (13.35)	-0.33 (5.00)	5.59 (3.07)	8.89 (10.93)	3.93 (2.94)	0.87	0.45	0.87	0.83 (0.58-0.94)	0.41 (-0.05-0.74)	0.87 (0.68-0.95)
Right temporal pole	2.67 (0.32)	2.66 (0.29)	2.62 (0.34)	2.64 (0.29)	12.17	10.92	12.84	10.83	0.02 (0.99)	1.36 (0.19)	0.47 (0.64)	0.15 (8.36)	-1.76 (5.33)	-0.71 (8.34)	5.63 (6.01)	4.03 (3.79)	5.65 (6.00)	0.80	0.92	0.80	0.80 (0.52-0.93)	0.91 (0.78-0.97)	0.80 (0.52-0.93)
Right transverse temporal gyrus	1.09 (0.20)	1.07 (0.19)	1.08 (0.19)	1.09 (0.21)	18.06	18.14	17.85	19.15	1.16 (0.27)	0.41 (0.69)	-0.20 (0.84)	-1.30 (4.37)	-0.27 (2.95)	0.00 (3.56)	3.51 (2.79)	2.32 (1.75)	2.62 (2.31)	0.97	0.98	0.98	0.97 (0.92-0.99)	0.99 (0.96-0.99)	0.98 (0.95-0.99)
Right insula	7.55 (0.91)	7.44 (0.82)	7.51 (0.96)	7.47 (0.92)	12.10	10.97	12.79	12.28	1.43 (0.17)	0.94 (0.36)	1.46 (0.16)	-1.27 (3.79)	-0.53 (1.95)	-1.11 (2.77)	2.85 (2.72)	1.55 (1.24)	2.42 (1.66)	0.95	0.99	0.97	0.94 (0.84-0.98)	0.99 (0.97-1.00)	0.97 (0.91-0.99)
Local cortical surface (cm^2)																							
Left banks superior temporal sulcus	10.73 (1.96)	10.64 (1.83)	10.67 (2.00)	10.52 (1.77)	18.24	17.22	18.75	16.81	1.02 (0.32)	0.64 (0.53)	2.21 (0.04)	-0.62 (2.89)	-0.64 (3.12)	-1.78 (3.20)	2.47 (1.50)	2.12 (2.32)	3.06 (1.91)	0.99	0.98	0.98	0.99 (0.96-0.99)	0.98 (0.95-0.99)	0.97 (0.91-0.99)
Left caudal anterior cingulate cortex	6.99 (1.84)	6.92 (1.81)	6.97 (1.82)	6.98 (1.74)	26.35	26.20	26.11	24.92	1.48 (0.16)	0.32 (0.75)	0.35 (0.73)	-0.98 (2.48)	-0.25 (3.46)	0.13 (2.45)	2.30 (1.24)	2.60 (2.21)	1.69 (1.72)	1.00	0.99	1.00	0.99 (0.99-1.00)	0.99 (0.98-1.00)	1.00 (0.99-1.00)
Left caudal middle frontal gyrus	24.06 (3.51)	23.97 (3.38)	24.11 (3.43)	24.20 (3.44)	14.60	14.10	14.22	14.22	1.15 (0.27)	-0.58 (0.57)	-1.36 (0.19)	-0.32 (1.28)	0.24 (1.21)	0.59 (1.57)	1.06 (0.74)	0.91 (0.81)	1.41 (0.84)	1.00	1.00	0.99	1.00 (0.99-1.00)	1.00 (0.99-1.00)	0.99 (0.98-1.00)
Left cuneus	16.57 (2.40)	16.51 (2.40)	16.54 (2.41)	16.65 (2.67)	14.49	14.56	14.59	16.05	0.59 (0.56)	0.30 (0.77)	-0.58 (0.57)	-0.36 (2.20)	-0.18 (2.31)	0.30 (2.94)	1.67 (1.41)	1.91 (1.22)	2.00 (2.11)	0.99	0.99	0.98	0.99 (0.96-1.00)	0.99 (0.96-1.00)	0.98 (0.93-0.99)
Left entorhinal cortex	4.47 (0.80)	4.46 (0.76)	4.47 (0.72)	4.52 (0.88)	17.98	17.06	15.98	19.52	0.13 (0.90)	-0.06 (0.96)	-0.72 (0.48)	-0.11 (8.48)	0.45 (9.38)	1.01 (6.73)	6.50 (5.18)	7.28 (5.62)	5.63 (3.54)	0.90	0.83	0.94	0.90 (0.74-0.96)	0.84 (0.59-0.94)	0.93 (0.83-0.98)
Left fusiform gyrus	33.50 (4.76)	33.40 (4.70)	33.37 (4.73)	33.49 (4.84)	14.20	14.09	14.17	14.44	0.72 (0.48)	1.11 (0.28)	0.13 (0.90)	-0.30 (1.88)	-0.40 (1.54)	-0.07 (1.29)	1.43 (1.19)	1.31 (0.84)	1.04 (0.72)	0.99	0.99	1.00	0.99 (0.98-1.00)	0.99 (0.99-1.00)	1.00 (0.99-1.00)
Left inferior parietal cortex	48.10 (7.38)	47.97 (7.59)	48.06 (7.65)	48.31 (7.33)	15.35	15.83	15.93	15.17	1.04 (0.31)	0.29 (0.78)	-1.44 (0.17)	-0.36 (1.12)	-0.20 (1.45)	0.43 (1.07)	0.88 (0.76)	1.07 (0.96)	0.81 (0.81)	1.00	1.00	1.00	1.00 (0.99-1.00)	1.00 (0.99-1.00)	1.00 (0.99-1.00)
Left inferior temporal gyrus	35.51 (5.18)	35.61 (5.42)	35.61 (5.23)	35.48 (5.27)	14.58	15.23	14.69	14.86	-0.50 (0.63)	-0.67 (0.52)	0.20 (0.85)	0.18 (2.06)	0.24 (1.65)	-0.11 (1.73)	1.54 (1.32)	1.25 (1.06)	1.49 (0.80)	0.99	0.99	0.99	0.99 (0.97-1.00)	0.99 (0.98-1.00)	0.99 (0.98-1.00)
Left isthmus cingulate cortex	10.67 (1.72)	10.73 (1.80)	10.73 (1.92)	10.73 (1.80)	16.10	16.78	17.87	16.73	-0.71 (0.49)	-0.83 (0.42)	-0.90 (0.38)	0.47 (3.28)	0.37 (2.68)	0.56 (2.82)	2.54 (2.02)	2.02 (1.72)	2.20 (1.76)	0.98	0.99	0.99	0.98 (0.95-0.99)	0.98 (0.96-0.99)	0.99 (0.96-0.99)
Left lateral occipital cortex	52.89 (9.37)	52.67 (9.05)	52.75 (9.41)	52.73 (9.25)	17.71	17.19	17.84	17.54	1.12 (0.28)	0.77 (0.46)	1.39 (0.18)	-0.35 (1.33)	-0.28 (1.47)	-0.28 (0.87)	1.05 (0.85)	1.13 (0.95)	0.72 (0.53)	1.00	1.00	1.00	1.00 (0.99-1.00)	1.00 (0.99-1.00)	1.00 (1.00-1.00)
Left lateral orbitofrontal cortex	27.74 (3.67)	27.89 (3.45)	27.74 (3.75)	27.94 (3.74)	13.23	12.39	13.52	13.40	-0.96 (0.35)	-0.01 (0.99)	-1.95 (0.07)	0.63 (2.21)	-0.04 (1.50)	0.69 (1.44)	1.78 (1.39)	1.13 (0.93)	1.25 (0.96)	0.99	0.99	0.99	0.99 (0.96-0.99)	0.99 (0.99-1.00)	0.99 (0.98-1.00)
Left lingual gyrus	32.10 (4.53)	32.28 (4.76)	32.32 (4.71)	32.37 (4.83)	14.10	14.74	14.59	14.92	-1.42 (0.17)	-1.36 (0.19)	-1.82 (0.09)	0.48 (1.38)	0.62 (1.86)	0.74 (1.79)	1.09 (0.94)	1.52 (1.18)	1.52 (1.15)	1.00	0.99	0.99	0.99 (0.98-1.00)	0.99 (0.97-1.00)	0.99 (0.97-1.00)
Left medial orbitofrontal cortex	19.04 (3.23)	18.59 (2.16)	18.81 (3.12)	19.20 (3.21)	16.98	11.62	16.60	16.71	1.02 (0.32)	0.61 (0.55)	-0.29 (0.77)	-1.69 (9.58)	-1.18 (7.69)	0.89 (11.62)	7.94 (5.24)	5.99 (4.73)	9.61 (6.10)	0.86	0.89	0.77	0.79 (0.51-0.92)	0.89 (0.72-0.96)	0.78 (0.48-0.92)
Left middle temporal gyrus	32.69 (3.38)	32.74 (3.21)	32.82 (3.32)	32.82 (3.53)	10.34	9.81	10.12	10.75	-0.36 (0.72)	-0.94 (0.36)	-1.18 (0.26)	0.22 (1.83)	0.42 (1.71)	0.36 (1.39)	1.33 (1.22)	1.43 (0.96)	1.00 (0.99)	0.98	0.99	0.99	0.98 (0.96-0.99)	0.99 (0.96-1.00)	0.99 (0.98-1.00)
Left parahippocampal gyrus	6.97 (1.16)	6.94 (1.19)	6.89 (1.27)	6.97 (1.20)	16.65	17.17	18.51	17.24	0.38 (0.71)	1.23 (0.24)	-0.08 (0.94)	-0.46 (4.29)	-1.41 (3.81)	0.01 (5.20)	3.22 (2.76)	3.49 (1.90)	4.15 (2.94)	0.97	0.98	0.95	0.97 (0.92-0.99)	0.98 (0.94-0.99)	0.95 (0.87-0.98)
Left paracentral lobule	14.06 (2.35)	14.11 (2.38)	14.07 (2.39)	14.02 (2.35)	16.74	16.87	17.00	16.73	-1.08 (0.30)	-0.28 (0.78)	0.71 (0.49)	0.38 (1.45)	0.06 (1.56)	-0.26 (1.57)	1.23 (0.80)	1.06 (1.11)	1.20 (1.01)	1.00	1.00	1.00	1.00 (0.99-1.00)	1.00 (0.99-1.00)	1.00 (0.99-1.00)
Left pars opercularis	17.21 (2.88)	16.96 (2.86)	16.98 (2.92)	16.97 (2.72)	16.76	16.83	17.18	16.00	2.08 (0.06)	2.06 (0.06)	1.99 (0.06)	-1.44 (2.65)	-1.36 (2.59)	-1.25 (2.65)	2.70 (1.20)	2.36 (1.66)	2.27 (1.77)	0.99	0.99	0.99	0.98 (0.95-0.99)	0.99 (0.96-1.00)	0.98 (0.95-0.99)
Left pars orbitalis	7.33 (1.17)	7.35 (1.14)	7.36 (1.18)	7.37 (1.27)	16.01	15.53	16.07	17.21	-0.25 (0.81)	-0.45 (0.66)	-0.67 (0.51)	0.26 (3.54)	0.32 (2.77)	0.38 (3.47)	2.74 (2.15)	2.13 (1.72)	2.90 (1.79)	0.97	0.98	0.98	0.97 (0.93-0.99)	0.98 (0.96-0.99)	0.98 (0.94-0.99)
Left pars triangularis	14.32 (3.12)	14.40 (3.09)	14.34 (3.21)	14.45 (3.08)	21.79	21.49	22.38	21.31	-1.19 (0.25)	-0.25 (0.80)	-1.48 (0.16)	0.62 (1.93)	0.02 (2.17)	1.00 (2.29)	1.48 (1.34)	1.63 (1.37)	2.01 (1.42)	1.00	1.00	0.99	1.00 (0.99-1.00)	1.00 (0.99-1.00)	0.99 (0.98-1.00)
Left pericalcarine cortex	15.15 (2.94)	15.22 (2.97)	15.21 (2.95)	15.21 (3.03)	19.42	19.54	19.42	19.91	-0.96 (0.35)	-0.69 (0.50)	-0.69 (0.50)	0.52 (1.99)	0.38 (2.25)	0.32 (2.14)	1.69 (1.10)	1.70 (1.46)	1.77 (1.17)	0.99	0.99	0.99	0.99 (0.98-1.00)	0.99 (0.98-1.00)	0.99 (0.98-1.00)
Left postcentral gyrus	42.60 (5.03)	42.74 (5.11)	42.66 (5.12)	42.72 (5.12)	11.82	11.95	12.01	11.99	-0.85 (0.41)	-0.64 (0.53)	-0.77 (0.46)	0.30 (1.47)	0.13 (0.94)	0.26 (1.38)	1.07 (1.02)	0.82 (0.44)	1.07 (0.87)	0.99	1.00	0.99	0.99 (0.98-1.00)	1.00 (0.99-1.00)	0.99 (0.98-1.00)
Left posterior cingulate cortex	12.75 (2.23)	12.75 (2.14)	12.76 (2.19)	12.80 (2.25)	17.47	16.83	17.16	17.55	0.09 (0.93)	-0.14 (0.89)	-0.70 (0.50)	0.07 (1.98)	0.11 (2.13)	0.40 (2.48)	1.56 (1.16)	1.60 (1.34)	2.02 (1.39)	0.99	0.99	0.99	0.99 (0.98-1.00)	0.99 (0.98-1.00)	0.99 (0.98-1.00)
Left precentral gyrus	48.01 (5.09)	48.19 (5.16)	48.06 (5.10)	48.06 (5.18)	10.60	10.70	10.61	10.77	-1.46 (0.17)	-0.44 (0.66)	-0.46 (0.66)	0.37 (1.05)	0.10 (0.95)	0.09 (0.85)	0.88 (0.65)	0.72 (0.60)	0.67 (0.51)	1.00	1.00	1.00	1.00 (0.99-1.00)	1.00 (0.99-1.00)	1.00 (0.99-1.00)
Left precuneus	39.54 (4.46)	39.34 (4.59)	39.41 (4.66)	39.51 (4.60)	11.27	11.67	11.82	11.64	1.80 (0.09)	1.12 (0.28)	0.26 (0.80)	-0.54 (1.12)	-0.38 (1.17)	-0.10 (1.08)	0.99 (0.73)	0.99 (0.70)	0.84 (0.66)	1.00	1.00	1.00	0.99 (0.98-1.00)	0.99 (0.99-1.00)	1.00 (0.99-1.00)
Left rostral anterior cingulate cortex	9.29 (1.87)	9.23 (1.87)	9.46 (1.78)	9.45 (1.85)	20.17	20.27	18.83	19.61	0.80 (0.43)	-1.49 (0.16)	-2.07 (0.06)	-0.65 (3.09)	2.11 (4.93)	1.81 (3.55)	2.51 (1.81)	4.51 (2.72)	2.99 (2.56)	0.99	0.97	0.99	0.99 (0.97-1.00)	0.96 (0.90-0.99)	0.98 (0.95-0.99)

Left rostral middle frontal gyrus	58.22 (9.29)	58.57 (9.29)	58.51 (9.11)	58.86 (9.36)	15.96	15.86	15.57	15.91	-1.41 (0.18)	-1.56 (0.14)	-2.77 (0.01)	0.60 (1.62)	0.55 (1.44)	1.09 (1.60)	1.47 (0.83)	0.96 (1.20)	1.55 (1.13)	0.99	1.00	1.00	0.99 (0.98-1.00)	1.00 (0.99-1.00)	0.99 (0.97-1.00)
Left superior frontal gyrus	77.07 (9.71)	77.19 (9.63)	76.90 (9.82)	77.26 (9.64)	12.60	12.48	12.77	12.47	-0.60 (0.56)	0.93 (0.37)	-1.16 (0.26)	0.16 (0.99)	-0.25 (0.93)	0.26 (0.82)	0.77 (0.61)	0.83 (0.44)	0.66 (0.52)	1.00	1.00	1.00	1.00 (0.99-1.00)	1.00 (0.99-1.00)	1.00 (0.99-1.00)
Left superior parietal cortex	58.92 (6.15)	58.80 (6.18)	58.96 (6.23)	58.90 (6.38)	10.44	10.52	10.56	10.84	1.39 (0.19)	-0.40 (0.69)	0.20 (0.85)	-0.23 (0.60)	0.06 (0.67)	-0.08 (0.75)	0.55 (0.31)	0.55 (0.37)	0.63 (0.38)	1.00	1.00	1.00	1.00 (0.99-1.00)	1.00 (0.99-1.00)	1.00 (0.99-1.00)
Left superior temporal gyrus	38.37 (4.67)	38.68 (4.80)	38.54 (4.71)	38.70 (4.79)	12.17	12.40	12.21	12.39	-2.97 (0.01)	-1.45 (0.17)	-2.23 (0.04)	0.78 (1.08)	0.43 (1.24)	0.82 (1.50)	1.04 (0.81)	0.92 (0.91)	1.27 (1.12)	1.00	1.00	0.99	0.99 (0.97-1.00)	0.99 (0.98-1.00)	0.99 (0.97-1.00)
Left supramarginal gyrus	42.86 (6.42)	42.73 (6.34)	42.62 (6.39)	42.75 (6.54)	14.98	14.83	15.00	15.30	0.75 (0.46)	1.34 (0.20)	0.75 (0.46)	-0.29 (1.51)	-0.55 (1.51)	-0.31 (1.34)	1.09 (1.04)	1.22 (1.01)	1.07 (0.83)	0.99	0.99	1.00	0.99 (0.98-1.00)	0.99 (0.98-1.00)	1.00 (0.99-1.00)
Left frontal pole	2.68 (0.20)	2.68 (0.26)	2.65 (0.22)	2.72 (0.25)	7.27	9.53	8.40	9.21	0.09 (0.93)	0.73 (0.48)	-1.09 (0.29)	-0.28 (5.08)	-1.22 (6.68)	1.25 (5.16)	3.98 (3.00)	4.95 (4.48)	4.31 (2.93)	0.86	0.68	0.84	0.84 (0.59-0.94)	0.68 (0.30-0.88)	0.81 (0.55-0.93)
Left temporal pole	4.84 (0.44)	4.90 (0.37)	4.83 (0.34)	4.90 (0.36)	9.06	7.61	7.09	7.40	-1.22 (0.24)	0.12 (0.91)	-1.00 (0.33)	1.54 (4.81)	0.01 (4.61)	1.44 (5.26)	3.81 (3.18)	3.05 (3.36)	4.24 (3.29)	0.85	0.86	0.81	0.84 (0.61-0.94)	0.84 (0.60-0.94)	0.80 (0.52-0.92)
Left transverse temporal gyrus	4.46 (0.59)	4.47 (0.59)	4.48 (0.67)	4.48 (0.60)	13.23	13.16	14.86	13.32	-0.19 (0.85)	-0.52 (0.61)	-0.68 (0.51)	0.15 (3.71)	0.25 (3.59)	0.47 (2.99)	2.51 (2.66)	2.75 (2.22)	2.27 (1.93)	0.96	0.97	0.98	0.97 (0.91-0.99)	0.97 (0.91-0.99)	0.98 (0.94-0.99)
Left insula	24.63 (3.08)	25.19 (4.27)	25.50 (3.38)	25.14 (3.10)	12.50	16.95	13.24	12.34	-0.87 (0.40)	-1.85 (0.08)	-0.84 (0.41)	1.65 (9.54)	3.35 (7.13)	1.99 (9.48)	6.81 (6.67)	5.75 (5.24)	8.05 (5.00)	0.80	0.83	0.70	0.76 (0.45-0.91)	0.81 (0.53-0.93)	0.70 (0.34-0.88)
Right banks superior temporal sulcus	9.18 (1.21)	9.23 (1.25)	9.34 (1.33)	9.28 (1.48)	13.13	13.57	14.26	15.95	-1.15 (0.27)	-2.20 (0.04)	-1.14 (0.27)	0.53 (2.01)	1.60 (3.24)	0.69 (3.41)	1.73 (1.07)	3.06 (1.80)	2.60 (2.23)	0.99	0.98	0.99	0.99 (0.97-1.00)	0.97 (0.89-0.99)	0.97 (0.91-0.99)
Right caudal anterior cingulate cortex	8.28 (2.09)	8.24 (2.01)	8.28 (2.01)	8.32 (2.13)	25.24	24.38	24.31	25.63	0.57 (0.58)	0.06 (0.95)	-0.72 (0.48)	-0.25 (3.33)	0.06 (4.70)	0.41 (2.99)	2.65 (1.92)	3.15 (3.39)	2.25 (1.93)	0.99	0.98	0.99	0.99 (0.98-1.00)	0.98 (0.94-0.99)	0.99 (0.98-1.00)
Right caudal middle frontal gyrus	23.13 (3.24)	23.11 (3.32)	23.14 (3.11)	23.06 (3.20)	14.03	14.38	13.42	13.89	0.19 (0.85)	-0.16 (0.87)	0.60 (0.56)	-0.14 (1.88)	0.16 (1.75)	-0.27 (2.01)	1.40 (1.21)	1.34 (1.08)	1.62 (1.15)	0.99	0.99	0.99	0.99 (0.98-1.00)	0.99 (0.98-1.00)	0.99 (0.98-1.00)
Right cuneus	17.63 (3.02)	17.72 (3.20)	17.47 (2.92)	17.47 (3.02)	17.12	18.06	16.72	17.27	-0.71 (0.49)	1.59 (0.13)	2.34 (0.03)	0.33 (2.63)	-0.86 (2.20)	-0.94 (1.56)	1.85 (1.84)	1.92 (1.30)	1.36 (1.19)	0.99	0.99	1.00	0.99 (0.97-1.00)	0.99 (0.97-1.00)	0.99 (0.98-1.00)
Right entorhinal cortex	4.12 (0.84)	4.03 (0.92)	4.07 (0.89)	4.08 (0.92)	20.40	22.84	21.93	22.51	1.21 (0.25)	0.92 (0.37)	0.52 (0.61)	-2.60 (6.91)	-1.40 (4.52)	-1.24 (6.57)	5.94 (4.16)	3.60 (2.95)	4.98 (4.29)	0.95	0.98	0.95	0.95 (0.86-0.98)	0.98 (0.93-0.99)	0.95 (0.87-0.98)
Right fusiform gyrus	31.00 (6.02)	30.94 (5.76)	30.98 (5.54)	31.11 (6.04)	19.43	18.61	17.89	19.42	0.59 (0.57)	0.14 (0.89)	-0.81 (0.43)	-0.09 (1.38)	0.13 (1.98)	0.33 (1.78)	1.13 (0.74)	1.61 (1.09)	1.28 (1.24)	1.00	1.00	1.00	1.00 (0.99-1.00)	0.99 (0.98-1.00)	1.00 (0.99-1.00)
Right inferior parietal cortex	55.65 (7.98)	55.67 (7.94)	55.56 (8.24)	55.35 (8.06)	14.34	14.26	14.84	14.56	-0.16 (0.87)	0.60 (0.56)	1.46 (0.17)	0.06 (1.08)	-0.22 (1.05)	-0.56 (1.48)	0.86 (0.62)	0.85 (0.61)	0.89 (1.29)	1.00	1.00	0.99	1.00 (0.99-1.00)	1.00 (0.99-1.00)	0.99 (0.98-1.00)
Right inferior temporal gyrus	33.69 (4.38)	33.78 (4.36)	33.80 (4.26)	33.54 (4.31)	13.00	12.90	12.62	12.86	-0.95 (0.36)	-0.88 (0.39)	1.16 (0.27)	0.27 (1.12)	0.39 (1.62)	-0.42 (1.55)	0.92 (0.66)	1.48 (0.67)	1.24 (0.97)	1.00	0.99	0.99	1.00 (0.99-1.00)	0.99 (0.98-1.00)	0.99 (0.98-1.00)
Right isthmus cingulate cortex	9.66 (1.80)	9.71 (2.08)	9.74 (1.96)	9.76 (2.06)	18.67	21.38	20.13	21.06	-0.58 (0.57)	-1.24 (0.23)	-1.26 (0.23)	0.23 (3.39)	0.61 (2.59)	0.77 (2.72)	2.65 (2.02)	2.17 (1.44)	2.03 (1.91)	0.99	0.99	0.99	0.98 (0.95-0.99)	0.99 (0.97-1.00)	0.98 (0.96-0.99)
Right lateral occipital cortex	52.98 (11.06)	52.79 (10.92)	52.87 (11.21)	52.80 (11.02)	20.87	20.68	21.21	20.88	1.07 (0.30)	0.67 (0.51)	0.98 (0.34)	-0.34 (1.38)	-0.29 (1.23)	-0.34 (1.33)	1.12 (0.85)	1.06 (0.62)	1.07 (0.83)	1.00	1.00	1.00	1.00 (0.99-1.00)	1.00 (1.00-1.00)	1.00 (0.99-1.00)
Right lateral orbitofrontal cortex	28.24 (3.86)	29.09 (4.30)	28.04 (4.01)	28.63 (3.27)	13.67	14.77	14.29	11.43	-2.09 (0.05)	0.60 (0.56)	-1.22 (0.24)	2.82 (5.59)	-0.82 (4.78)	1.61 (4.56)	4.95 (3.67)	3.55 (3.18)	3.39 (3.37)	0.93	0.94	0.95	0.90 (0.72-0.97)	0.94 (0.84-0.98)	0.94 (0.83-0.98)
Right lingual gyrus	34.07 (5.11)	34.03 (5.18)	34.05 (5.17)	34.04 (4.98)	14.98	15.21	15.20	14.64	0.43 (0.68)	0.30 (0.77)	0.23 (0.82)	-0.16 (1.29)	-0.10 (1.02)	-0.05 (1.78)	0.96 (0.84)	0.83 (0.56)	1.50 (0.88)	1.00	1.00	0.99	1.00 (0.99-1.00)	1.00 (0.99-1.00)	0.99 (0.98-1.00)
Right medial orbitofrontal cortex	20.64 (2.52)	20.24 (2.53)	20.62 (2.77)	20.86 (2.77)	12.21	12.50	13.44	13.27	1.96 (0.07)	0.10 (0.92)	-1.23 (0.24)	-1.97 (4.03)	-0.22 (3.99)	0.96 (3.38)	2.94 (3.34)	2.67 (2.89)	2.69 (2.16)	0.95	0.95	0.97	0.94 (0.82-0.98)	0.95 (0.86-0.98)	0.96 (0.89-0.99)
Right middle temporal gyrus	35.50 (3.93)	35.52 (3.94)	35.35 (4.10)	35.50 (3.97)	11.06	11.09	11.59	11.18	-0.11 (0.92)	1.48 (0.16)	0.00 (1.00)	0.03 (1.09)	-0.48 (1.17)	-0.01 (1.33)	0.80 (0.71)	0.99 (0.76)	1.02 (0.80)	0.99	1.00	0.99	1.00 (0.99-1.00)	0.99 (0.98-1.00)	0.99 (0.98-1.00)
Right parahippocampal gyrus	6.29 (1.22)	6.34 (1.05)	6.37 (1.26)	6.38 (1.15)	19.33	16.64	19.74	18.06	-0.77 (0.45)	-1.75 (0.10)	-1.56 (0.14)	1.22 (3.99)	1.12 (2.85)	1.64 (3.91)	3.26 (2.48)	2.48 (1.69)	2.95 (2.98)	0.98	0.99	0.98	0.97 (0.93-0.99)	0.99 (0.97-1.00)	0.98 (0.94-0.99)
Right paracentral lobule	15.83 (2.08)	15.88 (2.23)	15.84 (2.10)	15.84 (2.05)	13.13	14.03	13.23	12.96	-0.62 (0.55)	-0.14 (0.89)	-0.17 (0.87)	0.18 (1.74)	0.05 (1.52)	0.08 (1.31)	1.35 (1.05)	1.19 (0.88)	1.09 (0.67)	0.99	0.99	1.00	0.99 (0.98-1.00)	0.99 (0.98-1.00)	1.00 (0.99-1.00)
Right pars opercularis	15.47 (2.01)	15.51 (1.90)	15.31 (1.95)	15.52 (2.00)	13.02	12.26	12.76	12.89	-0.32 (0.76)	2.49 (0.02)	-0.44 (0.67)	0.34 (3.08)	-1.03 (1.73)	0.33 (2.89)	2.61 (1.54)	1.68 (1.06)	2.38 (1.57)	0.97	0.99	0.97	0.97 (0.92-0.99)	0.99 (0.95-1.00)	0.97 (0.93-0.99)
Right pars orbitalis	8.90 (1.43)	8.90 (1.43)	8.90 (1.54)	8.90 (1.52)	16.01	16.12	17.27	17.10	0.06 (0.95)	0.06 (0.95)	0.04 (0.97)	-0.05 (2.41)	-0.21 (2.37)	-0.17 (2.69)	1.69 (1.67)	2.02 (1.15)	2.22 (1.42)	0.99	0.99	0.99	0.99 (0.97-1.00)	0.99 (0.97-1.00)	0.99 (0.96-1.00)
Right pars triangularis	16.86 (2.91)	16.89 (3.11)	17.00 (3.05)	16.97 (2.98)	17.23	18.41	17.92	17.59	-0.28 (0.78)	-1.75 (0.10)	-1.59 (0.13)	-0.02 (2.39)	0.73 (1.88)	0.57 (1.58)	1.84 (1.45)	1.34 (1.48)	1.28 (1.06)	0.99	0.99	1.00	0.99 (0.98-1.00)	0.99 (0.98-1.00)	1.00 (0.99-1.00)
Right pericalcarine cortex	17.05 (3.07)	17.10 (3.14)	16.85 (3.09)	16.95 (3.02)	17.99	18.39	18.36	17.81	-0.52 (0.61)	3.00 (0.01)	1.22 (0.24)	0.17 (2.06)	-1.29 (1.87)	-0.59 (2.08)	1.57 (1.29)	1.64 (1.55)	1.69 (1.28)	0.99	1.00	0.99	0.99 (0.98-1.00)	0.99 (0.97-1.00)	0.99 (0.98-1.00)
Right postcentral gyrus	40.58 (3.23)	40.74 (3.08)	40.70 (3.24)	40.57 (3.24)	7.97	7.57	7.97	7.98	-1.36 (0.19)	-1.31 (0.21)	0.11 (0.92)	0.43 (1.20)	0.32 (0.99)	-0.02 (0.76)	0.92 (0.85)	0.88 (0.52)	0.61 (0.43)	0.99	0.99	1.00	0.99 (0.97-1.00)	0.99 (0.98-1.00)	1.00 (0.99-1.00)
Right posterior cingulate cortex	13.17 (1.94)	13.20 (2.04)	13.11 (1.98)	13.22 (2.04)	14.77	15.49	15.09	15.42	-0.33 (0.74)	0.78 (0.45)	-0.95 (0.36)	0.14 (2.57)	-0.43 (2.10)	0.30 (1.56)	2.10 (1.40)	1.60 (1.37)	1.30 (0.84)	0.98	0.99	1.00	0.98 (0.95-0.99)	0.99 (0.97-1.00)	0.99 (0.98-1.00)
Right precentral gyrus	49.48 (5.68)	49.54 (5.90)	49.60 (5.89)	49.72 (5.70)	11.48	11.90	11.87	11.47	-0.49 (0.63)	-1.00 (0.34)	-1.54 (0.14)	0.08 (1.14)	0.20 (1.00)	0.48 (1.23)	0.88 (0.69)	0.83 (0.56)	1.02 (0.80)	1.00	1.00	0.99	1.00 (0.99-1.00)	1.00 (0.99-1.00)	0.99 (0.98-1.00)
Right precuneus	41.22 (5.10)	41.08 (4.98)	41.16 (4.90)	41.01 (4.98)	12.36	12.13	11.92	12.14	1.20 (0.25)	0.49 (0.63)	1.65 (0.12)	-0.32 (1.10)	-0.10 (1.17)	-0.48 (1.15)	0.77 (0.83)	0.94 (0.65)	0.93 (0.81)	1.00	1.00	1.00	1.00 (0.99-1.00)	1.00 (0.99-1.00)	0.99 (0.98-1.00)
Right rostral anterior cingulate cortex	6.90 (1.47)	6.94 (1.58)	6.84 (1.45)	6.91 (1.52)	21.35	22.80	21.25	22.01	-0.57 (0.58)	1.10 (0.29)	-0.06 (0.95)	0.27 (3.77)	-0.88 (3.68)	-0.08 (2.94)	2.81 (2.43)	2.69 (2.58)	2.19 (1.88)	0.98	0.99	0.99	0.98 (0.95-0.99)	0.99 (0.97-1.00)	0.99 (0.98-1.00)
Right rostral middle frontal gyrus	59.95 (8.74)	59.99 (8.53)	59.79 (8.79)	60.16 (8.69)	14.59	14.22	14.71	14.45	-0.32 (0.76)	1.27 (0.22)	-1.47 (0.16)	0.11 (0.83)	-0.28 (0.92)	0.37 (1.06)	0.64 (0.51)	0.74 (0.58)	0.88 (0.67)	1.00	1.00	1.00	1.00 (1.00-1.00)	1.00 (1.00-1.00)	1.00 (0.99-1.00)
Right superior frontal gyrus	74.04 (8.84)	74.03 (8.69)	73.80 (8.93)	74.00 (8.76)	11.95	11.74	12.10	11.84	0.03 (0.98)	1.40 (0.18)	0.36 (0.73)	0.02 (0.79)	-0.34 (0.87)	-0.04 (0.62)	0.65 (0.43)	0.77 (0.49)	0.52 (0.31)	1.00	1.00	1.00	1.00 (0.99-1.00)	1.00 (0.99-1.00)	1.00 (1.00-1.00)
Right superior parietal cortex	54.85 (5.56)	54.99 (5.39)	55.09 (5.21)	55.34 (5.44)	10.13	9.80	9.46	9.83	-0.98 (0.34)	-1.21 (0.25)	-2.95 (0.01)	0.28 (1.04)	0.50 (1.48)	0.92 (1.25)	0.76 (0.74)	0.96 (1.21)	0.96 (1.21)	1.00	0.99	0.99	0.99 (0.99-1.00)	0.99 (0.97-1.00)	0.99 (0.95-1.00)
Right superior temporal gyrus	37.23 (4.42)	37.42 (4.35)	37.19 (4.19)	37.43 (4.34)	11.88	11.62	11.27	11.59	-1.55 (0.14)	0.34 (0.74)	-2.40 (0.03)	0.53 (1.31)	-0.04 (1.30)	0.56 (0.90)	1.18 (0.72)	1.07 (0.67)	0.82 (0.66)	0.99	1.00	1.00	0.99 (0.98-1.00)	0.99 (0.98-1.00)	1.00 (0.99-1.00)
Right supramarginal gyrus	38.99 (6.10)	38.90 (5.93)	38.90 (5.97)	38.94 (6.04)	15.64	15.24	15.34	15.51	0.71 (0.49)	0.95 (0.36)	0.69 (0.50)	-0.18 (1.27)	-0.20 (1.08)	-0.12 (0.84)	1.04 (0.72)	0.85 (0.66)	0.66 (0.50)	1.00	1.00	1.00	1.00 (0.99-1.00)	1.00 (0.99-1.00)	1.00 (1.00-1.00)
Right frontal pole	3.34 (0.33)	3.41 (0.42)	3.32 (0.37)	3.35 (0.39)	9.80	12.24	11.25	11.62	-1.70 (0.11)	0.33 (0.75)	-0.18 (0.86)	1.96 (4.95)	-0.60 (5.89)	0.09 (5.79)	4.30 (2.97)	4.41 (3.78)	4.65 (3.24)	0.92	0.86	0.86	0.88 (0.69-0.96)	0.86 (0.64-0.95)	0.86 (0.64-0.95)

Right temporal pole	4.70 (0.44)	4.69 (0.54)	4.58 (0.47)	4.64 (0.55)	9.30	11.56	10.15	11.85	0.14 (0.89)	2.76 (0.01)	0.61 (0.55)	-0.48 (7.15)	-2.55 (3.58)	-1.47 (8.02)	5.35 (4.57)	3.28 (2.87)	5.94 (5.38)	0.81	0.93	0.74	0.80 (0.51-0.93)	0.91 (0.66-0.97)	0.73 (0.38-0.90)
Right transverse temporal gyrus	3.35 (0.45)	3.33 (0.51)	3.34 (0.41)	3.35 (0.45)	13.37	15.36	12.39	13.49	0.51 (0.62)	0.30 (0.77)	-0.08 (0.94)	-0.92 (5.26)	-0.15 (3.03)	0.04 (3.99)	4.27 (3.03)	2.17 (2.05)	3.15 (2.31)	0.94	0.97	0.96	0.94 (0.83-0.98)	0.97 (0.92-0.99)	0.96 (0.89-0.99)
Right insula	23.57 (3.22)	22.86 (2.71)	23.64 (3.56)	23.15 (2.99)	13.67	11.86	15.08	12.91	1.83 (0.09)	-0.46 (0.65)	1.24 (0.24)	-2.82 (6.17)	0.11 (2.07)	-1.71 (5.42)	4.86 (4.63)	1.45 (1.43)	3.47 (4.43)	0.88	0.99	0.91	0.85 (0.61-0.94)	0.99 (0.96-1.00)	0.90 (0.75-0.96)
Local cortical thickness (mm)																							
Left banks superior temporal sulcus	2.61 (0.14)	2.61 (0.14)	2.62 (0.14)	2.62 (0.14)	5.49	5.41	5.51	5.48	0.40 (0.69)	-0.32 (0.75)	-1.30 (0.21)	-0.11 (1.18)	0.14 (1.68)	0.49 (1.51)	0.99 (0.60)	1.06 (1.27)	1.27 (0.89)	0.98	0.95	0.96	0.98 (0.94-0.99)	0.95 (0.87-0.98)	0.96 (0.89-0.99)
Left caudal anterior cingulate cortex	2.56 (0.16)	2.56 (0.19)	2.57 (0.20)	2.55 (0.20)	6.13	7.46	7.77	7.88	-0.21 (0.83)	-0.49 (0.63)	0.30 (0.77)	0.06 (2.68)	0.33 (3.47)	-0.39 (3.66)	2.03 (1.68)	2.35 (2.50)	2.59 (2.53)	0.93	0.89	0.90	0.92 (0.79-0.97)	0.87 (0.68-0.95)	0.88 (0.68-0.95)
Left caudal middle frontal gyrus	2.43 (0.09)	2.43 (0.10)	2.43 (0.10)	2.42 (0.09)	3.66	4.23	4.32	3.78	-0.04 (0.97)	-0.37 (0.71)	0.22 (0.83)	-0.01 (1.43)	0.16 (1.94)	-0.09 (1.51)	1.06 (0.93)	1.30 (1.41)	1.13 (0.96)	0.95	0.89	0.92	0.94 (0.84-0.98)	0.89 (0.70-0.96)	0.92 (0.78-0.97)
Left cuneus	1.97 (0.11)	1.96 (0.10)	1.96 (0.11)	1.96 (0.11)	5.83	5.22	5.67	5.61	0.66 (0.52)	0.32 (0.75)	1.33 (0.20)	-0.31 (2.03)	-0.13 (1.78)	-0.51 (1.53)	1.47 (1.37)	1.41 (1.04)	1.21 (1.03)	0.94	0.95	0.96	0.93 (0.83-0.98)	0.96 (0.88-0.98)	0.96 (0.90-0.99)
Left entorhinal cortex	3.47 (0.34)	3.45 (0.34)	3.47 (0.35)	3.48 (0.36)	9.80	9.91	10.04	10.23	0.49 (0.63)	-0.11 (0.91)	-0.42 (0.68)	-0.38 (3.14)	0.08 (2.92)	0.38 (3.96)	2.53 (1.79)	2.16 (1.89)	2.79 (2.74)	0.95	0.96	0.92	0.95 (0.87-0.98)	0.96 (0.89-0.99)	0.93 (0.80-0.97)
Left fusiform gyrus	2.84 (0.12)	2.84 (0.12)	2.84 (0.12)	2.84 (0.12)	4.34	4.40	4.13	4.13	-0.04 (0.97)	0.06 (0.96)	0.15 (0.88)	0.01 (0.98)	-0.01 (1.09)	-0.03 (1.05)	0.71 (0.65)	0.90 (0.56)	0.86 (0.55)	0.98	0.97	0.97	0.98 (0.94-0.99)	0.97 (0.91-0.99)	0.97 (0.92-0.99)
Left inferior parietal cortex	2.53 (0.08)	2.52 (0.08)	2.53 (0.07)	2.53 (0.08)	2.98	3.12	2.90	2.97	0.46 (0.65)	-0.53 (0.60)	0.12 (0.91)	-0.13 (1.13)	0.15 (1.11)	-0.04 (1.44)	0.83 (0.75)	0.77 (0.79)	1.05 (0.95)	0.93	0.93	0.88	0.93 (0.83-0.98)	0.93 (0.82-0.98)	0.89 (0.71-0.96)
Left inferior temporal gyrus	2.74 (0.13)	2.73 (0.14)	2.73 (0.12)	2.73 (0.14)	4.69	5.26	4.43	5.21	1.11 (0.28)	0.25 (0.80)	0.75 (0.46)	-0.40 (1.34)	-0.09 (1.76)	-0.34 (1.65)	1.15 (0.75)	1.41 (1.01)	1.28 (1.05)	0.97	0.93	0.94	0.96 (0.90-0.99)	0.93 (0.82-0.98)	0.94 (0.84-0.98)
Left isthmus cingulate cortex	2.53 (0.21)	2.51 (0.20)	2.55 (0.22)	2.52 (0.19)	8.40	8.07	8.53	7.71	0.74 (0.47)	-1.58 (0.13)	0.94 (0.36)	-0.57 (3.00)	0.72 (1.81)	-0.40 (1.75)	2.33 (1.88)	1.37 (1.36)	1.39 (1.09)	0.93	0.98	0.98	0.93 (0.81-0.97)	0.97 (0.93-0.99)	0.97 (0.93-0.99)
Left lateral occipital cortex	2.25 (0.08)	2.26 (0.08)	2.25 (0.08)	2.25 (0.08)	3.39	3.64	3.56	3.73	-0.98 (0.34)	-0.86 (0.40)	-0.72 (0.48)	0.30 (1.27)	0.18 (0.87)	0.18 (1.08)	0.88 (0.94)	0.72 (0.48)	0.82 (0.70)	0.94	0.97	0.96	0.93 (0.83-0.98)	0.97 (0.91-0.99)	0.96 (0.88-0.98)
Left lateral orbitofrontal cortex	2.60 (0.12)	2.59 (0.11)	2.60 (0.10)	2.60 (0.12)	4.44	4.42	3.88	4.49	0.67 (0.51)	0.07 (0.95)	0.18 (0.86)	-0.22 (1.31)	-0.01 (1.86)	-0.09 (1.95)	1.06 (0.76)	1.38 (1.18)	1.47 (1.23)	0.96	0.91	0.90	0.96 (0.88-0.98)	0.91 (0.76-0.97)	0.91 (0.75-0.97)
Left lingual gyrus	2.07 (0.12)	2.07 (0.12)	2.07 (0.12)	2.06 (0.11)	5.70	5.67	6.04	5.44	0.86 (0.40)	0.46 (0.65)	1.67 (0.12)	-0.28 (1.33)	-0.29 (2.33)	-0.65 (1.59)	1.04 (0.84)	1.96 (1.20)	1.45 (0.86)	0.97	0.92	0.96	0.97 (0.93-0.99)	0.93 (0.80-0.97)	0.95 (0.87-0.98)
Left medial orbitofrontal cortex	2.39 (0.10)	2.37 (0.11)	2.38 (0.08)	2.38 (0.09)	4.05	4.75	3.45	3.79	1.80 (0.09)	0.62 (0.54)	0.69 (0.50)	-1.17 (2.65)	-0.46 (3.17)	-0.37 (2.27)	1.95 (2.11)	2.47 (1.93)	1.69 (1.50)	0.84	0.67	0.84	0.81 (0.54-0.93)	0.67 (0.27-0.87)	0.84 (0.61-0.94)
Left middle temporal gyrus	2.80 (0.11)	2.80 (0.11)	2.80 (0.10)	2.80 (0.10)	4.02	4.10	3.53	3.65	-0.40 (0.69)	-0.17 (0.87)	-0.18 (0.86)	0.12 (1.30)	0.08 (1.59)	0.09 (1.81)	1.01 (0.79)	1.20 (0.99)	1.23 (1.29)	0.95	0.92	0.90	0.95 (0.87-0.98)	0.92 (0.78-0.97)	0.90 (0.73-0.96)
Left parahippocampal gyrus	3.03 (0.24)	3.02 (0.23)	3.05 (0.25)	3.04 (0.24)	7.97	7.49	8.21	7.87	0.83 (0.42)	-1.20 (0.25)	-0.24 (0.81)	-0.31 (1.62)	0.51 (1.66)	0.11 (1.74)	1.23 (1.06)	1.34 (1.06)	1.34 (1.07)	0.98	0.98	0.98	0.98 (0.94-0.99)	0.98 (0.93-0.99)	0.98 (0.94-0.99)
Left paracentral lobule	2.50 (0.10)	2.50 (0.12)	2.51 (0.12)	2.51 (0.12)	4.11	4.84	4.95	4.67	0.21 (0.84)	-0.62 (0.54)	-0.84 (0.42)	-0.13 (1.96)	0.24 (1.76)	0.37 (1.88)	1.35 (1.38)	1.42 (1.01)	1.64 (0.90)	0.92	0.94	0.92	0.91 (0.76-0.97)	0.93 (0.81-0.97)	0.91 (0.77-0.97)
Left pars opercularis	2.51 (0.11)	2.50 (0.11)	2.51 (0.11)	2.51 (0.10)	4.39	4.49	4.46	4.00	1.06 (0.30)	0.02 (0.98)	-0.07 (0.95)	-0.35 (1.30)	-0.01 (1.48)	0.06 (2.33)	1.15 (0.65)	1.20 (0.81)	1.52 (1.72)	0.96	0.94	0.86	0.96 (0.88-0.98)	0.95 (0.86-0.98)	0.86 (0.65-0.95)
Left pars orbitalis	2.62 (0.14)	2.62 (0.14)	2.61 (0.13)	2.61 (0.13)	5.34	5.51	4.95	5.15	-0.21 (0.84)	0.76 (0.46)	0.51 (0.62)	0.11 (2.20)	-0.36 (2.01)	-0.24 (1.95)	1.54 (1.53)	1.67 (1.08)	1.62 (1.03)	0.91	0.93	0.93	0.92 (0.78-0.97)	0.93 (0.80-0.97)	0.93 (0.82-0.98)
Left pars triangularis	2.39 (0.13)	2.39 (0.14)	2.39 (0.13)	2.40 (0.13)	5.44	5.90	5.30	5.45	0.48 (0.64)	0.42 (0.68)	-0.49 (0.63)	-0.23 (1.70)	-0.17 (1.76)	0.18 (1.45)	1.27 (1.11)	1.19 (1.27)	0.94 (1.10)	0.96	0.95	0.97	0.96 (0.88-0.98)	0.95 (0.87-0.98)	0.97 (0.91-0.99)
Left pericalcarine cortex	1.64 (0.12)	1.64 (0.12)	1.65 (0.12)	1.63 (0.14)	7.05	7.49	7.26	8.37	0.24 (0.81)	-0.47 (0.65)	1.05 (0.31)	-0.18 (2.41)	0.36 (3.10)	-0.99 (3.27)	1.97 (1.30)	2.59 (1.61)	2.66 (2.04)	0.95	0.91	0.91	0.95 (0.86-0.98)	0.91 (0.76-0.97)	0.90 (0.75-0.96)
Left postcentral gyrus	2.12 (0.09)	2.11 (0.09)	2.12 (0.09)	2.11 (0.08)	4.34	4.04	4.22	3.90	1.36 (0.19)	-0.15 (0.88)	0.80 (0.44)	-0.44 (1.31)	0.05 (1.35)	-0.26 (1.36)	1.13 (0.73)	1.02 (0.84)	1.19 (0.65)	0.95	0.95	0.95	0.95 (0.86-0.98)	0.95 (0.87-0.98)	0.95 (0.86-0.98)
Left posterior cingulate cortex	2.52 (0.11)	2.53 (0.11)	2.53 (0.11)	2.52 (0.11)	4.37	4.49	4.37	4.44	-1.18 (0.26)	-2.09 (0.05)	-0.58 (0.57)	0.38 (1.30)	0.59 (1.12)	0.20 (1.39)	1.10 (0.73)	0.97 (0.79)	1.08 (0.85)	0.96	0.97	0.95	0.96 (0.88-0.98)	0.96 (0.87-0.99)	0.95 (0.87-0.98)
Left precentral gyrus	2.58 (0.11)	2.58 (0.10)	2.59 (0.09)	2.58 (0.09)	4.08	3.82	3.33	3.52	0.65 (0.53)	-0.67 (0.51)	-0.25 (0.81)	-0.17 (1.12)	0.28 (1.58)	0.10 (1.27)	0.91 (0.63)	1.03 (1.19)	1.06 (0.66)	0.96	0.93	0.96	0.96 (0.90-0.99)	0.92 (0.78-0.97)	0.95 (0.86-0.98)
Left precuneus	2.51 (0.07)	2.51 (0.07)	2.52 (0.08)	2.51 (0.07)	2.96	2.76	3.16	2.93	0.26 (0.80)	-1.34 (0.20)	-0.55 (0.59)	-0.06 (0.94)	0.33 (0.98)	0.14 (1.03)	0.71 (0.59)	0.70 (0.74)	0.84 (0.58)	0.95	0.95	0.94	0.95 (0.86-0.98)	0.94 (0.85-0.98)	0.94 (0.85-0.98)
Left rostral anterior cingulate cortex	2.88 (0.25)	2.89 (0.25)	2.87 (0.23)	2.86 (0.22)	8.65	8.71	8.11	7.61	-0.56 (0.58)	0.40 (0.69)	0.88 (0.40)	0.38 (2.91)	-0.31 (3.49)	-0.45 (2.29)	2.35 (1.64)	2.88 (1.86)	1.56 (1.68)	0.95	0.92	0.97	0.95 (0.87-0.98)	0.92 (0.79-0.97)	0.96 (0.89-0.98)
Left rostral middle frontal gyrus	2.24 (0.09)	2.23 (0.09)	2.23 (0.08)	2.23 (0.09)	3.96	4.02	3.79	3.94	1.19 (0.25)	0.70 (0.49)	0.57 (0.58)	-0.38 (1.27)	-0.22 (1.32)	-0.15 (1.04)	1.07 (0.73)	1.06 (0.77)	0.82 (0.62)	0.95	0.94	0.96	0.95 (0.86-0.98)	0.94 (0.85-0.98)	0.97 (0.91-0.99)
Left superior frontal gyrus	2.62 (0.09)	2.62 (0.11)	2.61 (0.09)	2.62 (0.11)	3.43	4.35	3.43	4.21	-0.10 (0.92)	0.89 (0.39)	0.03 (0.97)	0.01 (1.74)	-0.34 (1.57)	-0.04 (1.97)	1.25 (1.17)	1.16 (1.07)	1.41 (1.33)	0.92	0.90	0.88	0.90 (0.74-0.96)	0.90 (0.75-0.96)	0.87 (0.66-0.95)
Left superior parietal cortex	2.23 (0.05)	2.23 (0.06)	2.23 (0.06)	2.24 (0.05)	2.16	2.46	2.57	2.33	0.11 (0.92)	0.02 (0.98)	-0.20 (0.85)	-0.04 (1.18)	-0.02 (1.36)	0.06 (1.20)	0.86 (0.77)	0.97 (0.92)	0.82 (0.85)	0.88	0.85	0.86	0.88 (0.69-0.96)	0.85 (0.61-0.94)	0.87 (0.65-0.95)
Left superior temporal gyrus	2.82 (0.12)	2.81 (0.12)	2.82 (0.11)	2.82 (0.12)	4.30	4.25	3.94	4.37	0.47 (0.65)	-0.04 (0.97)	-0.21 (0.84)	-0.15 (1.29)	0.03 (1.48)	0.05 (1.00)	1.06 (0.69)	1.11 (0.93)	0.86 (0.46)	0.96	0.94	0.97	0.96 (0.89-0.99)	0.94 (0.84-0.98)	0.97 (0.93-0.99)
Left supramarginal gyrus	2.54 (0.10)	2.54 (0.08)	2.55 (0.09)	2.54 (0.09)	3.81	3.32	3.48	3.41	-0.41 (0.69)	-1.19 (0.25)	-0.48 (0.64)	0.20 (1.81)	0.52 (1.69)	0.24 (1.86)	1.25 (1.29)	1.41 (1.01)	1.47 (1.11)	0.88	0.90	0.87	0.88 (0.69-0.96)	0.89 (0.72-0.96)	0.87 (0.67-0.95)
Left frontal pole	2.74 (0.28)	2.73 (0.23)	2.68 (0.20)	2.74 (0.26)	10.22	8.61	7.28	9.39	0.36 (0.73)	1.06 (0.31)	-0.22 (0.83)	-0.18 (3.61)	-1.89 (7.82)	0.28 (3.73)	2.85 (2.10)	5.11 (6.09)	2.92 (2.21)	0.94	0.62	0.93	0.93 (0.82-0.98)	0.58 (0.15-0.83)	0.93 (0.82-0.98)
Left temporal pole	3.75 (0.23)	3.73 (0.24)	3.74 (0.22)	3.75 (0.20)	6.13	6.42	5.87	5.27	1.18 (0.26)	0.67 (0.51)	0.20 (0.85)	-0.70 (2.35)	-0.45 (2.81)	-0.06 (2.09)	1.84 (1.56)	2.29 (1.59)	1.59 (1.29)	0.93	0.90	0.94	0.93 (0.82-0.98)	0.90 (0.74-0.96)	0.94 (0.83-0.98)
Left transverse temporal gyrus	2.67 (0.15)	2.67 (0.16)	2.67 (0.13)	2.67 (0.15)	5.71	5.89	4.92	5.78	-0.28 (0.78)	-0.16 (0.88)	-0.06 (0.96)	0.09 (1.41)	0.11 (1.86)	0.02 (1.64)	1.21 (0.66)	1.37 (1.21)	1.29 (0.95)	0.97	0.95	0.96	0.97 (0.92-0.99)	0.94 (0.85-0.98)	0.96 (0.89-0.99)
Left insula	3.11 (0.13)	3.09 (0.12)	3.06 (0.16)	3.07 (0.13)	4.30	3.92	5.15	4.24	0.92 (0.37)	1.80 (0.09)	1.12 (0.28)	-0.80 (3.58)	-1.60 (3.52)	-1.33 (4.70)	2.84 (2.21)	3.06 (2.26)	3.80 (2.94)	0.63	0.74	0.38	0.63 (0.23-0.85)	0.70 (0.34-0.89)	0.38 (-0.11-0.72)
Right banks superior temporal sulcus	2.61 (0.15)	2.60 (0.14)	2.60 (0.15)	2.59 (0.16)	5.79	5.47	5.77	6.31	0.69 (0.50)	0.91 (0.38)	1.13 (0.28)	-0.34 (2.13)	-0.38 (1.62)	-0.56 (1.84)	1.37 (1.63)	1.25 (1.05)	1.54 (1.10)	0.93	0.96	0.95	0.93 (0.82-0.98)	0.96 (0.89-0.99)	0.95 (0.87-0.98)
Right caudal anterior cingulate cortex	2.60 (0.20)	2.60 (0.21)	2.57 (0.17)	2.58 (0.21)	7.68	8.04	6.70	8.17	-0.17 (0.87)	2.43 (0.03)	0.73 (0.48)	0.07 (2.37)	-1.03 (1.77)	-0.57 (3.02)	1.86 (1.40)	1.64 (1.18)	2.38 (1.86)	0.95	0.98	0.93	0.96 (0.88-0.98)	0.96 (0.86-0.99)	0.93 (0.83-0.98)

Right caudal middle frontal gyrus	2.52 (0.09)	2.53 (0.09)	2.51 (0.10)	2.51 (0.10)	3.47	3.43	4.00	4.14	-0.54 (0.60)	0.90 (0.38)	0.99 (0.34)	0.22 (1.64)	-0.47 (2.02)	-0.60 (2.33)	1.40 (0.81)	1.71 (1.09)	1.87 (1.46)	0.89	0.86	0.83	0.89 (0.72-0.96)	0.86 (0.64-0.95)	0.81 (0.56-0.93)
Right cuneus	2.01 (0.15)	2.00 (0.14)	2.01 (0.14)	2.01 (0.15)	7.70	7.21	6.97	7.36	0.64 (0.53)	-0.57 (0.58)	-0.67 (0.52)	-0.23 (1.60)	0.31 (1.82)	0.29 (1.62)	1.23 (1.00)	1.54 (0.93)	1.24 (1.03)	0.98	0.97	0.98	0.98 (0.93-0.99)	0.97 (0.92-0.99)	0.98 (0.94-0.99)
Right entorhinal cortex	3.63 (0.37)	3.58 (0.36)	3.66 (0.34)	3.65 (0.39)	10.30	10.14	9.40	10.70	2.38 (0.03)	-2.51 (0.02)	-0.86 (0.41)	-1.29 (2.20)	0.95 (1.40)	0.44 (2.17)	2.06 (1.45)	1.44 (0.83)	1.54 (1.55)	0.98	0.99	0.98	0.97 (0.90-0.99)	0.99 (0.95-1.00)	0.98 (0.94-0.99)
Right fusiform gyrus	2.84 (0.13)	2.84 (0.13)	2.84 (0.13)	2.84 (0.14)	4.55	4.60	4.71	4.91	-0.33 (0.75)	-0.50 (0.62)	0.09 (0.93)	0.11 (1.40)	0.16 (1.30)	-0.04 (1.22)	1.15 (0.74)	1.04 (0.75)	0.82 (0.87)	0.95	0.96	0.97	0.96 (0.88-0.98)	0.96 (0.90-0.99)	0.97 (0.91-0.99)
Right inferior parietal cortex	2.43 (0.06)	2.42 (0.08)	2.42 (0.06)	2.42 (0.07)	2.66	3.11	2.66	3.07	0.87 (0.40)	0.94 (0.36)	0.76 (0.46)	-0.36 (1.60)	-0.34 (1.43)	-0.32 (1.63)	1.34 (0.88)	1.26 (0.69)	1.30 (0.99)	0.86	0.86	0.84	0.85 (0.63-0.94)	0.86 (0.64-0.95)	0.84 (0.61-0.94)
Right inferior temporal gyrus	2.74 (0.12)	2.74 (0.11)	2.73 (0.11)	2.74 (0.12)	4.29	3.99	4.00	4.52	-0.06 (0.95)	1.27 (0.22)	0.17 (0.86)	0.04 (1.69)	-0.54 (1.72)	-0.09 (1.76)	1.34 (0.96)	1.50 (0.93)	1.20 (1.25)	0.92	0.92	0.92	0.92 (0.80-0.97)	0.91 (0.77-0.97)	0.92 (0.79-0.97)
Right isthmus cingulate cortex	2.55 (0.16)	2.56 (0.14)	2.55 (0.15)	2.55 (0.14)	6.29	5.56	5.79	5.58	-0.84 (0.41)	0.00 (1.00)	-0.22 (0.83)	0.45 (1.92)	0.03 (1.90)	0.16 (2.15)	1.55 (1.17)	1.57 (0.99)	1.81 (1.08)	0.95	0.96	0.94	0.95 (0.86-0.98)	0.96 (0.88-0.98)	0.94 (0.83-0.98)
Right lateral occipital cortex	2.29 (0.09)	2.28 (0.08)	2.28 (0.08)	2.28 (0.08)	3.75	3.55	3.44	3.59	0.68 (0.51)	1.50 (0.15)	1.62 (0.13)	-0.19 (1.16)	-0.36 (0.96)	-0.42 (1.03)	0.95 (0.66)	0.84 (0.55)	0.85 (0.68)	0.95	0.97	0.96	0.95 (0.87-0.98)	0.96 (0.89-0.99)	0.96 (0.88-0.98)
Right lateral orbitofrontal cortex	2.64 (0.11)	2.63 (0.09)	2.63 (0.10)	2.62 (0.12)	4.14	3.54	3.84	4.56	1.08 (0.30)	0.57 (0.58)	1.60 (0.13)	-0.66 (2.57)	-0.41 (3.04)	-0.77 (1.89)	2.21 (1.36)	2.30 (1.95)	1.60 (1.21)	0.80	0.72	0.91	0.78 (0.49-0.92)	0.73 (0.38-0.90)	0.90 (0.73-0.96)
Right lingual gyrus	2.13 (0.09)	2.12 (0.08)	2.12 (0.09)	2.12 (0.08)	4.07	3.89	4.20	3.91	1.90 (0.08)	0.84 (0.42)	0.80 (0.44)	-0.47 (1.01)	-0.25 (1.16)	-0.26 (1.37)	0.89 (0.64)	0.91 (0.73)	1.04 (0.89)	0.97	0.96	0.94	0.96 (0.89-0.99)	0.96 (0.90-0.99)	0.94 (0.85-0.98)
Right medial orbitofrontal cortex	2.57 (0.08)	2.56 (0.06)	2.55 (0.09)	2.54 (0.09)	3.26	2.49	3.64	3.39	1.06 (0.31)	1.24 (0.23)	2.52 (0.02)	-0.52 (2.02)	-0.92 (2.95)	-1.27 (2.06)	1.68 (1.16)	2.51 (1.70)	1.96 (1.37)	0.78	0.64	0.82	0.75 (0.44-0.91)	0.63 (0.24-0.85)	0.77 (0.39-0.92)
Right middle temporal gyrus	2.86 (0.13)	2.86 (0.12)	2.84 (0.12)	2.83 (0.13)	4.61	4.33	4.27	4.51	-0.24 (0.81)	1.69 (0.11)	1.95 (0.07)	0.09 (1.35)	-0.50 (1.24)	-0.91 (1.89)	1.03 (0.83)	1.01 (0.84)	1.57 (1.36)	0.96	0.97	0.92	0.96 (0.88-0.98)	0.96 (0.88-0.99)	0.90 (0.73-0.97)
Right parahippocampal gyrus	2.99 (0.15)	2.99 (0.15)	3.01 (0.13)	3.00 (0.14)	4.90	4.91	4.43	4.71	0.53 (0.61)	-0.86 (0.40)	-0.96 (0.35)	-0.15 (1.11)	0.50 (2.18)	0.39 (1.60)	0.83 (0.72)	1.82 (1.22)	1.31 (0.95)	0.97	0.89	0.95	0.98 (0.93-0.99)	0.89 (0.71-0.96)	0.95 (0.86-0.98)
Right paracentral lobule	2.49 (0.13)	2.48 (0.13)	2.49 (0.12)	2.48 (0.12)	5.42	5.09	4.96	4.91	1.02 (0.32)	-0.50 (0.63)	0.56 (0.58)	-0.29 (1.21)	0.22 (1.58)	-0.19 (1.52)	1.06 (0.59)	1.28 (0.91)	1.22 (0.87)	0.98	0.96	0.96	0.97 (0.93-0.99)	0.95 (0.88-0.98)	0.96 (0.89-0.99)
Right pars opercularis	2.61 (0.12)	2.61 (0.11)	2.59 (0.12)	2.59 (0.12)	4.44	4.32	4.57	4.47	0.11 (0.92)	1.71 (0.11)	2.35 (0.03)	-0.03 (1.33)	-0.76 (1.74)	-0.76 (1.30)	1.08 (0.73)	1.52 (1.09)	1.19 (0.90)	0.95	0.92	0.96	0.96 (0.88-0.98)	0.92 (0.77-0.97)	0.95 (0.82-0.98)
Right pars orbitalis	2.67 (0.15)	2.67 (0.13)	2.66 (0.15)	2.66 (0.15)	5.71	5.03	5.59	5.82	-0.37 (0.72)	0.45 (0.66)	0.26 (0.80)	0.21 (1.96)	-0.20 (1.81)	-0.13 (1.84)	1.64 (1.01)	1.41 (1.10)	1.39 (1.16)	0.94	0.95	0.95	0.94 (0.83-0.98)	0.95 (0.86-0.98)	0.95 (0.86-0.98)
Right pars triangularis	2.49 (0.11)	2.49 (0.10)	2.48 (0.10)	2.49 (0.11)	4.26	4.21	4.01	4.22	0.30 (0.77)	1.09 (0.29)	0.49 (0.63)	-0.12 (1.59)	-0.41 (1.52)	-0.20 (1.67)	1.33 (0.80)	1.21 (0.96)	1.43 (0.80)	0.93	0.93	0.92	0.93 (0.82-0.98)	0.93 (0.81-0.97)	0.93 (0.80-0.97)
Right pericalcarine cortex	1.67 (0.15)	1.67 (0.15)	1.68 (0.15)	1.68 (0.16)	8.99	9.01	9.19	9.51	0.01 (0.99)	-0.69 (0.50)	-1.17 (0.26)	0.00 (2.19)	0.31 (1.92)	0.68 (2.35)	1.80 (1.16)	1.51 (1.15)	1.98 (1.36)	0.97	0.98	0.97	0.97 (0.92-0.99)	0.98 (0.94-0.99)	0.96 (0.90-0.99)
Right postcentral gyrus	2.10 (0.08)	2.09 (0.09)	2.09 (0.09)	2.09 (0.09)	3.97	4.28	4.07	4.46	1.15 (0.27)	0.75 (0.47)	1.16 (0.26)	-0.33 (1.09)	-0.23 (1.15)	-0.37 (1.20)	0.97 (0.53)	0.84 (0.79)	0.99 (0.75)	0.97	0.96	0.96	0.96 (0.90-0.99)	0.96 (0.88-0.98)	0.96 (0.88-0.98)
Right posterior cingulate cortex	2.46 (0.12)	2.47 (0.12)	2.46 (0.11)	2.46 (0.11)	4.70	4.69	4.48	4.66	-1.89 (0.08)	-0.92 (0.37)	-0.45 (0.66)	0.56 (1.18)	0.31 (1.29)	0.19 (1.66)	0.90 (0.93)	1.01 (0.82)	1.31 (0.98)	0.97	0.96	0.94	0.96 (0.89-0.99)	0.96 (0.89-0.99)	0.94 (0.84-0.98)
Right precentral gyrus	2.59 (0.11)	2.59 (0.09)	2.58 (0.09)	2.57 (0.10)	4.15	3.61	3.55	3.92	-0.06 (0.95)	0.77 (0.45)	1.82 (0.09)	0.04 (1.21)	-0.27 (1.48)	-0.62 (1.38)	1.05 (0.54)	1.29 (0.70)	1.17 (0.91)	0.96	0.93	0.94	0.95 (0.87-0.98)	0.92 (0.80-0.97)	0.93 (0.81-0.98)
Right precuneus	2.49 (0.08)	2.47 (0.07)	2.48 (0.07)	2.48 (0.07)	3.11	2.64	2.91	2.71	1.66 (0.12)	1.19 (0.25)	1.01 (0.33)	-0.55 (1.36)	-0.29 (1.00)	-0.26 (1.08)	1.24 (0.74)	0.84 (0.59)	0.85 (0.68)	0.90	0.95	0.94	0.88 (0.69-0.96)	0.94 (0.85-0.98)	0.93 (0.83-0.98)
Right rostral anterior cingulate cortex	2.98 (0.15)	2.97 (0.15)	2.99 (0.14)	2.95 (0.16)	5.06	5.00	4.61	5.53	1.06 (0.30)	-0.35 (0.73)	2.01 (0.06)	-0.47 (1.81)	0.20 (2.01)	-0.92 (1.80)	1.60 (0.88)	1.53 (1.26)	1.48 (1.34)	0.94	0.92	0.95	0.94 (0.83-0.98)	0.92 (0.79-0.97)	0.93 (0.80-0.98)
Right rostral middle frontal gyrus	2.36 (0.05)	2.36 (0.05)	2.35 (0.05)	2.35 (0.06)	2.29	2.06	2.02	2.48	-0.08 (0.94)	1.42 (0.18)	1.46 (0.17)	0.04 (1.69)	-0.66 (1.86)	-0.58 (1.56)	1.44 (0.82)	1.46 (1.29)	1.39 (0.86)	0.70	0.63	0.78	0.71 (0.33-0.89)	0.61 (0.20-0.84)	0.77 (0.47-0.91)
Right superior frontal gyrus	2.72 (0.09)	2.73 (0.09)	2.71 (0.09)	2.71 (0.11)	3.43	3.20	3.38	3.88	-0.16 (0.87)	1.07 (0.30)	1.45 (0.17)	0.08 (1.70)	-0.50 (1.89)	-0.67 (1.79)	1.22 (1.14)	1.44 (1.27)	1.44 (1.21)	0.87	0.85	0.89	0.88 (0.68-0.95)	0.85 (0.62-0.94)	0.87 (0.68-0.95)
Right superior parietal cortex	2.20 (0.07)	2.19 (0.07)	2.19 (0.07)	2.19 (0.06)	3.32	3.33	3.23	2.97	1.26 (0.23)	1.55 (0.14)	1.52 (0.15)	-0.44 (1.43)	-0.32 (0.82)	-0.59 (1.62)	1.21 (0.83)	0.69 (0.52)	1.34 (1.03)	0.91	0.97	0.88	0.91 (0.76-0.97)	0.96 (0.90-0.99)	0.86 (0.66-0.95)
Right superior temporal gyrus	2.95 (0.12)	2.94 (0.12)	2.93 (0.13)	2.93 (0.14)	4.14	4.12	4.35	4.78	0.84 (0.41)	1.37 (0.19)	1.34 (0.20)	-0.29 (1.36)	-0.41 (1.16)	-0.47 (1.27)	0.96 (0.97)	0.95 (0.74)	1.03 (0.85)	0.95	0.96	0.97	0.95 (0.86-0.98)	0.96 (0.89-0.99)	0.95 (0.88-0.98)
Right supramarginal gyrus	2.51 (0.08)	2.50 (0.08)	2.50 (0.08)	2.49 (0.08)	3.12	3.07	3.22	3.21	0.81 (0.43)	0.54 (0.60)	1.28 (0.22)	-0.26 (1.30)	-0.21 (1.56)	-0.49 (1.53)	1.12 (0.66)	1.30 (0.82)	1.34 (0.83)	0.91	0.88	0.88	0.92 (0.78-0.97)	0.88 (0.70-0.96)	0.88 (0.70-0.96)
Right frontal pole	2.66 (0.13)	2.68 (0.14)	2.60 (0.16)	2.67 (0.14)	4.70	5.23	6.08	5.42	-0.56 (0.59)	1.42 (0.18)	-0.53 (0.60)	0.44 (3.35)	-2.69 (7.60)	0.32 (2.64)	2.61 (2.05)	4.49 (6.63)	2.25 (1.30)	0.77	0.07	0.87	0.78 (0.47-0.92)	0.07 (-0.39-0.52)	0.86 (0.66-0.95)
Right temporal pole	3.83 (0.28)	3.81 (0.31)	3.82 (0.30)	3.83 (0.31)	7.26	8.06	7.81	8.08	0.42 (0.68)	0.17 (0.87)	-0.21 (0.84)	-0.45 (3.95)	-0.16 (3.30)	0.10 (3.18)	2.80 (2.73)	2.23 (2.37)	2.07 (2.35)	0.88	0.92	0.93	0.88 (0.70-0.96)	0.92 (0.80-0.97)	0.93 (0.80-0.97)
Right transverse temporal gyrus	2.74 (0.21)	2.73 (0.20)	2.75 (0.21)	2.74 (0.20)	7.65	7.48	7.70	7.31	1.01 (0.33)	-0.39 (0.70)	0.27 (0.79)	-0.27 (1.12)	0.12 (1.28)	-0.06 (1.29)	0.91 (0.67)	1.07 (0.65)	0.99 (0.80)	0.99	0.99	0.99	0.99 (0.97-1.00)	0.99 (0.96-1.00)	0.99 (0.96-1.00)
Right insula	3.14 (0.16)	3.16 (0.14)	3.13 (0.15)	3.14 (0.09)	4.97	4.43	4.65	2.77	-0.78 (0.45)	1.06 (0.30)	0.05 (0.96)	0.54 (2.70)	-0.50 (2.04)	0.04 (3.47)	2.17 (1.60)	1.46 (1.46)	2.44 (2.38)	0.85	0.92	0.77	0.85 (0.62-0.94)	0.92 (0.79-0.97)	0.67 (0.27-0.87)
Global brain measures																							
Left total cortical volume (ml)	250.57 (21.45)	250.33 (21.43)	250.59 (22.81)	250.78 (21.99)	8.56	8.56	9.10	8.77	0.39 (0.70)	-0.03 (0.98)	-0.31 (0.76)	-0.10 (0.98)	-0.04 (1.02)	0.07 (1.07)	0.72 (0.63)	0.68 (0.73)	0.71 (0.79)	0.99	0.99	0.99	0.99 (0.98-1.00)	0.99 (0.98-1.00)	0.99 (0.98-1.00)
Right total cortical volume (ml)	252.63 (22.32)	252.52 (22.69)	251.69 (22.47)	251.84 (21.50)	8.83	8.98	8.93	8.54	0.16 (0.88)	1.40 (0.18)	1.05 (0.31)	-0.05 (1.08)	-0.38 (1.09)	-0.29 (1.23)	0.86 (0.62)	0.92 (0.66)	0.86 (0.89)	0.99	0.99	0.99	0.99 (0.98-1.00)	0.99 (0.98-1.00)	0.99 (0.97-1.00)
Left total cortical white matter (ml)	227.42 (34.18)	227.87 (34.01)	227.63 (34.79)	228.08 (34.45)	15.03	14.92	15.29	15.10	-1.52 (0.15)	-0.49 (0.63)	-1.93 (0.07)	0.21 (0.45)	0.05 (0.68)	0.28 (0.58)	0.38 (0.32)	0.51 (0.44)	0.53 (0.35)	1.00	1.00	1.00	1.00 (1.00-1.00)	1.00 (1.00-1.00)	1.00 (1.00-1.00)
Right total cortical white matter (ml)	228.37 (35.52)	228.69 (35.53)	228.07 (35.46)	228.44 (35.33)	15.56	15.54	15.55	15.47	-1.78 (0.09)	2.10 (0.05)	-0.27 (0.79)	0.14 (0.31)	-0.13 (0.24)	0.04 (0.44)	0.27 (0.21)	0.24 (0.12)	0.35 (0.25)	1.00	1.00	1.00	1.00 (1.00-1.00)	1.00 (1.00-1.00)	1.00 (1.00-1.00)

Left total cortical surface area (cm²)	894.23 (95.31)	894.59 (95.92)	895.06 (97.01)	896.96 (96.45)	10.66	10.72	10.84	10.75	-0.74 (0.47)	-1.27 (0.22)	-3.54 (0.00)	0.03 (0.22)	0.08 (0.28)	0.30 (0.36)	0.18 (0.12)	0.22 (0.18)	0.36 (0.28)	1.00	1.00	1.00	1.00 (1.00-1.00)	1.00 (1.00-1.00)	1.00 (0.99-1.00)
Right total cortical surface area (cm²)	895.33 (96.45)	895.73 (96.95)	894.40 (97.20)	895.88 (95.86)	10.77	10.82	10.87	10.70	-0.62 (0.54)	1.88 (0.08)	-0.82 (0.42)	0.04 (0.28)	-0.11 (0.22)	0.07 (0.31)	0.21 (0.18)	0.20 (0.14)	0.23 (0.21)	1.00	1.00	1.00	1.00 (1.00-1.00)	1.00 (1.00-1.00)	1.00 (1.00-1.00)
Left mean cortical thickness (mm)	2.49 (0.06)	2.49 (0.06)	2.49 (0.05)	2.49 (0.06)	2.26	2.49	2.13	2.32	0.55 (0.59)	0.06 (0.95)	0.26 (0.80)	-0.14 (0.97)	-0.01 (1.07)	-0.07 (1.05)	0.76 (0.58)	0.74 (0.75)	0.80 (0.64)	0.92	0.89	0.89	0.92 (0.79-0.97)	0.89 (0.72-0.96)	0.90 (0.73-0.96)
Right mean cortical tickness (mm)	2.52 (0.05)	2.52 (0.05)	2.51 (0.05)	2.51 (0.06)	2.08	1.89	1.87	2.25	0.63 (0.54)	1.19 (0.25)	1.47 (0.16)	-0.17 (1.07)	-0.33 (1.13)	-0.41 (1.10)	0.90 (0.56)	0.93 (0.68)	0.86 (0.78)	0.86	0.84	0.87	0.86 (0.64-0.95)	0.83 (0.59-0.94)	0.86 (0.65-0.95)
Left cerebellar white matter (ml)	15.25 (1.48)	15.14 (1.59)	15.21 (1.59)	15.17 (1.68)	9.73	10.49	10.47	11.08	1.38 (0.19)	0.94 (0.36)	0.76 (0.46)	-0.83 (2.37)	-0.39 (1.41)	-0.69 (3.05)	1.66 (1.85)	1.07 (0.96)	2.19 (2.17)	0.98	0.99	0.97	0.98 (0.93-0.99)	0.99 (0.98-1.00)	0.97 (0.91-0.99)
Right cerebellar white matter (ml)	14.48 (1.58)	14.24 (1.64)	14.38 (1.62)	14.37 (1.55)	10.94	11.50	11.28	10.79	2.29 (0.04)	1.19 (0.25)	1.40 (0.18)	-1.70 (2.84)	-0.76 (2.52)	-0.75 (2.18)	2.51 (2.10)	1.94 (1.71)	1.67 (1.54)	0.97	0.98	0.98	0.96 (0.86-0.99)	0.97 (0.93-0.99)	0.98 (0.94-0.99)
Left cerebellar gray matter (ml)	55.82 (5.28)	55.83 (5.30)	55.75 (5.32)	55.82 (5.23)	9.46	9.49	9.53	9.37	-0.03 (0.97)	0.64 (0.53)	-0.03 (0.98)	0.00 (0.82)	-0.13 (0.82)	0.01 (0.70)	0.56 (0.58)	0.57 (0.59)	0.47 (0.50)	1.00	1.00	1.00	1.00 (0.99-1.00)	1.00 (0.99-1.00)	1.00 (0.99-1.00)
Right cerebellar gray matter (ml)	54.79 (5.44)	54.83 (5.50)	54.61 (5.49)	54.93 (5.41)	9.92	10.03	10.06	9.85	-0.31 (0.76)	1.41 (0.18)	-1.20 (0.25)	0.05 (0.80)	-0.35 (1.02)	0.26 (0.86)	0.56 (0.56)	0.60 (0.88)	0.66 (0.59)	1.00	1.00	1.00	1.00 (0.99-1.00)	1.00 (0.99-1.00)	1.00 (0.99-1.00)
Intracranial volume (ml)	1484.47 (258.06)	1497.06 (242.06)	1437.35 (264.02)	1516.68 (233.88)	17.38	16.17	18.37	15.42	-1.39 (0.18)	2.99 (0.01)	-2.15 (0.05)	1.06 (3.10)	-3.38 (5.25)	2.46 (4.65)	1.17 (3.06)	3.38 (5.25)	2.47 (4.65)	0.99	0.97	0.97	0.99 (0.97-1.00)	0.96 (0.80-0.99)	0.96 (0.88-0.99)
Total brain volume without ventricles (ml)	1158.90 (124.07)	1158.88 (123.80)	1157.40 (126.10)	1158.77 (123.46)	10.71	10.68	10.89	10.65	0.01 (0.99)	0.90 (0.38)	0.07 (0.94)	0.00 (0.52)	-0.15 (0.56)	-0.01 (0.68)	0.38 (0.35)	0.41 (0.39)	0.42 (0.52)	1.00	1.00	1.00	1.00 (1.00-1.00)	1.00 (1.00-1.00)	1.00 (1.00-1.00)

Abbreviations: SD = standard deviation; Orig = original scan; FS df = FreeSurfer defacing; FSL df = FSL defacing; FM = Face Masking; t = t-statistic; *p*=p-value; PD = Average Percentage Difference; CI = Confidence Interval.

Table S3. Effects of de-identification on brain measures in older adults.

Brain metric	Mean (SD)				Coefficient of Variation				Paired t-test t(*p*)			Signed PD in %			Absolute PD (%)			Pearson's r			ICC (95% CI)		
	Orig	FS df	FSL df	FM	Orig	FS df	FSL df	FM	FS df	FSL df	FM	FS df	FSL df	FM	FS df	FSL df	FM	FS df	FSL df	FM	FS df	FSL df	FM
Subcortical volume (ml)																							
Left thalamus	6.42 (0.80)	6.42 (0.78)	6.47 (0.81)	6.41 (0.79)	12.41	12.18	12.53	12.35	0.01 (0.99)	-1.74 (0.09)	0.16 (0.88)	0.04 (3.43)	0.78 (3.00)	-0.08 (3.85)	2.12 (2.67)	2.46 (1.85)	2.72 (2.69)	0.95	0.97	0.94	0.95 (0.91-0.97)	0.97 (0.94-0.98)	0.94 (0.90-0.97)
Left caudatus	3.13 (0.41)	3.12 (0.41)	3.11 (0.39)	3.14 (0.40)	12.98	13.05	12.62	12.77	0.08 (0.93)	0.73 (0.47)	-0.99 (0.33)	-0.06 (3.53)	-0.38 (4.00)	0.53 (3.08)	2.32 (2.64)	2.34 (3.25)	2.16 (2.24)	0.96	0.95	0.97	0.96 (0.93-0.98)	0.95 (0.91-0.97)	0.97 (0.95-0.98)
Left putamen	4.10 (0.56)	4.14 (0.48)	4.09 (0.55)	4.11 (0.50)	13.57	11.49	13.54	12.20	-1.28 (0.21)	0.54 (0.59)	-0.43 (0.67)	1.31 (6.64)	-0.21 (2.88)	0.49 (5.24)	2.50 (6.28)	2.04 (2.01)	2.28 (4.73)	0.94	0.98	0.96	0.92 (0.86-0.96)	0.98 (0.96-0.99)	0.96 (0.92-0.98)
Left pallidum	1.91 (0.20)	1.92 (0.20)	1.92 (0.18)	1.89 (0.19)	10.39	10.33	9.54	9.97	-0.60 (0.55)	-0.24 (0.81)	1.51 (0.14)	0.31 (3.19)	0.24 (4.62)	-0.89 (3.98)	2.60 (1.84)	3.55 (2.92)	2.93 (2.81)	0.95	0.89	0.92	0.95 (0.90-0.97)	0.89 (0.81-0.94)	0.92 (0.86-0.96)
Left hippocampus	3.34 (0.55)	3.31 (0.56)	3.33 (0.56)	3.31 (0.56)	16.60	16.92	16.74	16.84	2.36 (0.02)	1.05 (0.30)	2.72 (0.01)	-0.96 (2.64)	-0.42 (2.58)	-0.92 (2.15)	2.15 (1.79)	1.92 (1.74)	1.77 (1.52)	0.99	0.99	0.99	0.99 (0.98-0.99)	0.99 (0.98-0.99)	0.99 (0.98-1.00)
Left amygdala	1.34 (0.27)	1.34 (0.27)	1.33 (0.27)	1.30 (0.26)	20.32	19.90	20.44	20.39	-0.38 (0.71)	0.91 (0.37)	4.48 (0.00)	0.36 (4.77)	-0.50 (3.66)	-3.03 (4.79)	3.60 (3.10)	2.95 (2.18)	4.19 (3.79)	0.97	0.99	0.98	0.97 (0.95-0.98)	0.99 (0.98-0.99)	0.97 (0.89-0.99)
Left accumbens	0.36 (0.09)	0.35 (0.09)	0.36 (0.09)	0.36 (0.08)	25.12	24.38	23.59	23.59	0.70 (0.49)	-0.41 (0.68)	0.27 (0.79)	-0.95 (10.04)	0.91 (9.79)	-0.22 (11.23)	8.00 (6.02)	7.68 (6.02)	8.24 (7.53)	0.92	0.93	0.93	0.92 (0.86-0.96)	0.93 (0.88-0.96)	0.93 (0.87-0.96)
Right thalamus	6.16 (0.64)	6.21 (0.67)	6.17 (0.66)	6.21 (0.70)	10.42	10.76	10.70	11.21	-2.24 (0.03)	-0.19 (0.85)	-1.58 (0.12)	0.81 (2.30)	0.10 (3.52)	0.68 (2.86)	1.86 (1.56)	2.35 (2.60)	2.13 (2.00)	0.97	0.94	0.96	0.97 (0.94-0.98)	0.94 (0.89-0.97)	0.96 (0.92-0.98)
Right caudatus	3.25 (0.38)	3.26 (0.38)	3.23 (0.38)	3.24 (0.40)	11.82	11.62	11.70	12.30	-0.92 (0.36)	1.01 (0.32)	0.34 (0.73)	0.46 (2.91)	-0.46 (2.87)	-0.27 (3.97)	2.09 (2.05)	1.96 (2.13)	2.57 (3.01)	0.97	0.97	0.94	0.97 (0.94-0.98)	0.97 (0.94-0.98)	0.94 (0.90-0.97)
Right putamen	4.17 (0.52)	4.20 (0.52)	4.21 (0.52)	4.16 (0.52)	12.51	12.48	12.45	12.50	-1.85 (0.07)	-2.90 (0.01)	0.49 (0.62)	0.81 (3.11)	1.02 (2.25)	-0.27 (3.74)	2.22 (2.31)	1.77 (1.71)	2.29 (2.96)	0.97	0.98	0.96	0.97 (0.95-0.99)	0.98 (0.96-0.99)	0.96 (0.92-0.98)
Right pallidum	1.81 (0.22)	1.81 (0.21)	1.82 (0.22)	1.81 (0.23)	12.19	11.43	12.13	12.58	0.71 (0.48)	-0.50 (0.62)	0.39 (0.70)	-0.34 (4.08)	0.28 (3.59)	-0.40 (5.27)	3.06 (2.68)	3.02 (1.91)	3.81 (3.61)	0.95	0.96	0.90	0.95 (0.90-0.97)	0.96 (0.92-0.98)	0.90 (0.82-0.94)
Right hippocampus	3.50 (0.58)	3.48 (0.60)	3.50 (0.58)	3.50 (0.58)	16.69	17.19	16.69	16.56	1.90 (0.06)	0.64 (0.53)	0.75 (0.46)	-0.66 (2.15)	-0.19 (2.37)	-0.16 (1.91)	1.67 (1.49)	1.78 (1.55)	1.56 (1.08)	0.99	0.99	0.99	0.99 (0.99-1.00)	0.99 (0.99-1.00)	0.99 (0.99-1.00)
Right amygdala	1.58 (0.27)	1.59 (0.27)	1.59 (0.28)	1.57 (0.27)	17.12	16.65	17.32	17.45	-0.95 (0.35)	-0.87 (0.39)	1.87 (0.07)	0.68 (3.89)	0.36 (2.97)	-1.05 (3.50)	2.91 (2.63)	2.41 (1.72)	2.89 (2.20)	0.98	0.98	0.98	0.98 (0.96-0.99)	0.98 (0.97-0.99)	0.98 (0.96-0.99)
Right accumbens	0.45 (0.09)	0.45 (0.09)	0.46 (0.09)	0.45 (0.08)	19.99	20.67	19.81	18.72	-0.46 (0.65)	-2.20 (0.03)	-1.43 (0.16)	0.34 (6.88)	2.23 (6.73)	1.86 (7.65)	5.10 (4.55)	5.04 (4.94)	6.26 (4.69)	0.94	0.95	0.93	0.94 (0.90-0.97)	0.94 (0.89-0.97)	0.93 (0.87-0.96)
Ventricular volume (ml)																							
Third ventricle	1.99 (0.77)	1.98 (0.75)	1.95 (0.73)	1.97 (0.73)	38.84	38.06	37.30	36.88	0.42 (0.68)	0.94 (0.35)	0.62 (0.54)	-0.26 (3.54)	-1.35 (7.80)	-0.54 (7.39)	1.93 (2.96)	2.76 (7.41)	3.12 (6.71)	0.99	0.95	0.96	0.99 (0.98-0.99)	0.95 (0.91-0.97)	0.95 (0.92-0.98)
Left lateral ventricle	22.72 (11.54)	22.72 (11.55)	22.69 (11.40)	22.73 (11.56)	50.78	50.86	50.25	50.87	0.09 (0.93)	0.37 (0.71)	-0.41 (0.69)	-0.09 (0.75)	0.03 (1.13)	0.00 (0.78)	0.53 (0.53)	0.71 (0.87)	0.60 (0.49)	1.00	1.00	1.00	1.00 (1.00-1.00)	1.00 (1.00-1.00)	1.00 (1.00-1.00)
Left inferior lateral ventricle	1.06 (1.00)	1.06 (1.01)	1.06 (0.99)	1.05 (0.99)	94.71	95.42	93.45	94.86	-0.05 (0.96)	0.33 (0.74)	1.08 (0.29)	-0.53 (13.27)	0.45 (13.02)	-2.28 (12.95)	8.75 (9.90)	7.84 (10.34)	8.56 (9.91)	1.00	1.00	1.00	1.00 (0.99-1.00)	1.00 (0.99-1.00)	1.00 (0.99-1.00)
Right lateral ventricle	19.46 (8.92)	19.45 (8.92)	19.51 (9.04)	19.45 (8.93)	45.85	45.87	46.35	45.93	0.64 (0.52)	-1.30 (0.20)	0.53 (0.60)	-0.11 (0.89)	0.07 (0.74)	-0.12 (0.88)	0.62 (0.64)	0.51 (0.55)	0.68 (0.57)	1.00	1.00	1.00	1.00 (1.00-1.00)	1.00 (1.00-1.00)	1.00 (1.00-1.00)
Right inferior lateral ventricle	0.92 (0.82)	0.94 (0.84)	0.93 (0.83)	0.92 (0.81)	88.26	89.58	89.25	88.13	-2.11 (0.04)	-1.19 (0.24)	0.42 (0.68)	1.71 (7.25)	0.41 (9.24)	-0.66 (7.60)	5.22 (5.26)	5.63 (7.28)	5.01 (5.70)	1.00	1.00	1.00	1.00 (1.00-1.00)	1.00 (1.00-1.00)	1.00 (1.00-1.00)
Local cortical volume (ml)																							
Left banks superior temporal sulcus	1.99 (0.34)	1.96 (0.32)	1.97 (0.33)	1.98 (0.35)	17.06	16.54	16.63	17.86	1.51 (0.14)	1.17 (0.25)	0.41 (0.68)	-1.21 (5.62)	-0.63 (4.40)	-0.40 (4.55)	3.69 (4.37)	3.26 (2.98)	3.60 (2.75)	0.94	0.97	0.97	0.94 (0.90-0.97)	0.97 (0.95-0.98)	0.97 (0.94-0.98)
Left caudal anterior cingulate cortex	1.50 (0.42)	1.52 (0.40)	1.52 (0.39)	1.53 (0.39)	27.83	26.16	25.74	25.65	-1.16 (0.25)	-0.72 (0.47)	-1.75 (0.09)	2.30 (13.94)	2.00 (13.90)	3.20 (13.81)	6.15 (12.69)	5.77 (12.77)	6.09 (12.78)	0.96	0.96	0.96	0.96 (0.92-0.98)	0.95 (0.92-0.97)	0.95 (0.92-0.98)
Left caudal middle frontal gyrus	5.23 (0.94)	5.21 (0.96)	5.21 (0.96)	5.22 (0.96)	18.00	18.34	18.50	18.33	1.23 (0.23)	1.00 (0.32)	0.53 (0.60)	-0.47 (2.17)	-0.45 (2.45)	-0.27 (2.68)	1.80 (1.27)	1.80 (1.70)	2.17 (1.56)	0.99	0.99	0.99	0.99 (0.99-1.00)	0.99 (0.99-1.00)	0.99 (0.98-0.99)
Left cuneus	2.74 (0.43)	2.75 (0.44)	2.76 (0.43)	2.76 (0.43)	15.86	15.90	15.61	15.59	-0.77 (0.45)	-1.38 (0.17)	-0.78 (0.44)	0.33 (2.87)	0.93 (4.55)	0.68 (5.65)	2.15 (1.91)	2.66 (3.79)	2.64 (5.02)	0.98	0.96	0.94	0.98 (0.97-0.99)	0.96 (0.93-0.98)	0.94 (0.89-0.97)
Left entorhinal cortex	1.74 (0.52)	1.75 (0.49)	1.77 (0.44)	1.79 (0.47)	29.81	27.93	24.87	26.20	-0.46 (0.65)	-1.23 (0.23)	-2.05 (0.05)	1.12 (8.87)	3.45 (13.49)	3.89 (11.03)	7.15 (5.27)	7.76 (11.51)	8.15 (8.31)	0.96	0.93	0.95	0.96 (0.92-0.98)	0.92 (0.86-0.96)	0.95 (0.90-0.97)
Left fusiform gyrus	8.37 (1.29)	8.37 (1.24)	8.35 (1.28)	8.37 (1.26)	15.43	14.82	15.31	15.08	-0.06 (0.95)	0.63 (0.53)	-0.08 (0.94)	0.14 (3.08)	-0.28 (3.25)	0.09 (3.26)	2.05 (2.29)	2.26 (2.32)	2.16 (2.42)	0.98	0.98	0.98	0.98 (0.97-0.99)	0.98 (0.97-0.99)	0.98 (0.97-0.99)
Left inferior parietal cortex	9.56 (1.85)	9.43 (1.85)	9.55 (1.89)	9.59 (1.87)	19.37	19.65	19.79	19.53	1.61 (0.11)	0.25 (0.80)	-0.87 (0.39)	-1.32 (5.89)	-0.17 (2.09)	0.30 (2.63)	2.58 (5.44)	1.72 (1.18)	1.95 (1.77)	0.96	0.99	0.99	0.96 (0.93-0.98)	0.99 (0.99-1.00)	0.99 (0.99-1.00)
Left inferior temporal gyrus	9.30 (1.51)	9.30 (1.54)	9.32 (1.56)	9.33 (1.53)	16.26	16.60	16.72	16.37	-0.04 (0.97)	-0.43 (0.67)	-0.77 (0.45)	-0.04 (2.29)	0.10 (2.98)	0.23 (2.33)	1.81 (1.39)	2.37 (1.77)	1.69 (1.61)	0.99	0.99	0.99	0.99 (0.98-0.99)	0.99 (0.97-0.99)	0.99 (0.98-1.00)
Left isthmus cingulate cortex	2.29 (0.42)	2.27 (0.42)	2.27 (0.43)	2.30 (0.42)	18.43	18.66	18.78	18.39	0.83 (0.41)	1.38 (0.18)	-0.93 (0.36)	-0.72 (5.72)	-0.88 (4.29)	0.55 (4.52)	4.29 (3.78)	3.31 (2.83)	3.30 (3.10)	0.95	0.98	0.98	0.95 (0.92-0.97)	0.98 (0.96-0.99)	0.98 (0.96-0.99)
Left lateral occipital cortex	10.73 (1.62)	10.83 (1.65)	10.75 (1.59)	10.77 (1.62)	15.09	15.28	14.78	15.06	-2.04 (0.05)	-0.41 (0.69)	-1.04 (0.30)	0.81 (2.88)	0.21 (2.70)	0.32 (2.19)	1.84 (2.35)	1.97 (1.84)	1.58 (1.53)	0.98	0.98	0.99	0.98 (0.97-0.99)	0.98 (0.97-0.99)	0.99 (0.98-1.00)
Left lateral orbitofrontal cortex	6.75 (0.71)	6.73 (0.71)	6.71 (0.71)	6.75 (0.71)	10.56	10.59	10.65	10.45	0.97 (0.34)	1.51 (0.14)	-0.13 (0.90)	-0.32 (2.07)	-0.50 (2.11)	0.06 (2.40)	1.69 (1.21)	1.63 (1.40)	1.83 (1.53)	0.98	0.98	0.97	0.98 (0.96-0.99)	0.98 (0.96-0.99)	0.97 (0.95-0.99)
Left lingual gyrus	6.07 (0.90)	6.09 (0.91)	6.08 (0.91)	6.09 (0.90)	14.76	14.91	15.05	14.72	-0.44 (0.66)	-0.30 (0.77)	-0.85 (0.40)	0.16 (2.90)	0.06 (1.85)	0.25 (1.79)	1.78 (2.28)	1.45 (1.13)	1.27 (1.26)	0.98	0.99	0.99	0.98 (0.97-0.99)	0.99 (0.98-1.00)	0.99 (0.99-1.00)
Left medial orbitofrontal cortex	4.57 (0.55)	4.54 (0.56)	4.54 (0.54)	4.59 (0.54)	12.00	12.32	11.97	11.77	0.78 (0.44)	1.21 (0.23)	-0.54 (0.59)	-0.69 (5.61)	-0.80 (4.40)	0.41 (4.72)	4.08 (3.87)	3.63 (2.55)	3.60 (3.03)	0.90	0.93	0.92	0.90 (0.82-0.94)	0.93 (0.88-0.96)	0.92 (0.87-0.96)

Left middle temporal gyrus	9.10 (1.39)	9.07 (1.50)	9.05 (1.45)	9.05 (1.46)	15.33	16.50	16.01	16.11	0.62 (0.54)	1.83 (0.07)	1.71 (0.09)	-0.52 (3.61)	-0.73 (2.31)	-0.70 (2.37)	2.06 (2.99)	1.84 (1.55)	1.73 (1.76)	0.98	0.99	0.99	0.98 (0.96-0.99)	0.99 (0.98-0.99)	0.99 (0.98-0.99)
Left parahippocampal gyrus	1.84 (0.36)	1.85 (0.38)	1.85 (0.37)	1.87 (0.38)	19.31	20.36	19.73	20.63	-0.55 (0.58)	-0.62 (0.54)	-1.44 (0.16)	0.17 (4.33)	0.37 (4.39)	1.01 (5.08)	3.41 (2.61)	3.25 (2.93)	3.88 (3.38)	0.98	0.98	0.97	0.98 (0.96-0.99)	0.98 (0.96-0.99)	0.97 (0.94-0.98)
Left paracentral lobule	3.21 (0.43)	3.19 (0.45)	3.20 (0.44)	3.20 (0.44)	13.42	14.15	13.74	13.73	1.40 (0.17)	1.10 (0.28)	0.71 (0.48)	-0.83 (3.38)	-0.56 (3.14)	-0.35 (2.81)	2.68 (2.18)	2.62 (1.78)	2.33 (1.56)	0.97	0.97	0.98	0.97 (0.94-0.98)	0.97 (0.95-0.98)	0.98 (0.96-0.99)
Left pars opercularis	3.94 (0.53)	3.98 (0.54)	3.96 (0.56)	3.95 (0.52)	13.38	13.64	14.20	13.29	-1.66 (0.10)	-1.15 (0.26)	-0.37 (0.72)	0.93 (3.45)	0.38 (2.90)	0.16 (2.52)	2.59 (2.43)	2.25 (1.84)	2.01 (1.49)	0.96	0.98	0.98	0.96 (0.93-0.98)	0.98 (0.96-0.99)	0.98 (0.97-0.99)
Left pars orbitalis	2.16 (0.33)	2.14 (0.35)	2.16 (0.34)	2.14 (0.34)	15.14	16.17	15.75	15.74	1.23 (0.22)	-0.12 (0.91)	1.31 (0.20)	-1.05 (4.61)	-0.05 (3.46)	-0.86 (3.94)	3.77 (2.80)	2.75 (2.05)	3.02 (2.64)	0.96	0.98	0.97	0.96 (0.92-0.98)	0.98 (0.96-0.99)	0.97 (0.94-0.98)
Left pars triangularis	3.18 (0.45)	3.15 (0.46)	3.16 (0.45)	3.17 (0.45)	14.17	14.50	14.15	14.33	1.85 (0.07)	1.06 (0.30)	0.31 (0.76)	-0.82 (2.86)	-0.44 (2.75)	-0.18 (3.42)	2.38 (1.76)	2.17 (1.71)	2.68 (2.09)	0.98	0.98	0.97	0.98 (0.96-0.99)	0.98 (0.97-0.99)	0.97 (0.95-0.99)
Left pericalcarine cortex	1.93 (0.30)	1.96 (0.35)	1.92 (0.31)	1.93 (0.31)	15.50	17.97	16.36	16.06	-0.79 (0.44)	0.85 (0.40)	-0.80 (0.43)	1.35 (12.32)	-0.60 (3.72)	0.35 (3.74)	4.76 (11.42)	3.09 (2.10)	2.80 (2.46)	0.64	0.98	0.97	0.64 (0.42-0.78)	0.97 (0.95-0.99)	0.97 (0.95-0.99)
Left postcentral gyrus	8.44 (1.12)	8.43 (1.12)	8.41 (1.13)	8.45 (1.12)	13.27	13.27	13.47	13.27	0.27 (0.79)	1.74 (0.09)	-0.37 (0.71)	-0.10 (2.40)	-0.41 (1.42)	0.09 (1.65)	1.42 (1.93)	1.18 (0.87)	1.26 (1.05)	0.99	0.99	0.99	0.99 (0.97-0.99)	0.99 (0.99-1.00)	0.99 (0.99-1.00)
Left posterior cingulate cortex	2.66 (0.49)	2.64 (0.47)	2.66 (0.47)	2.67 (0.49)	18.39	17.80	17.58	18.49	0.67 (0.51)	0.05 (0.96)	-0.75 (0.46)	-0.41 (5.25)	0.11 (11.28)	0.59 (5.30)	3.87 (3.52)	5.30 (9.92)	3.96 (3.53)	0.96	0.87	0.96	0.96 (0.93-0.98)	0.88 (0.78-0.93)	0.96 (0.94-0.98)
Left precentral gyrus	12.26 (1.44)	12.26 (1.44)	12.27 (1.46)	12.26 (1.45)	11.75	11.77	11.93	11.84	0.21 (0.83)	-0.09 (0.93)	0.21 (0.83)	-0.06 (1.68)	0.00 (1.64)	-0.06 (1.76)	1.30 (1.05)	1.30 (0.98)	1.44 (0.99)	0.99	0.99	0.99	0.99 (0.98-0.99)	0.99 (0.98-1.00)	0.99 (0.98-0.99)
Left precuneus	8.14 (1.20)	8.11 (1.25)	8.12 (1.25)	8.12 (1.23)	14.79	15.38	15.44	15.10	0.92 (0.36)	1.05 (0.30)	1.10 (0.28)	-0.41 (2.47)	-0.39 (2.03)	-0.35 (1.90)	1.58 (1.92)	1.51 (1.40)	1.46 (1.25)	0.99	0.99	0.99	0.99 (0.98-0.99)	0.99 (0.99-1.00)	0.99 (0.99-1.00)
Left rostral anterior cingulate cortex	2.31 (0.55)	2.29 (0.46)	2.31 (0.51)	2.33 (0.52)	23.65	19.97	22.11	22.53	0.99 (0.33)	0.03 (0.98)	-0.86 (0.39)	0.03 (7.83)	0.54 (7.14)	1.25 (8.09)	4.50 (6.38)	3.66 (6.13)	4.49 (6.81)	0.97	0.98	0.97	0.96 (0.92-0.98)	0.98 (0.96-0.99)	0.97 (0.95-0.98)
Left rostral middle frontal gyrus	13.13 (1.95)	13.10 (1.98)	13.16 (1.96)	13.13 (1.93)	14.88	15.13	14.91	14.74	0.38 (0.71)	-0.74 (0.47)	-0.13 (0.90)	-0.19 (2.89)	0.24 (2.08)	0.05 (2.18)	1.91 (2.16)	1.66 (1.25)	1.75 (1.27)	0.98	0.99	0.99	0.98 (0.97-0.99)	0.99 (0.98-0.99)	0.99 (0.98-0.99)
Left superior frontal gyrus	19.35 (2.73)	19.28 (2.71)	19.33 (2.76)	19.35 (2.74)	14.12	14.04	14.26	14.14	1.28 (0.21)	0.49 (0.63)	-0.01 (1.00)	-0.32 (1.70)	-0.12 (1.37)	0.00 (1.57)	1.07 (1.35)	0.96 (0.97)	1.14 (1.07)	0.99	1.00	0.99	0.99 (0.99-1.00)	1.00 (0.99-1.00)	0.99 (0.99-1.00)
Left superior parietal cortex	11.37 (1.80)	11.29 (1.88)	11.34 (1.79)	11.36 (1.77)	15.87	16.68	15.80	15.59	1.24 (0.22)	0.98 (0.33)	0.40 (0.69)	-0.90 (5.04)	-0.23 (1.52)	-0.07 (1.72)	1.90 (4.75)	1.20 (0.94)	1.39 (1.00)	0.98	1.00	0.99	0.97 (0.95-0.99)	1.00 (0.99-1.00)	0.99 (0.99-1.00)
Left superior temporal gyrus	10.66 (1.47)	10.69 (1.52)	10.67 (1.53)	10.72 (1.53)	13.82	14.22	14.33	14.27	-0.77 (0.44)	-0.40 (0.69)	-1.60 (0.12)	0.16 (1.89)	0.04 (1.95)	0.45 (2.15)	1.41 (1.25)	1.53 (1.19)	1.68 (1.39)	0.99	0.99	0.99	0.99 (0.98-1.00)	0.99 (0.98-1.00)	0.99 (0.98-0.99)
Left supramarginal gyrus	9.54 (1.46)	9.53 (1.52)	9.56 (1.52)	9.56 (1.50)	15.32	15.98	15.94	15.65	0.16 (0.88)	-0.52 (0.60)	-0.58 (0.57)	-0.20 (4.72)	0.06 (1.87)	0.11 (1.94)	2.06 (4.24)	1.45 (1.16)	1.45 (1.27)	0.96	0.99	0.99	0.96 (0.92-0.98)	0.99 (0.99-1.00)	0.99 (0.99-1.00)
Left frontal pole	0.88 (0.14)	0.87 (0.14)	0.89 (0.14)	0.87 (0.15)	16.22	16.31	15.37	16.97	1.28 (0.21)	-0.52 (0.60)	1.79 (0.08)	-1.53 (7.78)	0.68 (7.52)	-1.75 (5.99)	6.36 (4.64)	6.41 (3.85)	4.89 (3.80)	0.88	0.89	0.93	0.88 (0.79-0.93)	0.89 (0.80-0.94)	0.93 (0.87-0.96)
Left temporal pole	2.30 (0.33)	2.23 (0.32)	2.31 (0.35)	2.27 (0.31)	14.26	14.30	15.14	13.63	2.56 (0.01)	-0.62 (0.54)	0.92 (0.36)	-2.93 (7.68)	0.55 (7.27)	-1.10 (8.61)	6.43 (5.05)	6.07 (3.93)	6.50 (5.67)	0.86	0.88	0.82	0.85 (0.72-0.92)	0.88 (0.79-0.93)	0.82 (0.69-0.90)
Left transverse temporal gyrus	1.04 (0.17)	1.03 (0.18)	1.04 (0.18)	1.03 (0.17)	16.86	17.74	17.01	16.71	0.80 (0.43)	-0.35 (0.73)	0.22 (0.83)	-0.64 (4.21)	0.19 (4.38)	-0.09 (3.64)	3.07 (2.91)	3.58 (2.46)	2.85 (2.23)	0.97	0.97	0.98	0.97 (0.95-0.99)	0.97 (0.94-0.98)	0.98 (0.96-0.99)
Left insula	6.50 (0.76)	6.50 (0.77)	6.51 (0.72)	6.51 (0.76)	11.64	11.87	11.06	11.66	-0.17 (0.86)	-0.30 (0.77)	-0.39 (0.70)	0.10 (4.18)	0.23 (3.40)	0.24 (3.88)	2.78 (3.09)	2.36 (2.43)	2.69 (2.78)	0.93	0.95	0.94	0.93 (0.87-0.96)	0.95 (0.91-0.97)	0.94 (0.89-0.97)
Right banks superior temporal sulcus	1.86 (0.34)	1.84 (0.37)	1.83 (0.31)	1.86 (0.35)	18.28	19.97	16.81	19.08	1.03 (0.31)	2.04 (0.05)	-0.15 (0.88)	-1.21 (6.18)	-1.18 (4.90)	0.11 (5.99)	4.37 (4.48)	3.69 (3.39)	4.27 (4.14)	0.95	0.97	0.95	0.94 (0.90-0.97)	0.96 (0.93-0.98)	0.95 (0.90-0.97)
Right caudal anterior cingulate cortex	1.81 (0.38)	1.82 (0.39)	1.80 (0.38)	1.79 (0.37)	21.17	21.62	21.00	20.92	-0.92 (0.36)	0.96 (0.34)	1.23 (0.23)	0.53 (5.07)	-0.46 (3.35)	-0.95 (5.62)	3.63 (3.54)	2.59 (2.14)	3.89 (4.12)	0.98	0.99	0.97	0.98 (0.96-0.99)	0.99 (0.98-0.99)	0.97 (0.94-0.98)
Right caudal middle frontal gyrus	5.12 (0.95)	5.08 (0.99)	5.12 (0.98)	5.14 (0.96)	18.58	19.39	19.12	18.67	1.84 (0.07)	-0.04 (0.97)	-0.97 (0.34)	-0.90 (3.14)	-0.08 (2.53)	0.40 (2.78)	1.96 (2.59)	1.94 (1.60)	2.12 (1.82)	0.99	0.99	0.99	0.99 (0.98-0.99)	0.99 (0.99-1.00)	0.99 (0.98-0.99)
Right cuneus	3.03 (0.60)	3.10 (0.62)	3.05 (0.55)	3.07 (0.59)	19.73	20.06	17.98	19.31	-1.50 (0.14)	-0.51 (0.61)	-1.55 (0.13)	2.24 (9.70)	0.78 (6.60)	1.36 (6.01)	3.88 (9.15)	3.45 (5.65)	3.14 (5.29)	0.87	0.95	0.96	0.87 (0.77-0.93)	0.95 (0.91-0.97)	0.96 (0.93-0.98)
Right entorhinal cortex	1.67 (0.44)	1.65 (0.43)	1.65 (0.46)	1.72 (0.44)	26.18	26.17	27.94	25.76	0.66 (0.51)	0.67 (0.50)	-1.52 (0.14)	-1.03 (11.77)	-1.82 (14.94)	3.26 (15.98)	8.52 (8.08)	10.06 (11.09)	10.36 (12.51)	0.92	0.87	0.86	0.92 (0.85-0.95)	0.87 (0.78-0.93)	0.86 (0.75-0.92)
Right fusiform gyrus	7.96 (1.14)	7.96 (1.15)	7.98 (1.09)	7.98 (1.13)	14.32	14.49	13.69	14.15	0.20 (0.84)	-0.54 (0.59)	-0.74 (0.47)	-0.07 (2.56)	0.31 (2.60)	0.32 (2.74)	1.84 (1.76)	1.94 (1.74)	2.14 (1.71)	0.99	0.99	0.98	0.99 (0.98-0.99)	0.99 (0.97-0.99)	0.99 (0.97-0.99)
Right inferior parietal cortex	11.46 (2.10)	11.37 (2.20)	11.39 (2.06)	11.48 (2.15)	18.32	19.36	18.05	18.70	1.09 (0.28)	1.41 (0.17)	-0.48 (0.64)	-1.01 (5.77)	-0.57 (2.62)	0.13 (2.74)	2.67 (5.21)	1.90 (1.87)	1.80 (2.05)	0.97	0.99	0.99	0.97 (0.94-0.98)	0.99 (0.98-0.99)	0.99 (0.98-0.99)
Right inferior temporal gyrus	9.04 (1.67)	9.00 (1.63)	9.04 (1.63)	9.05 (1.64)	18.50	18.13	18.00	18.08	1.06 (0.30)	0.16 (0.88)	-0.25 (0.80)	-0.39 (3.09)	-0.02 (3.44)	0.14 (3.03)	2.15 (2.23)	2.25 (2.58)	2.22 (2.04)	0.99	0.99	0.99	0.99 (0.98-0.99)	0.99 (0.97-0.99)	0.99 (0.98-0.99)
Right isthmus cingulate cortex	2.07 (0.41)	2.05 (0.41)	2.05 (0.44)	2.06 (0.44)	19.95	19.83	21.56	21.59	1.24 (0.22)	1.32 (0.19)	0.82 (0.42)	-1.04 (5.61)	-1.62 (6.91)	-1.09 (6.48)	4.13 (3.88)	4.75 (5.23)	4.52 (4.72)	0.96	0.96	0.96	0.96 (0.93-0.98)	0.96 (0.93-0.98)	0.96 (0.93-0.98)
Right lateral occipital cortex	10.87 (1.60)	10.85 (1.75)	10.87 (1.59)	10.95 (1.68)	14.69	16.14	14.64	15.34	0.37 (0.71)	0.17 (0.86)	-1.96 (0.06)	-0.48 (5.13)	-0.07 (3.11)	0.57 (2.24)	2.23 (4.63)	2.06 (2.32)	1.62 (1.63)	0.96	0.98	0.99	0.96 (0.93-0.98)	0.98 (0.96-0.99)	0.99 (0.98-0.99)
Right lateral orbitofrontal cortex	6.52 (0.72)	6.56 (0.76)	6.54 (0.72)	6.56 (0.73)	11.09	11.52	10.98	11.18	-1.08 (0.29)	-0.67 (0.50)	-1.17 (0.25)	0.57 (3.58)	0.34 (2.97)	0.62 (3.54)	2.69 (2.39)	2.19 (2.00)	2.82 (2.18)	0.95	0.96	0.95	0.95 (0.90-0.97)	0.96 (0.93-0.98)	0.95 (0.91-0.97)
Right lingual gyrus	6.59 (1.11)	6.60 (1.09)	6.59 (1.09)	6.59 (1.10)	16.90	16.45	16.57	16.62	-0.11 (0.91)	0.13 (0.89)	0.11 (0.92)	0.14 (3.65)	-0.01 (2.21)	0.04 (1.94)	2.07 (2.99)	1.64 (1.46)	1.47 (1.25)	0.98	0.99	0.99	0.98 (0.96-0.99)	0.99 (0.98-1.00)	0.99 (0.99-1.00)
Right medial orbitofrontal cortex	4.74 (0.52)	4.72 (0.50)	4.71 (0.50)	4.74 (0.50)	11.04	10.67	10.70	10.64	0.56 (0.58)	1.05 (0.30)	0.00 (1.00)	-0.29 (3.83)	-0.51 (3.32)	0.04 (3.85)	2.93 (2.44)	2.64 (2.04)	3.08 (2.27)	0.94	0.95	0.94	0.94 (0.89-0.96)	0.95 (0.92-0.97)	0.94 (0.89-0.97)
Right middle temporal gyrus	10.18 (1.51)	10.15 (1.57)	10.19 (1.53)	10.18 (1.55)	14.82	15.50	14.98	15.20	0.61 (0.55)	-0.71 (0.48)	-0.01 (1.00)	-0.39 (3.43)	0.13 (1.43)	-0.08 (2.26)	1.74 (2.96)	1.08 (0.93)	1.70 (1.47)	0.98	1.00	0.99	0.98 (0.97-0.99)	1.00 (0.99-1.00)	0.99 (0.98-0.99)
Right parahippocampal gyrus	1.74 (0.31)	1.74 (0.29)	1.75 (0.31)	1.75 (0.30)	17.85	16.83	17.88	16.94	0.04 (0.97)	-0.36 (0.72)	-0.56 (0.58)	0.18 (6.61)	0.28 (4.61)	0.66 (5.61)	4.37 (4.92)	3.34 (3.14)	3.94 (4.01)	0.94	0.97	0.96	0.94 (0.90-0.97)	0.97 (0.95-0.98)	0.96 (0.93-0.98)
Right paracentral lobule	3.62 (0.51)	3.62 (0.52)	3.62 (0.52)	3.62 (0.54)	14.17	14.35	14.49	14.81	0.18 (0.85)	-0.01 (0.99)	-0.33 (0.75)	-0.11 (2.70)	-0.05 (2.27)	0.03 (2.32)	2.20 (1.53)	1.69 (1.50)	1.84 (1.38)	0.98	0.99	0.99	0.98 (0.97-0.99)	0.99 (0.98-0.99)	0.99 (0.98-0.99)
Right pars opercularis	3.32 (0.54)	3.34 (0.58)	3.34 (0.55)	3.35 (0.56)	16.17	17.33	16.44	16.68	-1.07 (0.29)	-1.39 (0.17)	-1.43 (0.16)	0.41 (3.60)	0.48 (2.76)	0.66 (3.41)	2.72 (2.37)	2.06 (1.87)	2.69 (2.17)	0.98	0.99	0.98	0.98 (0.96-0.99)	0.99 (0.98-0.99)	0.98 (0.96-0.99)
Right pars orbitalis	2.40 (0.38)	2.40 (0.38)	2.40 (0.36)	2.40 (0.37)	15.68	15.94	15.19	15.50	0.62 (0.54)	0.00 (1.00)	-0.02 (0.98)	-0.29 (2.83)	0.08 (2.88)	0.04 (3.32)	2.08 (1.91)	2.33 (1.65)	2.61 (2.02)	0.99	0.98	0.98	0.99 (0.97-0.99)	0.98 (0.97-0.99)	0.98 (0.96-0.99)

Right pars triangularis	3.63 (0.60)	3.62 (0.60)	3.62 (0.57)	3.63 (0.59)	16.47	16.44	15.89	16.16	0.38 (0.70)	0.81 (0.42)	0.11 (0.91)	-0.17 (2.78)	-0.26 (3.01)	0.00 (2.53)	2.15 (1.73)	2.29 (1.95)	1.96 (1.57)	0.99	0.98	0.99	0.99 (0.97-0.99)	0.98 (0.97-0.99)	0.99 (0.98-0.99)
Right pericalcarine cortex	2.24 (0.42)	2.19 (0.41)	2.19 (0.39)	2.20 (0.38)	18.72	18.54	17.81	17.39	1.55 (0.13)	2.12 (0.04)	1.51 (0.14)	-2.05 (8.88)	-2.01 (6.23)	-1.38 (6.63)	3.93 (8.21)	3.92 (5.21)	3.67 (5.67)	0.89	0.93	0.93	0.88 (0.79-0.93)	0.93 (0.86-0.96)	0.92 (0.86-0.96)
Right postcentral gyrus	8.19 (1.15)	8.17 (1.17)	8.15 (1.17)	8.18 (1.15)	14.01	14.36	14.36	14.01	0.55 (0.59)	1.99 (0.05)	0.35 (0.72)	-0.21 (1.99)	-0.51 (1.56)	-0.10 (1.65)	1.22 (1.57)	1.30 (0.99)	1.30 (0.99)	0.99	0.99	0.99	0.99 (0.98-1.00)	0.99 (0.99-1.00)	0.99 (0.99-1.00)
Right posterior cingulate cortex	2.66 (0.59)	2.69 (0.59)	2.66 (0.61)	2.66 (0.61)	22.31	21.83	22.77	22.85	-0.91 (0.37)	-0.32 (0.75)	-0.10 (0.92)	1.15 (8.62)	0.04 (6.48)	-0.05 (10.29)	5.24 (6.90)	3.62 (5.35)	5.73 (8.50)	0.94	0.97	0.92	0.94 (0.89-0.97)	0.97 (0.94-0.98)	0.92 (0.86-0.96)
Right precentral gyrus	12.17 (1.58)	12.18 (1.55)	12.12 (1.54)	12.16 (1.59)	12.95	12.70	12.70	13.07	-0.17 (0.87)	1.77 (0.08)	0.39 (0.69)	0.07 (1.83)	-0.45 (1.68)	-0.12 (1.62)	1.57 (0.91)	1.43 (0.97)	1.25 (1.02)	0.99	0.99	0.99	0.99 (0.98-0.99)	0.99 (0.98-0.99)	0.99 (0.99-1.00)
Right precuneus	8.59 (1.21)	8.56 (1.21)	8.55 (1.22)	8.56 (1.24)	14.04	14.15	14.28	14.53	1.58 (0.12)	2.37 (0.02)	1.75 (0.09)	-0.44 (1.75)	-0.59 (1.53)	-0.49 (1.52)	1.25 (1.29)	1.24 (1.06)	1.25 (0.98)	0.99	0.99	0.99	0.99 (0.98-1.00)	0.99 (0.99-1.00)	0.99 (0.99-1.00)
Right rostral anterior cingulate cortex	1.86 (0.44)	1.86 (0.44)	1.86 (0.45)	1.87 (0.44)	23.47	23.81	24.24	23.49	-0.19 (0.85)	0.42 (0.67)	-0.36 (0.72)	-0.05 (4.15)	-0.52 (4.66)	0.16 (4.02)	3.41 (2.30)	3.58 (2.97)	3.00 (2.64)	0.98	0.98	0.99	0.98 (0.97-0.99)	0.98 (0.96-0.99)	0.99 (0.97-0.99)
Right rostral middle frontal gyrus	13.51 (1.63)	13.52 (1.69)	13.54 (1.63)	13.54 (1.64)	12.04	12.48	12.01	12.11	-0.15 (0.88)	-1.00 (0.32)	-0.95 (0.35)	0.00 (2.30)	0.25 (1.65)	0.23 (1.82)	1.58 (1.65)	1.36 (0.95)	1.42 (1.15)	0.98	0.99	0.99	0.98 (0.97-0.99)	0.99 (0.98-1.00)	0.99 (0.98-0.99)
Right superior frontal gyrus	19.01 (2.74)	18.91 (2.71)	19.01 (2.73)	19.01 (2.76)	14.42	14.32	14.36	14.54	1.30 (0.20)	0.04 (0.97)	0.06 (0.95)	-0.51 (2.66)	0.00 (1.54)	-0.04 (1.76)	1.55 (2.21)	1.04 (1.13)	1.31 (1.15)	0.98	0.99	0.99	0.98 (0.97-0.99)	0.99 (0.99-1.00)	0.99 (0.99-1.00)
Right superior parietal cortex	11.24 (1.67)	11.17 (1.70)	11.24 (1.67)	11.28 (1.68)	14.84	15.19	14.85	14.91	1.57 (0.12)	0.04 (0.97)	-1.79 (0.08)	-0.65 (2.95)	-0.03 (2.30)	0.33 (1.14)	1.58 (2.57)	1.51 (1.71)	0.96 (0.68)	0.99	0.99	1.00	0.99 (0.97-0.99)	0.99 (0.98-0.99)	1.00 (0.99-1.00)
Right superior temporal gyrus	10.11 (1.18)	10.07 (1.23)	10.13 (1.20)	10.07 (1.22)	11.64	12.18	11.87	12.12	1.20 (0.24)	-0.43 (0.67)	1.18 (0.25)	-0.49 (2.37)	0.13 (2.27)	-0.45 (2.36)	1.72 (1.68)	1.81 (1.34)	1.64 (1.74)	0.98	0.98	0.98	0.98 (0.96-0.99)	0.98 (0.97-0.99)	0.98 (0.97-0.99)
Right supramarginal gyrus	8.76 (1.18)	8.73 (1.20)	8.73 (1.18)	8.77 (1.17)	13.53	13.72	13.54	13.32	0.59 (0.56)	1.05 (0.30)	-0.72 (0.48)	-0.30 (3.24)	-0.26 (1.69)	0.23 (1.82)	2.05 (2.51)	1.32 (1.07)	1.47 (1.08)	0.97	0.99	0.99	0.97 (0.95-0.99)	0.99 (0.99-1.00)	0.99 (0.98-0.99)
Right frontal pole	1.03 (0.13)	1.03 (0.11)	1.06 (0.15)	1.02 (0.13)	12.66	10.94	14.02	13.03	0.21 (0.84)	-1.76 (0.08)	1.67 (0.10)	-0.03 (7.67)	1.97 (9.00)	-1.52 (6.12)	5.79 (4.95)	6.30 (6.65)	4.86 (3.95)	0.81	0.82	0.89	0.80 (0.67-0.89)	0.81 (0.67-0.89)	0.89 (0.81-0.94)
Right temporal pole	2.34 (0.30)	2.30 (0.31)	2.29 (0.34)	2.36 (0.31)	12.88	13.39	14.74	13.03	1.76 (0.09)	1.54 (0.13)	-1.10 (0.28)	-1.80 (6.69)	-2.26 (8.51)	0.91 (5.86)	5.10 (4.62)	6.75 (5.56)	4.80 (3.40)	0.88	0.82	0.91	0.88 (0.79-0.93)	0.81 (0.67-0.89)	0.90 (0.83-0.95)
Right transverse temporal gyrus	0.82 (0.16)	0.81 (0.15)	0.82 (0.16)	0.83 (0.16)	19.75	18.47	18.95	19.06	1.73 (0.09)	0.89 (0.38)	-0.51 (0.61)	-0.99 (4.76)	-0.40 (3.81)	0.43 (3.71)	3.86 (2.89)	3.36 (1.77)	3.16 (1.92)	0.97	0.98	0.98	0.97 (0.94-0.98)	0.98 (0.97-0.99)	0.98 (0.97-0.99)
Right insula	6.58 (0.73)	6.51 (0.80)	6.53 (0.77)	6.56 (0.83)	11.16	12.34	11.81	12.60	1.42 (0.16)	0.99 (0.33)	0.40 (0.69)	-1.11 (4.36)	-0.76 (4.32)	-0.47 (4.95)	3.29 (3.03)	3.34 (2.80)	3.99 (2.91)	0.93	0.92	0.92	0.93 (0.87-0.96)	0.92 (0.86-0.96)	0.92 (0.85-0.95)
Local cortical surface (cm²)																							
Left banks superior temporal sulcus	8.45 (1.30)	8.38 (1.25)	8.41 (1.26)	8.49 (1.33)	15.34	14.96	14.96	15.70	1.12 (0.27)	0.86 (0.39)	-0.86 (0.39)	-0.83 (5.55)	-0.42 (4.10)	0.41 (4.00)	3.51 (4.35)	2.66 (3.12)	2.67 (2.98)	0.94	0.97	0.97	0.94 (0.90-0.97)	0.97 (0.95-0.99)	0.97 (0.95-0.98)
Left caudal anterior cingulate cortex	5.63 (1.32)	5.72 (1.20)	5.64 (1.23)	5.75 (1.24)	23.48	21.04	21.74	21.51	-1.30 (0.20)	-0.09 (0.93)	-1.68 (0.10)	2.32 (11.49)	0.83 (11.66)	2.70 (11.23)	6.38 (9.79)	5.58 (10.24)	5.53 (10.12)	0.94	0.94	0.94	0.94 (0.89-0.97)	0.94 (0.89-0.97)	0.94 (0.89-0.97)
Left caudal middle frontal gyrus	19.79 (3.30)	19.74 (3.39)	19.74 (3.32)	19.79 (3.39)	16.66	17.19	16.85	17.13	0.81 (0.42)	0.95 (0.35)	-0.02 (0.98)	-0.33 (2.05)	-0.31 (2.09)	-0.06 (2.16)	1.50 (1.42)	1.45 (1.51)	1.72 (1.28)	0.99	0.99	0.99	0.99 (0.99-1.00)	0.99 (0.99-1.00)	0.99 (0.99-1.00)
Left cuneus	14.27 (1.98)	14.27 (2.12)	14.41 (2.02)	14.31 (2.04)	13.88	14.83	14.04	14.24	0.05 (0.96)	-2.02 (0.05)	-0.88 (0.38)	-0.15 (3.36)	0.91 (3.19)	0.21 (1.95)	2.29 (2.44)	2.41 (2.25)	1.57 (1.16)	0.98	0.98	0.99	0.98 (0.96-0.99)	0.98 (0.95-0.99)	0.99 (0.98-1.00)
Left entorhinal cortex	4.02 (1.04)	4.08 (0.96)	4.18 (0.96)	4.11 (0.94)	25.96	23.63	22.89	22.98	-0.95 (0.35)	-2.10 (0.04)	-1.69 (0.10)	2.00 (10.97)	4.42 (13.20)	2.94 (9.76)	8.52 (7.08)	8.23 (11.17)	6.17 (8.07)	0.92	0.88	0.95	0.92 (0.85-0.95)	0.87 (0.76-0.93)	0.94 (0.89-0.97)
Left fusiform gyrus	28.13 (4.08)	28.34 (4.04)	28.12 (4.04)	28.29 (3.94)	14.49	14.27	14.37	13.93	-1.76 (0.09)	0.11 (0.91)	-1.59 (0.12)	0.82 (3.06)	-0.01 (1.94)	0.65 (2.49)	2.35 (2.10)	1.58 (1.10)	1.91 (1.71)	0.98	0.99	0.99	0.98 (0.96-0.99)	0.99 (0.98-1.00)	0.99 (0.97-0.99)
Left inferior parietal cortex	38.37 (6.77)	37.97 (6.78)	38.21 (6.78)	38.52 (6.69)	17.64	17.85	17.74	17.36	1.58 (0.12)	1.47 (0.15)	-1.25 (0.22)	-1.17 (5.18)	-0.44 (1.84)	0.44 (2.20)	2.26 (4.79)	1.55 (1.06)	1.77 (1.35)	0.97	0.99	0.99	0.97 (0.94-0.98)	0.99 (0.99-1.00)	0.99 (0.99-1.00)
Left inferior temporal gyrus	29.12 (4.54)	29.31 (4.40)	29.22 (4.53)	29.42 (4.39)	15.58	15.00	15.51	14.91	-1.71 (0.09)	-1.10 (0.28)	-3.15 (0.00)	0.78 (2.94)	0.37 (2.16)	1.14 (2.33)	1.85 (2.41)	1.72 (1.33)	1.95 (1.69)	0.99	0.99	0.99	0.99 (0.97-0.99)	0.99 (0.98-1.00)	0.99 (0.97-0.99)
Left isthmus cingulate cortex	9.41 (1.53)	9.46 (1.48)	9.41 (1.51)	9.48 (1.49)	16.23	15.67	16.05	15.76	-0.63 (0.53)	0.09 (0.93)	-1.06 (0.29)	0.61 (5.36)	-0.01 (3.89)	0.76 (4.43)	3.92 (3.67)	2.80 (2.66)	3.00 (3.32)	0.95	0.97	0.96	0.95 (0.91-0.97)	0.97 (0.95-0.99)	0.96 (0.93-0.98)
Left lateral occipital cortex	46.84 (5.62)	46.85 (6.13)	46.92 (5.49)	46.94 (5.71)	12.00	13.09	11.70	12.16	-0.05 (0.96)	-0.69 (0.50)	-0.84 (0.41)	-0.11 (4.09)	0.22 (1.72)	0.21 (1.85)	1.84 (3.64)	1.42 (0.96)	1.30 (1.32)	0.96	0.99	0.99	0.96 (0.93-0.98)	0.99 (0.98-0.99)	0.99 (0.98-0.99)
Left lateral orbitofrontal cortex	24.50 (3.06)	24.63 (3.12)	24.45 (3.14)	24.63 (3.13)	12.49	12.66	12.85	12.73	-1.84 (0.07)	0.86 (0.40)	-2.38 (0.02)	0.49 (1.88)	-0.27 (1.66)	0.50 (1.40)	1.37 (1.37)	1.29 (1.05)	1.15 (0.93)	0.99	0.99	0.99	0.99 (0.98-0.99)	0.99 (0.98-1.00)	0.99 (0.99-1.00)
Left lingual gyrus	29.60 (4.04)	29.57 (4.13)	29.68 (4.14)	29.62 (3.96)	13.66	13.97	13.93	13.36	0.27 (0.79)	-0.91 (0.37)	-0.23 (0.82)	-0.13 (2.06)	0.25 (1.94)	0.12 (1.97)	1.74 (1.07)	1.61 (1.09)	1.59 (1.13)	0.99	0.99	0.99	0.99 (0.98-0.99)	0.99 (0.98-0.99)	0.99 (0.98-0.99)
Left medial orbitofrontal cortex	18.55 (2.55)	18.46 (2.56)	18.31 (2.40)	18.58 (2.45)	13.76	13.87	13.09	13.17	0.43 (0.67)	1.43 (0.16)	-0.18 (0.86)	-0.51 (7.62)	-1.15 (6.00)	0.26 (6.94)	5.88 (4.80)	4.82 (3.68)	5.06 (4.70)	0.86	0.91	0.88	0.86 (0.75-0.92)	0.90 (0.83-0.95)	0.88 (0.79-0.93)
Left middle temporal gyrus	27.49 (4.08)	27.69 (4.13)	27.41 (4.14)	27.64 (4.10)	14.84	14.91	15.12	14.84	-2.29 (0.03)	1.18 (0.24)	-1.86 (0.07)	0.72 (2.22)	-0.36 (1.85)	0.54 (1.95)	1.75 (1.52)	1.44 (1.20)	1.58 (1.24)	0.99	0.99	0.99	0.99 (0.98-0.99)	0.99 (0.99-1.00)	0.99 (0.98-1.00)
Left parahippocampal gyrus	5.97 (0.83)	6.04 (0.80)	6.02 (0.78)	6.08 (0.90)	13.93	13.22	12.89	14.74	-2.00 (0.05)	-1.32 (0.19)	-2.73 (0.01)	1.28 (4.20)	0.91 (4.02)	1.74 (4.16)	3.34 (2.82)	3.04 (2.75)	3.43 (2.89)	0.96	0.96	0.96	0.95 (0.92-0.98)	0.96 (0.93-0.98)	0.95 (0.89-0.97)
Left paracentral lobule	12.93 (1.77)	12.88 (1.82)	12.94 (1.82)	12.89 (1.81)	13.65	14.16	14.08	14.05	1.31 (0.20)	-0.09 (0.93)	1.28 (0.21)	-0.48 (2.04)	0.00 (2.01)	-0.39 (1.77)	1.75 (1.13)	1.62 (1.16)	1.43 (1.09)	0.99	0.99	0.99	0.99 (0.98-0.99)	0.99 (0.98-0.99)	0.99 (0.98-1.00)
Left pars opercularis	14.13 (2.03)	14.30 (2.04)	14.17 (2.10)	14.21 (2.03)	14.35	14.29	14.81	14.29	-2.87 (0.01)	-0.66 (0.51)	-1.76 (0.09)	1.18 (2.53)	0.23 (3.03)	0.59 (2.06)	2.20 (1.68)	2.18 (2.09)	1.77 (1.17)	0.98	0.98	0.99	0.98 (0.96-0.99)	0.98 (0.96-0.99)	0.99 (0.98-0.99)
Left pars orbitalis	6.36 (1.03)	6.37 (1.07)	6.37 (1.03)	6.37 (1.03)	16.18	16.82	16.14	16.14	-0.30 (0.76)	-0.51 (0.61)	-0.44 (0.66)	0.05 (3.63)	0.24 (3.34)	0.22 (3.44)	2.89 (2.15)	2.48 (2.21)	2.66 (2.17)	0.98	0.98	0.98	0.98 (0.96-0.99)	0.98 (0.96-0.99)	0.98 (0.96-0.99)
Left pars triangularis	12.11 (1.78)	12.11 (1.79)	12.09 (1.81)	12.13 (1.78)	14.69	14.81	14.96	14.64	0.19 (0.85)	0.48 (0.63)	-0.20 (0.84)	-0.08 (2.28)	-0.25 (2.83)	0.10 (3.00)	1.91 (1.22)	2.20 (1.76)	2.37 (1.80)	0.99	0.98	0.98	0.99 (0.98-0.99)	0.98 (0.96-0.99)	0.98 (0.96-0.99)
Left pericalcarine cortex	13.58 (2.08)	13.65 (2.07)	13.56 (2.17)	13.54 (2.23)	15.29	15.16	15.99	16.45	-0.79 (0.43)	0.43 (0.67)	0.62 (0.54)	0.51 (4.15)	-0.30 (3.15)	-0.49 (3.32)	2.49 (3.34)	2.18 (2.26)	2.33 (2.39)	0.96	0.98	0.98	0.97 (0.94-0.98)	0.98 (0.97-0.99)	0.98 (0.97-0.99)
Left postcentral gyrus	38.75 (4.81)	38.65 (4.85)	38.66 (4.90)	38.78 (4.84)	12.42	12.56	12.69	12.47	1.40 (0.17)	1.59 (0.12)	-0.37 (0.71)	-0.29 (1.31)	-0.29 (1.06)	0.06 (1.28)	0.93 (0.95)	0.90 (0.62)	0.93 (0.88)	0.99	1.00	1.00	0.99 (0.99-1.00)	1.00 (0.99-1.00)	1.00 (0.99-1.00)
Left posterior cingulate cortex	10.42 (1.79)	10.38 (1.69)	10.43 (1.75)	10.56 (1.80)	17.18	16.25	16.81	17.05	0.52 (0.61)	-0.10 (0.92)	-2.26 (0.03)	-0.16 (4.78)	0.26 (7.77)	1.40 (3.91)	3.14 (3.58)	3.88 (6.71)	3.17 (2.64)	0.96	0.93	0.97	0.96 (0.93-0.98)	0.94 (0.88-0.96)	0.97 (0.95-0.98)

Left precentral gyrus	46.24 (5.80)	46.22 (6.05)	46.30 (6.11)	46.23 (6.05)	12.54	13.09	13.21	13.09	0.13 (0.90)	-0.43 (0.67)	0.11 (0.91)	-0.11 (1.96)	0.05 (2.04)	-0.09 (1.24)	1.05 (1.65)	1.09 (1.71)	0.98 (0.75)	0.99	0.99	1.00	0.99 (0.98-0.99)	0.99 (0.98-0.99)	1.00 (0.99-1.00)
Left precuneus	34.36 (4.90)	34.23 (4.94)	34.32 (4.85)	34.35 (4.88)	14.26	14.44	14.13	14.22	1.06 (0.30)	0.64 (0.52)	0.20 (0.84)	-0.43 (2.73)	-0.10 (1.37)	-0.03 (1.31)	1.51 (2.31)	1.07 (0.86)	1.08 (0.74)	0.99	1.00	1.00	0.99 (0.97-0.99)	1.00 (0.99-1.00)	1.00 (0.99-1.00)
Left rostral anterior cingulate cortex	8.14 (2.02)	8.10 (1.80)	8.18 (2.00)	8.20 (2.03)	24.76	22.21	24.42	24.71	0.54 (0.59)	-0.76 (0.45)	-0.83 (0.41)	0.19 (6.11)	0.72 (4.92)	0.99 (6.15)	4.33 (4.27)	3.68 (3.30)	4.51 (4.24)	0.97	0.98	0.97	0.97 (0.94-0.98)	0.98 (0.97-0.99)	0.97 (0.95-0.98)
Left rostral middle frontal gyrus	50.90 (8.25)	50.94 (8.28)	50.83 (8.03)	51.23 (7.96)	16.21	16.25	15.80	15.53	-0.27 (0.78)	0.52 (0.61)	-2.31 (0.03)	0.08 (1.93)	-0.07 (1.83)	0.76 (1.99)	1.49 (1.21)	1.42 (1.14)	1.66 (1.32)	0.99	0.99	0.99	0.99 (0.99-1.00)	0.99 (0.99-1.00)	0.99 (0.99-1.00)
Left superior frontal gyrus	67.29 (10.69)	67.31 (10.74)	67.43 (10.67)	67.46 (10.69)	15.89	15.96	15.82	15.85	-0.13 (0.89)	-1.39 (0.17)	-1.45 (0.15)	0.01 (1.09)	0.22 (1.00)	0.26 (1.09)	0.86 (0.65)	0.80 (0.63)	0.84 (0.73)	1.00	1.00	1.00	1.00 (1.00-1.00)	1.00 (1.00-1.00)	1.00 (1.00-1.00)
Left superior parietal cortex	49.15 (7.13)	48.92 (7.59)	49.05 (7.11)	49.16 (7.02)	14.50	15.51	14.50	14.27	1.05 (0.30)	1.07 (0.29)	-0.12 (0.91)	-0.65 (3.98)	-0.18 (1.18)	0.06 (1.24)	1.43 (3.77)	1.03 (0.59)	0.94 (0.79)	0.98	1.00	1.00	0.98 (0.97-0.99)	1.00 (0.99-1.00)	1.00 (0.99-1.00)
Left superior temporal gyrus	36.75 (4.08)	36.80 (4.21)	36.72 (4.24)	36.84 (4.20)	11.11	11.45	11.54	11.40	-0.64 (0.53)	0.47 (0.64)	-1.18 (0.24)	0.10 (1.50)	-0.15 (1.42)	0.22 (1.42)	1.05 (1.06)	1.04 (0.97)	1.13 (0.87)	0.99	0.99	0.99	0.99 (0.99-1.00)	0.99 (0.99-1.00)	0.99 (0.99-1.00)
Left supramarginal gyrus	36.71 (5.89)	36.75 (6.40)	36.74 (6.02)	36.84 (6.02)	16.05	17.42	16.38	16.35	-0.14 (0.89)	-0.35 (0.73)	-1.58 (0.12)	-0.09 (3.60)	0.03 (1.63)	0.31 (1.48)	1.92 (3.03)	1.23 (1.06)	1.17 (0.95)	0.98	1.00	1.00	0.97 (0.95-0.99)	1.00 (0.99-1.00)	1.00 (0.99-1.00)
Left frontal pole	2.44 (0.30)	2.42 (0.31)	2.43 (0.28)	2.42 (0.30)	12.37	12.99	11.53	12.31	0.70 (0.49)	0.37 (0.71)	0.82 (0.42)	-0.92 (7.97)	-0.32 (7.37)	-0.75 (5.97)	6.51 (4.59)	5.71 (4.58)	4.89 (3.42)	0.81	0.82	0.88	0.81 (0.67-0.89)	0.82 (0.69-0.90)	0.88 (0.80-0.94)
Left temporal pole	4.40 (0.59)	4.32 (0.55)	4.43 (0.62)	4.38 (0.58)	13.48	12.61	13.97	13.32	1.85 (0.07)	-0.78 (0.44)	0.28 (0.78)	-1.54 (5.84)	0.68 (6.10)	-0.22 (5.88)	4.86 (3.50)	4.96 (3.53)	4.70 (3.47)	0.90	0.90	0.91	0.89 (0.81-0.94)	0.90 (0.83-0.95)	0.91 (0.84-0.95)
Left transverse temporal gyrus	4.22 (0.64)	4.25 (0.68)	4.24 (0.65)	4.22 (0.64)	15.13	15.96	15.28	15.10	-1.10 (0.28)	-0.57 (0.57)	-0.24 (0.81)	0.58 (4.18)	0.38 (4.62)	0.17 (3.94)	3.26 (2.64)	3.57 (2.90)	3.03 (2.48)	0.97	0.95	0.96	0.96 (0.93-0.98)	0.95 (0.91-0.97)	0.96 (0.93-0.98)
Left insula	23.45 (2.75)	23.60 (2.66)	23.48 (2.68)	23.55 (2.63)	11.74	11.27	11.39	11.18	-0.59 (0.56)	-0.12 (0.90)	-0.38 (0.71)	0.65 (6.40)	0.16 (6.37)	0.45 (6.85)	4.72 (4.31)	4.38 (4.58)	4.90 (4.74)	0.83	0.85	0.81	0.83 (0.71-0.90)	0.85 (0.74-0.92)	0.81 (0.67-0.89)
Right banks superior temporal sulcus	7.83 (1.19)	7.80 (1.32)	7.76 (1.05)	7.88 (1.20)	15.26	16.88	13.56	15.24	0.45 (0.66)	1.75 (0.09)	-0.84 (0.40)	-0.52 (5.20)	-0.68 (3.67)	0.64 (4.28)	3.80 (3.53)	2.70 (2.55)	2.99 (3.09)	0.94	0.98	0.96	0.94 (0.89-0.97)	0.97 (0.94-0.98)	0.96 (0.92-0.98)
Right caudal anterior cingulate cortex	6.65 (1.37)	6.72 (1.43)	6.65 (1.32)	6.62 (1.33)	20.61	21.23	19.81	20.04	-1.49 (0.14)	-0.03 (0.98)	0.63 (0.53)	1.00 (5.08)	0.16 (3.59)	-0.38 (4.83)	4.04 (3.19)	2.74 (2.29)	3.72 (3.06)	0.97	0.98	0.97	0.97 (0.95-0.98)	0.98 (0.97-0.99)	0.97 (0.94-0.98)
Right caudal middle frontal gyrus	19.27 (3.59)	19.20 (3.71)	19.33 (3.57)	19.33 (3.67)	18.65	19.33	18.49	18.98	1.02 (0.31)	-0.84 (0.41)	-0.86 (0.40)	-0.53 (2.81)	0.30 (2.36)	0.25 (2.49)	2.13 (1.88)	1.77 (1.57)	1.85 (1.67)	0.99	0.99	0.99	0.99 (0.98-0.99)	0.99 (0.99-1.00)	0.99 (0.99-1.00)
Right cuneus	15.35 (2.28)	15.47 (2.30)	15.33 (2.13)	15.47 (2.30)	14.85	14.90	13.92	14.85	-1.45 (0.15)	0.21 (0.83)	-1.15 (0.26)	0.72 (3.50)	-0.07 (4.85)	0.78 (4.72)	1.96 (2.97)	2.82 (3.92)	2.74 (3.90)	0.97	0.94	0.95	0.97 (0.95-0.99)	0.94 (0.89-0.97)	0.95 (0.92-0.97)
Right entorhinal cortex	3.48 (0.70)	3.52 (0.73)	3.53 (0.73)	3.57 (0.79)	20.01	20.83	20.80	22.22	-0.93 (0.36)	-0.88 (0.38)	-1.55 (0.13)	0.90 (7.99)	1.27 (10.03)	2.11 (10.66)	6.32 (4.88)	7.88 (6.21)	7.63 (7.65)	0.93	0.87	0.88	0.93 (0.88-0.96)	0.87 (0.78-0.93)	0.87 (0.77-0.93)
Right fusiform gyrus	27.02 (3.53)	27.08 (3.63)	27.04 (3.58)	27.06 (3.56)	13.08	13.41	13.23	13.14	-0.68 (0.50)	-0.23 (0.82)	-0.35 (0.73)	0.17 (2.01)	0.05 (1.94)	0.13 (2.36)	1.44 (1.40)	1.54 (1.16)	1.90 (1.38)	0.99	0.99	0.98	0.99 (0.98-0.99)	0.99 (0.98-0.99)	0.98 (0.97-0.99)
Right inferior parietal cortex	44.79 (7.25)	44.50 (7.73)	44.64 (7.05)	45.03 (7.31)	16.19	17.38	15.80	16.23	1.04 (0.30)	1.25 (0.22)	-1.82 (0.08)	-0.86 (5.12)	-0.27 (1.77)	0.53 (1.92)	2.08 (4.75)	1.35 (1.16)	1.41 (1.39)	0.97	0.99	0.99	0.97 (0.95-0.98)	0.99 (0.99-1.00)	0.99 (0.99-1.00)
Right inferior temporal gyrus	28.38 (4.97)	28.34 (4.82)	28.42 (4.71)	28.45 (4.84)	17.52	17.00	16.56	17.00	0.32 (0.75)	-0.25 (0.80)	-0.52 (0.61)	-0.09 (2.81)	0.24 (3.15)	0.30 (3.06)	2.05 (1.89)	2.23 (2.22)	2.41 (1.87)	0.99	0.98	0.98	0.99 (0.97-0.99)	0.98 (0.97-0.99)	0.98 (0.97-0.99)
Right isthmus cingulate cortex	8.32 (1.45)	8.34 (1.43)	8.31 (1.55)	8.36 (1.55)	17.41	17.13	18.67	18.53	-0.39 (0.70)	0.07 (0.94)	-0.56 (0.58)	0.29 (4.49)	-0.30 (6.65)	0.28 (6.22)	3.44 (2.85)	4.40 (4.95)	4.72 (3.99)	0.97	0.95	0.95	0.97 (0.95-0.98)	0.95 (0.91-0.97)	0.95 (0.91-0.97)
Right lateral occipital cortex	46.03 (5.44)	45.80 (6.10)	46.11 (5.47)	46.27 (5.64)	11.83	13.32	11.87	12.19	0.73 (0.47)	-0.76 (0.45)	-2.35 (0.02)	-0.72 (5.65)	0.18 (1.65)	0.47 (1.50)	2.04 (5.30)	1.21 (1.12)	1.20 (1.00)	0.94	0.99	0.99	0.94 (0.89-0.97)	0.99 (0.98-0.99)	0.99 (0.98-1.00)
Right lateral orbitofrontal cortex	24.57 (3.01)	25.16 (3.30)	25.01 (3.11)	24.85 (3.14)	12.23	13.13	12.45	12.62	-3.15 (0.00)	-2.28 (0.03)	-1.26 (0.22)	2.24 (4.63)	1.74 (4.82)	1.11 (6.00)	3.99 (3.21)	3.92 (3.26)	4.49 (4.07)	0.93	0.92	0.89	0.91 (0.81-0.96)	0.91 (0.83-0.95)	0.89 (0.80-0.94)
Right lingual gyrus	30.74 (4.43)	30.76 (4.40)	30.76 (4.26)	30.75 (4.50)	14.40	14.32	13.85	14.63	-0.16 (0.88)	-0.24 (0.81)	-0.09 (0.93)	0.06 (2.26)	0.14 (2.02)	0.00 (1.95)	1.79 (1.36)	1.55 (1.28)	1.42 (1.31)	0.99	0.99	0.99	0.99 (0.98-0.99)	0.99 (0.98-0.99)	0.99 (0.98-0.99)
Right medial orbitofrontal cortex	18.57 (2.28)	18.63 (2.18)	18.56 (2.32)	18.54 (2.14)	12.26	11.69	12.50	11.55	-0.40 (0.69)	0.15 (0.88)	0.24 (0.81)	0.35 (4.47)	-0.12 (4.17)	-0.08 (4.81)	3.66 (2.54)	3.26 (2.55)	3.67 (3.06)	0.93	0.94	0.92	0.93 (0.88-0.96)	0.94 (0.90-0.97)	0.92 (0.86-0.96)
Right middle temporal gyrus	31.03 (4.35)	30.99 (4.37)	31.12 (4.36)	31.21 (4.35)	14.03	14.11	14.00	13.92	0.52 (0.60)	-1.11 (0.27)	-2.21 (0.03)	-0.17 (2.08)	0.29 (1.74)	0.57 (1.69)	1.17 (1.72)	1.47 (0.95)	1.26 (1.25)	0.99	0.99	0.99	0.99 (0.98-1.00)	0.99 (0.99-1.00)	0.99 (0.98-1.00)
Right parahippocampal gyrus	5.78 (0.72)	5.81 (0.70)	5.84 (0.68)	5.84 (0.73)	12.45	11.96	11.61	12.53	-0.83 (0.41)	-1.92 (0.06)	-1.66 (0.10)	0.73 (5.29)	1.25 (4.00)	1.15 (4.89)	3.82 (3.69)	3.35 (2.47)	3.84 (3.18)	0.90	0.95	0.93	0.90 (0.83-0.95)	0.95 (0.90-0.97)	0.93 (0.87-0.96)
Right paracentral lobule	14.62 (2.09)	14.61 (2.03)	14.60 (2.08)	14.66 (2.10)	14.29	13.89	14.26	14.33	0.24 (0.81)	0.50 (0.62)	-0.73 (0.47)	-0.03 (2.03)	-0.15 (2.03)	0.22 (2.03)	1.52 (1.33)	1.66 (1.15)	1.66 (1.17)	0.99	0.99	0.99	0.99 (0.98-0.99)	0.99 (0.98-1.00)	0.99 (0.98-0.99)
Right pars opercularis	12.18 (1.93)	12.24 (2.10)	12.18 (1.90)	12.18 (1.97)	15.83	17.13	15.64	16.20	-0.91 (0.37)	-0.02 (0.98)	-0.10 (0.92)	0.35 (3.64)	0.03 (2.98)	0.01 (3.18)	2.69 (2.44)	2.22 (1.95)	2.59 (1.80)	0.98	0.98	0.98	0.97 (0.95-0.99)	0.98 (0.97-0.99)	0.98 (0.97-0.99)
Right pars orbitalis	7.59 (1.13)	7.56 (1.12)	7.56 (1.09)	7.54 (1.10)	14.91	14.81	14.42	14.53	0.81 (0.42)	0.77 (0.45)	1.22 (0.23)	-0.32 (2.69)	-0.28 (2.93)	-0.50 (2.92)	2.09 (1.70)	2.42 (1.63)	2.47 (1.59)	0.98	0.98	0.98	0.98 (0.97-0.99)	0.98 (0.96-0.99)	0.98 (0.96-0.99)
Right pars triangularis	13.71 (2.23)	13.78 (2.30)	13.71 (2.21)	13.71 (2.26)	16.24	16.70	16.13	16.50	-1.41 (0.17)	0.02 (0.98)	0.00 (1.00)	0.40 (2.25)	0.01 (2.84)	-0.05 (2.30)	1.87 (1.29)	2.24 (1.72)	1.74 (1.48)	0.99	0.99	0.99	0.99 (0.98-0.99)	0.99 (0.97-0.99)	0.99 (0.98-0.99)
Right pericalcarine cortex	14.90 (2.56)	14.74 (2.65)	14.73 (2.56)	14.78 (2.57)	17.16	18.00	17.35	17.35	1.22 (0.23)	1.82 (0.08)	1.41 (0.16)	-1.27 (6.69)	-1.18 (4.23)	-0.83 (3.88)	3.54 (5.80)	2.89 (3.27)	2.50 (3.05)	0.94	0.97	0.98	0.94 (0.89-0.97)	0.97 (0.94-0.98)	0.98 (0.96-0.99)
Right postcentral gyrus	37.28 (4.98)	37.30 (5.00)	37.23 (4.95)	37.32 (4.90)	13.36	13.39	13.29	13.13	-0.37 (0.71)	0.62 (0.54)	-0.72 (0.48)	0.07 (1.33)	-0.12 (1.33)	0.15 (1.12)	1.07 (0.78)	0.98 (0.90)	0.82 (0.76)	1.00	1.00	1.00	1.00 (0.99-1.00)	1.00 (0.99-1.00)	1.00 (0.99-1.00)
Right posterior cingulate cortex	10.49 (2.24)	10.57 (2.24)	10.55 (2.23)	10.53 (2.27)	21.31	21.19	21.12	21.53	-0.82 (0.42)	-0.69 (0.49)	-0.30 (0.76)	0.77 (7.12)	0.46 (6.21)	0.25 (8.52)	4.96 (5.10)	4.06 (4.69)	4.93 (6.91)	0.96	0.97	0.94	0.96 (0.92-0.98)	0.97 (0.94-0.98)	0.94 (0.89-0.97)
Right precentral gyrus	45.84 (6.30)	45.79 (6.32)	45.64 (6.23)	45.71 (6.44)	13.74	13.80	13.66	14.09	0.60 (0.55)	1.84 (0.07)	0.98 (0.33)	-0.12 (1.18)	-0.42 (1.53)	-0.33 (1.87)	0.93 (0.72)	1.06 (1.17)	1.15 (1.51)	1.00	0.99	0.99	1.00 (0.99-1.00)	0.99 (0.99-1.00)	0.99 (0.98-0.99)
Right precuneus	36.25 (5.09)	36.28 (5.22)	36.16 (5.10)	36.27 (5.19)	14.05	14.39	14.10	14.32	-0.40 (0.69)	1.13 (0.27)	-0.29 (0.77)	0.06 (1.45)	-0.25 (1.33)	0.03 (1.38)	1.04 (1.00)	1.09 (0.80)	1.07 (0.86)	0.99	0.99	1.00	0.99 (0.99-1.00)	0.99 (0.99-1.00)	1.00 (0.99-1.00)
Right rostral anterior cingulate cortex	5.74 (1.33)	5.81 (1.46)	5.77 (1.42)	5.74 (1.34)	23.11	25.20	24.64	23.32	-1.23 (0.23)	-0.53 (0.60)	0.08 (0.93)	0.71 (5.83)	0.18 (6.62)	-0.13 (5.28)	4.68 (3.47)	5.03 (4.24)	3.96 (3.45)	0.97	0.96	0.97	0.97 (0.94-0.98)	0.96 (0.93-0.98)	0.97 (0.95-0.99)
Right rostral middle frontal gyrus	52.97 (7.97)	53.19 (8.01)	52.94 (7.92)	53.44 (7.85)	15.04	15.07	14.96	14.69	-1.67 (0.10)	0.27 (0.79)	-3.43 (0.00)	0.41 (1.60)	-0.05 (1.57)	0.92 (1.71)	1.35 (0.93)	1.20 (1.00)	1.60 (1.09)	0.99	0.99	0.99	0.99 (0.99-1.00)	1.00 (0.99-1.00)	0.99 (0.98-1.00)
Right superior frontal gyrus	65.53 (10.75)	65.53 (10.81)	65.59 (10.72)	65.62 (10.61)	16.40	16.50	16.34	16.17	0.01 (0.99)	-0.56 (0.58)	-0.74 (0.46)	-0.01 (1.05)	0.10 (1.12)	0.16 (1.18)	0.82 (0.65)	0.89 (0.67)	0.89 (0.77)	1.00	1.00	1.00	1.00 (1.00-1.00)	1.00 (1.00-1.00)	1.00 (1.00-1.00)

Right superior parietal cortex	48.46 (6.78)	48.22 (6.95)	48.44 (6.70)	48.63 (6.61)	13.99	14.42	13.82	13.60	1.86 (0.07)	0.16 (0.88)	-1.93 (0.06)	-0.55 (2.07)	0.00 (1.47)	0.41 (1.29)	1.05 (1.87)	1.11 (0.94)	1.04 (0.85)	0.99	0.99	1.00	0.99 (0.99-1.00)	0.99 (0.99-1.00)	1.00 (0.99-1.00)
Right superior temporal gyrus	34.21 (3.72)	34.20 (3.78)	34.31 (3.78)	34.15 (3.66)	10.88	11.05	11.01	10.71	0.11 (0.91)	-0.99 (0.33)	0.74 (0.46)	-0.05 (1.85)	0.28 (1.80)	-0.15 (1.48)	1.35 (1.24)	1.29 (1.27)	1.13 (0.95)	0.99	0.98	0.99	0.99 (0.97-0.99)	0.98 (0.97-0.99)	0.99 (0.98-1.00)
Right supramarginal gyrus	33.68 (4.96)	33.66 (4.96)	33.67 (4.97)	33.69 (4.90)	14.73	14.73	14.75	14.53	0.23 (0.82)	0.21 (0.83)	-0.16 (0.87)	-0.06 (1.45)	-0.04 (1.21)	0.05 (1.18)	1.15 (0.86)	0.93 (0.77)	0.95 (0.68)	0.99	1.00	1.00	0.99 (0.99-1.00)	1.00 (0.99-1.00)	1.00 (0.99-1.00)
Right frontal pole	2.93 (0.35)	2.94 (0.33)	2.99 (0.37)	2.91 (0.31)	11.85	11.35	12.21	10.70	-0.44 (0.66)	-2.47 (0.02)	0.53 (0.60)	0.50 (7.06)	2.07 (5.81)	-0.34 (6.23)	5.54 (4.33)	4.82 (3.80)	4.88 (3.83)	0.83	0.89	0.87	0.84 (0.72-0.91)	0.88 (0.78-0.94)	0.87 (0.77-0.93)
Right temporal pole	4.40 (0.69)	4.35 (0.70)	4.37 (0.69)	4.39 (0.69)	15.71	16.16	15.86	15.71	1.33 (0.19)	0.78 (0.44)	0.16 (0.88)	-1.32 (6.82)	-0.82 (6.79)	-0.18 (6.44)	4.86 (4.91)	5.32 (4.22)	4.98 (4.02)	0.92	0.91	0.93	0.92 (0.86-0.96)	0.91 (0.84-0.95)	0.93 (0.87-0.96)
Right transverse temporal gyrus	3.12 (0.45)	3.08 (0.42)	3.09 (0.45)	3.11 (0.45)	14.50	13.48	14.60	14.56	1.44 (0.16)	1.31 (0.20)	0.42 (0.67)	-0.91 (4.91)	-0.99 (4.59)	-0.30 (4.40)	3.84 (3.15)	3.77 (2.75)	3.65 (2.41)	0.94	0.95	0.95	0.94 (0.89-0.97)	0.94 (0.90-0.97)	0.95 (0.92-0.98)
Right insula	23.77 (2.58)	23.36 (3.22)	23.56 (2.91)	23.48 (3.23)	10.87	13.80	12.35	13.75	1.61 (0.12)	0.80 (0.43)	1.03 (0.31)	-2.08 (6.96)	-1.01 (6.76)	-1.55 (7.79)	5.51 (4.68)	5.39 (4.13)	6.14 (4.96)	0.86	0.83	0.83	0.83 (0.71-0.91)	0.82 (0.69-0.90)	0.81 (0.68-0.89)
Local cortical thickness (mm)																						
Left banks superior temporal sulcus	2.41 (0.17)	2.40 (0.18)	2.41 (0.17)	2.41 (0.18)	7.23	7.45	7.22	7.31	1.38 (0.17)	-0.18 (0.86)	0.97 (0.34)	-0.51 (2.27)	0.04 (1.32)	-0.24 (1.51)	1.65 (1.62)	0.98 (0.87)	1.15 (0.98)	0.95	0.98	0.98	0.95 (0.91-0.97)	0.98 (0.97-0.99)	0.98 (0.96-0.99)
Left caudal anterior cingulate cortex	2.64 (0.24)	2.63 (0.24)	2.65 (0.24)	2.62 (0.22)	9.22	9.24	8.97	8.45	0.20 (0.84)	-0.66 (0.52)	0.90 (0.37)	-0.13 (3.81)	0.42 (3.91)	-0.51 (4.07)	3.00 (2.30)	3.03 (2.46)	3.06 (2.69)	0.91	0.90	0.90	0.91 (0.84-0.95)	0.90 (0.83-0.95)	0.89 (0.81-0.94)
Left caudal middle frontal gyrus	2.41 (0.14)	2.41 (0.13)	2.41 (0.13)	2.40 (0.13)	5.77	5.43	5.58	5.56	0.18 (0.85)	0.37 (0.71)	0.68 (0.50)	-0.04 (1.89)	-0.09 (1.88)	-0.17 (1.76)	1.52 (1.10)	1.50 (1.10)	1.30 (1.17)	0.94	0.95	0.95	0.94 (0.90-0.97)	0.95 (0.90-0.97)	0.95 (0.91-0.97)
Left cuneus	1.81 (0.10)	1.83 (0.14)	1.82 (0.10)	1.82 (0.11)	5.73	7.55	5.73	6.17	-0.97 (0.34)	-0.50 (0.62)	-0.91 (0.37)	0.76 (5.16)	0.18 (2.25)	0.38 (2.86)	2.05 (4.79)	1.35 (1.80)	1.44 (2.49)	0.65	0.92	0.88	0.62 (0.40-0.77)	0.92 (0.86-0.96)	0.88 (0.79-0.93)
Left entorhinal cortex	3.17 (0.49)	3.15 (0.46)	3.16 (0.45)	3.18 (0.46)	15.48	14.58	14.25	14.35	0.62 (0.54)	0.46 (0.65)	-0.73 (0.47)	-0.27 (5.05)	-0.09 (4.63)	0.63 (3.96)	3.42 (3.70)	3.54 (2.93)	3.02 (2.59)	0.95	0.96	0.97	0.95 (0.91-0.97)	0.96 (0.92-0.98)	0.97 (0.94-0.98)
Left fusiform gyrus	2.61 (0.20)	2.60 (0.20)	2.60 (0.19)	2.60 (0.19)	7.49	7.65	7.29	7.29	1.88 (0.07)	1.32 (0.19)	1.67 (0.10)	-0.44 (1.51)	-0.30 (1.62)	-0.32 (1.35)	1.26 (0.91)	1.15 (1.17)	1.14 (0.77)	0.98	0.98	0.98	0.98 (0.96-0.99)	0.98 (0.96-0.99)	0.98 (0.97-0.99)
Left inferior parietal cortex	2.26 (0.17)	2.26 (0.17)	2.26 (0.18)	2.26 (0.18)	7.70	7.73	7.75	7.80	0.26 (0.79)	-0.17 (0.86)	-0.29 (0.77)	-0.05 (1.29)	0.03 (1.16)	0.06 (1.45)	0.89 (0.92)	0.99 (0.59)	1.10 (0.92)	0.99	0.99	0.98	0.99 (0.97-0.99)	0.99 (0.98-0.99)	0.98 (0.97-0.99)
Left inferior temporal gyrus	2.68 (0.16)	2.67 (0.17)	2.68 (0.16)	2.67 (0.16)	6.05	6.51	6.08	6.03	1.41 (0.17)	0.10 (0.92)	1.94 (0.06)	-0.40 (1.79)	-0.03 (1.71)	-0.46 (1.59)	1.18 (1.39)	1.33 (1.05)	1.24 (1.09)	0.96	0.96	0.97	0.96 (0.93-0.98)	0.96 (0.93-0.98)	0.96 (0.93-0.98)
Left isthmus cingulate cortex	2.24 (0.21)	2.23 (0.24)	2.23 (0.23)	2.24 (0.23)	9.21	10.86	10.23	10.12	1.27 (0.21)	1.49 (0.14)	-0.19 (0.85)	-0.81 (3.34)	-0.80 (3.10)	0.01 (3.46)	2.66 (2.15)	2.58 (1.85)	2.66 (2.17)	0.96	0.96	0.94	0.95 (0.90-0.97)	0.95 (0.91-0.97)	0.94 (0.88-0.96)
Left lateral occipital cortex	2.09 (0.13)	2.09 (0.13)	2.08 (0.12)	2.09 (0.12)	6.34	6.08	5.86	5.92	-0.71 (0.48)	0.17 (0.87)	-0.29 (0.77)	0.31 (2.66)	-0.03 (1.93)	0.10 (1.80)	1.56 (2.16)	1.49 (1.20)	1.25 (1.29)	0.90	0.95	0.96	0.90 (0.83-0.95)	0.95 (0.91-0.97)	0.96 (0.92-0.98)
Left lateral orbitofrontal cortex	2.58 (0.16)	2.57 (0.16)	2.58 (0.16)	2.58 (0.15)	6.05	6.07	6.06	5.76	1.77 (0.08)	0.15 (0.88)	0.47 (0.64)	-0.48 (1.78)	-0.03 (1.45)	-0.12 (1.96)	1.38 (1.20)	1.04 (1.00)	1.46 (1.29)	0.96	0.97	0.94	0.95 (0.92-0.98)	0.97 (0.95-0.98)	0.94 (0.90-0.97)
Left lingual gyrus	1.94 (0.09)	1.95 (0.09)	1.94 (0.09)	1.94 (0.10)	4.76	4.43	4.41	5.01	-0.80 (0.43)	-0.09 (0.93)	-0.54 (0.59)	0.22 (1.65)	0.04 (1.63)	0.13 (1.70)	1.24 (1.10)	1.30 (0.96)	1.19 (1.21)	0.94	0.94	0.94	0.94 (0.88-0.96)	0.94 (0.89-0.97)	0.94 (0.90-0.97)
Left medial orbitofrontal cortex	2.26 (0.16)	2.26 (0.17)	2.27 (0.17)	2.27 (0.17)	7.24	7.52	7.72	7.39	-0.34 (0.73)	-0.79 (0.43)	-1.71 (0.09)	0.10 (2.25)	0.27 (2.48)	0.55 (2.12)	1.74 (1.41)	1.98 (1.49)	1.74 (1.31)	0.95	0.94	0.96	0.95 (0.91-0.97)	0.94 (0.90-0.97)	0.95 (0.92-0.98)
Left middle temporal gyrus	2.67 (0.17)	2.66 (0.17)	2.67 (0.17)	2.66 (0.17)	6.29	6.53	6.27	6.24	1.41 (0.17)	1.15 (0.26)	2.03 (0.05)	-0.42 (1.89)	-0.25 (1.42)	-0.47 (1.52)	1.24 (1.48)	1.17 (0.82)	1.24 (0.99)	0.96	0.97	0.97	0.96 (0.92-0.98)	0.97 (0.95-0.99)	0.97 (0.94-0.98)
Left parahippocampal gyrus	2.68 (0.33)	2.65 (0.32)	2.67 (0.31)	2.66 (0.33)	12.29	11.90	11.77	12.34	3.45 (0.00)	1.34 (0.19)	2.30 (0.03)	-1.14 (2.19)	-0.35 (1.96)	-0.78 (2.24)	1.91 (1.55)	1.66 (1.08)	2.00 (1.25)	0.98	0.99	0.98	0.98 (0.95-0.99)	0.99 (0.97-0.99)	0.98 (0.97-0.99)
Left paracentral lobule	2.31 (0.13)	2.30 (0.12)	2.30 (0.14)	2.31 (0.12)	5.63	5.37	5.97	5.41	1.20 (0.24)	1.51 (0.14)	0.30 (0.76)	-0.40 (2.19)	-0.48 (2.05)	-0.08 (1.92)	1.72 (1.39)	1.48 (1.48)	1.53 (1.14)	0.92	0.94	0.94	0.92 (0.85-0.95)	0.94 (0.89-0.97)	0.94 (0.89-0.97)
Left pars opercularis	2.49 (0.11)	2.48 (0.10)	2.49 (0.10)	2.48 (0.11)	4.36	4.13	4.09	4.28	1.27 (0.21)	0.56 (0.58)	1.82 (0.08)	-0.39 (2.01)	-0.11 (1.43)	-0.42 (1.50)	1.16 (1.68)	1.12 (0.88)	1.16 (1.03)	0.88	0.94	0.94	0.88 (0.79-0.93)	0.94 (0.90-0.97)	0.94 (0.88-0.97)
Left pars orbitalis	2.64 (0.20)	2.63 (0.21)	2.64 (0.21)	2.64 (0.21)	7.49	8.12	7.81	8.05	0.87 (0.39)	0.04 (0.97)	0.62 (0.54)	-0.43 (2.87)	-0.04 (2.06)	-0.29 (2.57)	2.24 (1.81)	1.63 (1.22)	2.00 (1.61)	0.94	0.96	0.95	0.93 (0.88-0.96)	0.96 (0.93-0.98)	0.94 (0.90-0.97)
Left pars triangularis	2.35 (0.14)	2.34 (0.14)	2.34 (0.13)	2.35 (0.14)	5.95	6.12	5.53	6.11	1.37 (0.18)	0.91 (0.37)	0.58 (0.56)	-0.48 (2.25)	-0.23 (1.82)	-0.15 (1.58)	1.71 (1.52)	1.36 (1.21)	1.25 (0.96)	0.93	0.95	0.97	0.93 (0.87-0.96)	0.95 (0.91-0.97)	0.97 (0.94-0.98)
Left pericalcarine cortex	1.58 (0.10)	1.60 (0.14)	1.58 (0.09)	1.59 (0.10)	6.38	8.55	5.94	6.37	-1.00 (0.32)	0.02 (0.99)	-1.93 (0.06)	1.20 (7.71)	0.02 (2.86)	0.84 (2.91)	3.09 (7.15)	2.00 (2.01)	2.10 (2.16)	0.34	0.90	0.90	0.33 (0.04-0.57)	0.90 (0.82-0.94)	0.89 (0.81-0.94)
Left postcentral gyrus	1.98 (0.12)	1.98 (0.12)	1.98 (0.12)	1.98 (0.12)	5.93	6.00	6.02	5.91	-0.58 (0.57)	0.59 (0.56)	-0.48 (0.63)	0.13 (1.50)	-0.11 (1.18)	0.07 (0.95)	1.08 (1.03)	0.91 (0.74)	0.78 (0.52)	0.97	0.98	0.99	0.97 (0.95-0.98)	0.98 (0.96-0.99)	0.99 (0.98-0.99)
Left posterior cingulate cortex	2.37 (0.18)	2.35 (0.19)	2.36 (0.18)	2.34 (0.18)	7.40	8.05	7.66	7.87	1.75 (0.09)	1.30 (0.20)	2.83 (0.01)	-0.89 (3.25)	-0.63 (3.06)	-1.30 (2.93)	2.79 (1.84)	2.40 (1.97)	2.53 (1.94)	0.92	0.92	0.93	0.91 (0.85-0.95)	0.92 (0.85-0.95)	0.92 (0.83-0.96)
Left precentral gyrus	2.47 (0.14)	2.47 (0.14)	2.47 (0.14)	2.47 (0.14)	5.63	5.76	5.66	5.54	0.33 (0.74)	-0.13 (0.90)	-0.58 (0.57)	-0.09 (1.69)	0.02 (1.19)	0.12 (1.34)	0.98 (1.38)	0.89 (0.78)	1.05 (0.83)	0.96	0.98	0.97	0.96 (0.93-0.98)	0.98 (0.96-0.99)	0.97 (0.95-0.99)
Left precuneus	2.23 (0.17)	2.23 (0.18)	2.23 (0.18)	2.23 (0.17)	7.76	7.93	8.11	7.72	0.05 (0.96)	1.21 (0.23)	1.01 (0.32)	-0.02 (1.31)	-0.27 (1.31)	-0.17 (1.15)	1.03 (0.79)	1.06 (0.80)	0.93 (0.69)	0.99	0.99	0.99	0.99 (0.97-0.99)	0.99 (0.97-0.99)	0.99 (0.98-0.99)
Left rostral anterior cingulate cortex	2.60 (0.24)	2.61 (0.24)	2.60 (0.22)	2.60 (0.24)	9.35	9.39	8.29	9.08	-0.29 (0.77)	0.45 (0.65)	0.07 (0.94)	0.20 (4.61)	-0.17 (3.64)	-0.03 (4.36)	2.90 (3.56)	2.52 (2.60)	3.14 (2.98)	0.88	0.92	0.89	0.88 (0.79-0.93)	0.91 (0.84-0.95)	0.89 (0.80-0.94)
Left rostral middle frontal gyrus	2.25 (0.12)	2.24 (0.12)	2.25 (0.12)	2.24 (0.12)	5.35	5.37	5.20	5.19	0.52 (0.60)	-0.88 (0.38)	1.28 (0.21)	-0.16 (1.94)	0.21 (1.48)	-0.35 (1.80)	1.31 (1.42)	1.19 (0.89)	1.37 (1.21)	0.93	0.96	0.94	0.93 (0.88-0.96)	0.96 (0.93-0.98)	0.94 (0.89-0.97)
Left superior frontal gyrus	2.51 (0.13)	2.50 (0.12)	2.50 (0.13)	2.50 (0.13)	5.01	5.00	5.05	5.07	1.29 (0.20)	0.97 (0.34)	0.35 (0.73)	-0.33 (1.70)	-0.23 (1.56)	-0.07 (1.17)	1.13 (1.30)	1.30 (0.86)	0.99 (0.62)	0.94	0.95	0.97	0.94 (0.90-0.97)	0.95 (0.92-0.97)	0.97 (0.95-0.99)
Left superior parietal cortex	2.08 (0.16)	2.08 (0.16)	2.08 (0.16)	2.08 (0.16)	7.86	7.76	7.64	7.65	1.00 (0.32)	0.05 (0.96)	-0.40 (0.69)	-0.19 (1.28)	0.01 (1.03)	0.09 (1.33)	0.97 (0.84)	0.77 (0.67)	1.01 (0.86)	0.99	0.99	0.99	0.99 (0.98-0.99)	0.99 (0.98-1.00)	0.99 (0.97-0.99)
Left superior temporal gyrus	2.55 (0.17)	2.55 (0.17)	2.54 (0.17)	2.55 (0.17)	6.68	6.62	6.59	6.60	-0.42 (0.68)	0.66 (0.52)	-0.26 (0.79)	0.07 (1.07)	-0.09 (0.96)	0.04 (0.97)	0.73 (0.78)	0.78 (0.56)	0.76 (0.59)	0.99	0.99	0.99	0.99 (0.98-0.99)	0.99 (0.98-0.99)	0.99 (0.98-0.99)
Left supramarginal gyrus	2.37 (0.14)	2.37 (0.14)	2.37 (0.14)	2.37 (0.14)	5.84	5.88	5.90	5.79	-0.47 (0.64)	-0.55 (0.58)	-0.41 (0.69)	0.13 (1.82)	0.11 (1.36)	0.09 (1.33)	1.16 (1.39)	1.02 (0.90)	0.95 (0.92)	0.95	0.97	0.97	0.95 (0.92-0.98)	0.97 (0.95-0.99)	0.97 (0.95-0.99)
Left frontal pole	2.57 (0.22)	2.56 (0.24)	2.57 (0.22)	2.55 (0.23)	8.73	9.19	8.51	9.16	0.78 (0.44)	-0.06 (0.96)	1.40 (0.17)	-0.45 (3.42)	0.05 (3.40)	-0.65 (2.87)	2.61 (2.22)	2.32 (2.46)	2.12 (2.01)	0.92	0.92	0.95	0.92 (0.86-0.96)	0.93 (0.87-0.96)	0.95 (0.90-0.97)
Left temporal pole	3.52 (0.39)	3.48 (0.39)	3.50 (0.38)	3.51 (0.41)	10.94	11.24	10.84	11.62	1.76 (0.09)	1.05 (0.30)	0.85 (0.40)	-1.23 (4.64)	-0.66 (4.27)	-0.63 (4.35)	3.26 (3.49)	3.22 (2.84)	2.92 (3.25)	0.92	0.92	0.93	0.91 (0.85-0.95)	0.92 (0.87-0.96)	0.93 (0.88-0.96)

Left transverse temporal gyrus	2.25 (0.21)	2.23 (0.22)	2.25 (0.21)	2.25 (0.22)	9.48	9.76	9.48	9.59	2.35 (0.02)	0.43 (0.67)	0.95 (0.35)	-0.83 (2.35)	-0.15 (2.32)	-0.25 (1.60)	1.95 (1.54)	1.65 (1.62)	1.24 (1.03)	0.97	0.97	0.99	0.97 (0.94-0.98)	0.97 (0.95-0.98)	0.99 (0.97-0.99)
Left insula	2.78 (0.20)	2.76 (0.20)	2.79 (0.21)	2.78 (0.21)	7.08	7.07	7.55	7.72	1.15 (0.26)	-0.29 (0.77)	0.15 (0.88)	-0.58 (3.19)	0.15 (4.14)	-0.13 (3.80)	2.43 (2.12)	3.02 (2.80)	2.93 (2.37)	0.89	0.84	0.87	0.89 (0.81-0.94)	0.84 (0.72-0.91)	0.87 (0.77-0.93)
Right banks superior temporal sulcus	2.43 (0.16)	2.42 (0.18)	2.43 (0.17)	2.42 (0.17)	6.79	7.26	7.05	7.13	1.73 (0.09)	0.23 (0.82)	0.86 (0.40)	-0.44 (1.57)	-0.07 (1.60)	-0.22 (1.53)	1.21 (1.08)	1.19 (1.06)	1.25 (0.89)	0.98	0.97	0.98	0.97 (0.95-0.99)	0.97 (0.95-0.99)	0.98 (0.96-0.99)
Right caudal anterior cingulate cortex	2.47 (0.21)	2.45 (0.21)	2.44 (0.20)	2.44 (0.21)	8.49	8.72	8.12	8.42	1.79 (0.08)	2.71 (0.01)	2.99 (0.00)	-0.85 (3.20)	-1.16 (2.81)	-1.01 (2.30)	2.36 (2.29)	2.39 (1.84)	1.96 (1.55)	0.94	0.94	0.97	0.93 (0.88-0.96)	0.93 (0.86-0.96)	0.96 (0.91-0.98)
Right caudal middle frontal gyrus	2.40 (0.15)	2.40 (0.15)	2.40 (0.15)	2.41 (0.15)	6.06	6.12	6.10	6.06	0.99 (0.33)	1.16 (0.25)	-0.36 (0.72)	-0.28 (1.87)	-0.31 (1.71)	0.10 (1.88)	1.39 (1.26)	1.31 (1.12)	1.47 (1.15)	0.95	0.96	0.95	0.95 (0.92-0.97)	0.96 (0.93-0.98)	0.95 (0.92-0.97)
Right cuneus	1.83 (0.14)	1.84 (0.15)	1.84 (0.14)	1.84 (0.13)	7.55	8.14	7.36	7.29	-0.96 (0.34)	-0.86 (0.39)	-1.35 (0.18)	0.53 (3.44)	0.25 (1.74)	0.35 (1.69)	1.62 (3.07)	1.33 (1.14)	1.20 (1.23)	0.88	0.97	0.98	0.88 (0.79-0.93)	0.97 (0.95-0.98)	0.98 (0.96-0.99)
Right entorhinal cortex	3.30 (0.46)	3.27 (0.47)	3.25 (0.46)	3.30 (0.44)	14.00	14.36	14.24	13.46	1.56 (0.13)	1.60 (0.12)	0.09 (0.93)	-0.97 (4.00)	-1.41 (5.74)	-0.01 (5.60)	3.01 (2.78)	3.94 (4.37)	4.08 (3.78)	0.96	0.92	0.92	0.96 (0.93-0.98)	0.92 (0.85-0.95)	0.92 (0.86-0.96)
Right fusiform gyrus	2.60 (0.19)	2.60 (0.19)	2.61 (0.19)	2.61 (0.19)	7.18	7.28	7.18	7.12	1.42 (0.16)	-0.31 (0.76)	-1.02 (0.31)	-0.27 (1.20)	0.07 (1.43)	0.23 (1.46)	0.99 (0.70)	1.19 (0.77)	1.17 (0.90)	0.99	0.98	0.98	0.99 (0.97-0.99)	0.98 (0.96-0.99)	0.98 (0.96-0.99)
Right inferior parietal cortex	2.26 (0.15)	2.26 (0.15)	2.25 (0.15)	2.26 (0.16)	6.63	6.67	6.57	7.05	1.13 (0.27)	1.51 (0.14)	0.62 (0.54)	-0.27 (1.55)	-0.34 (1.45)	-0.17 (1.57)	1.14 (1.06)	1.04 (1.06)	1.15 (1.07)	0.97	0.97	0.98	0.97 (0.95-0.99)	0.97 (0.95-0.99)	0.97 (0.95-0.99)
Right inferior temporal gyrus	2.69 (0.14)	2.69 (0.14)	2.69 (0.15)	2.70 (0.14)	5.32	5.04	5.41	5.04	0.73 (0.47)	0.15 (0.88)	-0.85 (0.40)	-0.21 (2.07)	-0.04 (1.41)	0.24 (1.79)	1.30 (1.61)	1.08 (0.89)	1.27 (1.27)	0.92	0.97	0.94	0.92 (0.86-0.96)	0.97 (0.94-0.98)	0.94 (0.89-0.97)
Right isthmus cingulate cortex	2.23 (0.26)	2.22 (0.27)	2.20 (0.28)	2.21 (0.30)	11.88	11.97	12.77	13.42	0.84 (0.41)	1.77 (0.08)	1.14 (0.26)	-0.41 (3.31)	-1.07 (4.04)	-0.97 (5.07)	2.37 (2.32)	2.51 (3.33)	2.91 (4.24)	0.96	0.96	0.96	0.96 (0.94-0.98)	0.96 (0.93-0.98)	0.95 (0.91-0.97)
Right lateral occipital cortex	2.12 (0.13)	2.12 (0.13)	2.11 (0.13)	2.12 (0.13)	6.16	6.13	6.26	6.34	-1.20 (0.23)	0.76 (0.45)	-1.58 (0.12)	0.20 (1.13)	-0.22 (1.83)	0.25 (1.09)	0.80 (0.81)	1.23 (1.37)	0.93 (0.60)	0.98	0.96	0.99	0.98 (0.97-0.99)	0.96 (0.92-0.98)	0.98 (0.97-0.99)
Right lateral orbitofrontal cortex	2.55 (0.14)	2.52 (0.13)	2.52 (0.13)	2.53 (0.14)	5.40	5.36	5.33	5.70	3.36 (0.00)	2.85 (0.01)	1.23 (0.23)	-1.07 (2.11)	-1.09 (2.47)	-0.59 (3.00)	1.93 (1.35)	2.05 (1.73)	2.11 (2.19)	0.92	0.89	0.85	0.91 (0.80-0.95)	0.88 (0.76-0.93)	0.85 (0.73-0.91)
Right lingual gyrus	2.00 (0.12)	2.00 (0.12)	2.00 (0.12)	2.00 (0.12)	6.15	5.93	5.99	6.12	0.57 (0.57)	0.20 (0.84)	0.59 (0.56)	-0.17 (2.26)	-0.04 (1.56)	-0.14 (1.72)	1.50 (1.68)	1.19 (1.00)	1.14 (1.29)	0.93	0.97	0.96	0.93 (0.88-0.96)	0.97 (0.94-0.98)	0.97 (0.94-0.98)
Right medial orbitofrontal cortex	2.34 (0.14)	2.33 (0.15)	2.33 (0.15)	2.34 (0.16)	5.97	6.54	6.48	6.70	1.93 (0.06)	1.55 (0.13)	0.05 (0.96)	-0.71 (2.26)	-0.55 (2.19)	-0.07 (2.86)	1.93 (1.35)	1.73 (1.42)	2.07 (1.95)	0.94	0.94	0.91	0.93 (0.87-0.96)	0.94 (0.88-0.96)	0.90 (0.82-0.95)
Right middle temporal gyrus	2.73 (0.15)	2.72 (0.17)	2.73 (0.16)	2.73 (0.16)	5.64	6.19	5.97	5.95	0.96 (0.34)	0.67 (0.51)	0.44 (0.66)	-0.23 (1.39)	-0.14 (1.19)	-0.11 (1.37)	0.95 (1.03)	0.88 (0.80)	1.11 (0.80)	0.98	0.98	0.97	0.97 (0.95-0.99)	0.98 (0.96-0.99)	0.97 (0.95-0.99)
Right parahippocampal gyrus	2.63 (0.29)	2.62 (0.29)	2.61 (0.29)	2.62 (0.28)	11.19	10.93	11.14	10.71	1.48 (0.15)	2.03 (0.05)	1.27 (0.21)	-0.47 (2.25)	-0.80 (2.62)	-0.39 (2.31)	1.83 (1.37)	2.17 (1.64)	1.85 (1.42)	0.98	0.97	0.98	0.98 (0.96-0.99)	0.97 (0.95-0.98)	0.98 (0.96-0.99)
Right paracentral lobule	2.31 (0.13)	2.31 (0.14)	2.31 (0.14)	2.30 (0.14)	5.59	5.98	5.96	6.12	0.31 (0.76)	0.77 (0.44)	1.05 (0.30)	-0.10 (1.72)	-0.22 (1.64)	-0.30 (1.70)	1.39 (1.00)	1.30 (1.00)	1.28 (1.14)	0.96	0.96	0.96	0.96 (0.92-0.98)	0.96 (0.93-0.98)	0.96 (0.93-0.98)
Right pars opercularis	2.50 (0.13)	2.49 (0.14)	2.50 (0.14)	2.50 (0.13)	5.29	5.63	5.51	5.28	0.82 (0.42)	-0.49 (0.63)	-0.83 (0.41)	-0.24 (1.76)	0.09 (1.37)	0.20 (1.59)	1.24 (1.26)	1.04 (0.88)	1.20 (1.05)	0.95	0.97	0.96	0.95 (0.91-0.97)	0.97 (0.94-0.98)	0.96 (0.92-0.98)
Right pars orbitalis	2.60 (0.17)	2.60 (0.17)	2.60 (0.16)	2.62 (0.17)	6.50	6.63	6.22	6.45	0.01 (0.99)	0.46 (0.65)	-1.68 (0.10)	-0.01 (2.32)	-0.15 (2.31)	0.58 (2.29)	1.74 (1.52)	1.86 (1.35)	1.70 (1.62)	0.94	0.93	0.94	0.94 (0.89-0.97)	0.93 (0.88-0.96)	0.94 (0.89-0.97)
Right pars triangularis	2.39 (0.15)	2.37 (0.15)	2.38 (0.14)	2.39 (0.15)	6.14	6.27	6.06	6.39	1.52 (0.14)	0.42 (0.68)	-0.14 (0.89)	-0.56 (2.36)	-0.10 (1.67)	0.02 (1.74)	1.56 (1.84)	1.29 (1.05)	1.35 (1.08)	0.93	0.96	0.96	0.93 (0.87-0.96)	0.96 (0.93-0.98)	0.96 (0.93-0.98)
Right pericalcarine cortex	1.63 (0.13)	1.62 (0.12)	1.62 (0.13)	1.62 (0.12)	7.92	7.56	7.99	7.28	1.29 (0.21)	1.53 (0.13)	0.57 (0.57)	-0.41 (2.26)	-0.56 (2.50)	-0.18 (2.77)	1.63 (1.61)	1.79 (1.82)	1.77 (2.11)	0.96	0.95	0.94	0.96 (0.92-0.98)	0.95 (0.91-0.97)	0.94 (0.89-0.97)
Right postcentral gyrus	1.99 (0.10)	1.98 (0.11)	1.98 (0.11)	1.98 (0.11)	5.23	5.52	5.46	5.46	0.52 (0.61)	0.94 (0.35)	0.87 (0.39)	-0.14 (1.59)	-0.20 (1.31)	-0.16 (1.12)	0.95 (1.28)	1.05 (0.79)	0.91 (0.65)	0.96	0.97	0.98	0.96 (0.93-0.98)	0.97 (0.95-0.98)	0.98 (0.96-0.99)
Right posterior cingulate cortex	2.29 (0.19)	2.29 (0.19)	2.29 (0.18)	2.30 (0.19)	8.21	8.49	7.77	8.40	0.10 (0.92)	0.32 (0.75)	-0.14 (0.89)	-0.09 (3.81)	-0.15 (3.71)	0.05 (4.53)	2.35 (2.98)	2.69 (2.53)	2.93 (3.43)	0.90	0.89	0.85	0.90 (0.82-0.94)	0.90 (0.82-0.94)	0.85 (0.74-0.92)
Right precentral gyrus	2.47 (0.13)	2.48 (0.14)	2.47 (0.13)	2.48 (0.13)	5.06	5.50	5.20	5.07	-0.59 (0.56)	-0.05 (0.96)	-0.77 (0.44)	0.12 (1.59)	0.01 (1.41)	0.15 (1.30)	1.17 (1.06)	1.07 (0.91)	0.89 (0.94)	0.96	0.96	0.97	0.96 (0.92-0.98)	0.96 (0.93-0.98)	0.97 (0.94-0.98)
Right precuneus	2.24 (0.18)	2.23 (0.17)	2.23 (0.18)	2.23 (0.18)	7.88	7.69	7.90	8.01	1.77 (0.08)	0.58 (0.57)	1.17 (0.25)	-0.39 (1.47)	-0.12 (1.29)	-0.25 (1.29)	0.98 (1.15)	0.98 (0.82)	0.91 (0.94)	0.98	0.99	0.99	0.98 (0.96-0.99)	0.99 (0.97-0.99)	0.99 (0.97-0.99)
Right rostral anterior cingulate cortex	2.88 (0.23)	2.86 (0.24)	2.85 (0.24)	2.88 (0.23)	7.85	8.38	8.57	7.82	1.34 (0.19)	1.68 (0.10)	0.08 (0.94)	-0.68 (3.24)	-0.87 (3.27)	-0.03 (2.53)	2.57 (2.04)	2.39 (2.36)	1.97 (1.56)	0.93	0.93	0.95	0.92 (0.86-0.96)	0.92 (0.86-0.96)	0.95 (0.91-0.97)
Right rostral middle frontal gyrus	2.27 (0.13)	2.26 (0.14)	2.27 (0.13)	2.26 (0.14)	5.83	6.05	5.84	6.09	1.38 (0.18)	-0.33 (0.75)	1.48 (0.15)	-0.36 (1.62)	0.07 (1.47)	-0.35 (1.48)	1.12 (1.21)	1.21 (0.81)	1.24 (0.86)	0.96	0.97	0.97	0.96 (0.93-0.98)	0.97 (0.94-0.98)	0.97 (0.94-0.98)
Right superior frontal gyrus	2.54 (0.13)	2.53 (0.14)	2.53 (0.14)	2.53 (0.14)	5.15	5.47	5.35	5.39	1.88 (0.07)	1.18 (0.24)	1.40 (0.17)	-0.46 (1.60)	-0.27 (1.46)	-0.23 (1.04)	1.12 (1.23)	1.09 (0.99)	0.83 (0.66)	0.96	0.96	0.98	0.95 (0.92-0.98)	0.96 (0.93-0.98)	0.98 (0.96-0.99)
Right superior parietal cortex	2.07 (0.16)	2.07 (0.16)	2.07 (0.16)	2.07 (0.16)	7.74	7.65	7.82	7.86	1.00 (0.32)	0.51 (0.61)	-0.01 (0.99)	-0.22 (1.51)	-0.13 (1.58)	-0.01 (1.02)	0.97 (1.17)	1.16 (1.06)	0.84 (0.56)	0.98	0.98	0.99	0.98 (0.97-0.99)	0.98 (0.97-0.99)	0.99 (0.99-1.00)
Right superior temporal gyrus	2.61 (0.17)	2.60 (0.17)	2.61 (0.17)	2.61 (0.17)	6.35	6.58	6.53	6.70	1.56 (0.13)	0.60 (0.55)	0.40 (0.69)	-0.43 (1.77)	-0.11 (1.03)	-0.09 (1.12)	1.01 (1.52)	0.70 (0.75)	0.82 (0.76)	0.96	0.99	0.99	0.96 (0.93-0.98)	0.99 (0.98-0.99)	0.99 (0.98-0.99)
Right supramarginal gyrus	2.37 (0.11)	2.37 (0.12)	2.37 (0.10)	2.38 (0.12)	4.78	5.25	4.39	5.01	1.30 (0.20)	1.22 (0.23)	-0.50 (0.62)	-0.32 (1.58)	-0.22 (1.29)	0.09 (1.38)	0.96 (1.29)	1.03 (0.79)	1.05 (0.87)	0.96	0.96	0.96	0.96 (0.92-0.98)	0.96 (0.93-0.98)	0.96 (0.93-0.98)
Right frontal pole	2.62 (0.22)	2.60 (0.20)	2.60 (0.24)	2.60 (0.22)	8.35	7.76	9.35	8.40	1.43 (0.16)	0.90 (0.38)	1.57 (0.12)	-0.79 (3.70)	-0.78 (5.29)	-0.75 (3.00)	2.69 (2.64)	3.21 (4.25)	2.63 (1.59)	0.89	0.84	0.93	0.89 (0.80-0.94)	0.84 (0.72-0.91)	0.93 (0.88-0.96)
Right temporal pole	3.56 (0.36)	3.57 (0.35)	3.55 (0.37)	3.60 (0.33)	10.23	9.79	10.56	9.11	-0.45 (0.66)	0.70 (0.49)	-1.30 (0.20)	0.38 (5.43)	-0.47 (4.24)	1.17 (5.74)	3.32 (4.28)	3.04 (2.95)	3.40 (4.74)	0.87	0.92	0.85	0.88 (0.78-0.93)	0.92 (0.86-0.96)	0.85 (0.73-0.91)
Right transverse temporal gyrus	2.32 (0.27)	2.31 (0.27)	2.32 (0.28)	2.33 (0.28)	11.78	11.61	12.11	11.79	0.87 (0.39)	-1.07 (0.29)	-2.34 (0.02)	-0.31 (2.46)	0.30 (2.09)	0.70 (1.98)	1.81 (1.67)	1.69 (1.25)	1.58 (1.36)	0.98	0.98	0.99	0.98 (0.96-0.99)	0.98 (0.97-0.99)	0.98 (0.97-0.99)
Right insula	2.77 (0.19)	2.78 (0.20)	2.77 (0.19)	2.80 (0.22)	6.84	7.10	7.02	7.69	-0.91 (0.37)	-0.33 (0.75)	-1.78 (0.08)	0.45 (3.47)	0.13 (2.97)	0.84 (3.35)	2.82 (2.03)	2.30 (1.86)	2.73 (2.09)	0.88	0.91	0.90	0.88 (0.79-0.93)	0.91 (0.84-0.95)	0.89 (0.81-0.94)
Global brain measures																						
Left total cortical volume (ml)	204.13 (21.66)	203.72 (22.02)	204.01 (21.88)	204.34 (21.88)	10.61	10.81	10.72	10.71	1.12 (0.27)	0.58 (0.56)	-1.28 (0.21)	-0.22 (1.26)	-0.07 (0.70)	0.09 (0.53)	0.60 (1.12)	0.49 (0.50)	0.42 (0.33)	0.99	1.00	1.00	0.99 (0.99-1.00)	1.00 (1.00-1.00)	1.00 (1.00-1.00)
Right total cortical volume (ml)	206.22 (22.14)	205.60 (22.57)	205.87 (22.18)	206.46	10.73	10.98	10.77	10.86	1.35 (0.18)	1.38 (0.18)	-1.29 (0.20)	-0.33 (1.57)	-0.17 (0.80)	0.11 (0.60)	0.60 (1.49)	0.59 (0.56)	0.51 (0.33)	0.99	1.00	1.00	0.99 (0.98-1.00)	1.00 (0.99-1.00)	1.00 (1.00-1.00)

				(22.42)																			
Left total cortical white matter (ml)	210.68 (32.31)	210.45 (32.92)	210.68 (32.08)	210.88 (31.98)	15.34	15.64	15.23	15.16	0.33 (0.75)	0.01 (0.99)	-0.69 (0.49)	-0.14 (2.35)	0.02 (1.16)	0.13 (0.93)	1.20 (2.02)	0.90 (0.72)	0.75 (0.55)	0.99	1.00	1.00	0.99 (0.98-0.99)	1.00 (0.99-1.00)	1.00 (1.00-1.00)
Right total cortical white matter (ml)	210.65 (31.41)	210.22 (32.07)	210.64 (30.88)	210.82 (31.67)	14.91	15.25	14.66	15.02	0.92 (0.36)	0.03 (0.98)	-0.48 (0.63)	-0.25 (1.48)	0.03 (1.00)	0.07 (1.06)	0.89 (1.20)	0.76 (0.64)	0.80 (0.68)	1.00	1.00	1.00	1.00 (0.99-1.00)	1.00 (1.00-1.00)	1.00 (1.00-1.00)
Left total cortical surface area (cm²)	782.37 (89.05)	782.54 (90.29)	782.33 (88.65)	784.82 (88.75)	11.38	11.54	11.33	11.31	-0.14 (0.89)	0.06 (0.95)	-4.14 (0.00)	0.01 (1.16)	0.00 (0.62)	0.32 (0.55)	0.61 (0.98)	0.46 (0.42)	0.46 (0.44)	1.00	1.00	1.00	1.00 (0.99-1.00)	1.00 (1.00-1.00)	1.00 (1.00-1.00)
Right total cortical surface area (cm²)	785.24 (88.21)	785.21 (90.39)	785.28 (88.08)	786.95 (89.09)	11.23	11.51	11.22	11.32	0.02 (0.98)	-0.05 (0.96)	-2.14 (0.04)	-0.04 (1.04)	0.01 (0.63)	0.21 (0.65)	0.60 (0.85)	0.49 (0.39)	0.52 (0.43)	1.00	1.00	1.00	1.00 (0.99-1.00)	1.00 (1.00-1.00)	1.00 (1.00-1.00)
Left mean cortical thickness (mm)	2.34 (0.10)	2.34 (0.10)	2.34 (0.10)	2.34 (0.10)	4.34	4.39	4.32	4.36	1.04 (0.30)	0.55 (0.58)	0.54 (0.59)	-0.11 (0.67)	-0.06 (0.70)	-0.05 (0.62)	0.53 (0.42)	0.50 (0.48)	0.48 (0.38)	0.99	0.99	0.99	0.99 (0.98-0.99)	0.99 (0.98-0.99)	0.99 (0.98-0.99)
Right mean cortical tickness (mm)	2.36 (0.10)	2.35 (0.10)	2.35 (0.10)	2.36 (0.11)	4.35	4.43	4.41	4.46	1.89 (0.07)	1.70 (0.10)	0.32 (0.75)	-0.25 (0.85)	-0.20 (0.73)	-0.03 (0.59)	0.48 (0.74)	0.51 (0.55)	0.45 (0.37)	0.98	0.99	0.99	0.98 (0.96-0.99)	0.98 (0.97-0.99)	0.99 (0.98-1.00)
Left cerebellar white matter (ml)	14.88 (1.68)	14.82 (1.42)	14.97 (1.72)	14.41 (1.63)	11.31	9.60	11.47	11.28	0.66 (0.51)	-1.01 (0.32)	4.06 (0.00)	-0.29 (4.12)	0.54 (3.54)	-3.26 (5.34)	3.10 (2.69)	2.79 (2.20)	4.58 (4.22)	0.93	0.95	0.89	0.91 (0.85-0.95)	0.95 (0.91-0.97)	0.86 (0.65-0.93)
Right cerebellar white matter (ml)	14.76 (2.32)	14.51 (1.43)	14.49 (1.70)	14.48 (3.05)	15.69	9.84	11.74	21.05	1.03 (0.31)	1.09 (0.28)	0.72 (0.48)	-1.16 (8.65)	-1.48 (8.87)	-2.57 (13.26)	4.46 (7.47)	4.13 (7.97)	7.47 (11.20)	0.72	0.70	0.56	0.65 (0.44-0.79)	0.66 (0.46-0.80)	0.54 (0.29-0.72)
Left cerebellar gray matter (ml)	49.15 (4.41)	49.20 (4.46)	49.12 (4.42)	49.65 (4.47)	8.97	9.07	8.99	9.01	-0.46 (0.65)	0.37 (0.71)	-5.19 (0.00)	0.10 (1.55)	-0.06 (1.01)	1.02 (1.27)	1.03 (1.15)	0.73 (0.70)	1.22 (1.07)	0.98	0.99	0.99	0.99 (0.97-0.99)	0.99 (0.99-1.00)	0.98 (0.92-0.99)
Right cerebellar gray matter (ml)	49.26 (4.60)	49.28 (4.54)	49.16 (4.44)	49.57 (4.90)	9.34	9.21	9.04	9.89	-0.08 (0.94)	0.39 (0.70)	-1.00 (0.32)	0.04 (3.00)	-0.18 (3.49)	0.57 (4.41)	1.47 (2.60)	1.45 (3.18)	2.19 (3.85)	0.95	0.92	0.91	0.95 (0.91-0.97)	0.93 (0.87-0.96)	0.91 (0.84-0.95)
Intracranial volume (ml)	1511.53 (214.93)	1507.06 (221.30)	1506.74 (215.21)	1529.33 (199.68)	14.22	14.68	14.28	13.06	0.99 (0.33)	4.78 (0.00)	-2.89 (0.01)	-0.37 (2.55)	-0.32 (0.46)	1.33 (3.08)	0.79 (2.45)	0.40 (0.39)	1.38 (3.06)	0.99	1.00	0.98	0.99 (0.98-0.99)	1.00 (1.00-1.00)	0.98 (0.95-0.99)
Total brain volume without ventricles (ml)	1011.87 (113.09)	1009.93 (115.62)	1011.07 (113.24)	1012.58 (113.74)	11.18	11.45	11.20	11.23	0.95 (0.35)	1.00 (0.32)	-1.09 (0.28)	-0.22 (1.40)	-0.08 (0.53)	0.06 (0.42)	0.52 (1.32)	0.40 (0.36)	0.32 (0.27)	0.99	1.00	1.00	0.99 (0.99-1.00)	1.00 (1.00-1.00)	1.00 (1.00-1.00)

Abbreviations: SD = standard deviation; Orig = original scan; FS df = FreeSurfer defacing; FSL df = FSL defacing; FM = Face Masking; t = t-statistic; *p*=p-value; PD = Average Percentage Difference; CI = Confidence Interval.

Table S4. Effects of de-identification on brain measures in older adults diagnosed with Alzheimer's disease.

Brain metric	Mean (SD)				Coefficient of Variation				Paired t-test t(*p*)			Signed PD in %			Absolute PD (%)			Pearson's r			ICC (95% CI)		
	Orig	FS df	FSL df	FM	Orig	FS df	FSL df	FM	FS df	FSL df	FM	FS df	FSL df	FM	FS df	FSL df	FM	FS df	FSL df	FM	FS df	FSL df	FM
Subcortical volume (ml)																							
Left thalamus	6.36 (0.94)	6.35 (0.93)	6.38 (0.98)	6.33 (0.91)	14.78	14.61	15.41	14.36	0.15 (0.88)	-0.44 (0.66)	0.44 (0.66)	-0.10 (4.57)	0.23 (3.24)	-0.43 (4.73)	2.78 (3.58)	2.72 (1.67)	3.10 (3.53)	0.94	0.98	0.93	0.94 (0.86-0.97)	0.98 (0.94-0.99)	0.94 (0.85-0.97)
Left caudatus	3.15 (0.48)	3.16 (0.48)	3.12 (0.46)	3.15 (0.48)	15.37	15.36	14.80	15.15	-0.23 (0.82)	0.66 (0.51)	-0.19 (0.85)	0.22 (4.42)	-0.62 (5.23)	0.22 (3.52)	2.75 (3.41)	2.93 (4.33)	2.25 (2.68)	0.95	0.94	0.97	0.96 (0.90-0.98)	0.94 (0.86-0.97)	0.97 (0.93-0.99)
Left putamen	3.93 (0.63)	4.00 (0.52)	3.94 (0.64)	3.95 (0.57)	16.07	13.00	16.35	14.47	-1.27 (0.22)	-0.43 (0.67)	-0.63 (0.54)	2.41 (9.08)	0.31 (3.41)	0.98 (6.69)	3.60 (8.66)	2.42 (2.37)	2.59 (6.22)	0.91	0.98	0.96	0.89 (0.75-0.95)	0.98 (0.95-0.99)	0.95 (0.89-0.98)
Left pallidum	1.91 (0.22)	1.90 (0.21)	1.90 (0.18)	1.89 (0.20)	11.65	10.84	9.71	10.49	0.56 (0.58)	0.23 (0.82)	0.79 (0.44)	-0.36 (3.38)	-0.09 (5.61)	-0.73 (4.87)	2.52 (2.22)	4.57 (3.09)	3.53 (3.36)	0.95	0.88	0.91	0.95 (0.88-0.98)	0.87 (0.70-0.94)	0.90 (0.78-0.96)
Left hippocampus	3.04 (0.50)	3.01 (0.52)	3.02 (0.50)	3.00 (0.50)	16.53	17.26	16.45	16.63	1.31 (0.20)	0.80 (0.43)	3.28 (0.00)	-1.02 (3.27)	-0.47 (2.98)	-1.38 (2.04)	2.71 (2.02)	2.38 (1.79)	1.83 (1.63)	0.98	0.98	0.99	0.98 (0.96-0.99)	0.98 (0.96-0.99)	0.99 (0.96-1.00)
Left amygdala	1.18 (0.22)	1.20 (0.23)	1.17 (0.22)	1.15 (0.21)	18.34	19.58	18.45	18.63	-1.67 (0.11)	0.35 (0.73)	2.28 (0.03)	1.80 (5.31)	-0.37 (4.45)	-2.24 (5.12)	4.27 (3.53)	3.66 (2.44)	4.11 (3.71)	0.96	0.97	0.97	0.95 (0.89-0.98)	0.97 (0.94-0.99)	0.97 (0.91-0.99)
Left accumbens	0.34 (0.10)	0.34 (0.10)	0.34 (0.10)	0.33 (0.10)	30.67	30.69	28.15	29.90	-0.07 (0.94)	-0.36 (0.72)	1.36 (0.19)	0.04 (10.52)	1.23 (9.79)	-2.98 (12.92)	8.75 (5.53)	7.27 (6.49)	9.87 (8.61)	0.94	0.97	0.94	0.95 (0.88-0.98)	0.96 (0.92-0.99)	0.94 (0.86-0.97)
Right thalamus	6.08 (0.73)	6.15 (0.79)	6.11 (0.80)	6.18 (0.82)	11.94	12.84	13.03	13.21	-1.97 (0.06)	-0.52 (0.61)	-2.07 (0.05)	1.10 (2.51)	0.40 (4.11)	1.45 (3.04)	2.02 (1.82)	2.40 (3.32)	2.25 (2.47)	0.98	0.94	0.97	0.97 (0.93-0.99)	0.94 (0.86-0.97)	0.95 (0.89-0.98)
Right caudatus	3.23 (0.42)	3.27 (0.44)	3.23 (0.43)	3.23 (0.45)	12.95	13.49	13.35	13.81	-2.73 (0.01)	0.06 (0.95)	0.15 (0.88)	1.26 (2.15)	-0.08 (3.25)	-0.23 (3.65)	1.72 (1.79)	2.21 (2.34)	2.27 (2.82)	0.99	0.97	0.96	0.98 (0.94-0.99)	0.97 (0.92-0.99)	0.96 (0.91-0.98)
Right putamen	4.04 (0.61)	4.07 (0.62)	4.09 (0.63)	4.03 (0.58)	15.22	15.31	15.44	14.47	-1.29 (0.21)	-2.74 (0.01)	0.33 (0.74)	0.88 (3.61)	1.17 (1.96)	-0.18 (3.87)	2.47 (2.73)	1.89 (1.25)	2.38 (3.02)	0.98	0.99	0.96	0.98 (0.95-0.99)	0.99 (0.96-1.00)	0.96 (0.91-0.98)
Right pallidum	1.78 (0.23)	1.77 (0.22)	1.79 (0.23)	1.77 (0.23)	13.02	12.40	12.80	12.83	1.33 (0.20)	-0.34 (0.74)	0.50 (0.62)	-1.06 (3.94)	0.28 (3.61)	-0.73 (6.18)	3.26 (2.36)	3.05 (1.83)	4.09 (4.61)	0.95	0.96	0.87	0.95 (0.89-0.98)	0.96 (0.91-0.98)	0.87 (0.71-0.94)
Right hippocampus	3.23 (0.56)	3.20 (0.57)	3.20 (0.55)	3.21 (0.54)	17.35	17.88	17.08	16.95	2.10 (0.05)	1.90 (0.07)	2.13 (0.05)	-1.09 (2.47)	-0.80 (2.58)	-0.68 (2.01)	2.03 (1.74)	1.99 (1.79)	1.70 (1.24)	0.99	0.99	1.00	0.99 (0.98-1.00)	0.99 (0.98-1.00)	0.99 (0.98-1.00)
Right amygdala	1.46 (0.28)	1.47 (0.26)	1.45 (0.27)	1.45 (0.29)	19.38	18.00	18.89	19.86	-0.96 (0.35)	0.43 (0.67)	1.12 (0.28)	0.98 (3.70)	-0.25 (3.31)	-0.86 (3.26)	2.82 (2.53)	2.60 (1.99)	2.71 (1.92)	0.99	0.98	0.99	0.99 (0.97-0.99)	0.98 (0.96-0.99)	0.99 (0.97-0.99)
Right accumbens	0.43 (0.10)	0.44 (0.11)	0.44 (0.10)	0.43 (0.10)	23.71	25.14	23.21	23.26	-2.32 (0.03)	-1.36 (0.19)	-0.76 (0.46)	2.82 (6.20)	2.24 (7.37)	1.40 (7.38)	4.42 (5.12)	4.92 (5.85)	5.94 (4.43)	0.97	0.95	0.95	0.96 (0.88-0.98)	0.95 (0.88-0.98)	0.95 (0.90-0.98)
Ventricular volume (ml)																							
Third ventricle	2.23 (0.72)	2.21 (0.68)	2.17 (0.64)	2.18 (0.64)	32.41	30.92	29.63	29.24	0.58 (0.57)	0.80 (0.43)	0.65 (0.52)	-0.62 (4.57)	-1.98 (10.85)	-1.30 (9.87)	2.34 (3.94)	3.89 (10.30)	3.75 (9.19)	0.98	0.88	0.90	0.98 (0.95-0.99)	0.88 (0.73-0.95)	0.90 (0.77-0.96)
Left lateral ventricle	26.17 (12.03)	26.17 (12.01)	26.10 (11.79)	26.20 (12.03)	45.98	45.90	45.17	45.91	0.04 (0.96)	0.54 (0.60)	-0.78 (0.44)	0.00 (0.55)	-0.03 (1.43)	0.18 (0.82)	0.44 (0.32)	0.84 (1.15)	0.61 (0.56)	1.00	1.00	1.00	1.00 (1.00-1.00)	1.00 (1.00-1.00)	1.00 (1.00-1.00)
Left inferior lateral ventricle	1.48 (1.21)	1.49 (1.22)	1.48 (1.18)	1.47 (1.19)	81.54	81.49	80.11	80.77	-0.39 (0.70)	0.25 (0.80)	0.42 (0.68)	1.14 (7.55)	1.42 (8.78)	0.22 (8.28)	5.77 (4.84)	5.37 (7.00)	6.62 (4.77)	1.00	1.00	1.00	1.00 (0.99-1.00)	1.00 (0.99-1.00)	1.00 (0.99-1.00)
Right lateral ventricle	22.63 (8.89)	22.61 (8.89)	22.70 (9.07)	22.59 (8.89)	39.31	39.33	39.94	39.33	0.45 (0.66)	-1.09 (0.29)	0.72 (0.48)	-0.08 (0.74)	0.16 (0.84)	-0.12 (0.76)	0.64 (0.35)	0.52 (0.67)	0.58 (0.48)	1.00	1.00	1.00	1.00 (1.00-1.00)	1.00 (1.00-1.00)	1.00 (1.00-1.00)
Right inferior lateral ventricle	1.27 (0.98)	1.30 (1.01)	1.29 (1.00)	1.26 (0.97)	76.96	78.35	77.73	76.88	-1.89 (0.07)	-1.52 (0.14)	0.48 (0.64)	1.43 (5.36)	1.14 (3.74)	-0.50 (3.88)	4.34 (3.34)	2.83 (2.64)	2.95 (2.49)	1.00	1.00	1.00	1.00 (0.99-1.00)	1.00 (1.00-1.00)	1.00 (1.00-1.00)
Local cortical volume (ml)																							
Left banks superior temporal sulcus	1.82 (0.34)	1.82 (0.33)	1.82 (0.32)	1.80 (0.34)	18.48	18.33	17.64	18.70	0.15 (0.88)	0.00 (1.00)	1.13 (0.27)	-0.15 (4.61)	0.14 (4.56)	-1.17 (4.86)	3.25 (3.19)	3.40 (2.96)	4.05 (2.80)	0.97	0.97	0.97	0.97 (0.92-0.99)	0.97 (0.94-0.99)	0.97 (0.92-0.99)
Left caudal anterior cingulate cortex	1.51 (0.46)	1.56 (0.41)	1.54 (0.38)	1.55 (0.41)	30.44	26.34	24.73	26.42	-1.39 (0.18)	-0.96 (0.35)	-1.26 (0.22)	5.12 (18.68)	4.64 (18.24)	4.93 (19.05)	8.78 (17.19)	7.69 (17.13)	9.20 (17.30)	0.94	0.95	0.94	0.93 (0.85-0.97)	0.93 (0.85-0.97)	0.93 (0.84-0.97)
Left caudal middle frontal gyrus	4.97 (0.96)	4.94 (0.98)	4.94 (0.99)	4.94 (0.96)	19.40	19.87	19.93	19.43	1.09 (0.29)	0.87 (0.39)	0.80 (0.43)	-0.63 (2.32)	-0.54 (2.62)	-0.50 (2.97)	2.03 (1.23)	1.88 (1.86)	2.35 (1.81)	0.99	0.99	0.99	0.99 (0.98-1.00)	0.99 (0.98-1.00)	0.99 (0.98-1.00)
Left cuneus	2.77 (0.43)	2.77 (0.45)	2.78 (0.45)	2.76 (0.42)	15.69	16.13	16.05	15.34	0.25 (0.80)	-0.52 (0.61)	0.40 (0.70)	-0.19 (2.78)	0.29 (3.22)	-0.16 (2.90)	2.21 (1.62)	2.40 (2.10)	2.24 (1.78)	0.98	0.98	0.99	0.99 (0.96-0.99)	0.98 (0.96-0.99)	0.99 (0.97-0.99)
Left entorhinal cortex	1.48 (0.43)	1.50 (0.41)	1.57 (0.34)	1.56 (0.38)	29.03	27.29	21.87	24.21	-0.69 (0.50)	-1.82 (0.08)	-2.08 (0.05)	1.90 (8.85)	7.82 (17.36)	6.33 (13.44)	6.83 (5.76)	11.63 (14.96)	10.75 (10.07)	0.95	0.83	0.92	0.95 (0.90-0.98)	0.79 (0.56-0.91)	0.90 (0.76-0.96)
Left fusiform gyrus	7.86 (1.35)	7.92 (1.29)	7.87 (1.37)	7.93 (1.32)	17.19	16.30	17.42	16.62	-1.36 (0.19)	-0.20 (0.84)	-1.21 (0.24)	0.93 (3.16)	0.02 (3.82)	0.88 (3.92)	1.91 (2.66)	2.42 (2.91)	2.39 (3.19)	0.99	0.99	0.98	0.99 (0.97-0.99)	0.99 (0.97-0.99)	0.98 (0.96-0.99)
Left inferior parietal cortex	8.85 (2.06)	8.82 (2.00)	8.87 (2.11)	8.90 (2.07)	23.23	22.68	23.74	23.26	0.75 (0.46)	-0.28 (0.78)	-0.90 (0.38)	-0.19 (2.03)	0.03 (2.27)	0.56 (3.21)	1.66 (1.13)	1.88 (1.21)	2.55 (1.95)	1.00	0.99	0.99	1.00 (0.99-1.00)	0.99 (0.99-1.00)	0.99 (0.98-1.00)
Left inferior temporal gyrus	8.48 (1.31)	8.55 (1.40)	8.53 (1.36)	8.50 (1.31)	15.44	16.41	16.00	15.43	-1.59 (0.13)	-0.71 (0.49)	-0.54 (0.59)	0.72 (2.48)	0.41 (3.62)	0.27 (2.59)	2.13 (1.40)	3.00 (1.96)	2.05 (1.55)	0.99	0.98	0.99	0.99 (0.97-0.99)	0.98 (0.94-0.99)	0.99 (0.97-0.99)
Left isthmus cingulate cortex	2.26 (0.53)	2.24 (0.53)	2.26 (0.52)	2.30 (0.51)	23.39	23.71	23.07	22.39	0.74 (0.47)	0.15 (0.88)	-1.64 (0.12)	-0.87 (5.49)	-0.09 (4.30)	1.66 (4.82)	4.37 (3.30)	3.19 (2.80)	3.70 (3.42)	0.97	0.99	0.98	0.97 (0.94-0.99)	0.99 (0.97-1.00)	0.98 (0.95-0.99)
Left lateral occipital cortex	10.70 (1.86)	10.77 (1.92)	10.73 (1.80)	10.70 (1.88)	17.41	17.83	16.75	17.58	-1.55 (0.14)	-0.56 (0.58)	-0.16 (0.87)	0.58 (2.14)	0.42 (2.55)	0.01 (1.77)	1.59 (1.52)	1.97 (1.62)	1.32 (1.14)	0.99	0.99	1.00	0.99 (0.98-1.00)	0.99 (0.97-1.00)	1.00 (0.99-1.00)
Left lateral orbitofrontal cortex	6.64 (0.81)	6.62 (0.77)	6.60 (0.80)	6.65 (0.76)	12.23	11.67	12.17	11.50	0.50 (0.62)	0.84 (0.41)	-0.27 (0.79)	-0.18 (2.12)	-0.48 (2.61)	0.24 (2.65)	1.73 (1.18)	2.09 (1.57)	2.02 (1.68)	0.98	0.98	0.98	0.98 (0.96-0.99)	0.98 (0.94-0.99)	0.98 (0.94-0.99)
Left lingual gyrus	6.10 (1.07)	6.07 (1.10)	6.10 (1.08)	6.09 (1.05)	17.51	18.07	17.75	17.31	1.31 (0.20)	-0.18 (0.86)	0.34 (0.74)	-0.58 (1.83)	0.08 (2.11)	-0.08 (2.22)	1.55 (1.09)	1.58 (1.35)	1.58 (1.52)	1.00	0.99	0.99	0.99 (0.99-1.00)	0.99 (0.98-1.00)	0.99 (0.98-1.00)
Left medial orbitofrontal cortex	4.50 (0.52)	4.50 (0.57)	4.54 (0.50)	4.51 (0.49)	11.63	12.65	11.00	10.77	0.09 (0.93)	-1.02 (0.32)	-0.09 (0.93)	-0.22 (5.94)	0.83 (3.43)	0.20 (5.23)	4.05 (4.26)	3.03 (1.69)	3.68 (3.64)	0.88	0.95	0.90	0.88 (0.73-0.95)	0.95 (0.89-0.98)	0.90 (0.78-0.96)

Left middle temporal gyrus	8.42 (1.38)	8.35 (1.47)	8.31 (1.40)	8.33 (1.45)	16.41	17.55	16.80	17.38	1.50 (0.15)	2.68 (0.01)	1.99 (0.06)	-1.01 (2.98)	-1.40 (2.39)	-1.25 (2.82)	1.93 (2.46)	2.15 (1.71)	2.16 (2.16)	0.99	0.99	0.99	0.99 (0.97-1.00)	0.99 (0.96-1.00)	0.99 (0.97-1.00)
Left parahippocampal gyrus	1.69 (0.31)	1.71 (0.33)	1.70 (0.29)	1.71 (0.31)	18.24	19.14	17.28	17.84	-1.27 (0.22)	-0.41 (0.69)	-1.52 (0.14)	0.87 (4.10)	0.60 (5.13)	1.66 (5.05)	3.58 (2.06)	3.46 (3.77)	3.77 (3.69)	0.98	0.97	0.97	0.98 (0.95-0.99)	0.97 (0.93-0.99)	0.97 (0.92-0.99)
Left paracentral lobule	3.12 (0.39)	3.08 (0.38)	3.08 (0.36)	3.11 (0.39)	12.50	12.47	11.55	12.67	1.79 (0.09)	2.20 (0.04)	0.24 (0.81)	-1.25 (3.23)	-1.20 (2.83)	-0.18 (2.83)	2.45 (2.41)	2.58 (1.61)	2.19 (1.74)	0.97	0.98	0.97	0.96 (0.91-0.98)	0.97 (0.92-0.99)	0.98 (0.94-0.99)
Left pars opercularis	3.85 (0.51)	3.90 (0.51)	3.88 (0.55)	3.83 (0.50)	13.18	13.10	14.30	13.04	-1.91 (0.07)	-1.16 (0.26)	1.08 (0.29)	1.18 (2.76)	0.58 (2.98)	-0.44 (1.98)	2.29 (1.90)	2.21 (2.02)	1.70 (1.05)	0.98	0.98	0.99	0.97 (0.93-0.99)	0.98 (0.95-0.99)	0.99 (0.97-1.00)
Left pars orbitalis	2.15 (0.38)	2.10 (0.39)	2.13 (0.39)	2.10 (0.39)	17.58	18.52	18.11	18.70	2.40 (0.03)	1.34 (0.19)	2.99 (0.01)	-2.50 (4.44)	-1.09 (3.39)	-2.37 (3.54)	4.08 (2.96)	2.82 (2.10)	3.26 (2.70)	0.97	0.98	0.98	0.96 (0.90-0.99)	0.98 (0.96-0.99)	0.98 (0.91-0.99)
Left pars triangularis	3.15 (0.39)	3.12 (0.40)	3.15 (0.41)	3.14 (0.41)	12.52	12.87	12.89	13.16	1.77 (0.09)	0.05 (0.96)	0.59 (0.56)	-1.01 (2.65)	-0.09 (3.20)	-0.45 (3.17)	2.30 (1.61)	2.57 (1.82)	2.31 (2.16)	0.98	0.97	0.98	0.98 (0.95-0.99)	0.97 (0.93-0.99)	0.98 (0.94-0.99)
Left pericalcarine cortex	1.95 (0.34)	1.94 (0.36)	1.94 (0.36)	1.96 (0.36)	17.33	18.39	18.60	18.50	0.58 (0.57)	0.24 (0.81)	-0.52 (0.61)	-0.65 (4.01)	-0.42 (4.25)	0.21 (4.15)	3.44 (2.03)	3.41 (2.46)	2.96 (2.84)	0.98	0.98	0.98	0.98 (0.94-0.99)	0.97 (0.94-0.99)	0.98 (0.95-0.99)
Left postcentral gyrus	8.38 (1.10)	8.38 (1.08)	8.34 (1.12)	8.38 (1.10)	13.09	12.93	13.43	13.06	-0.04 (0.97)	1.65 (0.11)	-0.12 (0.90)	0.03 (1.39)	-0.56 (1.50)	0.03 (1.45)	0.98 (0.96)	1.35 (0.82)	1.07 (0.95)	0.99	0.99	0.99	0.99 (0.99-1.00)	0.99 (0.98-1.00)	0.99 (0.99-1.00)
Left posterior cingulate cortex	2.55 (0.59)	2.55 (0.56)	2.59 (0.57)	2.55 (0.59)	23.25	21.88	22.04	22.98	-0.03 (0.98)	-0.60 (0.55)	-0.22 (0.83)	0.30 (5.49)	1.81 (15.14)	0.37 (5.98)	3.84 (3.84)	7.00 (13.47)	4.17 (4.20)	0.98	0.86	0.98	0.98 (0.94-0.99)	0.86 (0.70-0.94)	0.98 (0.95-0.99)
Left precentral gyrus	11.99 (1.37)	11.98 (1.40)	11.97 (1.41)	11.97 (1.36)	11.38	11.66	11.80	11.40	0.22 (0.83)	0.46 (0.65)	0.49 (0.63)	-0.12 (1.76)	-0.21 (1.67)	-0.17 (1.56)	1.24 (1.22)	1.28 (1.04)	1.34 (0.78)	0.99	0.99	0.99	0.99 (0.98-1.00)	0.99 (0.98-1.00)	0.99 (0.98-1.00)
Left precuneus	7.85 (1.40)	7.86 (1.42)	7.82 (1.44)	7.82 (1.40)	17.77	18.13	18.43	17.85	-0.18 (0.86)	1.09 (0.29)	1.29 (0.21)	0.02 (1.69)	-0.54 (2.12)	-0.44 (1.65)	1.22 (1.14)	1.66 (1.39)	1.36 (0.99)	1.00	0.99	1.00	1.00 (0.99-1.00)	0.99 (0.99-1.00)	1.00 (0.99-1.00)
Left rostral anterior cingulate cortex	2.23 (0.62)	2.22 (0.49)	2.25 (0.55)	2.28 (0.59)	27.91	22.28	24.66	25.71	0.39 (0.70)	-0.60 (0.56)	-1.49 (0.15)	0.92 (10.07)	1.91 (9.44)	3.13 (10.36)	5.49 (8.41)	4.73 (8.34)	5.69 (9.14)	0.97	0.98	0.97	0.95 (0.88-0.98)	0.97 (0.94-0.99)	0.97 (0.93-0.99)
Left rostral middle frontal gyrus	12.86 (2.26)	12.90 (2.21)	12.92 (2.25)	12.87 (2.22)	17.58	17.15	17.44	17.25	-0.70 (0.49)	-0.97 (0.34)	-0.24 (0.81)	0.37 (2.07)	0.47 (2.03)	0.15 (2.20)	1.67 (1.22)	1.49 (1.43)	1.71 (1.34)	0.99	0.99	0.99	0.99 (0.98-1.00)	0.99 (0.98-1.00)	0.99 (0.98-1.00)
Left superior frontal gyrus	18.59 (2.82)	18.55 (2.72)	18.56 (2.78)	18.61 (2.80)	15.16	14.66	14.97	15.04	0.70 (0.49)	0.63 (0.54)	-0.42 (0.68)	-0.13 (1.16)	-0.14 (1.26)	0.13 (1.30)	0.93 (0.67)	0.94 (0.83)	1.04 (0.76)	1.00	1.00	1.00	1.00 (0.99-1.00)	1.00 (0.99-1.00)	1.00 (0.99-1.00)
Left superior parietal cortex	11.20 (2.12)	11.12 (2.04)	11.16 (2.11)	11.14 (2.08)	18.92	18.33	18.89	18.66	1.94 (0.07)	1.06 (0.30)	1.40 (0.18)	-0.66 (1.59)	-0.39 (1.60)	-0.53 (2.00)	1.37 (1.00)	1.23 (1.07)	1.74 (1.05)	1.00	1.00	1.00	0.99 (0.99-1.00)	1.00 (0.99-1.00)	0.99 (0.99-1.00)
Left superior temporal gyrus	10.18 (1.37)	10.22 (1.44)	10.19 (1.46)	10.26 (1.47)	13.50	14.06	14.33	14.36	-0.87 (0.40)	-0.24 (0.81)	-1.57 (0.13)	0.30 (2.04)	-0.02 (2.11)	0.69 (2.45)	1.59 (1.27)	1.61 (1.32)	1.99 (1.53)	0.99	0.99	0.99	0.99 (0.97-1.00)	0.99 (0.98-1.00)	0.98 (0.96-0.99)
Left supramarginal gyrus	9.05 (1.42)	9.10 (1.45)	9.04 (1.46)	9.08 (1.45)	15.66	15.89	16.12	15.97	-1.46 (0.16)	0.44 (0.66)	-0.68 (0.50)	0.46 (1.51)	-0.22 (1.68)	0.20 (1.85)	1.20 (1.00)	1.36 (0.95)	1.34 (1.27)	1.00	1.00	0.99	0.99 (0.99-1.00)	1.00 (0.99-1.00)	0.99 (0.98-1.00)
Left frontal pole	0.89 (0.16)	0.86 (0.13)	0.89 (0.13)	0.86 (0.15)	17.88	15.53	14.69	17.38	1.88 (0.07)	0.03 (0.98)	2.89 (0.01)	-2.44 (6.98)	0.42 (7.53)	-3.17 (5.34)	6.04 (4.09)	6.58 (3.40)	4.98 (3.62)	0.92	0.91	0.96	0.90 (0.77-0.96)	0.89 (0.76-0.95)	0.94 (0.81-0.98)
Left temporal pole	2.16 (0.30)	2.09 (0.29)	2.14 (0.33)	2.12 (0.27)	13.81	14.07	15.31	12.84	1.93 (0.07)	0.78 (0.44)	1.08 (0.29)	-3.50 (8.72)	-1.26 (6.51)	-1.66 (8.34)	7.19 (5.89)	5.54 (3.45)	6.04 (5.84)	0.81	0.90	0.83	0.80 (0.56-0.91)	0.90 (0.78-0.96)	0.83 (0.64-0.92)
Left transverse temporal gyrus	1.06 (0.17)	1.06 (0.18)	1.07 (0.16)	1.06 (0.17)	16.27	17.25	15.03	15.94	0.82 (0.42)	-0.33 (0.74)	0.73 (0.47)	-0.94 (4.59)	0.47 (4.50)	-0.50 (3.37)	3.28 (3.27)	3.76 (2.39)	2.67 (2.03)	0.97	0.96	0.98	0.97 (0.93-0.99)	0.96 (0.90-0.98)	0.98 (0.95-0.99)
Left insula	6.30 (0.80)	6.30 (0.83)	6.29 (0.75)	6.36 (0.86)	12.77	13.14	11.93	13.59	0.03 (0.97)	0.19 (0.85)	-1.09 (0.29)	-0.06 (5.09)	-0.03 (3.23)	0.83 (3.60)	3.20 (3.90)	2.21 (2.31)	2.57 (2.60)	0.91	0.96	0.96	0.91 (0.79-0.96)	0.96 (0.91-0.98)	0.95 (0.90-0.98)
Right banks superior temporal sulcus	1.73 (0.33)	1.73 (0.37)	1.71 (0.29)	1.74 (0.36)	19.31	21.48	17.07	20.74	-0.15 (0.88)	1.30 (0.21)	-0.37 (0.71)	0.09 (5.99)	-0.88 (4.96)	0.60 (7.56)	4.42 (3.93)	3.41 (3.64)	5.42 (5.17)	0.94	0.98	0.92	0.94 (0.85-0.97)	0.97 (0.92-0.99)	0.92 (0.81-0.97)
Right caudal anterior cingulate cortex	1.70 (0.35)	1.73 (0.35)	1.70 (0.35)	1.71 (0.37)	20.44	20.07	20.55	21.41	-1.28 (0.21)	0.77 (0.45)	-0.01 (0.99)	1.50 (5.91)	-0.44 (2.56)	-0.20 (6.62)	4.31 (4.23)	2.15 (1.39)	4.44 (4.82)	0.97	0.99	0.96	0.96 (0.92-0.99)	0.99 (0.98-1.00)	0.96 (0.90-0.98)
Right caudal middle frontal gyrus	4.78 (0.89)	4.78 (0.90)	4.82 (0.92)	4.86 (0.92)	18.66	18.83	19.19	19.00	0.10 (0.92)	-1.38 (0.18)	-2.93 (0.01)	-0.07 (2.06)	0.61 (2.54)	1.50 (2.74)	1.60 (1.24)	2.08 (1.52)	2.15 (2.25)	1.00	0.99	0.99	1.00 (0.99-1.00)	0.99 (0.98-1.00)	0.99 (0.96-1.00)
Right cuneus	3.12 (0.56)	3.13 (0.58)	3.10 (0.54)	3.12 (0.57)	18.08	18.61	17.34	18.35	-0.39 (0.70)	0.80 (0.43)	0.27 (0.79)	0.16 (2.34)	-0.49 (3.37)	-0.17 (2.94)	1.79 (1.46)	2.40 (2.37)	2.10 (2.01)	0.99	0.98	0.99	0.99 (0.98-1.00)	0.98 (0.95-0.99)	0.99 (0.97-1.00)
Right entorhinal cortex	1.49 (0.40)	1.46 (0.40)	1.46 (0.42)	1.56 (0.40)	26.56	27.39	28.98	25.56	0.63 (0.53)	0.56 (0.58)	-1.15 (0.26)	-1.77 (14.05)	-2.56 (18.67)	4.94 (21.35)	9.44 (10.36)	12.16 (14.16)	14.65 (16.01)	0.88	0.81	0.71	0.88 (0.74-0.95)	0.82 (0.61-0.92)	0.71 (0.43-0.87)
Right fusiform gyrus	7.62 (1.18)	7.67 (1.18)	7.69 (1.16)	7.68 (1.22)	15.54	15.33	15.08	15.89	-1.36 (0.19)	-1.66 (0.11)	-1.46 (0.16)	0.73 (2.57)	1.09 (3.04)	0.83 (3.13)	2.10 (1.60)	2.48 (2.00)	2.52 (1.97)	0.99	0.98	0.99	0.99 (0.97-1.00)	0.98 (0.96-0.99)	0.98 (0.96-0.99)
Right inferior parietal cortex	10.96 (2.45)	11.01 (2.56)	10.97 (2.52)	11.07 (2.54)	22.32	23.24	22.95	22.94	-0.74 (0.47)	-0.17 (0.87)	-1.64 (0.12)	0.30 (2.85)	-0.06 (2.05)	0.87 (2.66)	1.99 (2.02)	1.63 (1.21)	1.64 (2.25)	0.99	1.00	0.99	0.99 (0.98-1.00)	1.00 (0.99-1.00)	0.99 (0.98-1.00)
Right inferior temporal gyrus	8.20 (1.27)	8.21 (1.23)	8.25 (1.26)	8.24 (1.31)	15.52	15.04	15.23	15.85	-0.19 (0.85)	-0.77 (0.45)	-0.68 (0.51)	0.14 (3.49)	0.66 (4.25)	0.43 (3.89)	2.44 (2.45)	2.76 (3.25)	2.99 (2.44)	0.98	0.97	0.97	0.98 (0.95-0.99)	0.97 (0.92-0.99)	0.97 (0.94-0.99)
Right isthmus cingulate cortex	2.02 (0.51)	1.98 (0.48)	1.99 (0.53)	2.00 (0.53)	25.12	24.31	26.73	26.75	1.74 (0.10)	1.96 (0.06)	0.92 (0.37)	-1.77 (5.09)	-2.15 (5.04)	-1.32 (5.13)	3.71 (3.84)	3.59 (4.09)	3.32 (4.08)	0.98	0.99	0.99	0.98 (0.94-0.99)	0.99 (0.97-0.99)	0.98 (0.96-0.99)
Right lateral occipital cortex	10.60 (1.60)	10.67 (1.69)	10.64 (1.61)	10.69 (1.72)	15.07	15.84	15.14	16.06	-1.78 (0.09)	-0.61 (0.55)	-2.09 (0.05)	0.54 (1.68)	0.33 (2.63)	0.71 (1.79)	1.38 (1.06)	2.12 (1.52)	1.26 (1.45)	1.00	0.99	0.99	0.99 (0.98-1.00)	0.99 (0.97-0.99)	0.99 (0.98-1.00)
Right lateral orbitofrontal cortex	6.39 (0.77)	6.43 (0.73)	6.42 (0.73)	6.48 (0.76)	12.00	11.39	11.34	11.65	-0.87 (0.39)	-0.60 (0.55)	-1.87 (0.08)	0.66 (3.19)	0.47 (2.92)	1.47 (3.65)	2.46 (2.06)	2.16 (1.97)	3.07 (2.39)	0.96	0.97	0.95	0.96 (0.91-0.98)	0.96 (0.92-0.99)	0.95 (0.88-0.98)
Right lingual gyrus	6.64 (1.31)	6.59 (1.27)	6.63 (1.29)	6.61 (1.29)	19.73	19.30	19.41	19.50	0.94 (0.36)	0.38 (0.71)	1.32 (0.20)	-0.62 (3.07)	-0.15 (2.12)	-0.39 (1.85)	1.73 (2.59)	1.57 (1.38)	1.39 (1.25)	0.98	0.99	1.00	0.98 (0.96-0.99)	0.99 (0.98-1.00)	1.00 (0.99-1.00)
Right medial orbitofrontal cortex	4.68 (0.51)	4.65 (0.51)	4.68 (0.51)	4.67 (0.48)	10.86	10.92	11.00	10.19	0.43 (0.67)	-0.13 (0.90)	0.14 (0.89)	-0.42 (4.49)	0.06 (2.91)	-0.02 (3.79)	3.47 (2.78)	2.10 (1.96)	2.99 (2.24)	0.91	0.96	0.94	0.91 (0.79-0.96)	0.97 (0.92-0.99)	0.94 (0.86-0.97)
Right middle temporal gyrus	9.56 (1.56)	9.54 (1.56)	9.56 (1.57)	9.51 (1.62)	16.29	16.36	16.37	17.00	0.43 (0.67)	-0.07 (0.95)	0.95 (0.35)	-0.20 (1.83)	-0.01 (1.57)	-0.63 (2.38)	1.61 (0.84)	1.14 (1.05)	1.93 (1.47)	0.99	1.00	0.99	0.99 (0.98-1.00)	1.00 (0.99-1.00)	0.99 (0.98-1.00)
Right parahippocampal gyrus	1.65 (0.33)	1.66 (0.31)	1.65 (0.33)	1.67 (0.31)	19.98	18.55	20.05	18.51	-0.16 (0.87)	0.29 (0.78)	-0.79 (0.44)	0.59 (8.21)	-0.25 (5.84)	1.24 (6.02)	5.29 (6.20)	4.26 (3.90)	4.19 (4.41)	0.93	0.96	0.97	0.93 (0.84-0.97)	0.96 (0.90-0.98)	0.97 (0.93-0.99)
Right paracentral lobule	3.57 (0.57)	3.57 (0.59)	3.55 (0.58)	3.56 (0.60)	15.86	16.50	16.35	16.85	0.20 (0.85)	1.46 (0.16)	0.52 (0.61)	-0.21 (2.55)	-0.66 (2.01)	-0.38 (2.20)	2.24 (1.12)	1.57 (1.38)	1.76 (1.32)	0.99	0.99	0.99	0.99 (0.97-1.00)	0.99 (0.98-1.00)	0.99 (0.98-1.00)
Right pars opercularis	3.24 (0.55)	3.27 (0.63)	3.24 (0.56)	3.28 (0.60)	17.03	19.18	17.20	18.20	-1.08 (0.29)	0.09 (0.93)	-1.57 (0.13)	0.67 (3.93)	-0.11 (2.90)	1.00 (3.45)	3.02 (2.53)	2.00 (2.06)	2.48 (2.56)	0.98	0.99	0.98	0.97 (0.93-0.99)	0.99 (0.97-1.00)	0.98 (0.95-0.99)
Right pars orbitalis	2.38 (0.45)	2.37 (0.45)	2.39 (0.44)	2.38 (0.45)	18.75	18.81	18.50	18.73	1.06 (0.30)	-0.45 (0.66)	0.35 (0.73)	-0.56 (2.56)	0.36 (3.03)	-0.21 (3.34)	2.12 (1.47)	2.39 (1.82)	2.69 (1.90)	0.99	0.99	0.99	0.99 (0.98-1.00)	0.99 (0.97-0.99)	0.99 (0.97-0.99)

Right pars triangularis	3.52 (0.58)	3.51 (0.59)	3.52 (0.56)	3.52 (0.57)	16.49	16.88	15.94	16.28	0.52 (0.61)	0.10 (0.92)	0.19 (0.85)	-0.36 (2.49)	0.02 (3.13)	-0.10 (2.82)	2.01 (1.45)	2.20 (2.18)	2.25 (1.63)	0.99	0.98	0.98	0.99 (0.98-1.00)	0.98 (0.96-0.99)	0.98 (0.96-0.99)
Right pericalcarine cortex	2.24 (0.47)	2.22 (0.47)	2.21 (0.47)	2.22 (0.44)	21.14	21.04	21.04	19.94	1.38 (0.18)	1.52 (0.14)	1.12 (0.28)	-0.66 (2.17)	-1.17 (3.30)	-0.49 (2.86)	1.88 (1.20)	2.59 (2.30)	2.31 (1.68)	0.99	0.98	0.99	0.99 (0.98-1.00)	0.98 (0.96-0.99)	0.99 (0.97-1.00)
Right postcentral gyrus	8.03 (1.11)	8.03 (1.14)	7.96 (1.13)	8.04 (1.16)	13.87	14.24	14.22	14.41	0.18 (0.86)	2.94 (0.01)	-0.30 (0.77)	-0.11 (1.32)	-0.92 (1.44)	0.02 (1.78)	1.01 (0.83)	1.39 (0.96)	1.43 (1.01)	1.00	1.00	0.99	1.00 (0.99-1.00)	0.99 (0.98-1.00)	0.99 (0.98-1.00)
Right posterior cingulate cortex	2.51 (0.62)	2.53 (0.62)	2.49 (0.65)	2.50 (0.66)	24.64	24.37	25.96	26.20	-0.43 (0.67)	0.86 (0.40)	0.05 (0.96)	0.93 (9.87)	-1.18 (5.50)	-0.43 (12.66)	5.76 (7.97)	3.45 (4.38)	6.77 (10.60)	0.93	0.99	0.90	0.93 (0.85-0.97)	0.99 (0.97-0.99)	0.90 (0.77-0.96)
Right precentral gyrus	12.15 (1.86)	12.10 (1.82)	12.06 (1.80)	12.13 (1.89)	15.32	15.04	14.90	15.58	1.39 (0.18)	1.82 (0.08)	0.36 (0.72)	-0.39 (1.43)	-0.63 (1.63)	-0.18 (1.60)	1.32 (0.61)	1.41 (1.00)	1.23 (1.01)	1.00	0.99	0.99	1.00 (0.99-1.00)	0.99 (0.98-1.00)	0.99 (0.99-1.00)
Right precuneus	8.29 (1.38)	8.23 (1.35)	8.23 (1.39)	8.25 (1.41)	16.68	16.44	16.87	17.07	1.90 (0.07)	2.57 (0.02)	1.53 (0.14)	-0.63 (1.53)	-0.70 (1.27)	-0.52 (1.35)	1.09 (1.22)	1.07 (0.96)	1.09 (0.92)	1.00	1.00	1.00	0.99 (0.99-1.00)	1.00 (0.99-1.00)	1.00 (0.99-1.00)
Right rostral anterior cingulate cortex	1.82 (0.42)	1.82 (0.43)	1.81 (0.43)	1.83 (0.43)	22.83	23.80	23.52	23.45	-0.16 (0.87)	0.65 (0.52)	-0.53 (0.60)	-0.12 (3.69)	-0.82 (4.63)	0.27 (3.58)	2.97 (2.10)	3.66 (2.86)	2.55 (2.47)	0.99	0.97	0.99	0.99 (0.97-0.99)	0.97 (0.94-0.99)	0.99 (0.97-0.99)
Right rostral middle frontal gyrus	13.15 (1.59)	13.15 (1.59)	13.12 (1.50)	13.10 (1.58)	12.11	12.05	11.47	12.03	0.01 (0.99)	0.81 (0.42)	1.16 (0.26)	0.00 (2.07)	-0.19 (1.48)	-0.39 (1.74)	1.55 (1.34)	1.20 (0.85)	1.23 (1.27)	0.98	0.99	0.99	0.99 (0.97-0.99)	0.99 (0.98-1.00)	0.99 (0.98-1.00)
Right superior frontal gyrus	18.68 (3.10)	18.62 (2.98)	18.70 (3.04)	18.73 (3.08)	16.60	15.98	16.24	16.43	1.06 (0.30)	-0.41 (0.69)	-1.06 (0.30)	-0.20 (1.36)	0.19 (1.49)	0.32 (1.36)	1.12 (0.76)	1.04 (1.06)	1.11 (0.81)	1.00	1.00	1.00	1.00 (0.99-1.00)	1.00 (0.99-1.00)	1.00 (0.99-1.00)
Right superior parietal cortex	11.04 (1.86)	11.04 (1.87)	11.05 (1.93)	11.12 (1.92)	16.89	16.96	17.45	17.26	0.15 (0.88)	-0.14 (0.89)	-2.76 (0.01)	-0.05 (1.37)	-0.05 (2.72)	0.63 (1.02)	1.12 (0.75)	1.78 (2.02)	0.93 (0.75)	1.00	0.99	1.00	1.00 (0.99-1.00)	0.99 (0.98-1.00)	1.00 (0.99-1.00)
Right superior temporal gyrus	9.85 (1.19)	9.79 (1.26)	9.86 (1.24)	9.76 (1.22)	12.09	12.84	12.61	12.51	1.32 (0.20)	-0.25 (0.80)	1.92 (0.07)	-0.69 (2.21)	0.08 (2.58)	-0.99 (2.58)	1.74 (1.50)	1.97 (1.61)	1.76 (2.10)	0.99	0.98	0.98	0.99 (0.96-0.99)	0.98 (0.95-0.99)	0.98 (0.95-0.99)
Right supramarginal gyrus	8.33 (1.20)	8.36 (1.20)	8.34 (1.19)	8.37 (1.19)	14.46	14.29	14.30	14.19	-1.14 (0.27)	-0.46 (0.65)	-1.38 (0.18)	0.48 (1.90)	0.18 (1.65)	0.58 (1.79)	1.67 (0.96)	1.13 (1.19)	1.52 (1.08)	0.99	0.99	0.99	0.99 (0.98-1.00)	0.99 (0.99-1.00)	0.99 (0.98-1.00)
Right frontal pole	1.02 (0.11)	1.01 (0.11)	1.05 (0.13)	1.00 (0.12)	10.35	10.94	12.06	12.39	0.71 (0.49)	-2.05 (0.05)	1.65 (0.11)	-0.96 (5.93)	2.53 (6.26)	-2.19 (5.65)	4.46 (3.92)	5.50 (3.78)	4.41 (4.07)	0.84	0.86	0.89	0.84 (0.66-0.93)	0.83 (0.61-0.93)	0.87 (0.71-0.94)
Right temporal pole	2.23 (0.32)	2.17 (0.34)	2.16 (0.35)	2.23 (0.32)	14.42	15.79	16.03	14.09	1.73 (0.10)	1.73 (0.10)	-0.32 (0.75)	-2.78 (7.36)	-3.43 (8.78)	0.36 (5.90)	5.72 (5.29)	7.30 (5.80)	4.93 (3.08)	0.90	0.84	0.93	0.89 (0.74-0.95)	0.83 (0.63-0.93)	0.93 (0.84-0.97)
Right transverse temporal gyrus	0.81 (0.18)	0.80 (0.16)	0.80 (0.16)	0.81 (0.17)	21.79	20.60	20.09	20.79	0.73 (0.47)	0.28 (0.78)	-0.93 (0.36)	-0.56 (5.09)	-0.02 (4.10)	1.12 (4.41)	4.00 (3.08)	3.49 (2.02)	3.98 (2.03)	0.97	0.98	0.98	0.97 (0.93-0.99)	0.98 (0.95-0.99)	0.98 (0.95-0.99)
Right insula	6.44 (0.85)	6.41 (0.91)	6.39 (0.82)	6.38 (0.94)	13.23	14.27	12.81	14.66	0.48 (0.63)	0.94 (0.36)	0.80 (0.43)	-0.55 (3.93)	-0.68 (3.44)	-1.04 (5.05)	2.92 (2.61)	2.90 (1.88)	4.18 (2.88)	0.96	0.96	0.94	0.96 (0.91-0.98)	0.96 (0.91-0.98)	0.94 (0.86-0.97)
Local cortical surface (cm²)																							
Left banks superior temporal sulcus	7.92 (1.20)	7.94 (1.11)	7.98 (1.13)	7.94 (1.16)	15.09	13.94	14.13	14.59	-0.21 (0.83)	-1.06 (0.30)	-0.22 (0.83)	0.38 (4.89)	0.89 (3.79)	0.25 (4.27)	3.39 (3.47)	2.60 (2.85)	2.82 (3.17)	0.95	0.97	0.96	0.95 (0.88-0.98)	0.97 (0.94-0.99)	0.96 (0.92-0.99)
Left caudal anterior cingulate cortex	5.70 (1.54)	5.84 (1.34)	5.80 (1.29)	5.86 (1.39)	26.93	23.00	22.33	23.63	-1.16 (0.26)	-0.78 (0.44)	-1.29 (0.21)	3.84 (15.15)	3.29 (14.94)	4.13 (15.30)	8.63 (12.92)	7.52 (13.24)	7.89 (13.67)	0.93	0.94	0.93	0.92 (0.83-0.97)	0.93 (0.83-0.97)	0.92 (0.82-0.97)
Left caudal middle frontal gyrus	19.09 (3.23)	19.04 (3.34)	19.07 (3.23)	19.06 (3.30)	16.93	17.56	16.93	17.34	0.57 (0.58)	0.20 (0.84)	0.38 (0.71)	-0.35 (2.06)	-0.08 (1.92)	-0.23 (2.27)	1.55 (1.36)	1.38 (1.30)	1.84 (1.29)	0.99	0.99	0.99	0.99 (0.99-1.00)	0.99 (0.99-1.00)	0.99 (0.98-1.00)
Left cuneus	14.22 (1.96)	14.27 (2.06)	14.29 (2.01)	14.25 (2.01)	13.77	14.43	14.05	14.12	-0.58 (0.56)	-0.76 (0.46)	-0.52 (0.61)	0.28 (2.84)	0.44 (3.04)	0.18 (1.98)	2.40 (1.46)	2.26 (2.02)	1.66 (1.04)	0.98	0.98	0.99	0.98 (0.95-0.99)	0.98 (0.95-0.99)	0.99 (0.98-1.00)
Left entorhinal cortex	3.76 (0.90)	3.85 (0.89)	4.09 (0.83)	3.92 (0.74)	23.94	23.19	20.38	19.00	-0.89 (0.38)	-2.54 (0.02)	-1.80 (0.09)	2.75 (12.94)	9.07 (16.46)	5.13 (12.25)	10.29 (8.04)	11.57 (14.73)	7.91 (10.59)	0.86	0.75	0.89	0.86 (0.70-0.94)	0.71 (0.37-0.87)	0.86 (0.69-0.94)
Left fusiform gyrus	27.11 (4.38)	27.60 (4.34)	27.08 (4.30)	27.36 (4.27)	16.16	15.73	15.88	15.60	-3.00 (0.01)	0.31 (0.76)	-1.84 (0.08)	1.86 (3.16)	-0.08 (2.02)	1.00 (2.44)	2.65 (2.49)	1.66 (1.09)	1.94 (1.75)	0.99	0.99	0.99	0.98 (0.93-0.99)	0.99 (0.98-1.00)	0.99 (0.97-1.00)
Left inferior parietal cortex	36.43 (6.86)	36.40 (6.99)	36.41 (6.99)	36.74 (6.74)	18.83	19.19	19.19	18.34	0.23 (0.82)	0.16 (0.87)	-1.79 (0.09)	-0.15 (1.84)	-0.12 (1.75)	0.94 (2.38)	1.44 (1.10)	1.43 (0.97)	2.01 (1.53)	1.00	1.00	0.99	1.00 (0.99-1.00)	1.00 (0.99-1.00)	0.99 (0.98-1.00)
Left inferior temporal gyrus	27.05 (4.21)	27.59 (4.15)	27.33 (4.21)	27.42 (4.00)	15.58	15.04	15.40	14.57	-3.17 (0.00)	-2.20 (0.04)	-2.71 (0.01)	2.13 (3.43)	1.09 (2.24)	1.56 (2.59)	2.72 (2.96)	1.95 (1.52)	2.30 (1.94)	0.98	0.99	0.99	0.97 (0.90-0.99)	0.99 (0.97-1.00)	0.98 (0.95-0.99)
Left isthmus cingulate cortex	9.65 (1.85)	9.77 (1.80)	9.76 (1.79)	9.87 (1.76)	19.21	18.42	18.30	17.79	-1.09 (0.29)	-1.26 (0.22)	-2.12 (0.05)	1.35 (5.31)	1.27 (4.38)	2.48 (5.23)	3.95 (3.71)	3.30 (3.07)	3.91 (4.22)	0.96	0.98	0.97	0.96 (0.91-0.98)	0.98 (0.94-0.99)	0.96 (0.89-0.98)
Left lateral occipital cortex	46.43 (6.52)	46.89 (6.76)	46.73 (6.24)	46.73 (6.53)	14.03	14.41	13.36	13.97	-3.05 (0.01)	-1.79 (0.09)	-1.98 (0.06)	0.91 (1.43)	0.72 (1.63)	0.63 (1.47)	1.39 (0.96)	1.52 (0.89)	1.31 (0.87)	1.00	0.99	0.99	0.99 (0.97-1.00)	0.99 (0.98-1.00)	0.99 (0.98-1.00)
Left lateral orbitofrontal cortex	24.31 (3.43)	24.51 (3.40)	24.23 (3.51)	24.43 (3.41)	14.13	13.87	14.47	13.96	-2.02 (0.06)	0.81 (0.43)	-1.63 (0.12)	0.86 (1.94)	-0.36 (1.80)	0.52 (1.43)	1.49 (1.49)	1.35 (1.22)	1.18 (0.94)	0.99	0.99	0.99	0.99 (0.97-1.00)	0.99 (0.98-1.00)	0.99 (0.99-1.00)
Left lingual gyrus	29.55 (4.40)	29.50 (4.68)	29.60 (4.56)	29.49 (4.36)	14.88	15.86	15.40	14.80	0.36 (0.72)	-0.40 (0.69)	0.50 (0.62)	-0.31 (2.22)	0.08 (1.75)	-0.18 (1.83)	1.94 (1.05)	1.36 (1.06)	1.48 (1.06)	0.99	0.99	0.99	0.99 (0.97-1.00)	0.99 (0.98-1.00)	0.99 (0.98-1.00)
Left medial orbitofrontal cortex	18.44 (2.73)	18.51 (2.63)	18.54 (2.33)	18.38 (2.56)	14.78	14.22	12.54	13.94	-0.21 (0.84)	-0.49 (0.63)	0.20 (0.84)	0.42 (8.43)	0.82 (5.03)	-0.21 (7.84)	6.19 (5.57)	4.18 (2.78)	5.50 (5.47)	0.84	0.94	0.86	0.84 (0.66-0.93)	0.93 (0.85-0.97)	0.87 (0.71-0.94)
Left middle temporal gyrus	26.16 (4.24)	26.38 (4.29)	25.95 (4.23)	26.22 (4.28)	16.21	16.28	16.29	16.33	-1.90 (0.07)	1.68 (0.11)	-0.53 (0.60)	0.86 (2.24)	-0.79 (2.16)	0.22 (2.10)	1.94 (1.35)	1.88 (1.26)	1.66 (1.26)	0.99	0.99	0.99	0.99 (0.98-1.00)	0.99 (0.98-1.00)	0.99 (0.98-1.00)
Left parahippocampal gyrus	5.79 (0.75)	5.88 (0.72)	5.84 (0.63)	5.91 (0.78)	12.96	12.18	10.75	13.19	-1.54 (0.14)	-1.02 (0.32)	-2.40 (0.03)	1.60 (4.87)	1.13 (4.43)	2.06 (3.89)	3.81 (3.34)	3.21 (3.18)	3.13 (3.04)	0.93	0.96	0.95	0.93 (0.84-0.97)	0.94 (0.87-0.98)	0.94 (0.85-0.98)
Left paracentral lobule	12.51 (1.65)	12.44 (1.64)	12.50 (1.57)	12.49 (1.62)	13.16	13.18	12.53	12.96	1.23 (0.23)	0.33 (0.75)	0.65 (0.52)	-0.57 (2.15)	-0.05 (1.81)	-0.20 (1.60)	1.97 (0.96)	1.62 (0.74)	1.24 (1.00)	0.99	0.99	0.99	0.99 (0.97-0.99)	0.99 (0.98-1.00)	0.99 (0.98-1.00)
Left pars opercularis	13.78 (2.05)	13.99 (2.09)	13.88 (2.21)	13.82 (2.02)	14.89	14.94	15.91	14.64	-2.35 (0.03)	-0.88 (0.39)	-0.56 (0.58)	1.46 (2.77)	0.50 (3.47)	0.29 (2.12)	2.45 (1.90)	2.51 (2.38)	1.78 (1.13)	0.98	0.98	0.99	0.98 (0.93-0.99)	0.97 (0.94-0.99)	0.99 (0.97-1.00)
Left pars orbitalis	6.31 (1.18)	6.30 (1.24)	6.30 (1.20)	6.30 (1.23)	18.68	19.70	18.99	19.59	0.35 (0.73)	0.26 (0.80)	0.39 (0.70)	-0.46 (3.84)	-0.26 (3.64)	-0.40 (3.24)	3.01 (2.34)	2.63 (2.46)	2.30 (2.27)	0.98	0.98	0.99	0.98 (0.96-0.99)	0.98 (0.96-0.99)	0.99 (0.97-1.00)
Left pars triangularis	12.07 (1.70)	12.07 (1.80)	12.13 (1.83)	12.11 (1.74)	14.07	14.92	15.13	14.38	-0.02 (0.98)	-0.59 (0.56)	-0.63 (0.54)	-0.10 (2.53)	0.32 (3.54)	0.32 (2.92)	2.13 (1.28)	2.87 (2.00)	2.28 (1.78)	0.99	0.97	0.98	0.99 (0.96-0.99)	0.97 (0.93-0.99)	0.98 (0.96-0.99)
Left pericalcarine cortex	13.47 (2.36)	13.53 (2.44)	13.51 (2.45)	13.49 (2.51)	17.54	18.01	18.13	18.57	-0.77 (0.45)	-0.51 (0.62)	-0.30 (0.77)	0.35 (2.56)	0.19 (2.57)	-0.03 (2.84)	2.16 (1.34)	1.97 (1.60)	2.05 (1.91)	0.99	0.99	0.99	0.99 (0.98-1.00)	0.99 (0.97-1.00)	0.99 (0.97-1.00)
Left postcentral gyrus	38.10 (5.22)	38.12 (5.26)	38.05 (5.34)	38.17 (5.24)	13.71	13.81	14.04	13.74	-0.18 (0.86)	0.66 (0.52)	-0.72 (0.48)	0.04 (1.12)	-0.18 (0.97)	0.19 (1.38)	0.87 (0.69)	0.76 (0.61)	0.95 (1.01)	1.00	1.00	1.00	1.00 (0.99-1.00)	1.00 (0.99-1.00)	1.00 (0.99-1.00)
Left posterior cingulate cortex	10.40 (2.29)	10.42 (2.12)	10.52 (2.20)	10.48 (2.27)	22.03	20.29	20.93	21.68	-0.33 (0.75)	-0.75 (0.46)	-0.96 (0.35)	0.63 (3.70)	1.49 (9.61)	0.97 (4.12)	2.75 (2.49)	4.72 (8.45)	3.11 (2.79)	0.99	0.95	0.98	0.99 (0.97-1.00)	0.95 (0.88-0.98)	0.98 (0.96-0.99)

Left precentral gyrus	45.54 (6.12)	45.41 (6.33)	45.46 (6.43)	45.51 (6.39)	13.44	13.93	14.14	14.03	1.07 (0.30)	0.57 (0.57)	0.22 (0.82)	-0.35 (1.35)	-0.26 (1.47)	-0.14 (1.40)	0.98 (0.98)	1.07 (1.02)	1.09 (0.86)	1.00	1.00	1.00	1.00 (0.99-1.00)	1.00 (0.99-1.00)	1.00 (0.99-1.00)
Left precuneus	33.59 (5.27)	33.60 (5.27)	33.55 (5.15)	33.54 (5.24)	15.70	15.68	15.36	15.62	-0.11 (0.91)	0.37 (0.71)	0.50 (0.62)	0.04 (1.38)	-0.06 (1.53)	-0.12 (1.34)	1.12 (0.76)	1.18 (0.94)	1.13 (0.70)	1.00	1.00	1.00	1.00 (0.99-1.00)	1.00 (0.99-1.00)	1.00 (0.99-1.00)
Left rostral anterior cingulate cortex	7.89 (2.22)	7.84 (1.91)	7.90 (2.10)	8.01 (2.28)	28.14	24.40	26.56	28.45	0.45 (0.66)	-0.23 (0.82)	-0.95 (0.35)	0.33 (6.11)	0.78 (5.40)	1.88 (7.50)	4.22 (4.34)	3.90 (3.73)	5.36 (5.47)	0.98	0.99	0.97	0.97 (0.93-0.99)	0.99 (0.97-0.99)	0.97 (0.92-0.99)
Left rostral middle frontal gyrus	49.62 (9.21)	49.95 (9.06)	49.74 (9.06)	50.14 (8.91)	18.57	18.14	18.22	17.78	-2.28 (0.03)	-0.99 (0.33)	-3.09 (0.01)	0.73 (1.41)	0.30 (1.18)	1.20 (1.81)	1.23 (0.98)	0.91 (0.78)	1.59 (1.45)	1.00	1.00	1.00	1.00 (0.99-1.00)	1.00 (1.00-1.00)	0.99 (0.98-1.00)
Left superior frontal gyrus	64.77 (11.18)	65.08 (11.41)	64.94 (11.23)	65.06 (11.14)	17.26	17.53	17.29	17.12	-2.20 (0.04)	-1.36 (0.19)	-2.93 (0.01)	0.43 (0.92)	0.26 (0.97)	0.46 (0.76)	0.83 (0.56)	0.77 (0.62)	0.67 (0.58)	1.00	1.00	1.00	1.00 (0.99-1.00)	1.00 (1.00-1.00)	1.00 (1.00-1.00)
Left superior parietal cortex	48.82 (8.05)	48.72 (7.95)	48.82 (7.97)	48.86 (7.91)	16.48	16.33	16.32	16.19	0.96 (0.35)	0.01 (0.99)	-0.32 (0.75)	-0.18 (1.01)	0.02 (1.07)	0.14 (1.48)	0.78 (0.64)	0.85 (0.62)	1.08 (0.99)	1.00	1.00	1.00	1.00 (1.00-1.00)	1.00 (1.00-1.00)	1.00 (0.99-1.00)
Left superior temporal gyrus	36.03 (3.89)	36.15 (4.05)	35.93 (4.01)	36.19 (4.00)	10.78	11.20	11.17	11.06	-0.90 (0.38)	0.86 (0.40)	-1.46 (0.16)	0.29 (1.87)	-0.32 (1.50)	0.41 (1.47)	1.34 (1.30)	1.12 (1.03)	1.18 (0.93)	0.99	0.99	0.99	0.99 (0.97-0.99)	0.99 (0.98-1.00)	0.99 (0.98-1.00)
Left supramarginal gyrus	35.50 (5.38)	35.55 (5.46)	35.58 (5.49)	35.74 (5.47)	15.15	15.37	15.43	15.31	-0.36 (0.72)	-0.60 (0.55)	-1.86 (0.08)	0.12 (1.85)	0.19 (1.88)	0.65 (1.70)	1.39 (1.18)	1.46 (1.17)	1.37 (1.16)	0.99	0.99	0.99	0.99 (0.98-1.00)	0.99 (0.98-1.00)	0.99 (0.98-1.00)
Left frontal pole	2.44 (0.31)	2.40 (0.29)	2.44 (0.27)	2.39 (0.29)	12.61	11.95	11.08	12.29	0.87 (0.40)	-0.14 (0.89)	1.53 (0.14)	-1.44 (8.41)	0.43 (8.40)	-2.01 (6.16)	7.04 (4.58)	7.02 (4.37)	5.29 (3.59)	0.77	0.76	0.88	0.77 (0.53-0.90)	0.76 (0.50-0.89)	0.87 (0.71-0.94)
Left temporal pole	4.37 (0.63)	4.30 (0.58)	4.40 (0.73)	4.36 (0.65)	14.43	13.40	16.61	14.93	1.04 (0.31)	-0.49 (0.63)	0.22 (0.83)	-1.40 (6.99)	0.39 (5.99)	-0.32 (6.41)	5.88 (3.83)	4.83 (3.40)	4.87 (4.05)	0.88	0.93	0.91	0.87 (0.72-0.95)	0.93 (0.83-0.97)	0.91 (0.80-0.96)
Left transverse temporal gyrus	4.26 (0.56)	4.28 (0.61)	4.29 (0.54)	4.26 (0.54)	13.16	14.28	12.50	12.73	-0.78 (0.45)	-0.72 (0.48)	-0.03 (0.97)	0.49 (4.16)	0.79 (4.53)	0.10 (4.24)	3.22 (2.58)	3.75 (2.54)	3.22 (2.66)	0.96	0.94	0.94	0.96 (0.91-0.98)	0.94 (0.85-0.97)	0.94 (0.87-0.98)
Left insula	23.49 (2.80)	23.57 (2.78)	23.18 (2.56)	23.65 (2.82)	11.94	11.77	11.03	11.92	-0.21 (0.84)	1.18 (0.25)	-0.47 (0.64)	0.39 (7.47)	-1.22 (5.17)	0.70 (6.78)	5.58 (4.83)	3.52 (3.92)	4.96 (4.55)	0.78	0.90	0.82	0.79 (0.55-0.91)	0.89 (0.76-0.95)	0.83 (0.63-0.93)
Right banks superior temporal sulcus	7.42 (1.30)	7.50 (1.51)	7.39 (1.15)	7.50 (1.36)	17.50	20.09	15.49	18.10	-0.65 (0.52)	0.52 (0.61)	-0.84 (0.41)	0.77 (5.60)	-0.05 (3.84)	1.07 (5.32)	3.94 (3.96)	2.90 (2.44)	3.62 (3.97)	0.94	0.98	0.95	0.93 (0.84-0.97)	0.98 (0.95-0.99)	0.95 (0.88-0.98)
Right caudal anterior cingulate cortex	6.37 (1.41)	6.47 (1.41)	6.40 (1.38)	6.39 (1.43)	22.06	21.84	21.58	22.36	-1.39 (0.18)	-0.67 (0.51)	-0.20 (0.84)	1.56 (5.45)	0.56 (3.24)	0.19 (5.38)	4.24 (3.67)	2.40 (2.19)	4.01 (3.48)	0.97	0.99	0.97	0.97 (0.93-0.99)	0.99 (0.97-1.00)	0.97 (0.93-0.99)
Right caudal middle frontal gyrus	18.25 (3.33)	18.29 (3.40)	18.39 (3.35)	18.45 (3.48)	18.23	18.58	18.24	18.84	-0.43 (0.67)	-1.53 (0.14)	-2.16 (0.04)	0.15 (2.63)	0.76 (2.40)	1.00 (2.50)	2.12 (1.50)	1.84 (1.68)	1.93 (1.84)	0.99	0.99	0.99	0.99 (0.98-1.00)	0.99 (0.98-1.00)	0.99 (0.97-1.00)
Right cuneus	15.64 (2.34)	15.68 (2.32)	15.48 (2.08)	15.63 (2.32)	14.95	14.81	13.44	14.82	-0.70 (0.49)	1.25 (0.22)	0.07 (0.94)	0.25 (1.63)	-0.86 (3.34)	-0.01 (2.55)	1.24 (1.06)	2.15 (2.66)	2.05 (1.46)	0.99	0.97	0.99	0.99 (0.99-1.00)	0.96 (0.91-0.98)	0.99 (0.97-0.99)
Right entorhinal cortex	3.48 (0.65)	3.48 (0.75)	3.45 (0.67)	3.54 (0.79)	18.76	21.54	19.50	22.28	-0.02 (0.98)	0.53 (0.60)	-0.69 (0.50)	-0.41 (7.92)	-1.13 (9.67)	1.17 (12.23)	6.39 (4.49)	7.33 (6.21)	8.46 (8.72)	0.94	0.89	0.83	0.93 (0.85-0.97)	0.89 (0.77-0.95)	0.82 (0.62-0.92)
Right fusiform gyrus	26.51 (3.86)	26.73 (3.89)	26.67 (3.90)	26.73 (4.03)	14.57	14.56	14.61	15.07	-2.26 (0.03)	-1.64 (0.12)	-1.70 (0.10)	0.84 (1.71)	0.60 (1.79)	0.77 (2.26)	1.49 (1.15)	1.51 (1.09)	1.98 (1.28)	0.99	0.99	0.99	0.99 (0.98-1.00)	0.99 (0.98-1.00)	0.99 (0.97-0.99)
Right inferior parietal cortex	43.73 (8.27)	43.93 (8.65)	43.73 (8.31)	44.30 (8.40)	18.91	19.70	18.99	18.97	-0.77 (0.45)	-0.03 (0.98)	-3.19 (0.00)	0.35 (2.41)	-0.01 (1.60)	1.28 (1.70)	1.62 (1.79)	1.23 (0.98)	1.54 (1.45)	0.99	1.00	1.00	0.99 (0.98-1.00)	1.00 (0.99-1.00)	0.99 (0.97-1.00)
Right inferior temporal gyrus	26.40 (4.45)	26.43 (4.20)	26.68 (4.22)	26.54 (4.30)	16.84	15.88	15.82	16.21	-0.15 (0.88)	-1.34 (0.20)	-0.64 (0.53)	0.18 (3.33)	1.15 (3.59)	0.55 (3.62)	2.42 (2.24)	2.66 (2.62)	2.99 (2.01)	0.98	0.98	0.97	0.98 (0.94-0.99)	0.97 (0.94-0.99)	0.97 (0.94-0.99)
Right isthmus cingulate cortex	8.54 (1.70)	8.54 (1.70)	8.47 (1.93)	8.56 (1.88)	19.94	19.95	22.76	21.97	0.04 (0.97)	0.75 (0.46)	-0.21 (0.84)	-0.07 (4.18)	-1.47 (6.42)	-0.25 (5.75)	3.14 (2.67)	3.47 (5.56)	3.97 (4.08)	0.98	0.98	0.97	0.98 (0.96-0.99)	0.97 (0.94-0.99)	0.97 (0.93-0.99)
Right lateral occipital cortex	45.69 (5.48)	45.80 (5.52)	45.84 (5.39)	46.03 (5.62)	12.00	12.05	11.75	12.21	-0.53 (0.60)	-1.15 (0.26)	-2.84 (0.01)	0.22 (1.89)	0.35 (1.37)	0.71 (1.21)	1.38 (1.28)	1.00 (0.97)	1.05 (0.92)	0.99	0.99	1.00	0.99 (0.97-1.00)	0.99 (0.99-1.00)	0.99 (0.98-1.00)
Right lateral orbitofrontal cortex	24.43 (3.24)	24.80 (3.30)	24.71 (3.36)	24.90 (3.35)	13.25	13.30	13.60	13.47	-1.62 (0.12)	-1.11 (0.28)	-1.26 (0.22)	1.50 (4.19)	1.08 (4.47)	1.89 (7.32)	3.31 (2.92)	3.25 (3.18)	5.62 (4.92)	0.95	0.94	0.86	0.94 (0.86-0.98)	0.94 (0.86-0.97)	0.86 (0.69-0.94)
Right lingual gyrus	30.77 (4.81)	30.74 (4.77)	30.85 (4.75)	30.82 (5.05)	15.63	15.52	15.39	16.37	0.21 (0.83)	-0.58 (0.57)	-0.40 (0.69)	-0.10 (2.16)	0.27 (1.88)	0.07 (1.86)	1.58 (1.43)	1.45 (1.19)	1.22 (1.39)	0.99	0.99	0.99	0.99 (0.97-1.00)	0.99 (0.98-1.00)	0.99 (0.98-1.00)
Right medial orbitofrontal cortex	18.47 (2.35)	18.47 (2.30)	18.54 (2.61)	18.39 (2.30)	12.73	12.45	14.07	12.52	0.05 (0.96)	-0.38 (0.70)	0.47 (0.64)	0.00 (4.27)	0.19 (4.02)	-0.43 (4.59)	3.54 (2.27)	3.01 (2.59)	3.56 (2.82)	0.94	0.95	0.93	0.94 (0.87-0.98)	0.95 (0.89-0.98)	0.94 (0.85-0.97)
Right middle temporal gyrus	29.67 (4.83)	29.81 (4.88)	29.82 (4.92)	29.78 (4.82)	16.28	16.37	16.49	16.20	-1.84 (0.08)	-1.45 (0.16)	-1.09 (0.29)	0.46 (1.27)	0.46 (1.71)	0.35 (1.47)	1.12 (0.72)	1.40 (1.05)	1.11 (1.00)	1.00	1.00	1.00	1.00 (0.99-1.00)	0.99 (0.99-1.00)	1.00 (0.99-1.00)
Right parahippocampal gyrus	5.70 (0.79)	5.76 (0.78)	5.76 (0.71)	5.78 (0.82)	13.88	13.46	12.39	14.27	-0.77 (0.45)	-1.19 (0.25)	-1.56 (0.13)	1.16 (6.62)	1.24 (4.24)	1.31 (4.72)	4.93 (4.45)	3.40 (2.73)	3.74 (3.07)	0.88	0.96	0.96	0.88 (0.74-0.95)	0.95 (0.89-0.98)	0.95 (0.89-0.98)
Right paracentral lobule	14.40 (2.42)	14.44 (2.38)	14.38 (2.37)	14.51 (2.40)	16.80	16.46	16.45	16.57	-0.58 (0.57)	0.35 (0.73)	-2.16 (0.04)	0.31 (2.24)	-0.12 (2.21)	0.77 (1.65)	1.76 (1.37)	1.82 (1.20)	1.52 (0.95)	0.99	0.99	1.00	0.99 (0.98-1.00)	0.99 (0.98-1.00)	0.99 (0.99-1.00)
Right pars opercularis	11.97 (2.10)	12.07 (2.44)	11.94 (2.08)	12.00 (2.25)	17.51	20.20	17.42	18.79	-0.95 (0.36)	0.46 (0.65)	-0.34 (0.74)	0.44 (3.81)	-0.28 (2.89)	0.02 (3.04)	2.81 (2.54)	2.22 (1.81)	2.47 (1.69)	0.99	0.99	0.99	0.97 (0.94-0.99)	0.99 (0.97-1.00)	0.99 (0.97-0.99)
Right pars orbitalis	7.66 (1.28)	7.59 (1.27)	7.65 (1.28)	7.62 (1.23)	16.67	16.72	16.67	16.17	1.47 (0.16)	0.07 (0.94)	0.73 (0.47)	-0.82 (2.63)	-0.05 (2.99)	-0.37 (2.88)	2.08 (1.75)	2.49 (1.56)	2.48 (1.41)	0.99	0.98	0.98	0.99 (0.97-0.99)	0.98 (0.96-0.99)	0.98 (0.96-0.99)
Right pars triangularis	13.45 (2.39)	13.52 (2.54)	13.47 (2.44)	13.45 (2.48)	17.77	18.78	18.11	18.40	-1.15 (0.26)	-0.39 (0.70)	-0.11 (0.91)	0.34 (2.25)	0.12 (2.29)	-0.05 (2.20)	1.88 (1.22)	1.75 (1.42)	1.81 (1.19)	0.99	0.99	0.99	0.99 (0.98-1.00)	0.99 (0.98-1.00)	0.99 (0.98-1.00)
Right pericalcarine cortex	14.88 (2.77)	14.92 (2.78)	14.88 (2.80)	14.90 (2.78)	18.64	18.65	18.85	18.65	-0.38 (0.71)	0.04 (0.97)	-0.23 (0.82)	0.23 (3.17)	-0.05 (3.25)	0.14 (2.52)	2.36 (2.07)	2.42 (2.10)	1.97 (1.53)	0.99	0.98	0.99	0.99 (0.97-0.99)	0.98 (0.96-0.99)	0.99 (0.98-1.00)
Right postcentral gyrus	36.71 (5.02)	36.84 (5.09)	36.73 (5.02)	36.83 (4.98)	13.67	13.82	13.66	13.53	-1.24 (0.23)	-0.15 (0.89)	-1.56 (0.13)	0.33 (1.31)	0.04 (1.37)	0.33 (0.95)	1.08 (0.78)	1.06 (0.83)	0.73 (0.69)	1.00	0.99	1.00	1.00 (0.99-1.00)	1.00 (0.99-1.00)	1.00 (0.99-1.00)
Right posterior cingulate cortex	10.11 (2.71)	10.14 (2.69)	10.10 (2.74)	10.15 (2.81)	26.79	26.57	27.11	27.66	-0.21 (0.84)	0.05 (0.96)	-0.18 (0.86)	0.34 (7.73)	-0.33 (6.54)	0.12 (10.68)	5.20 (5.61)	4.17 (4.96)	6.01 (8.72)	0.97	0.98	0.94	0.97 (0.93-0.99)	0.98 (0.96-0.99)	0.94 (0.85-0.97)
Right precentral gyrus	45.39 (7.27)	45.38 (7.37)	45.26 (7.20)	45.27 (7.49)	16.01	16.23	15.91	16.54	0.14 (0.89)	1.25 (0.22)	1.29 (0.21)	-0.06 (1.08)	-0.28 (1.08)	-0.35 (1.02)	0.84 (0.65)	0.93 (0.58)	0.81 (0.69)	1.00	1.00	1.00	1.00 (0.99-1.00)	1.00 (0.99-1.00)	1.00 (1.00-1.00)
Right precuneus	35.57 (5.40)	35.44 (5.29)	35.46 (5.40)	35.54 (5.48)	15.17	14.94	15.22	15.41	1.19 (0.25)	1.20 (0.24)	0.27 (0.79)	-0.33 (1.29)	-0.31 (1.22)	-0.12 (1.45)	0.94 (0.93)	1.03 (0.70)	1.20 (0.78)	1.00	1.00	1.00	1.00 (0.99-1.00)	1.00 (0.99-1.00)	1.00 (0.99-1.00)
Right rostral anterior cingulate cortex	5.66 (1.36)	5.68 (1.56)	5.61 (1.45)	5.66 (1.42)	24.09	27.55	25.89	25.02	-0.22 (0.83)	0.57 (0.58)	-0.04 (0.97)	-0.34 (6.30)	-1.19 (7.18)	-0.14 (5.06)	4.93 (3.80)	5.42 (4.71)	3.52 (3.56)	0.97	0.96	0.98	0.96 (0.91-0.98)	0.96 (0.91-0.98)	0.98 (0.95-0.99)
Right rostral middle frontal gyrus	50.93 (7.18)	51.25 (7.36)	50.90 (7.26)	51.37 (7.26)	14.10	14.36	14.26	14.13	-1.51 (0.15)	0.18 (0.86)	-2.18 (0.04)	0.57 (1.82)	-0.08 (1.87)	0.86 (1.84)	1.54 (1.07)	1.45 (1.13)	1.69 (1.07)	0.99	0.99	0.99	0.99 (0.98-1.00)	0.99 (0.98-1.00)	0.99 (0.97-1.00)

Right superior frontal gyrus	64.26 (11.91)	64.36 (11.82)	64.56 (11.84)	64.65 (11.80)	18.54	18.36	18.35	18.25	-0.84 (0.41)	-2.37 (0.03)	-2.78 (0.01)	0.18 (0.91)	0.49 (0.99)	0.64 (1.10)	0.69 (0.61)	0.88 (0.66)	1.00 (0.78)	1.00	1.00	1.00	1.00 (1.00-1.00)	1.00 (1.00-1.00)	1.00 (0.99-1.00)
Right superior parietal cortex	47.96 (7.89)	47.87 (7.90)	48.06 (7.77)	48.29 (7.75)	16.44	16.51	16.16	16.04	1.10 (0.29)	-0.81 (0.43)	-2.81 (0.01)	-0.20 (0.83)	0.26 (1.29)	0.74 (1.24)	0.71 (0.45)	1.08 (0.71)	1.14 (0.87)	1.00	1.00	1.00	1.00 (1.00-1.00)	1.00 (0.99-1.00)	1.00 (0.99-1.00)
Right superior temporal gyrus	33.81 (4.25)	33.76 (4.33)	33.93 (4.39)	33.68 (4.21)	12.58	12.82	12.94	12.51	0.35 (0.73)	-0.67 (0.51)	1.20 (0.24)	-0.19 (2.01)	0.31 (2.18)	-0.38 (1.52)	1.49 (1.33)	1.45 (1.64)	1.26 (0.89)	0.99	0.98	0.99	0.99 (0.97-0.99)	0.98 (0.96-0.99)	0.99 (0.98-1.00)
Right supramarginal gyrus	32.22 (4.87)	32.31 (4.98)	32.29 (4.91)	32.29 (4.86)	15.13	15.40	15.22	15.04	-0.92 (0.37)	-0.80 (0.43)	-0.86 (0.40)	0.22 (1.30)	0.20 (1.30)	0.23 (1.14)	1.06 (0.75)	1.02 (0.81)	0.88 (0.73)	1.00	1.00	1.00	1.00 (0.99-1.00)	1.00 (0.99-1.00)	1.00 (0.99-1.00)
Right frontal pole	2.89 (0.31)	2.88 (0.31)	2.96 (0.31)	2.87 (0.30)	10.70	10.83	10.52	10.59	0.26 (0.80)	-1.84 (0.08)	0.81 (0.43)	-0.33 (5.59)	2.38 (6.02)	-0.90 (5.52)	4.60 (3.02)	5.60 (3.06)	4.31 (3.44)	0.87	0.84	0.88	0.88 (0.73-0.95)	0.82 (0.62-0.92)	0.88 (0.73-0.95)
Right temporal pole	4.38 (0.82)	4.28 (0.79)	4.36 (0.80)	4.32 (0.81)	18.82	18.47	18.33	18.65	1.85 (0.08)	0.25 (0.80)	0.90 (0.38)	-2.17 (6.35)	-0.31 (7.11)	-1.29 (7.01)	4.53 (4.87)	5.65 (4.15)	5.47 (4.42)	0.96	0.93	0.94	0.95 (0.88-0.98)	0.93 (0.84-0.97)	0.94 (0.87-0.97)
Right transverse temporal gyrus	3.00 (0.42)	2.98 (0.37)	2.99 (0.41)	3.01 (0.42)	13.91	12.35	13.85	13.86	0.47 (0.64)	0.30 (0.77)	-0.54 (0.59)	-0.28 (4.75)	-0.29 (4.30)	0.46 (3.92)	3.80 (2.75)	3.45 (2.48)	3.12 (2.31)	0.94	0.95	0.96	0.94 (0.86-0.97)	0.95 (0.89-0.98)	0.96 (0.91-0.98)
Right insula	24.16 (2.88)	23.91 (3.62)	24.08 (2.99)	23.53 (3.67)	11.93	15.13	12.40	15.59	0.70 (0.49)	0.23 (0.82)	1.73 (0.10)	-1.47 (6.93)	-0.38 (6.80)	-3.13 (7.67)	5.67 (4.08)	5.55 (3.76)	6.23 (5.33)	0.89	0.84	0.89	0.87 (0.72-0.94)	0.84 (0.66-0.93)	0.86 (0.68-0.94)
Local cortical thickness (mm)																							
Left banks superior temporal sulcus	2.32 (0.16)	2.32 (0.17)	2.33 (0.15)	2.32 (0.16)	6.86	7.44	6.61	7.08	0.59 (0.56)	-0.48 (0.64)	0.88 (0.39)	-0.30 (2.05)	0.17 (1.50)	-0.28 (1.43)	1.58 (1.30)	1.00 (1.11)	1.11 (0.91)	0.96	0.97	0.98	0.96 (0.91-0.98)	0.97 (0.94-0.99)	0.98 (0.95-0.99)
Left caudal anterior cingulate cortex	2.62 (0.20)	2.64 (0.24)	2.63 (0.21)	2.60 (0.19)	7.77	9.07	8.15	7.44	-1.11 (0.28)	-0.68 (0.51)	0.63 (0.53)	0.92 (4.21)	0.59 (4.29)	-0.61 (4.72)	3.54 (2.34)	3.39 (2.60)	3.42 (3.23)	0.88	0.85	0.81	0.87 (0.71-0.94)	0.85 (0.68-0.94)	0.82 (0.61-0.92)
Left caudal middle frontal gyrus	2.37 (0.15)	2.37 (0.14)	2.37 (0.14)	2.37 (0.14)	6.37	5.83	5.84	6.01	0.27 (0.79)	0.62 (0.54)	0.40 (0.69)	-0.09 (2.07)	-0.24 (2.05)	-0.15 (1.99)	1.65 (1.19)	1.60 (1.26)	1.54 (1.23)	0.94	0.95	0.95	0.94 (0.87-0.98)	0.94 (0.87-0.98)	0.95 (0.88-0.98)
Left cuneus	1.83 (0.11)	1.82 (0.11)	1.83 (0.11)	1.82 (0.11)	6.03	5.97	6.02	6.04	0.63 (0.53)	-0.20 (0.84)	0.72 (0.48)	-0.25 (1.84)	0.07 (1.58)	-0.21 (1.38)	1.46 (1.10)	1.18 (1.02)	1.09 (0.84)	0.95	0.96	0.98	0.95 (0.89-0.98)	0.97 (0.92-0.99)	0.98 (0.94-0.99)
Left entorhinal cortex	2.91 (0.52)	2.90 (0.47)	2.91 (0.45)	2.93 (0.47)	17.79	16.18	15.63	15.90	0.15 (0.88)	-0.02 (0.98)	-0.74 (0.46)	0.09 (6.57)	0.39 (5.36)	1.13 (4.90)	4.57 (4.61)	3.98 (3.51)	3.84 (3.14)	0.93	0.96	0.96	0.93 (0.84-0.97)	0.95 (0.89-0.98)	0.96 (0.90-0.98)
Left fusiform gyrus	2.54 (0.21)	2.53 (0.21)	2.54 (0.21)	2.54 (0.19)	8.17	8.22	8.34	7.66	1.83 (0.08)	0.24 (0.81)	0.74 (0.47)	-0.58 (1.48)	-0.11 (1.80)	-0.19 (1.50)	1.24 (0.96)	1.15 (1.37)	1.27 (0.78)	0.98	0.98	0.99	0.98 (0.96-0.99)	0.98 (0.95-0.99)	0.98 (0.96-0.99)
Left inferior parietal cortex	2.20 (0.19)	2.20 (0.19)	2.20 (0.19)	2.20 (0.19)	8.54	8.56	8.53	8.73	-0.64 (0.53)	0.19 (0.85)	0.30 (0.77)	0.14 (1.03)	-0.06 (1.33)	-0.11 (1.50)	0.79 (0.65)	1.13 (0.66)	1.16 (0.94)	0.99	0.99	0.99	0.99 (0.98-1.00)	0.99 (0.97-1.00)	0.99 (0.97-0.99)
Left inferior temporal gyrus	2.61 (0.17)	2.60 (0.18)	2.61 (0.17)	2.60 (0.17)	6.38	6.92	6.58	6.43	1.25 (0.22)	0.45 (0.66)	1.21 (0.24)	-0.53 (1.87)	-0.20 (2.01)	-0.50 (1.94)	1.21 (1.49)	1.62 (1.15)	1.52 (1.27)	0.96	0.95	0.95	0.96 (0.91-0.98)	0.95 (0.90-0.98)	0.95 (0.89-0.98)
Left isthmus cingulate cortex	2.14 (0.23)	2.11 (0.27)	2.11 (0.25)	2.11 (0.23)	10.75	12.86	12.07	10.89	2.13 (0.04)	2.59 (0.02)	2.26 (0.03)	-1.80 (3.44)	-1.68 (2.92)	-1.39 (2.83)	3.13 (2.23)	2.63 (2.07)	2.56 (1.78)	0.97	0.98	0.97	0.95 (0.87-0.98)	0.96 (0.89-0.99)	0.96 (0.89-0.98)
Left lateral occipital cortex	2.08 (0.15)	2.08 (0.14)	2.08 (0.13)	2.08 (0.14)	7.14	6.60	6.41	6.51	0.92 (0.37)	0.36 (0.72)	1.11 (0.28)	-0.29 (1.56)	-0.11 (1.88)	-0.36 (1.65)	1.24 (0.95)	1.49 (1.11)	1.29 (1.06)	0.98	0.96	0.97	0.97 (0.94-0.99)	0.96 (0.91-0.98)	0.97 (0.93-0.99)
Left lateral orbitofrontal cortex	2.54 (0.16)	2.52 (0.16)	2.54 (0.16)	2.53 (0.15)	6.12	6.18	6.37	5.94	2.02 (0.06)	-0.07 (0.95)	0.15 (0.88)	-0.76 (1.74)	0.01 (1.69)	-0.06 (2.13)	1.46 (1.18)	1.26 (1.09)	1.61 (1.35)	0.96	0.96	0.94	0.95 (0.89-0.98)	0.96 (0.91-0.98)	0.94 (0.86-0.97)
Left lingual gyrus	1.94 (0.09)	1.94 (0.09)	1.94 (0.09)	1.94 (0.10)	4.82	4.79	4.44	5.13	-0.51 (0.62)	-0.58 (0.57)	-0.23 (0.82)	0.14 (1.30)	0.17 (1.25)	0.06 (1.64)	0.97 (0.85)	1.00 (0.75)	1.19 (1.10)	0.96	0.97	0.95	0.96 (0.91-0.98)	0.97 (0.92-0.99)	0.95 (0.89-0.98)
Left medial orbitofrontal cortex	2.24 (0.17)	2.24 (0.17)	2.24 (0.18)	2.26 (0.17)	7.52	7.54	7.87	7.56	0.17 (0.87)	0.19 (0.85)	-1.42 (0.17)	-0.08 (2.32)	-0.11 (2.07)	0.72 (2.37)	1.78 (1.44)	1.66 (1.20)	1.92 (1.51)	0.95	0.96	0.95	0.95 (0.89-0.98)	0.96 (0.92-0.99)	0.95 (0.88-0.98)
Left middle temporal gyrus	2.58 (0.18)	2.57 (0.19)	2.58 (0.18)	2.57 (0.18)	6.97	7.38	6.97	6.84	1.05 (0.30)	0.78 (0.44)	1.31 (0.21)	-0.41 (1.71)	-0.22 (1.30)	-0.42 (1.54)	1.03 (1.41)	1.12 (0.66)	1.19 (1.03)	0.97	0.98	0.98	0.97 (0.94-0.99)	0.98 (0.96-0.99)	0.97 (0.94-0.99)
Left parahippocampal gyrus	2.54 (0.27)	2.52 (0.27)	2.53 (0.26)	2.52 (0.28)	10.81	10.64	10.25	10.99	2.38 (0.03)	0.62 (0.54)	1.79 (0.09)	-0.91 (1.82)	-0.24 (2.12)	-0.89 (2.30)	1.63 (1.18)	1.74 (1.18)	2.10 (1.23)	0.99	0.98	0.98	0.98 (0.95-0.99)	0.98 (0.95-0.99)	0.98 (0.94-0.99)
Left paracentral lobule	2.30 (0.13)	2.29 (0.12)	2.28 (0.13)	2.30 (0.12)	5.51	5.04	5.87	5.20	1.25 (0.23)	2.16 (0.04)	-0.02 (0.98)	-0.52 (2.00)	-0.95 (2.06)	0.02 (2.00)	1.50 (1.39)	1.59 (1.60)	1.60 (1.16)	0.93	0.94	0.93	0.92 (0.83-0.97)	0.93 (0.82-0.97)	0.93 (0.84-0.97)
Left pars opercularis	2.48 (0.08)	2.48 (0.09)	2.48 (0.09)	2.47 (0.09)	3.31	3.68	3.53	3.63	0.71 (0.49)	0.28 (0.78)	1.85 (0.08)	-0.21 (1.36)	-0.08 (1.25)	-0.53 (1.32)	0.95 (0.98)	1.04 (0.67)	1.07 (0.91)	0.93	0.94	0.93	0.93 (0.84-0.97)	0.94 (0.85-0.97)	0.92 (0.81-0.97)
Left pars orbitalis	2.61 (0.22)	2.59 (0.23)	2.60 (0.22)	2.59 (0.22)	8.31	9.01	8.54	8.67	1.45 (0.16)	0.22 (0.83)	1.13 (0.27)	-0.86 (2.69)	-0.13 (2.29)	-0.66 (2.60)	2.14 (1.79)	1.79 (1.39)	2.08 (1.64)	0.96	0.96	0.95	0.95 (0.89-0.98)	0.96 (0.91-0.98)	0.95 (0.89-0.98)
Left pars triangularis	2.33 (0.11)	2.32 (0.13)	2.32 (0.11)	2.32 (0.13)	4.91	5.41	4.88	5.51	1.52 (0.14)	1.37 (0.18)	1.84 (0.08)	-0.60 (1.76)	-0.45 (1.52)	-0.63 (1.53)	1.37 (1.23)	1.08 (1.14)	1.29 (1.00)	0.95	0.95	0.96	0.94 (0.86-0.97)	0.95 (0.88-0.98)	0.95 (0.89-0.98)
Left pericalcarine cortex	1.60 (0.09)	1.60 (0.09)	1.60 (0.10)	1.60 (0.10)	5.88	5.86	6.03	6.27	0.39 (0.70)	0.33 (0.75)	-0.65 (0.52)	-0.22 (2.67)	-0.16 (2.16)	0.29 (2.27)	2.04 (1.67)	1.59 (1.42)	1.78 (1.38)	0.90	0.93	0.93	0.90 (0.77-0.96)	0.93 (0.85-0.97)	0.93 (0.85-0.97)
Left postcentral gyrus	2.00 (0.12)	2.00 (0.12)	1.99 (0.12)	2.00 (0.11)	5.96	5.78	5.98	5.67	-0.65 (0.52)	0.84 (0.41)	0.31 (0.76)	0.15 (0.96)	-0.21 (1.17)	-0.04 (0.89)	0.79 (0.55)	0.99 (0.63)	0.72 (0.51)	0.99	0.98	0.99	0.99 (0.97-0.99)	0.98 (0.96-0.99)	0.99 (0.97-1.00)
Left posterior cingulate cortex	2.28 (0.17)	2.26 (0.17)	2.27 (0.18)	2.24 (0.16)	7.30	7.58	7.98	7.31	1.40 (0.18)	0.85 (0.40)	2.72 (0.01)	-1.02 (3.47)	-0.67 (3.42)	-1.82 (3.12)	2.89 (2.10)	2.63 (2.23)	2.94 (2.04)	0.90	0.90	0.91	0.90 (0.77-0.96)	0.90 (0.78-0.96)	0.88 (0.68-0.95)
Left precentral gyrus	2.46 (0.14)	2.46 (0.13)	2.46 (0.14)	2.46 (0.13)	5.55	5.39	5.50	5.27	-0.47 (0.64)	-0.68 (0.51)	-0.59 (0.56)	0.10 (0.87)	0.15 (1.03)	0.15 (1.12)	0.67 (0.55)	0.80 (0.64)	0.91 (0.64)	0.99	0.98	0.98	0.99 (0.97-0.99)	0.98 (0.96-0.99)	0.98 (0.95-0.99)
Left precuneus	2.19 (0.19)	2.19 (0.19)	2.18 (0.19)	2.19 (0.18)	8.46	8.76	8.78	8.26	-0.75 (0.46)	0.90 (0.38)	0.68 (0.50)	0.15 (1.15)	-0.25 (1.21)	-0.12 (0.93)	0.87 (0.74)	0.96 (0.76)	0.78 (0.49)	0.99	0.99	0.99	0.99 (0.98-1.00)	0.99 (0.98-1.00)	0.99 (0.99-1.00)
Left rostral anterior cingulate cortex	2.59 (0.28)	2.61 (0.29)	2.61 (0.26)	2.61 (0.29)	10.75	11.14	9.84	11.03	-0.76 (0.45)	-1.04 (0.31)	-0.81 (0.43)	0.91 (5.78)	0.98 (3.99)	0.81 (4.93)	3.53 (4.60)	2.64 (3.10)	3.17 (3.80)	0.86	0.92	0.90	0.86 (0.69-0.94)	0.92 (0.82-0.97)	0.90 (0.78-0.96)
Left rostral middle frontal gyrus	2.24 (0.12)	2.24 (0.13)	2.24 (0.12)	2.23 (0.11)	5.40	5.76	5.56	5.03	-0.02 (0.99)	-0.78 (0.44)	1.17 (0.26)	-0.01 (1.68)	0.24 (1.51)	-0.46 (1.87)	1.22 (1.13)	1.20 (0.92)	1.30 (1.40)	0.95	0.96	0.94	0.95 (0.89-0.98)	0.96 (0.91-0.98)	0.93 (0.85-0.97)
Left superior frontal gyrus	2.49 (0.14)	2.48 (0.14)	2.49 (0.14)	2.49 (0.14)	5.62	5.67	5.63	5.72	1.50 (0.15)	0.70 (0.49)	0.60 (0.55)	-0.37 (1.16)	-0.23 (1.52)	-0.17 (1.24)	0.97 (0.70)	1.24 (0.87)	1.06 (0.63)	0.98	0.96	0.98	0.98 (0.95-0.99)	0.96 (0.92-0.98)	0.98 (0.95-0.99)
Left superior parietal cortex	2.06 (0.18)	2.05 (0.17)	2.05 (0.17)	2.05 (0.17)	8.50	8.22	8.19	8.37	1.44 (0.16)	0.88 (0.39)	1.07 (0.30)	-0.37 (1.24)	-0.22 (1.27)	-0.32 (1.49)	1.00 (0.80)	0.99 (0.80)	1.07 (1.07)	0.99	0.99	0.99	0.99 (0.97-1.00)	0.99 (0.97-0.99)	0.99 (0.96-0.99)
Left superior temporal gyrus	2.47 (0.15)	2.48 (0.15)	2.47 (0.15)	2.47 (0.15)	6.11	6.20	6.21	6.00	-0.88 (0.39)	-0.17 (0.87)	-0.61 (0.55)	0.16 (0.92)	0.03 (0.93)	0.14 (1.05)	0.75 (0.54)	0.79 (0.46)	0.80 (0.68)	0.99	0.99	0.99	0.99 (0.98-1.00)	0.99 (0.97-1.00)	0.99 (0.97-0.99)
Left supramarginal gyrus	2.31 (0.13)	2.33 (0.13)	2.31 (0.13)	2.31 (0.13)	5.66	5.42	5.80	5.63	-2.33 (0.03)	-0.12 (0.91)	-0.09 (0.93)	0.56 (1.13)	0.02 (1.07)	0.02 (1.09)	0.98 (0.78)	0.86 (0.61)	0.70 (0.82)	0.98	0.98	0.98	0.98 (0.93-0.99)	0.98 (0.96-0.99)	0.98 (0.96-0.99)
Left frontal pole	2.56 (0.26)	2.55 (0.26)	2.55 (0.25)	2.54 (0.26)	10.08	10.24	9.85	10.31	0.72 (0.48)	0.78 (0.44)	1.51 (0.15)	-0.47 (3.00)	-0.34 (2.14)	-0.89 (2.77)	2.24 (1.99)	1.76 (1.21)	2.05 (2.03)	0.96	0.98	0.96	0.96 (0.90-0.98)	0.98 (0.94-0.99)	0.96 (0.91-0.98)

Left temporal pole	3.32 (0.37)	3.28 (0.36)	3.29 (0.35)	3.32 (0.40)	11.12	11.06	10.65	12.21	1.24 (0.23)	1.14 (0.27)	0.21 (0.83)	-1.33 (5.17)	-0.90 (4.06)	-0.31 (4.15)	3.77 (3.70)	2.98 (2.83)	2.75 (3.06)	0.89	0.94	0.94	0.89 (0.76-0.95)	0.93 (0.85-0.97)	0.94 (0.86-0.98)
Left transverse temporal gyrus	2.27 (0.21)	2.25 (0.23)	2.27 (0.23)	2.26 (0.23)	9.25	10.31	10.17	10.07	1.58 (0.13)	0.10 (0.92)	0.68 (0.51)	-0.85 (2.40)	-0.13 (2.37)	-0.30 (1.57)	1.87 (1.70)	1.71 (1.61)	1.17 (1.06)	0.98	0.98	0.99	0.97 (0.93-0.99)	0.97 (0.93-0.99)	0.99 (0.97-0.99)
Left insula	2.72 (0.21)	2.70 (0.19)	2.75 (0.22)	2.72 (0.21)	7.64	6.88	7.98	7.88	0.82 (0.42)	-1.41 (0.17)	-0.10 (0.92)	-0.56 (3.25)	1.17 (3.99)	0.06 (3.98)	2.46 (2.14)	3.05 (2.76)	3.27 (2.16)	0.89	0.87	0.87	0.89 (0.75-0.95)	0.86 (0.70-0.94)	0.87 (0.72-0.95)
Right banks superior temporal sulcus	2.37 (0.14)	2.36 (0.16)	2.37 (0.15)	2.37 (0.15)	6.07	6.67	6.42	6.45	0.75 (0.46)	0.06 (0.95)	-0.18 (0.86)	-0.26 (1.42)	-0.04 (1.52)	0.04 (1.66)	1.14 (0.85)	1.18 (0.93)	1.38 (0.89)	0.98	0.97	0.97	0.98 (0.94-0.99)	0.97 (0.93-0.99)	0.97 (0.92-0.99)
Right caudal anterior cingulate cortex	2.46 (0.18)	2.43 (0.18)	2.41 (0.16)	2.42 (0.18)	7.51	7.39	6.72	7.59	1.13 (0.27)	3.40 (0.00)	2.62 (0.02)	-0.91 (4.10)	-1.84 (2.58)	-1.29 (2.47)	3.02 (2.85)	2.46 (1.96)	2.18 (1.69)	0.87	0.94	0.95	0.87 (0.71-0.94)	0.90 (0.66-0.97)	0.94 (0.83-0.98)
Right caudal middle frontal gyrus	2.38 (0.17)	2.37 (0.16)	2.37 (0.17)	2.39 (0.17)	7.08	6.90	7.00	6.94	0.47 (0.64)	0.62 (0.54)	-1.24 (0.23)	-0.15 (1.68)	-0.18 (1.41)	0.47 (1.78)	1.36 (0.94)	1.13 (0.82)	1.42 (1.14)	0.97	0.98	0.97	0.97 (0.94-0.99)	0.98 (0.95-0.99)	0.97 (0.93-0.99)
Right cuneus	1.85 (0.13)	1.85 (0.13)	1.85 (0.13)	1.85 (0.13)	6.97	6.98	6.95	6.98	0.18 (0.86)	-0.11 (0.92)	0.19 (0.85)	-0.06 (1.62)	0.04 (1.52)	-0.06 (1.36)	1.26 (0.98)	1.30 (0.75)	1.08 (0.79)	0.97	0.98	0.98	0.97 (0.93-0.99)	0.98 (0.95-0.99)	0.98 (0.96-0.99)
Right entorhinal cortex	3.05 (0.45)	3.01 (0.48)	3.03 (0.47)	3.08 (0.43)	14.89	15.84	15.34	14.11	1.03 (0.32)	0.38 (0.71)	-0.73 (0.47)	-1.24 (5.03)	-0.58 (6.12)	1.03 (6.04)	3.76 (3.49)	4.00 (4.58)	5.08 (3.24)	0.95	0.92	0.91	0.95 (0.87-0.98)	0.92 (0.82-0.97)	0.91 (0.80-0.96)
Right fusiform gyrus	2.54 (0.20)	2.53 (0.21)	2.54 (0.20)	2.54 (0.21)	8.00	8.15	8.07	8.10	0.26 (0.80)	-0.56 (0.58)	-0.80 (0.43)	-0.08 (1.16)	0.18 (1.54)	0.29 (1.72)	0.93 (0.67)	1.27 (0.84)	1.36 (1.05)	0.99	0.98	0.98	0.99 (0.98-1.00)	0.98 (0.96-0.99)	0.98 (0.95-0.99)
Right inferior parietal cortex	2.21 (0.14)	2.21 (0.15)	2.21 (0.15)	2.21 (0.16)	6.32	6.72	6.87	7.17	-0.61 (0.55)	0.12 (0.91)	0.14 (0.89)	0.11 (1.11)	-0.07 (1.35)	-0.11 (1.73)	0.89 (0.64)	1.00 (0.88)	1.18 (1.24)	0.99	0.98	0.98	0.99 (0.97-0.99)	0.98 (0.95-0.99)	0.97 (0.93-0.99)
Right inferior temporal gyrus	2.62 (0.12)	2.62 (0.11)	2.62 (0.13)	2.64 (0.12)	4.60	4.33	4.78	4.48	-0.61 (0.55)	0.20 (0.85)	-1.97 (0.06)	0.25 (1.83)	-0.06 (1.19)	0.74 (1.78)	1.24 (1.35)	0.95 (0.70)	1.43 (1.27)	0.92	0.97	0.93	0.92 (0.82-0.97)	0.97 (0.93-0.99)	0.92 (0.80-0.97)
Right isthmus cingulate cortex	2.09 (0.21)	2.06 (0.20)	2.07 (0.25)	2.07 (0.26)	10.03	9.71	11.91	12.78	1.50 (0.15)	0.86 (0.40)	0.72 (0.48)	-1.14 (3.74)	-1.01 (4.88)	-1.11 (6.16)	2.61 (2.86)	3.02 (3.92)	2.86 (5.54)	0.93	0.94	0.95	0.93 (0.83-0.97)	0.93 (0.84-0.97)	0.92 (0.83-0.97)
Right lateral occipital cortex	2.08 (0.13)	2.09 (0.14)	2.08 (0.14)	2.09 (0.14)	6.32	6.53	6.50	6.80	-1.41 (0.17)	-0.29 (0.78)	-1.08 (0.29)	0.32 (1.14)	0.09 (1.58)	0.25 (1.23)	0.77 (0.89)	1.22 (0.96)	1.09 (0.59)	0.99	0.97	0.99	0.98 (0.96-0.99)	0.97 (0.93-0.99)	0.98 (0.96-0.99)
Right lateral orbitofrontal cortex	2.51 (0.12)	2.50 (0.12)	2.50 (0.13)	2.50 (0.14)	4.74	4.87	5.36	5.76	0.72 (0.48)	0.54 (0.60)	0.29 (0.77)	-0.27 (1.68)	-0.28 (2.20)	-0.28 (3.65)	1.34 (1.01)	1.73 (1.35)	2.34 (2.77)	0.94	0.91	0.76	0.94 (0.86-0.97)	0.91 (0.79-0.96)	0.75 (0.49-0.89)
Right lingual gyrus	2.00 (0.12)	1.99 (0.13)	2.00 (0.13)	1.99 (0.12)	6.25	6.37	6.49	6.26	0.82 (0.42)	0.03 (0.98)	0.97 (0.34)	-0.34 (2.00)	-0.02 (1.36)	-0.38 (1.97)	1.36 (1.48)	1.03 (0.85)	1.18 (1.60)	0.95	0.98	0.96	0.95 (0.89-0.98)	0.98 (0.95-0.99)	0.96 (0.90-0.98)
Right medial orbitofrontal cortex	2.33 (0.14)	2.31 (0.15)	2.32 (0.15)	2.34 (0.17)	5.97	6.69	6.63	7.19	1.32 (0.20)	0.76 (0.45)	-0.66 (0.51)	-0.66 (2.24)	-0.40 (2.22)	0.34 (2.92)	1.97 (1.20)	1.77 (1.34)	1.92 (2.19)	0.94	0.94	0.92	0.94 (0.86-0.97)	0.94 (0.86-0.97)	0.90 (0.79-0.96)
Right middle temporal gyrus	2.67 (0.16)	2.66 (0.16)	2.67 (0.16)	2.67 (0.17)	5.87	6.14	6.12	6.32	2.00 (0.06)	0.47 (0.64)	0.47 (0.64)	-0.41 (0.93)	-0.13 (1.14)	-0.16 (1.36)	0.81 (0.59)	0.88 (0.71)	1.08 (0.81)	0.99	0.98	0.98	0.99 (0.97-0.99)	0.98 (0.96-0.99)	0.98 (0.95-0.99)
Right parahippocampal gyrus	2.53 (0.31)	2.52 (0.30)	2.51 (0.32)	2.53 (0.30)	12.43	12.03	12.66	11.75	1.46 (0.16)	1.37 (0.18)	0.17 (0.86)	-0.52 (1.95)	-0.83 (2.85)	0.00 (2.31)	1.63 (1.14)	2.36 (1.74)	1.86 (1.31)	0.99	0.98	0.98	0.99 (0.97-1.00)	0.98 (0.94-0.99)	0.98 (0.96-0.99)
Right paracentral lobule	2.31 (0.12)	2.30 (0.13)	2.29 (0.13)	2.29 (0.14)	5.40	5.78	5.53	6.06	0.42 (0.68)	2.53 (0.02)	2.01 (0.06)	-0.18 (1.75)	-0.66 (1.26)	-0.82 (1.89)	1.44 (0.96)	1.10 (0.88)	1.52 (1.36)	0.96	0.98	0.95	0.95 (0.89-0.98)	0.97 (0.91-0.99)	0.94 (0.86-0.98)
Right pars opercularis	2.47 (0.12)	2.47 (0.13)	2.48 (0.13)	2.48 (0.11)	4.74	5.19	5.30	4.27	0.13 (0.90)	-0.35 (0.73)	-0.64 (0.53)	-0.07 (1.64)	0.10 (1.66)	0.26 (1.81)	1.21 (1.07)	1.26 (1.04)	1.30 (1.25)	0.95	0.95	0.93	0.95 (0.88-0.98)	0.95 (0.88-0.98)	0.93 (0.83-0.97)
Right pars orbitalis	2.56 (0.17)	2.56 (0.18)	2.55 (0.18)	2.56 (0.18)	6.54	7.14	7.09	7.13	-0.46 (0.65)	0.42 (0.68)	-0.27 (0.79)	0.17 (2.09)	-0.25 (2.35)	0.09 (2.25)	1.46 (1.48)	1.91 (1.33)	1.64 (1.50)	0.95	0.94	0.95	0.95 (0.89-0.98)	0.94 (0.86-0.97)	0.95 (0.88-0.98)
Right pars triangularis	2.35 (0.15)	2.34 (0.15)	2.36 (0.15)	2.36 (0.15)	6.22	6.25	6.55	6.56	0.73 (0.47)	-1.01 (0.32)	-0.83 (0.42)	-0.24 (1.48)	0.39 (1.83)	0.25 (1.57)	1.11 (0.98)	1.41 (1.19)	1.21 (1.00)	0.97	0.96	0.97	0.97 (0.93-0.99)	0.96 (0.90-0.98)	0.97 (0.93-0.99)
Right pericalcarine cortex	1.62 (0.12)	1.61 (0.12)	1.61 (0.13)	1.61 (0.11)	7.38	7.43	7.85	6.87	1.56 (0.13)	1.74 (0.10)	1.19 (0.25)	-0.63 (1.86)	-0.98 (2.51)	-0.70 (2.92)	1.41 (1.33)	1.97 (1.81)	1.88 (2.30)	0.97	0.94	0.92	0.97 (0.92-0.99)	0.94 (0.85-0.97)	0.91 (0.81-0.96)
Right postcentral gyrus	1.97 (0.09)	1.97 (0.10)	1.96 (0.10)	1.97 (0.10)	4.76	4.84	5.09	5.26	0.48 (0.63)	1.34 (0.19)	0.42 (0.68)	-0.11 (1.04)	-0.44 (1.51)	-0.14 (1.27)	0.80 (0.66)	1.25 (0.92)	1.02 (0.75)	0.98	0.96	0.97	0.98 (0.95-0.99)	0.95 (0.89-0.98)	0.97 (0.93-0.99)
Right posterior cingulate cortex	2.27 (0.21)	2.27 (0.24)	2.25 (0.21)	2.27 (0.23)	9.38	10.41	9.19	9.98	-0.15 (0.88)	0.63 (0.54)	-0.16 (0.87)	0.04 (4.68)	-0.57 (4.38)	0.09 (5.36)	2.80 (3.69)	3.28 (2.86)	3.57 (3.92)	0.89	0.89	0.84	0.89 (0.75-0.95)	0.89 (0.76-0.95)	0.85 (0.66-0.93)
Right precentral gyrus	2.49 (0.12)	2.48 (0.13)	2.48 (0.12)	2.49 (0.12)	4.86	5.17	4.99	4.64	0.91 (0.37)	0.80 (0.43)	-0.11 (0.91)	-0.27 (1.32)	-0.26 (1.47)	0.04 (1.15)	1.01 (0.87)	0.96 (1.13)	0.81 (0.81)	0.97	0.95	0.97	0.97 (0.92-0.99)	0.96 (0.90-0.98)	0.97 (0.94-0.99)
Right precuneus	2.18 (0.17)	2.18 (0.17)	2.18 (0.17)	2.18 (0.18)	7.64	7.85	7.87	8.09	0.41 (0.69)	0.06 (0.96)	-0.04 (0.97)	-0.12 (1.18)	-0.03 (1.22)	-0.03 (1.05)	0.82 (0.84)	1.02 (0.64)	0.83 (0.62)	0.99	0.99	0.99	0.99 (0.97-1.00)	0.99 (0.97-1.00)	0.99 (0.98-1.00)
Right rostral anterior cingulate cortex	2.89 (0.26)	2.89 (0.28)	2.89 (0.28)	2.89 (0.26)	9.13	9.84	9.63	8.89	-0.27 (0.79)	0.01 (0.99)	-0.14 (0.89)	0.13 (3.23)	-0.05 (3.22)	0.07 (1.91)	2.53 (1.93)	2.08 (2.42)	1.35 (1.31)	0.95	0.95	0.98	0.95 (0.88-0.98)	0.95 (0.88-0.98)	0.98 (0.95-0.99)
Right rostral middle frontal gyrus	2.26 (0.13)	2.26 (0.15)	2.26 (0.14)	2.25 (0.14)	5.93	6.47	6.22	6.26	1.01 (0.32)	0.09 (0.93)	1.74 (0.10)	-0.31 (1.30)	-0.05 (1.60)	-0.58 (1.46)	1.08 (0.76)	1.38 (0.76)	1.28 (0.87)	0.98	0.97	0.97	0.98 (0.95-0.99)	0.97 (0.92-0.99)	0.97 (0.92-0.99)
Right superior frontal gyrus	2.54 (0.13)	2.53 (0.14)	2.53 (0.13)	2.53 (0.14)	5.18	5.36	5.15	5.44	2.03 (0.06)	0.75 (0.46)	0.89 (0.39)	-0.41 (0.93)	-0.22 (1.35)	-0.20 (0.96)	0.83 (0.57)	1.05 (0.85)	0.73 (0.64)	0.99	0.97	0.98	0.98 (0.95-0.99)	0.97 (0.92-0.99)	0.98 (0.96-0.99)
Right superior parietal cortex	2.05 (0.16)	2.06 (0.16)	2.05 (0.17)	2.06 (0.17)	7.79	8.02	8.14	8.11	-1.05 (0.31)	0.45 (0.66)	-0.61 (0.55)	0.19 (0.88)	-0.20 (1.77)	0.12 (1.07)	0.73 (0.50)	1.30 (1.18)	0.87 (0.60)	0.99	0.98	0.99	0.99 (0.98-1.00)	0.98 (0.95-0.99)	0.99 (0.98-1.00)
Right superior temporal gyrus	2.57 (0.13)	2.56 (0.13)	2.57 (0.14)	2.56 (0.14)	4.90	5.14	5.49	5.43	1.45 (0.16)	0.08 (0.94)	0.90 (0.38)	-0.23 (0.75)	-0.04 (0.90)	-0.23 (1.17)	0.64 (0.44)	0.66 (0.60)	0.76 (0.90)	0.99	0.99	0.98	0.99 (0.97-1.00)	0.99 (0.97-0.99)	0.98 (0.95-0.99)
Right supramarginal gyrus	2.35 (0.09)	2.35 (0.10)	2.35 (0.08)	2.36 (0.10)	3.96	4.23	3.60	4.20	-0.40 (0.69)	0.04 (0.97)	-1.04 (0.31)	0.07 (0.95)	0.00 (1.53)	0.34 (1.58)	0.75 (0.57)	1.21 (0.90)	1.28 (0.95)	0.97	0.92	0.93	0.97 (0.94-0.99)	0.92 (0.82-0.97)	0.93 (0.83-0.97)
Right frontal pole	2.63 (0.26)	2.61 (0.24)	2.63 (0.26)	2.61 (0.25)	9.91	9.38	10.06	9.53	1.21 (0.24)	0.39 (0.70)	1.89 (0.07)	-0.90 (3.56)	-0.28 (3.13)	-1.02 (2.48)	2.90 (2.18)	2.30 (2.08)	2.29 (1.33)	0.93	0.95	0.97	0.93 (0.84-0.97)	0.96 (0.90-0.98)	0.96 (0.90-0.98)
Right temporal pole	3.38 (0.34)	3.43 (0.31)	3.36 (0.32)	3.47 (0.29)	10.08	9.02	9.61	8.25	-0.97 (0.34)	0.86 (0.40)	-1.71 (0.10)	1.32 (6.24)	-0.82 (4.90)	2.53 (6.87)	3.66 (5.17)	3.70 (3.23)	4.17 (5.97)	0.81	0.89	0.76	0.81 (0.60-0.91)	0.89 (0.75-0.95)	0.73 (0.46-0.88)
Right transverse temporal gyrus	2.34 (0.31)	2.33 (0.30)	2.34 (0.32)	2.35 (0.31)	13.30	13.09	13.72	13.36	1.04 (0.31)	-0.41 (0.69)	-0.92 (0.37)	-0.44 (2.29)	0.09 (1.69)	0.36 (1.98)	1.69 (1.57)	1.35 (0.98)	1.47 (1.34)	0.99	0.99	0.99	0.99 (0.97-0.99)	0.99 (0.98-1.00)	0.99 (0.98-1.00)
Right insula	2.69 (0.18)	2.70 (0.19)	2.68 (0.19)	2.73 (0.23)	6.74	7.22	7.04	8.26	-0.27 (0.79)	0.40 (0.69)	-1.95 (0.06)	0.19 (4.07)	-0.33 (3.58)	1.39 (3.70)	3.39 (2.14)	2.86 (2.09)	3.18 (2.25)	0.83	0.87	0.91	0.83 (0.64-0.93)	0.87 (0.71-0.94)	0.87 (0.70-0.95)
Global brain measures																							
Left total cortical volume (ml)	196.87 (23.92)	196.76 (23.89)	196.80 (23.98)	197.04 (23.93)	12.15	12.14	12.18	12.15	0.46 (0.65)	0.23 (0.82)	-0.75 (0.46)	-0.06 (0.58)	-0.04 (0.77)	0.09 (0.57)	0.48 (0.31)	0.56 (0.51)	0.45 (0.36)	1.00	1.00	1.00	1.00 (1.00-1.00)	1.00 (1.00-1.00)	1.00 (1.00-1.00)
Right total cortical volume (ml)	199.97 (25.16)	199.74 (25.08)	199.74 (25.18)	200.36	12.58	12.56	12.61	12.75	1.32 (0.20)	0.62 (0.54)	-1.67 (0.11)	-0.11 (0.40)	-0.12 (0.86)	0.17 (0.56)	0.30 (0.28)	0.68 (0.53)	0.50 (0.30)	1.00	1.00	1.00	1.00 (1.00-1.00)	1.00 (0.99-1.00)	1.00 (1.00-1.00)

				(25.55)																			
Left total cortical white matter (ml)	204.85 (34.21)	206.10 (35.09)	205.36 (33.72)	205.51 (33.73)	16.70	17.03	16.42	16.41	-2.50 (0.02)	-1.16 (0.26)	-1.99 (0.06)	0.57 (1.10)	0.30 (1.06)	0.37 (0.85)	0.99 (0.73)	0.80 (0.74)	0.74 (0.55)	1.00	1.00	1.00	1.00 (0.99-1.00)	1.00 (1.00-1.00)	1.00 (1.00-1.00)
Right total cortical white matter (ml)	205.81 (33.43)	206.33 (34.81)	206.45 (33.01)	206.61 (34.28)	16.24	16.87	15.99	16.59	-1.21 (0.24)	-1.42 (0.17)	-1.91 (0.07)	0.16 (0.96)	0.34 (0.99)	0.34 (0.91)	0.75 (0.60)	0.83 (0.62)	0.74 (0.61)	1.00	1.00	1.00	1.00 (1.00-1.00)	1.00 (0.99-1.00)	1.00 (1.00-1.00)
Left total cortical surface area (cm²)	764.44 (98.33)	767.48 (99.08)	765.73 (97.73)	767.93 (97.43)	12.86	12.91	12.76	12.69	-3.06 (0.01)	-1.34 (0.19)	-3.80 (0.00)	0.40 (0.66)	0.18 (0.65)	0.48 (0.64)	0.54 (0.54)	0.46 (0.49)	0.58 (0.55)	1.00	1.00	1.00	1.00 (0.99-1.00)	1.00 (1.00-1.00)	1.00 (0.99-1.00)
Right total cortical surface area (cm²)	770.18 (99.46)	771.90 (101.43)	771.59 (99.72)	773.14 (101.8 6)	12.91	13.14	12.92	13.17	-1.81 (0.09)	-1.32 (0.20)	-2.67 (0.01)	0.19 (0.57)	0.18 (0.63)	0.35 (0.64)	0.49 (0.33)	0.50 (0.40)	0.57 (0.44)	1.00	1.00	1.00	1.00 (1.00-1.00)	1.00 (1.00-1.00)	1.00 (0.99-1.00)
Left mean cortical thickness (mm)	2.31 (0.10)	2.31 (0.10)	2.31 (0.10)	2.31 (0.10)	4.21	4.31	4.31	4.12	1.13 (0.27)	0.39 (0.70)	1.11 (0.28)	-0.15 (0.60)	-0.07 (0.75)	-0.16 (0.69)	0.52 (0.31)	0.58 (0.47)	0.57 (0.40)	0.99	0.98	0.99	0.99 (0.98-1.00)	0.98 (0.96-0.99)	0.99 (0.97-0.99)
Right mean cortical tickness (mm)	2.33 (0.09)	2.32 (0.10)	2.32 (0.10)	2.33 (0.10)	4.07	4.21	4.28	4.24	1.37 (0.19)	1.03 (0.31)	-0.18 (0.86)	-0.14 (0.46)	-0.17 (0.75)	0.02 (0.59)	0.35 (0.32)	0.59 (0.47)	0.43 (0.39)	0.99	0.98	0.99	0.99 (0.98-1.00)	0.98 (0.96-0.99)	0.99 (0.98-1.00)
Left cerebellar white matter (ml)	14.80 (1.80)	14.79 (1.59)	14.92 (1.86)	14.25 (1.84)	12.15	10.78	12.48	12.89	0.04 (0.96)	-1.01 (0.33)	3.25 (0.00)	0.08 (3.76)	0.79 (3.79)	-3.90 (5.65)	2.80 (2.44)	2.97 (2.40)	5.05 (4.61)	0.94	0.95	0.90	0.94 (0.86-0.97)	0.95 (0.89-0.98)	0.87 (0.59-0.95)
Right cerebellar white matter (ml)	14.43 (1.70)	14.51 (1.69)	14.30 (1.73)	14.18 (2.31)	11.81	11.63	12.11	16.28	-0.46 (0.65)	1.27 (0.22)	0.66 (0.52)	0.52 (5.06)	-0.97 (3.71)	-2.35 (11.31)	3.26 (3.84)	2.90 (2.45)	7.00 (9.07)	0.90	0.96	0.62	0.90 (0.78-0.96)	0.96 (0.90-0.98)	0.60 (0.25-0.81)
Left cerebellar gray matter (ml)	49.83 (5.56)	49.69 (5.59)	49.71 (5.55)	50.35 (5.62)	11.17	11.26	11.17	11.15	1.11 (0.28)	1.37 (0.19)	-4.32 (0.00)	-0.29 (1.19)	-0.24 (0.79)	1.04 (1.08)	0.90 (0.81)	0.65 (0.50)	1.21 (0.87)	0.99	1.00	1.00	0.99 (0.99-1.00)	1.00 (0.99-1.00)	0.99 (0.93-1.00)
Right cerebellar gray matter (ml)	50.14 (5.92)	49.93 (5.86)	49.65 (5.78)	50.49 (5.98)	11.81	11.73	11.65	11.85	1.40 (0.18)	1.42 (0.17)	-1.29 (0.21)	-0.41 (1.33)	-0.96 (3.12)	0.71 (2.71)	1.18 (0.69)	1.60 (2.83)	1.89 (2.03)	0.99	0.96	0.98	0.99 (0.98-1.00)	0.96 (0.91-0.98)	0.98 (0.94-0.99)
Intracranial volume (ml)	1527.80 (236.78)	1527.88 (238.30)	1523.00 (238.59)	1540.39 (226.30)	15.50	15.60	15.67	14.69	-0.04 (0.97)	3.30 (0.00)	-2.42 (0.02)	-0.01 (0.74)	-0.33 (0.48)	0.93 (1.77)	0.38 (0.63)	0.39 (0.44)	1.01 (1.72)	1.00	1.00	1.00	1.00 (1.00-1.00)	1.00 (1.00-1.00)	0.99 (0.98-1.00)
Total brain volume without ventricles (ml)	987.57 (128.07)	988.90 (130.25)	987.85 (127.59)	989.36 (129.17)	12.97	13.17	12.92	13.06	-1.50 (0.15)	-0.23 (0.82)	-1.92 (0.07)	0.11 (0.40)	0.03 (0.61)	0.17 (0.44)	0.31 (0.26)	0.45 (0.41)	0.36 (0.29)	1.00	1.00	1.00	1.00 (1.00-1.00)	1.00 (1.00-1.00)	1.00 (1.00-1.00)

Abbreviations: SD = standard deviation; Orig = original scan; FS df = FreeSurfer defacing; FSL df = FSL defacing; FM = Face Masking; t = t-statistic; *p*=p-value; PD = Average Percentage Difference; CI = Confidence Interval.

Table S5. Effects of de-identification on brain measures in older adults without cognitive impairment.

Brain metric	Mean (SD)				Coefficient of Variation				Paired t-test t(*p*)			Signed PD in %			Absolute PD (%)			Pearson's r			ICC (95% CI)		
	Orig	FS df	FSL df	FM	Orig	FS df	FSL df	FM	FS df	FSL df	FM	FS df	FSL df	FM	FS df	FSL df	FM	FS df	FSL df	FM	FS df	FSL df	FM
Subcortical volume (ml)																							
Left thalamus	6.47 (0.63)	6.48 (0.61)	6.56 (0.59)	6.49 (0.66)	9.73	9.37	9.00	10.14	-0.44 (0.67)	-2.27 (0.03)	-0.53 (0.60)	0.18 (1.64)	1.36 (2.68)	0.28 (2.72)	1.44 (0.74)	2.18 (2.03)	2.32 (1.34)	0.99	0.96	0.96	0.99 (0.97-0.99)	0.95 (0.88-0.98)	0.96 (0.92-0.99)
Left caudatus	3.10 (0.31)	3.09 (0.32)	3.10 (0.31)	3.13 (0.31)	10.13	10.24	10.15	10.01	0.67 (0.51)	0.28 (0.78)	-1.54 (0.14)	-0.36 (2.35)	-0.13 (2.17)	0.85 (2.59)	1.87 (1.41)	1.71 (1.29)	2.07 (1.73)	0.97	0.98	0.97	0.97 (0.94-0.99)	0.98 (0.95-0.99)	0.97 (0.92-0.99)
Left putamen	4.27 (0.41)	4.28 (0.39)	4.24 (0.40)	4.27 (0.36)	9.55	9.02	9.40	8.45	-0.23 (0.82)	1.65 (0.11)	0.18 (0.86)	0.15 (1.78)	-0.76 (2.13)	-0.02 (3.17)	1.36 (1.12)	1.64 (1.52)	1.96 (2.46)	0.98	0.98	0.95	0.98 (0.95-0.99)	0.97 (0.93-0.99)	0.95 (0.88-0.98)
Left pallidum	1.92 (0.18)	1.94 (0.19)	1.93 (0.18)	1.90 (0.18)	9.18	9.91	9.52	9.66	-1.71 (0.10)	-0.84 (0.41)	1.62 (0.12)	1.02 (2.89)	0.59 (3.41)	-1.06 (2.89)	2.67 (1.39)	2.49 (2.34)	2.30 (1.98)	0.96	0.93	0.96	0.95 (0.88-0.98)	0.93 (0.85-0.97)	0.95 (0.88-0.98)
Left hippocampus	3.66 (0.42)	3.62 (0.42)	3.65 (0.43)	3.64 (0.41)	11.43	11.47	11.74	11.29	2.25 (0.04)	0.66 (0.51)	0.95 (0.35)	-0.90 (1.84)	-0.36 (2.14)	-0.44 (2.21)	1.55 (1.31)	1.45 (1.59)	1.70 (1.43)	0.99	0.98	0.98	0.98 (0.96-0.99)	0.98 (0.95-0.99)	0.98 (0.96-0.99)
Left amygdala	1.51 (0.22)	1.49 (0.22)	1.50 (0.22)	1.45 (0.22)	14.50	14.52	14.89	15.50	1.43 (0.17)	1.02 (0.32)	4.08 (0.00)	-1.14 (3.68)	-0.64 (2.71)	-3.86 (4.39)	2.91 (2.46)	2.22 (1.62)	4.27 (3.97)	0.97	0.98	0.96	0.97 (0.92-0.99)	0.98 (0.96-0.99)	0.93 (0.64-0.98)
Left accumbens	0.38 (0.07)	0.37 (0.06)	0.38 (0.07)	0.39 (0.06)	18.46	16.66	18.08	14.31	1.07 (0.30)	-0.24 (0.81)	-1.16 (0.26)	-1.98 (9.67)	0.58 (10.01)	2.68 (8.51)	7.22 (6.55)	8.10 (5.62)	6.52 (5.94)	0.86	0.85	0.90	0.86 (0.68-0.94)	0.85 (0.67-0.94)	0.88 (0.73-0.95)
Right thalamus	6.25 (0.55)	6.28 (0.53)	6.23 (0.49)	6.24 (0.56)	8.73	8.36	7.89	8.99	-1.08 (0.29)	0.49 (0.63)	0.17 (0.86)	0.52 (2.07)	-0.23 (2.85)	-0.12 (2.47)	1.69 (1.25)	2.29 (1.64)	1.99 (1.41)	0.97	0.95	0.96	0.97 (0.93-0.99)	0.95 (0.88-0.98)	0.97 (0.92-0.99)
Right caudatus	3.27 (0.35)	3.25 (0.31)	3.24 (0.32)	3.26 (0.35)	10.84	9.56	10.03	10.84	0.67 (0.51)	1.63 (0.12)	0.32 (0.76)	-0.39 (3.38)	-0.85 (2.43)	-0.32 (4.38)	2.48 (2.26)	1.69 (1.91)	2.89 (3.25)	0.95	0.97	0.91	0.94 (0.87-0.98)	0.97 (0.92-0.99)	0.91 (0.80-0.96)
Right putamen	4.31 (0.37)	4.34 (0.36)	4.34 (0.35)	4.30 (0.41)	8.56	8.35	8.13	9.63	-1.32 (0.20)	-1.49 (0.15)	0.36 (0.72)	0.74 (2.57)	0.85 (2.56)	-0.37 (3.70)	1.95 (1.78)	1.64 (2.12)	2.19 (2.96)	0.96	0.96	0.94	0.95 (0.89-0.98)	0.95 (0.88-0.98)	0.93 (0.84-0.97)
Right pallidum	1.84 (0.21)	1.85 (0.19)	1.85 (0.21)	1.84 (0.23)	11.39	10.18	11.50	12.31	-0.31 (0.76)	-0.36 (0.72)	-0.05 (0.96)	0.40 (4.17)	0.29 (3.66)	-0.06 (4.24)	2.84 (3.02)	2.98 (2.03)	3.53 (2.21)	0.94	0.95	0.94	0.94 (0.85-0.97)	0.95 (0.88-0.98)	0.94 (0.85-0.97)
Right hippocampus	3.78 (0.47)	3.78 (0.48)	3.80 (0.46)	3.80 (0.45)	12.49	12.68	12.04	11.97	0.50 (0.62)	-0.93 (0.36)	-0.88 (0.39)	-0.22 (1.70)	0.46 (1.97)	0.39 (1.66)	1.29 (1.09)	1.55 (1.26)	1.42 (0.90)	0.99	0.99	0.99	0.99 (0.98-1.00)	0.99 (0.97-0.99)	0.99 (0.98-1.00)
Right amygdala	1.72 (0.19)	1.72 (0.20)	1.73 (0.20)	1.69 (0.19)	10.97	11.56	11.32	11.46	-0.49 (0.63)	-1.87 (0.08)	1.47 (0.16)	0.37 (4.15)	1.01 (2.47)	-1.26 (3.81)	3.02 (2.80)	2.22 (1.41)	3.08 (2.49)	0.93	0.97	0.94	0.93 (0.84-0.97)	0.97 (0.92-0.99)	0.94 (0.86-0.98)
Right accumbens	0.47 (0.07)	0.46 (0.07)	0.48 (0.07)	0.48 (0.06)	15.26	15.53	15.45	12.34	1.68 (0.11)	-1.75 (0.10)	-1.22 (0.24)	-2.26 (6.71)	2.22 (6.18)	2.35 (8.08)	5.82 (3.86)	5.16 (3.91)	6.60 (5.04)	0.92	0.93	0.87	0.91 (0.80-0.96)	0.92 (0.82-0.97)	0.86 (0.68-0.94)
Ventricular volume (ml)																							
Third ventricle	1.74 (0.76)	1.74 (0.77)	1.73 (0.76)	1.74 (0.76)	43.66	43.97	43.92	43.41	-0.78 (0.44)	1.53 (0.14)	-0.19 (0.85)	0.13 (2.02)	-0.70 (1.79)	0.25 (3.34)	1.50 (1.32)	1.58 (1.05)	2.46 (2.22)	1.00	1.00	1.00	1.00 (1.00-1.00)	1.00 (1.00-1.00)	1.00 (0.99-1.00)
Left lateral ventricle	19.10 (10.03)	19.10 (10.10)	19.13 (10.06)	19.09 (10.08)	52.51	52.87	52.57	52.80	0.08 (0.93)	-0.86 (0.40)	0.41 (0.69)	-0.19 (0.92)	0.10 (0.70)	-0.19 (0.71)	0.64 (0.68)	0.57 (0.41)	0.59 (0.42)	1.00	1.00	1.00	1.00 (1.00-1.00)	1.00 (1.00-1.00)	1.00 (1.00-1.00)
Left inferior lateral ventricle	0.62 (0.43)	0.61 (0.43)	0.62 (0.42)	0.60 (0.41)	69.20	70.22	68.43	68.67	0.58 (0.57)	0.21 (0.83)	1.38 (0.18)	-2.29 (17.43)	-0.57 (16.52)	-4.89 (16.32)	11.86 (12.71)	10.44 (12.61)	10.59 (13.18)	0.99	0.99	0.99	0.99 (0.97-1.00)	0.99 (0.98-1.00)	0.99 (0.97-0.99)
Right lateral ventricle	16.15 (7.85)	16.14 (7.85)	16.17 (7.91)	16.15 (7.90)	48.60	48.64	48.90	48.88	0.48 (0.64)	-0.84 (0.41)	-0.17 (0.87)	-0.13 (1.04)	-0.03 (0.64)	-0.12 (1.01)	0.60 (0.85)	0.49 (0.40)	0.77 (0.64)	1.00	1.00	1.00	1.00 (1.00-1.00)	1.00 (1.00-1.00)	1.00 (1.00-1.00)
Right inferior lateral ventricle	0.56 (0.35)	0.57 (0.36)	0.56 (0.36)	0.56 (0.36)	62.94	62.73	63.24	63.50	-0.95 (0.35)	-0.11 (0.91)	0.04 (0.97)	2.00 (8.94)	-0.35 (12.78)	-0.82 (10.26)	6.14 (6.67)	8.57 (9.29)	7.17 (7.22)	1.00	0.99	0.99	1.00 (0.99-1.00)	0.99 (0.98-1.00)	0.99 (0.99-1.00)
Local cortical volume (ml)																							
Left banks superior temporal sulcus	2.16 (0.25)	2.11 (0.24)	2.13 (0.26)	2.17 (0.27)	11.51	11.56	12.16	12.28	1.76 (0.09)	1.64 (0.12)	-0.57 (0.57)	-2.32 (6.43)	-1.43 (4.18)	0.40 (4.16)	4.14 (5.39)	3.12 (3.08)	3.14 (2.67)	0.86	0.95	0.94	0.85 (0.66-0.94)	0.95 (0.87-0.98)	0.94 (0.87-0.98)
Left caudal anterior cingulate cortex	1.49 (0.38)	1.49 (0.39)	1.49 (0.41)	1.51 (0.38)	25.49	26.39	27.32	25.35	0.42 (0.68)	0.29 (0.77)	-1.95 (0.06)	-0.66 (4.85)	-0.77 (6.36)	1.39 (3.60)	3.38 (3.46)	3.77 (5.12)	2.83 (2.56)	0.99	0.98	0.99	0.99 (0.97-1.00)	0.98 (0.95-0.99)	0.99 (0.98-1.00)
Left caudal middle frontal gyrus	5.50 (0.85)	5.49 (0.86)	5.49 (0.88)	5.51 (0.88)	15.51	15.68	16.00	16.04	0.62 (0.54)	0.53 (0.60)	-0.09 (0.93)	-0.29 (2.03)	-0.35 (2.32)	-0.04 (2.39)	1.55 (1.30)	1.71 (1.56)	1.98 (1.26)	0.99	0.99	0.99	0.99 (0.98-1.00)	0.99 (0.98-1.00)	0.99 (0.97-1.00)
Left cuneus	2.70 (0.44)	2.73 (0.44)	2.74 (0.43)	2.75 (0.45)	16.34	16.02	15.50	16.23	-1.37 (0.19)	-1.29 (0.21)	-0.98 (0.34)	0.88 (2.93)	1.60 (5.63)	1.55 (7.52)	2.09 (2.20)	2.93 (5.03)	3.05 (7.02)	0.98	0.95	0.90	0.98 (0.96-0.99)	0.94 (0.87-0.98)	0.90 (0.77-0.96)
Left entorhinal cortex	2.01 (0.47)	2.01 (0.43)	1.98 (0.44)	2.03 (0.44)	23.42	21.45	22.30	21.63	-0.04 (0.97)	1.26 (0.22)	-0.68 (0.51)	0.31 (9.04)	-1.14 (4.75)	1.34 (7.25)	7.48 (4.81)	3.71 (3.07)	5.43 (4.84)	0.93	0.98	0.96	0.93 (0.84-0.97)	0.98 (0.95-0.99)	0.96 (0.90-0.98)
Left fusiform gyrus	8.90 (1.00)	8.84 (1.01)	8.85 (0.97)	8.84 (1.04)	11.27	11.46	10.98	11.72	1.06 (0.30)	1.10 (0.29)	1.41 (0.17)	-0.69 (2.84)	-0.59 (2.57)	-0.74 (2.18)	2.20 (1.86)	2.09 (1.54)	1.93 (1.19)	0.97	0.97	0.98	0.97 (0.92-0.99)	0.97 (0.93-0.99)	0.98 (0.95-0.99)
Left inferior parietal cortex	10.29 (1.28)	10.07 (1.47)	10.26 (1.34)	10.30 (1.34)	12.46	14.64	13.07	13.05	1.47 (0.16)	0.70 (0.49)	-0.25 (0.81)	-2.50 (8.10)	-0.37 (1.92)	0.04 (1.90)	3.55 (7.67)	1.55 (1.16)	1.32 (1.33)	0.89	0.99	0.99	0.87 (0.71-0.95)	0.99 (0.97-1.00)	0.99 (0.97-1.00)
Left inferior temporal gyrus	10.16 (1.22)	10.09 (1.29)	10.15 (1.31)	10.18 (1.25)	11.99	12.83	12.93	12.29	1.85 (0.08)	0.24 (0.81)	-0.53 (0.60)	-0.85 (1.81)	-0.22 (2.18)	0.20 (2.09)	1.47 (1.33)	1.72 (1.30)	1.31 (1.61)	0.99	0.99	0.99	0.99 (0.97-1.00)	0.99 (0.96-0.99)	0.99 (0.97-1.00)
Left isthmus cingulate cortex	2.32 (0.28)	2.30 (0.28)	2.28 (0.31)	2.31 (0.31)	12.08	12.18	13.59	13.61	0.44 (0.66)	1.72 (0.10)	0.50 (0.63)	-0.56 (6.07)	-1.72 (4.23)	-0.62 (3.97)	4.21 (4.32)	3.45 (2.92)	2.87 (2.74)	0.88	0.96	0.97	0.88 (0.74-0.95)	0.95 (0.87-0.98)	0.96 (0.91-0.98)
Left lateral occipital cortex	10.77 (1.37)	10.88 (1.37)	10.77 (1.38)	10.84 (1.34)	12.68	12.55	12.82	12.36	-1.42 (0.17)	-0.02 (0.99)	-1.15 (0.26)	1.05 (3.53)	0.00 (2.89)	0.64 (2.56)	2.11 (3.00)	1.96 (2.08)	1.85 (1.85)	0.97	0.98	0.98	0.96 (0.91-0.99)	0.98 (0.95-0.99)	0.98 (0.96-0.99)
Left lateral orbitofrontal cortex	6.87 (0.59)	6.84 (0.64)	6.83 (0.61)	6.86 (0.64)	8.55	9.39	8.87	9.30	0.87 (0.40)	1.62 (0.12)	0.13 (0.90)	-0.46 (2.06)	-0.52 (1.47)	-0.13 (2.15)	1.65 (1.27)	1.15 (1.03)	1.63 (1.36)	0.98	0.99	0.98	0.97 (0.94-0.99)	0.99 (0.97-0.99)	0.97 (0.94-0.99)
Left lingual gyrus	6.05 (0.70)	6.10 (0.68)	6.05 (0.72)	6.09 (0.72)	11.56	11.18	11.94	11.84	-1.23 (0.23)	-0.25 (0.80)	-2.34 (0.03)	0.93 (3.60)	0.05 (1.60)	0.59 (1.14)	2.03 (3.09)	1.31 (0.87)	0.95 (0.85)	0.96	0.99	1.00	0.96 (0.90-0.98)	0.99 (0.98-1.00)	0.99 (0.98-1.00)
Left medial orbitofrontal cortex	4.65 (0.58)	4.59 (0.56)	4.53 (0.60)	4.68 (0.59)	12.42	12.20	13.18	12.65	1.10 (0.28)	2.35 (0.03)	-0.72 (0.48)	-1.19 (5.35)	-2.51 (4.71)	0.63 (4.25)	4.11 (3.52)	4.25 (3.14)	3.53 (2.32)	0.92	0.93	0.94	0.91 (0.80-0.96)	0.92 (0.78-0.97)	0.94 (0.87-0.98)

Left middle temporal gyrus	9.82 (1.01)	9.83 (1.13)	9.82 (1.06)	9.81 (1.04)	10.32	11.52	10.83	10.65	-0.12 (0.91)	-0.03 (0.98)	0.28 (0.78)	-0.02 (4.18)	-0.03 (2.04)	-0.13 (1.68)	2.19 (3.52)	1.50 (1.34)	1.27 (1.08)	0.94	0.98	0.99	0.94 (0.86-0.98)	0.98 (0.96-0.99)	0.99 (0.97-0.99)
Left parahippocampal gyrus	2.01 (0.34)	2.00 (0.37)	2.01 (0.37)	2.02 (0.40)	16.72	18.66	18.28	19.87	0.27 (0.79)	-0.45 (0.66)	-0.66 (0.51)	-0.57 (4.53)	0.13 (3.56)	0.33 (5.14)	3.24 (3.13)	3.03 (1.75)	4.01 (3.12)	0.97	0.98	0.97	0.97 (0.93-0.99)	0.98 (0.95-0.99)	0.96 (0.89-0.98)
Left paracentral lobule	3.32 (0.46)	3.31 (0.49)	3.32 (0.49)	3.30 (0.47)	13.82	14.97	14.76	14.34	0.34 (0.74)	-0.27 (0.79)	0.73 (0.48)	-0.39 (3.55)	0.12 (3.37)	-0.52 (2.84)	2.92 (1.95)	2.66 (1.98)	2.48 (1.36)	0.97	0.97	0.98	0.97 (0.92-0.99)	0.97 (0.93-0.99)	0.98 (0.95-0.99)
Left pars opercularis	4.03 (0.54)	4.06 (0.57)	4.05 (0.57)	4.07 (0.54)	13.46	14.14	14.12	13.18	-0.73 (0.48)	-0.43 (0.67)	-1.14 (0.27)	0.66 (4.11)	0.16 (2.88)	0.80 (2.89)	2.91 (2.90)	2.29 (1.68)	2.33 (1.82)	0.95	0.98	0.97	0.95 (0.88-0.98)	0.98 (0.95-0.99)	0.97 (0.94-0.99)
Left pars orbitalis	2.17 (0.27)	2.19 (0.30)	2.20 (0.29)	2.19 (0.27)	12.57	13.65	13.25	12.31	-0.63 (0.54)	-1.58 (0.13)	-0.85 (0.40)	0.47 (4.38)	1.04 (3.26)	0.72 (3.78)	3.44 (2.65)	2.68 (2.05)	2.77 (2.61)	0.95	0.97	0.95	0.95 (0.87-0.98)	0.97 (0.92-0.99)	0.96 (0.89-0.98)
Left pars triangularis	3.20 (0.51)	3.18 (0.52)	3.17 (0.50)	3.20 (0.50)	15.94	16.25	15.66	15.69	0.89 (0.38)	1.83 (0.08)	-0.08 (0.94)	-0.62 (3.12)	-0.81 (2.21)	0.10 (3.72)	2.46 (1.95)	1.75 (1.53)	3.08 (1.98)	0.98	0.99	0.97	0.98 (0.96-0.99)	0.99 (0.97-1.00)	0.97 (0.94-0.99)
Left pericalcarine cortex	1.90 (0.26)	1.98 (0.35)	1.89 (0.26)	1.91 (0.25)	13.55	17.90	13.78	13.26	-0.92 (0.37)	1.11 (0.28)	-0.62 (0.54)	3.45 (17.11)	-0.78 (3.16)	0.50 (3.35)	6.14 (16.30)	2.75 (1.64)	2.64 (2.05)	0.21	0.97	0.97	0.20 (-0.24-0.58)	0.97 (0.94-0.99)	0.97 (0.92-0.99)
Left postcentral gyrus	8.51 (1.17)	8.49 (1.18)	8.49 (1.17)	8.52 (1.17)	13.72	13.89	13.78	13.75	0.31 (0.76)	0.78 (0.44)	-0.37 (0.71)	-0.23 (3.17)	-0.24 (1.36)	0.15 (1.87)	1.88 (2.52)	1.01 (0.91)	1.46 (1.13)	0.98	1.00	0.99	0.98 (0.95-0.99)	1.00 (0.99-1.00)	0.99 (0.98-1.00)
Left posterior cingulate cortex	2.77 (0.33)	2.74 (0.34)	2.73 (0.33)	2.80 (0.35)	11.77	12.52	11.93	12.35	0.95 (0.36)	1.61 (0.12)	-0.81 (0.43)	-1.15 (5.01)	-1.68 (4.45)	0.81 (4.63)	3.89 (3.26)	3.52 (3.12)	3.74 (2.74)	0.91	0.92	0.92	0.91 (0.80-0.96)	0.92 (0.80-0.97)	0.92 (0.81-0.96)
Left precentral gyrus	12.55 (1.50)	12.55 (1.46)	12.58 (1.49)	12.56 (1.51)	11.92	11.68	11.81	12.03	0.08 (0.94)	-0.55 (0.59)	-0.12 (0.90)	0.01 (1.64)	0.22 (1.63)	0.05 (1.97)	1.37 (0.85)	1.32 (0.95)	1.54 (1.18)	0.99	0.99	0.99	0.99 (0.98-1.00)	0.99 (0.98-1.00)	0.99 (0.97-0.99)
Left precuneus	8.45 (0.90)	8.38 (0.99)	8.43 (0.96)	8.43 (0.95)	10.66	11.87	11.35	11.32	1.18 (0.25)	0.41 (0.69)	0.43 (0.67)	-0.86 (3.06)	-0.24 (1.98)	-0.26 (2.18)	1.96 (2.47)	1.36 (1.43)	1.57 (1.49)	0.97	0.99	0.98	0.97 (0.92-0.99)	0.98 (0.96-0.99)	0.98 (0.96-0.99)
Left rostral anterior cingulate cortex	2.39 (0.45)	2.36 (0.41)	2.37 (0.47)	2.38 (0.46)	18.98	17.51	19.61	19.35	1.38 (0.18)	1.25 (0.23)	0.68 (0.50)	-0.91 (4.54)	-0.90 (3.05)	-0.72 (4.10)	3.46 (2.99)	2.53 (1.84)	3.23 (2.53)	0.98	0.99	0.98	0.97 (0.94-0.99)	0.99 (0.97-1.00)	0.98 (0.95-0.99)
Left rostral middle frontal gyrus	13.40 (1.58)	13.32 (1.74)	13.41 (1.62)	13.40 (1.59)	11.76	13.04	12.08	11.88	0.89 (0.38)	-0.06 (0.95)	0.06 (0.95)	-0.78 (3.52)	-0.01 (2.16)	-0.05 (2.21)	2.16 (2.85)	1.85 (1.03)	1.80 (1.23)	0.97	0.98	0.98	0.97 (0.92-0.99)	0.98 (0.96-0.99)	0.98 (0.96-0.99)
Left superior frontal gyrus	20.14 (2.46)	20.04 (2.53)	20.13 (2.55)	20.12 (2.50)	12.20	12.65	12.68	12.44	1.07 (0.30)	0.10 (0.92)	0.29 (0.78)	-0.53 (2.14)	-0.09 (1.51)	-0.13 (1.84)	1.22 (1.82)	0.99 (1.12)	1.24 (1.33)	0.99	0.99	0.99	0.99 (0.97-0.99)	0.99 (0.99-1.00)	0.99 (0.97-1.00)
Left superior parietal cortex	11.55 (1.43)	11.47 (1.74)	11.54 (1.42)	11.59 (1.39)	12.41	15.15	12.27	12.02	0.61 (0.55)	0.25 (0.81)	-1.35 (0.19)	-1.15 (7.11)	-0.05 (1.44)	0.41 (1.25)	2.45 (6.76)	1.16 (0.82)	1.01 (0.81)	0.95	0.99	1.00	0.94 (0.85-0.97)	0.99 (0.99-1.00)	0.99 (0.99-1.00)
Left superior temporal gyrus	11.17 (1.43)	11.17 (1.48)	11.18 (1.46)	11.19 (1.47)	12.82	13.25	13.10	13.14	-0.17 (0.87)	-0.33 (0.75)	-0.59 (0.56)	0.01 (1.75)	0.09 (1.82)	0.19 (1.80)	1.21 (1.23)	1.44 (1.07)	1.35 (1.17)	0.99	0.99	0.99	0.99 (0.98-1.00)	0.99 (0.98-1.00)	0.99 (0.98-1.00)
Left supramarginal gyrus	10.06 (1.35)	9.99 (1.50)	10.10 (1.43)	10.07 (1.40)	13.46	15.02	14.13	13.94	0.50 (0.62)	-0.94 (0.36)	-0.18 (0.86)	-0.89 (6.60)	0.35 (2.05)	0.02 (2.06)	2.97 (5.92)	1.54 (1.36)	1.57 (1.30)	0.91	0.99	0.99	0.91 (0.79-0.96)	0.99 (0.97-1.00)	0.99 (0.97-1.00)
Left frontal pole	0.88 (0.13)	0.88 (0.15)	0.89 (0.15)	0.88 (0.15)	14.71	17.35	16.39	16.84	0.10 (0.92)	-0.78 (0.45)	-0.01 (0.99)	-0.58 (8.61)	0.94 (7.68)	-0.27 (6.38)	6.69 (5.24)	6.24 (4.36)	4.80 (4.08)	0.87	0.89	0.93	0.86 (0.69-0.94)	0.89 (0.75-0.95)	0.92 (0.82-0.97)
Left temporal pole	2.44 (0.30)	2.38 (0.28)	2.49 (0.28)	2.42 (0.28)	12.41	11.74	11.20	11.36	1.64 (0.12)	-1.41 (0.17)	0.32 (0.76)	-2.32 (6.59)	2.45 (7.68)	-0.51 (9.06)	5.63 (3.97)	6.62 (4.38)	6.98 (5.58)	0.85	0.80	0.71	0.84 (0.64-0.93)	0.79 (0.56-0.91)	0.72 (0.42-0.88)
Left transverse temporal gyrus	1.01 (0.18)	1.00 (0.18)	1.01 (0.19)	1.01 (0.18)	17.47	18.35	19.00	17.64	0.27 (0.79)	-0.15 (0.89)	-0.41 (0.69)	-0.32 (3.86)	-0.11 (4.34)	0.34 (3.95)	2.85 (2.55)	3.40 (2.59)	3.04 (2.45)	0.98	0.98	0.98	0.98 (0.94-0.99)	0.97 (0.94-0.99)	0.98 (0.94-0.99)
Left insula	6.70 (0.66)	6.72 (0.66)	6.73 (0.63)	6.67 (0.61)	9.84	9.83	9.31	9.17	-0.41 (0.69)	-0.58 (0.57)	0.47 (0.64)	0.26 (3.06)	0.51 (3.62)	-0.37 (4.15)	2.35 (1.92)	2.53 (2.59)	2.81 (3.02)	0.95	0.93	0.91	0.95 (0.89-0.98)	0.93 (0.83-0.97)	0.91 (0.79-0.96)
Right banks superior temporal sulcus	1.99 (0.30)	1.95 (0.34)	1.96 (0.27)	1.98 (0.31)	14.89	17.22	14.03	15.57	1.85 (0.08)	1.55 (0.14)	0.37 (0.72)	-2.56 (6.23)	-1.49 (4.93)	-0.39 (3.84)	4.33 (5.10)	3.99 (3.16)	3.07 (2.24)	0.95	0.95	0.97	0.94 (0.85-0.97)	0.94 (0.86-0.98)	0.97 (0.92-0.99)
Right caudal anterior cingulate cortex	1.92 (0.40)	1.91 (0.42)	1.91 (0.39)	1.88 (0.37)	20.61	22.19	20.25	19.78	0.15 (0.88)	0.65 (0.52)	2.11 (0.05)	-0.49 (3.88)	-0.48 (4.09)	-1.73 (4.36)	2.91 (2.54)	3.05 (2.67)	3.32 (3.26)	0.99	0.98	0.98	0.99 (0.96-0.99)	0.98 (0.95-0.99)	0.98 (0.93-0.99)
Right caudal middle frontal gyrus	5.47 (0.90)	5.39 (0.99)	5.44 (0.95)	5.43 (0.93)	16.45	18.41	17.56	17.07	2.06 (0.05)	1.36 (0.19)	1.33 (0.20)	-1.76 (3.83)	-0.81 (2.36)	-0.76 (2.36)	2.34 (3.49)	1.79 (1.70)	2.08 (1.28)	0.99	0.99	0.99	0.98 (0.95-0.99)	0.99 (0.98-1.00)	0.99 (0.98-1.00)
Right cuneus	2.94 (0.63)	3.07 (0.67)	2.98 (0.56)	3.02 (0.62)	21.47	21.95	18.90	20.66	-1.47 (0.16)	-0.95 (0.35)	-1.85 (0.08)	4.42 (13.49)	2.11 (8.70)	2.96 (7.85)	6.06 (12.81)	4.55 (7.66)	4.22 (7.22)	0.78	0.93	0.95	0.77 (0.52-0.90)	0.92 (0.82-0.97)	0.94 (0.85-0.98)
Right entorhinal cortex	1.86 (0.41)	1.85 (0.38)	1.84 (0.42)	1.89 (0.43)	21.78	20.51	22.88	22.95	0.26 (0.80)	0.36 (0.72)	-1.14 (0.27)	-0.26 (9.08)	-1.05 (10.08)	1.50 (7.18)	7.56 (4.75)	7.87 (6.15)	5.86 (4.22)	0.91	0.88	0.95	0.92 (0.80-0.96)	0.88 (0.74-0.95)	0.95 (0.88-0.98)
Right fusiform gyrus	8.32 (1.00)	8.26 (1.08)	8.27 (0.95)	8.30 (0.95)	11.98	13.02	11.54	11.50	1.68 (0.11)	1.45 (0.16)	0.57 (0.58)	-0.91 (2.32)	-0.52 (1.77)	-0.22 (2.20)	1.56 (1.91)	1.36 (1.22)	1.74 (1.30)	0.99	0.99	0.98	0.98 (0.96-0.99)	0.99 (0.97-1.00)	0.98 (0.96-0.99)
Right inferior parietal cortex	11.98 (1.55)	11.74 (1.74)	11.83 (1.35)	11.91 (1.59)	12.95	14.79	11.40	13.35	1.62 (0.12)	1.79 (0.09)	1.08 (0.29)	-2.39 (7.59)	-1.12 (3.06)	-0.65 (2.66)	3.38 (7.18)	2.20 (2.37)	1.97 (1.85)	0.92	0.97	0.98	0.91 (0.78-0.96)	0.96 (0.90-0.98)	0.98 (0.96-0.99)
Right inferior temporal gyrus	9.93 (1.60)	9.84 (1.60)	9.86 (1.59)	9.90 (1.53)	16.15	16.29	16.09	15.48	2.01 (0.06)	1.51 (0.15)	0.70 (0.49)	-0.95 (2.58)	-0.72 (2.21)	-0.16 (1.78)	1.85 (2.00)	1.72 (1.53)	1.41 (1.06)	0.99	0.99	0.99	0.99 (0.97-1.00)	0.99 (0.98-1.00)	0.99 (0.99-1.00)
Right isthmus cingulate cortex	2.13 (0.29)	2.12 (0.30)	2.11 (0.32)	2.12 (0.33)	13.50	14.35	15.32	15.40	0.13 (0.90)	0.45 (0.65)	0.35 (0.73)	-0.28 (6.13)	-1.06 (8.54)	-0.85 (7.77)	4.58 (3.96)	5.97 (6.06)	5.79 (5.10)	0.91	0.88	0.90	0.92 (0.80-0.97)	0.88 (0.72-0.95)	0.90 (0.77-0.96)
Right lateral occipital cortex	11.16 (1.59)	11.03 (1.83)	11.11 (1.57)	11.22 (1.64)	14.20	16.63	14.15	14.59	0.92 (0.37)	0.62 (0.54)	-0.87 (0.40)	-1.55 (7.07)	-0.48 (3.57)	0.42 (2.66)	3.11 (6.50)	1.99 (2.98)	2.00 (1.75)	0.94	0.97	0.98	0.93 (0.84-0.97)	0.97 (0.92-0.99)	0.98 (0.96-0.99)
Right lateral orbitofrontal cortex	6.66 (0.67)	6.70 (0.77)	6.67 (0.70)	6.64 (0.72)	9.99	11.55	10.50	10.83	-0.68 (0.50)	-0.34 (0.74)	0.26 (0.80)	0.48 (4.02)	0.19 (3.08)	-0.27 (3.26)	2.92 (2.73)	2.22 (2.09)	2.57 (1.95)	0.93	0.95	0.95	0.93 (0.83-0.97)	0.96 (0.89-0.98)	0.95 (0.89-0.98)
Right lingual gyrus	6.54 (0.89)	6.60 (0.88)	6.55 (0.87)	6.57 (0.88)	13.67	13.32	13.34	13.39	-1.09 (0.29)	-0.20 (0.84)	-1.06 (0.30)	0.94 (4.09)	0.14 (2.34)	0.49 (1.98)	2.44 (3.38)	1.71 (1.56)	1.55 (1.29)	0.96	0.99	0.99	0.96 (0.91-0.98)	0.99 (0.97-0.99)	0.99 (0.97-1.00)
Right medial orbitofrontal cortex	4.81 (0.54)	4.80 (0.50)	4.75 (0.50)	4.81 (0.53)	11.30	10.45	10.60	11.10	0.35 (0.73)	1.46 (0.16)	-0.14 (0.89)	-0.15 (3.09)	-1.11 (3.68)	0.11 (4.01)	2.36 (1.93)	3.20 (2.01)	3.18 (2.34)	0.97	0.95	0.94	0.96 (0.91-0.99)	0.94 (0.86-0.98)	0.94 (0.86-0.98)
Right middle temporal gyrus	10.83 (1.17)	10.79 (1.34)	10.86 (1.19)	10.87 (1.13)	10.81	12.46	10.98	10.42	0.46 (0.65)	-0.93 (0.36)	-1.07 (0.30)	-0.59 (4.59)	0.27 (1.30)	0.49 (2.03)	1.88 (4.20)	1.02 (0.81)	1.45 (1.47)	0.96	0.99	0.98	0.95 (0.89-0.98)	0.99 (0.98-1.00)	0.98 (0.96-0.99)
Right parahippocampal gyrus	1.84 (0.26)	1.84 (0.25)	1.86 (0.26)	1.84 (0.26)	14.35	13.80	13.95	14.17	0.31 (0.76)	-1.33 (0.20)	-0.05 (0.96)	-0.25 (4.53)	0.84 (2.84)	0.06 (5.24)	3.39 (2.92)	2.39 (1.68)	3.69 (3.63)	0.95	0.98	0.94	0.95 (0.89-0.98)	0.98 (0.95-0.99)	0.94 (0.86-0.98)
Right paracentral lobule	3.67 (0.46)	3.67 (0.44)	3.69 (0.46)	3.69 (0.47)	12.51	12.09	12.51	12.68	0.07 (0.94)	-1.14 (0.27)	-0.88 (0.39)	0.00 (2.90)	0.59 (2.40)	0.46 (2.41)	2.14 (1.90)	1.81 (1.65)	1.93 (1.46)	0.97	0.98	0.98	0.97 (0.93-0.99)	0.98 (0.95-0.99)	0.98 (0.95-0.99)
Right pars opercularis	3.41 (0.52)	3.41 (0.53)	3.44 (0.53)	3.42 (0.52)	15.29	15.51	15.48	15.21	-0.30 (0.77)	-2.14 (0.05)	-0.41 (0.69)	0.14 (3.29)	1.09 (2.51)	0.31 (3.41)	2.40 (2.19)	2.11 (1.69)	2.91 (1.70)	0.98	0.99	0.98	0.98 (0.96-0.99)	0.99 (0.96-0.99)	0.98 (0.95-0.99)
Right pars orbitalis	2.43 (0.30)	2.43 (0.31)	2.42 (0.27)	2.43 (0.29)	12.20	12.77	11.27	11.77	-0.10 (0.92)	0.46 (0.65)	-0.37 (0.72)	-0.01 (3.12)	-0.21 (2.76)	0.30 (3.37)	2.04 (2.31)	2.28 (1.48)	2.53 (2.18)	0.97	0.97	0.96	0.97 (0.94-0.99)	0.97 (0.93-0.99)	0.96 (0.91-0.98)
Right pars triangularis	3.74 (0.61)	3.74 (0.59)	3.72 (0.58)	3.74 (0.59)	16.27	15.80	15.74	15.81	0.08 (0.93)	1.05 (0.31)	-0.06 (0.95)	0.03 (3.10)	-0.54 (2.93)	0.10 (2.26)	2.30 (2.01)	2.37 (1.73)	1.66 (1.49)	0.98	0.99	0.99	0.98 (0.96-0.99)	0.98 (0.96-0.99)	0.99 (0.98-1.00)

Right pericalcarine cortex	2.24 (0.37)	2.16 (0.34)	2.17 (0.30)	2.18 (0.32)	16.34	15.70	13.94	14.59	1.31 (0.20)	1.66 (0.11)	1.22 (0.24)	-3.50 (12.50)	-2.89 (8.27)	-2.32 (9.05)	6.07 (11.42)	5.32 (6.89)	5.09 (7.77)	0.69	0.84	0.81	0.68 (0.38-0.86)	0.81 (0.60-0.92)	0.80 (0.57-0.91)
Right postcentral gyrus	8.34 (1.19)	8.32 (1.21)	8.34 (1.20)	8.32 (1.14)	14.21	14.58	14.44	13.73	0.53 (0.61)	0.18 (0.86)	0.88 (0.39)	-0.31 (2.54)	-0.08 (1.61)	-0.23 (1.53)	1.44 (2.09)	1.20 (1.04)	1.17 (0.99)	0.99	0.99	0.99	0.99 (0.97-1.00)	0.99 (0.99-1.00)	0.99 (0.99-1.00)
Right posterior cingulate cortex	2.81 (0.54)	2.85 (0.52)	2.85 (0.51)	2.82 (0.52)	19.04	18.14	18.07	18.37	-0.90 (0.38)	-0.85 (0.40)	-0.27 (0.79)	1.38 (7.34)	1.31 (7.29)	0.34 (7.33)	4.70 (5.72)	3.79 (6.32)	4.64 (5.59)	0.93	0.93	0.94	0.93 (0.85-0.97)	0.93 (0.84-0.97)	0.94 (0.87-0.98)
Right precentral gyrus	12.20 (1.26)	12.27 (1.24)	12.17 (1.25)	12.19 (1.25)	10.30	10.10	10.30	10.22	-1.15 (0.26)	0.66 (0.51)	0.19 (0.85)	0.55 (2.10)	-0.25 (1.75)	-0.06 (1.67)	1.83 (1.10)	1.44 (0.97)	1.27 (1.05)	0.98	0.99	0.99	0.98 (0.95-0.99)	0.99 (0.97-0.99)	0.99 (0.97-0.99)
Right precuneus	8.91 (0.92)	8.90 (0.96)	8.87 (0.94)	8.88 (0.97)	10.27	10.78	10.58	10.98	0.47 (0.64)	1.12 (0.27)	1.01 (0.32)	-0.24 (1.97)	-0.47 (1.79)	-0.45 (1.72)	1.41 (1.36)	1.41 (1.16)	1.41 (1.04)	0.98	0.99	0.99	0.98 (0.96-0.99)	0.98 (0.96-0.99)	0.99 (0.97-0.99)
Right rostral anterior cingulate cortex	1.91 (0.46)	1.91 (0.46)	1.91 (0.48)	1.91 (0.46)	24.39	24.15	25.14	23.89	-0.11 (0.91)	-0.06 (0.95)	-0.03 (0.98)	0.04 (4.68)	-0.20 (4.77)	0.04 (4.52)	3.88 (2.46)	3.50 (3.16)	3.47 (2.79)	0.98	0.98	0.98	0.98 (0.95-0.99)	0.98 (0.95-0.99)	0.98 (0.96-0.99)
Right rostral middle frontal gyrus	13.88 (1.61)	13.90 (1.74)	13.99 (1.66)	14.01 (1.61)	11.63	12.53	11.90	11.50	-0.20 (0.84)	-2.23 (0.04)	-2.53 (0.02)	-0.01 (2.56)	0.71 (1.73)	0.89 (1.71)	1.61 (1.96)	1.53 (1.04)	1.61 (1.01)	0.98	0.99	0.99	0.98 (0.96-0.99)	0.99 (0.97-1.00)	0.99 (0.96-1.00)
Right superior frontal gyrus	19.36 (2.33)	19.21 (2.43)	19.33 (2.40)	19.29 (2.44)	12.03	12.66	12.41	12.62	0.98 (0.34)	0.41 (0.69)	0.75 (0.46)	-0.84 (3.56)	-0.19 (1.61)	-0.40 (2.06)	1.99 (3.04)	1.05 (1.22)	1.52 (1.41)	0.96	0.99	0.99	0.96 (0.90-0.98)	0.99 (0.98-1.00)	0.99 (0.97-0.99)
Right superior parietal cortex	11.44 (1.45)	11.31 (1.53)	11.43 (1.37)	11.44 (1.42)	12.67	13.50	11.95	12.39	1.70 (0.10)	0.23 (0.82)	0.09 (0.93)	-1.28 (3.94)	-0.01 (1.82)	0.01 (1.19)	2.07 (3.58)	1.24 (1.31)	0.99 (0.62)	0.97	0.99	1.00	0.97 (0.93-0.99)	0.99 (0.97-1.00)	1.00 (0.99-1.00)
Right superior temporal gyrus	10.39 (1.12)	10.36 (1.15)	10.40 (1.12)	10.40 (1.16)	10.83	11.12	10.78	11.15	0.46 (0.65)	-0.37 (0.72)	-0.31 (0.76)	-0.28 (2.56)	0.17 (1.95)	0.10 (2.01)	1.71 (1.89)	1.64 (0.99)	1.51 (1.29)	0.97	0.98	0.98	0.97 (0.94-0.99)	0.98 (0.96-0.99)	0.98 (0.96-0.99)
Right supramarginal gyrus	9.21 (1.00)	9.11 (1.10)	9.14 (1.05)	9.20 (1.01)	10.90	12.07	11.45	10.99	1.16 (0.26)	1.89 (0.07)	0.27 (0.79)	-1.13 (4.11)	-0.73 (1.63)	-0.14 (1.82)	2.44 (3.45)	1.51 (0.92)	1.41 (1.11)	0.95	0.99	0.98	0.94 (0.86-0.98)	0.99 (0.97-1.00)	0.99 (0.96-0.99)
Right frontal pole	1.04 (0.15)	1.05 (0.11)	1.06 (0.17)	1.03 (0.14)	14.80	10.92	16.08	13.75	-0.23 (0.82)	-0.76 (0.46)	0.74 (0.47)	0.95 (9.21)	1.38 (11.32)	-0.83 (6.64)	7.19 (5.60)	7.15 (8.74)	5.33 (3.86)	0.81	0.80	0.91	0.78 (0.53-0.90)	0.80 (0.58-0.92)	0.90 (0.78-0.96)
Right temporal pole	2.46 (0.23)	2.44 (0.20)	2.44 (0.27)	2.49 (0.24)	9.44	8.01	11.06	9.70	0.71 (0.49)	0.48 (0.64)	-1.18 (0.25)	-0.77 (5.90)	-1.03 (8.25)	1.49 (5.89)	4.45 (3.83)	6.18 (5.39)	4.66 (3.77)	0.79	0.68	0.82	0.78 (0.54-0.91)	0.68 (0.36-0.86)	0.81 (0.60-0.92)
Right transverse temporal gyrus	0.84 (0.15)	0.83 (0.14)	0.84 (0.15)	0.84 (0.15)	17.76	16.40	18.00	17.51	1.81 (0.09)	1.06 (0.30)	0.61 (0.55)	-1.44 (4.47)	-0.80 (3.53)	-0.29 (2.71)	3.72 (2.75)	3.22 (1.50)	2.30 (1.37)	0.97	0.98	0.99	0.97 (0.91-0.99)	0.98 (0.96-0.99)	0.99 (0.98-1.00)
Right insula	6.72 (0.57)	6.62 (0.67)	6.68 (0.71)	6.74 (0.67)	8.48	10.19	10.58	9.90	1.41 (0.17)	0.56 (0.58)	-0.22 (0.83)	-1.69 (4.80)	-0.84 (5.17)	0.12 (4.90)	3.68 (3.44)	3.80 (3.51)	3.78 (3.01)	0.87	0.87	0.86	0.85 (0.67-0.94)	0.85 (0.67-0.94)	0.86 (0.68-0.94)
Local cortical surface (cm^2)																							
Left banks superior temporal sulcus	9.01 (1.18)	8.84 (1.26)	8.87 (1.25)	9.08 (1.28)	13.11	14.20	14.14	14.05	1.66 (0.11)	2.28 (0.03)	-1.02 (0.32)	-2.09 (6.02)	-1.79 (4.04)	0.57 (3.79)	3.63 (5.20)	2.73 (3.45)	2.51 (2.84)	0.93	0.97	0.97	0.92 (0.81-0.97)	0.97 (0.91-0.99)	0.97 (0.93-0.99)
Left caudal anterior cingulate cortex	5.56 (1.09)	5.59 (1.06)	5.48 (1.16)	5.62 (1.08)	19.58	18.87	21.16	19.15	-0.56 (0.58)	1.20 (0.24)	-1.42 (0.17)	0.74 (5.57)	-1.74 (6.13)	1.19 (3.81)	4.02 (3.82)	3.55 (5.25)	3.05 (2.50)	0.97	0.96	0.98	0.97 (0.92-0.99)	0.96 (0.90-0.98)	0.98 (0.96-0.99)
Left caudal middle frontal gyrus	20.53 (3.28)	20.48 (3.37)	20.43 (3.36)	20.56 (3.38)	15.97	16.45	16.44	16.46	0.57 (0.57)	1.05 (0.31)	-0.42 (0.68)	-0.30 (2.09)	-0.56 (2.27)	0.11 (2.07)	1.45 (1.51)	1.53 (1.74)	1.60 (1.28)	0.99	0.99	0.99	0.99 (0.98-1.00)	0.99 (0.98-1.00)	0.99 (0.98-1.00)
Left cuneus	14.33 (2.05)	14.27 (2.22)	14.53 (2.08)	14.37 (2.11)	14.33	15.58	14.32	14.70	0.57 (0.58)	-2.07 (0.05)	-0.72 (0.48)	-0.60 (3.85)	1.40 (3.34)	0.23 (1.96)	2.18 (3.19)	2.58 (2.50)	1.46 (1.29)	0.98	0.98	0.99	0.98 (0.94-0.99)	0.97 (0.93-0.99)	0.99 (0.98-1.00)
Left entorhinal cortex	4.30 (1.13)	4.33 (1.00)	4.28 (1.08)	4.31 (1.10)	26.38	23.06	25.36	25.52	-0.37 (0.71)	0.37 (0.72)	-0.25 (0.81)	1.21 (8.68)	-0.45 (5.70)	0.64 (5.63)	6.66 (5.51)	4.74 (3.02)	4.35 (3.50)	0.95	0.98	0.98	0.95 (0.87-0.98)	0.98 (0.94-0.99)	0.98 (0.96-0.99)
Left fusiform gyrus	29.19 (3.52)	29.12 (3.65)	29.21 (3.53)	29.26 (3.40)	12.07	12.52	12.08	11.63	0.42 (0.68)	-0.13 (0.90)	-0.46 (0.65)	-0.28 (2.59)	0.05 (1.90)	0.29 (2.55)	2.03 (1.58)	1.49 (1.12)	1.87 (1.70)	0.98	0.99	0.98	0.98 (0.95-0.99)	0.99 (0.97-0.99)	0.98 (0.95-0.99)
Left inferior parietal cortex	40.41 (6.19)	39.62 (6.30)	40.10 (6.16)	40.39 (6.25)	15.33	15.89	15.36	15.49	1.61 (0.12)	1.89 (0.07)	0.13 (0.90)	-2.24 (7.10)	-0.79 (1.90)	-0.08 (1.92)	3.12 (6.74)	1.66 (1.17)	1.52 (1.13)	0.94	0.99	0.99	0.93 (0.84-0.97)	0.99 (0.98-1.00)	0.99 (0.98-1.00)
Left inferior temporal gyrus	31.30 (3.86)	31.12 (3.98)	31.21 (4.06)	31.51 (3.83)	12.33	12.78	13.00	12.16	2.13 (0.05)	0.75 (0.46)	-1.67 (0.11)	-0.63 (1.33)	-0.38 (1.82)	0.71 (1.98)	0.94 (1.12)	1.48 (1.09)	1.58 (1.35)	1.00	0.99	0.99	0.99 (0.98-1.00)	0.99 (0.98-1.00)	0.99 (0.97-1.00)
Left isthmus cingulate cortex	9.16 (1.08)	9.13 (1.00)	9.04 (1.08)	9.07 (1.05)	11.78	10.95	11.94	11.58	0.28 (0.78)	2.18 (0.04)	2.07 (0.05)	-0.18 (5.44)	-1.35 (2.83)	-1.03 (2.43)	3.88 (3.72)	2.28 (2.11)	2.05 (1.62)	0.90	0.97	0.98	0.91 (0.78-0.96)	0.97 (0.91-0.99)	0.98 (0.94-0.99)
Left lateral occipital cortex	47.26 (4.63)	46.81 (5.57)	47.12 (4.72)	47.17 (4.86)	9.79	11.89	10.01	10.30	0.93 (0.36)	0.79 (0.44)	0.43 (0.67)	-1.19 (5.53)	-0.31 (1.69)	-0.23 (2.13)	2.31 (5.14)	1.32 (1.05)	1.29 (1.69)	0.92	0.99	0.98	0.91 (0.79-0.96)	0.99 (0.97-0.99)	0.98 (0.95-0.99)
Left lateral orbitofrontal cortex	24.71 (2.68)	24.75 (2.87)	24.68 (2.78)	24.85 (2.88)	10.86	11.61	11.26	11.61	-0.50 (0.62)	0.36 (0.73)	-1.69 (0.11)	0.10 (1.77)	-0.17 (1.53)	0.48 (1.39)	1.24 (1.24)	1.24 (0.87)	1.12 (0.94)	0.99	0.99	0.99	0.99 (0.97-1.00)	0.99 (0.98-1.00)	0.99 (0.98-1.00)
Left lingual gyrus	29.65 (3.75)	29.65 (3.58)	29.77 (3.75)	29.76 (3.58)	12.64	12.09	12.60	12.04	-0.02 (0.99)	-0.85 (0.41)	-0.76 (0.46)	0.06 (1.91)	0.42 (2.15)	0.43 (2.09)	1.54 (1.08)	1.86 (1.08)	1.72 (1.22)	0.99	0.98	0.98	0.99 (0.97-1.00)	0.98 (0.96-0.99)	0.98 (0.96-0.99)
Left medial orbitofrontal cortex	18.66 (2.42)	18.40 (2.55)	18.07 (2.50)	18.79 (2.37)	12.97	13.84	13.85	12.60	0.97 (0.34)	2.37 (0.03)	-0.55 (0.59)	-1.48 (6.75)	-3.21 (6.34)	0.75 (6.01)	5.55 (3.94)	5.49 (4.40)	4.60 (3.80)	0.88	0.90	0.90	0.88 (0.74-0.95)	0.87 (0.68-0.95)	0.90 (0.77-0.96)
Left middle temporal gyrus	28.89 (3.48)	29.07 (3.55)	28.93 (3.54)	29.13 (3.39)	12.03	12.20	12.24	11.65	-1.32 (0.20)	-0.40 (0.69)	-2.20 (0.04)	0.58 (2.24)	0.08 (1.38)	0.88 (1.77)	1.55 (1.69)	0.98 (0.95)	1.50 (1.25)	0.99	0.99	0.99	0.98 (0.96-0.99)	0.99 (0.99-1.00)	0.99 (0.97-1.00)
Left parahippocampal gyrus	6.16 (0.89)	6.21 (0.86)	6.20 (0.88)	6.26 (0.99)	14.44	13.87	14.25	15.87	-1.25 (0.23)	-0.83 (0.42)	-1.52 (0.14)	0.95 (3.46)	0.69 (3.64)	1.40 (4.50)	2.84 (2.11)	2.85 (2.28)	3.75 (2.76)	0.97	0.96	0.96	0.97 (0.93-0.99)	0.97 (0.92-0.99)	0.95 (0.88-0.98)
Left paracentral lobule	13.37 (1.82)	13.33 (1.93)	13.40 (1.99)	13.31 (1.94)	13.60	14.51	14.86	14.59	0.63 (0.54)	-0.33 (0.74)	1.11 (0.28)	-0.38 (1.97)	0.06 (2.25)	-0.59 (1.95)	1.52 (1.28)	1.63 (1.51)	1.63 (1.17)	0.99	0.99	0.99	0.99 (0.97-1.00)	0.98 (0.96-0.99)	0.99 (0.98-1.00)
Left pars opercularis	14.49 (1.98)	14.62 (1.99)	14.49 (1.98)	14.62 (2.00)	13.69	13.62	13.70	13.70	-1.63 (0.12)	0.07 (0.94)	-1.93 (0.07)	0.88 (2.28)	-0.04 (2.55)	0.91 (1.99)	1.95 (1.43)	1.84 (1.73)	1.76 (1.24)	0.98	0.98	0.99	0.98 (0.96-0.99)	0.98 (0.96-0.99)	0.99 (0.96-0.99)
Left pars orbitalis	6.40 (0.87)	6.44 (0.88)	6.44 (0.84)	6.44 (0.78)	13.59	13.72	13.05	12.12	-0.85 (0.41)	-1.05 (0.31)	-0.86 (0.40)	0.59 (3.40)	0.75 (2.99)	0.87 (3.61)	2.76 (1.99)	2.32 (1.97)	3.03 (2.04)	0.97	0.97	0.96	0.97 (0.93-0.99)	0.97 (0.94-0.99)	0.96 (0.90-0.98)
Left pars triangularis	12.16 (1.90)	12.14 (1.83)	12.05 (1.83)	12.14 (1.85)	15.64	15.06	15.16	15.26	0.33 (0.75)	2.21 (0.04)	0.26 (0.80)	-0.06 (2.05)	-0.85 (1.70)	-0.13 (3.13)	1.68 (1.13)	1.50 (1.14)	2.46 (1.85)	0.99	0.99	0.98	0.99 (0.98-1.00)	0.99 (0.97-1.00)	0.98 (0.95-0.99)
Left pericalcarine cortex	13.70 (1.78)	13.77 (1.65)	13.60 (1.89)	13.59 (1.96)	12.99	11.99	13.88	14.39	-0.49 (0.63)	0.98 (0.34)	1.06 (0.30)	0.68 (5.40)	-0.81 (3.65)	-0.97 (3.77)	2.83 (4.61)	2.40 (2.82)	2.62 (2.82)	0.92	0.97	0.97	0.92 (0.81-0.97)	0.97 (0.93-0.99)	0.97 (0.93-0.99)
Left postcentral gyrus	39.44 (4.36)	39.20 (4.44)	39.29 (4.44)	39.42 (4.41)	11.05	11.33	11.30	11.19	2.04 (0.05)	1.50 (0.15)	0.24 (0.81)	-0.62 (1.43)	-0.40 (1.16)	-0.07 (1.18)	1.00 (1.18)	1.05 (0.61)	0.90 (0.74)	0.99	0.99	0.99	0.99 (0.98-1.00)	0.99 (0.99-1.00)	1.00 (0.99-1.00)
Left posterior cingulate cortex	10.43 (1.10)	10.33 (1.13)	10.33 (1.16)	10.63 (1.17)	10.54	10.91	11.21	10.97	0.79 (0.44)	0.90 (0.38)	-2.26 (0.03)	-1.00 (5.67)	-1.04 (5.13)	1.85 (3.72)	3.54 (4.48)	3.00 (4.24)	3.23 (2.55)	0.86	0.89	0.94	0.86 (0.69-0.94)	0.89 (0.76-0.96)	0.92 (0.80-0.97)
Left precentral gyrus	46.97 (5.50)	47.07 (5.78)	47.18 (5.79)	46.98 (5.74)	11.71	12.28	12.27	12.22	-0.38 (0.71)	-0.79 (0.44)	-0.09 (0.93)	0.14 (2.45)	0.38 (2.49)	-0.03 (1.09)	1.12 (2.17)	1.11 (2.25)	0.88 (0.62)	0.98	0.98	1.00	0.98 (0.95-0.99)	0.98 (0.94-0.99)	1.00 (0.99-1.00)
Left precuneus	35.18 (4.46)	34.88 (4.61)	35.13 (4.49)	35.20 (4.45)	12.68	13.22	12.78	12.64	1.21 (0.24)	0.55 (0.59)	-0.21 (0.83)	-0.92 (3.63)	-0.15 (1.22)	0.06 (1.31)	1.91 (3.20)	0.95 (0.76)	1.02 (0.79)	0.97	1.00	1.00	0.97 (0.93-0.99)	1.00 (0.99-1.00)	1.00 (0.99-1.00)

Left rostral anterior cingulate cortex	8.41 (1.79)	8.38 (1.67)	8.48 (1.89)	8.41 (1.76)	21.33	19.98	22.32	20.93	0.30 (0.77)	-0.83 (0.42)	0.00 (1.00)	0.04 (6.27)	0.64 (4.50)	0.05 (4.30)	4.44 (4.31)	3.44 (2.87)	3.62 (2.17)	0.97	0.98	0.98	0.97 (0.92-0.99)	0.98 (0.95-0.99)	0.98 (0.96-0.99)
Left rostral middle frontal gyrus	52.24 (7.08)	51.98 (7.44)	51.97 (6.82)	52.37 (6.85)	13.54	14.32	13.12	13.07	1.11 (0.28)	1.06 (0.30)	-0.57 (0.58)	-0.60 (2.19)	-0.46 (2.29)	0.31 (2.11)	1.75 (1.39)	1.94 (1.23)	1.73 (1.19)	0.99	0.99	0.99	0.99 (0.97-1.00)	0.99 (0.97-0.99)	0.99 (0.97-1.00)
Left superior frontal gyrus	69.93 (9.72)	69.64 (9.72)	70.04 (9.61)	69.97 (9.84)	13.90	13.96	13.73	14.06	1.78 (0.09)	-0.68 (0.51)	-0.20 (0.84)	-0.42 (1.10)	0.18 (1.06)	0.05 (1.33)	0.90 (0.74)	0.83 (0.66)	1.02 (0.83)	1.00	1.00	1.00	1.00 (0.99-1.00)	1.00 (0.99-1.00)	1.00 (0.99-1.00)
Left superior parietal cortex	49.49 (6.20)	49.13 (7.38)	49.30 (6.28)	49.46 (6.12)	12.53	15.01	12.75	12.38	0.84 (0.41)	1.38 (0.18)	0.27 (0.79)	-1.15 (5.63)	-0.40 (1.27)	-0.03 (0.95)	2.11 (5.33)	1.21 (0.51)	0.80 (0.49)	0.97	1.00	1.00	0.96 (0.90-0.98)	0.99 (0.99-1.00)	1.00 (0.99-1.00)
Left superior temporal gyrus	37.51 (4.24)	37.49 (4.37)	37.54 (4.40)	37.53 (4.38)	11.30	11.65	11.73	11.68	0.30 (0.77)	-0.27 (0.79)	-0.20 (0.85)	-0.10 (0.97)	0.03 (1.35)	0.02 (1.37)	0.74 (0.63)	0.95 (0.92)	1.07 (0.82)	1.00	0.99	0.99	1.00 (0.99-1.00)	0.99 (0.99-1.00)	0.99 (0.98-1.00)
Left supramarginal gyrus	37.99 (6.26)	38.00 (7.18)	37.96 (6.43)	38.00 (6.48)	16.48	18.88	16.94	17.06	-0.02 (0.98)	0.23 (0.82)	-0.16 (0.87)	-0.31 (4.85)	-0.13 (1.35)	-0.04 (1.16)	2.48 (4.15)	0.99 (0.89)	0.95 (0.62)	0.97	1.00	1.00	0.96 (0.91-0.98)	1.00 (0.99-1.00)	1.00 (0.99-1.00)
Left frontal pole	2.44 (0.30)	2.44 (0.35)	2.42 (0.30)	2.46 (0.31)	12.41	14.21	12.26	12.45	0.07 (0.94)	0.85 (0.41)	-0.48 (0.63)	-0.37 (7.65)	-1.11 (6.22)	0.56 (5.60)	5.95 (4.64)	4.34 (4.50)	4.47 (3.27)	0.85	0.88	0.90	0.85 (0.66-0.94)	0.89 (0.74-0.95)	0.90 (0.78-0.96)
Left temporal pole	4.42 (0.56)	4.34 (0.52)	4.46 (0.49)	4.41 (0.52)	12.76	12.06	11.01	11.78	1.72 (0.10)	-0.59 (0.56)	0.17 (0.86)	-1.68 (4.49)	0.98 (6.34)	-0.12 (5.42)	3.80 (2.82)	5.10 (3.74)	4.52 (2.83)	0.93	0.87	0.91	0.92 (0.81-0.97)	0.87 (0.70-0.94)	0.91 (0.78-0.96)
Left transverse temporal gyrus	4.18 (0.72)	4.21 (0.75)	4.18 (0.76)	4.19 (0.74)	17.30	17.92	18.08	17.59	-0.76 (0.46)	-0.08 (0.93)	-0.33 (0.74)	0.67 (4.31)	-0.05 (4.78)	0.24 (3.70)	3.29 (2.77)	3.38 (3.30)	2.83 (2.32)	0.97	0.96	0.98	0.97 (0.92-0.99)	0.96 (0.91-0.98)	0.98 (0.95-0.99)
Left insula	23.42 (2.77)	23.63 (2.60)	23.80 (2.82)	23.44 (2.49)	11.82	11.01	11.85	10.61	-0.74 (0.47)	-1.03 (0.31)	-0.06 (0.95)	0.92 (5.21)	1.60 (7.27)	0.19 (7.07)	3.81 (3.59)	5.28 (5.12)	4.84 (5.05)	0.88	0.81	0.79	0.89 (0.74-0.95)	0.81 (0.60-0.92)	0.79 (0.55-0.91)
Right banks superior temporal sulcus	8.26 (0.92)	8.12 (1.02)	8.14 (0.81)	8.27 (0.88)	11.16	12.62	9.90	10.64	1.95 (0.06)	1.92 (0.07)	-0.20 (0.84)	-1.88 (4.48)	-1.33 (3.45)	0.19 (2.88)	3.66 (3.11)	2.48 (2.70)	2.33 (1.61)	0.95	0.95	0.97	0.94 (0.84-0.97)	0.94 (0.85-0.97)	0.97 (0.92-0.99)
Right caudal anterior cingulate cortex	6.94 (1.30)	6.99 (1.43)	6.91 (1.22)	6.86 (1.20)	18.76	20.42	17.73	17.43	-0.69 (0.50)	0.50 (0.63)	1.21 (0.24)	0.42 (4.73)	-0.26 (3.95)	-0.98 (4.24)	3.84 (2.66)	3.09 (2.38)	3.41 (2.60)	0.97	0.98	0.97	0.97 (0.93-0.99)	0.98 (0.94-0.99)	0.97 (0.92-0.99)
Right caudal middle frontal gyrus	20.35 (3.62)	20.15 (3.87)	20.31 (3.61)	20.26 (3.72)	17.81	19.19	17.76	18.36	1.81 (0.08)	0.41 (0.68)	1.00 (0.33)	-1.24 (2.88)	-0.17 (2.29)	-0.53 (2.28)	2.15 (2.25)	1.71 (1.49)	1.75 (1.51)	0.99	0.99	0.99	0.99 (0.97-1.00)	0.99 (0.98-1.00)	0.99 (0.98-1.00)
Right cuneus	15.05 (2.23)	15.25 (2.32)	15.17 (2.23)	15.31 (2.32)	14.85	15.24	14.67	15.17	-1.28 (0.21)	-0.56 (0.58)	-1.30 (0.21)	1.21 (4.73)	0.76 (6.02)	1.61 (6.21)	2.71 (4.03)	3.52 (4.89)	3.47 (5.36)	0.95	0.91	0.92	0.95 (0.89-0.98)	0.91 (0.80-0.96)	0.92 (0.82-0.97)
Right entorhinal cortex	3.49 (0.76)	3.56 (0.73)	3.62 (0.80)	3.60 (0.82)	21.70	20.54	22.13	22.66	-1.28 (0.21)	-1.56 (0.13)	-1.69 (0.11)	2.27 (8.02)	3.78 (10.00)	3.09 (8.92)	6.24 (5.36)	8.46 (6.32)	6.76 (6.45)	0.93	0.87	0.92	0.93 (0.84-0.97)	0.86 (0.69-0.94)	0.91 (0.80-0.96)
Right fusiform gyrus	27.56 (3.16)	27.44 (3.39)	27.43 (3.26)	27.40 (3.04)	11.46	12.37	11.89	11.11	0.98 (0.34)	1.13 (0.27)	1.09 (0.29)	-0.53 (2.10)	-0.52 (1.97)	-0.54 (2.33)	1.39 (1.64)	1.57 (1.26)	1.82 (1.51)	0.99	0.99	0.98	0.99 (0.97-0.99)	0.99 (0.97-0.99)	0.98 (0.94-0.99)
Right inferior parietal cortex	45.90 (6.00)	45.09 (6.80)	45.59 (5.49)	45.80 (6.06)	13.08	15.08	12.05	13.24	1.66 (0.11)	1.60 (0.12)	0.61 (0.55)	-2.13 (6.76)	-0.55 (1.94)	-0.25 (1.86)	2.57 (6.60)	1.48 (1.33)	1.28 (1.34)	0.95	0.99	0.99	0.94 (0.85-0.97)	0.99 (0.97-0.99)	0.99 (0.98-1.00)
Right inferior temporal gyrus	30.46 (4.72)	30.35 (4.69)	30.24 (4.59)	30.46 (4.63)	15.51	15.45	15.17	15.21	0.84 (0.41)	1.45 (0.16)	0.03 (0.98)	-0.37 (2.18)	-0.71 (2.33)	0.03 (2.40)	1.68 (1.40)	1.77 (1.63)	1.80 (1.53)	0.99	0.99	0.99	0.99 (0.98-1.00)	0.99 (0.97-0.99)	0.99 (0.97-1.00)
Right isthmus cingulate cortex	8.09 (1.12)	8.13 (1.07)	8.15 (1.05)	8.15 (1.11)	13.83	13.20	12.85	13.64	-0.55 (0.59)	-0.54 (0.60)	-0.54 (0.59)	0.67 (4.87)	0.92 (6.82)	0.84 (6.76)	3.75 (3.06)	5.38 (4.12)	5.51 (3.82)	0.94	0.89	0.89	0.94 (0.86-0.98)	0.89 (0.76-0.96)	0.89 (0.75-0.95)
Right lateral occipital cortex	46.38 (5.52)	45.80 (6.79)	46.40 (5.68)	46.51 (5.79)	11.89	14.83	12.24	12.45	0.96 (0.35)	-0.12 (0.91)	-0.81 (0.43)	-1.70 (7.82)	0.01 (1.92)	0.22 (1.75)	2.74 (7.51)	1.42 (1.25)	1.36 (1.09)	0.92	0.99	0.99	0.90 (0.78-0.96)	0.99 (0.97-1.00)	0.99 (0.98-1.00)
Right lateral orbitofrontal cortex	24.72 (2.81)	25.53 (3.35)	25.32 (2.88)	24.80 (2.97)	11.38	13.11	11.38	11.98	-2.76 (0.01)	-2.05 (0.05)	-0.34 (0.73)	3.02 (5.03)	2.44 (5.18)	0.29 (4.25)	4.70 (3.42)	4.61 (3.28)	3.31 (2.57)	0.92	0.89	0.93	0.88 (0.66-0.95)	0.87 (0.70-0.95)	0.93 (0.84-0.97)
Right lingual gyrus	30.70 (4.11)	30.77 (4.10)	30.67 (3.80)	30.67 (3.97)	13.38	13.32	12.40	12.94	-0.42 (0.68)	0.19 (0.85)	0.26 (0.80)	0.22 (2.41)	0.00 (2.19)	-0.08 (2.08)	2.00 (1.27)	1.64 (1.39)	1.64 (1.23)	0.98	0.99	0.99	0.98 (0.96-0.99)	0.98 (0.96-0.99)	0.99 (0.97-0.99)
Right medial orbitofrontal cortex	18.68 (2.25)	18.79 (2.09)	18.57 (2.04)	18.70 (2.00)	12.04	11.10	10.97	10.71	-0.59 (0.56)	0.61 (0.55)	-0.12 (0.91)	0.71 (4.76)	-0.44 (4.39)	0.27 (5.13)	3.79 (2.84)	3.52 (2.55)	3.79 (3.36)	0.92	0.94	0.91	0.92 (0.82-0.97)	0.93 (0.85-0.97)	0.91 (0.79-0.96)
Right middle temporal gyrus	32.46 (3.34)	32.22 (3.47)	32.49 (3.26)	32.71 (3.26)	10.28	10.77	10.04	9.96	1.58 (0.13)	-0.21 (0.83)	-1.96 (0.06)	-0.82 (2.56)	0.10 (1.80)	0.80 (1.91)	1.22 (2.39)	1.55 (0.85)	1.42 (1.48)	0.98	0.98	0.98	0.98 (0.94-0.99)	0.98 (0.96-0.99)	0.98 (0.95-0.99)
Right parahippocampal gyrus	5.85 (0.65)	5.87 (0.61)	5.93 (0.65)	5.91 (0.63)	11.03	10.48	10.89	10.75	-0.32 (0.76)	-1.51 (0.15)	-0.86 (0.40)	0.29 (3.51)	1.25 (3.84)	0.99 (5.16)	2.65 (2.25)	3.30 (2.23)	3.94 (3.37)	0.95	0.94	0.89	0.95 (0.88-0.98)	0.94 (0.85-0.97)	0.89 (0.75-0.95)
Right paracentral lobule	14.86 (1.71)	14.79 (1.63)	14.84 (1.77)	14.81 (1.77)	11.48	11.01	11.90	11.97	1.05 (0.31)	0.35 (0.73)	0.60 (0.56)	-0.39 (1.78)	-0.19 (1.87)	-0.35 (2.27)	1.28 (1.27)	1.49 (1.10)	1.80 (1.38)	0.99	0.99	0.98	0.99 (0.97-0.99)	0.99 (0.97-1.00)	0.98 (0.96-0.99)
Right pars opercularis	12.40 (1.76)	12.42 (1.71)	12.43 (1.72)	12.38 (1.67)	14.19	13.78	13.81	13.45	-0.27 (0.79)	-0.46 (0.65)	0.17 (0.87)	0.26 (3.55)	0.36 (3.10)	0.00 (3.39)	2.57 (2.40)	2.22 (2.13)	2.71 (1.95)	0.97	0.98	0.97	0.97 (0.93-0.99)	0.98 (0.95-0.99)	0.97 (0.93-0.99)
Right pars orbitalis	7.51 (0.98)	7.53 (0.97)	7.46 (0.88)	7.46 (0.96)	13.07	12.87	11.74	12.82	-0.32 (0.75)	1.03 (0.31)	0.97 (0.35)	0.20 (2.72)	-0.53 (2.91)	-0.64 (3.03)	2.09 (1.69)	2.34 (1.73)	2.46 (1.80)	0.98	0.98	0.97	0.98 (0.95-0.99)	0.97 (0.93-0.99)	0.97 (0.93-0.99)
Right pars triangularis	13.99 (2.06)	14.05 (2.05)	13.97 (1.97)	13.99 (2.04)	14.75	14.58	14.13	14.60	-0.83 (0.41)	0.27 (0.79)	0.10 (0.92)	0.46 (2.31)	-0.11 (3.38)	-0.04 (2.45)	1.85 (1.39)	2.74 (1.89)	1.66 (1.76)	0.99	0.97	0.99	0.99 (0.97-0.99)	0.98 (0.94-0.99)	0.99 (0.97-1.00)
Right pericalcarine cortex	14.93 (2.38)	14.54 (2.56)	14.57 (2.33)	14.66 (2.38)	15.93	17.63	15.96	16.25	1.49 (0.15)	2.29 (0.03)	1.83 (0.08)	-2.85 (8.86)	-2.36 (4.85)	-1.83 (4.77)	4.77 (7.94)	3.39 (4.17)	3.06 (4.06)	0.89	0.95	0.96	0.88 (0.73-0.95)	0.95 (0.85-0.98)	0.96 (0.89-0.98)
Right postcentral gyrus	37.87 (4.99)	37.79 (4.97)	37.76 (4.94)	37.84 (4.88)	13.18	13.15	13.09	12.89	0.75 (0.46)	1.09 (0.29)	0.31 (0.76)	-0.20 (1.32)	-0.29 (1.31)	-0.05 (1.26)	1.05 (0.79)	0.90 (0.98)	0.92 (0.84)	1.00	1.00	1.00	1.00 (0.99-1.00)	1.00 (0.99-1.00)	1.00 (0.99-1.00)
Right posterior cingulate cortex	10.89 (1.57)	11.03 (1.58)	11.02 (1.45)	10.93 (1.48)	14.40	14.31	13.15	13.54	-0.96 (0.35)	-0.97 (0.34)	-0.30 (0.77)	1.23 (6.57)	1.28 (5.90)	0.38 (5.71)	4.71 (4.64)	3.93 (4.50)	3.79 (4.21)	0.91	0.92	0.93	0.91 (0.80-0.96)	0.92 (0.82-0.97)	0.94 (0.85-0.97)
Right precentral gyrus	46.31 (5.24)	46.22 (5.14)	46.04 (5.18)	46.17 (5.28)	11.31	11.13	11.25	11.43	0.67 (0.51)	1.39 (0.18)	0.53 (0.60)	-0.18 (1.30)	-0.57 (1.91)	-0.30 (2.51)	1.03 (0.79)	1.20 (1.57)	1.50 (2.00)	0.99	0.99	0.98	0.99 (0.98-1.00)	0.98 (0.96-0.99)	0.98 (0.94-0.99)
Right precuneus	36.95 (4.78)	37.16 (5.12)	36.89 (4.78)	37.03 (4.89)	12.93	13.77	12.97	13.20	-1.50 (0.15)	0.51 (0.62)	-0.78 (0.45)	0.48 (1.51)	-0.19 (1.47)	0.18 (1.33)	1.13 (1.08)	1.15 (0.91)	0.93 (0.95)	0.99	0.99	1.00	0.99 (0.98-1.00)	0.99 (0.98-1.00)	1.00 (0.99-1.00)
Right rostral anterior cingulate cortex	5.83 (1.32)	5.95 (1.38)	5.94 (1.41)	5.82 (1.28)	22.56	23.12	23.65	22.02	-1.78 (0.09)	-1.50 (0.15)	0.14 (0.89)	1.81 (5.21)	1.61 (5.82)	-0.11 (5.63)	4.43 (3.16)	4.63 (3.76)	4.41 (3.35)	0.97	0.97	0.97	0.97 (0.93-0.99)	0.97 (0.92-0.99)	0.97 (0.92-0.99)
Right rostral middle frontal gyrus	55.11 (8.36)	55.24 (8.34)	55.08 (8.19)	55.60 (8.03)	15.16	15.09	14.86	14.45	-0.76 (0.46)	0.21 (0.84)	-2.65 (0.02)	0.24 (1.35)	-0.03 (1.24)	0.98 (1.61)	1.15 (0.72)	0.93 (0.79)	1.50 (1.12)	1.00	1.00	1.00	1.00 (0.99-1.00)	1.00 (0.99-1.00)	0.99 (0.98-1.00)
Right superior frontal gyrus	66.87 (9.49)	66.76 (9.79)	66.68 (9.56)	66.63 (9.39)	14.19	14.67	14.34	14.10	0.64 (0.53)	1.14 (0.27)	1.49 (0.15)	-0.22 (1.16)	-0.30 (1.13)	-0.34 (1.06)	0.95 (0.68)	0.91 (0.70)	0.78 (0.78)	1.00	1.00	1.00	1.00 (0.99-1.00)	1.00 (0.99-1.00)	1.00 (0.99-1.00)
Right superior parietal cortex	48.97 (5.53)	48.59 (5.97)	48.84 (5.52)	48.98 (5.34)	11.30	12.29	11.31	10.91	1.60 (0.13)	0.80 (0.44)	-0.05 (0.96)	-0.92 (2.83)	-0.28 (1.62)	0.06 (1.26)	1.40 (2.62)	1.14 (1.16)	0.92 (0.84)	0.98	0.99	0.99	0.98 (0.95-0.99)	0.99 (0.98-1.00)	0.99 (0.99-1.00)
Right superior temporal gyrus	34.62 (3.12)	34.66 (3.14)	34.70 (3.06)	34.64 (2.99)	9.00	9.07	8.82	8.64	-0.27 (0.79)	-0.82 (0.42)	-0.20 (0.85)	0.09 (1.70)	0.25 (1.34)	0.09 (1.44)	1.21 (1.16)	1.12 (0.74)	1.00 (1.01)	0.98	0.99	0.99	0.98 (0.96-0.99)	0.99 (0.98-1.00)	0.99 (0.97-1.00)
Right supramarginal gyrus	35.21 (4.69)	35.09 (4.63)	35.11 (4.71)	35.16 (4.60)	13.31	13.21	13.42	13.08	1.01 (0.33)	1.18 (0.25)	0.52 (0.61)	-0.36 (1.56)	-0.29 (1.08)	-0.14 (1.22)	1.25 (0.97)	0.84 (0.73)	1.02 (0.64)	0.99	1.00	1.00	0.99 (0.98-1.00)	1.00 (0.99-1.00)	1.00 (0.99-1.00)

Right frontal pole	2.96 (0.39)	3.00 (0.35)	3.02 (0.42)	2.96 (0.32)	13.07	11.78	13.93	10.82	-0.71 (0.48)	-1.61 (0.12)	0.01 (0.99)	1.36 (8.39)	1.76 (5.72)	0.24 (6.99)	6.51 (5.27)	4.00 (4.38)	5.47 (4.19)	0.81	0.93	0.87	0.81 (0.59-0.92)	0.92 (0.81-0.97)	0.86 (0.68-0.94)
Right temporal pole	4.42 (0.54)	4.41 (0.61)	4.37 (0.58)	4.47 (0.55)	12.16	13.79	13.25	12.39	0.20 (0.84)	0.87 (0.39)	-0.82 (0.42)	-0.42 (7.33)	-1.37 (6.56)	0.98 (5.73)	5.19 (5.05)	4.97 (4.37)	4.47 (3.58)	0.87	0.87	0.90	0.87 (0.70-0.94)	0.87 (0.72-0.95)	0.90 (0.77-0.96)
Right transverse temporal gyrus	3.24 (0.46)	3.19 (0.44)	3.19 (0.47)	3.21 (0.48)	14.30	13.95	14.88	14.82	1.51 (0.15)	1.43 (0.17)	0.96 (0.35)	-1.58 (5.10)	-1.72 (4.87)	-1.10 (4.81)	3.88 (3.59)	4.11 (3.02)	4.20 (2.44)	0.94	0.94	0.95	0.94 (0.85-0.97)	0.93 (0.84-0.97)	0.95 (0.87-0.98)
Right insula	23.35 (2.22)	22.77 (2.71)	23.02 (2.80)	23.43 (2.79)	9.52	11.92	12.14	11.89	1.56 (0.14)	0.91 (0.37)	-0.20 (0.84)	-2.71 (7.11)	-1.67 (6.83)	0.12 (7.76)	5.34 (5.33)	5.22 (4.58)	6.05 (4.67)	0.78	0.81	0.75	0.75 (0.49-0.89)	0.79 (0.55-0.91)	0.74 (0.46-0.89)
Local cortical thickness (mm)																						
Left banks superior temporal sulcus	2.50 (0.14)	2.49 (0.14)	2.50 (0.15)	2.50 (0.14)	5.59	5.78	5.99	5.51	1.27 (0.22)	0.33 (0.75)	0.52 (0.61)	-0.72 (2.51)	-0.10 (1.12)	-0.19 (1.62)	1.72 (1.93)	0.97 (0.52)	1.20 (1.07)	0.90	0.98	0.95	0.89 (0.76-0.96)	0.98 (0.96-0.99)	0.96 (0.90-0.98)
Left caudal anterior cingulate cortex	2.66 (0.28)	2.62 (0.25)	2.66 (0.26)	2.64 (0.25)	10.62	9.62	9.90	9.46	1.86 (0.08)	-0.21 (0.84)	0.64 (0.53)	-1.23 (3.05)	0.24 (3.56)	-0.39 (3.37)	2.43 (2.16)	2.65 (2.31)	2.69 (1.98)	0.95	0.93	0.94	0.94 (0.86-0.98)	0.94 (0.85-0.97)	0.94 (0.86-0.97)
Left caudal middle frontal gyrus	2.44 (0.12)	2.44 (0.11)	2.45 (0.12)	2.44 (0.12)	4.80	4.65	4.92	4.78	-0.03 (0.98)	-0.17 (0.87)	0.58 (0.57)	0.02 (1.74)	0.06 (1.71)	-0.19 (1.52)	1.38 (1.02)	1.40 (0.93)	1.05 (1.08)	0.93	0.94	0.95	0.93 (0.84-0.97)	0.94 (0.86-0.98)	0.95 (0.89-0.98)
Left cuneus	1.80 (0.10)	1.83 (0.17)	1.80 (0.10)	1.81 (0.12)	5.38	9.05	5.42	6.45	-1.16 (0.26)	-0.46 (0.65)	-1.23 (0.23)	1.82 (7.08)	0.29 (2.83)	1.00 (3.79)	2.66 (6.80)	1.52 (2.38)	1.80 (3.47)	0.47	0.86	0.79	0.41 (-0.01-0.71)	0.86 (0.69-0.94)	0.77 (0.53-0.90)
Left entorhinal cortex	3.43 (0.27)	3.41 (0.27)	3.41 (0.27)	3.44 (0.27)	7.99	7.93	7.85	7.82	1.09 (0.29)	0.75 (0.46)	-0.16 (0.87)	-0.65 (2.85)	-0.60 (3.79)	0.11 (2.67)	2.22 (1.84)	3.09 (2.17)	2.15 (1.51)	0.94	0.89	0.95	0.94 (0.86-0.97)	0.89 (0.76-0.96)	0.95 (0.88-0.98)
Left fusiform gyrus	2.69 (0.16)	2.68 (0.16)	2.67 (0.14)	2.67 (0.16)	5.78	5.99	5.22	6.03	0.85 (0.40)	1.73 (0.10)	1.68 (0.11)	-0.29 (1.56)	-0.50 (1.42)	-0.45 (1.19)	1.28 (0.89)	1.15 (0.95)	1.01 (0.75)	0.97	0.97	0.98	0.97 (0.92-0.99)	0.96 (0.91-0.99)	0.98 (0.94-0.99)
Left inferior parietal cortex	2.32 (0.14)	2.32 (0.14)	2.32 (0.14)	2.33 (0.13)	5.86	6.07	5.92	5.71	0.75 (0.46)	-0.54 (0.60)	-0.74 (0.47)	-0.26 (1.51)	0.11 (0.98)	0.23 (1.40)	1.00 (1.14)	0.84 (0.48)	1.05 (0.93)	0.97	0.99	0.97	0.97 (0.92-0.99)	0.99 (0.97-0.99)	0.97 (0.93-0.99)
Left inferior temporal gyrus	2.75 (0.13)	2.74 (0.14)	2.76 (0.12)	2.74 (0.12)	4.57	4.96	4.20	4.43	0.70 (0.49)	-0.46 (0.65)	1.69 (0.11)	-0.27 (1.73)	0.15 (1.35)	-0.43 (1.16)	1.15 (1.30)	1.04 (0.85)	0.95 (0.77)	0.94	0.95	0.97	0.94 (0.86-0.97)	0.95 (0.89-0.98)	0.96 (0.91-0.99)
Left isthmus cingulate cortex	2.34 (0.11)	2.35 (0.12)	2.35 (0.10)	2.38 (0.12)	4.70	5.13	4.43	5.13	-0.40 (0.69)	-0.17 (0.87)	-1.94 (0.07)	0.24 (2.96)	0.12 (3.07)	1.47 (3.51)	2.16 (1.98)	2.54 (1.64)	2.77 (2.56)	0.82	0.77	0.75	0.82 (0.62-0.93)	0.78 (0.52-0.90)	0.72 (0.42-0.88)
Left lateral occipital cortex	2.09 (0.12)	2.11 (0.12)	2.09 (0.11)	2.10 (0.11)	5.57	5.58	5.38	5.36	-1.24 (0.23)	-0.12 (0.91)	-1.44 (0.17)	0.93 (3.38)	0.06 (2.02)	0.59 (1.86)	1.90 (2.93)	1.50 (1.32)	1.21 (1.52)	0.81	0.93	0.94	0.80 (0.58-0.91)	0.93 (0.84-0.97)	0.94 (0.86-0.98)
Left lateral orbitofrontal cortex	2.63 (0.15)	2.62 (0.14)	2.62 (0.14)	2.62 (0.14)	5.60	5.38	5.38	5.19	0.52 (0.61)	0.38 (0.71)	0.53 (0.60)	-0.19 (1.81)	-0.08 (1.18)	-0.19 (1.82)	1.31 (1.24)	0.80 (0.85)	1.31 (1.24)	0.95	0.98	0.94	0.95 (0.87-0.98)	0.98 (0.95-0.99)	0.94 (0.87-0.98)
Left lingual gyrus	1.95 (0.09)	1.95 (0.08)	1.94 (0.09)	1.95 (0.10)	4.79	4.11	4.48	4.98	-0.61 (0.55)	0.27 (0.79)	-0.51 (0.62)	0.29 (1.99)	-0.10 (1.97)	0.19 (1.80)	1.52 (1.27)	1.62 (1.06)	1.19 (1.33)	0.91	0.91	0.93	0.90 (0.78-0.96)	0.91 (0.80-0.96)	0.94 (0.85-0.97)
Left medial orbitofrontal cortex	2.28 (0.16)	2.29 (0.17)	2.29 (0.17)	2.29 (0.17)	7.01	7.53	7.54	7.32	-0.66 (0.52)	-1.11 (0.28)	-0.94 (0.36)	0.29 (2.22)	0.68 (2.84)	0.37 (1.87)	1.69 (1.42)	2.31 (1.72)	1.54 (1.07)	0.95	0.92	0.97	0.95 (0.89-0.98)	0.92 (0.81-0.97)	0.96 (0.92-0.99)
Left middle temporal gyrus	2.77 (0.08)	2.76 (0.08)	2.76 (0.08)	2.76 (0.08)	2.80	2.95	2.75	2.93	0.95 (0.35)	0.83 (0.42)	1.53 (0.14)	-0.44 (2.11)	-0.28 (1.57)	-0.52 (1.54)	1.46 (1.55)	1.23 (0.98)	1.29 (0.96)	0.73	0.84	0.86	0.73 (0.46-0.88)	0.84 (0.66-0.93)	0.85 (0.66-0.93)
Left parahippocampal gyrus	2.83 (0.32)	2.79 (0.31)	2.81 (0.31)	2.81 (0.32)	11.47	11.06	10.97	11.35	2.55 (0.02)	1.31 (0.21)	1.43 (0.17)	-1.38 (2.56)	-0.47 (1.83)	-0.67 (2.22)	2.20 (1.86)	1.58 (0.98)	1.89 (1.29)	0.98	0.99	0.98	0.97 (0.90-0.99)	0.99 (0.97-0.99)	0.98 (0.95-0.99)
Left paracentral lobule	2.32 (0.14)	2.31 (0.13)	2.32 (0.14)	2.31 (0.13)	5.85	5.76	6.08	5.72	0.50 (0.62)	-0.07 (0.95)	0.47 (0.64)	-0.27 (2.41)	0.01 (1.97)	-0.19 (1.87)	1.94 (1.38)	1.37 (1.38)	1.45 (1.16)	0.91	0.95	0.95	0.91 (0.80-0.96)	0.95 (0.89-0.98)	0.95 (0.88-0.98)
Left pars opercularis	2.50 (0.13)	2.48 (0.11)	2.50 (0.12)	2.49 (0.12)	5.29	4.62	4.66	4.90	1.06 (0.30)	0.48 (0.64)	0.85 (0.41)	-0.58 (2.54)	-0.14 (1.63)	-0.30 (1.69)	1.38 (2.19)	1.20 (1.08)	1.25 (1.15)	0.87	0.95	0.95	0.86 (0.68-0.94)	0.95 (0.87-0.98)	0.94 (0.87-0.98)
Left pars orbitalis	2.68 (0.17)	2.68 (0.18)	2.68 (0.19)	2.68 (0.19)	6.48	6.90	6.92	7.16	-0.07 (0.95)	-0.20 (0.84)	-0.26 (0.80)	0.03 (3.04)	0.06 (1.83)	0.10 (2.54)	2.34 (1.87)	1.48 (1.03)	1.91 (1.62)	0.89	0.96	0.93	0.90 (0.76-0.96)	0.96 (0.91-0.98)	0.93 (0.84-0.97)
Left pars triangularis	2.37 (0.16)	2.36 (0.16)	2.37 (0.14)	2.38 (0.16)	6.87	6.76	6.05	6.52	0.63 (0.54)	0.12 (0.91)	-1.01 (0.33)	-0.37 (2.71)	0.00 (2.10)	0.35 (1.51)	2.06 (1.73)	1.64 (1.25)	1.21 (0.94)	0.92	0.95	0.98	0.92 (0.82-0.97)	0.95 (0.88-0.98)	0.97 (0.94-0.99)
Left pericalcarine cortex	1.56 (0.10)	1.60 (0.17)	1.56 (0.09)	1.58 (0.10)	6.73	10.81	5.72	6.52	-1.12 (0.28)	-0.20 (0.84)	-1.91 (0.07)	2.68 (10.62)	0.21 (3.49)	1.41 (3.42)	4.19 (10.09)	2.43 (2.46)	2.43 (2.76)	0.10	0.86	0.87	0.09 (-0.34-0.49)	0.86 (0.68-0.94)	0.86 (0.68-0.94)
Left postcentral gyrus	1.96 (0.11)	1.96 (0.12)	1.96 (0.12)	1.96 (0.12)	5.86	6.21	6.07	6.18	-0.30 (0.77)	-0.02 (0.99)	-0.96 (0.35)	0.11 (1.93)	-0.01 (1.20)	0.19 (1.01)	1.39 (1.31)	0.83 (0.85)	0.85 (0.53)	0.95	0.98	0.99	0.95 (0.89-0.98)	0.98 (0.95-0.99)	0.99 (0.97-0.99)
Left posterior cingulate cortex	2.46 (0.14)	2.44 (0.16)	2.45 (0.13)	2.44 (0.15)	5.55	6.64	5.37	5.99	1.04 (0.31)	0.99 (0.34)	1.24 (0.23)	-0.75 (3.07)	-0.58 (2.71)	-0.75 (2.68)	2.68 (1.58)	2.16 (1.67)	2.09 (1.79)	0.89	0.88	0.89	0.88 (0.73-0.95)	0.88 (0.72-0.95)	0.89 (0.75-0.95)
Left precentral gyrus	2.48 (0.14)	2.48 (0.15)	2.48 (0.15)	2.49 (0.15)	5.79	6.23	5.92	5.89	0.56 (0.58)	0.37 (0.72)	-0.28 (0.78)	-0.28 (2.27)	-0.11 (1.35)	0.09 (1.57)	1.30 (1.86)	0.98 (0.91)	1.20 (0.98)	0.94	0.97	0.97	0.94 (0.86-0.97)	0.98 (0.94-0.99)	0.97 (0.92-0.99)
Left precuneus	2.28 (0.15)	2.28 (0.15)	2.28 (0.16)	2.28 (0.15)	6.58	6.73	7.04	6.77	0.62 (0.54)	0.81 (0.43)	0.75 (0.46)	-0.20 (1.47)	-0.29 (1.43)	-0.23 (1.37)	1.20 (0.82)	1.16 (0.84)	1.09 (0.83)	0.98	0.98	0.98	0.98 (0.94-0.99)	0.98 (0.95-0.99)	0.98 (0.95-0.99)
Left rostral anterior cingulate cortex	2.62 (0.21)	2.61 (0.19)	2.58 (0.17)	2.60 (0.17)	7.87	7.39	6.42	6.68	0.90 (0.38)	2.24 (0.04)	1.22 (0.24)	-0.54 (2.90)	-1.38 (2.85)	-0.91 (3.58)	2.23 (1.86)	2.40 (2.02)	3.12 (1.86)	0.93	0.93	0.88	0.93 (0.83-0.97)	0.90 (0.74-0.96)	0.87 (0.71-0.94)
Left rostral middle frontal gyrus	2.25 (0.12)	2.25 (0.11)	2.26 (0.11)	2.25 (0.12)	5.41	5.06	4.91	5.43	0.67 (0.51)	-0.44 (0.66)	0.61 (0.55)	-0.31 (2.20)	0.17 (1.48)	-0.24 (1.77)	1.39 (1.71)	1.17 (0.88)	1.44 (1.00)	0.91	0.96	0.95	0.91 (0.79-0.96)	0.96 (0.91-0.98)	0.95 (0.88-0.98)
Left superior frontal gyrus	2.52 (0.11)	2.51 (0.11)	2.51 (0.11)	2.52 (0.11)	4.37	4.29	4.46	4.37	0.63 (0.54)	0.65 (0.52)	-0.15 (0.89)	-0.29 (2.15)	-0.23 (1.63)	0.04 (1.12)	1.28 (1.73)	1.37 (0.86)	0.91 (0.62)	0.88	0.93	0.97	0.88 (0.73-0.95)	0.94 (0.85-0.97)	0.97 (0.92-0.99)
Left superior parietal cortex	2.11 (0.15)	2.11 (0.15)	2.11 (0.15)	2.12 (0.14)	7.19	7.22	6.98	6.72	-0.02 (0.98)	-1.71 (0.10)	-2.30 (0.03)	0.01 (1.31)	0.25 (0.64)	0.53 (1.00)	0.93 (0.90)	0.53 (0.42)	0.96 (0.58)	0.98	1.00	0.99	0.98 (0.96-0.99)	1.00 (0.99-1.00)	0.99 (0.96-1.00)
Left superior temporal gyrus	2.62 (0.16)	2.62 (0.15)	2.62 (0.15)	2.62 (0.16)	5.93	5.78	5.74	5.95	0.12 (0.91)	1.03 (0.32)	0.28 (0.78)	-0.02 (1.23)	-0.21 (0.99)	-0.05 (0.89)	0.72 (0.98)	0.76 (0.65)	0.73 (0.49)	0.98	0.99	0.99	0.98 (0.95-0.99)	0.99 (0.97-0.99)	0.99 (0.97-1.00)
Left supramarginal gyrus	2.43 (0.12)	2.42 (0.14)	2.43 (0.12)	2.43 (0.12)	5.10	5.78	5.00	4.96	0.61 (0.55)	-0.57 (0.57)	-0.42 (0.68)	-0.33 (2.28)	0.21 (1.64)	0.15 (1.56)	1.36 (1.84)	1.19 (1.12)	1.21 (0.97)	0.93	0.95	0.95	0.92 (0.82-0.97)	0.95 (0.88-0.98)	0.96 (0.89-0.98)
Left frontal pole	2.58 (0.19)	2.57 (0.21)	2.59 (0.18)	2.57 (0.21)	7.30	8.21	7.07	8.04	0.42 (0.68)	-0.48 (0.64)	0.51 (0.62)	-0.44 (3.89)	0.46 (4.36)	-0.39 (3.02)	3.00 (2.43)	2.90 (3.23)	2.20 (2.04)	0.87	0.82	0.92	0.87 (0.71-0.95)	0.83 (0.63-0.93)	0.92 (0.82-0.97)
Left temporal pole	3.74 (0.28)	3.70 (0.30)	3.72 (0.27)	3.70 (0.31)	7.44	8.12	7.33	8.38	1.23 (0.23)	0.43 (0.67)	0.93 (0.37)	-1.13 (4.14)	-0.41 (4.57)	-0.97 (4.62)	2.73 (3.26)	3.47 (2.90)	3.10 (3.50)	0.87	0.81	0.85	0.87 (0.70-0.94)	0.82 (0.61-0.92)	0.85 (0.67-0.93)
Left transverse temporal gyrus	2.23 (0.22)	2.21 (0.21)	2.23 (0.20)	2.23 (0.21)	9.88	9.31	8.82	9.21	1.71 (0.10)	0.51 (0.62)	0.66 (0.52)	-0.81 (2.35)	-0.17 (2.32)	-0.19 (1.67)	2.03 (1.38)	1.58 (1.66)	1.31 (1.01)	0.97	0.97	0.99	0.97 (0.92-0.99)	0.97 (0.92-0.99)	0.98 (0.96-0.99)
Left insula	2.85 (0.16)	2.83 (0.19)	2.82 (0.20)	2.84 (0.20)	5.78	6.61	7.05	7.11	0.78 (0.45)	0.90 (0.38)	0.32 (0.75)	-0.60 (3.21)	-0.91 (4.13)	-0.33 (3.68)	2.41 (2.15)	3.00 (2.91)	2.58 (2.59)	0.87	0.80	0.86	0.86 (0.70-0.94)	0.79 (0.55-0.91)	0.84 (0.66-0.93)
Right banks superior temporal sulcus	2.50 (0.16)	2.48 (0.18)	2.49 (0.17)	2.48 (0.18)	6.51	7.09	6.76	7.07	1.62 (0.12)	0.25 (0.81)	1.53 (0.14)	-0.63 (1.73)	-0.11 (1.72)	-0.49 (1.37)	1.29 (1.30)	1.20 (1.21)	1.12 (0.90)	0.97	0.97	0.98	0.97 (0.92-0.99)	0.97 (0.93-0.99)	0.98 (0.95-0.99)

Right caudal anterior cingulate cortex	2.48 (0.24)	2.47 (0.25)	2.47 (0.23)	2.47 (0.23)	9.54	10.04	9.30	9.29	1.66 (0.11)	0.71 (0.49)	1.54 (0.14)	-0.78 (1.94)	-0.45 (2.92)	-0.71 (2.12)	1.68 (1.21)	2.33 (1.75)	1.72 (1.38)	0.98	0.95	0.97	0.98 (0.94-0.99)	0.95 (0.88-0.98)	0.97 (0.93-0.99)
Right caudal middle frontal gyrus	2.43 (0.11)	2.42 (0.12)	2.42 (0.12)	2.43 (0.12)	4.69	5.15	4.95	5.02	0.87 (0.39)	0.97 (0.35)	0.61 (0.55)	-0.42 (2.08)	-0.43 (2.00)	-0.28 (1.94)	1.41 (1.55)	1.50 (1.36)	1.53 (1.18)	0.91	0.91	0.92	0.91 (0.79-0.96)	0.91 (0.80-0.96)	0.92 (0.82-0.97)
Right cuneus	1.82 (0.15)	1.84 (0.17)	1.83 (0.14)	1.83 (0.14)	8.21	9.39	7.88	7.75	-1.07 (0.30)	-1.00 (0.33)	-1.90 (0.07)	1.14 (4.62)	0.47 (1.96)	0.77 (1.93)	2.01 (4.30)	1.36 (1.46)	1.32 (1.58)	0.83	0.97	0.98	0.82 (0.62-0.92)	0.97 (0.92-0.99)	0.97 (0.93-0.99)
Right entorhinal cortex	3.56 (0.30)	3.53 (0.28)	3.48 (0.34)	3.52 (0.33)	8.48	7.93	9.71	9.34	1.30 (0.21)	1.91 (0.07)	0.99 (0.34)	-0.68 (2.62)	-2.27 (5.33)	-1.10 (5.01)	2.22 (1.46)	3.88 (4.25)	3.03 (4.08)	0.95	0.84	0.86	0.95 (0.88-0.98)	0.82 (0.60-0.92)	0.86 (0.69-0.94)
Right fusiform gyrus	2.68 (0.14)	2.66 (0.15)	2.68 (0.14)	2.68 (0.13)	5.21	5.46	5.24	4.95	1.71 (0.10)	0.16 (0.87)	-0.61 (0.55)	-0.47 (1.23)	-0.05 (1.34)	0.17 (1.18)	1.06 (0.75)	1.11 (0.71)	0.97 (0.66)	0.97	0.97	0.97	0.97 (0.93-0.99)	0.97 (0.92-0.99)	0.97 (0.94-0.99)
Right inferior parietal cortex	2.32 (0.14)	2.30 (0.14)	2.30 (0.13)	2.31 (0.15)	6.23	6.19	5.73	6.33	1.69 (0.11)	1.91 (0.07)	0.75 (0.46)	-0.67 (1.85)	-0.63 (1.52)	-0.25 (1.43)	1.41 (1.34)	1.07 (1.24)	1.12 (0.88)	0.96	0.97	0.97	0.95 (0.89-0.98)	0.96 (0.90-0.98)	0.97 (0.94-0.99)
Right inferior temporal gyrus	2.77 (0.12)	2.75 (0.13)	2.77 (0.12)	2.77 (0.12)	4.39	4.57	4.42	4.46	1.46 (0.16)	0.04 (0.97)	0.74 (0.47)	-0.69 (2.23)	-0.02 (1.63)	-0.28 (1.69)	1.36 (1.88)	1.21 (1.06)	1.10 (1.29)	0.88	0.93	0.93	0.88 (0.73-0.95)	0.93 (0.85-0.97)	0.93 (0.83-0.97)
Right isthmus cingulate cortex	2.37 (0.24)	2.38 (0.23)	2.34 (0.25)	2.35 (0.26)	10.17	9.69	10.70	11.17	-0.53 (0.60)	1.79 (0.09)	0.89 (0.38)	0.36 (2.67)	-1.13 (3.04)	-0.81 (3.75)	2.12 (1.59)	1.98 (2.55)	2.96 (2.35)	0.97	0.97	0.94	0.97 (0.92-0.99)	0.96 (0.91-0.98)	0.94 (0.86-0.98)
Right lateral occipital cortex	2.16 (0.12)	2.16 (0.11)	2.15 (0.12)	2.17 (0.12)	5.50	5.28	5.72	5.36	-0.29 (0.78)	1.22 (0.24)	-1.15 (0.26)	0.08 (1.12)	-0.53 (2.06)	0.24 (0.94)	0.82 (0.75)	1.23 (1.72)	0.76 (0.58)	0.98	0.94	0.99	0.98 (0.95-0.99)	0.94 (0.85-0.97)	0.98 (0.96-0.99)
Right lateral orbitofrontal cortex	2.58 (0.15)	2.54 (0.15)	2.53 (0.14)	2.56 (0.14)	5.71	5.86	5.37	5.56	4.03 (0.00)	3.53 (0.00)	1.91 (0.07)	-1.91 (2.22)	-1.93 (2.51)	-0.91 (2.17)	2.55 (1.40)	2.39 (2.04)	1.88 (1.37)	0.93	0.90	0.92	0.89 (0.49-0.96)	0.85 (0.48-0.95)	0.91 (0.79-0.97)
Right lingual gyrus	2.01 (0.12)	2.01 (0.11)	2.01 (0.11)	2.01 (0.12)	6.18	5.54	5.57	6.07	0.07 (0.95)	0.23 (0.82)	-0.32 (0.75)	0.00 (2.53)	-0.05 (1.79)	0.11 (1.42)	1.64 (1.89)	1.36 (1.13)	1.10 (0.88)	0.92	0.96	0.97	0.92 (0.80-0.96)	0.96 (0.90-0.98)	0.98 (0.94-0.99)
Right medial orbitofrontal cortex	2.35 (0.14)	2.34 (0.15)	2.34 (0.15)	2.34 (0.15)	6.06	6.51	6.46	6.33	1.39 (0.18)	1.41 (0.17)	0.79 (0.44)	-0.76 (2.33)	-0.72 (2.20)	-0.49 (2.80)	1.88 (1.52)	1.69 (1.53)	2.23 (1.69)	0.93	0.94	0.90	0.92 (0.82-0.97)	0.93 (0.84-0.97)	0.90 (0.78-0.96)
Right middle temporal gyrus	2.79 (0.13)	2.79 (0.15)	2.79 (0.14)	2.79 (0.13)	4.55	5.37	5.06	4.73	0.00 (1.00)	0.46 (0.65)	0.16 (0.88)	-0.04 (1.75)	-0.15 (1.28)	-0.06 (1.41)	1.09 (1.35)	0.89 (0.91)	1.14 (0.80)	0.96	0.97	0.96	0.95 (0.88-0.98)	0.97 (0.92-0.99)	0.96 (0.90-0.98)
Right parahippocampal gyrus	2.73 (0.24)	2.72 (0.23)	2.71 (0.23)	2.71 (0.24)	8.74	8.56	8.33	8.70	0.77 (0.45)	1.47 (0.16)	1.58 (0.13)	-0.41 (2.58)	-0.77 (2.42)	-0.80 (2.29)	2.03 (1.57)	1.98 (1.54)	1.83 (1.55)	0.96	0.96	0.96	0.96 (0.90-0.98)	0.95 (0.89-0.98)	0.96 (0.91-0.98)
Right paracentral lobule	2.31 (0.14)	2.31 (0.15)	2.32 (0.15)	2.32 (0.15)	5.92	6.33	6.45	6.27	0.01 (1.00)	-0.69 (0.50)	-0.90 (0.38)	-0.03 (1.74)	0.25 (1.87)	0.25 (1.30)	1.35 (1.05)	1.51 (1.09)	1.03 (0.81)	0.96	0.96	0.98	0.96 (0.91-0.98)	0.95 (0.89-0.98)	0.98 (0.94-0.99)
Right pars opercularis	2.53 (0.14)	2.52 (0.15)	2.53 (0.14)	2.53 (0.15)	5.69	6.04	5.62	6.06	0.97 (0.34)	-0.35 (0.73)	-0.52 (0.61)	-0.42 (1.91)	0.08 (1.02)	0.13 (1.37)	1.27 (1.46)	0.81 (0.60)	1.09 (0.81)	0.95	0.98	0.98	0.95 (0.88-0.98)	0.98 (0.96-0.99)	0.97 (0.94-0.99)
Right pars orbitalis	2.65 (0.16)	2.65 (0.15)	2.65 (0.12)	2.68 (0.13)	6.07	5.82	4.70	4.92	0.40 (0.70)	0.21 (0.83)	-2.13 (0.05)	-0.20 (2.58)	-0.03 (2.32)	1.08 (2.28)	2.03 (1.54)	1.80 (1.40)	1.77 (1.77)	0.91	0.94	0.94	0.91 (0.80-0.96)	0.92 (0.80-0.96)	0.91 (0.77-0.96)
Right pars triangularis	2.43 (0.14)	2.41 (0.15)	2.41 (0.13)	2.42 (0.15)	5.74	6.15	5.46	6.05	1.33 (0.20)	2.20 (0.04)	0.49 (0.63)	-0.89 (3.03)	-0.62 (1.33)	-0.22 (1.91)	2.04 (2.37)	1.15 (0.88)	1.49 (1.17)	0.88	0.97	0.95	0.87 (0.71-0.95)	0.97 (0.91-0.99)	0.95 (0.88-0.98)
Right pericalcarine cortex	1.63 (0.14)	1.63 (0.13)	1.63 (0.13)	1.63 (0.13)	8.64	7.87	8.29	7.78	0.42 (0.68)	0.32 (0.75)	-0.53 (0.60)	-0.18 (2.65)	-0.12 (2.47)	0.36 (2.56)	1.85 (1.86)	1.60 (1.85)	1.66 (1.94)	0.96	0.97	0.96	0.95 (0.89-0.98)	0.97 (0.92-0.99)	0.96 (0.90-0.98)
Right postcentral gyrus	2.00 (0.11)	2.00 (0.12)	2.00 (0.12)	1.99 (0.11)	5.72	6.19	5.80	5.72	0.32 (0.75)	-0.28 (0.78)	0.87 (0.39)	-0.16 (2.04)	0.06 (1.04)	-0.18 (0.96)	1.11 (1.71)	0.84 (0.59)	0.81 (0.53)	0.95	0.98	0.99	0.95 (0.88-0.98)	0.98 (0.96-0.99)	0.99 (0.97-0.99)
Right posterior cingulate cortex	2.32 (0.16)	2.32 (0.14)	2.33 (0.14)	2.32 (0.15)	6.88	6.09	5.86	6.51	0.47 (0.64)	-0.36 (0.72)	0.01 (0.99)	-0.23 (2.72)	0.29 (2.91)	0.01 (3.59)	1.87 (1.95)	2.07 (2.02)	2.25 (2.75)	0.92	0.91	0.86	0.92 (0.81-0.97)	0.90 (0.77-0.96)	0.87 (0.70-0.94)
Right precentral gyrus	2.46 (0.13)	2.47 (0.15)	2.46 (0.14)	2.46 (0.14)	5.31	5.95	5.51	5.57	-1.46 (0.16)	-0.99 (0.33)	-0.90 (0.38)	0.53 (1.77)	0.28 (1.32)	0.27 (1.45)	1.35 (1.23)	1.18 (0.61)	0.99 (1.07)	0.96	0.97	0.97	0.95 (0.88-0.98)	0.97 (0.93-0.99)	0.96 (0.92-0.99)
Right precuneus	2.30 (0.17)	2.28 (0.16)	2.29 (0.17)	2.28 (0.17)	7.44	7.02	7.32	7.42	1.88 (0.07)	0.69 (0.50)	1.44 (0.16)	-0.68 (1.70)	-0.21 (1.37)	-0.48 (1.49)	1.14 (1.41)	0.95 (1.00)	0.99 (1.20)	0.97	0.98	0.98	0.97 (0.92-0.99)	0.98 (0.96-0.99)	0.98 (0.95-0.99)
Right rostral anterior cingulate cortex	2.87 (0.18)	2.83 (0.18)	2.82 (0.21)	2.87 (0.19)	6.43	6.47	7.31	6.70	2.33 (0.03)	2.41 (0.03)	0.17 (0.86)	-1.52 (3.10)	-1.74 (3.16)	-0.13 (3.10)	2.62 (2.20)	2.72 (2.31)	2.63 (1.55)	0.89	0.90	0.89	0.87 (0.68-0.95)	0.87 (0.67-0.95)	0.89 (0.76-0.96)
Right rostral middle frontal gyrus	2.27 (0.13)	2.26 (0.13)	2.27 (0.13)	2.27 (0.14)	5.86	5.75	5.57	6.05	0.97 (0.34)	-0.62 (0.54)	0.32 (0.75)	-0.42 (1.93)	0.20 (1.35)	-0.11 (1.50)	1.17 (1.57)	1.04 (0.85)	1.19 (0.88)	0.94	0.97	0.97	0.94 (0.86-0.98)	0.97 (0.94-0.99)	0.97 (0.93-0.99)
Right superior frontal gyrus	2.54 (0.13)	2.53 (0.14)	2.54 (0.14)	2.54 (0.14)	5.24	5.71	5.67	5.46	1.11 (0.28)	0.89 (0.38)	1.06 (0.30)	-0.52 (2.12)	-0.33 (1.59)	-0.27 (1.15)	1.42 (1.63)	1.13 (1.14)	0.94 (0.68)	0.93	0.96	0.98	0.93 (0.84-0.97)	0.96 (0.90-0.98)	0.98 (0.95-0.99)
Right superior parietal cortex	2.09 (0.16)	2.08 (0.15)	2.09 (0.16)	2.09 (0.16)	7.75	7.41	7.53	7.70	1.73 (0.10)	0.25 (0.81)	0.67 (0.51)	-0.65 (1.90)	-0.05 (1.39)	-0.13 (0.98)	1.23 (1.57)	1.01 (0.92)	0.82 (0.52)	0.97	0.99	0.99	0.97 (0.93-0.99)	0.99 (0.96-0.99)	0.99 (0.98-1.00)
Right superior temporal gyrus	2.66 (0.19)	2.64 (0.20)	2.65 (0.19)	2.66 (0.20)	7.21	7.55	7.17	7.40	1.18 (0.25)	0.68 (0.50)	-0.31 (0.76)	-0.64 (2.43)	-0.18 (1.17)	0.06 (1.08)	1.39 (2.08)	0.75 (0.90)	0.88 (0.60)	0.95	0.99	0.99	0.95 (0.87-0.98)	0.99 (0.97-0.99)	0.99 (0.97-1.00)
Right supramarginal gyrus	2.39 (0.13)	2.38 (0.15)	2.38 (0.12)	2.39 (0.14)	5.45	6.19	5.07	5.75	1.73 (0.10)	2.28 (0.03)	0.64 (0.53)	-0.73 (1.99)	-0.46 (0.95)	-0.17 (1.10)	1.18 (1.75)	0.84 (0.62)	0.82 (0.74)	0.96	0.99	0.98	0.95 (0.87-0.98)	0.98 (0.94-0.99)	0.98 (0.96-0.99)
Right frontal pole	2.61 (0.17)	2.59 (0.15)	2.58 (0.22)	2.59 (0.19)	6.48	5.78	8.63	7.25	0.81 (0.43)	0.80 (0.43)	0.53 (0.60)	-0.67 (3.93)	-1.31 (6.93)	-0.46 (3.50)	2.47 (3.09)	4.16 (5.62)	2.98 (1.78)	0.79	0.65	0.87	0.79 (0.55-0.91)	0.63 (0.28-0.83)	0.87 (0.71-0.95)
Right temporal pole	3.75 (0.29)	3.73 (0.33)	3.75 (0.32)	3.74 (0.32)	7.81	8.79	8.57	8.44	0.57 (0.58)	0.04 (0.97)	0.23 (0.82)	-0.60 (4.37)	-0.09 (3.49)	-0.25 (3.93)	2.97 (3.19)	2.35 (2.53)	2.59 (2.91)	0.89	0.91	0.89	0.89 (0.74-0.95)	0.91 (0.80-0.96)	0.89 (0.75-0.95)
Right transverse temporal gyrus	2.29 (0.23)	2.29 (0.23)	2.31 (0.24)	2.32 (0.24)	10.10	10.03	10.38	10.14	0.28 (0.78)	-1.03 (0.32)	-2.38 (0.03)	-0.17 (2.67)	0.52 (2.47)	1.05 (1.96)	1.94 (1.80)	2.04 (1.42)	1.70 (1.41)	0.96	0.97	0.98	0.96 (0.91-0.98)	0.97 (0.93-0.99)	0.98 (0.93-0.99)
Right insula	2.85 (0.16)	2.87 (0.16)	2.87 (0.15)	2.86 (0.19)	5.79	5.59	5.38	6.54	-1.18 (0.25)	-1.27 (0.22)	-0.45 (0.65)	0.73 (2.78)	0.61 (2.15)	0.25 (2.93)	2.22 (1.77)	1.71 (1.40)	2.24 (1.83)	0.88	0.93	0.89	0.88 (0.73-0.95)	0.92 (0.83-0.97)	0.89 (0.75-0.95)
Global brain measures																							
Left total cortical volume (ml)	211.74 (16.31)	211.01 (17.60)	211.56 (16.86)	211.98 (16.86)	7.70	8.34	7.97	7.96	1.02 (0.32)	0.62 (0.54)	-1.04 (0.31)	-0.40 (1.70)	-0.11 (0.63)	0.09 (0.49)	0.74 (1.58)	0.41 (0.48)	0.39 (0.31)	0.98	1.00	1.00	0.98 (0.95-0.99)	1.00 (0.99-1.00)	1.00 (1.00-1.00)
Right total cortical volume (ml)	212.77 (16.62)	211.74 (18.22)	212.29 (16.81)	212.86 (16.91)	7.81	8.61	7.92	7.94	1.12 (0.28)	1.35 (0.19)	-0.30 (0.77)	-0.55 (2.22)	-0.23 (0.75)	0.03 (0.64)	0.91 (2.09)	0.50 (0.60)	0.52 (0.37)	0.97	1.00	1.00	0.97 (0.93-0.99)	1.00 (0.99-1.00)	1.00 (0.99-1.00)
Left total cortical white matter (ml)	216.79 (29.78)	215.00 (30.66)	216.25 (30.06)	216.50 (29.79)	13.74	14.26	13.90	13.76	1.38 (0.18)	0.94 (0.36)	0.63 (0.53)	-0.90 (3.03)	-0.28 (1.21)	-0.13 (0.96)	1.42 (2.81)	0.99 (0.72)	0.77 (0.56)	0.98	1.00	1.00	0.98 (0.95-0.99)	1.00 (0.99-1.00)	1.00 (0.99-1.00)
Right total cortical white matter (ml)	215.72 (29.09)	214.30 (29.21)	215.03 (28.61)	215.23 (28.85)	13.49	13.63	13.31	13.41	1.81 (0.08)	1.56 (0.13)	0.92 (0.37)	-0.68 (1.80)	-0.30 (0.93)	-0.22 (1.15)	1.03 (1.61)	0.69 (0.67)	0.87 (0.76)	0.99	1.00	1.00	0.99 (0.98-1.00)	1.00 (0.99-1.00)	1.00 (0.99-1.00)

Left total cortical surface area (cm²)	801.15 (76.01)	798.32 (79.37)	799.72 (76.51)	802.52 (77.01)	9.49	9.94	9.57	9.60	1.28 (0.22)	1.51 (0.15)	-2.01 (0.06)	-0.40 (1.42)	-0.19 (0.55)	0.16 (0.38)	0.68 (1.30)	0.45 (0.35)	0.32 (0.25)	0.99	1.00	1.00	0.99 (0.98-1.00)	1.00 (1.00-1.00)	1.00 (1.00-1.00)
Right total cortical surface area (cm²)	801.02 (73.78)	799.16 (77.17)	799.63 (73.67)	801.42 (73.10)	9.21	9.66	9.21	9.12	0.86 (0.40)	1.34 (0.19)	-0.36 (0.72)	-0.27 (1.36)	-0.18 (0.60)	0.06 (0.64)	0.72 (1.17)	0.48 (0.38)	0.48 (0.41)	0.99	1.00	1.00	0.99 (0.98-1.00)	1.00 (0.99-1.00)	1.00 (0.99-1.00)
Left mean cortical thickness (mm)	2.38 (0.09)	2.38 (0.09)	2.38 (0.09)	2.38 (0.10)	3.97	3.96	3.82	4.01	0.42 (0.68)	0.38 (0.71)	-0.52 (0.61)	-0.07 (0.76)	-0.05 (0.65)	0.06 (0.53)	0.53 (0.53)	0.42 (0.49)	0.40 (0.35)	0.98	0.99	0.99	0.98 (0.96-0.99)	0.99 (0.97-0.99)	0.99 (0.98-1.00)
Right mean cortical tickness (mm)	2.39 (0.10)	2.38 (0.10)	2.39 (0.10)	2.39 (0.10)	4.26	4.36	4.20	4.36	1.47 (0.16)	1.35 (0.19)	0.63 (0.54)	-0.36 (1.12)	-0.22 (0.74)	-0.09 (0.60)	0.61 (1.00)	0.43 (0.63)	0.47 (0.37)	0.97	0.98	0.99	0.97 (0.92-0.99)	0.98 (0.96-0.99)	0.99 (0.98-1.00)
Left cerebellar white matter (ml)	14.97 (1.60)	14.84 (1.26)	15.01 (1.59)	14.58 (1.40)	10.66	8.46	10.62	9.58	0.83 (0.42)	-0.37 (0.71)	2.43 (0.02)	-0.67 (4.53)	0.27 (3.34)	-2.58 (5.03)	3.42 (2.96)	2.61 (2.01)	4.10 (3.83)	0.90	0.95	0.88	0.88 (0.73-0.95)	0.95 (0.89-0.98)	0.85 (0.63-0.94)
Right cerebellar white matter (ml)	15.11 (2.82)	14.51 (1.14)	14.68 (1.69)	14.80 (3.70)	18.69	7.85	11.50	25.03	1.27 (0.22)	0.83 (0.42)	0.44 (0.67)	-2.91 (11.13)	-2.01 (12.25)	-2.79 (15.32)	5.73 (9.92)	5.41 (11.12)	7.95 (13.29)	0.72	0.56	0.53	0.49 (0.10-0.75)	0.50 (0.09-0.76)	0.52 (0.11-0.77)
Left cerebellar gray matter (ml)	48.43 (2.68)	48.69 (2.90)	48.49 (2.79)	48.92 (2.80)	5.54	5.96	5.76	5.71	-1.34 (0.20)	-0.48 (0.64)	-3.12 (0.01)	0.51 (1.79)	0.12 (1.20)	1.01 (1.47)	1.16 (1.44)	0.81 (0.87)	1.23 (1.27)	0.95	0.98	0.97	0.95 (0.88-0.98)	0.98 (0.94-0.99)	0.95 (0.82-0.98)
Right cerebellar gray matter (ml)	48.34 (2.44)	48.60 (2.50)	48.65 (2.42)	48.61 (3.31)	5.04	5.15	4.97	6.81	-0.59 (0.56)	-0.76 (0.46)	-0.46 (0.65)	0.51 (4.07)	0.63 (3.75)	0.43 (5.75)	1.78 (3.68)	1.29 (3.57)	2.51 (5.16)	0.68	0.71	0.62	0.69 (0.37-0.86)	0.72 (0.42-0.87)	0.60 (0.23-0.82)
Intracranial volume (ml)	1494.49 (193.75)	1485.25 (205.52)	1489.70 (192.09)	1517.75 (172.28)	12.96	13.84	12.89	11.35	1.03 (0.31)	3.38 (0.00)	-2.03 (0.06)	-0.76 (3.57)	-0.31 (0.44)	1.75 (4.04)	1.21 (3.44)	0.42 (0.34)	1.75 (4.04)	0.98	1.00	0.97	0.98 (0.95-0.99)	1.00 (1.00-1.00)	0.95 (0.88-0.98)
Total brain volume without ventricles (ml)	1037.32 (91.14)	1031.95 (96.27)	1035.39 (92.84)	1036.89 (91.84)	8.79	9.33	8.97	8.86	1.35 (0.19)	2.06 (0.05)	0.49 (0.63)	-0.57 (1.92)	-0.20 (0.42)	-0.05 (0.37)	0.73 (1.86)	0.35 (0.30)	0.28 (0.24)	0.98	1.00	1.00	0.98 (0.95-0.99)	1.00 (1.00-1.00)	1.00 (1.00-1.00)

Abbreviations: SD = standard deviation; Orig = original scan; FS df = FreeSurfer defacing; FSL df = FSL defacing; FM = Face Masking; t = t-statistic; *p*=p-value; PD = Average Percentage Difference; CI = Confidence Interval.

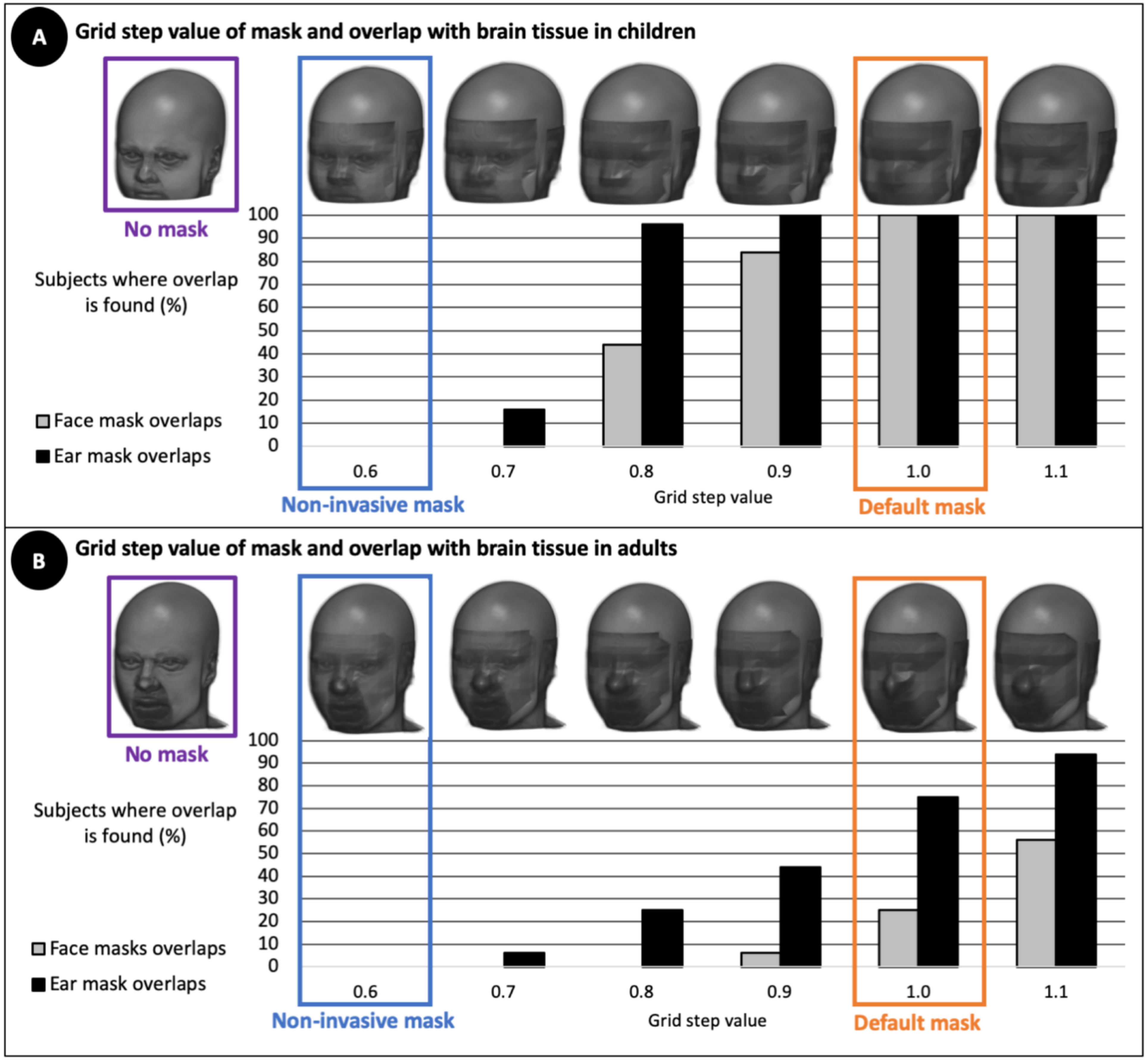

Figure S3. De-identification properties and invasiveness of masks with varying coarseness.
Invasiveness is defined as percentage of subjects where the mask overlapped cortical gray matter on multiple axial slices. To find the optimal mask settings we altered the coarseness of the masks by varying the grid step value (x-axis) and calculated the percentage of subjects where the face mask (grey) or ear mask (black) overlapped cortical gray matter (y-axis). Box A shows the child data (N=25). The face renderings shown here are visualizations of the average child created from the 25 children in this study. Box B shows the young adult data (N=16). The face renderings shown here are visualizations of the average young adult created from the 16 young adults in this study. The render of the non-invasive mask that did not overlap in any individual is indicated with blue and the mask with default coarseness with orange. In the purple square the unmasked brain is shown for comparison.

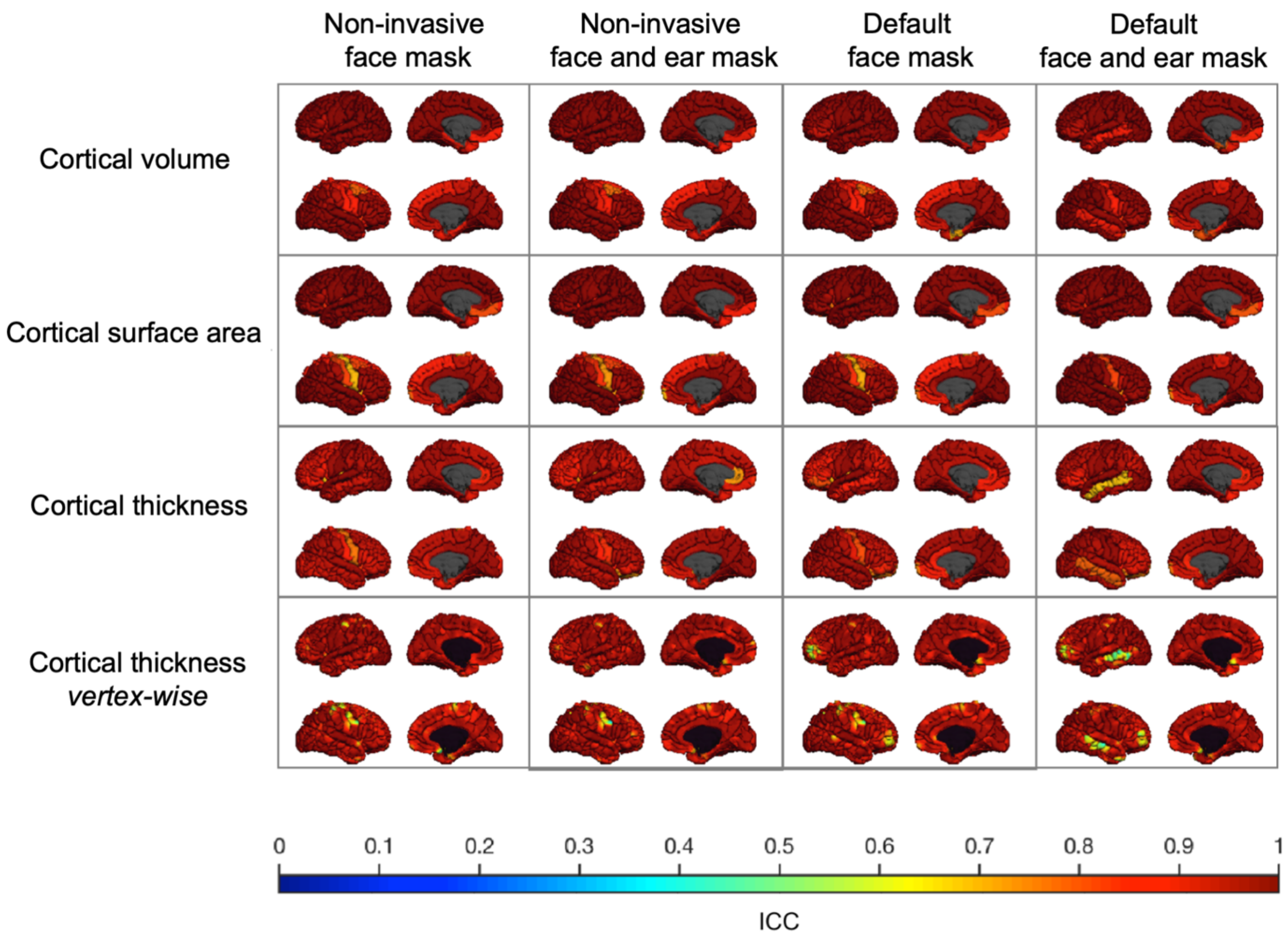

Figure S4. ICC between brain measures in children derived from original scans versus scans subjected to different mask settings.
The ICC for each brain measure is plotted on the average child scan. Each column shows a different masking setting. Within each square, the left hemisphere (top) and the right hemisphere (bottom) are shown from an outer and medial view.

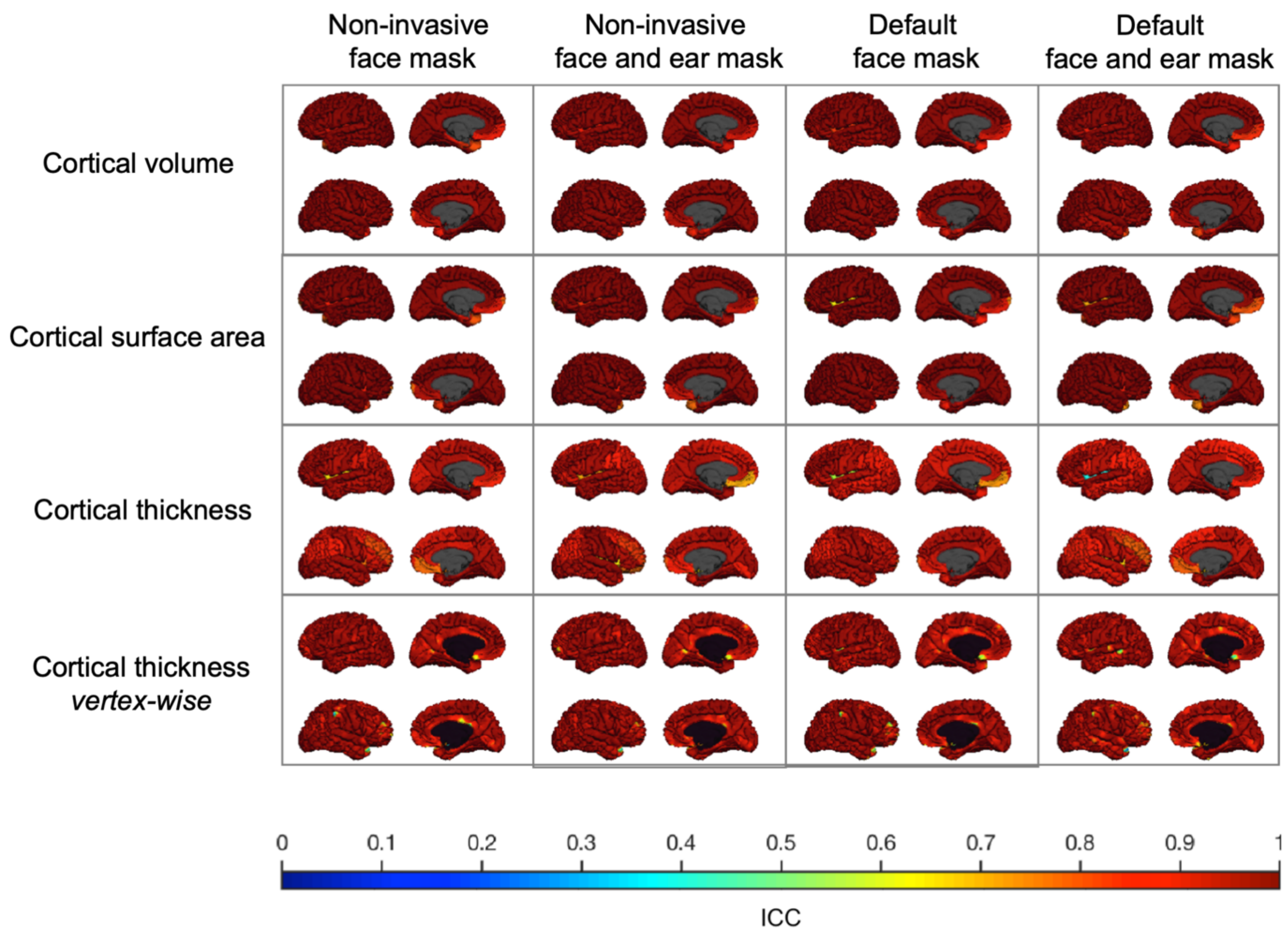

Figure S5. ICC between brain measures in adults derived from original scans versus scans subjected to different mask settings.
The ICC for each brain measure is plotted on the average adult scan. Each column shows a different masking setting. Within each square, the left hemisphere (top) and the right hemisphere (bottom) are shown from an outer and medial view.

Table S6. Effects of de-identification on test-retest reliability in young adults: Means, coefficients of variation and t-tests

Brain metric	Mean (SD)								Coefficient of Variation								Paired t-test t(*p*)						
	Orig1	Orig2	FS df1	FS df2	FSL df1	FSL df2	FM1	FM2	Orig1	Orig2	FS df1	FS df2	FSL df1	FSL df2	FM1	FM2	Orig1 - Orig2	FS df1 - FS df2	Orig1 - FS df2	FSL df1 - FSL df2	Orig1 - FSL df2	FM1 - FM2	Orig1 - FM2
Subcortical volume (ml)																							
Left thalamus	7.58 (0.91)	7.60 (0.93)	7.56 (0.95)	7.50 (0.83)	7.59 (0.88)	7.50 (0.91)	7.58 (0.83)	7.57 (0.87)	12.06	12.18	12.55	11.09	11.61	12.12	10.95	11.46	-0.38 (0.71)	0.81 (0.43)	1.19 (0.25)	1.50 (0.15)	1.94 (0.07)	0.19 (0.85)	0.15 (0.88)
Left caudatus	3.85 (0.40)	3.81 (0.41)	3.85 (0.41)	3.81 (0.40)	3.84 (0.39)	3.83 (0.41)	3.82 (0.39)	3.80 (0.38)	10.28	10.75	10.62	10.56	10.24	10.63	10.26	10.08	1.85 (0.08)	2.13 (0.05)	2.02 (0.06)	0.50 (0.63)	0.96 (0.35)	1.46 (0.17)	2.59 (0.02)
Left putamen	5.26 (0.74)	5.25 (0.70)	5.28 (0.70)	5.24 (0.69)	5.27 (0.72)	5.23 (0.67)	5.27 (0.69)	5.22 (0.68)	14.01	13.37	13.32	13.26	13.65	12.72	13.07	12.98	0.15 (0.89)	0.95 (0.36)	0.29 (0.78)	0.75 (0.47)	0.42 (0.68)	1.69 (0.11)	0.77 (0.45)
Left pallidum	1.92 (0.24)	1.92 (0.21)	1.96 (0.24)	1.92 (0.23)	1.94 (0.23)	1.91 (0.22)	1.93 (0.22)	1.91 (0.24)	12.57	11.03	12.36	11.83	11.83	11.62	11.6	12.54	-0.03 (0.98)	1.36 (0.19)	-0.01 (0.99)	1.25 (0.23)	0.26 (0.80)	0.47 (0.64)	0.29 (0.77)
Left hippocampus	4.11 (0.34)	4.13 (0.33)	4.12 (0.35)	4.11 (0.31)	4.11 (0.34)	4.11 (0.34)	4.13 (0.36)	4.15 (0.31)	8.32	8.04	8.49	7.52	8.24	8.19	8.8	7.56	-1.16 (0.26)	0.24 (0.81)	-0.13 (0.90)	-0.09 (0.93)	0.13 (0.90)	-0.81 (0.43)	-1.79 (0.09)
Left amygdala	1.73 (0.23)	1.74 (0.27)	1.74 (0.24)	1.73 (0.24)	1.74 (0.24)	1.72 (0.24)	1.72 (0.25)	1.72 (0.26)	13.41	15.39	13.48	13.79	13.93	13.96	14.55	15.17	-0.50 (0.63)	0.65 (0.53)	-0.05 (0.96)	0.34 (0.74)	0.09 (0.93)	0.06 (0.95)	0.21 (0.84)
Left accumbens	0.62 (0.14)	0.65 (0.16)	0.64 (0.14)	0.64 (0.15)	0.64 (0.15)	0.63 (0.13)	0.65 (0.16)	0.65 (0.16)	22.05	24.48	22.74	24.13	23.46	21.25	24.21	24.66	-1.61 (0.13)	-0.52 (0.61)	-1.12 (0.28)	0.43 (0.67)	-0.40 (0.69)	-0.10 (0.92)	-1.57 (0.14)
Right thalamus	7.21 (0.85)	7.13 (0.87)	7.19 (0.84)	7.08 (0.81)	7.23 (0.83)	7.10 (0.83)	7.23 (0.85)	7.09 (0.77)	11.81	12.16	11.71	11.52	11.48	11.74	11.71	10.92	1.69 (0.11)	1.89 (0.08)	2.28 (0.04)	2.55 (0.02)	2.04 (0.06)	2.22 (0.04)	1.89 (0.08)
Right caudatus	3.92 (0.37)	3.89 (0.37)	3.89 (0.37)	3.87 (0.36)	3.91 (0.37)	3.87 (0.34)	3.93 (0.39)	3.87 (0.35)	9.53	9.53	9.4	9.3	9.46	8.83	9.9	9.1	1.47 (0.16)	0.43 (0.67)	1.68 (0.11)	1.66 (0.12)	2.81 (0.01)	2.84 (0.01)	2.31 (0.04)
Right putamen	5.29 (0.66)	5.28 (0.67)	5.27 (0.68)	5.25 (0.65)	5.31 (0.67)	5.26 (0.64)	5.27 (0.66)	5.23 (0.65)	12.48	12.76	12.97	12.45	12.55	12.17	12.46	12.39	0.65 (0.53)	0.81 (0.43)	1.63 (0.12)	1.94 (0.07)	1.31 (0.21)	1.66 (0.12)	2.81 (0.01)
Right pallidum	1.89 (0.20)	1.88 (0.19)	1.90 (0.22)	1.89 (0.20)	1.89 (0.22)	1.89 (0.24)	1.87 (0.21)	1.89 (0.21)	10.85	10.16	11.53	10.83	11.91	12.74	11.27	11.37	0.43 (0.67)	0.30 (0.77)	-0.23 (0.82)	0.18 (0.86)	0.00 (1.00)	-0.67 (0.52)	-0.03 (0.98)
Right hippocampus	4.25 (0.34)	4.30 (0.37)	4.27 (0.35)	4.26 (0.34)	4.27 (0.35)	4.27 (0.35)	4.26 (0.34)	4.28 (0.38)	7.94	8.54	8.25	8.04	8.12	8.26	8	8.98	-2.39 (0.03)	0.23 (0.82)	-0.25 (0.80)	0.17 (0.87)	-0.64 (0.53)	-0.55 (0.59)	-0.87 (0.40)
Right amygdala	1.90 (0.24)	1.87 (0.24)	1.87 (0.25)	1.89 (0.26)	1.87 (0.25)	1.91 (0.26)	1.85 (0.24)	1.88 (0.26)	12.73	13.04	13.33	13.57	13.2	13.74	12.96	13.66	0.90 (0.38)	-0.71 (0.49)	0.36 (0.72)	-1.11 (0.28)	-0.23 (0.82)	-0.86 (0.40)	0.53 (0.60)
Right accumbens	0.63 (0.12)	0.64 (0.12)	0.63 (0.13)	0.65 (0.13)	0.63 (0.12)	0.64 (0.12)	0.63 (0.13)	0.64 (0.13)	19.31	18.71	20.35	19.84	19.27	18.92	20.51	20.22	-0.40 (0.70)	-0.83 (0.42)	-0.89 (0.39)	-0.57 (0.58)	-0.56 (0.58)	-0.67 (0.51)	-0.52 (0.61)
Ventricular volume (ml)																							
Third ventricle	0.86 (0.38)	0.88 (0.39)	0.86 (0.39)	0.87 (0.39)	0.85 (0.39)	0.87 (0.40)	0.86 (0.39)	0.88 (0.39)	44.70	44.44	45.12	44.55	45.64	45.71	45.09	44.57	-1.49 (0.16)	-1.50 (0.16)	-1.25 (0.23)	-1.43 (0.17)	-0.77 (0.45)	-1.56 (0.14)	-1.50 (0.15)
Left lateral ventricle	8.45 (5.15)	8.51 (5.15)	8.45 (5.15)	8.48 (5.17)	8.46 (5.14)	8.51 (5.16)	8.44 (5.14)	8.48 (5.16)	60.95	60.60	61	60.97	60.72	60.6	60.9	60.78	-1.49 (0.16)	-0.78 (0.45)	-0.73 (0.47)	-1.16 (0.27)	-1.58 (0.13)	-0.92 (0.37)	-0.91 (0.38)
Left inferior lateral ventricle	0.41 (0.23)	0.43 (0.21)	0.41 (0.21)	0.43 (0.20)	0.43 (0.21)	0.43 (0.21)	0.41 (0.23)	0.43 (0.22)	55.57	50.38	50.2	46.64	49.35	50.38	55.77	52.24	-1.27 (0.22)	-1.48 (0.16)	-1.37 (0.19)	0.20 (0.85)	-0.86 (0.40)	-0.99 (0.34)	-1.02 (0.33)
Right lateral ventricle	7.02 (3.69)	7.08 (3.69)	6.99 (3.68)	7.07 (3.65)	6.99 (3.70)	7.05 (3.68)	7.02 (3.68)	7.08 (3.69)	52.60	52.10	52.72	51.64	52.94	52.17	52.39	52.2	-1.74 (0.10)	-1.92 (0.07)	-1.32 (0.21)	-1.54 (0.15)	-0.88 (0.39)	-1.56 (0.14)	-1.65 (0.12)
Right inferior lateral ventricle	0.38 (0.18)	0.39 (0.18)	0.38 (0.19)	0.39 (0.18)	0.38 (0.18)	0.38 (0.18)	0.37 (0.19)	0.38 (0.19)	47.35	45.80	49.6	46.95	46.72	47.82	51.17	49.58	-0.86 (0.40)	-1.05 (0.31)	-1.17 (0.26)	0.02 (0.99)	0.23 (0.82)	-0.94 (0.36)	-0.39 (0.70)
Local cortical volume (ml)																							
Left banks superior temporal sulcus	2.74 (0.53)	2.73 (0.56)	2.71 (0.50)	2.74 (0.53)	2.73 (0.54)	2.68 (0.55)	2.68 (0.49)	2.71 (0.52)	19.45	20.64	18.3	19.46	19.86	20.32	18.13	19.21	0.21 (0.84)	-0.97 (0.35)	-0.06 (0.95)	1.40 (0.18)	1.38 (0.19)	-1.04 (0.31)	0.77 (0.46)
Left caudal anterior cingulate cortex	1.96 (0.57)	1.88 (0.51)	1.94 (0.57)	1.91 (0.58)	1.95 (0.56)	1.92 (0.57)	1.95 (0.57)	1.94 (0.54)	28.91	27.32	29.45	30.65	28.44	29.88	28.95	27.88	2.58 (0.02)	1.08 (0.30)	1.85 (0.08)	1.13 (0.28)	1.27 (0.22)	0.71 (0.49)	0.81 (0.43)
Left caudal middle frontal gyrus	6.53 (0.89)	6.52 (0.97)	6.50 (0.87)	6.44 (0.94)	6.54 (0.90)	6.47 (0.98)	6.55 (0.89)	6.51 (0.94)	13.57	14.89	13.32	14.65	13.82	15.13	13.66	14.43	0.17 (0.87)	1.02 (0.32)	1.36 (0.19)	1.19 (0.25)	0.91 (0.38)	0.96 (0.35)	0.31 (0.76)
Left cuneus	3.39 (0.63)	3.37 (0.58)	3.36 (0.60)	3.37 (0.59)	3.38 (0.62)	3.39 (0.63)	3.39 (0.64)	3.38 (0.61)	18.67	17.13	17.82	17.68	18.27	18.51	18.84	18.1	0.47 (0.65)	-0.23 (0.82)	0.59 (0.56)	-0.17 (0.87)	-0.14 (0.89)	0.26 (0.80)	0.01 (0.99)
Left entorhinal cortex	2.03 (0.34)	2.04 (0.28)	2.00 (0.32)	2.02 (0.36)	2.04 (0.31)	2.01 (0.32)	2.04 (0.38)	2.01 (0.39)	16.96	13.64	16.11	18.01	14.99	15.67	18.8	19.48	-0.26 (0.80)	-0.49 (0.63)	0.19 (0.86)	0.43 (0.67)	0.30 (0.77)	0.61 (0.55)	0.30 (0.77)
Left fusiform gyrus	10.75 (1.15)	10.84 (1.10)	10.69 (1.14)	10.83 (1.08)	10.66 (1.23)	10.81 (1.13)	10.70 (1.21)	10.90 (1.22)	10.74	10.13	10.69	9.98	11.5	10.49	11.27	11.2	-1.29 (0.21)	-1.72 (0.11)	-0.88 (0.39)	-1.54 (0.14)	-0.65 (0.52)	-2.02 (0.06)	-1.81 (0.09)
Left inferior parietal cortex	13.36 (2.06)	13.35 (2.09)	13.31 (2.09)	13.33 (2.09)	13.39 (2.18)	13.41 (2.23)	13.43 (2.07)	13.39 (2.11)	15.41	15.63	15.73	15.65	16.3	16.64	15.43	15.79	0.10 (0.92)	-0.24 (0.81)	0.52 (0.61)	-0.20 (0.85)	-0.49 (0.63)	0.40 (0.70)	-0.48 (0.64)
Left inferior temporal gyrus	11.50 (1.31)	11.46 (1.43)	11.46 (1.42)	11.51 (1.39)	11.53 (1.46)	11.53 (1.53)	11.45 (1.41)	11.53 (1.49)	11.42	12.45	12.36	12.1	12.66	13.28	12.34	12.9	0.38 (0.71)	-0.45 (0.66)	-0.10 (0.92)	0.04 (0.97)	-0.28 (0.78)	-0.81 (0.43)	-0.31 (0.76)
Left isthmus cingulate cortex	2.91 (0.43)	2.94 (0.43)	2.93 (0.45)	2.92 (0.42)	2.94 (0.48)	2.94 (0.42)	2.94 (0.43)	2.93 (0.41)	14.86	14.64	15.51	14.26	16.28	14.27	14.67	14.03	-0.89 (0.39)	0.67 (0.51)	-0.42 (0.68)	0.30 (0.77)	-0.82 (0.43)	0.18 (0.86)	-0.60 (0.56)
Left lateral occipital cortex	12.84 (2.13)	12.80 (2.10)	12.85 (2.06)	12.76 (2.03)	12.85 (2.17)	12.73 (2.03)	12.81 (2.08)	12.86 (2.02)	16.56	16.40	16	15.91	16.85	15.93	16.26	15.74	0.75 (0.47)	1.14 (0.27)	1.53 (0.15)	1.41 (0.18)	1.77 (0.10)	-0.87 (0.40)	-0.26 (0.80)
Left lateral orbitofrontal cortex	8.01 (0.82)	7.96 (0.83)	8.04 (0.76)	8.02 (0.83)	8.02 (0.85)	8.00 (0.86)	8.04 (0.81)	8.04 (0.85)	10.23	10.45	9.45	10.34	10.6	10.77	10.04	10.53	0.66 (0.52)	0.28 (0.78)	-0.21 (0.83)	0.29 (0.78)	0.15 (0.88)	-0.08 (0.94)	-0.43 (0.67)
Left lingual gyrus	6.98 (1.22)	6.93 (1.23)	6.99 (1.22)	6.93 (1.16)	7.00 (1.24)	6.95 (1.22)	6.98 (1.24)	6.92 (1.21)	17.50	17.80	17.51	16.75	17.67	17.54	17.79	17.45	1.04 (0.31)	1.06 (0.31)	0.90 (0.38)	1.28 (0.22)	0.67 (0.51)	1.21 (0.24)	1.12 (0.28)
Left medial orbitofrontal cortex	5.22 (0.86)	5.17 (0.78)	5.06 (0.62)	5.16 (0.69)	5.11 (0.79)	5.13 (0.67)	5.21 (0.78)	5.25 (0.83)	16.49	15.07	12.22	13.35	15.5	13.17	14.97	15.8	0.56 (0.59)	-0.98 (0.34)	0.58 (0.57)	-0.15 (0.88)	0.75 (0.46)	-0.49 (0.63)	-0.20 (0.85)
Left middle temporal gyrus	11.22 (1.29)	11.29 (1.31)	11.25 (1.22)	11.25 (1.30)	11.24 (1.29)	11.29 (1.34)	11.20 (1.27)	11.24 (1.27)	11.52	11.63	10.81	11.55	11.46	11.83	11.32	11.29	-0.76 (0.46)	0.06 (0.95)	-0.39 (0.70)	-0.64 (0.53)	-0.89 (0.39)	-0.32 (0.75)	-0.14 (0.89)
Left parahippocampal gyrus	2.39 (0.28)	2.38 (0.27)	2.38 (0.26)	2.37 (0.27)	2.39 (0.28)	2.39 (0.26)	2.40 (0.27)	2.38 (0.25)	11.72	11.24	11.07	11.21	11.79	11.06	11.05	10.4	0.29 (0.77)	0.29 (0.78)	0.54 (0.60)	-0.18 (0.86)	-0.05 (0.96)	0.78 (0.44)	0.49 (0.63)

Left paracentral lobule	3.85 (0.60)	3.83 (0.61)	3.86 (0.57)	3.85 (0.65)	3.88 (0.59)	3.87 (0.65)	3.86 (0.55)	3.88 (0.69)	15.51	15.92	14.76	17	15.33	16.87	14.36	17.71	0.85 (0.41)	0.50 (0.62)	0.30 (0.77)	0.26 (0.80)	-0.59 (0.57)	-0.51 (0.61)	-0.69 (0.50)
Left pars opercularis	4.93 (0.73)	4.83 (0.78)	4.84 (0.71)	4.87 (0.72)	4.86 (0.78)	4.86 (0.76)	4.85 (0.70)	4.85 (0.71)	14.78	16.04	14.75	14.81	16.13	15.56	14.53	14.55	2.32 (0.03)	-0.65 (0.53)	1.54 (0.14)	0.02 (0.99)	1.94 (0.07)	-0.05 (0.96)	2.08 (0.05)
Left pars orbitalis	2.53 (0.34)	2.52 (0.31)	2.54 (0.32)	2.52 (0.32)	2.54 (0.34)	2.48 (0.34)	2.53 (0.35)	2.55 (0.35)	13.49	12.42	12.58	12.57	13.38	13.85	13.81	13.9	0.52 (0.61)	0.64 (0.53)	0.34 (0.74)	1.96 (0.07)	1.94 (0.07)	-0.56 (0.58)	-0.63 (0.54)
Left pars triangularis	3.89 (0.73)	3.88 (0.72)	3.91 (0.73)	3.89 (0.75)	3.89 (0.74)	3.90 (0.74)	3.93 (0.74)	3.90 (0.75)	18.87	18.52	18.65	19.14	19.04	18.95	18.9	19.16	0.22 (0.83)	0.40 (0.69)	-0.06 (0.95)	-0.32 (0.75)	-0.16 (0.88)	0.74 (0.47)	-0.32 (0.75)
Left pericalcarine cortex	2.19 (0.47)	2.14 (0.42)	2.19 (0.48)	2.16 (0.43)	2.20 (0.46)	2.15 (0.41)	2.17 (0.47)	2.11 (0.41)	21.62	19.58	22	19.67	20.89	19.27	21.76	19.53	1.95 (0.07)	0.70 (0.50)	0.86 (0.40)	2.14 (0.05)	1.67 (0.11)	2.20 (0.04)	2.51 (0.02)
Left postcentral gyrus	10.23 (1.45)	10.22 (1.58)	10.21 (1.44)	10.18 (1.53)	10.23 (1.46)	10.19 (1.49)	10.20 (1.43)	10.22 (1.61)	14.21	15.48	14.09	15.02	14.22	14.6	14.02	15.79	0.21 (0.84)	0.50 (0.63)	1.02 (0.32)	0.79 (0.44)	0.63 (0.54)	-0.22 (0.83)	0.26 (0.80)
Left posterior cingulate cortex	3.49 (0.60)	3.48 (0.61)	3.51 (0.57)	3.49 (0.60)	3.51 (0.60)	3.48 (0.61)	3.50 (0.61)	3.48 (0.61)	17.04	17.39	16.12	17.07	17.08	17.55	17.51	17.41	0.45 (0.66)	1.08 (0.30)	0.35 (0.73)	1.34 (0.20)	0.75 (0.46)	0.87 (0.40)	0.68 (0.51)
Left precentral gyrus	13.69 (1.36)	13.75 (1.32)	13.69 (1.30)	13.66 (1.39)	13.72 (1.32)	13.70 (1.42)	13.73 (1.39)	13.73 (1.40)	9.96	9.61	9.46	10.2	9.63	10.35	10.11	10.21	-1.00 (0.33)	0.68 (0.51)	0.52 (0.61)	0.30 (0.77)	-0.13 (0.90)	-0.01 (0.99)	-0.52 (0.61)
Left precuneus	10.50 (1.19)	10.37 (1.25)	10.41 (1.25)	10.39 (1.25)	10.49 (1.30)	10.35 (1.30)	10.48 (1.22)	10.45 (1.30)	11.31	12.09	11.96	12.01	12.38	12.61	11.61	12.44	2.24 (0.04)	0.57 (0.57)	1.79 (0.09)	3.27 (0.01)	2.46 (0.03)	0.69 (0.50)	0.82 (0.42)
Left rostral anterior cingulate cortex	3.06 (0.50)	3.03 (0.53)	3.05 (0.52)	3.06 (0.50)	3.09 (0.51)	3.07 (0.50)	3.09 (0.51)	3.05 (0.50)	16.48	17.46	16.9	16.19	16.39	16.43	16.65	16.44	1.00 (0.33)	-0.19 (0.85)	-0.07 (0.95)	0.55 (0.59)	-0.32 (0.75)	0.99 (0.34)	0.27 (0.79)
Left rostral middle frontal gyrus	15.34 (2.11)	15.20 (2.09)	15.34 (2.04)	15.23 (2.06)	15.32 (2.10)	15.13 (2.15)	15.38 (2.00)	15.25 (1.96)	13.77	13.77	13.28	13.51	13.73	14.24	12.99	12.84	1.30 (0.21)	0.88 (0.39)	0.92 (0.37)	1.60 (0.13)	1.95 (0.07)	1.19 (0.25)	0.87 (0.40)
Left superior frontal gyrus	23.51 (2.34)	23.32 (2.31)	23.55 (2.33)	23.28 (2.22)	23.37 (2.44)	23.10 (2.30)	23.57 (2.40)	23.38 (2.26)	9.94	9.92	9.89	9.55	10.44	9.96	10.2	9.65	1.03 (0.32)	1.82 (0.09)	1.40 (0.18)	1.49 (0.16)	2.39 (0.03)	1.08 (0.30)	0.65 (0.53)
Left superior parietal cortex	14.57 (1.57)	14.52 (1.68)	14.56 (1.59)	14.51 (1.68)	14.58 (1.65)	14.48 (1.78)	14.58 (1.70)	14.48 (1.65)	10.74	11.59	10.94	11.59	11.34	12.3	11.63	11.38	0.67 (0.51)	0.67 (0.51)	0.88 (0.39)	1.19 (0.25)	0.96 (0.35)	1.36 (0.19)	1.28 (0.22)
Left superior temporal gyrus	12.23 (1.27)	12.27 (1.33)	12.28 (1.28)	12.25 (1.32)	12.27 (1.33)	12.22 (1.31)	12.31 (1.26)	12.30 (1.28)	10.35	10.83	10.39	10.79	10.84	10.73	10.24	10.38	-0.46 (0.65)	0.39 (0.71)	-0.19 (0.86)	0.69 (0.50)	0.11 (0.91)	0.08 (0.94)	-0.62 (0.54)
Left supramarginal gyrus	11.97 (1.60)	11.94 (1.66)	11.97 (1.58)	11.96 (1.67)	11.97 (1.66)	11.99 (1.73)	11.98 (1.69)	12.05 (1.67)	13.39	13.90	13.24	13.97	13.87	14.43	14.1	13.87	0.34 (0.74)	0.15 (0.88)	0.07 (0.94)	-0.33 (0.74)	-0.31 (0.76)	-1.03 (0.32)	-0.96 (0.35)
Left frontal pole	1.04 (0.14)	1.03 (0.12)	1.03 (0.14)	1.03 (0.12)	1.00 (0.11)	1.02 (0.11)	1.04 (0.15)	1.02 (0.12)	13.60	11.50	13.72	12.12	11.33	11.25	13.96	11.52	0.35 (0.73)	0.17 (0.86)	0.40 (0.69)	-0.87 (0.40)	0.72 (0.48)	1.21 (0.25)	0.60 (0.56)
Left temporal pole	2.63 (0.23)	2.70 (0.23)	2.66 (0.22)	2.73 (0.25)	2.65 (0.25)	2.72 (0.28)	2.65 (0.24)	2.73 (0.31)	8.66	8.36	8.29	9.17	9.61	10.29	9.25	11.42	-1.39 (0.19)	-1.79 (0.09)	-2.55 (0.02)	-2.05 (0.06)	-1.95 (0.07)	-1.37 (0.19)	-1.77 (0.10)
Left transverse temporal gyrus	1.33 (0.19)	1.34 (0.24)	1.33 (0.20)	1.33 (0.20)	1.33 (0.22)	1.33 (0.21)	1.33 (0.20)	1.33 (0.21)	14.70	17.66	15.39	14.84	16.64	15.78	14.76	15.5	-0.72 (0.48)	0.15 (0.88)	0.00 (1.00)	0.42 (0.68)	-0.19 (0.85)	-0.34 (0.74)	-0.67 (0.51)
Left insula	7.80 (0.81)	7.84 (0.68)	7.89 (1.08)	7.78 (0.74)	7.99 (0.94)	7.93 (0.84)	7.87 (0.90)	7.88 (0.71)	10.40	8.73	13.73	9.47	11.8	10.57	11.4	9.05	-0.30 (0.77)	0.83 (0.42)	0.18 (0.86)	0.43 (0.67)	-0.89 (0.39)	-0.12 (0.90)	-0.58 (0.57)
Right banks superior temporal sulcus	2.31 (0.32)	2.33 (0.37)	2.31 (0.34)	2.35 (0.36)	2.36 (0.35)	2.29 (0.39)	2.32 (0.40)	2.33 (0.38)	13.91	16.00	14.73	15.18	15.01	17.08	17.05	16.47	-0.67 (0.52)	-1.66 (0.12)	-1.70 (0.11)	2.22 (0.04)	0.66 (0.52)	-0.27 (0.79)	-0.59 (0.56)
Right caudal anterior cingulate cortex	2.45 (0.63)	2.34 (0.55)	2.45 (0.62)	2.33 (0.54)	2.42 (0.58)	2.35 (0.54)	2.45 (0.63)	2.34 (0.57)	25.86	23.54	25.43	23.15	23.93	22.85	25.54	24.21	1.87 (0.08)	1.63 (0.12)	1.39 (0.19)	2.47 (0.03)	1.48 (0.16)	1.51 (0.15)	1.58 (0.13)
Right caudal middle frontal gyrus	6.49 (0.84)	6.41 (0.86)	6.50 (0.88)	6.40 (0.83)	6.49 (0.83)	6.44 (0.85)	6.45 (0.82)	6.43 (0.81)	12.89	13.42	13.51	13.03	12.75	13.25	12.65	12.68	1.22 (0.24)	1.79 (0.09)	1.32 (0.21)	0.82 (0.42)	0.81 (0.43)	0.42 (0.68)	0.95 (0.36)
Right cuneus	3.77 (0.74)	3.74 (0.72)	3.77 (0.76)	3.73 (0.72)	3.75 (0.69)	3.72 (0.71)	3.74 (0.72)	3.74 (0.72)	19.54	19.15	20.1	19.39	18.39	18.99	19.12	19.16	1.09 (0.29)	1.18 (0.26)	1.26 (0.23)	1.31 (0.21)	1.40 (0.18)	0.02 (0.98)	1.12 (0.28)
Right entorhinal cortex	2.01 (0.33)	1.96 (0.43)	1.95 (0.46)	1.93 (0.47)	2.00 (0.38)	1.98 (0.41)	2.01 (0.41)	2.02 (0.50)	16.22	22.08	23.65	24.32	18.8	20.54	20.36	24.58	0.58 (0.57)	0.17 (0.87)	0.83 (0.42)	0.22 (0.83)	0.32 (0.75)	-0.08 (0.93)	-0.12 (0.90)
Right fusiform gyrus	9.91 (1.62)	9.97 (1.51)	9.86 (1.52)	10.00 (1.49)	9.94 (1.45)	9.99 (1.58)	9.94 (1.52)	9.95 (1.52)	16.39	15.19	15.46	14.86	14.56	15.82	15.34	15.24	-0.60 (0.56)	-1.15 (0.27)	-0.79 (0.44)	-0.63 (0.54)	-0.89 (0.39)	-0.11 (0.92)	-0.37 (0.72)
Right inferior parietal cortex	15.28 (2.33)	15.18 (2.54)	15.24 (2.36)	15.16 (2.45)	15.21 (2.39)	15.20 (2.49)	15.16 (2.39)	15.33 (2.53)	15.22	16.71	15.51	16.16	15.71	16.38	15.76	16.51	0.82 (0.43)	0.89 (0.39)	1.17 (0.26)	0.15 (0.88)	0.83 (0.42)	-1.54 (0.14)	-0.46 (0.65)
Right inferior temporal gyrus	10.78 (1.13)	10.82 (0.98)	10.81 (1.14)	10.87 (1.04)	10.76 (1.11)	10.75 (1.13)	10.69 (1.13)	10.81 (0.98)	10.49	9.06	10.57	9.54	10.32	10.47	10.58	9.11	-0.45 (0.66)	-0.59 (0.56)	-0.95 (0.36)	0.03 (0.97)	0.19 (0.85)	-0.99 (0.34)	-0.29 (0.77)
Right isthmus cingulate cortex	2.84 (0.55)	2.85 (0.50)	2.85 (0.59)	2.84 (0.54)	2.86 (0.56)	2.84 (0.52)	2.86 (0.58)	2.85 (0.54)	19.35	17.55	20.73	19.14	19.55	18.2	20.09	18.86	-0.27 (0.79)	0.06 (0.96)	-0.15 (0.89)	0.48 (0.64)	0.03 (0.97)	0.56 (0.59)	-0.26 (0.80)
Right lateral occipital cortex	13.27 (2.52)	13.23 (2.53)	13.21 (2.47)	13.22 (2.43)	13.23 (2.55)	13.19 (2.32)	13.18 (2.49)	13.24 (2.30)	18.96	19.12	18.68	18.39	19.25	17.58	18.87	17.39	0.53 (0.60)	-0.08 (0.94)	0.64 (0.53)	0.36 (0.73)	0.73 (0.48)	-0.53 (0.61)	0.32 (0.76)
Right lateral orbitofrontal cortex	8.06 (0.98)	8.09 (0.97)	8.22 (1.06)	8.15 (0.85)	8.01 (0.98)	8.10 (1.01)	8.09 (0.84)	8.10 (0.91)	12.20	11.93	12.89	10.46	12.23	12.51	10.36	11.19	-0.34 (0.74)	0.59 (0.56)	-1.20 (0.25)	-0.87 (0.40)	-0.51 (0.62)	-0.07 (0.95)	-0.39 (0.70)
Right lingual gyrus	7.62 (1.12)	7.58 (1.12)	7.60 (1.13)	7.59 (1.14)	7.60 (1.15)	7.58 (1.10)	7.61 (1.10)	7.58 (1.12)	14.75	14.77	14.87	14.97	15.16	14.48	14.52	14.73	0.76 (0.46)	0.14 (0.89)	0.62 (0.55)	0.41 (0.69)	1.05 (0.31)	0.40 (0.69)	0.67 (0.51)
Right medial orbitofrontal cortex	6.01 (0.74)	5.99 (0.78)	5.87 (0.75)	5.96 (0.78)	5.95 (0.73)	5.92 (0.70)	6.02 (0.78)	5.92 (0.76)	12.37	13.01	12.74	13.11	12.36	11.83	12.9	12.9	0.36 (0.72)	-1.67 (0.12)	1.21 (0.24)	0.37 (0.72)	1.71 (0.11)	1.59 (0.13)	1.67 (0.12)
Right middle temporal gyrus	12.16 (1.20)	12.20 (1.23)	12.16 (1.23)	12.18 (1.21)	12.04 (1.30)	12.07 (1.30)	12.01 (1.19)	12.14 (1.20)	9.85	10.06	10.11	9.9	10.77	10.76	9.94	9.85	-0.54 (0.60)	-0.25 (0.81)	-0.23 (0.82)	-0.33 (0.74)	0.89 (0.39)	-1.79 (0.09)	0.32 (0.76)
Right parahippocampal gyrus	2.20 (0.40)	2.23 (0.42)	2.22 (0.35)	2.23 (0.39)	2.23 (0.42)	2.22 (0.40)	2.24 (0.37)	2.25 (0.40)	18.36	18.71	15.66	17.35	18.65	17.86	16.39	17.65	-1.46 (0.16)	-0.36 (0.72)	-1.52 (0.15)	0.51 (0.61)	-0.69 (0.50)	-0.47 (0.64)	-2.76 (0.01)
Right paracentral lobule	4.31 (0.57)	4.26 (0.56)	4.31 (0.62)	4.25 (0.55)	4.33 (0.57)	4.28 (0.56)	4.30 (0.55)	4.26 (0.56)	13.12	13.27	14.33	12.87	13.13	12.98	12.74	13.24	1.25 (0.23)	1.48 (0.16)	1.34 (0.20)	1.70 (0.11)	0.63 (0.54)	1.35 (0.20)	1.26 (0.23)
Right pars opercularis	4.48 (0.54)	4.50 (0.49)	4.50 (0.51)	4.48 (0.44)	4.42 (0.53)	4.48 (0.48)	4.47 (0.49)	4.45 (0.52)	12.00	10.88	11.33	9.72	11.95	10.8	10.95	11.72	-0.58 (0.57)	0.29 (0.78)	-0.07 (0.95)	-1.59 (0.13)	0.16 (0.87)	0.46 (0.65)	0.83 (0.42)
Right pars orbitalis	3.00 (0.40)	3.01 (0.37)	3.01 (0.37)	3.01 (0.36)	3.00 (0.40)	2.99 (0.41)	2.99 (0.41)	3.03 (0.38)	13.16	12.13	12.28	12.11	13.23	13.64	13.85	12.58	-0.33 (0.75)	-0.34 (0.74)	-0.39 (0.70)	0.36 (0.72)	0.38 (0.71)	-1.23 (0.24)	-0.80 (0.44)
Right pars triangularis	4.72 (0.72)	4.66 (0.64)	4.71 (0.73)	4.68 (0.69)	4.72 (0.73)	4.66 (0.66)	4.73 (0.73)	4.71 (0.67)	15.20	13.74	15.57	14.83	15.39	14.14	15.51	14.21	1.20 (0.25)	0.84 (0.42)	0.98 (0.34)	1.72 (0.11)	1.35 (0.20)	0.53 (0.60)	0.27 (0.79)
Right pericalcarine cortex	2.52 (0.54)	2.47 (0.49)	2.52 (0.52)	2.48 (0.49)	2.51 (0.56)	2.49 (0.51)	2.53 (0.53)	2.48 (0.48)	21.41	19.86	20.84	19.76	22.18	20.45	20.87	19.2	1.49 (0.16)	1.46 (0.17)	1.13 (0.27)	0.65 (0.52)	0.82 (0.43)	1.81 (0.09)	1.28 (0.22)
Right postcentral gyrus	9.48 (0.82)	9.48 (0.82)	9.50 (0.82)	9.52 (0.82)	9.48 (0.85)	9.52 (0.82)	9.44 (0.80)	9.48 (0.80)	8.60	8.67	8.62	8.66	8.94	8.64	8.45	8.44	0.02 (0.98)	-0.64 (0.53)	-0.85 (0.41)	-0.72 (0.48)	-0.69 (0.50)	-0.67 (0.51)	-0.01 (0.99)

Right posterior cingulate cortex	3.66 (0.58)	3.65 (0.58)	3.69 (0.59)	3.65 (0.58)	3.67 (0.59)	3.64 (0.58)	3.68 (0.58)	3.65 (0.59)	15.82	15.94	15.96	15.82	15.95	15.79	15.84	16.06	0.32 (0.75)	1.15 (0.27)	0.37 (0.72)	1.12 (0.28)	0.84 (0.42)	0.95 (0.36)	0.48 (0.64)
Right precentral gyrus	14.04 (1.49)	14.04 (1.53)	14.07 (1.53)	14.04 (1.54)	14.03 (1.49)	14.01 (1.50)	14.02 (1.45)	14.10 (1.55)	10.57	10.90	10.89	10.99	10.59	10.7	10.31	10.97	0.02 (0.98)	0.30 (0.77)	0.07 (0.95)	0.23 (0.82)	0.40 (0.69)	-0.71 (0.49)	-0.55 (0.59)
Right precuneus	10.80 (1.14)	10.72 (1.20)	10.71 (1.16)	10.76 (1.15)	10.76 (1.12)	10.73 (1.16)	10.70 (1.08)	10.74 (1.16)	10.52	11.22	10.84	10.7	10.44	10.77	10.08	10.79	1.02 (0.32)	-1.18 (0.26)	0.55 (0.59)	0.79 (0.44)	2.08 (0.06)	-0.75 (0.46)	0.69 (0.50)
Right rostral anterior cingulate cortex	2.38 (0.45)	2.39 (0.42)	2.39 (0.50)	2.37 (0.39)	2.37 (0.44)	2.37 (0.39)	2.37 (0.47)	2.39 (0.42)	19.07	17.41	20.85	16.65	18.47	16.55	19.81	17.7	-0.32 (0.76)	0.50 (0.62)	0.30 (0.77)	0.11 (0.91)	0.48 (0.64)	-0.59 (0.56)	-0.22 (0.83)
Right rostral middle frontal gyrus	16.08 (2.07)	15.92 (1.89)	16.08 (1.98)	15.92 (1.78)	15.92 (2.10)	15.75 (1.97)	16.01 (2.01)	15.91 (1.87)	12.86	11.87	12.3	11.19	13.21	12.53	12.54	11.76	0.91 (0.38)	1.07 (0.30)	1.01 (0.33)	1.38 (0.19)	2.37 (0.03)	0.77 (0.45)	1.12 (0.28)
Right superior frontal gyrus	23.13 (2.42)	22.99 (2.64)	23.15 (2.42)	22.99 (2.50)	22.95 (2.36)	22.77 (2.40)	23.00 (2.39)	23.02 (2.46)	10.45	11.49	10.44	10.89	10.27	10.54	10.39	10.7	0.77 (0.45)	0.84 (0.41)	0.68 (0.50)	1.34 (0.20)	2.08 (0.06)	-0.15 (0.88)	0.61 (0.55)
Right superior parietal cortex	13.49 (1.59)	13.57 (1.68)	13.51 (1.57)	13.56 (1.61)	13.51 (1.48)	13.49 (1.67)	13.54 (1.51)	13.56 (1.67)	11.75	12.37	11.61	11.89	10.93	12.38	11.13	12.29	-0.82 (0.43)	-0.49 (0.63)	-0.66 (0.52)	0.23 (0.82)	0.09 (0.93)	-0.26 (0.80)	-0.71 (0.49)
Right superior temporal gyrus	12.33 (1.25)	12.18 (1.30)	12.38 (1.28)	12.19 (1.24)	12.29 (1.22)	12.16 (1.28)	12.35 (1.25)	12.27 (1.23)	10.16	10.66	10.37	10.14	9.92	10.56	10.14	10.04	1.70 (0.11)	2.64 (0.02)	1.86 (0.08)	1.34 (0.20)	1.62 (0.13)	1.48 (0.16)	0.82 (0.42)
Right supramarginal gyrus	10.77 (1.58)	10.78 (1.65)	10.71 (1.57)	10.70 (1.61)	10.72 (1.61)	10.71 (1.67)	10.70 (1.54)	10.76 (1.62)	14.70	15.30	14.65	15.01	15	15.59	14.37	15	-0.23 (0.82)	0.36 (0.72)	1.40 (0.18)	0.24 (0.82)	1.14 (0.27)	-1.34 (0.20)	0.11 (0.92)
Right frontal pole	1.24 (0.12)	1.23 (0.13)	1.27 (0.15)	1.23 (0.16)	1.18 (0.15)	1.19 (0.19)	1.23 (0.11)	1.19 (0.12)	9.52	10.31	12.12	13.15	13.05	15.8	9.14	10.18	0.19 (0.85)	1.06 (0.31)	0.02 (0.98)	-0.36 (0.72)	1.18 (0.26)	1.64 (0.12)	2.19 (0.05)
Right temporal pole	2.67 (0.32)	2.67 (0.27)	2.66 (0.29)	2.67 (0.30)	2.62 (0.34)	2.66 (0.28)	2.64 (0.29)	2.61 (0.27)	12.17	10.26	10.92	11.05	12.84	10.68	10.83	10.37	-0.16 (0.87)	-0.20 (0.84)	-0.12 (0.90)	-0.67 (0.52)	0.12 (0.90)	0.86 (0.40)	1.16 (0.27)
Right transverse temporal gyrus	1.09 (0.20)	1.07 (0.19)	1.07 (0.19)	1.07 (0.17)	1.08 (0.19)	1.07 (0.18)	1.09 (0.21)	1.07 (0.20)	18.06	17.99	18.14	15.99	17.85	16.64	19.15	18.75	1.08 (0.30)	0.14 (0.89)	1.07 (0.30)	0.70 (0.50)	0.89 (0.39)	1.39 (0.18)	1.52 (0.15)
Right insula	7.55 (0.91)	7.54 (0.90)	7.44 (0.82)	7.49 (0.92)	7.51 (0.96)	7.47 (0.84)	7.47 (0.92)	7.69 (0.97)	12.10	11.88	10.97	12.33	12.79	11.26	12.28	12.66	0.10 (0.92)	-0.52 (0.61)	0.59 (0.56)	0.40 (0.69)	0.89 (0.39)	-2.36 (0.03)	-1.93 (0.07)
Local cortical surface (cm²)																							
Left banks superior temporal sulcus	10.73 (1.96)	10.67 (1.97)	10.64 (1.83)	10.74 (1.88)	10.67 (2.00)	10.58 (1.94)	10.52 (1.77)	10.66 (1.84)	18.24	18.42	17.22	17.53	18.75	18.31	16.81	17.28	0.53 (0.60)	-1.04 (0.31)	-0.12 (0.91)	1.07 (0.30)	1.27 (0.22)	-1.72 (0.11)	0.68 (0.51)
Left caudal anterior cingulate cortex	6.99 (1.84)	6.88 (1.72)	6.92 (1.81)	6.93 (1.95)	6.97 (1.82)	7.00 (1.89)	6.98 (1.74)	7.05 (1.85)	26.35	24.97	26.2	28.14	26.11	26.97	24.92	26.21	1.42 (0.18)	-0.03 (0.98)	0.91 (0.38)	-0.30 (0.77)	-0.09 (0.93)	-1.42 (0.18)	-1.48 (0.16)
Left caudal middle frontal gyrus	24.06 (3.51)	24.18 (3.43)	23.97 (3.38)	24.10 (3.48)	24.11 (3.43)	24.14 (3.47)	24.20 (3.44)	24.17 (3.48)	14.60	14.19	14.1	14.45	14.22	14.37	14.22	14.39	-1.04 (0.32)	-1.05 (0.31)	-0.23 (0.82)	-0.22 (0.83)	-0.53 (0.61)	0.23 (0.82)	-0.91 (0.38)
Left cuneus	16.57 (2.40)	16.47 (2.39)	16.51 (2.40)	16.56 (2.39)	16.54 (2.41)	16.62 (2.58)	16.65 (2.67)	16.62 (2.56)	14.49	14.52	14.56	14.44	14.59	15.54	16.05	15.4	0.79 (0.44)	-0.51 (0.62)	0.07 (0.94)	-0.60 (0.56)	-0.42 (0.68)	0.27 (0.79)	-0.58 (0.57)
Left entorhinal cortex	4.47 (0.80)	4.50 (0.75)	4.46 (0.76)	4.50 (0.78)	4.47 (0.72)	4.46 (0.72)	4.52 (0.88)	4.44 (0.77)	17.98	16.62	17.06	17.26	15.98	16.19	19.52	17.44	-0.25 (0.81)	-0.52 (0.61)	-0.28 (0.79)	0.23 (0.82)	0.11 (0.91)	1.01 (0.33)	0.31 (0.76)
Left fusiform gyrus	33.50 (4.76)	33.70 (4.76)	33.40 (4.70)	33.66 (4.60)	33.37 (4.73)	33.54 (4.71)	33.49 (4.84)	33.88 (4.89)	14.20	14.13	14.09	13.66	14.17	14.05	14.44	14.43	-1.15 (0.27)	-1.31 (0.21)	-0.74 (0.47)	-0.84 (0.41)	-0.19 (0.85)	-2.07 (0.06)	-2.24 (0.04)
Left inferior parietal cortex	48.10 (7.38)	48.05 (7.57)	47.97 (7.59)	47.94 (7.52)	48.06 (7.65)	48.21 (7.84)	48.31 (7.33)	48.05 (7.59)	15.35	15.75	15.83	15.68	15.93	16.27	15.17	15.8	0.24 (0.81)	0.17 (0.87)	0.85 (0.41)	-0.52 (0.61)	-0.43 (0.68)	1.29 (0.22)	0.32 (0.76)
Left inferior temporal gyrus	35.51 (5.18)	35.38 (5.35)	35.61 (5.42)	35.44 (5.20)	35.61 (5.23)	35.56 (5.35)	35.48 (5.27)	35.43 (5.49)	14.58	15.12	15.23	14.66	14.69	15.06	14.86	15.5	0.65 (0.52)	0.55 (0.59)	0.36 (0.72)	0.24 (0.81)	-0.22 (0.83)	0.29 (0.77)	0.46 (0.65)
Left isthmus cingulate cortex	10.67 (1.72)	10.75 (1.89)	10.73 (1.80)	10.68 (1.87)	10.73 (1.92)	10.77 (1.79)	10.73 (1.80)	10.68 (1.74)	16.10	17.57	16.78	17.48	17.87	16.6	16.73	16.25	-0.72 (0.48)	0.46 (0.65)	-0.15 (0.88)	-0.53 (0.60)	-1.54 (0.14)	0.52 (0.61)	-0.12 (0.90)
Left lateral occipital cortex	52.89 (9.37)	53.15 (9.52)	52.67 (9.05)	53.10 (9.17)	52.75 (9.41)	52.82 (9.28)	52.73 (9.25)	53.20 (9.33)	17.71	17.91	17.19	17.27	17.84	17.56	17.54	17.54	-1.19 (0.25)	-2.20 (0.04)	-0.84 (0.41)	-0.28 (0.79)	0.26 (0.80)	-2.34 (0.03)	-1.70 (0.11)
Left lateral orbitofrontal cortex	27.74 (3.67)	27.46 (3.68)	27.89 (3.45)	27.66 (3.55)	27.74 (3.75)	27.50 (3.63)	27.94 (3.74)	27.54 (3.53)	13.23	13.41	12.39	12.84	13.52	13.21	13.4	12.83	1.09 (0.29)	1.21 (0.24)	0.31 (0.76)	0.93 (0.37)	0.98 (0.34)	1.57 (0.14)	0.72 (0.48)
Left lingual gyrus	32.10 (4.53)	32.40 (4.75)	32.28 (4.76)	32.54 (4.66)	32.32 (4.71)	32.39 (4.85)	32.37 (4.83)	32.52 (4.86)	14.10	14.65	14.74	14.32	14.59	14.96	14.92	14.95	-1.40 (0.18)	-1.86 (0.08)	-3.55 (0.00)	-0.44 (0.67)	-1.44 (0.17)	-1.00 (0.33)	-2.44 (0.03)
Left medial orbitofrontal cortex	19.04 (3.23)	19.22 (3.14)	18.59 (2.16)	19.23 (2.73)	18.81 (3.12)	19.10 (2.85)	19.20 (3.21)	19.39 (3.24)	16.98	16.35	11.62	14.18	16.6	14.9	16.71	16.7	-0.41 (0.68)	-1.30 (0.21)	-0.37 (0.71)	-0.57 (0.58)	-0.10 (0.92)	-0.47 (0.64)	-0.63 (0.54)
Left middle temporal gyrus	32.69 (3.38)	32.70 (3.36)	32.74 (3.21)	32.76 (3.46)	32.82 (3.32)	32.92 (3.40)	32.82 (3.53)	32.88 (3.33)	10.34	10.26	9.81	10.56	10.12	10.33	10.75	10.11	-0.09 (0.93)	-0.07 (0.94)	-0.34 (0.74)	-0.58 (0.57)	-1.29 (0.22)	-0.29 (0.78)	-0.91 (0.38)
Left parahippocampal gyrus	6.97 (1.16)	6.89 (1.11)	6.94 (1.19)	6.94 (1.19)	6.89 (1.27)	6.95 (1.18)	6.97 (1.20)	6.89 (1.08)	16.65	16.08	17.17	17.09	18.51	16.97	17.24	15.64	1.20 (0.25)	-0.06 (0.95)	0.34 (0.74)	-0.88 (0.39)	0.30 (0.77)	0.99 (0.34)	1.08 (0.30)
Left paracentral lobule	14.06 (2.35)	14.01 (2.30)	14.11 (2.38)	14.05 (2.44)	14.07 (2.39)	14.09 (2.39)	14.02 (2.35)	14.08 (2.39)	16.74	16.45	16.87	17.4	17	16.94	16.73	16.99	0.68 (0.51)	0.89 (0.39)	0.10 (0.92)	-0.35 (0.73)	-0.61 (0.55)	-0.68 (0.51)	-0.34 (0.74)
Left pars opercularis	17.21 (2.88)	16.95 (2.74)	16.96 (2.86)	17.04 (2.69)	16.98 (2.92)	17.00 (2.75)	16.97 (2.72)	16.97 (2.64)	16.76	16.19	16.83	15.78	17.18	16.19	16	15.58	2.09 (0.05)	-0.54 (0.60)	1.10 (0.29)	-0.19 (0.85)	1.71 (0.11)	0.01 (0.99)	1.83 (0.09)
Left pars orbitalis	7.33 (1.17)	7.43 (1.06)	7.35 (1.14)	7.40 (1.10)	7.36 (1.18)	7.33 (1.16)	7.37 (1.27)	7.44 (1.14)	16.01	14.30	15.53	14.94	16.07	15.79	17.21	15.34	-1.14 (0.27)	-0.71 (0.49)	-0.89 (0.39)	0.54 (0.60)	0.14 (0.89)	-0.73 (0.48)	-1.44 (0.17)
Left pars triangularis	14.32 (3.12)	14.31 (3.10)	14.40 (3.09)	14.31 (3.09)	14.34 (3.21)	14.34 (3.06)	14.45 (3.08)	14.24 (3.12)	21.79	21.63	21.49	21.61	22.38	21.37	21.31	21.91	0.06 (0.95)	0.84 (0.41)	0.11 (0.91)	-0.03 (0.97)	-0.26 (0.80)	1.76 (0.10)	0.83 (0.42)
Left pericalcarine cortex	15.15 (2.94)	15.12 (2.87)	15.22 (2.97)	15.35 (2.96)	15.21 (2.95)	15.27 (2.95)	15.21 (3.03)	15.15 (2.82)	19.42	18.99	19.54	19.25	19.42	19.33	19.91	18.64	0.23 (0.82)	-1.09 (0.29)	-1.90 (0.08)	-0.66 (0.52)	-1.28 (0.22)	0.43 (0.67)	0.01 (0.99)
Left postcentral gyrus	42.60 (5.03)	42.68 (5.34)	42.74 (5.11)	42.54 (5.12)	42.66 (5.12)	42.52 (5.04)	42.72 (5.12)	42.55 (5.23)	11.82	12.52	11.95	12.03	12.01	11.85	11.99	12.29	-0.55 (0.59)	1.47 (0.16)	0.54 (0.59)	1.37 (0.19)	0.96 (0.35)	1.14 (0.27)	0.52 (0.61)
Left posterior cingulate cortex	12.75 (2.23)	12.76 (2.24)	12.75 (2.14)	12.75 (2.21)	12.76 (2.19)	12.77 (2.23)	12.80 (2.25)	12.75 (2.21)	17.47	17.59	16.83	17.33	17.16	17.42	17.55	17.3	-0.05 (0.96)	-0.10 (0.92)	-0.01 (0.99)	-0.11 (0.91)	-0.17 (0.87)	0.50 (0.62)	0.00 (1.00)
Left precentral gyrus	48.01 (5.09)	48.28 (5.09)	48.19 (5.16)	48.15 (5.38)	48.06 (5.10)	48.08 (5.32)	48.06 (5.18)	48.08 (5.32)	10.60	10.54	10.7	11.17	10.61	11.06	10.77	11.07	-2.02 (0.06)	0.44 (0.67)	-1.10 (0.29)	-0.14 (0.89)	-0.50 (0.63)	-0.12 (0.91)	-0.46 (0.65)
Left precuneus	39.54 (4.46)	39.20 (4.56)	39.34 (4.59)	39.20 (4.68)	39.41 (4.66)	39.14 (4.64)	39.51 (4.60)	39.35 (4.79)	11.27	11.64	11.67	11.95	11.82	11.87	11.64	12.16	2.48 (0.03)	1.32 (0.21)	2.31 (0.04)	2.14 (0.05)	3.66 (0.00)	1.18 (0.26)	1.22 (0.24)
Left rostral anterior cingulate cortex	9.29 (1.87)	9.28 (1.96)	9.23 (1.87)	9.42 (1.82)	9.46 (1.78)	9.36 (1.79)	9.45 (1.85)	9.30 (1.78)	20.17	21.08	20.27	19.27	18.83	19.16	19.61	19.11	0.08 (0.93)	-1.09 (0.29)	-0.92 (0.37)	0.78 (0.45)	-0.81 (0.43)	1.72 (0.11)	-0.16 (0.87)
Left rostral middle frontal gyrus	58.22 (9.29)	57.71 (9.19)	58.57 (9.29)	57.88 (9.15)	58.51 (9.11)	57.84 (9.30)	58.86 (9.36)	58.34 (9.10)	15.96	15.92	15.86	15.8	15.57	16.08	15.91	15.6	1.22 (0.24)	1.50 (0.16)	0.89 (0.39)	1.79 (0.09)	1.07 (0.30)	1.57 (0.14)	-0.40 (0.70)

Left superior frontal gyrus	77.07 (9.71)	77.00 (9.52)	77.19 (9.63)	76.98 (9.20)	76.90 (9.82)	76.59 (9.24)	77.26 (9.64)	77.05 (9.20)	12.60	12.37	12.48	11.95	12.77	12.06	12.47	11.94	0.22 (0.83)	0.65 (0.53)	0.30 (0.77)	0.94 (0.36)	1.75 (0.10)	0.65 (0.53)	0.07 (0.95)
Left superior parietal cortex	58.92 (6.15)	58.97 (6.18)	58.80 (6.18)	59.08 (6.22)	58.96 (6.23)	58.85 (6.33)	58.90 (6.38)	58.80 (6.02)	10.44	10.49	10.52	10.53	10.56	10.76	10.84	10.23	-0.43 (0.67)	-2.32 (0.03)	-1.24 (0.23)	0.68 (0.51)	0.46 (0.66)	0.69 (0.50)	1.06 (0.31)
Left superior temporal gyrus	38.37 (4.67)	38.66 (4.81)	38.68 (4.80)	38.59 (4.72)	38.54 (4.71)	38.57 (4.75)	38.70 (4.79)	38.66 (4.63)	12.17	12.44	12.4	12.23	12.21	12.32	12.39	11.98	-2.37 (0.03)	0.91 (0.37)	-1.53 (0.15)	-0.26 (0.80)	-1.58 (0.14)	0.25 (0.81)	-1.82 (0.09)
Left supramarginal gyrus	42.86 (6.42)	42.48 (6.28)	42.73 (6.34)	42.65 (6.36)	42.62 (6.39)	42.68 (6.27)	42.75 (6.54)	42.75 (6.30)	14.98	14.78	14.83	14.91	15	14.69	15.3	14.74	1.85 (0.08)	0.59 (0.56)	0.91 (0.38)	-0.37 (0.72)	1.10 (0.29)	0.01 (0.99)	0.66 (0.52)
Left frontal pole	2.68 (0.20)	2.61 (0.25)	2.68 (0.26)	2.66 (0.21)	2.65 (0.22)	2.65 (0.29)	2.72 (0.25)	2.67 (0.26)	7.27	9.52	9.53	8.09	8.4	10.75	9.21	9.65	1.34 (0.20)	0.50 (0.63)	0.59 (0.57)	-0.07 (0.95)	0.50 (0.63)	1.16 (0.26)	0.33 (0.75)
Left temporal pole	4.84 (0.44)	4.82 (0.36)	4.90 (0.37)	4.93 (0.35)	4.83 (0.34)	4.88 (0.37)	4.90 (0.36)	4.85 (0.40)	9.06	7.47	7.61	7.13	7.09	7.66	7.4	8.23	0.26 (0.80)	-0.33 (0.75)	-1.29 (0.22)	-0.73 (0.48)	-0.55 (0.59)	0.57 (0.58)	-0.20 (0.85)
Left transverse temporal gyrus	4.46 (0.59)	4.51 (0.68)	4.47 (0.59)	4.45 (0.59)	4.48 (0.67)	4.45 (0.59)	4.48 (0.60)	4.49 (0.61)	13.23	15.05	13.16	13.36	14.86	13.33	13.32	13.69	-0.93 (0.37)	0.68 (0.51)	0.64 (0.53)	0.80 (0.44)	0.45 (0.66)	-0.49 (0.63)	-1.36 (0.19)
Left insula	24.63 (3.08)	24.65 (1.89)	25.19 (4.27)	24.73 (2.98)	25.50 (3.38)	25.01 (3.00)	25.14 (3.10)	24.70 (2.54)	12.50	7.67	16.95	12.06	13.24	11.99	12.34	10.27	-0.03 (0.97)	0.87 (0.40)	-0.14 (0.89)	0.81 (0.43)	-0.64 (0.53)	0.84 (0.41)	-0.12 (0.91)
Right banks superior temporal sulcus	9.18 (1.21)	9.23 (1.39)	9.23 (1.25)	9.30 (1.32)	9.34 (1.33)	9.06 (1.39)	9.28 (1.48)	9.21 (1.42)	13.13	15.02	13.57	14.24	14.26	15.34	15.95	15.41	-0.71 (0.49)	-0.89 (0.39)	-1.75 (0.10)	3.03 (0.01)	1.21 (0.24)	0.86 (0.41)	-0.25 (0.81)
Right caudal anterior cingulate cortex	8.28 (2.09)	8.14 (1.78)	8.24 (2.01)	8.15 (1.77)	8.28 (2.01)	8.19 (1.86)	8.32 (2.13)	8.23 (1.91)	25.24	21.82	24.38	21.76	24.31	22.77	25.63	23.25	1.06 (0.31)	0.61 (0.55)	0.67 (0.51)	0.99 (0.34)	0.75 (0.46)	0.62 (0.54)	0.37 (0.72)
Right caudal middle frontal gyrus	23.13 (3.24)	23.02 (3.17)	23.11 (3.32)	22.92 (3.28)	23.14 (3.11)	23.18 (3.17)	23.06 (3.20)	23.14 (3.14)	14.03	13.76	14.38	14.3	13.42	13.69	13.89	13.55	0.77 (0.45)	1.80 (0.09)	1.26 (0.23)	-0.22 (0.83)	-0.28 (0.78)	-0.55 (0.59)	-0.11 (0.91)
Right cuneus	17.63 (3.02)	17.70 (3.34)	17.72 (3.20)	17.70 (3.24)	17.47 (2.92)	17.64 (3.30)	17.47 (3.02)	17.72 (3.27)	17.12	18.86	18.06	18.29	16.72	18.7	17.27	18.43	-0.68 (0.51)	0.13 (0.90)	-0.78 (0.45)	-1.18 (0.25)	-0.11 (0.92)	-2.34 (0.03)	-0.76 (0.46)
Right entorhinal cortex	4.12 (0.84)	4.05 (0.93)	4.03 (0.92)	3.99 (1.06)	4.07 (0.89)	4.08 (0.89)	4.08 (0.92)	4.18 (1.00)	20.40	22.87	22.84	26.6	21.93	21.82	22.51	23.99	0.60 (0.56)	0.24 (0.81)	0.89 (0.39)	-0.17 (0.87)	0.32 (0.76)	-1.03 (0.32)	-0.55 (0.59)
Right fusiform gyrus	31.00 (6.02)	31.23 (6.15)	30.94 (5.76)	31.14 (5.99)	30.98 (5.54)	31.18 (6.16)	31.11 (6.04)	31.01 (5.93)	19.43	19.71	18.61	19.25	17.89	19.76	19.42	19.12	-1.54 (0.14)	-0.95 (0.36)	-0.71 (0.49)	-0.96 (0.35)	-1.21 (0.25)	0.64 (0.53)	-0.07 (0.95)
Right inferior parietal cortex	55.65 (7.98)	55.29 (8.15)	55.67 (7.94)	55.26 (8.21)	55.56 (8.24)	55.49 (8.14)	55.35 (8.06)	55.59 (8.17)	14.34	14.73	14.26	14.86	14.84	14.68	14.56	14.69	1.42 (0.18)	1.21 (0.25)	1.45 (0.17)	0.18 (0.86)	0.48 (0.64)	-0.97 (0.35)	0.36 (0.72)
Right inferior temporal gyrus	33.69 (4.38)	33.78 (3.84)	33.78 (4.36)	33.97 (4.18)	33.80 (4.26)	33.82 (4.12)	33.54 (4.31)	33.80 (4.04)	13.00	11.37	12.9	12.29	12.62	12.18	12.86	11.96	-0.37 (0.71)	-1.13 (0.28)	-1.57 (0.14)	-0.05 (0.96)	-0.55 (0.59)	-1.12 (0.28)	-0.48 (0.64)
Right isthmus cingulate cortex	9.66 (1.80)	9.75 (1.88)	9.71 (2.08)	9.72 (2.00)	9.74 (1.96)	9.63 (1.94)	9.76 (2.06)	9.77 (2.12)	18.67	19.30	21.38	20.58	20.13	20.17	21.06	21.71	-1.17 (0.26)	-0.13 (0.90)	-0.78 (0.45)	1.39 (0.19)	0.35 (0.73)	-0.04 (0.97)	-1.10 (0.29)
Right lateral occipital cortex	52.98 (11.06)	52.73 (11.63)	52.79 (10.92)	52.79 (11.23)	52.87 (11.21)	52.51 (10.68)	52.80 (11.02)	52.55 (10.77)	20.87	22.05	20.68	21.27	21.21	20.34	20.88	20.49	1.11 (0.28)	0.02 (0.98)	1.15 (0.27)	1.39 (0.19)	1.55 (0.14)	0.77 (0.45)	1.63 (0.12)
Right lateral orbitofrontal cortex	28.24 (3.86)	28.37 (4.30)	29.09 (4.30)	28.78 (3.80)	28.04 (4.01)	28.60 (4.39)	28.63 (3.27)	28.49 (3.99)	13.67	15.14	14.77	13.21	14.29	15.35	11.43	14.02	-0.27 (0.79)	1.19 (0.25)	-1.26 (0.23)	-1.09 (0.29)	-0.81 (0.43)	0.26 (0.80)	-0.55 (0.59)
Right lingual gyrus	34.07 (5.11)	34.06 (5.14)	34.03 (5.18)	34.33 (5.32)	34.05 (5.17)	34.15 (5.16)	34.04 (4.98)	34.28 (5.37)	14.98	15.09	15.21	15.51	15.2	15.12	14.64	15.65	0.08 (0.94)	-2.01 (0.06)	-1.44 (0.17)	-0.77 (0.46)	-0.56 (0.58)	-1.05 (0.31)	-1.00 (0.33)
Right medial orbitofrontal cortex	20.64 (2.52)	20.75 (2.80)	20.24 (2.53)	20.65 (2.75)	20.62 (2.77)	20.79 (2.45)	20.86 (2.77)	20.49 (2.62)	12.21	13.51	12.5	13.3	13.44	11.77	13.27	12.78	-0.46 (0.65)	-1.86 (0.08)	-0.09 (0.93)	-0.50 (0.63)	-0.54 (0.59)	1.28 (0.22)	0.73 (0.48)
Right middle temporal gyrus	35.50 (3.93)	35.58 (3.93)	35.52 (3.94)	35.51 (3.86)	35.35 (4.10)	35.55 (3.85)	35.50 (3.97)	35.57 (3.75)	11.06	11.04	11.09	10.86	11.59	10.82	11.18	10.55	-0.56 (0.58)	0.00 (1.00)	-0.08 (0.94)	-0.97 (0.35)	-0.35 (0.73)	-0.55 (0.59)	-0.73 (0.48)
Right parahippocampal gyrus	6.29 (1.22)	6.41 (1.21)	6.34 (1.05)	6.40 (1.15)	6.37 (1.26)	6.35 (1.17)	6.38 (1.15)	6.44 (1.20)	19.33	18.91	16.64	17.94	19.74	18.46	18.06	18.57	-2.31 (0.04)	-0.74 (0.47)	-2.03 (0.06)	0.22 (0.83)	-0.92 (0.37)	-1.10 (0.29)	-2.75 (0.01)
Right paracentral lobule	15.83 (2.08)	15.81 (2.16)	15.88 (2.23)	15.81 (2.09)	15.84 (2.10)	15.86 (2.09)	15.84 (2.05)	15.81 (2.08)	13.13	13.68	14.03	13.25	13.23	13.19	12.96	13.17	0.16 (0.87)	0.58 (0.57)	0.21 (0.83)	-0.21 (0.84)	-0.24 (0.81)	0.31 (0.76)	0.17 (0.86)
Right pars opercularis	15.47 (2.01)	15.53 (1.95)	15.51 (1.90)	15.47 (1.77)	15.31 (1.95)	15.49 (1.82)	15.52 (2.00)	15.34 (2.10)	13.02	12.55	12.26	11.46	12.76	11.76	12.89	13.67	-0.63 (0.54)	0.32 (0.75)	0.03 (0.98)	-1.71 (0.11)	-0.16 (0.88)	1.00 (0.33)	1.15 (0.27)
Right pars orbitalis	8.90 (1.43)	8.89 (1.37)	8.90 (1.43)	8.96 (1.43)	8.90 (1.54)	8.94 (1.49)	8.90 (1.52)	8.95 (1.42)	16.01	15.44	16.12	15.95	17.27	16.67	17.1	15.91	0.16 (0.88)	-0.89 (0.39)	-0.90 (0.38)	-0.63 (0.54)	-0.55 (0.59)	-0.90 (0.38)	-0.65 (0.53)
Right pars triangularis	16.86 (2.91)	16.78 (2.88)	16.89 (3.11)	16.81 (3.06)	17.00 (3.05)	16.73 (2.85)	16.97 (2.98)	16.88 (2.84)	17.23	17.18	18.41	18.2	17.92	17.04	17.59	16.81	0.86 (0.40)	0.89 (0.39)	0.61 (0.55)	2.72 (0.02)	1.27 (0.22)	0.91 (0.38)	-0.26 (0.80)
Right pericalcarine cortex	17.05 (3.07)	17.14 (3.23)	17.10 (3.14)	17.15 (3.22)	16.85 (3.09)	17.17 (3.25)	16.95 (3.02)	17.05 (3.14)	17.99	18.81	18.39	18.78	18.36	18.95	17.81	18.41	-0.75 (0.46)	-0.53 (0.61)	-0.83 (0.42)	-2.46 (0.03)	-0.97 (0.35)	-0.96 (0.35)	0.00 (1.00)
Right postcentral gyrus	40.58 (3.23)	40.48 (3.08)	40.74 (3.08)	40.68 (3.18)	40.70 (3.24)	40.61 (3.18)	40.57 (3.24)	40.55 (3.02)	7.97	7.62	7.57	7.81	7.97	7.83	7.98	7.44	0.65 (0.53)	0.56 (0.59)	-0.85 (0.41)	0.87 (0.40)	-0.26 (0.80)	0.12 (0.91)	0.17 (0.87)
Right posterior cingulate cortex	13.17 (1.94)	13.19 (1.97)	13.20 (2.04)	13.10 (2.05)	13.11 (1.98)	13.06 (2.01)	13.22 (2.04)	13.12 (2.05)	14.77	14.94	15.49	15.62	15.09	15.43	15.42	15.6	-0.29 (0.77)	0.90 (0.38)	0.94 (0.36)	0.69 (0.50)	1.80 (0.09)	1.28 (0.22)	0.68 (0.51)
Right precentral gyrus	49.48 (5.68)	49.55 (5.80)	49.54 (5.90)	49.50 (5.89)	49.60 (5.89)	49.52 (5.74)	49.72 (5.70)	49.75 (5.96)	11.48	11.71	11.9	11.91	11.87	11.6	11.47	11.99	-0.49 (0.63)	0.26 (0.80)	-0.12 (0.90)	0.70 (0.50)	-0.22 (0.83)	-0.21 (0.84)	-1.46 (0.16)
Right precuneus	41.22 (5.10)	41.04 (5.10)	41.08 (4.98)	41.08 (5.08)	41.16 (4.90)	41.11 (5.07)	41.01 (4.98)	41.12 (4.96)	12.36	12.43	12.13	12.36	11.92	12.33	12.14	12.06	1.12 (0.28)	-0.04 (0.97)	1.42 (0.18)	0.37 (0.72)	0.83 (0.42)	-0.85 (0.41)	0.71 (0.49)
Right rostral anterior cingulate cortex	6.90 (1.47)	6.93 (1.43)	6.94 (1.58)	6.88 (1.34)	6.84 (1.45)	6.85 (1.37)	6.91 (1.52)	6.92 (1.36)	21.35	20.66	22.8	19.41	21.25	19.97	22.01	19.67	-0.47 (0.65)	0.47 (0.65)	0.19 (0.85)	-0.15 (0.88)	0.65 (0.52)	-0.13 (0.90)	-0.19 (0.85)
Right rostral middle frontal gyrus	59.95 (8.74)	59.76 (7.96)	59.99 (8.53)	59.82 (7.82)	59.79 (8.79)	59.41 (8.11)	60.16 (8.69)	59.85 (8.10)	14.59	13.33	14.22	13.08	14.71	13.66	14.45	13.54	0.60 (0.56)	0.56 (0.58)	0.38 (0.71)	1.15 (0.27)	1.69 (0.11)	0.95 (0.36)	0.32 (0.75)
Right superior frontal gyrus	74.04 (8.84)	74.09 (9.12)	74.03 (8.69)	74.07 (8.92)	73.80 (8.93)	73.92 (8.91)	74.00 (8.76)	74.09 (8.76)	11.95	12.31	11.74	12.05	12.1	12.06	11.84	11.82	-0.28 (0.78)	-0.13 (0.90)	-0.13 (0.90)	-0.77 (0.45)	0.63 (0.54)	-0.48 (0.64)	-0.29 (0.78)
Right superior parietal cortex	54.85 (5.56)	55.19 (5.59)	54.99 (5.39)	55.30 (5.72)	55.09 (5.21)	55.15 (5.69)	55.34 (5.44)	55.18 (5.66)	10.13	10.13	9.8	10.35	9.46	10.32	9.83	10.25	-1.99 (0.07)	-1.62 (0.13)	-2.23 (0.04)	-0.19 (0.85)	-1.62 (0.13)	1.31 (0.21)	-1.75 (0.10)
Right superior temporal gyrus	37.23 (4.42)	37.12 (4.22)	37.42 (4.35)	37.19 (4.30)	37.19 (4.19)	37.26 (4.20)	37.43 (4.34)	37.25 (4.35)	11.88	11.37	11.62	11.57	11.27	11.27	11.59	11.67	1.00 (0.33)	2.25 (0.04)	0.49 (0.63)	-0.49 (0.63)	-0.23 (0.82)	1.67 (0.11)	-0.17 (0.87)
Right supramarginal gyrus	38.99 (6.10)	39.02 (5.94)	38.90 (5.93)	38.67 (5.90)	38.90 (5.97)	38.87 (6.12)	38.94 (6.04)	38.85 (5.89)	15.64	15.22	15.24	15.27	15.34	15.75	15.51	15.16	-0.27 (0.79)	1.60 (0.13)	2.15 (0.05)	0.15 (0.88)	1.46 (0.16)	0.71 (0.49)	1.29 (0.22)
Right frontal pole	3.34 (0.33)	3.31 (0.37)	3.41 (0.42)	3.35 (0.40)	3.32 (0.37)	3.35 (0.38)	3.35 (0.39)	3.27 (0.34)	9.80	11.25	12.24	11.81	11.25	11.47	11.62	10.35	0.77 (0.45)	1.06 (0.30)	-0.20 (0.84)	-0.49 (0.63)	-0.30 (0.77)	1.26 (0.23)	1.62 (0.13)
Right temporal pole	4.70 (0.44)	4.60 (0.48)	4.69 (0.54)	4.59 (0.54)	4.58 (0.47)	4.60 (0.54)	4.64 (0.55)	4.54 (0.53)	9.30	10.38	11.56	11.68	10.15	11.79	11.85	11.67	1.34 (0.20)	1.10 (0.29)	1.91 (0.08)	-0.25 (0.81)	1.51 (0.15)	1.13 (0.28)	2.18 (0.05)

Right transverse temporal gyrus	3.35 (0.45)	3.34 (0.45)	3.33 (0.51)	3.38 (0.43)	3.34 (0.41)	3.37 (0.42)	3.35 (0.45)	3.32 (0.47)	13.37	13.34	15.36	12.76	12.39	12.52	13.49	14.23	0.26 (0.80)	-1.26 (0.23)	-0.91 (0.38)	-1.15 (0.27)	-0.76 (0.46)	0.68 (0.51)	0.88 (0.39)
Right insula	23.57 (3.22)	23.24 (2.71)	22.86 (2.71)	23.01 (3.19)	23.64 (3.56)	22.92 (2.64)	23.15 (2.99)	24.06 (3.61)	13.67	11.64	11.86	13.87	15.08	11.51	12.91	15.01	0.92 (0.37)	-0.36 (0.73)	1.67 (0.12)	1.30 (0.21)	1.44 (0.17)	-1.86 (0.08)	-1.54 (0.15)
Local cortical thickness (mm)																							
Left banks superior temporal sulcus	2.61 (0.14)	2.61 (0.15)	2.61 (0.14)	2.61 (0.15)	2.62 (0.14)	2.60 (0.15)	2.62 (0.14)	2.63 (0.15)	5.49	5.63	5.41	5.83	5.51	5.81	5.48	5.63	-0.10 (0.92)	-0.29 (0.78)	-0.07 (0.94)	0.71 (0.49)	0.48 (0.64)	-0.70 (0.50)	-1.19 (0.25)
Left caudal anterior cingulate cortex	2.56 (0.16)	2.54 (0.21)	2.56 (0.19)	2.53 (0.22)	2.57 (0.20)	2.52 (0.23)	2.55 (0.20)	2.53 (0.23)	6.13	8.29	7.46	8.89	7.77	8.94	7.88	9.2	0.62 (0.54)	1.21 (0.24)	0.92 (0.37)	1.54 (0.14)	1.00 (0.33)	0.46 (0.65)	0.60 (0.56)
Left caudal middle frontal gyrus	2.43 (0.09)	2.41 (0.10)	2.43 (0.10)	2.40 (0.09)	2.43 (0.10)	2.40 (0.11)	2.42 (0.09)	2.41 (0.08)	3.66	4.06	4.23	3.78	4.32	4.56	3.78	3.3	1.20 (0.25)	1.53 (0.15)	1.65 (0.12)	1.93 (0.07)	1.35 (0.20)	0.73 (0.48)	0.84 (0.41)
Left cuneus	1.97 (0.11)	1.97 (0.10)	1.96 (0.10)	1.96 (0.10)	1.96 (0.11)	1.97 (0.11)	1.96 (0.11)	1.96 (0.11)	5.83	4.94	5.22	5.3	5.67	5.37	5.61	5.54	-0.51 (0.62)	-0.14 (0.89)	0.43 (0.67)	-0.43 (0.68)	-0.19 (0.85)	-0.79 (0.44)	0.11 (0.91)
Left entorhinal cortex	3.47 (0.34)	3.51 (0.35)	3.45 (0.34)	3.49 (0.36)	3.47 (0.35)	3.52 (0.35)	3.48 (0.36)	3.48 (0.40)	9.80	10.10	9.91	10.25	10.04	9.81	10.23	11.38	-1.05 (0.31)	-0.83 (0.42)	-0.88 (0.39)	-1.18 (0.26)	-1.54 (0.14)	-0.07 (0.95)	-0.48 (0.64)
Left fusiform gyrus	2.84 (0.12)	2.85 (0.13)	2.84 (0.12)	2.86 (0.13)	2.84 (0.12)	2.86 (0.12)	2.84 (0.12)	2.86 (0.12)	4.34	4.44	4.4	4.71	4.13	4.19	4.13	4.3	-1.14 (0.27)	-1.35 (0.20)	-1.39 (0.19)	-1.37 (0.19)	-1.48 (0.16)	-1.19 (0.25)	-1.28 (0.22)
Left inferior parietal cortex	2.53 (0.08)	2.53 (0.06)	2.52 (0.08)	2.54 (0.07)	2.53 (0.07)	2.53 (0.06)	2.53 (0.08)	2.54 (0.07)	2.98	2.29	3.12	2.74	2.9	2.55	2.97	2.6	-0.28 (0.78)	-1.53 (0.15)	-1.17 (0.26)	0.16 (0.88)	-0.19 (0.86)	-1.96 (0.07)	-1.69 (0.11)
Left inferior temporal gyrus	2.74 (0.13)	2.75 (0.15)	2.73 (0.14)	2.76 (0.15)	2.73 (0.12)	2.76 (0.14)	2.73 (0.14)	2.77 (0.14)	4.69	5.55	5.26	5.3	4.43	5.12	5.21	5.1	-0.89 (0.39)	-2.31 (0.04)	-1.65 (0.12)	-1.41 (0.18)	-1.46 (0.17)	-2.39 (0.03)	-2.15 (0.05)
Left isthmus cingulate cortex	2.53 (0.21)	2.54 (0.22)	2.51 (0.20)	2.55 (0.24)	2.55 (0.22)	2.53 (0.19)	2.52 (0.19)	2.55 (0.24)	8.40	8.70	8.07	9.26	8.53	7.53	7.71	9.28	-0.57 (0.58)	-1.68 (0.11)	-1.07 (0.30)	1.26 (0.23)	-0.05 (0.96)	-1.36 (0.19)	-0.76 (0.46)
Left lateral occipital cortex	2.25 (0.08)	2.24 (0.09)	2.26 (0.08)	2.24 (0.08)	2.25 (0.08)	2.24 (0.08)	2.25 (0.08)	2.25 (0.09)	3.39	3.89	3.64	3.75	3.56	3.64	3.73	3.91	1.36 (0.19)	1.97 (0.07)	1.97 (0.07)	1.66 (0.12)	1.48 (0.16)	0.42 (0.68)	-0.10 (0.92)
Left lateral orbitofrontal cortex	2.60 (0.12)	2.62 (0.10)	2.59 (0.11)	2.61 (0.11)	2.60 (0.10)	2.62 (0.10)	2.60 (0.12)	2.63 (0.10)	4.44	3.99	4.42	4.28	3.88	3.88	4.49	3.94	-1.33 (0.20)	-0.99 (0.34)	-0.60 (0.56)	-0.97 (0.35)	-0.85 (0.41)	-2.01 (0.06)	-1.87 (0.08)
Left lingual gyrus	2.07 (0.12)	2.05 (0.14)	2.07 (0.12)	2.04 (0.12)	2.07 (0.12)	2.05 (0.13)	2.06 (0.11)	2.04 (0.13)	5.70	6.81	5.67	6.02	6.04	6.49	5.44	6.19	1.36 (0.19)	2.09 (0.05)	2.08 (0.06)	1.48 (0.16)	1.42 (0.18)	1.30 (0.21)	1.87 (0.08)
Left medial orbitofrontal cortex	2.39 (0.10)	2.37 (0.10)	2.37 (0.11)	2.36 (0.10)	2.38 (0.08)	2.36 (0.10)	2.38 (0.09)	2.38 (0.11)	4.05	4.08	4.75	4.37	3.45	4.2	3.79	4.48	1.22 (0.24)	0.21 (0.84)	1.63 (0.12)	1.14 (0.27)	1.52 (0.15)	0.13 (0.90)	0.56 (0.58)
Left middle temporal gyrus	2.80 (0.11)	2.80 (0.12)	2.80 (0.11)	2.80 (0.11)	2.80 (0.10)	2.81 (0.10)	2.80 (0.10)	2.80 (0.12)	4.02	4.20	4.1	3.89	3.53	3.43	3.65	4.2	-0.33 (0.75)	-0.22 (0.83)	-0.42 (0.68)	-0.40 (0.70)	-0.53 (0.60)	-0.09 (0.93)	-0.17 (0.86)
Left parahippocampal gyrus	3.03 (0.24)	3.05 (0.23)	3.02 (0.23)	3.03 (0.25)	3.05 (0.25)	3.05 (0.22)	3.04 (0.24)	3.05 (0.22)	7.97	7.46	7.49	8.29	8.21	7.36	7.87	7.32	-1.09 (0.29)	-0.43 (0.67)	0.15 (0.88)	0.28 (0.78)	-0.73 (0.48)	-0.74 (0.47)	-0.74 (0.47)
Left paracentral lobule	2.50 (0.10)	2.50 (0.11)	2.50 (0.12)	2.49 (0.13)	2.51 (0.12)	2.50 (0.14)	2.51 (0.12)	2.51 (0.13)	4.11	4.49	4.84	5.22	4.95	5.59	4.67	5.2	0.28 (0.78)	0.26 (0.80)	0.54 (0.60)	0.39 (0.71)	-0.09 (0.93)	0.03 (0.97)	-0.60 (0.56)
Left pars opercularis	2.51 (0.11)	2.50 (0.12)	2.50 (0.11)	2.51 (0.12)	2.51 (0.11)	2.51 (0.11)	2.51 (0.10)	2.51 (0.12)	4.39	4.88	4.49	4.67	4.46	4.42	4	4.87	0.84 (0.42)	-0.46 (0.65)	0.26 (0.80)	0.49 (0.63)	0.41 (0.69)	0.18 (0.86)	0.14 (0.89)
Left pars orbitalis	2.62 (0.14)	2.59 (0.15)	2.62 (0.14)	2.59 (0.13)	2.61 (0.13)	2.58 (0.13)	2.61 (0.13)	2.61 (0.14)	5.34	5.61	5.51	4.92	4.95	5.16	5.15	5.29	1.24 (0.23)	1.47 (0.16)	1.14 (0.27)	1.39 (0.18)	1.81 (0.09)	-0.31 (0.76)	0.03 (0.97)
Left pars triangularis	2.39 (0.13)	2.38 (0.14)	2.39 (0.14)	2.40 (0.14)	2.39 (0.13)	2.39 (0.13)	2.40 (0.13)	2.41 (0.14)	5.44	5.75	5.9	5.75	5.3	5.49	5.45	5.87	0.85 (0.41)	-1.08 (0.30)	-0.53 (0.61)	-0.51 (0.62)	-0.18 (0.86)	-1.05 (0.31)	-1.26 (0.23)
Left pericalcarine cortex	1.64 (0.12)	1.63 (0.12)	1.64 (0.12)	1.62 (0.12)	1.65 (0.12)	1.62 (0.12)	1.63 (0.14)	1.61 (0.13)	7.05	7.27	7.49	7.59	7.26	7.53	8.37	8.02	0.44 (0.66)	0.71 (0.49)	0.90 (0.38)	1.76 (0.10)	1.36 (0.19)	0.93 (0.37)	2.01 (0.06)
Left postcentral gyrus	2.12 (0.09)	2.11 (0.10)	2.11 (0.09)	2.11 (0.09)	2.12 (0.09)	2.12 (0.11)	2.11 (0.08)	2.12 (0.09)	4.34	4.64	4.04	4.33	4.22	5.01	3.9	4.45	0.70 (0.49)	-0.14 (0.89)	0.95 (0.36)	0.43 (0.68)	0.24 (0.81)	-0.32 (0.76)	0.32 (0.75)
Left posterior cingulate cortex	2.52 (0.11)	2.51 (0.13)	2.53 (0.11)	2.52 (0.12)	2.53 (0.11)	2.52 (0.12)	2.52 (0.11)	2.52 (0.10)	4.37	5.00	4.49	4.82	4.37	4.77	4.44	3.8	0.34 (0.74)	0.34 (0.74)	-0.26 (0.80)	1.04 (0.32)	0.18 (0.86)	0.65 (0.52)	0.27 (0.79)
Left precentral gyrus	2.58 (0.11)	2.58 (0.09)	2.58 (0.10)	2.58 (0.09)	2.59 (0.09)	2.59 (0.10)	2.58 (0.09)	2.59 (0.07)	4.08	3.42	3.82	3.33	3.33	3.83	3.52	2.83	-0.12 (0.90)	0.26 (0.80)	0.71 (0.49)	0.37 (0.72)	-0.26 (0.80)	-0.63 (0.54)	-0.71 (0.49)
Left precuneus	2.51 (0.07)	2.50 (0.08)	2.51 (0.07)	2.51 (0.08)	2.52 (0.08)	2.50 (0.09)	2.51 (0.07)	2.51 (0.08)	2.96	3.09	2.76	3.24	3.16	3.58	2.93	3.02	0.68 (0.51)	0.04 (0.97)	0.20 (0.85)	1.79 (0.09)	0.88 (0.39)	0.55 (0.59)	0.20 (0.85)
Left rostral anterior cingulate cortex	2.88 (0.25)	2.85 (0.27)	2.89 (0.25)	2.86 (0.24)	2.87 (0.23)	2.88 (0.24)	2.86 (0.22)	2.86 (0.25)	8.65	9.37	8.71	8.53	8.11	8.43	7.61	8.66	0.90 (0.38)	1.11 (0.29)	0.69 (0.50)	-0.40 (0.69)	0.02 (0.98)	0.10 (0.92)	0.62 (0.55)
Left rostral middle frontal gyrus	2.24 (0.09)	2.23 (0.11)	2.23 (0.09)	2.23 (0.10)	2.23 (0.08)	2.22 (0.10)	2.23 (0.09)	2.23 (0.11)	3.96	4.77	4.02	4.66	3.79	4.68	3.94	4.82	1.07 (0.30)	0.03 (0.97)	0.76 (0.46)	1.26 (0.23)	1.86 (0.08)	0.58 (0.57)	0.92 (0.37)
Left superior frontal gyrus	2.62 (0.09)	2.58 (0.09)	2.62 (0.11)	2.59 (0.10)	2.61 (0.09)	2.58 (0.10)	2.62 (0.11)	2.60 (0.10)	3.43	3.66	4.35	3.84	3.43	3.95	4.21	3.68	2.15 (0.05)	1.90 (0.08)	2.06 (0.06)	2.13 (0.05)	2.31 (0.04)	1.18 (0.25)	1.29 (0.22)
Left superior parietal cortex	2.23 (0.05)	2.23 (0.07)	2.23 (0.06)	2.23 (0.07)	2.23 (0.06)	2.23 (0.08)	2.24 (0.05)	2.23 (0.07)	2.16	3.11	2.46	3.06	2.57	3.42	2.33	3.03	0.57 (0.58)	0.83 (0.42)	1.18 (0.26)	0.72 (0.48)	0.66 (0.52)	0.95 (0.36)	0.75 (0.46)
Left superior temporal gyrus	2.82 (0.12)	2.81 (0.15)	2.81 (0.12)	2.81 (0.15)	2.82 (0.11)	2.81 (0.15)	2.82 (0.12)	2.82 (0.15)	4.30	5.39	4.25	5.5	3.94	5.51	4.37	5.43	0.11 (0.92)	-0.03 (0.97)	0.17 (0.87)	0.44 (0.67)	0.39 (0.70)	-0.32 (0.75)	-0.37 (0.72)
Left supramarginal gyrus	2.54 (0.10)	2.55 (0.09)	2.54 (0.08)	2.54 (0.09)	2.55 (0.09)	2.55 (0.09)	2.54 (0.09)	2.55 (0.08)	3.81	3.36	3.32	3.37	3.48	3.44	3.41	3.29	-0.58 (0.57)	-0.44 (0.67)	-0.72 (0.48)	0.13 (0.90)	-0.66 (0.52)	-1.32 (0.21)	-1.15 (0.27)
Left frontal pole	2.74 (0.28)	2.75 (0.22)	2.73 (0.23)	2.72 (0.21)	2.68 (0.20)	2.68 (0.20)	2.74 (0.26)	2.73 (0.20)	10.22	8.01	8.61	7.82	7.28	7.49	9.39	7.5	-0.52 (0.61)	0.31 (0.76)	0.50 (0.62)	-0.09 (0.93)	0.88 (0.39)	0.46 (0.65)	0.28 (0.79)
Left temporal pole	3.75 (0.23)	3.77 (0.22)	3.73 (0.24)	3.75 (0.23)	3.74 (0.22)	3.77 (0.25)	3.75 (0.20)	3.79 (0.22)	6.13	5.84	6.42	6.05	5.87	6.66	5.27	5.88	-0.30 (0.77)	-0.49 (0.63)	0.17 (0.87)	-1.09 (0.29)	-0.39 (0.70)	-0.96 (0.35)	-0.77 (0.45)
Left transverse temporal gyrus	2.67 (0.15)	2.66 (0.15)	2.67 (0.16)	2.67 (0.15)	2.67 (0.13)	2.68 (0.14)	2.67 (0.15)	2.67 (0.15)	5.71	5.55	5.89	5.56	4.92	5.21	5.78	5.7	0.12 (0.91)	-0.30 (0.77)	-0.39 (0.71)	-0.62 (0.54)	-0.58 (0.57)	0.01 (0.99)	-0.02 (0.98)
Left insula	3.11 (0.13)	3.10 (0.10)	3.09 (0.12)	3.11 (0.11)	3.06 (0.16)	3.11 (0.13)	3.07 (0.13)	3.12 (0.10)	4.30	3.17	3.92	3.68	5.15	4.05	4.24	3.2	0.29 (0.78)	-0.82 (0.43)	0.06 (0.95)	-1.42 (0.17)	-0.05 (0.96)	-1.67 (0.12)	-0.40 (0.70)
Right banks superior temporal sulcus	2.61 (0.15)	2.60 (0.15)	2.60 (0.14)	2.61 (0.14)	2.60 (0.15)	2.61 (0.15)	2.59 (0.16)	2.62 (0.14)	5.79	5.74	5.47	5.24	5.77	5.59	6.31	5.33	0.36 (0.72)	-1.12 (0.28)	-0.28 (0.78)	-0.49 (0.63)	0.07 (0.94)	-1.62 (0.13)	-0.79 (0.44)
Right caudal anterior cingulate cortex	2.60 (0.20)	2.54 (0.20)	2.60 (0.21)	2.55 (0.20)	2.57 (0.17)	2.56 (0.21)	2.58 (0.21)	2.53 (0.19)	7.68	7.87	8.04	7.77	6.7	8.32	8.17	7.52	1.95 (0.07)	1.56 (0.14)	1.56 (0.14)	0.35 (0.73)	1.27 (0.22)	1.82 (0.09)	2.46 (0.03)
Right caudal middle frontal gyrus	2.52 (0.09)	2.49 (0.12)	2.53 (0.09)	2.50 (0.12)	2.51 (0.10)	2.49 (0.13)	2.51 (0.10)	2.49 (0.11)	3.47	4.82	3.43	4.86	4	5.08	4.14	4.6	1.12 (0.28)	1.42 (0.18)	1.02 (0.33)	1.16 (0.26)	1.39 (0.18)	0.96 (0.35)	1.36 (0.19)

Right cuneus	2.01 (0.15)	1.99 (0.14)	2.00 (0.14)	1.99 (0.13)	2.01 (0.14)	1.99 (0.14)	2.01 (0.15)	1.99 (0.15)	7.70	7.01	7.21	6.71	6.97	6.94	7.36	7.32	1.41 (0.18)	1.32 (0.21)	1.73 (0.10)	2.18 (0.05)	1.48 (0.16)	3.00 (0.01)	1.84 (0.09)
Right entorhinal cortex	3.63 (0.37)	3.60 (0.30)	3.58 (0.36)	3.64 (0.30)	3.66 (0.34)	3.65 (0.26)	3.65 (0.39)	3.62 (0.29)	10.30	8.44	10.14	8.29	9.4	7.04	10.7	8.15	0.52 (0.61)	-0.87 (0.40)	-0.11 (0.91)	0.32 (0.75)	-0.29 (0.78)	0.45 (0.66)	0.15 (0.88)
Right fusiform gyrus	2.84 (0.13)	2.84 (0.13)	2.84 (0.13)	2.84 (0.13)	2.84 (0.13)	2.84 (0.13)	2.84 (0.14)	2.85 (0.14)	4.55	4.72	4.6	4.44	4.71	4.69	4.91	5.05	-0.23 (0.82)	-0.23 (0.82)	-0.42 (0.68)	0.06 (0.95)	-0.18 (0.86)	-0.76 (0.46)	-0.70 (0.49)
Right inferior parietal cortex	2.43 (0.06)	2.43 (0.08)	2.42 (0.08)	2.43 (0.08)	2.42 (0.06)	2.43 (0.08)	2.42 (0.07)	2.44 (0.08)	2.66	3.09	3.11	3.1	2.66	3.18	3.07	3.35	0.08 (0.94)	-0.76 (0.46)	0.03 (0.98)	-0.30 (0.77)	0.32 (0.75)	-1.69 (0.11)	-0.99 (0.34)
Right inferior temporal gyrus	2.74 (0.12)	2.75 (0.12)	2.74 (0.11)	2.75 (0.10)	2.73 (0.11)	2.74 (0.12)	2.74 (0.12)	2.75 (0.12)	4.29	4.34	3.99	3.77	4	4.25	4.52	4.27	-0.53 (0.61)	-0.36 (0.72)	-0.41 (0.68)	-0.45 (0.66)	0.25 (0.80)	-0.57 (0.58)	-0.51 (0.62)
Right isthmus cingulate cortex	2.55 (0.16)	2.55 (0.16)	2.56 (0.14)	2.55 (0.14)	2.55 (0.15)	2.57 (0.16)	2.55 (0.14)	2.55 (0.17)	6.29	6.40	5.56	5.39	5.79	6.41	5.58	6.53	-0.24 (0.81)	0.66 (0.52)	-0.08 (0.93)	-1.29 (0.22)	-1.05 (0.31)	0.01 (0.99)	-0.13 (0.90)
Right lateral occipital cortex	2.29 (0.09)	2.29 (0.08)	2.28 (0.08)	2.29 (0.08)	2.28 (0.08)	2.30 (0.08)	2.28 (0.08)	2.30 (0.08)	3.75	3.65	3.55	3.35	3.44	3.46	3.59	3.51	-0.49 (0.63)	-0.81 (0.43)	-0.62 (0.55)	-1.50 (0.16)	-0.98 (0.34)	-2.44 (0.03)	-1.96 (0.07)
Right lateral orbitofrontal cortex	2.64 (0.11)	2.64 (0.13)	2.63 (0.09)	2.62 (0.14)	2.63 (0.10)	2.63 (0.15)	2.62 (0.12)	2.64 (0.10)	4.14	5.10	3.54	5.29	3.84	5.57	4.56	3.91	0.24 (0.81)	0.08 (0.93)	0.70 (0.49)	-0.08 (0.94)	0.35 (0.73)	-1.00 (0.33)	0.16 (0.87)
Right lingual gyrus	2.13 (0.09)	2.11 (0.11)	2.12 (0.08)	2.10 (0.11)	2.12 (0.09)	2.11 (0.11)	2.12 (0.08)	2.11 (0.11)	4.07	5.29	3.89	5.04	4.2	5.05	3.91	5.2	1.10 (0.29)	1.14 (0.27)	2.02 (0.06)	1.03 (0.32)	1.45 (0.17)	1.14 (0.27)	1.50 (0.15)
Right medial orbitofrontal cortex	2.57 (0.08)	2.56 (0.10)	2.56 (0.06)	2.56 (0.09)	2.55 (0.09)	2.53 (0.11)	2.54 (0.09)	2.57 (0.08)	3.26	3.96	2.49	3.58	3.64	4.25	3.39	2.93	0.75 (0.46)	0.19 (0.85)	0.86 (0.40)	0.77 (0.45)	2.13 (0.05)	-1.19 (0.25)	0.55 (0.59)
Right middle temporal gyrus	2.86 (0.13)	2.87 (0.11)	2.86 (0.12)	2.87 (0.12)	2.84 (0.12)	2.85 (0.12)	2.83 (0.13)	2.86 (0.11)	4.61	3.84	4.33	4.11	4.27	4.23	4.51	3.8	-0.60 (0.56)	-0.71 (0.49)	-0.80 (0.43)	-0.14 (0.89)	0.58 (0.57)	-2.00 (0.06)	-0.26 (0.80)
Right parahippocampal gyrus	2.99 (0.15)	3.01 (0.14)	2.99 (0.15)	3.01 (0.14)	3.01 (0.13)	3.01 (0.14)	3.00 (0.14)	3.01 (0.16)	4.90	4.63	4.91	4.61	4.43	4.68	4.71	5.43	-0.93 (0.37)	-1.59 (0.13)	-1.02 (0.32)	-0.24 (0.82)	-0.89 (0.39)	-0.04 (0.97)	-0.60 (0.56)
Right paracentral lobule	2.49 (0.13)	2.47 (0.11)	2.48 (0.13)	2.47 (0.13)	2.49 (0.12)	2.48 (0.14)	2.48 (0.12)	2.47 (0.12)	5.42	4.62	5.09	5.11	4.96	5.59	4.91	5.01	0.80 (0.44)	0.63 (0.54)	0.97 (0.35)	1.23 (0.24)	0.73 (0.48)	0.58 (0.57)	0.86 (0.40)
Right pars opercularis	2.61 (0.12)	2.61 (0.13)	2.61 (0.11)	2.60 (0.12)	2.59 (0.12)	2.60 (0.13)	2.59 (0.12)	2.61 (0.12)	4.44	4.84	4.32	4.49	4.57	4.82	4.47	4.49	0.63 (0.54)	0.56 (0.58)	0.64 (0.53)	-0.67 (0.52)	1.08 (0.30)	-0.89 (0.39)	0.71 (0.49)
Right pars orbitalis	2.67 (0.15)	2.68 (0.16)	2.67 (0.13)	2.67 (0.17)	2.66 (0.15)	2.66 (0.15)	2.66 (0.15)	2.69 (0.16)	5.71	6.08	5.03	6.47	5.59	5.82	5.82	5.97	-0.71 (0.49)	-0.24 (0.81)	-0.49 (0.63)	0.13 (0.90)	0.42 (0.68)	-1.45 (0.17)	-1.56 (0.14)
Right pars triangularis	2.49 (0.11)	2.48 (0.11)	2.49 (0.10)	2.49 (0.11)	2.48 (0.10)	2.49 (0.11)	2.49 (0.11)	2.50 (0.11)	4.26	4.25	4.21	4.44	4.01	4.53	4.22	4.37	0.46 (0.65)	-0.09 (0.93)	0.08 (0.94)	-0.23 (0.82)	0.38 (0.71)	-0.54 (0.60)	-0.20 (0.85)
Right pericalcarine cortex	1.67 (0.15)	1.65 (0.16)	1.67 (0.15)	1.65 (0.17)	1.68 (0.15)	1.66 (0.16)	1.68 (0.16)	1.66 (0.16)	8.99	9.42	9.01	10.1	9.19	9.52	9.51	9.84	1.12 (0.28)	1.04 (0.31)	0.86 (0.41)	1.11 (0.28)	0.75 (0.47)	1.03 (0.32)	0.44 (0.66)
Right postcentral gyrus	2.10 (0.08)	2.10 (0.09)	2.09 (0.09)	2.10 (0.10)	2.09 (0.09)	2.10 (0.10)	2.09 (0.09)	2.10 (0.09)	3.97	4.39	4.28	4.63	4.07	4.58	4.46	4.44	-0.17 (0.86)	-1.77 (0.10)	-0.76 (0.46)	-1.17 (0.26)	-0.55 (0.59)	-0.90 (0.38)	0.01 (0.99)
Right posterior cingulate cortex	2.46 (0.12)	2.46 (0.15)	2.47 (0.12)	2.47 (0.13)	2.46 (0.11)	2.48 (0.14)	2.46 (0.11)	2.47 (0.14)	4.70	6.18	4.69	5.43	4.48	5.5	4.66	5.48	-0.23 (0.82)	-0.08 (0.93)	-0.93 (0.37)	-0.69 (0.50)	-1.40 (0.18)	-0.46 (0.65)	-0.71 (0.49)
Right precentral gyrus	2.59 (0.11)	2.59 (0.10)	2.59 (0.09)	2.59 (0.09)	2.58 (0.09)	2.58 (0.10)	2.57 (0.10)	2.59 (0.09)	4.15	3.69	3.61	3.56	3.55	4.02	3.92	3.56	0.14 (0.89)	0.16 (0.87)	0.11 (0.91)	-0.11 (0.92)	0.38 (0.71)	-0.75 (0.47)	0.29 (0.77)
Right precuneus	2.49 (0.08)	2.49 (0.09)	2.47 (0.07)	2.49 (0.09)	2.48 (0.07)	2.48 (0.09)	2.48 (0.07)	2.49 (0.09)	3.11	3.81	2.64	3.79	2.91	3.64	2.71	3.72	-0.09 (0.93)	-1.41 (0.18)	-0.29 (0.78)	-0.57 (0.58)	0.17 (0.87)	-0.62 (0.54)	0.07 (0.94)
Right rostral anterior cingulate cortex	2.98 (0.15)	2.98 (0.14)	2.97 (0.15)	2.97 (0.14)	2.99 (0.14)	2.98 (0.14)	2.95 (0.16)	2.96 (0.14)	5.06	4.81	5	4.84	4.61	4.86	5.53	4.73	0.27 (0.79)	-0.09 (0.93)	0.50 (0.62)	0.35 (0.73)	0.14 (0.89)	-0.44 (0.67)	0.77 (0.45)
Right rostral middle frontal gyrus	2.36 (0.05)	2.34 (0.07)	2.36 (0.05)	2.34 (0.07)	2.35 (0.05)	2.33 (0.06)	2.35 (0.06)	2.34 (0.07)	2.29	3.21	2.06	3.18	2.02	2.65	2.48	2.86	1.06 (0.30)	1.23 (0.24)	1.07 (0.30)	1.16 (0.27)	1.77 (0.10)	0.51 (0.62)	1.11 (0.28)
Right superior frontal gyrus	2.72 (0.09)	2.70 (0.11)	2.73 (0.09)	2.70 (0.11)	2.71 (0.09)	2.68 (0.12)	2.71 (0.11)	2.70 (0.10)	3.43	3.93	3.2	4.06	3.38	4.52	3.88	3.82	1.25 (0.23)	1.30 (0.21)	1.12 (0.28)	1.92 (0.07)	1.97 (0.07)	0.31 (0.76)	1.15 (0.27)
Right superior parietal cortex	2.20 (0.07)	2.19 (0.08)	2.19 (0.07)	2.19 (0.09)	2.19 (0.07)	2.19 (0.09)	2.19 (0.06)	2.20 (0.09)	3.32	3.72	3.33	4.04	3.23	4.13	2.97	3.92	0.42 (0.68)	-0.48 (0.64)	0.39 (0.70)	0.61 (0.55)	1.41 (0.18)	-1.16 (0.27)	0.05 (0.96)
Right superior temporal gyrus	2.95 (0.12)	2.93 (0.15)	2.94 (0.12)	2.93 (0.13)	2.93 (0.13)	2.92 (0.14)	2.93 (0.14)	2.94 (0.15)	4.14	5.16	4.12	4.54	4.35	4.71	4.78	5.08	1.12 (0.28)	0.82 (0.43)	1.50 (0.15)	1.29 (0.22)	1.90 (0.08)	-0.44 (0.67)	0.49 (0.63)
Right supramarginal gyrus	2.51 (0.08)	2.50 (0.08)	2.50 (0.08)	2.50 (0.09)	2.50 (0.08)	2.49 (0.09)	2.49 (0.08)	2.51 (0.09)	3.12	3.32	3.07	3.61	3.22	3.56	3.21	3.48	0.55 (0.59)	-0.44 (0.67)	0.28 (0.78)	0.63 (0.54)	1.03 (0.32)	-1.41 (0.18)	-0.33 (0.75)
Right frontal pole	2.66 (0.13)	2.72 (0.16)	2.68 (0.14)	2.69 (0.15)	2.60 (0.16)	2.62 (0.18)	2.67 (0.14)	2.68 (0.16)	4.70	6.02	5.23	5.69	6.08	7.04	5.42	6.06	-1.50 (0.15)	-0.35 (0.73)	-0.71 (0.49)	-0.55 (0.59)	0.86 (0.40)	-0.28 (0.79)	-0.55 (0.59)
Right temporal pole	3.83 (0.28)	3.91 (0.19)	3.81 (0.31)	3.91 (0.20)	3.82 (0.30)	3.89 (0.18)	3.83 (0.31)	3.93 (0.18)	7.26	4.77	8.06	5.05	7.81	4.7	8.08	4.65	-1.70 (0.11)	-1.80 (0.09)	-1.41 (0.18)	-1.18 (0.25)	-1.04 (0.32)	-1.60 (0.13)	-1.66 (0.12)
Right transverse temporal gyrus	2.74 (0.21)	2.72 (0.22)	2.73 (0.20)	2.70 (0.22)	2.75 (0.21)	2.70 (0.22)	2.74 (0.20)	2.72 (0.22)	7.65	7.96	7.48	7.97	7.7	8.22	7.31	8.14	0.79 (0.44)	1.21 (0.25)	1.56 (0.14)	1.74 (0.10)	1.49 (0.16)	1.07 (0.30)	1.02 (0.32)
Right insula	3.14 (0.16)	3.17 (0.12)	3.16 (0.14)	3.18 (0.12)	3.13 (0.15)	3.18 (0.10)	3.14 (0.09)	3.11 (0.17)	4.97	3.77	4.43	3.72	4.65	3.01	2.77	5.52	-0.79 (0.44)	-0.74 (0.47)	-1.29 (0.22)	-1.59 (0.13)	-1.11 (0.29)	0.81 (0.43)	0.89 (0.39)
Global brain measures																						
Left total cortical volume (ml)	250.57 (21.45)	249.88 (22.55)	250.33 (21.43)	249.79 (21.86)	250.59 (22.81)	249.56 (23.16)	250.78 (21.99)	250.65 (22.36)	8.56	9.02	8.56	8.75	9.1	9.28	8.77	8.92	0.70 (0.50)	0.67 (0.51)	0.86 (0.40)	1.11 (0.29)	1.04 (0.31)	0.14 (0.89)	-0.09 (0.93)
Right total cortical volume (ml)	252.63 (22.32)	251.89 (22.92)	252.52 (22.69)	251.83 (21.53)	251.69 (22.47)	250.94 (22.40)	251.84 (21.50)	252.12 (21.96)	8.83	9.10	8.98	8.55	8.93	8.93	8.54	8.71	0.57 (0.58)	0.65 (0.53)	0.70 (0.50)	0.72 (0.48)	1.43 (0.17)	-0.25 (0.81)	0.42 (0.68)
Left total cortical white matter (ml)	227.42 (34.18)	227.83 (35.14)	227.87 (34.01)	228.27 (34.94)	227.63 (34.79)	228.16 (35.76)	228.08 (34.45)	228.53 (35.60)	15.03	15.42	14.92	15.31	15.29	15.67	15.1	15.58	-0.62 (0.54)	-1.03 (0.32)	-1.78 (0.09)	-1.02 (0.32)	-1.08 (0.30)	-1.08 (0.30)	-1.89 (0.08)
Right total cortical white matter (ml)	228.37 (35.52)	228.82 (36.23)	228.69 (35.53)	229.20 (35.77)	228.07 (35.46)	228.92 (36.03)	228.44 (35.33)	229.19 (36.30)	15.56	15.83	15.54	15.61	15.55	15.74	15.47	15.84	-0.65 (0.52)	-0.85 (0.41)	-1.25 (0.23)	-1.44 (0.17)	-0.93 (0.37)	-1.30 (0.21)	-1.19 (0.25)
Left total cortical surface area (cm²)	894.23 (95.31)	893.60 (95.58)	894.59 (95.92)	894.44 (94.55)	895.06 (97.01)	893.83 (95.83)	896.96 (96.45)	895.53 (95.71)	10.66	10.70	10.72	10.57	10.84	10.72	10.75	10.69	0.43 (0.67)	0.09 (0.93)	-0.11 (0.91)	0.81 (0.43)	0.26 (0.80)	0.89 (0.39)	-0.80 (0.44)
Right total cortical surface area (cm²)	895.33 (96.45)	894.90 (97.05)	895.73 (96.95)	895.28 (95.81)	894.40 (97.20)	894.00 (96.15)	895.88 (95.86)	896.15 (96.22)	10.77	10.84	10.82	10.7	10.87	10.76	10.7	10.74	0.39 (0.70)	0.30 (0.77)	0.05 (0.96)	0.33 (0.74)	1.31 (0.21)	-0.26 (0.80)	-0.98 (0.34)
Left mean cortical thickness (mm)	2.49 (0.06)	2.49 (0.06)	2.49 (0.06)	2.49 (0.06)	2.49 (0.05)	2.49 (0.06)	2.49 (0.06)	2.49 (0.06)	2.26	2.50	2.49	2.45	2.13	2.42	2.32	2.3	0.81 (0.43)	0.55 (0.59)	1.04 (0.32)	0.86 (0.40)	0.81 (0.43)	-0.34 (0.74)	-0.08 (0.93)
Right mean cortical tickness (mm)	2.52 (0.05)	2.51 (0.06)	2.52 (0.05)	2.51 (0.06)	2.51 (0.05)	2.51 (0.06)	2.51 (0.06)	2.52 (0.06)	2.08	2.45	1.89	2.42	1.87	2.39	2.25	2.47	0.67 (0.52)	0.21 (0.84)	0.65 (0.52)	0.39 (0.70)	1.22 (0.24)	-0.79 (0.44)	0.41 (0.69)

Left cerebellar white matter (ml)	15.25 (1.48)	15.15 (1.66)	15.14 (1.59)	15.00 (1.57)	15.21 (1.59)	14.98 (1.53)	15.17 (1.68)	15.05 (1.65)	9.73	10.98	10.49	10.45	10.47	10.22	11.08	10.96	0.58 (0.57)	0.80 (0.43)	1.41 (0.18)	1.37 (0.19)	1.68 (0.11)	0.70 (0.49)	1.30 (0.21)
Right cerebellar white matter (ml)	14.48 (1.58)	14.42 (1.85)	14.24 (1.64)	14.39 (1.65)	14.38 (1.62)	14.39 (1.69)	14.37 (1.55)	14.34 (1.74)	10.94	12.82	11.5	11.48	11.28	11.78	10.79	12.11	0.34 (0.74)	-1.17 (0.26)	0.73 (0.48)	-0.11 (0.92)	0.70 (0.50)	0.21 (0.84)	1.01 (0.33)
Left cerebellar gray matter (ml)	55.82 (5.28)	55.82 (5.20)	55.83 (5.30)	55.83 (5.22)	55.75 (5.32)	55.79 (5.29)	55.82 (5.23)	56.00 (5.24)	9.46	9.32	9.49	9.36	9.53	9.48	9.37	9.36	0.01 (0.99)	-0.02 (0.98)	-0.04 (0.97)	-0.19 (0.85)	0.17 (0.87)	-0.94 (0.36)	-0.94 (0.36)
Right cerebellar gray matter (ml)	54.79 (5.44)	54.78 (5.44)	54.83 (5.50)	54.76 (5.47)	54.61 (5.49)	54.68 (5.44)	54.93 (5.41)	54.96 (5.39)	9.92	9.93	10.03	9.98	10.06	9.96	9.85	9.81	0.12 (0.91)	0.37 (0.72)	0.23 (0.82)	-0.45 (0.66)	0.67 (0.51)	-0.21 (0.84)	-1.18 (0.26)
Intracranial volume (ml)	1484.47 (258.06)	1494.06 (261.63)	1497.06 (242.06)	1495.98 (254.54)	1437.35 (264.02)	1434.48 (284.46)	1516.68 (233.88)	1516.81 (237.82)	17.38	17.51	16.17	17.01	18.37	19.83	15.42	15.68	-1.31 (0.21)	0.10 (0.92)	-1.42 (0.18)	0.22 (0.83)	2.66 (0.02)	-0.03 (0.97)	-2.42 (0.03)
Total brain volume without ventricles (ml)	1158.90 (124.07)	1158.06 (125.74)	1158.88 (123.80)	1158.36 (123.73)	1157.40 (126.10)	1156.51 (127.45)	1158.77 (123.46)	1160.14 (125.34)	10.71	10.86	10.68	10.68	10.89	11.02	10.65	10.8	0.38 (0.71)	0.31 (0.76)	0.27 (0.79)	0.45 (0.66)	0.99 (0.34)	-0.80 (0.44)	-0.57 (0.58)

Abbreviations: SD=standard deviation; Orig = Original scan; FS df =FreeSurfer defacing; FSL df =FSL defacing; FM = Face Masking; Suffix 1 and 2 indicate first and second scan respectively, i.e. test-retest; t=t-statistic; *p*=p-value.

Table S7. Effects of de-identification on test-retest reliability in young adults: Average signed and absolute percentage differences

Brain metric	Signed PD in % (SD)							Absolute PD (%) (SD)						
	Orig1 - Orig2	Orig1 - Orig2	Orig1 - FS df2	FSL df1 - FSL df2	Orig1 - FSL df2	FM1 - FM2	Orig1 - FM2	Orig1 - Orig2	Orig1 - Orig2	Orig1 - FS df2	FSL df1 - FSL df2	Orig1 - FSL df2	FM1 - FM2	Orig1 - FM2
Subcortical volume (ml)														
Left thalamus	0.30 (3.25)	-0.62 (3.54)	-0.97 (3.39)	-1.26 (3.37)	-1.13 (2.29)	-0.20 (3.09)	-0.03 (2.07)	2.44 (2.07)	2.48 (2.53)	2.90 (1.87)	2.52 (2.50)	2.05 (1.46)	1.97 (2.33)	1.68 (1.13)
Left caudatus	-1.29 (2.76)	-0.98 (1.92)	-1.20 (2.35)	-0.27 (1.86)	-0.57 (2.19)	-0.57 (1.63)	-1.36 (2.09)	2.01 (2.25)	1.49 (1.53)	1.79 (1.91)	1.27 (1.35)	1.62 (1.53)	1.21 (1.19)	1.89 (1.59)
Left putamen	-0.03 (4.02)	-0.71 (3.13)	-0.18 (4.44)	-0.48 (3.47)	-0.24 (4.42)	-1.02 (2.42)	-0.61 (4.29)	2.94 (2.64)	2.53 (1.87)	3.17 (3.00)	2.46 (2.42)	3.18 (2.96)	1.89 (1.77)	3.11 (2.91)
Left pallidum	0.20 (5.49)	-2.04 (5.86)	0.09 (5.39)	-1.30 (4.12)	-0.19 (4.66)	-0.93 (6.75)	-0.45 (6.45)	4.12 (3.47)	4.69 (3.91)	4.17 (3.24)	3.04 (2.98)	3.51 (2.93)	5.05 (4.39)	5.19 (3.62)
Left hippocampus	0.57 (1.85)	-0.10 (2.80)	0.13 (2.14)	0.06 (2.37)	-0.07 (2.27)	0.60 (2.53)	1.13 (2.38)	1.40 (1.29)	2.15 (1.71)	1.76 (1.15)	1.94 (1.27)	1.52 (1.64)	1.98 (1.60)	2.14 (1.46)
Left amygdala	0.43 (5.16)	-0.99 (5.75)	0.00 (4.86)	-0.79 (8.56)	-0.24 (7.23)	-0.22 (6.71)	-0.65 (6.88)	4.02 (3.10)	4.50 (3.53)	4.01 (2.55)	6.69 (5.12)	5.03 (5.04)	5.44 (3.66)	5.18 (4.39)
Left accumbens	4.38 (11.00)	0.78 (6.45)	2.46 (9.96)	-0.59 (9.85)	1.01 (8.58)	0.15 (12.90)	4.31 (11.52)	9.02 (7.40)	5.06 (3.86)	7.84 (6.32)	8.83 (3.79)	6.93 (4.83)	9.90 (7.87)	9.37 (7.67)
Right thalamus	-1.23 (2.85)	-1.62 (3.67)	-1.88 (3.31)	-1.90 (3.03)	-1.61 (3.15)	-1.82 (3.36)	-1.57 (3.42)	2.37 (1.94)	2.84 (2.77)	3.08 (2.14)	2.98 (1.89)	3.11 (1.55)	3.21 (1.95)	3.27 (1.70)
Right caudatus	-0.80 (2.10)	-0.33 (2.92)	-1.08 (2.47)	-0.90 (2.17)	-1.01 (1.47)	-1.39 (1.97)	-1.09 (1.86)	1.76 (1.32)	2.16 (1.91)	2.13 (1.58)	1.66 (1.63)	1.47 (0.97)	1.89 (1.47)	1.62 (1.39)
Right putamen	-0.30 (1.69)	-0.32 (1.86)	-0.70 (1.82)	-0.85 (1.95)	-0.54 (1.91)	-0.61 (1.58)	-1.08 (1.61)	1.34 (1.02)	1.52 (1.05)	1.46 (1.25)	1.57 (1.39)	1.48 (1.28)	1.18 (1.18)	1.52 (1.16)
Right pallidum	-0.35 (3.91)	-0.28 (4.25)	0.25 (4.78)	-0.28 (4.16)	-0.21 (4.52)	0.91 (5.59)	-0.02 (5.88)	2.80 (2.66)	3.28 (2.59)	3.62 (3.00)	3.01 (2.77)	3.72 (2.40)	4.63 (3.03)	4.71 (3.31)
Right hippocampus	0.96 (1.69)	-0.17 (3.04)	0.14 (2.25)	-0.13 (2.50)	0.33 (2.24)	0.35 (3.18)	0.48 (2.71)	1.58 (1.09)	2.47 (1.67)	1.74 (1.36)	1.85 (1.62)	1.74 (1.39)	2.50 (1.90)	2.19 (1.57)
Right amygdala	-1.53 (6.49)	1.21 (6.55)	-0.71 (6.30)	2.19 (7.68)	0.31 (6.72)	1.85 (8.35)	-1.13 (7.13)	4.20 (5.08)	4.99 (4.23)	4.42 (4.41)	6.29 (4.68)	4.70 (4.66)	5.93 (5.99)	4.51 (5.52)
Right accumbens	1.08 (10.27)	2.42 (9.15)	2.39 (9.56)	1.07 (6.05)	1.32 (7.72)	1.39 (6.94)	1.15 (8.81)	7.94 (6.28)	7.79 (5.02)	8.32 (4.87)	5.00 (3.35)	6.45 (4.14)	5.93 (3.57)	7.10 (5.01)
Ventricular volume (ml)														
Third ventricle	1.94 (5.38)	1.92 (5.20)	1.38 (4.96)	1.82 (5.67)	0.66 (5.91)	2.52 (5.97)	2.14 (5.09)	3.76 (4.23)	4.19 (3.51)	3.78 (3.38)	4.10 (4.21)	4.23 (4.04)	4.53 (4.54)	3.60 (4.12)
Left lateral ventricle	1.00 (3.20)	0.57 (2.64)	0.53 (3.06)	0.65 (2.57)	1.05 (2.93)	0.74 (2.96)	0.71 (3.05)	2.36 (2.31)	1.93 (1.83)	2.13 (2.20)	1.88 (1.81)	2.27 (2.06)	2.29 (1.94)	2.25 (2.11)
Left inferior lateral ventricle	5.03 (10.82)	6.02 (13.71)	7.03 (14.09)	-0.97 (11.97)	4.76 (15.22)	4.45 (17.34)	4.19 (16.05)	9.57 (6.81)	10.15 (10.81)	11.14 (10.91)	9.70 (6.63)	9.58 (12.56)	10.26 (14.48)	9.83 (13.17)
Right lateral ventricle	1.26 (2.69)	1.73 (2.97)	1.31 (2.90)	1.17 (2.72)	0.80 (2.76)	1.20 (2.73)	1.23 (2.63)	2.20 (1.94)	2.71 (2.05)	2.53 (1.85)	2.01 (2.13)	2.15 (1.84)	2.29 (1.84)	2.29 (1.71)
Right inferior lateral ventricle	3.28 (12.86)	3.27 (12.89)	2.77 (9.58)	-0.67 (13.29)	-1.08 (14.63)	3.34 (17.13)	0.00 (10.00)	9.36 (9.13)	7.38 (10.93)	7.56 (6.23)	9.59 (8.89)	9.78 (10.64)	10.10 (14.02)	7.39 (6.47)
Local cortical volume (ml)														
Left banks superior temporal sulcus	-0.65 (6.27)	0.82 (4.42)	0.02 (4.92)	-1.72 (4.44)	-2.29 (5.40)	1.02 (4.94)	-1.02 (5.10)	4.87 (3.80)	3.85 (2.10)	3.95 (2.76)	3.54 (3.09)	4.49 (3.63)	3.86 (3.10)	4.26 (2.78)
Left caudal anterior cingulate cortex	-3.23 (5.45)	-1.61 (5.95)	-2.77 (5.80)	-2.04 (6.59)	-1.96 (6.44)	-0.45 (4.95)	-0.61 (5.74)	5.11 (3.60)	4.50 (4.07)	4.74 (4.23)	4.92 (4.69)	4.81 (4.57)	3.74 (3.14)	3.80 (4.24)
Left caudal middle frontal gyrus	-0.32 (3.05)	-1.01 (3.42)	-1.46 (3.95)	-1.17 (3.25)	-1.08 (3.67)	-0.69 (2.41)	-0.39 (3.39)	2.54 (1.60)	2.69 (2.26)	3.40 (2.35)	2.76 (1.98)	3.28 (1.81)	1.94 (1.51)	2.76 (1.88)
Left cuneus	-0.25 (4.00)	0.23 (3.65)	-0.44 (3.66)	0.11 (3.21)	0.14 (3.02)	-0.05 (3.06)	0.09 (3.40)	3.24 (2.21)	3.10 (1.78)	3.00 (2.00)	2.60 (1.76)	2.61 (1.38)	2.53 (1.58)	2.57 (2.13)
Left entorhinal cortex	1.23 (10.24)	0.61 (10.73)	-0.82 (10.57)	-1.28 (12.85)	-0.55 (11.64)	-1.83 (10.33)	-1.48 (11.62)	8.85 (4.78)	8.39 (6.35)	8.34 (6.19)	10.57 (6.91)	9.92 (5.54)	8.37 (5.97)	9.93 (5.69)
Left fusiform gyrus	0.95 (2.67)	1.44 (3.23)	0.88 (3.58)	1.44 (3.62)	0.59 (3.45)	1.89 (3.59)	1.40 (3.07)	2.11 (1.82)	2.78 (2.11)	2.84 (2.25)	3.27 (1.98)	2.94 (1.74)	2.89 (2.79)	2.40 (2.31)
Left inferior parietal cortex	-0.07 (2.51)	0.18 (2.35)	-0.26 (1.99)	0.11 (3.00)	0.14 (2.64)	-0.30 (2.54)	0.18 (1.95)	1.95 (1.50)	1.80 (1.44)	1.66 (1.06)	2.37 (1.75)	2.12 (1.47)	1.96 (1.56)	1.44 (1.28)
Left inferior temporal gyrus	-0.49 (4.02)	0.45 (3.51)	-0.01 (3.34)	-0.14 (3.78)	-0.02 (3.95)	0.60 (3.28)	0.05 (3.29)	3.30 (2.19)	2.24 (2.68)	2.57 (2.02)	3.05 (2.09)	2.80 (2.69)	2.93 (1.40)	2.60 (1.90)
Left isthmus cingulate cortex	0.99 (4.27)	-0.25 (2.63)	0.45 (3.58)	-0.03 (3.92)	0.98 (4.44)	-0.10 (4.56)	0.85 (5.13)	3.55 (2.41)	2.21 (1.33)	2.60 (2.41)	2.71 (2.75)	2.82 (3.50)	3.05 (3.31)	3.85 (3.36)
Left lateral occipital cortex	-0.29 (1.68)	-0.64 (2.44)	-0.53 (1.61)	-0.89 (2.88)	-0.83 (2.04)	0.43 (1.67)	0.22 (1.76)	1.20 (1.18)	2.08 (1.33)	1.39 (0.91)	2.42 (1.70)	1.66 (1.39)	1.40 (0.94)	1.43 (0.98)
Left lateral orbitofrontal cortex	-0.57 (3.20)	-0.29 (3.06)	0.16 (3.00)	-0.27 (3.55)	-0.18 (3.28)	0.03 (3.89)	0.42 (4.10)	2.26 (2.26)	2.16 (2.12)	2.22 (1.95)	2.39 (2.57)	2.22 (2.35)	2.74 (2.67)	2.74 (3.00)
Left lingual gyrus	-0.77 (2.73)	-0.72 (3.08)	-0.64 (3.23)	-0.76 (2.29)	-0.50 (2.76)	-0.81 (2.69)	-0.78 (2.70)	2.13 (1.80)	2.47 (1.89)	2.63 (1.88)	1.65 (1.71)	2.15 (1.72)	2.11 (1.78)	2.01 (1.90)
Left medial orbitofrontal cortex	-0.86 (7.18)	1.88 (8.07)	-0.77 (8.11)	0.61 (8.99)	-1.44 (9.74)	0.63 (6.05)	0.57 (8.80)	5.11 (4.95)	6.73 (4.54)	6.68 (4.33)	7.70 (4.24)	8.13 (5.15)	4.71 (3.66)	7.54 (4.14)
Left middle temporal gyrus	0.63 (3.48)	-0.14 (3.03)	0.29 (3.01)	0.40 (2.91)	0.63 (3.17)	0.34 (4.07)	0.23 (5.22)	2.63 (2.27)	2.27 (1.92)	2.34 (1.82)	2.25 (1.81)	2.66 (1.71)	3.22 (2.37)	3.85 (3.39)
Left parahippocampal gyrus	-0.22 (3.57)	-0.31 (4.01)	-0.64 (5.09)	0.25 (4.01)	0.12 (4.69)	-0.96 (5.08)	-0.41 (4.28)	2.79 (2.12)	3.38 (2.00)	4.40 (2.39)	3.38 (2.00)	4.03 (2.18)	3.75 (3.44)	3.33 (2.58)
Left paracentral lobule	-0.66 (2.79)	-0.73 (3.51)	-0.40 (2.71)	-0.33 (2.19)	0.26 (2.93)	0.14 (3.63)	0.33 (3.39)	2.18 (1.79)	3.00 (1.82)	2.23 (1.49)	1.69 (1.36)	2.19 (1.88)	2.77 (2.24)	2.68 (1.99)
Left pars opercularis	-2.15 (3.53)	0.54 (3.46)	-1.33 (3.52)	0.04 (2.87)	-1.52 (3.08)	0.05 (2.42)	-1.57 (3.09)	3.00 (2.79)	3.00 (1.63)	3.04 (2.11)	2.23 (1.72)	2.28 (2.52)	1.68 (1.68)	2.41 (2.45)
Left pars orbitalis	-0.45 (3.89)	-0.77 (4.74)	-0.33 (4.27)	-2.29 (4.48)	-1.99 (3.70)	0.55 (4.17)	0.55 (4.02)	3.16 (2.15)	3.36 (3.32)	3.01 (2.95)	4.04 (2.88)	3.14 (2.72)	3.57 (2.04)	3.46 (1.93)

Left pars triangularis	-0.18 (3.82)	-0.58 (4.74)	0.02 (3.92)	0.31 (3.39)	0.11 (3.34)	-0.81 (3.65)	0.21 (3.53)	3.09 (2.11)	3.82 (2.69)	2.83 (2.61)	2.62 (2.08)	2.56 (2.05)	2.60 (2.61)	2.98 (1.74)
Left pericalcarine cortex	-2.03 (4.98)	-0.82 (6.24)	-0.97 (6.04)	-1.96 (3.76)	-1.65 (4.62)	-2.59 (6.09)	-3.53 (6.57)	4.41 (2.91)	4.90 (3.75)	4.66 (3.79)	3.35 (2.51)	3.71 (3.11)	5.30 (3.77)	5.91 (4.38)
Left postcentral gyrus	-0.34 (2.99)	-0.39 (2.21)	-0.65 (2.15)	-0.47 (2.12)	-0.52 (2.65)	-0.10 (2.07)	-0.39 (2.59)	2.32 (1.82)	1.85 (1.18)	1.90 (1.12)	1.61 (1.39)	2.13 (1.56)	1.61 (1.23)	2.09 (1.48)
Left posterior cingulate cortex	-0.37 (2.46)	-0.92 (2.86)	-0.23 (2.46)	-0.93 (2.48)	-0.51 (2.17)	-0.77 (3.42)	-0.58 (3.02)	2.20 (1.01)	2.48 (1.60)	2.01 (1.35)	2.09 (1.54)	1.80 (1.24)	2.89 (1.85)	2.60 (1.50)
Left precentral gyrus	0.46 (1.75)	-0.30 (1.35)	-0.22 (1.58)	-0.24 (2.44)	0.04 (2.30)	0.00 (2.07)	0.27 (2.22)	1.52 (0.91)	1.08 (0.82)	1.34 (0.80)	2.16 (1.03)	1.83 (1.30)	1.59 (1.27)	1.95 (0.99)
Left precuneus	-1.35 (2.39)	-0.27 (1.83)	-1.16 (2.55)	-1.38 (1.75)	-1.58 (2.48)	-0.42 (2.04)	-0.65 (2.65)	1.87 (1.98)	1.41 (1.14)	2.18 (1.70)	1.86 (1.18)	2.28 (1.81)	1.54 (1.35)	1.93 (1.88)
Left rostral anterior cingulate cortex	-1.23 (4.37)	0.38 (6.22)	0.09 (4.99)	-0.64 (4.51)	0.30 (3.42)	-1.04 (4.24)	-0.27 (3.95)	3.42 (2.87)	4.33 (4.35)	3.52 (3.42)	3.55 (2.71)	2.75 (1.94)	3.46 (2.52)	3.03 (2.42)
Left rostral middle frontal gyrus	-0.88 (2.94)	-0.77 (3.56)	-0.68 (3.25)	-1.30 (3.23)	-1.43 (3.05)	-0.82 (2.89)	-0.46 (2.74)	1.98 (2.30)	2.17 (2.87)	2.24 (2.39)	2.13 (2.72)	2.24 (2.47)	1.92 (2.26)	2.13 (1.70)
Left superior frontal gyrus	-0.78 (2.93)	-1.10 (2.42)	-0.93 (2.65)	-1.11 (3.00)	-1.75 (2.97)	-0.74 (2.90)	-0.49 (3.18)	1.87 (2.35)	1.87 (1.85)	1.89 (2.03)	2.23 (2.24)	2.36 (2.48)	2.13 (2.05)	2.09 (2.39)
Left superior parietal cortex	-0.40 (1.94)	-0.43 (2.27)	-0.50 (1.93)	-0.78 (2.29)	-0.75 (2.49)	-0.65 (2.06)	-0.66 (1.96)	1.58 (1.12)	1.79 (1.39)	1.35 (1.42)	2.13 (1.02)	2.04 (1.54)	1.74 (1.20)	1.43 (1.45)
Left superior temporal gyrus	0.31 (3.09)	-0.27 (2.51)	0.12 (3.43)	-0.40 (2.49)	-0.09 (2.48)	-0.08 (3.61)	0.54 (3.64)	2.23 (2.08)	1.68 (1.84)	2.56 (2.18)	1.74 (1.78)	1.97 (1.41)	2.24 (2.77)	2.45 (2.68)
Left supramarginal gyrus	-0.29 (2.51)	-0.15 (1.64)	-0.12 (2.57)	0.16 (2.53)	0.11 (2.91)	0.59 (2.03)	0.61 (2.74)	2.01 (1.45)	1.31 (0.93)	2.15 (1.29)	1.97 (1.51)	2.35 (1.61)	1.61 (1.31)	2.10 (1.78)
Left frontal pole	-0.65 (8.79)	-0.31 (8.59)	-0.73 (7.39)	1.68 (8.00)	-1.88 (10.42)	-1.83 (6.20)	-1.23 (8.93)	7.00 (5.05)	6.06 (5.89)	5.18 (5.16)	5.88 (5.49)	7.27 (7.47)	5.02 (3.89)	7.16 (5.15)
Left temporal pole	2.46 (7.17)	2.32 (5.31)	3.47 (5.66)	2.58 (5.24)	3.15 (6.70)	2.80 (8.05)	3.39 (7.66)	6.13 (4.23)	5.11 (2.46)	5.77 (3.06)	4.76 (3.23)	5.88 (4.31)	5.85 (6.06)	6.90 (4.50)
Left transverse temporal gyrus	0.69 (5.03)	-0.09 (4.44)	0.00 (4.25)	-0.30 (4.59)	0.15 (4.63)	0.24 (3.82)	0.66 (4.37)	3.85 (3.17)	3.58 (2.45)	3.09 (2.81)	3.77 (2.44)	3.60 (2.77)	3.14 (2.04)	3.13 (3.01)
Left insula	0.55 (5.50)	-0.98 (6.63)	-0.21 (6.22)	-0.70 (7.38)	1.52 (6.62)	0.38 (5.17)	1.04 (6.66)	3.85 (3.84)	4.90 (4.41)	4.64 (3.96)	5.92 (4.18)	5.18 (4.19)	3.46 (3.77)	5.41 (3.78)
Right banks superior temporal sulcus	0.59 (4.90)	1.68 (4.19)	1.69 (4.39)	-3.24 (5.47)	-1.44 (5.99)	0.46 (4.66)	0.60 (5.76)	3.79 (3.01)	3.28 (3.02)	4.13 (2.03)	4.74 (4.14)	4.83 (3.63)	3.95 (2.31)	4.70 (3.15)
Right caudal anterior cingulate cortex	-4.09 (8.40)	-4.68 (11.82)	-4.21 (12.75)	-2.57 (4.52)	-3.17 (8.64)	-4.36 (10.85)	-4.07 (10.14)	6.11 (6.98)	6.60 (10.80)	7.31 (11.16)	3.78 (3.50)	5.37 (7.39)	7.16 (9.13)	6.07 (9.01)
Right caudal middle frontal gyrus	-1.22 (3.76)	-1.41 (3.25)	-1.32 (3.90)	-0.86 (3.73)	-0.84 (4.01)	-0.35 (3.46)	-0.95 (4.09)	3.16 (2.26)	2.81 (2.06)	3.36 (2.25)	2.93 (2.35)	3.38 (2.17)	2.72 (2.06)	3.07 (2.76)
Right cuneus	-0.91 (3.29)	-0.91 (3.19)	-1.09 (3.36)	-1.07 (3.02)	-1.37 (3.53)	-0.05 (2.34)	-0.81 (2.90)	2.45 (2.30)	2.59 (1.98)	2.57 (2.35)	2.49 (1.94)	2.75 (2.54)	1.79 (1.44)	2.53 (1.51)
Right entorhinal cortex	-3.14 (14.91)	-0.94 (19.41)	-5.23 (18.30)	-1.17 (13.40)	-1.91 (14.72)	-0.58 (16.97)	-1.07 (15.93)	12.06 (8.82)	13.97 (13.03)	13.51 (13.01)	10.37 (8.15)	10.52 (10.13)	12.02 (11.59)	12.42 (9.52)
Right fusiform gyrus	0.85 (4.25)	1.44 (4.84)	1.16 (4.56)	0.33 (3.38)	0.92 (3.73)	0.09 (3.50)	0.54 (4.13)	3.44 (2.48)	3.48 (3.56)	3.44 (3.10)	2.63 (2.04)	3.10 (2.14)	2.75 (2.05)	3.26 (2.47)
Right inferior parietal cortex	-0.86 (3.18)	-0.65 (2.59)	-0.93 (2.76)	-0.20 (3.00)	-0.72 (2.82)	0.98 (2.43)	0.12 (2.28)	2.43 (2.15)	1.81 (1.92)	1.95 (2.12)	2.24 (1.92)	2.24 (1.78)	1.61 (2.04)	1.63 (1.54)
Right inferior temporal gyrus	0.56 (3.71)	0.67 (3.76)	0.96 (3.60)	-0.06 (4.87)	-0.23 (4.65)	1.17 (4.11)	0.40 (3.58)	3.06 (2.03)	2.86 (2.42)	3.00 (2.08)	3.54 (3.22)	3.62 (2.77)	3.24 (2.69)	2.83 (2.11)
Right isthmus cingulate cortex	0.55 (5.46)	0.02 (4.09)	0.05 (4.07)	-0.50 (4.34)	0.00 (4.76)	-0.50 (3.60)	0.21 (3.61)	4.44 (3.02)	3.14 (2.49)	3.05 (2.57)	3.11 (2.96)	3.80 (2.69)	2.94 (2.01)	2.87 (2.07)
Right lateral occipital cortex	-0.36 (2.87)	0.11 (2.91)	-0.32 (2.92)	-0.08 (3.79)	-0.43 (3.46)	0.64 (3.31)	-0.01 (3.46)	2.06 (1.96)	2.10 (1.94)	1.93 (2.15)	2.74 (2.53)	2.50 (2.34)	1.93 (2.72)	2.27 (2.54)
Right lateral orbitofrontal cortex	0.44 (4.97)	-0.47 (5.01)	1.42 (4.01)	1.10 (5.25)	0.48 (4.20)	0.01 (5.63)	0.65 (5.62)	3.47 (3.48)	4.08 (2.75)	3.79 (1.72)	4.06 (3.36)	2.96 (2.92)	4.70 (2.84)	4.32 (3.48)
Right lingual gyrus	-0.53 (2.72)	-0.09 (2.44)	-0.35 (2.05)	-0.19 (2.68)	-0.53 (2.08)	-0.36 (3.37)	-0.46 (2.84)	2.26 (1.50)	1.84 (1.54)	1.71 (1.10)	2.15 (1.52)	1.76 (1.15)	2.90 (1.59)	2.39 (1.49)
Right medial orbitofrontal cortex	-0.39 (3.61)	1.47 (3.76)	-0.92 (2.71)	-0.34 (4.60)	-1.41 (3.74)	-1.78 (4.79)	-1.68 (3.97)	2.82 (2.17)	2.99 (2.63)	2.34 (1.55)	3.34 (3.06)	3.09 (2.45)	3.89 (3.20)	3.48 (2.41)
Right middle temporal gyrus	0.28 (2.33)	0.18 (2.57)	0.12 (2.20)	0.29 (3.50)	-0.86 (3.58)	1.10 (2.38)	-0.22 (2.83)	1.96 (1.20)	1.90 (1.66)	1.73 (1.29)	2.58 (2.29)	2.64 (2.48)	2.34 (1.07)	2.39 (1.41)
Right parahippocampal gyrus	1.09 (2.79)	0.15 (3.92)	1.30 (3.11)	-0.48 (5.55)	0.81 (4.16)	0.23 (3.63)	1.93 (2.75)	2.56 (1.44)	2.83 (2.61)	2.34 (2.38)	4.37 (3.27)	3.13 (2.75)	2.59 (2.47)	2.61 (2.06)
Right paracentral lobule	-1.24 (3.77)	-1.17 (3.51)	-1.37 (4.08)	-1.07 (2.43)	-0.63 (3.94)	-1.08 (2.98)	-1.16 (3.44)	3.00 (2.50)	2.62 (2.54)	3.24 (2.73)	1.86 (1.86)	3.15 (2.31)	2.56 (1.76)	2.94 (2.01)
Right pars opercularis	0.47 (2.46)	-0.11 (3.50)	0.28 (3.15)	1.46 (3.24)	-0.01 (2.89)	-0.63 (5.02)	-0.75 (3.83)	2.05 (1.35)	2.77 (2.02)	2.22 (2.18)	2.76 (2.16)	2.30 (1.65)	3.90 (3.06)	3.52 (1.44)
Right pars orbitalis	0.44 (3.88)	0.25 (2.75)	0.47 (3.45)	-0.42 (3.98)	-0.48 (4.54)	1.34 (3.76)	0.92 (4.11)	3.21 (2.05)	2.08 (1.74)	2.82 (1.90)	2.88 (2.68)	3.62 (2.61)	3.07 (2.45)	3.62 (1.96)
Right pars triangularis	-1.05 (3.74)	-0.63 (3.07)	-0.74 (2.86)	-1.03 (2.31)	-1.03 (3.12)	-0.39 (3.55)	-0.12 (3.39)	2.96 (2.41)	2.30 (2.05)	2.22 (1.88)	1.72 (1.82)	2.14 (2.44)	2.65 (2.29)	2.32 (2.40)
Right pericalcarine cortex	-1.73 (5.06)	-1.31 (3.92)	-1.34 (5.51)	-0.43 (4.51)	-0.89 (4.97)	-1.85 (4.36)	-1.23 (4.68)	4.26 (3.07)	2.91 (2.85)	4.24 (3.62)	3.46 (2.78)	3.84 (3.13)	3.45 (3.15)	3.75 (2.92)
Right postcentral gyrus	-0.02 (2.79)	0.27 (1.76)	0.45 (2.14)	0.46 (2.49)	0.43 (2.59)	0.41 (2.52)	0.01 (2.69)	1.99 (1.89)	1.41 (1.04)	1.58 (1.46)	2.12 (1.28)	1.86 (1.80)	1.78 (1.77)	1.98 (1.74)
Right posterior cingulate cortex	-0.17 (2.39)	-0.86 (2.99)	-0.19 (2.28)	-0.76 (2.63)	-0.48 (2.31)	-0.79 (3.34)	-0.34 (2.60)	1.78 (1.53)	2.20 (2.13)	2.01 (0.97)	1.85 (1.98)	1.83 (1.41)	2.67 (2.06)	1.92 (1.72)
Right precentral gyrus	-0.05 (2.97)	-0.23 (2.85)	-0.11 (3.32)	-0.17 (2.62)	-0.29 (2.77)	0.50 (3.13)	0.34 (2.85)	2.25 (1.85)	1.70 (2.26)	2.40 (2.21)	1.92 (1.71)	1.97 (1.90)	2.18 (2.23)	2.11 (1.87)
Right precuneus	-0.73 (2.58)	0.53 (1.86)	-0.32 (2.17)	-0.38 (1.86)	-0.69 (1.31)	0.34 (2.11)	-0.51 (2.88)	1.93 (1.81)	1.36 (1.34)	1.62 (1.42)	1.47 (1.15)	1.08 (0.99)	1.65 (1.28)	2.00 (2.07)
Right rostral anterior cingulate cortex	0.70 (4.82)	-0.39 (7.28)	-0.10 (5.86)	0.11 (4.68)	-0.22 (4.86)	1.06 (4.81)	0.47 (4.19)	3.75 (2.96)	5.41 (4.69)	4.10 (4.05)	3.88 (2.42)	3.80 (2.88)	3.79 (3.00)	3.19 (2.64)
Right rostral middle frontal gyrus	-0.87 (4.40)	-0.88 (3.83)	-0.83 (4.11)	-1.00 (3.14)	-2.06 (3.51)	-0.53 (3.21)	-0.91 (3.79)	3.01 (3.24)	2.75 (2.72)	3.12 (2.69)	2.55 (2.00)	2.68 (3.02)	2.54 (1.92)	2.91 (2.49)
Right superior frontal gyrus	-0.74 (3.09)	-0.72 (3.08)	-0.64 (3.35)	-0.81 (2.22)	-1.56 (3.04)	0.09 (2.71)	-0.49 (2.99)	2.30 (2.12)	2.20 (2.21)	2.31 (2.44)	1.74 (1.55)	2.42 (2.37)	1.78 (2.00)	2.13 (2.08)
Right superior parietal cortex	0.46 (2.64)	0.33 (2.94)	0.46 (2.79)	-0.33 (3.15)	-0.13 (2.53)	0.02 (2.52)	0.44 (3.04)	1.90 (1.82)	2.29 (1.78)	2.08 (1.84)	2.14 (2.26)	1.70 (1.82)	1.71 (1.79)	1.80 (2.45)
Right superior temporal gyrus	-1.29 (3.16)	-1.48 (2.19)	-1.10 (2.46)	-1.08 (3.20)	-1.39 (3.57)	-0.71 (2.01)	-0.49 (2.49)	2.52 (2.24)	2.09 (1.58)	1.96 (1.81)	2.48 (2.22)	2.72 (2.62)	1.72 (1.19)	1.96 (1.53)

Right supramarginal gyrus	0.06 (2.32)	-0.21 (1.64)	-0.72 (1.93)	-0.24 (2.36)	-0.69 (2.00)	0.46 (1.57)	-0.10 (1.97)	1.78 (1.42)	1.36 (0.88)	1.70 (1.09)	1.95 (1.26)	1.64 (1.28)	1.15 (1.14)	1.68 (0.93)
Right frontal pole	-0.30 (4.75)	-2.55 (9.58)	-0.36 (8.41)	0.74 (10.36)	-4.17 (13.09)	-3.08 (7.75)	-3.41 (6.36)	3.64 (2.93)	7.76 (5.88)	5.76 (5.95)	7.32 (7.12)	8.52 (10.61)	6.08 (5.55)	5.02 (5.10)
Right temporal pole	0.56 (9.38)	0.24 (4.57)	0.39 (7.56)	1.68 (9.34)	-0.09 (9.33)	-1.08 (5.33)	-1.78 (7.65)	6.43 (6.65)	3.74 (2.47)	5.09 (5.44)	7.21 (5.90)	6.29 (6.69)	4.49 (2.85)	5.36 (5.58)
Right transverse temporal gyrus	-1.47 (6.00)	0.17 (6.87)	-1.13 (6.04)	-0.85 (6.33)	-1.12 (6.44)	-2.23 (7.11)	-2.23 (5.74)	3.99 (4.62)	5.54 (3.81)	4.64 (3.85)	4.96 (3.82)	4.83 (4.24)	5.89 (4.35)	4.98 (3.44)
Right insula	-0.10 (4.58)	0.52 (5.14)	-0.75 (4.48)	-0.39 (5.17)	-0.92 (4.23)	2.87 (4.65)	1.77 (3.68)	3.80 (2.36)	3.65 (3.53)	3.49 (2.77)	4.03 (3.09)	3.41 (2.53)	4.02 (3.63)	3.01 (2.69)
Local cortical surface (cm^2)														
Left banks superior temporal sulcus	-0.55 (3.40)	0.83 (3.56)	0.21 (3.87)	-0.72 (2.66)	-1.37 (3.60)	1.24 (3.48)	-0.54 (3.66)	2.77 (1.92)	2.61 (2.47)	3.19 (2.06)	2.25 (1.48)	2.86 (2.51)	2.57 (2.59)	3.14 (1.79)
Left caudal anterior cingulate cortex	-1.29 (3.96)	-0.34 (4.40)	-1.32 (4.24)	0.25 (5.31)	0.00 (4.71)	0.75 (2.86)	0.89 (2.40)	3.49 (2.10)	3.78 (2.05)	3.99 (1.68)	4.14 (3.15)	3.89 (2.46)	2.26 (1.84)	1.95 (1.60)
Left caudal middle frontal gyrus	0.54 (1.89)	0.47 (2.10)	0.15 (2.64)	0.08 (2.20)	0.32 (2.27)	-0.14 (1.87)	0.45 (1.90)	1.56 (1.14)	1.57 (1.42)	1.89 (1.78)	1.42 (1.64)	1.70 (1.47)	1.42 (1.18)	1.54 (1.14)
Left cuneus	-0.58 (2.88)	0.32 (2.49)	-0.04 (2.24)	0.31 (3.27)	0.13 (2.96)	-0.11 (2.31)	0.19 (2.10)	2.34 (1.68)	1.80 (1.70)	1.78 (1.28)	2.47 (2.07)	1.85 (2.27)	1.71 (1.49)	1.58 (1.33)
Left entorhinal cortex	0.83 (10.17)	0.85 (8.51)	0.73 (9.73)	-0.50 (8.11)	-0.06 (12.03)	-1.65 (7.52)	-0.64 (8.65)	8.06 (5.91)	6.21 (5.66)	7.94 (5.29)	6.23 (4.97)	10.05 (6.08)	6.47 (3.85)	7.24 (4.39)
Left fusiform gyrus	0.59 (2.05)	0.83 (2.46)	0.54 (2.63)	0.53 (2.63)	0.12 (2.37)	1.20 (2.37)	1.12 (2.07)	1.72 (1.19)	2.24 (1.20)	1.97 (1.75)	2.05 (1.65)	1.86 (1.40)	2.09 (1.57)	1.67 (1.63)
Left inferior parietal cortex	-0.16 (1.72)	-0.02 (1.46)	-0.38 (1.59)	0.29 (2.38)	0.08 (1.97)	-0.62 (1.71)	-0.19 (1.56)	1.33 (1.05)	1.17 (0.82)	1.24 (1.02)	1.59 (1.75)	1.43 (1.31)	1.37 (1.16)	1.26 (0.87)
Left inferior temporal gyrus	-0.43 (2.33)	-0.39 (3.02)	-0.21 (2.20)	-0.20 (2.46)	0.04 (2.26)	-0.25 (2.04)	-0.36 (2.10)	1.75 (1.53)	1.86 (2.36)	1.73 (1.31)	2.01 (1.33)	1.55 (1.60)	1.65 (1.16)	1.70 (1.21)
Left isthmus cingulate cortex	0.51 (3.96)	-0.55 (3.67)	-0.08 (3.14)	0.49 (2.21)	0.86 (2.19)	-0.45 (3.70)	0.10 (4.07)	3.37 (1.97)	2.93 (2.15)	2.33 (2.02)	1.78 (1.33)	1.82 (1.44)	3.15 (1.82)	3.31 (2.23)
Left lateral occipital cortex	0.46 (1.61)	0.80 (1.50)	0.45 (1.83)	0.15 (1.92)	-0.13 (1.84)	0.89 (1.62)	0.62 (1.39)	1.35 (0.94)	1.12 (1.27)	1.55 (1.01)	1.58 (1.04)	1.56 (0.90)	1.36 (1.22)	1.16 (0.95)
Left lateral orbitofrontal cortex	-1.04 (3.64)	-0.86 (2.70)	-0.23 (3.53)	-0.80 (3.47)	-0.85 (3.41)	-1.37 (3.53)	-0.68 (3.96)	3.04 (2.13)	2.16 (1.77)	2.77 (2.09)	2.51 (2.45)	2.84 (1.93)	2.73 (2.55)	3.01 (2.55)
Left lingual gyrus	0.84 (2.54)	0.84 (1.58)	1.32 (1.48)	0.13 (2.01)	0.76 (2.48)	0.43 (1.92)	1.17 (2.22)	2.04 (1.67)	1.54 (0.86)	1.52 (1.26)	1.46 (1.33)	1.81 (1.81)	1.41 (1.33)	1.91 (1.57)
Left medial orbitofrontal cortex	1.04 (8.61)	3.09 (10.36)	1.40 (10.40)	1.76 (10.80)	0.58 (11.91)	0.99 (8.19)	1.89 (11.28)	6.48 (5.52)	9.25 (5.12)	8.46 (5.81)	8.49 (6.56)	9.76 (6.38)	6.54 (4.73)	10.19 (4.49)
Left middle temporal gyrus	0.06 (2.24)	-0.03 (2.25)	0.18 (2.32)	0.28 (2.08)	0.71 (2.20)	0.24 (2.47)	0.59 (2.56)	1.56 (1.56)	1.81 (1.26)	1.76 (1.44)	1.48 (1.44)	1.83 (1.35)	1.88 (1.54)	1.88 (1.77)
Left parahippocampal gyrus	-0.98 (3.62)	0.05 (3.92)	-0.41 (3.96)	1.06 (3.50)	-0.35 (3.94)	-0.93 (4.50)	-0.92 (4.04)	2.97 (2.17)	3.40 (1.74)	3.10 (2.37)	2.62 (2.48)	3.35 (1.91)	3.22 (3.18)	3.09 (2.65)
Left paracentral lobule	-0.33 (1.95)	-0.51 (1.93)	-0.13 (1.62)	0.11 (1.36)	0.17 (1.53)	0.39 (2.58)	0.12 (1.98)	1.57 (1.13)	1.45 (1.32)	1.40 (0.75)	0.97 (0.93)	1.24 (0.85)	2.25 (1.20)	1.71 (0.90)
Left pars opercularis	-1.44 (3.00)	0.61 (3.27)	-0.83 (3.58)	0.24 (1.98)	-1.12 (2.90)	0.06 (2.06)	-1.19 (2.87)	2.50 (2.12)	2.75 (1.75)	2.76 (2.33)	1.57 (1.16)	2.29 (2.03)	1.69 (1.09)	2.21 (2.13)
Left pars orbitalis	1.50 (4.12)	0.76 (4.13)	1.01 (3.58)	-0.38 (3.16)	-0.06 (2.59)	1.12 (4.41)	1.50 (3.77)	3.66 (2.26)	2.66 (3.18)	2.74 (2.43)	2.47 (1.92)	2.07 (1.46)	3.46 (2.83)	3.02 (2.62)
Left pars triangularis	0.01 (2.70)	-0.65 (2.89)	-0.02 (2.67)	0.24 (3.02)	0.26 (2.54)	-1.54 (2.99)	-0.54 (2.37)	2.14 (1.54)	2.23 (1.87)	2.27 (1.29)	2.41 (1.73)	2.02 (1.47)	2.75 (1.85)	1.60 (1.80)
Left pericalcarine cortex	-0.09 (2.92)	0.82 (2.96)	1.34 (2.68)	0.42 (2.65)	0.80 (2.48)	-0.21 (3.74)	0.11 (3.80)	2.39 (1.55)	2.71 (1.29)	2.28 (1.88)	2.02 (1.69)	2.15 (1.39)	2.99 (2.11)	2.91 (2.33)
Left postcentral gyrus	0.13 (1.39)	-0.46 (1.27)	-0.15 (1.01)	-0.32 (1.01)	-0.19 (0.79)	-0.43 (1.34)	-0.17 (0.96)	1.19 (0.67)	0.94 (0.94)	0.88 (0.46)	0.87 (0.56)	0.61 (0.51)	1.08 (0.87)	0.74 (0.61)
Left posterior cingulate cortex	0.04 (3.14)	-0.04 (2.11)	0.03 (2.98)	0.07 (2.64)	0.18 (3.49)	-0.36 (3.28)	0.04 (3.41)	2.63 (1.58)	1.67 (1.23)	2.24 (1.88)	1.94 (1.72)	3.04 (1.56)	2.48 (2.08)	2.72 (1.93)
Left precentral gyrus	0.57 (1.15)	-0.13 (0.77)	0.24 (0.96)	0.00 (1.19)	0.10 (1.10)	0.00 (1.19)	0.09 (1.14)	1.00 (0.78)	0.65 (0.40)	0.71 (0.66)	0.93 (0.71)	0.92 (0.56)	0.88 (0.76)	0.88 (0.69)
Left precuneus	-0.90 (1.41)	-0.40 (1.11)	-0.93 (1.55)	-0.70 (1.34)	-1.08 (1.21)	-0.46 (1.42)	-0.57 (1.60)	1.41 (0.83)	0.94 (0.68)	1.46 (1.02)	1.28 (0.76)	1.28 (0.99)	1.13 (0.94)	1.27 (1.08)
Left rostral anterior cingulate cortex	-0.27 (4.84)	2.25 (7.31)	1.60 (5.93)	-1.13 (5.27)	0.99 (3.76)	-1.43 (3.62)	0.38 (4.16)	3.52 (3.21)	4.75 (5.90)	4.39 (4.16)	4.08 (3.38)	3.17 (2.10)	3.21 (2.08)	3.39 (2.28)
Left rostral middle frontal gyrus	-0.87 (2.94)	-1.15 (3.08)	-0.55 (2.56)	-1.21 (2.53)	-0.67 (2.44)	-0.82 (2.27)	0.27 (2.07)	2.18 (2.10)	2.12 (2.47)	1.84 (1.80)	1.94 (1.98)	1.78 (1.75)	1.83 (1.53)	1.60 (1.29)
Left superior frontal gyrus	-0.06 (1.64)	-0.21 (1.62)	-0.04 (1.49)	-0.32 (1.66)	-0.57 (1.38)	-0.21 (1.64)	0.05 (1.65)	1.33 (0.89)	1.36 (0.84)	1.16 (0.89)	1.27 (1.07)	1.19 (0.86)	1.34 (0.91)	1.34 (0.90)
Left superior parietal cortex	0.08 (0.80)	0.48 (0.86)	0.25 (0.86)	-0.21 (1.13)	-0.15 (1.05)	-0.12 (1.02)	-0.19 (0.79)	0.58 (0.54)	0.78 (0.58)	0.77 (0.43)	0.92 (0.65)	0.83 (0.62)	0.82 (0.59)	0.70 (0.38)
Left superior temporal gyrus	0.72 (1.34)	-0.22 (1.03)	0.56 (1.57)	0.07 (1.11)	0.49 (1.30)	-0.05 (1.43)	0.77 (1.71)	1.19 (0.92)	0.87 (0.55)	1.27 (1.04)	0.90 (0.61)	1.08 (0.84)	1.17 (0.77)	1.23 (1.40)
Left supramarginal gyrus	-0.85 (1.76)	-0.17 (1.16)	-0.46 (2.00)	0.18 (1.46)	-0.37 (1.51)	0.08 (1.31)	-0.22 (1.62)	1.50 (1.21)	0.92 (0.70)	1.54 (1.31)	1.18 (0.82)	1.22 (0.92)	1.12 (0.63)	1.28 (0.97)
Left frontal pole	-2.87 (8.06)	-0.76 (6.85)	-1.03 (6.94)	-0.07 (6.84)	-1.30 (8.50)	-2.01 (6.72)	-0.76 (7.27)	7.54 (3.63)	5.47 (3.96)	5.19 (4.53)	5.05 (4.43)	6.54 (5.33)	5.62 (3.97)	5.93 (4.00)
Left temporal pole	-0.28 (6.10)	0.57 (6.72)	2.11 (6.30)	0.99 (5.69)	1.00 (6.56)	-1.02 (6.52)	0.42 (7.11)	5.08 (3.12)	5.11 (4.19)	4.67 (4.61)	4.25 (3.76)	4.92 (4.27)	5.34 (3.64)	6.05 (3.42)
Left transverse temporal gyrus	0.87 (4.19)	-0.50 (2.91)	-0.35 (2.08)	-0.61 (4.31)	-0.36 (3.12)	0.17 (1.73)	0.63 (2.07)	2.91 (3.06)	2.16 (1.95)	1.78 (1.04)	3.50 (2.41)	2.49 (1.80)	1.50 (0.79)	1.57 (1.44)
Left insula	0.48 (7.84)	-1.26 (8.31)	0.39 (10.59)	-1.82 (9.40)	1.54 (9.09)	-1.51 (8.38)	0.48 (9.68)	6.06 (4.75)	6.70 (4.79)	8.69 (5.63)	7.97 (4.90)	7.04 (5.68)	6.34 (5.45)	7.95 (5.15)
Right banks superior temporal sulcus	0.29 (3.11)	0.56 (2.94)	1.10 (2.85)	-3.17 (4.17)	-1.57 (4.37)	-0.72 (3.53)	-0.03 (4.12)	2.43 (1.87)	2.32 (1.80)	2.30 (1.95)	3.66 (3.72)	3.51 (2.93)	3.08 (1.70)	3.65 (1.66)
Right caudal anterior cingulate cortex	-1.09 (6.09)	-0.60 (6.67)	-0.84 (8.73)	-0.71 (4.30)	-0.65 (5.50)	-0.63 (6.25)	-0.22 (6.25)	4.91 (3.56)	4.61 (4.72)	6.00 (6.21)	3.35 (2.65)	4.14 (3.51)	4.51 (4.21)	4.45 (4.25)
Right caudal middle frontal gyrus	-0.40 (2.24)	-0.80 (1.81)	-0.94 (2.94)	0.13 (3.10)	0.28 (3.40)	0.40 (2.54)	0.13 (2.34)	1.82 (1.30)	1.59 (1.13)	2.61 (1.53)	2.67 (1.42)	2.57 (2.14)	2.00 (1.53)	1.84 (1.37)
Right cuneus	0.14 (2.26)	-0.09 (1.92)	0.24 (2.25)	0.69 (3.18)	-0.17 (3.37)	1.24 (2.23)	0.30 (2.71)	1.75 (1.37)	1.52 (1.10)	1.72 (1.41)	2.39 (2.14)	2.62 (2.01)	1.97 (1.58)	1.93 (1.85)
Right entorhinal cortex	-2.00 (10.03)	-1.77 (16.25)	-4.40 (14.70)	0.35 (8.13)	-1.05 (9.58)	2.10 (9.13)	0.85 (10.62)	8.30 (5.59)	9.42 (13.15)	11.12 (10.24)	5.97 (5.31)	7.58 (5.63)	7.60 (5.14)	8.85 (5.48)

Right fusiform gyrus	0.71 (1.87)	0.55 (2.68)	0.45 (2.43)	0.38 (2.29)	0.51 (1.87)	-0.26 (2.01)	0.07 (2.03)	1.77 (0.83)	1.94 (1.87)	2.00 (1.36)	1.75 (1.46)	1.67 (0.90)	1.70 (1.00)	1.62 (1.16)
Right inferior parietal cortex	-0.68 (1.77)	-0.80 (2.47)	-0.75 (1.91)	-0.11 (2.70)	-0.32 (2.30)	0.41 (1.82)	-0.15 (1.13)	1.35 (1.30)	1.77 (1.86)	1.33 (1.53)	1.78 (1.99)	1.65 (1.58)	1.25 (1.35)	0.97 (0.55)
Right inferior temporal gyrus	0.46 (2.78)	0.63 (1.92)	0.91 (2.02)	0.09 (2.83)	0.48 (2.68)	0.88 (2.73)	0.46 (2.70)	2.34 (1.45)	1.79 (0.83)	1.91 (1.03)	2.04 (1.89)	2.12 (1.63)	2.33 (1.57)	2.33 (1.32)
Right isthmus cingulate cortex	0.81 (3.18)	0.15 (3.27)	0.37 (3.51)	-1.07 (3.18)	-0.46 (2.88)	-0.05 (2.32)	0.73 (3.01)	2.63 (1.86)	2.76 (1.60)	2.71 (2.15)	2.63 (1.99)	2.12 (1.92)	1.80 (1.38)	2.05 (2.26)
Right lateral occipital cortex	-0.67 (1.66)	-0.10 (1.21)	-0.44 (1.42)	-0.56 (1.68)	-0.84 (1.89)	-0.45 (2.01)	-0.79 (1.77)	1.30 (1.20)	0.93 (0.74)	1.12 (0.94)	1.34 (1.11)	1.51 (1.37)	1.54 (1.30)	1.67 (0.90)
Right lateral orbitofrontal cortex	0.27 (6.91)	-0.87 (3.83)	1.94 (6.17)	1.83 (7.24)	1.01 (6.16)	-0.78 (7.68)	0.83 (6.70)	4.99 (4.60)	3.34 (1.89)	5.32 (3.45)	5.39 (5.00)	4.21 (4.48)	6.12 (4.43)	4.93 (4.43)
Right lingual gyrus	-0.05 (2.12)	0.84 (1.64)	0.68 (2.00)	0.32 (1.66)	0.21 (1.78)	0.56 (2.78)	0.52 (2.25)	1.62 (1.30)	1.37 (1.19)	1.61 (1.30)	1.24 (1.12)	1.20 (1.30)	2.18 (1.74)	1.58 (1.64)
Right medial orbitofrontal cortex	0.35 (4.55)	1.91 (4.30)	-0.06 (2.70)	0.98 (6.44)	0.76 (5.60)	-1.77 (5.45)	-0.81 (4.06)	3.48 (2.82)	3.73 (2.74)	2.33 (1.22)	4.70 (4.35)	4.08 (3.77)	4.67 (3.13)	3.20 (2.51)
Right middle temporal gyrus	0.20 (1.45)	0.02 (1.84)	0.05 (1.40)	0.64 (2.25)	0.15 (1.53)	0.25 (1.47)	0.24 (1.06)	1.17 (0.83)	1.37 (1.17)	1.11 (0.80)	1.79 (1.45)	1.05 (1.10)	1.02 (1.05)	0.87 (0.62)
Right parahippocampal gyrus	1.95 (3.65)	0.70 (4.59)	1.91 (3.60)	-0.01 (5.08)	1.11 (4.50)	0.85 (3.79)	2.49 (3.90)	2.78 (3.02)	3.70 (2.63)	3.10 (2.57)	3.76 (3.28)	3.55 (2.85)	3.26 (1.95)	3.39 (3.10)
Right paracentral lobule	-0.19 (2.99)	-0.33 (2.78)	-0.15 (2.28)	0.13 (2.33)	0.18 (2.84)	-0.20 (2.10)	-0.11 (2.39)	2.39 (1.70)	1.92 (1.98)	1.85 (1.25)	1.47 (1.78)	2.11 (1.83)	1.68 (1.20)	2.02 (1.17)
Right pars opercularis	0.41 (2.15)	-0.19 (3.31)	0.15 (2.40)	1.25 (2.65)	0.23 (2.18)	-1.28 (4.70)	-0.95 (3.10)	1.45 (1.59)	2.40 (2.20)	1.95 (1.32)	2.37 (1.64)	1.82 (1.13)	3.74 (2.98)	2.84 (1.39)
Right pars orbitalis	-0.07 (3.95)	0.69 (2.99)	0.64 (2.83)	0.60 (3.03)	0.39 (3.33)	0.75 (2.69)	0.58 (3.39)	2.90 (2.58)	2.51 (1.64)	2.42 (1.49)	2.24 (2.06)	2.71 (1.84)	2.08 (1.79)	2.78 (1.90)
Right pars triangularis	-0.52 (2.30)	-0.48 (1.98)	-0.49 (2.15)	-1.49 (2.45)	-0.76 (2.39)	-0.39 (2.25)	0.18 (2.03)	1.90 (1.33)	1.71 (1.02)	1.78 (1.22)	2.47 (1.37)	2.11 (1.26)	1.83 (1.28)	1.56 (1.24)
Right pericalcarine cortex	0.34 (3.21)	0.23 (2.40)	0.40 (2.98)	1.78 (2.73)	0.49 (2.95)	0.51 (2.20)	-0.07 (2.16)	2.39 (2.08)	2.09 (1.07)	2.03 (2.16)	2.65 (1.82)	2.44 (1.62)	1.90 (1.12)	1.76 (1.16)
Right postcentral gyrus	-0.21 (1.41)	-0.16 (1.07)	0.27 (1.21)	-0.22 (1.06)	0.10 (1.34)	0.00 (1.29)	-0.03 (1.35)	1.05 (0.93)	0.77 (0.73)	1.03 (0.65)	0.92 (0.52)	1.10 (0.72)	1.04 (0.71)	0.96 (0.91)
Right posterior cingulate cortex	0.16 (2.35)	-0.80 (3.30)	-0.66 (2.30)	-0.46 (2.28)	-0.89 (1.75)	-0.76 (2.36)	-0.47 (2.03)	1.83 (1.41)	2.61 (2.07)	1.97 (1.27)	1.79 (1.42)	1.52 (1.20)	1.97 (1.44)	1.84 (0.87)
Right precentral gyrus	0.11 (1.31)	-0.09 (1.22)	-0.01 (1.72)	-0.15 (0.98)	0.05 (1.42)	0.01 (1.25)	0.48 (1.58)	0.98 (0.83)	0.90 (0.79)	1.18 (1.22)	0.81 (0.54)	1.05 (0.91)	0.90 (0.84)	1.25 (1.04)
Right precuneus	-0.44 (1.57)	-0.01 (1.03)	-0.34 (0.93)	-0.17 (1.40)	-0.27 (1.29)	0.27 (1.18)	-0.21 (1.41)	1.23 (1.02)	0.76 (0.67)	0.86 (0.44)	0.98 (0.97)	1.06 (0.74)	1.03 (0.57)	1.11 (0.86)
Right rostral anterior cingulate cortex	0.48 (3.04)	-0.21 (6.93)	0.06 (5.49)	0.41 (4.98)	-0.47 (4.02)	0.62 (6.64)	0.55 (5.61)	2.48 (1.71)	4.67 (4.98)	3.95 (3.68)	4.14 (2.60)	3.42 (1.97)	5.18 (3.99)	4.10 (3.73)
Right rostral middle frontal gyrus	-0.15 (2.18)	-0.12 (1.95)	-0.01 (2.31)	-0.50 (2.17)	-0.78 (2.02)	-0.38 (2.08)	-0.01 (1.95)	1.53 (1.51)	1.46 (1.24)	1.73 (1.46)	1.62 (1.48)	1.53 (1.50)	1.62 (1.29)	1.49 (1.20)
Right superior frontal gyrus	0.03 (0.90)	0.02 (1.43)	0.04 (1.34)	0.17 (0.84)	-0.17 (0.98)	0.14 (0.98)	0.09 (0.94)	0.69 (0.55)	0.88 (1.10)	0.96 (0.89)	0.65 (0.53)	0.78 (0.59)	0.74 (0.62)	0.74 (0.56)
Right superior parietal cortex	0.61 (1.25)	0.52 (1.33)	0.80 (1.47)	0.02 (2.11)	0.52 (1.34)	-0.33 (0.88)	0.59 (1.40)	0.94 (1.00)	1.05 (0.93)	1.13 (1.22)	1.47 (1.47)	1.07 (0.93)	0.80 (0.46)	1.09 (1.02)
Right superior temporal gyrus	-0.24 (1.16)	-0.59 (1.07)	-0.07 (0.77)	0.17 (1.49)	0.13 (1.20)	-0.48 (1.16)	0.08 (1.34)	0.88 (0.76)	1.02 (0.64)	0.63 (0.43)	1.22 (0.82)	0.88 (0.80)	0.99 (0.73)	1.11 (0.71)
Right supramarginal gyrus	0.12 (1.01)	-0.60 (1.53)	-0.78 (1.49)	-0.13 (1.61)	-0.33 (0.85)	-0.18 (1.26)	-0.30 (1.09)	0.76 (0.64)	1.19 (1.10)	1.40 (0.88)	1.37 (0.79)	0.74 (0.51)	1.00 (0.74)	0.94 (0.60)
Right frontal pole	-1.02 (4.80)	-1.85 (7.20)	0.10 (6.10)	0.78 (6.79)	0.18 (4.79)	-2.26 (7.12)	-2.17 (5.41)	3.65 (3.16)	5.67 (4.61)	3.51 (4.90)	5.05 (4.42)	3.57 (3.06)	5.29 (5.13)	4.75 (3.19)
Right temporal pole	-2.13 (5.95)	-1.96 (7.59)	-2.44 (4.66)	0.17 (5.82)	-2.37 (5.70)	-2.07 (7.45)	-3.54 (6.17)	5.22 (3.35)	5.34 (5.60)	4.03 (3.28)	4.50 (3.50)	5.54 (2.39)	5.30 (5.49)	5.39 (4.53)
Right transverse temporal gyrus	-0.29 (4.37)	1.81 (4.92)	0.89 (3.79)	0.90 (3.30)	0.75 (3.58)	-0.91 (5.45)	-0.87 (3.74)	3.30 (2.75)	4.09 (3.15)	3.15 (2.16)	2.80 (1.84)	2.83 (2.21)	4.03 (3.64)	2.87 (2.45)
Right insula	-1.19 (6.01)	0.41 (7.13)	-2.42 (5.68)	-2.66 (8.88)	-2.56 (7.52)	3.57 (7.72)	1.87 (5.10)	4.27 (4.26)	5.20 (4.71)	4.21 (4.42)	6.96 (5.90)	6.08 (4.91)	6.01 (5.89)	4.09 (3.44)
Local cortical thickness (mm)														
Left banks superior temporal sulcus	0.08 (3.51)	0.13 (2.20)	0.02 (2.22)	-0.48 (2.73)	-0.34 (2.79)	0.37 (2.18)	0.86 (2.96)	2.80 (2.00)	1.60 (1.46)	1.70 (1.35)	2.18 (1.63)	2.30 (1.51)	1.67 (1.38)	2.56 (1.60)
Left caudal anterior cingulate cortex	-1.07 (6.03)	-1.62 (5.22)	-1.56 (6.03)	-2.07 (5.21)	-1.73 (6.20)	-0.87 (6.71)	-1.25 (7.10)	4.50 (4.00)	3.91 (3.70)	4.13 (4.56)	4.46 (3.23)	5.17 (3.63)	5.31 (3.96)	5.16 (4.87)
Left caudal middle frontal gyrus	-0.68 (2.24)	-0.97 (2.55)	-0.98 (2.35)	-1.18 (2.41)	-1.02 (2.91)	-0.40 (2.28)	-0.49 (2.38)	1.75 (1.50)	2.22 (1.50)	2.05 (1.43)	2.13 (1.55)	2.57 (1.60)	1.80 (1.39)	1.70 (1.69)
Left cuneus	0.39 (2.73)	0.09 (2.74)	-0.21 (2.19)	0.27 (2.37)	0.13 (2.37)	0.46 (2.35)	-0.04 (2.06)	2.24 (1.50)	2.37 (1.24)	1.78 (1.20)	1.96 (1.26)	1.78 (1.50)	1.99 (1.24)	1.64 (1.16)
Left entorhinal cortex	1.11 (4.42)	1.05 (5.50)	0.67 (3.41)	1.47 (5.08)	1.55 (4.19)	-0.03 (7.17)	0.35 (4.62)	3.60 (2.65)	4.59 (2.99)	2.69 (2.09)	4.22 (3.02)	3.43 (2.75)	5.60 (4.24)	3.69 (2.63)
Left fusiform gyrus	0.57 (2.06)	0.68 (2.06)	0.69 (2.05)	0.74 (2.18)	0.73 (1.97)	0.70 (2.35)	0.66 (2.07)	1.62 (1.35)	1.46 (1.57)	1.74 (1.22)	1.80 (1.37)	1.60 (1.33)	1.86 (1.53)	1.55 (1.48)
Left inferior parietal cortex	0.13 (1.65)	0.51 (1.32)	0.38 (1.28)	-0.05 (1.66)	0.10 (1.81)	0.64 (1.30)	0.59 (1.39)	1.28 (1.00)	1.14 (0.80)	1.04 (0.80)	1.47 (0.69)	1.58 (0.80)	1.05 (0.98)	0.94 (1.17)
Left inferior temporal gyrus	0.56 (2.73)	1.31 (2.36)	0.91 (2.35)	0.92 (2.75)	0.82 (2.32)	1.44 (2.41)	1.09 (2.09)	2.27 (1.52)	2.05 (1.71)	2.10 (1.31)	2.13 (1.90)	1.76 (1.68)	2.33 (1.49)	1.84 (1.42)
Left isthmus cingulate cortex	0.56 (3.99)	1.30 (3.06)	0.73 (2.97)	-0.63 (2.14)	0.08 (2.02)	1.05 (3.24)	0.65 (3.60)	2.82 (2.79)	2.47 (2.15)	2.32 (1.90)	1.72 (1.37)	1.44 (1.37)	2.63 (2.07)	2.36 (2.72)
Left lateral occipital cortex	-0.36 (1.00)	-0.86 (1.73)	-0.55 (1.10)	-0.58 (1.38)	-0.39 (1.04)	-0.17 (1.57)	0.01 (1.27)	0.82 (0.65)	1.50 (1.17)	0.96 (0.74)	1.23 (0.80)	0.81 (0.74)	1.23 (0.95)	0.93 (0.83)
Left lateral orbitofrontal cortex	0.78 (2.32)	0.58 (2.31)	0.36 (2.41)	0.59 (2.41)	0.58 (2.65)	1.22 (2.41)	1.13 (2.42)	1.91 (1.46)	1.84 (1.45)	2.03 (1.25)	2.10 (1.21)	2.31 (1.30)	2.10 (1.63)	2.12 (1.55)
Left lingual gyrus	-1.31 (3.69)	-1.32 (2.53)	-1.61 (3.07)	-0.82 (2.25)	-1.11 (3.03)	-0.92 (2.74)	-1.57 (3.33)	2.88 (2.57)	2.24 (1.71)	2.80 (1.95)	1.81 (1.51)	2.59 (1.82)	2.34 (1.60)	2.99 (2.05)
Left medial orbitofrontal cortex	-1.02 (3.43)	-0.18 (3.81)	-1.35 (3.41)	-1.14 (3.96)	-1.61 (4.29)	-0.12 (3.08)	-0.50 (3.55)	2.64 (2.34)	2.66 (2.65)	2.49 (2.64)	2.69 (3.05)	3.55 (2.78)	2.14 (2.15)	2.28 (2.71)
Left middle temporal gyrus	0.19 (2.43)	0.10 (1.65)	0.22 (2.15)	0.23 (2.31)	0.32 (2.25)	0.03 (2.58)	0.13 (3.15)	1.91 (1.43)	1.24 (1.06)	1.83 (1.04)	1.89 (1.26)	1.93 (1.09)	1.75 (1.84)	2.36 (1.99)
Left parahippocampal gyrus	0.55 (1.82)	0.19 (2.24)	-0.11 (2.39)	-0.05 (1.47)	0.47 (2.23)	0.35 (1.71)	0.47 (2.24)	1.53 (1.07)	1.75 (1.33)	1.61 (1.73)	1.17 (0.85)	1.87 (1.21)	1.21 (1.23)	1.52 (1.67)

Left paracentral lobule	-0.17 (2.09)	-0.20 (2.79)	-0.33 (2.10)	-0.25 (2.30)	-0.01 (2.67)	-0.04 (2.35)	0.33 (2.51)	1.75 (1.06)	2.31 (1.46)	1.65 (1.26)	1.81 (1.36)	2.21 (1.39)	1.67 (1.60)	2.18 (1.15)
Left pars opercularis	-0.50 (2.32)	0.19 (1.75)	-0.16 (2.34)	-0.23 (1.89)	-0.24 (2.38)	-0.15 (2.59)	-0.10 (2.11)	1.93 (1.31)	1.45 (0.92)	1.62 (1.64)	1.61 (0.92)	1.80 (1.50)	1.90 (1.69)	1.65 (1.24)
Left pars orbitalis	-0.94 (3.02)	-0.90 (2.53)	-0.79 (2.84)	-0.82 (2.36)	-1.18 (2.67)	0.22 (2.97)	-0.02 (2.94)	2.16 (2.26)	2.17 (1.48)	2.26 (1.82)	1.87 (1.60)	2.06 (2.03)	2.39 (1.67)	2.35 (1.66)
Left pars triangularis	-0.51 (2.35)	0.43 (1.57)	0.20 (1.64)	0.27 (2.31)	0.10 (2.37)	0.48 (1.88)	0.66 (2.13)	1.66 (1.69)	1.32 (0.90)	1.30 (0.96)	1.75 (1.46)	1.91 (1.32)	1.65 (0.93)	1.81 (1.23)
Left pericalcarine cortex	-0.61 (5.65)	-0.89 (5.09)	-1.06 (4.75)	-1.59 (3.60)	-1.23 (3.69)	-1.16 (4.97)	-2.15 (4.21)	4.17 (3.71)	3.77 (3.40)	3.83 (2.85)	2.95 (2.52)	3.05 (2.31)	4.04 (2.94)	3.83 (2.65)
Left postcentral gyrus	-0.40 (2.19)	0.03 (1.27)	-0.40 (1.68)	-0.22 (1.80)	-0.17 (2.31)	0.08 (1.34)	-0.17 (2.09)	1.91 (1.04)	1.08 (0.61)	1.51 (0.76)	1.46 (1.02)	1.97 (1.11)	1.05 (0.79)	1.60 (1.28)
Left posterior cingulate cortex	-0.25 (2.58)	-0.24 (2.60)	0.14 (2.47)	-0.73 (2.78)	-0.14 (2.79)	-0.34 (2.27)	-0.14 (2.40)	1.90 (1.68)	2.14 (1.39)	2.20 (0.98)	2.25 (1.70)	2.20 (1.63)	1.70 (1.47)	1.90 (1.40)
Left precentral gyrus	0.06 (1.34)	-0.06 (1.23)	-0.23 (1.44)	-0.15 (1.51)	0.13 (1.87)	0.27 (1.58)	0.37 (1.84)	0.95 (0.92)	0.97 (0.71)	1.15 (0.86)	1.30 (0.71)	1.43 (1.16)	1.23 (0.98)	1.19 (1.42)
Left precuneus	-0.26 (1.52)	-0.03 (1.18)	-0.08 (1.49)	-0.68 (1.48)	-0.35 (1.50)	-0.22 (1.59)	-0.08 (1.52)	1.10 (1.05)	0.94 (0.67)	1.03 (1.04)	1.34 (0.87)	1.18 (0.94)	1.21 (1.00)	0.94 (1.17)
Left rostral anterior cingulate cortex	-1.06 (4.47)	-1.12 (4.26)	-0.74 (4.45)	0.30 (3.23)	0.00 (4.55)	-0.19 (4.71)	-0.64 (4.18)	3.85 (2.32)	3.39 (2.69)	3.33 (2.93)	2.51 (1.95)	3.37 (2.93)	3.67 (2.79)	3.32 (2.48)
Left rostral middle frontal gyrus	-0.59 (2.15)	-0.05 (2.04)	-0.43 (2.15)	-0.69 (2.14)	-0.91 (1.97)	-0.36 (2.27)	-0.51 (2.10)	1.55 (1.55)	1.62 (1.17)	1.62 (1.42)	1.63 (1.50)	1.65 (1.36)	1.45 (1.75)	1.43 (1.58)
Left superior frontal gyrus	-1.35 (2.47)	-1.26 (2.62)	-1.25 (2.38)	-1.24 (2.29)	-1.57 (2.70)	-0.87 (2.98)	-0.91 (2.79)	1.65 (2.26)	1.96 (2.11)	1.75 (2.02)	1.94 (1.69)	2.25 (2.13)	2.20 (2.12)	1.74 (2.33)
Left superior parietal cortex	-0.24 (1.56)	-0.39 (1.80)	-0.43 (1.38)	-0.33 (1.67)	-0.35 (1.91)	-0.36 (1.47)	-0.31 (1.55)	1.24 (0.92)	1.49 (1.02)	1.12 (0.88)	1.43 (0.86)	1.51 (1.17)	1.06 (1.05)	1.17 (1.03)
Left superior temporal gyrus	-0.15 (3.57)	-0.05 (2.59)	-0.19 (3.38)	-0.36 (2.75)	-0.33 (2.93)	0.15 (2.82)	0.21 (3.11)	2.35 (2.62)	1.64 (1.96)	2.18 (2.52)	1.85 (2.01)	1.92 (2.18)	1.82 (2.10)	1.95 (2.38)
Left supramarginal gyrus	0.39 (2.61)	0.14 (1.32)	0.35 (1.85)	-0.06 (1.92)	0.46 (2.70)	0.43 (1.27)	0.67 (2.26)	2.25 (1.26)	0.95 (0.89)	1.46 (1.12)	1.48 (1.16)	2.10 (1.67)	1.01 (0.84)	1.74 (1.53)
Left frontal pole	0.79 (4.65)	-0.26 (4.16)	-0.44 (4.98)	0.10 (4.64)	-1.79 (9.02)	-0.49 (5.63)	-0.21 (6.36)	3.85 (2.55)	3.34 (2.35)	3.70 (3.22)	3.43 (2.99)	5.86 (6.94)	4.41 (3.35)	4.66 (4.17)
Left temporal pole	0.29 (3.87)	0.51 (4.26)	-0.19 (4.51)	0.82 (3.25)	0.37 (4.35)	0.87 (3.87)	0.81 (4.29)	3.01 (2.31)	3.38 (2.50)	3.50 (2.71)	2.66 (1.93)	3.41 (2.58)	3.11 (2.34)	3.43 (2.56)
Left transverse temporal gyrus	-0.09 (3.48)	0.20 (2.54)	0.29 (3.01)	0.35 (2.42)	0.46 (3.09)	0.00 (2.57)	0.02 (3.30)	2.78 (1.98)	1.95 (1.57)	2.35 (1.81)	1.99 (1.32)	2.45 (1.84)	1.98 (1.55)	2.47 (2.09)
Left insula	-0.18 (3.05)	0.74 (3.67)	-0.06 (5.00)	1.66 (4.54)	0.06 (3.92)	1.72 (4.10)	0.38 (3.50)	2.38 (1.81)	2.70 (2.51)	4.07 (2.70)	3.94 (2.65)	2.93 (2.50)	3.44 (2.71)	2.75 (2.09)
Right banks superior temporal sulcus	-0.21 (2.39)	0.58 (2.05)	0.24 (2.99)	0.33 (2.67)	-0.04 (2.94)	1.08 (2.58)	0.52 (2.52)	1.90 (1.38)	1.59 (1.36)	1.84 (2.32)	2.20 (1.45)	1.95 (2.15)	2.22 (1.64)	1.89 (1.67)
Right caudal anterior cingulate cortex	-2.29 (4.69)	-1.91 (4.84)	-1.84 (4.72)	-0.56 (5.14)	-1.59 (4.85)	-2.28 (5.09)	-2.84 (4.58)	4.11 (3.09)	3.60 (3.68)	3.81 (3.24)	3.62 (3.57)	3.73 (3.38)	4.19 (3.56)	4.40 (2.99)
Right caudal middle frontal gyrus	-1.11 (3.78)	-1.18 (3.23)	-0.96 (3.57)	-0.85 (2.82)	-1.33 (3.67)	-0.63 (2.59)	-1.23 (3.49)	2.88 (2.60)	2.54 (2.24)	2.76 (2.36)	2.36 (1.68)	3.05 (2.33)	1.82 (1.89)	2.93 (2.16)
Right cuneus	-0.83 (2.50)	-0.86 (2.59)	-1.09 (2.62)	-1.07 (1.98)	-0.77 (2.17)	-1.29 (1.70)	-1.00 (2.22)	2.00 (1.64)	2.12 (1.65)	2.12 (1.82)	1.91 (1.12)	1.89 (1.24)	1.73 (1.21)	2.12 (1.10)
Right entorhinal cortex	-0.72 (6.66)	1.63 (6.55)	0.34 (6.41)	-0.27 (5.29)	0.68 (5.73)	-0.51 (6.06)	-0.07 (6.48)	5.38 (3.74)	4.91 (4.48)	4.87 (3.99)	4.09 (3.20)	4.48 (3.45)	4.51 (3.92)	5.22 (3.59)
Right fusiform gyrus	0.15 (2.85)	0.13 (2.27)	0.24 (2.41)	-0.05 (3.13)	0.11 (2.76)	0.45 (2.50)	0.41 (2.55)	2.09 (1.87)	1.69 (1.47)	1.75 (1.61)	2.35 (1.98)	2.12 (1.68)	1.85 (1.69)	1.88 (1.71)
Right inferior parietal cortex	-0.05 (1.88)	0.34 (1.76)	-0.02 (1.86)	0.15 (2.18)	-0.19 (2.15)	0.81 (1.90)	0.49 (2.03)	1.47 (1.12)	1.41 (1.04)	1.42 (1.14)	1.66 (1.36)	1.58 (1.41)	1.27 (1.61)	1.47 (1.43)
Right inferior temporal gyrus	0.33 (2.56)	0.25 (2.74)	0.29 (2.63)	0.35 (3.17)	-0.19 (3.09)	0.37 (2.56)	0.28 (2.22)	2.00 (1.55)	2.05 (1.76)	2.01 (1.64)	2.59 (1.74)	2.29 (1.99)	2.06 (1.47)	1.70 (1.39)
Right isthmus cingulate cortex	0.19 (3.27)	-0.34 (2.18)	0.10 (2.76)	0.81 (2.57)	0.84 (3.15)	-0.06 (2.31)	0.10 (3.27)	2.47 (2.06)	1.74 (1.29)	2.23 (1.53)	2.09 (1.63)	2.49 (2.02)	1.80 (1.37)	2.27 (2.28)
Right lateral occipital cortex	0.22 (1.80)	0.47 (2.34)	0.28 (1.76)	0.79 (2.13)	0.44 (1.80)	1.20 (2.01)	0.79 (1.65)	1.20 (1.32)	1.58 (1.74)	1.06 (1.41)	1.70 (1.45)	1.22 (1.36)	1.48 (1.80)	1.06 (1.48)
Right lateral orbitofrontal cortex	-0.27 (3.88)	-0.14 (3.47)	-0.80 (4.34)	-0.01 (3.10)	-0.42 (4.10)	0.65 (2.57)	-0.12 (3.29)	3.22 (2.02)	2.61 (2.20)	3.51 (2.53)	2.32 (1.96)	3.15 (2.53)	1.95 (1.74)	2.35 (2.23)
Right lingual gyrus	-0.83 (2.80)	-0.86 (2.84)	-1.33 (2.53)	-0.67 (2.45)	-0.92 (2.37)	-0.89 (2.94)	-1.16 (2.95)	2.18 (1.87)	2.29 (1.80)	2.31 (1.61)	2.04 (1.42)	2.05 (1.43)	2.43 (1.79)	2.37 (2.04)
Right medial orbitofrontal cortex	-0.70 (3.64)	-0.15 (2.45)	-0.67 (3.01)	-0.75 (3.77)	-1.66 (3.05)	0.93 (3.12)	-0.34 (2.57)	2.63 (2.53)	2.11 (1.13)	2.25 (2.03)	2.99 (2.31)	2.88 (1.85)	2.51 (1.98)	2.03 (1.51)
Right middle temporal gyrus	0.32 (2.00)	0.39 (2.17)	0.48 (2.30)	0.09 (2.61)	-0.41 (2.94)	1.12 (2.21)	0.21 (2.68)	1.60 (1.17)	1.70 (1.34)	1.82 (1.42)	2.06 (1.51)	2.39 (1.65)	2.12 (1.19)	2.06 (1.63)
Right parahippocampal gyrus	0.62 (2.55)	0.83 (2.06)	0.68 (2.58)	0.12 (2.23)	0.62 (2.69)	-0.01 (2.54)	0.38 (2.71)	2.02 (1.60)	1.84 (1.17)	2.24 (1.34)	1.64 (1.45)	2.00 (1.83)	2.09 (1.34)	2.22 (1.50)
Right paracentral lobule	-0.52 (2.74)	-0.40 (2.55)	-0.69 (2.91)	-0.73 (2.28)	-0.51 (2.76)	-0.33 (2.22)	-0.51 (2.45)	1.83 (2.05)	1.57 (2.01)	1.97 (2.19)	1.79 (1.54)	1.82 (2.09)	1.63 (1.48)	1.66 (1.83)
Right pars opercularis	-0.35 (2.13)	-0.32 (2.29)	-0.35 (2.21)	0.26 (1.64)	-0.50 (1.83)	0.42 (1.92)	-0.34 (1.89)	1.84 (1.02)	1.51 (1.71)	1.84 (1.20)	1.13 (1.19)	1.33 (1.32)	1.45 (1.28)	1.38 (1.29)
Right pars orbitalis	0.51 (2.85)	0.08 (2.50)	0.29 (2.66)	-0.08 (2.17)	-0.28 (2.67)	1.13 (3.07)	1.00 (2.56)	2.13 (1.88)	1.97 (1.45)	2.05 (1.64)	1.73 (1.23)	2.04 (1.67)	2.36 (2.20)	2.20 (1.56)
Right pars triangularis	-0.37 (3.22)	0.05 (2.68)	-0.07 (3.08)	0.12 (2.44)	-0.30 (3.00)	0.33 (2.60)	0.13 (2.80)	2.33 (2.18)	2.21 (1.40)	2.53 (1.64)	1.66 (1.73)	2.30 (1.86)	2.07 (1.53)	2.21 (1.63)
Right pericalcarine cortex	-1.32 (4.40)	-1.23 (4.13)	-1.23 (5.02)	-1.27 (4.35)	-0.96 (4.64)	-1.27 (4.60)	-0.59 (4.56)	3.01 (3.40)	3.37 (2.56)	3.59 (3.62)	3.37 (2.92)	3.46 (3.11)	3.70 (2.88)	2.98 (3.41)
Right postcentral gyrus	0.06 (1.83)	0.63 (1.45)	0.30 (1.70)	0.45 (1.65)	0.22 (1.85)	0.35 (1.58)	-0.02 (1.75)	1.54 (0.92)	1.28 (0.89)	1.46 (0.84)	1.34 (1.00)	1.45 (1.11)	1.31 (0.89)	1.37 (1.03)
Right posterior cingulate cortex	0.07 (2.55)	0.02 (2.36)	0.57 (2.58)	0.45 (2.82)	0.76 (2.22)	0.25 (2.53)	0.44 (2.67)	2.10 (1.35)	1.99 (1.16)	2.29 (1.19)	2.28 (1.63)	1.80 (1.44)	1.88 (1.64)	2.26 (1.36)
Right precentral gyrus	-0.08 (2.80)	-0.09 (2.24)	-0.05 (2.40)	0.04 (2.13)	-0.23 (2.53)	0.45 (2.38)	-0.18 (2.67)	2.40 (1.31)	1.39 (1.72)	1.79 (1.53)	1.63 (1.30)	1.93 (1.58)	1.84 (1.51)	2.18 (1.45)
Right precuneus	0.02 (2.01)	0.68 (1.95)	0.13 (2.05)	0.21 (1.59)	-0.09 (1.67)	0.21 (1.53)	-0.05 (1.82)	1.47 (1.31)	1.38 (1.50)	1.48 (1.38)	1.18 (1.04)	1.29 (1.01)	1.28 (0.81)	1.40 (1.10)
Right rostral anterior cingulate cortex	-0.16 (2.66)	0.08 (2.99)	-0.39 (3.28)	-0.30 (3.30)	-0.10 (3.32)	0.37 (3.00)	-0.55 (2.99)	1.72 (1.99)	2.41 (1.66)	2.53 (2.03)	2.41 (2.20)	2.41 (2.20)	2.50 (1.58)	2.46 (1.68)
Right rostral middle frontal gyrus	-1.03 (3.75)	-0.99 (3.14)	-0.95 (3.47)	-0.74 (2.53)	-1.40 (3.14)	-0.35 (2.67)	-0.93 (3.24)	2.71 (2.71)	2.47 (2.10)	2.63 (2.36)	2.07 (1.55)	2.51 (2.29)	2.02 (1.71)	2.33 (2.38)

Right superior frontal gyrus	-1.00 (3.15)	-0.89 (2.66)	-0.82 (2.86)	-1.06 (2.13)	-1.55 (3.09)	-0.18 (2.35)	-0.85 (2.87)	2.48 (2.10)	1.89 (2.04)	2.30 (1.80)	1.82 (1.49)	2.73 (2.05)	1.72 (1.56)	2.31 (1.82)
Right superior parietal cortex	-0.23 (2.07)	0.22 (2.06)	-0.22 (1.98)	-0.33 (1.94)	-0.65 (1.78)	0.54 (2.01)	-0.05 (2.45)	1.66 (1.18)	1.76 (0.99)	1.59 (1.13)	1.48 (1.24)	1.54 (1.04)	1.67 (1.18)	1.58 (1.83)
Right superior temporal gyrus	-0.65 (2.22)	-0.41 (1.99)	-0.70 (1.88)	-0.62 (1.92)	-1.02 (2.19)	0.17 (1.74)	-0.29 (2.03)	1.64 (1.58)	1.51 (1.31)	1.49 (1.29)	1.59 (1.18)	1.65 (1.73)	1.40 (1.00)	1.49 (1.35)
Right supramarginal gyrus	-0.27 (1.88)	0.14 (1.39)	-0.12 (1.49)	-0.29 (1.77)	-0.51 (1.90)	0.65 (1.85)	0.16 (2.02)	1.61 (0.92)	1.21 (0.64)	1.10 (0.97)	1.46 (0.99)	1.51 (1.20)	1.36 (1.38)	1.74 (0.94)
Right frontal pole	1.96 (5.21)	0.42 (4.88)	0.86 (4.96)	0.68 (5.28)	-2.00 (9.00)	0.21 (3.61)	0.53 (4.38)	3.70 (4.07)	3.12 (3.69)	3.44 (3.58)	4.20 (3.10)	5.76 (7.07)	2.46 (2.57)	3.37 (2.72)
Right temporal pole	2.38 (5.73)	2.80 (6.15)	2.35 (6.69)	2.04 (6.54)	1.88 (7.01)	2.62 (6.38)	2.71 (6.59)	3.23 (5.27)	4.31 (5.13)	3.94 (5.84)	4.86 (4.69)	4.19 (5.85)	4.25 (5.36)	3.88 (5.93)
Right transverse temporal gyrus	-0.69 (3.51)	-1.20 (4.03)	-1.48 (3.91)	-1.55 (3.57)	-1.43 (3.87)	-0.97 (3.52)	-1.03 (4.05)	2.40 (2.58)	2.90 (2.97)	2.96 (2.87)	3.08 (2.27)	2.80 (2.96)	2.82 (2.21)	2.92 (2.91)
Right insula	0.76 (3.68)	0.62 (3.28)	1.16 (3.49)	1.69 (4.13)	1.19 (4.11)	-1.06 (4.89)	-1.02 (4.51)	2.64 (2.60)	2.50 (2.12)	2.90 (2.16)	3.43 (2.74)	3.06 (2.90)	3.49 (3.48)	3.67 (2.66)
Global brain measures														
Left total cortical volume (ml)	-0.31 (1.53)	-0.23 (1.25)	-0.32 (1.40)	-0.43 (1.44)	-0.47 (1.56)	-0.06 (1.38)	0.01 (1.48)	1.09 (1.08)	0.90 (0.86)	0.92 (1.09)	1.13 (0.95)	1.22 (1.04)	1.07 (0.83)	1.07 (0.99)
Right total cortical volume (ml)	-0.31 (1.99)	-0.24 (1.64)	-0.29 (1.75)	-0.30 (1.62)	-0.68 (1.88)	0.10 (1.70)	-0.19 (1.88)	1.43 (1.37)	1.03 (1.27)	1.14 (1.33)	1.23 (1.05)	1.29 (1.51)	1.06 (1.30)	1.29 (1.34)
Left total cortical white matter (ml)	0.12 (1.02)	0.12 (0.69)	0.33 (0.78)	0.17 (0.88)	0.23 (1.10)	0.13 (0.70)	0.41 (0.91)	0.73 (0.69)	0.58 (0.35)	0.68 (0.48)	0.64 (0.61)	0.90 (0.62)	0.56 (0.42)	0.75 (0.64)
Right total cortical white matter (ml)	0.15 (1.08)	0.21 (0.92)	0.35 (1.02)	0.34 (0.91)	0.21 (0.91)	0.27 (0.91)	0.31 (1.06)	0.79 (0.72)	0.68 (0.64)	0.80 (0.69)	0.71 (0.64)	0.70 (0.60)	0.76 (0.54)	0.78 (0.76)
Left total cortical surface area (cm^2)	-0.08 (0.63)	0.00 (0.75)	0.03 (0.77)	-0.13 (0.63)	-0.05 (0.66)	-0.15 (0.69)	0.14 (0.69)	0.45 (0.43)	0.52 (0.53)	0.55 (0.52)	0.41 (0.48)	0.49 (0.43)	0.48 (0.50)	0.55 (0.42)
Right total cortical surface area (cm^2)	-0.06 (0.51)	-0.04 (0.64)	0.00 (0.48)	-0.04 (0.53)	-0.15 (0.47)	0.02 (0.48)	0.09 (0.40)	0.42 (0.27)	0.50 (0.38)	0.35 (0.33)	0.41 (0.32)	0.36 (0.33)	0.38 (0.29)	0.29 (0.27)
Left mean cortical thickness (mm)	-0.23 (1.15)	-0.10 (0.75)	-0.24 (0.93)	-0.23 (1.04)	-0.24 (1.18)	0.09 (1.09)	0.02 (1.11)	0.88 (0.75)	0.60 (0.44)	0.69 (0.64)	0.87 (0.58)	1.00 (0.64)	0.92 (0.53)	0.81 (0.73)
Right mean cortical tickness (mm)	-0.28 (1.67)	-0.08 (1.28)	-0.24 (1.46)	-0.13 (1.28)	-0.47 (1.53)	0.25 (1.31)	-0.16 (1.54)	1.25 (1.10)	0.90 (0.88)	1.06 (0.99)	1.04 (0.70)	1.18 (1.05)	1.00 (0.85)	1.14 (1.01)
Left cerebellar white matter (ml)	-0.80 (4.58)	-0.90 (4.31)	-1.73 (4.56)	-1.45 (4.28)	-1.84 (4.19)	-0.77 (4.32)	-1.46 (4.07)	3.28 (3.20)	3.45 (2.60)	3.65 (3.13)	3.49 (2.76)	3.48 (2.87)	2.78 (3.32)	3.05 (2.99)
Right cerebellar white matter (ml)	-0.62 (4.62)	1.04 (3.54)	-0.66 (3.35)	0.02 (3.34)	-0.74 (3.57)	-0.37 (4.25)	-1.12 (3.99)	3.35 (3.12)	3.03 (1.97)	2.54 (2.20)	2.56 (2.04)	3.12 (1.71)	2.79 (3.14)	3.05 (2.71)
Left cerebellar gray matter (ml)	0.01 (1.21)	0.02 (1.45)	0.03 (1.46)	0.07 (1.38)	-0.06 (1.34)	0.31 (1.27)	0.33 (1.31)	0.94 (0.72)	1.03 (0.98)	1.06 (0.97)	0.99 (0.93)	1.04 (0.80)	0.88 (0.94)	1.05 (0.81)
Right cerebellar gray matter (ml)	-0.03 (0.92)	-0.11 (1.24)	-0.06 (0.91)	0.14 (1.16)	-0.21 (1.24)	0.05 (0.85)	0.32 (1.03)	0.72 (0.55)	0.99 (0.71)	0.67 (0.59)	0.94 (0.65)	1.00 (0.72)	0.68 (0.48)	0.85 (0.63)
Intracranial volume (ml)	0.62 (2.06)	-0.23 (3.42)	0.83 (2.28)	-0.49 (4.43)	-3.87 (6.58)	-0.02 (1.06)	2.44 (4.31)	1.11 (1.82)	1.55 (3.03)	1.11 (2.15)	2.03 (3.94)	4.05 (6.46)	0.73 (0.75)	2.52 (4.26)
Total brain volume without ventricles (ml)	-0.09 (0.75)	-0.05 (0.56)	-0.04 (0.66)	-0.09 (0.70)	-0.24 (0.88)	0.10 (0.55)	0.10 (0.77)	0.67 (0.31)	0.42 (0.37)	0.57 (0.31)	0.54 (0.43)	0.68 (0.58)	0.42 (0.35)	0.54 (0.54)

Abbreviations: PD=Average Percentage Difference; SD=standard deviation; Orig = Original scan; FS df =FreeSurfer defacing; FSL df =FSL defacing; FM = Face Masking; Suffix 1 and 2 indicate first and second scan respectively, i.e. test-retest.

Table S8. Effects of de-identification on test-retest reliability in young adults: Pearson's correlation coefficients and intra-class correlation coefficients

Brain metric	Pearson's r							ICC (95% CI)						
	Orig1 - Orig2	Orig1 - Orig2	Orig1 - FS df2	FSL df1 - FSL df2	Orig1 - FSL df2	FM1 - FM2	Orig1 - FM2	Orig1 - Orig2	Orig1 - Orig2	Orig1 - FS df2	FSL df1 - FSL df2	Orig1 - FSL df2	FM1 - FM2	Orig1 - FM2
Subcortical volume (ml)														
Left thalamus	0.96	0.96	0.96	0.96	0.98	0.97	0.98	0.97 (0.90-0.99)	0.95 (0.86-0.98)	0.95 (0.87-0.98)	0.96 (0.89-0.99)	0.98 (0.94-0.99)	0.97 (0.91-0.99)	0.98 (0.95-0.99)
Left caudatus	0.97	0.98	0.98	0.98	0.98	0.99	0.98	0.96 (0.89-0.99)	0.98 (0.94-0.99)	0.97 (0.91-0.99)	0.98 (0.96-0.99)	0.98 (0.94-0.99)	0.99 (0.96-1.00)	0.97 (0.88-0.99)
Left putamen	0.97	0.97	0.96	0.97	0.96	0.98	0.96	0.97 (0.91-0.99)	0.97 (0.93-0.99)	0.96 (0.88-0.98)	0.97 (0.92-0.99)	0.96 (0.88-0.98)	0.98 (0.94-0.99)	0.96 (0.89-0.99)
Left pallidum	0.90	0.87	0.9	0.94	0.93	0.84	0.87	0.90 (0.73-0.96)	0.87 (0.66-0.95)	0.91 (0.75-0.97)	0.93 (0.82-0.98)	0.93 (0.81-0.98)	0.84 (0.61-0.94)	0.88 (0.69-0.96)
Left hippocampus	0.97	0.94	0.97	0.96	0.96	0.96	0.96	0.97 (0.93-0.99)	0.94 (0.84-0.98)	0.96 (0.90-0.99)	0.96 (0.89-0.99)	0.96 (0.90-0.99)	0.96 (0.88-0.98)	0.95 (0.85-0.98)
Left amygdala	0.93	0.91	0.93	0.79	0.85	0.89	0.88	0.93 (0.80-0.97)	0.91 (0.77-0.97)	0.93 (0.82-0.98)	0.80 (0.51-0.93)	0.85 (0.63-0.95)	0.89 (0.72-0.96)	0.88 (0.69-0.96)
Left accumbens	0.87	0.96	0.91	0.9	0.93	0.84	0.87	0.85 (0.62-0.94)	0.96 (0.88-0.98)	0.90 (0.74-0.96)	0.90 (0.74-0.96)	0.93 (0.81-0.97)	0.85 (0.61-0.94)	0.85 (0.62-0.94)
Right thalamus	0.97	0.96	0.96	0.97	0.96	0.96	0.96	0.97 (0.91-0.99)	0.95 (0.85-0.98)	0.95 (0.83-0.98)	0.96 (0.84-0.99)	0.96 (0.87-0.99)	0.94 (0.82-0.98)	0.95 (0.84-0.98)
Right caudatus	0.97	0.95	0.96	0.97	0.99	0.98	0.98	0.97 (0.92-0.99)	0.95 (0.86-0.98)	0.96 (0.88-0.98)	0.96 (0.90-0.99)	0.98 (0.91-0.99)	0.97 (0.85-0.99)	0.97 (0.90-0.99)
Right putamen	0.99	0.99	0.99	0.99	0.99	0.99	0.99	0.99 (0.98-1.00)	0.99 (0.97-1.00)	0.99 (0.97-1.00)	0.99 (0.96-1.00)	0.99 (0.97-1.00)	0.99 (0.98-1.00)	0.99 (0.95-1.00)
Right pallidum	0.94	0.93	0.9	0.95	0.95	0.88	0.86	0.94 (0.83-0.98)	0.93 (0.81-0.97)	0.91 (0.75-0.97)	0.95 (0.86-0.98)	0.94 (0.83-0.98)	0.88 (0.71-0.96)	0.87 (0.66-0.95)
Right hippocampus	0.98	0.93	0.96	0.95	0.96	0.94	0.96	0.97 (0.90-0.99)	0.93 (0.82-0.98)	0.96 (0.89-0.99)	0.96 (0.88-0.98)	0.96 (0.90-0.99)	0.93 (0.82-0.98)	0.95 (0.87-0.98)
Right amygdala	0.86	0.87	0.87	0.83	0.86	0.79	0.83	0.86 (0.66-0.95)	0.87 (0.68-0.95)	0.88 (0.68-0.96)	0.82 (0.57-0.93)	0.86 (0.65-0.95)	0.79 (0.50-0.92)	0.83 (0.58-0.94)
Right accumbens	0.86	0.88	0.86	0.94	0.9	0.93	0.88	0.86 (0.65-0.95)	0.88 (0.69-0.96)	0.86 (0.65-0.95)	0.94 (0.84-0.98)	0.91 (0.76-0.97)	0.93 (0.83-0.98)	0.89 (0.71-0.96)
Ventricular volume (ml)														
Third ventricle	0.99	0.99	0.99	0.99	0.99	0.99	0.99	0.99 (0.98-1.00)	0.99 (0.98-1.00)	0.99 (0.98-1.00)	0.99 (0.97-1.00)	0.99 (0.98-1.00)	0.99 (0.97-1.00)	0.99 (0.97-1.00)
Left lateral ventricle	1.00	1	1	1	1	1	1	1.00 (1.00-1.00)	1.00 (1.00-1.00)	1.00 (1.00-1.00)	1.00 (1.00-1.00)	1.00 (1.00-1.00)	1.00 (1.00-1.00)	1.00 (1.00-1.00)
Left inferior lateral ventricle	0.98	0.97	0.97	0.97	0.96	0.97	0.97	0.98 (0.95-0.99)	0.97 (0.91-0.99)	0.96 (0.89-0.99)	0.97 (0.92-0.99)	0.96 (0.90-0.99)	0.97 (0.91-0.99)	0.97 (0.92-0.99)
Right lateral ventricle	1.00	1	1	1	1	1	1	1.00 (1.00-1.00)	1.00 (1.00-1.00)	1.00 (1.00-1.00)	1.00 (1.00-1.00)	1.00 (1.00-1.00)	1.00 (1.00-1.00)	1.00 (1.00-1.00)
Right inferior lateral ventricle	0.97	0.98	0.98	0.98	0.97	0.98	0.99	0.97 (0.92-0.99)	0.98 (0.96-0.99)	0.98 (0.95-0.99)	0.98 (0.94-0.99)	0.97 (0.93-0.99)	0.98 (0.94-0.99)	0.99 (0.97-1.00)
Local cortical volume (ml)														
Left banks superior temporal sulcus	0.95	0.98	0.97	0.98	0.96	0.97	0.96	0.95 (0.87-0.98)	0.97 (0.93-0.99)	0.97 (0.92-0.99)	0.97 (0.93-0.99)	0.95 (0.87-0.98)	0.97 (0.91-0.99)	0.96 (0.90-0.99)
Left caudal anterior cingulate cortex	0.98	0.98	0.98	0.98	0.98	0.99	0.99	0.97 (0.89-0.99)	0.98 (0.95-0.99)	0.98 (0.94-0.99)	0.98 (0.93-0.99)	0.98 (0.94-0.99)	0.99 (0.97-1.00)	0.99 (0.96-1.00)
Left caudal middle frontal gyrus	0.98	0.98	0.97	0.98	0.97	0.99	0.97	0.98 (0.94-0.99)	0.97 (0.93-0.99)	0.96 (0.90-0.99)	0.97 (0.92-0.99)	0.96 (0.90-0.99)	0.98 (0.96-0.99)	0.97 (0.93-0.99)
Left cuneus	0.97	0.98	0.98	0.99	0.99	0.99	0.98	0.97 (0.92-0.99)	0.98 (0.94-0.99)	0.98 (0.94-0.99)	0.99 (0.96-1.00)	0.99 (0.96-1.00)	0.99 (0.96-1.00)	0.98 (0.95-0.99)
Left entorhinal cortex	0.82	0.86	0.85	0.67	0.77	0.85	0.8	0.81 (0.53-0.93)	0.86 (0.64-0.95)	0.86 (0.64-0.95)	0.68 (0.29-0.88)	0.77 (0.46-0.92)	0.86 (0.64-0.95)	0.80 (0.52-0.93)
Left fusiform gyrus	0.97	0.95	0.94	0.95	0.95	0.95	0.96	0.96 (0.90-0.99)	0.95 (0.85-0.98)	0.94 (0.84-0.98)	0.95 (0.85-0.98)	0.95 (0.86-0.98)	0.94 (0.81-0.98)	0.95 (0.86-0.98)
Left inferior parietal cortex	0.99	0.99	0.99	0.98	0.99	0.99	0.99	0.99 (0.96-1.00)	0.99 (0.97-1.00)	0.99 (0.98-1.00)	0.98 (0.95-0.99)	0.99 (0.96-1.00)	0.99 (0.96-1.00)	0.99 (0.98-1.00)
Left inferior temporal gyrus	0.95	0.96	0.96	0.96	0.97	0.97	0.98	0.95 (0.87-0.98)	0.96 (0.88-0.99)	0.96 (0.90-0.99)	0.96 (0.90-0.99)	0.96 (0.90-0.99)	0.97 (0.91-0.99)	0.97 (0.92-0.99)
Left isthmus cingulate cortex	0.96	0.99	0.97	0.97	0.95	0.95	0.94	0.96 (0.89-0.99)	0.98 (0.96-0.99)	0.97 (0.92-0.99)	0.97 (0.91-0.99)	0.95 (0.88-0.98)	0.96 (0.88-0.98)	0.94 (0.84-0.98)
Left lateral occipital cortex	1.00	0.99	1	0.99	0.99	0.99	1	1.00 (0.99-1.00)	0.99 (0.97-1.00)	0.99 (0.98-1.00)	0.98 (0.96-0.99)	0.99 (0.97-1.00)	0.99 (0.98-1.00)	0.99 (0.98-1.00)
Left lateral orbitofrontal cortex	0.95	0.96	0.96	0.95	0.95	0.93	0.92	0.95 (0.86-0.98)	0.95 (0.87-0.98)	0.96 (0.89-0.99)	0.95 (0.86-0.98)	0.96 (0.88-0.98)	0.93 (0.82-0.98)	0.93 (0.80-0.97)
Left lingual gyrus	0.99	0.98	0.98	0.99	0.99	0.99	0.99	0.99 (0.96-1.00)	0.98 (0.95-0.99)	0.98 (0.95-0.99)	0.99 (0.97-1.00)	0.99 (0.96-1.00)	0.99 (0.96-1.00)	0.99 (0.96-1.00)
Left medial orbitofrontal cortex	0.89	0.8	0.87	0.82	0.8	0.93	0.85	0.89 (0.71-0.96)	0.79 (0.51-0.92)	0.85 (0.63-0.94)	0.82 (0.55-0.93)	0.78 (0.49-0.92)	0.93 (0.81-0.97)	0.85 (0.63-0.95)
Left middle temporal gyrus	0.96	0.97	0.97	0.97	0.97	0.93	0.9	0.96 (0.89-0.99)	0.97 (0.91-0.99)	0.97 (0.91-0.99)	0.97 (0.92-0.99)	0.97 (0.91-0.99)	0.94 (0.83-0.98)	0.90 (0.75-0.97)
Left parahippocampal gyrus	0.96	0.94	0.9	0.94	0.92	0.88	0.93	0.96 (0.88-0.98)	0.94 (0.84-0.98)	0.90 (0.75-0.97)	0.94 (0.84-0.98)	0.92 (0.80-0.97)	0.89 (0.71-0.96)	0.93 (0.81-0.97)
Left paracentral lobule	0.99	0.98	0.99	0.99	0.99	0.99	0.99	0.99 (0.96-0.99)	0.97 (0.93-0.99)	0.99 (0.96-0.99)	0.99 (0.97-1.00)	0.98 (0.95-0.99)	0.97 (0.91-0.99)	0.98 (0.94-0.99)
Left pars opercularis	0.98	0.97	0.97	0.98	0.98	0.99	0.98	0.97 (0.89-0.99)	0.97 (0.93-0.99)	0.97 (0.92-0.99)	0.98 (0.95-0.99)	0.98 (0.93-0.99)	0.99 (0.97-1.00)	0.97 (0.91-0.99)
Left pars orbitalis	0.95	0.93	0.94	0.94	0.96	0.96	0.96	0.95 (0.86-0.98)	0.93 (0.81-0.97)	0.94 (0.83-0.98)	0.94 (0.81-0.98)	0.95 (0.85-0.98)	0.96 (0.88-0.98)	0.96 (0.88-0.98)

Left pars triangularis	0.98	0.96	0.97	0.98	0.98	0.98	0.98	0.98 (0.94-0.99)	0.97 (0.90-0.99)	0.98 (0.93-0.99)	0.98 (0.95-0.99)	0.98 (0.95-0.99)	0.98 (0.94-0.99)	0.98 (0.95-0.99)
Left pericalcarine cortex	0.98	0.96	0.96	0.98	0.98	0.97	0.96	0.97 (0.90-0.99)	0.95 (0.86-0.98)	0.96 (0.89-0.98)	0.97 (0.91-0.99)	0.97 (0.91-0.99)	0.96 (0.86-0.99)	0.94 (0.79-0.98)
Left postcentral gyrus	0.98	0.99	0.99	0.99	0.98	1	0.99	0.98 (0.95-0.99)	0.99 (0.97-1.00)	0.99 (0.97-1.00)	0.99 (0.97-1.00)	0.98 (0.95-0.99)	0.99 (0.97-1.00)	0.99 (0.96-1.00)
Left posterior cingulate cortex	0.99	0.99	0.99	0.99	0.99	0.98	0.99	0.99 (0.97-1.00)	0.99 (0.96-1.00)	0.99 (0.97-1.00)	0.99 (0.97-1.00)	0.99 (0.98-1.00)	0.98 (0.94-0.99)	0.99 (0.96-0.99)
Left precentral gyrus	0.99	0.99	0.99	0.97	0.98	0.98	0.98	0.99 (0.96-0.99)	0.99 (0.97-1.00)	0.99 (0.97-1.00)	0.97 (0.93-0.99)	0.98 (0.93-0.99)	0.98 (0.95-0.99)	0.98 (0.93-0.99)
Left precuneus	0.98	0.99	0.98	0.99	0.99	0.99	0.98	0.98 (0.92-0.99)	0.99 (0.97-1.00)	0.98 (0.93-0.99)	0.99 (0.92-1.00)	0.98 (0.91-0.99)	0.99 (0.97-1.00)	0.98 (0.94-0.99)
Left rostral anterior cingulate cortex	0.97	0.92	0.95	0.96	0.97	0.97	0.97	0.97 (0.92-0.99)	0.93 (0.80-0.97)	0.95 (0.87-0.98)	0.96 (0.90-0.99)	0.98 (0.93-0.99)	0.97 (0.91-0.99)	0.97 (0.92-0.99)
Left rostral middle frontal gyrus	0.98	0.97	0.97	0.98	0.98	0.98	0.98	0.98 (0.94-0.99)	0.97 (0.92-0.99)	0.97 (0.93-0.99)	0.97 (0.92-0.99)	0.98 (0.93-0.99)	0.98 (0.93-0.99)	0.98 (0.95-0.99)
Left superior frontal gyrus	0.95	0.97	0.96	0.96	0.96	0.96	0.95	0.95 (0.87-0.98)	0.96 (0.89-0.99)	0.96 (0.88-0.98)	0.95 (0.86-0.98)	0.94 (0.81-0.98)	0.96 (0.88-0.98)	0.95 (0.86-0.98)
Left superior parietal cortex	0.99	0.98	0.99	0.98	0.99	0.99	0.99	0.99 (0.96-1.00)	0.98 (0.95-0.99)	0.99 (0.96-0.99)	0.98 (0.95-0.99)	0.98 (0.94-0.99)	0.98 (0.96-0.99)	0.98 (0.96-0.99)
Left superior temporal gyrus	0.96	0.97	0.95	0.97	0.97	0.94	0.94	0.96 (0.89-0.99)	0.98 (0.93-0.99)	0.95 (0.87-0.98)	0.98 (0.93-0.99)	0.97 (0.93-0.99)	0.94 (0.85-0.98)	0.94 (0.85-0.98)
Left supramarginal gyrus	0.98	0.99	0.98	0.98	0.98	0.99	0.98	0.98 (0.95-0.99)	0.99 (0.98-1.00)	0.98 (0.95-0.99)	0.98 (0.95-0.99)	0.98 (0.94-0.99)	0.99 (0.96-1.00)	0.98 (0.94-0.99)
Left frontal pole	0.73	0.75	0.8	0.77	0.59	0.88	0.74	0.73 (0.37-0.90)	0.75 (0.42-0.91)	0.80 (0.52-0.93)	0.77 (0.47-0.91)	0.58 (0.14-0.83)	0.85 (0.64-0.95)	0.74 (0.40-0.90)
Left temporal pole	0.66	0.82	0.81	0.87	0.77	0.67	0.71	0.65 (0.26-0.86)	0.80 (0.50-0.92)	0.76 (0.37-0.91)	0.85 (0.59-0.94)	0.72 (0.36-0.89)	0.64 (0.25-0.85)	0.65 (0.26-0.86)
Left transverse temporal gyrus	0.96	0.96	0.96	0.96	0.96	0.97	0.96	0.94 (0.85-0.98)	0.96 (0.89-0.99)	0.97 (0.91-0.99)	0.96 (0.90-0.99)	0.96 (0.88-0.98)	0.97 (0.92-0.99)	0.96 (0.89-0.99)
Left insula	0.84	0.89	0.81	0.77	0.78	0.9	0.78	0.83 (0.58-0.94)	0.83 (0.59-0.94)	0.81 (0.54-0.93)	0.78 (0.47-0.92)	0.78 (0.49-0.92)	0.88 (0.70-0.96)	0.78 (0.48-0.92)
Right banks superior temporal sulcus	0.95	0.96	0.96	0.95	0.94	0.96	0.93	0.94 (0.85-0.98)	0.96 (0.87-0.98)	0.95 (0.86-0.98)	0.93 (0.79-0.98)	0.93 (0.81-0.97)	0.96 (0.90-0.99)	0.92 (0.79-0.97)
Right caudal anterior cingulate cortex	0.93	0.87	0.84	0.98	0.92	0.88	0.9	0.91 (0.75-0.97)	0.84 (0.61-0.94)	0.82 (0.57-0.93)	0.97 (0.90-0.99)	0.90 (0.74-0.96)	0.86 (0.66-0.95)	0.89 (0.71-0.96)
Right caudal middle frontal gyrus	0.96	0.97	0.95	0.96	0.95	0.96	0.95	0.96 (0.88-0.98)	0.97 (0.90-0.99)	0.95 (0.87-0.98)	0.96 (0.89-0.99)	0.96 (0.88-0.98)	0.96 (0.90-0.99)	0.95 (0.87-0.98)
Right cuneus	0.99	0.99	0.99	0.99	0.98	0.99	0.99	0.98 (0.96-0.99)	0.98 (0.96-0.99)	0.98 (0.96-0.99)	0.99 (0.96-1.00)	0.98 (0.94-0.99)	0.99 (0.98-1.00)	0.99 (0.97-1.00)
Right entorhinal cortex	0.73	0.65	0.64	0.77	0.72	0.77	0.79	0.71 (0.34-0.89)	0.67 (0.26-0.87)	0.61 (0.18-0.84)	0.78 (0.48-0.92)	0.71 (0.35-0.89)	0.77 (0.44-0.91)	0.73 (0.38-0.90)
Right fusiform gyrus	0.96	0.95	0.95	0.98	0.97	0.97	0.96	0.96 (0.89-0.99)	0.95 (0.86-0.98)	0.95 (0.87-0.98)	0.98 (0.93-0.99)	0.97 (0.92-0.99)	0.97 (0.91-0.99)	0.96 (0.89-0.99)
Right inferior parietal cortex	0.98	0.99	0.99	0.98	0.99	0.99	0.99	0.98 (0.94-0.99)	0.99 (0.96-1.00)	0.98 (0.96-0.99)	0.98 (0.95-0.99)	0.99 (0.96-0.99)	0.98 (0.95-0.99)	0.99 (0.97-1.00)
Right inferior temporal gyrus	0.93	0.93	0.94	0.89	0.9	0.92	0.94	0.93 (0.81-0.97)	0.93 (0.82-0.98)	0.93 (0.82-0.98)	0.89 (0.72-0.96)	0.90 (0.75-0.97)	0.91 (0.77-0.97)	0.93 (0.82-0.98)
Right isthmus cingulate cortex	0.94	0.98	0.98	0.97	0.96	0.98	0.98	0.94 (0.84-0.98)	0.97 (0.93-0.99)	0.98 (0.94-0.99)	0.97 (0.92-0.99)	0.96 (0.90-0.99)	0.98 (0.94-0.99)	0.98 (0.95-0.99)
Right lateral occipital cortex	0.99	0.99	0.99	0.98	0.99	0.99	0.99	0.99 (0.98-1.00)	0.99 (0.97-1.00)	0.99 (0.97-1.00)	0.98 (0.95-0.99)	0.98 (0.95-0.99)	0.99 (0.96-0.99)	0.99 (0.96-0.99)
Right lateral orbitofrontal cortex	0.92	0.93	0.94	0.91	0.95	0.86	0.89	0.92 (0.79-0.97)	0.91 (0.77-0.97)	0.93 (0.82-0.98)	0.91 (0.78-0.97)	0.95 (0.86-0.98)	0.87 (0.67-0.95)	0.89 (0.72-0.96)
Right lingual gyrus	0.98	0.99	0.99	0.98	0.99	0.97	0.98	0.98 (0.95-0.99)	0.99 (0.96-1.00)	0.99 (0.97-1.00)	0.98 (0.95-0.99)	0.99 (0.97-1.00)	0.97 (0.93-0.99)	0.98 (0.95-0.99)
Right medial orbitofrontal cortex	0.96	0.96	0.98	0.93	0.96	0.94	0.95	0.96 (0.90-0.99)	0.96 (0.88-0.98)	0.98 (0.93-0.99)	0.94 (0.83-0.98)	0.95 (0.87-0.98)	0.93 (0.82-0.98)	0.95 (0.86-0.98)
Right middle temporal gyrus	0.98	0.97	0.98	0.95	0.95	0.97	0.96	0.98 (0.93-0.99)	0.97 (0.92-0.99)	0.98 (0.93-0.99)	0.95 (0.86-0.98)	0.95 (0.85-0.98)	0.97 (0.90-0.99)	0.96 (0.89-0.99)
Right parahippocampal gyrus	0.99	0.97	0.99	0.96	0.98	0.98	0.99	0.99 (0.96-0.99)	0.97 (0.92-0.99)	0.98 (0.96-0.99)	0.96 (0.90-0.99)	0.98 (0.93-0.99)	0.98 (0.93-0.99)	0.98 (0.93-1.00)
Right paracentral lobule	0.96	0.97	0.95	0.98	0.95	0.97	0.96	0.95 (0.88-0.98)	0.96 (0.89-0.99)	0.94 (0.85-0.98)	0.98 (0.94-0.99)	0.95 (0.87-0.98)	0.97 (0.92-0.99)	0.96 (0.89-0.99)
Right pars opercularis	0.98	0.95	0.98	0.96	0.97	0.91	0.95	0.98 (0.94-0.99)	0.94 (0.85-0.98)	0.96 (0.89-0.99)	0.95 (0.86-0.98)	0.97 (0.91-0.99)	0.91 (0.77-0.97)	0.95 (0.87-0.98)
Right pars orbitalis	0.95	0.98	0.96	0.96	0.95	0.96	0.95	0.95 (0.88-0.98)	0.98 (0.94-0.99)	0.96 (0.90-0.99)	0.96 (0.90-0.99)	0.95 (0.87-0.98)	0.96 (0.88-0.98)	0.95 (0.86-0.98)
Right pars triangularis	0.97	0.98	0.98	0.99	0.98	0.97	0.97	0.96 (0.89-0.99)	0.98 (0.93-0.99)	0.98 (0.94-0.99)	0.98 (0.94-0.99)	0.97 (0.92-0.99)	0.97 (0.91-0.99)	0.97 (0.93-0.99)
Right pericalcarine cortex	0.97	0.98	0.97	0.98	0.97	0.98	0.98	0.97 (0.91-0.99)	0.98 (0.94-0.99)	0.96 (0.90-0.99)	0.98 (0.94-0.99)	0.97 (0.92-0.99)	0.97 (0.91-0.99)	0.97 (0.91-0.99)
Right postcentral gyrus	0.95	0.98	0.97	0.96	0.96	0.96	0.95	0.95 (0.87-0.98)	0.98 (0.95-0.99)	0.97 (0.92-0.99)	0.96 (0.90-0.99)	0.96 (0.89-0.99)	0.96 (0.89-0.99)	0.95 (0.88-0.98)
Right posterior cingulate cortex	0.99	0.98	0.99	0.98	0.99	0.98	0.99	0.99 (0.97-1.00)	0.98 (0.95-0.99)	0.99 (0.97-1.00)	0.98 (0.96-0.99)	0.99 (0.97-1.00)	0.98 (0.95-0.99)	0.99 (0.97-1.00)
Right precentral gyrus	0.96	0.97	0.95	0.97	0.97	0.96	0.97	0.97 (0.90-0.99)	0.97 (0.91-0.99)	0.96 (0.88-0.98)	0.97 (0.92-0.99)	0.97 (0.91-0.99)	0.96 (0.88-0.98)	0.97 (0.91-0.99)
Right precuneus	0.97	0.99	0.98	0.99	0.99	0.98	0.96	0.97 (0.92-0.99)	0.99 (0.96-1.00)	0.98 (0.94-0.99)	0.99 (0.96-1.00)	0.99 (0.97-1.00)	0.98 (0.94-0.99)	0.97 (0.90-0.99)
Right rostral anterior cingulate cortex	0.96	0.93	0.95	0.97	0.97	0.97	0.98	0.96 (0.89-0.99)	0.91 (0.76-0.97)	0.94 (0.84-0.98)	0.96 (0.90-0.99)	0.96 (0.89-0.99)	0.97 (0.91-0.99)	0.97 (0.93-0.99)
Right rostral middle frontal gyrus	0.94	0.95	0.95	0.97	0.96	0.97	0.96	0.94 (0.83-0.98)	0.95 (0.86-0.98)	0.94 (0.85-0.98)	0.97 (0.91-0.99)	0.95 (0.83-0.98)	0.97 (0.91-0.99)	0.95 (0.87-0.98)
Right superior frontal gyrus	0.96	0.95	0.94	0.97	0.96	0.96	0.96	0.96 (0.88-0.98)	0.95 (0.87-0.98)	0.94 (0.85-0.98)	0.97 (0.93-0.99)	0.95 (0.85-0.98)	0.96 (0.90-0.99)	0.96 (0.88-0.98)
Right superior parietal cortex	0.98	0.97	0.97	0.98	0.98	0.98	0.97	0.98 (0.94-0.99)	0.97 (0.91-0.99)	0.97 (0.92-0.99)	0.97 (0.92-0.99)	0.98 (0.95-0.99)	0.98 (0.94-0.99)	0.97 (0.92-0.99)
Right superior temporal gyrus	0.96	0.98	0.97	0.96	0.95	0.98	0.97	0.96 (0.88-0.98)	0.97 (0.87-0.99)	0.97 (0.91-0.99)	0.95 (0.88-0.98)	0.94 (0.85-0.98)	0.98 (0.94-0.99)	0.97 (0.92-0.99)

Right supramarginal gyrus	0.99	0.99	0.99	0.99	0.99	0.99	0.99	0.99 (0.97-1.00)	0.99 (0.98-1.00)	0.99 (0.97-1.00)	0.99 (0.97-1.00)	0.99 (0.98-1.00)	0.99 (0.98-1.00)	0.99 (0.97-1.00)
Right frontal pole	0.89	0.72	0.76	0.72	0.65	0.71	0.8	0.89 (0.72-0.96)	0.72 (0.37-0.89)	0.74 (0.39-0.90)	0.72 (0.35-0.89)	0.58 (0.15-0.83)	0.69 (0.32-0.88)	0.77 (0.42-0.91)
Right temporal pole	0.73	0.91	0.83	0.74	0.73	0.88	0.82	0.73 (0.37-0.90)	0.92 (0.78-0.97)	0.84 (0.60-0.94)	0.73 (0.39-0.90)	0.73 (0.38-0.90)	0.88 (0.69-0.95)	0.81 (0.54-0.93)
Right transverse temporal gyrus	0.95	0.94	0.96	0.94	0.94	0.94	0.96	0.95 (0.87-0.98)	0.94 (0.83-0.98)	0.95 (0.86-0.98)	0.94 (0.84-0.98)	0.94 (0.83-0.98)	0.94 (0.83-0.98)	0.95 (0.87-0.98)
Right insula	0.92	0.91	0.92	0.91	0.93	0.92	0.96	0.93 (0.80-0.97)	0.90 (0.75-0.96)	0.92 (0.80-0.97)	0.90 (0.75-0.96)	0.93 (0.81-0.97)	0.90 (0.69-0.97)	0.95 (0.84-0.98)
Local cortical surface (cm^2)														
Left banks superior temporal sulcus	0.98	0.98	0.98	0.99	0.97	0.98	0.98	0.98 (0.95-0.99)	0.98 (0.95-0.99)	0.98 (0.93-0.99)	0.99 (0.97-1.00)	0.97 (0.92-0.99)	0.98 (0.95-0.99)	0.98 (0.94-0.99)
Left caudal anterior cingulate cortex	0.99	0.99	0.99	0.98	0.99	1	1	0.98 (0.96-0.99)	0.99 (0.97-1.00)	0.99 (0.97-1.00)	0.98 (0.95-0.99)	0.99 (0.96-1.00)	0.99 (0.98-1.00)	1.00 (0.99-1.00)
Left caudal middle frontal gyrus	0.99	0.99	0.98	0.99	0.99	0.99	0.99	0.99 (0.98-1.00)	0.99 (0.97-1.00)	0.98 (0.96-0.99)	0.99 (0.97-1.00)	0.99 (0.97-1.00)	0.99 (0.98-1.00)	0.99 (0.98-1.00)
Left cuneus	0.98	0.99	0.99	0.98	0.99	0.99	0.99	0.98 (0.95-0.99)	0.99 (0.96-1.00)	0.99 (0.97-1.00)	0.98 (0.94-0.99)	0.98 (0.96-0.99)	0.99 (0.96-1.00)	0.99 (0.97-1.00)
Left entorhinal cortex	0.85	0.91	0.84	0.89	0.79	0.93	0.88	0.86 (0.63-0.95)	0.91 (0.77-0.97)	0.85 (0.62-0.95)	0.89 (0.72-0.96)	0.79 (0.50-0.92)	0.92 (0.79-0.97)	0.89 (0.71-0.96)
Left fusiform gyrus	0.99	0.99	0.98	0.99	0.99	0.99	0.99	0.99 (0.97-1.00)	0.98 (0.96-0.99)	0.98 (0.95-0.99)	0.99 (0.96-0.99)	0.99 (0.96-1.00)	0.98 (0.95-0.99)	0.99 (0.96-1.00)
Left inferior parietal cortex	0.99	1	0.99	0.99	0.99	0.99	1	0.99 (0.98-1.00)	1.00 (0.99-1.00)	0.99 (0.99-1.00)	0.99 (0.97-1.00)	0.99 (0.98-1.00)	0.99 (0.98-1.00)	1.00 (0.99-1.00)
Left inferior temporal gyrus	0.99	0.97	0.99	0.99	0.99	0.99	0.99	0.99 (0.97-1.00)	0.97 (0.93-0.99)	0.99 (0.97-1.00)	0.99 (0.97-1.00)	0.99 (0.97-1.00)	0.99 (0.97-1.00)	0.99 (0.97-1.00)
Left isthmus cingulate cortex	0.97	0.97	0.98	0.99	0.99	0.97	0.97	0.97 (0.92-0.99)	0.98 (0.93-0.99)	0.98 (0.95-0.99)	0.99 (0.98-1.00)	0.99 (0.97-1.00)	0.97 (0.93-0.99)	0.97 (0.91-0.99)
Left lateral occipital cortex	1.00	1	0.99	0.99	0.99	1	1	1.00 (0.99-1.00)	1.00 (0.98-1.00)	0.99 (0.98-1.00)	0.99 (0.98-1.00)	0.99 (0.98-1.00)	1.00 (0.98-1.00)	1.00 (0.99-1.00)
Left lateral orbitofrontal cortex	0.96	0.98	0.96	0.96	0.97	0.96	0.95	0.96 (0.89-0.99)	0.98 (0.94-0.99)	0.96 (0.90-0.99)	0.96 (0.90-0.99)	0.97 (0.91-0.99)	0.96 (0.88-0.99)	0.95 (0.87-0.98)
Left lingual gyrus	0.98	0.99	0.99	0.99	0.99	0.99	0.99	0.98 (0.95-0.99)	0.99 (0.98-1.00)	0.99 (0.93-1.00)	0.99 (0.98-1.00)	0.98 (0.96-0.99)	0.99 (0.98-1.00)	0.99 (0.95-1.00)
Left medial orbitofrontal cortex	0.86	0.7	0.79	0.77	0.74	0.88	0.78	0.86 (0.65-0.95)	0.67 (0.29-0.87)	0.78 (0.48-0.92)	0.77 (0.47-0.92)	0.74 (0.40-0.90)	0.89 (0.72-0.96)	0.78 (0.49-0.92)
Left middle temporal gyrus	0.98	0.98	0.97	0.98	0.98	0.98	0.97	0.98 (0.94-0.99)	0.98 (0.93-0.99)	0.97 (0.93-0.99)	0.98 (0.94-0.99)	0.98 (0.93-0.99)	0.98 (0.93-0.99)	0.97 (0.92-0.99)
Left parahippocampal gyrus	0.98	0.97	0.97	0.98	0.97	0.96	0.97	0.98 (0.94-0.99)	0.98 (0.93-0.99)	0.97 (0.93-0.99)	0.98 (0.94-0.99)	0.97 (0.93-0.99)	0.96 (0.89-0.99)	0.97 (0.92-0.99)
Left paracentral lobule	0.99	0.99	1	1	1	0.99	0.99	0.99 (0.98-1.00)	0.99 (0.98-1.00)	1.00 (0.99-1.00)	1.00 (0.99-1.00)	1.00 (0.99-1.00)	0.99 (0.97-1.00)	0.99 (0.98-1.00)
Left pars opercularis	0.99	0.98	0.98	0.99	0.99	0.99	0.99	0.98 (0.94-0.99)	0.98 (0.95-0.99)	0.98 (0.93-0.99)	0.99 (0.98-1.00)	0.98 (0.95-0.99)	0.99 (0.98-1.00)	0.98 (0.94-0.99)
Left pars orbitalis	0.96	0.97	0.97	0.98	0.99	0.96	0.97	0.96 (0.88-0.98)	0.97 (0.92-0.99)	0.97 (0.91-0.99)	0.98 (0.95-0.99)	0.99 (0.96-1.00)	0.96 (0.89-0.99)	0.97 (0.91-0.99)
Left pars triangularis	0.99	0.99	0.99	0.99	0.99	0.99	0.99	0.99 (0.98-1.00)	0.99 (0.97-1.00)	0.99 (0.98-1.00)	0.99 (0.97-1.00)	0.99 (0.98-1.00)	0.99 (0.96-1.00)	0.99 (0.98-1.00)
Left pericalcarine cortex	0.99	0.99	0.99	0.99	0.99	0.98	0.98	0.99 (0.97-1.00)	0.99 (0.97-1.00)	0.99 (0.96-1.00)	0.99 (0.97-1.00)	0.99 (0.97-1.00)	0.98 (0.95-0.99)	0.98 (0.94-0.99)
Left postcentral gyrus	0.99	0.99	1	1	1	0.99	1	0.99 (0.98-1.00)	0.99 (0.98-1.00)	1.00 (0.99-1.00)	1.00 (0.99-1.00)	1.00 (0.99-1.00)	0.99 (0.98-1.00)	1.00 (0.99-1.00)
Left posterior cingulate cortex	0.99	0.99	0.99	0.99	0.98	0.98	0.98	0.99 (0.96-1.00)	0.99 (0.98-1.00)	0.99 (0.97-1.00)	0.99 (0.97-1.00)	0.98 (0.95-0.99)	0.98 (0.95-0.99)	0.98 (0.96-0.99)
Left precentral gyrus	0.99	1	1	1	1	1	1	0.99 (0.98-1.00)	1.00 (0.99-1.00)	1.00 (0.99-1.00)	0.99 (0.99-1.00)	0.99 (0.99-1.00)	0.99 (0.99-1.00)	0.99 (0.99-1.00)
Left precuneus	0.99	1	0.99	0.99	1	0.99	0.99	0.99 (0.96-1.00)	1.00 (0.99-1.00)	0.99 (0.96-1.00)	0.99 (0.98-1.00)	0.99 (0.94-1.00)	0.99 (0.98-1.00)	0.99 (0.98-1.00)
Left rostral anterior cingulate cortex	0.98	0.93	0.95	0.96	0.98	0.98	0.98	0.98 (0.94-0.99)	0.92 (0.80-0.97)	0.95 (0.86-0.98)	0.96 (0.89-0.99)	0.98 (0.95-0.99)	0.98 (0.94-0.99)	0.98 (0.95-0.99)
Left rostral middle frontal gyrus	0.98	0.98	0.99	0.99	0.99	0.99	0.99	0.98 (0.95-0.99)	0.98 (0.94-0.99)	0.99 (0.96-1.00)	0.99 (0.96-0.99)	0.99 (0.97-1.00)	0.99 (0.97-1.00)	0.99 (0.98-1.00)
Left superior frontal gyrus	0.99	0.99	0.99	0.99	0.99	0.99	0.99	0.99 (0.98-1.00)	0.99 (0.98-1.00)	0.99 (0.98-1.00)	0.99 (0.97-1.00)	0.99 (0.98-1.00)	0.99 (0.97-1.00)	0.99 (0.97-1.00)
Left superior parietal cortex	1.00	1	1	0.99	1	1	1	1.00 (0.99-1.00)	1.00 (0.99-1.00)	1.00 (0.99-1.00)	0.99 (0.98-1.00)	1.00 (0.99-1.00)	1.00 (0.99-1.00)	1.00 (0.99-1.00)
Left superior temporal gyrus	1.00	1	0.99	1	0.99	0.99	0.99	0.99 (0.97-1.00)	1.00 (0.99-1.00)	0.99 (0.98-1.00)	1.00 (0.99-1.00)	0.99 (0.98-1.00)	0.99 (0.98-1.00)	0.99 (0.97-1.00)
Left supramarginal gyrus	0.99	1	0.99	1	0.99	1	0.99	0.99 (0.97-1.00)	1.00 (0.99-1.00)	0.99 (0.97-1.00)	1.00 (0.99-1.00)	0.99 (0.99-1.00)	1.00 (0.99-1.00)	0.99 (0.98-1.00)
Left frontal pole	0.56	0.7	0.62	0.77	0.63	0.73	0.65	0.53 (0.09-0.80)	0.70 (0.33-0.88)	0.63 (0.21-0.85)	0.76 (0.44-0.91)	0.60 (0.16-0.84)	0.73 (0.39-0.90)	0.64 (0.22-0.86)
Left temporal pole	0.75	0.6	0.74	0.71	0.71	0.64	0.65	0.74 (0.40-0.90)	0.62 (0.18-0.85)	0.71 (0.36-0.89)	0.72 (0.36-0.89)	0.71 (0.34-0.89)	0.65 (0.23-0.86)	0.66 (0.25-0.87)
Left transverse temporal gyrus	0.96	0.98	0.99	0.96	0.97	0.99	0.99	0.95 (0.86-0.98)	0.98 (0.94-0.99)	0.99 (0.97-1.00)	0.96 (0.89-0.99)	0.97 (0.93-0.99)	0.99 (0.98-1.00)	0.99 (0.97-1.00)
Left insula	0.77	0.88	0.61	0.71	0.7	0.75	0.67	0.70 (0.32-0.88)	0.83 (0.59-0.94)	0.63 (0.19-0.85)	0.71 (0.36-0.89)	0.71 (0.34-0.89)	0.74 (0.40-0.90)	0.67 (0.27-0.87)
Right banks superior temporal sulcus	0.99	0.98	0.98	0.96	0.97	0.97	0.97	0.98 (0.94-0.99)	0.98 (0.94-0.99)	0.98 (0.93-0.99)	0.95 (0.75-0.98)	0.96 (0.88-0.98)	0.97 (0.92-0.99)	0.96 (0.88-0.99)
Right caudal anterior cingulate cortex	0.97	0.96	0.93	0.99	0.97	0.96	0.96	0.96 (0.89-0.99)	0.95 (0.86-0.98)	0.92 (0.79-0.97)	0.98 (0.95-0.99)	0.97 (0.91-0.99)	0.96 (0.88-0.98)	0.96 (0.89-0.99)
Right caudal middle frontal gyrus	0.99	0.99	0.98	0.97	0.97	0.98	0.99	0.99 (0.96-1.00)	0.99 (0.97-1.00)	0.98 (0.94-0.99)	0.98 (0.93-0.99)	0.97 (0.92-0.99)	0.98 (0.95-0.99)	0.99 (0.96-1.00)
Right cuneus	1.00	0.99	0.99	0.99	0.99	0.99	0.99	0.99 (0.97-1.00)	0.99 (0.98-1.00)	0.99 (0.98-1.00)	0.98 (0.95-0.99)	0.98 (0.95-0.99)	0.99 (0.96-1.00)	0.99 (0.97-1.00)
Right entorhinal cortex	0.90	0.79	0.85	0.93	0.9	0.92	0.89	0.90 (0.74-0.96)	0.80 (0.51-0.92)	0.83 (0.59-0.94)	0.93 (0.82-0.98)	0.91 (0.75-0.97)	0.92 (0.79-0.97)	0.88 (0.70-0.96)

Right fusiform gyrus	1.00	0.99	0.99	1	1	0.99	1	0.99 (0.99-1.00)	0.99 (0.97-1.00)	0.99 (0.98-1.00)	0.99 (0.97-1.00)	1.00 (0.99-1.00)	0.99 (0.99-1.00)	1.00 (0.99-1.00)
Right inferior parietal cortex	0.99	0.99	0.99	0.98	0.99	0.99	1	0.99 (0.98-1.00)	0.99 (0.96-0.99)	0.99 (0.97-1.00)	0.98 (0.95-0.99)	0.99 (0.97-1.00)	0.99 (0.98-1.00)	1.00 (0.99-1.00)
Right inferior temporal gyrus	0.98	0.99	0.99	0.97	0.98	0.98	0.98	0.98 (0.93-0.99)	0.99 (0.97-1.00)	0.99 (0.96-0.99)	0.97 (0.93-0.99)	0.98 (0.94-0.99)	0.97 (0.93-0.99)	0.98 (0.94-0.99)
Right isthmus cingulate cortex	0.99	0.99	0.99	0.99	0.99	0.99	0.99	0.99 (0.96-0.99)	0.99 (0.97-1.00)	0.98 (0.96-0.99)	0.99 (0.96-1.00)	0.99 (0.97-1.00)	0.99 (0.98-1.00)	0.98 (0.94-0.99)
Right lateral occipital cortex	1.00	1	1	1	0.99	0.99	1	1.00 (0.99-1.00)	1.00 (1.00-1.00)	1.00 (0.99-1.00)	1.00 (0.99-1.00)	0.99 (0.98-1.00)	0.99 (0.98-1.00)	0.99 (0.98-1.00)
Right lateral orbitofrontal cortex	0.89	0.97	0.9	0.89	0.92	0.85	0.89	0.89 (0.72-0.96)	0.97 (0.91-0.99)	0.90 (0.73-0.96)	0.88 (0.70-0.96)	0.91 (0.77-0.97)	0.84 (0.60-0.94)	0.90 (0.73-0.96)
Right lingual gyrus	0.99	0.99	0.99	0.99	0.99	0.99	0.99	0.99 (0.98-1.00)	0.99 (0.98-1.00)	0.99 (0.97-1.00)	0.99 (0.98-1.00)	0.99 (0.98-1.00)	0.98 (0.96-0.99)	0.99 (0.97-1.00)
Right medial orbitofrontal cortex	0.94	0.95	0.98	0.88	0.91	0.91	0.95	0.94 (0.84-0.98)	0.94 (0.82-0.98)	0.98 (0.94-0.99)	0.88 (0.68-0.95)	0.91 (0.77-0.97)	0.90 (0.75-0.96)	0.95 (0.86-0.98)
Right middle temporal gyrus	0.99	0.99	0.99	0.98	0.99	0.99	1	0.99 (0.98-1.00)	0.99 (0.96-1.00)	0.99 (0.98-1.00)	0.98 (0.94-0.99)	0.99 (0.97-1.00)	0.99 (0.98-1.00)	1.00 (0.99-1.00)
Right parahippocampal gyrus	0.99	0.97	0.99	0.97	0.98	0.98	0.98	0.98 (0.94-0.99)	0.96 (0.90-0.99)	0.98 (0.94-0.99)	0.97 (0.92-0.99)	0.98 (0.94-0.99)	0.98 (0.95-0.99)	0.98 (0.90-0.99)
Right paracentral lobule	0.97	0.98	0.98	0.98	0.97	0.99	0.98	0.97 (0.93-0.99)	0.98 (0.94-0.99)	0.98 (0.95-0.99)	0.98 (0.95-0.99)	0.97 (0.93-0.99)	0.99 (0.97-1.00)	0.98 (0.95-0.99)
Right pars opercularis	0.98	0.96	0.99	0.98	0.99	0.94	0.97	0.98 (0.96-0.99)	0.96 (0.89-0.99)	0.98 (0.94-0.99)	0.97 (0.92-0.99)	0.98 (0.96-0.99)	0.93 (0.83-0.98)	0.97 (0.93-0.99)
Right pars orbitalis	0.96	0.98	0.99	0.98	0.98	0.99	0.98	0.97 (0.90-0.99)	0.98 (0.95-0.99)	0.99 (0.96-0.99)	0.98 (0.95-0.99)	0.98 (0.94-0.99)	0.99 (0.97-1.00)	0.98 (0.94-0.99)
Right pars triangularis	0.99	0.99	0.99	0.99	0.99	0.99	0.99	0.99 (0.98-1.00)	0.99 (0.98-1.00)	0.99 (0.98-1.00)	0.99 (0.94-1.00)	0.99 (0.97-1.00)	0.99 (0.98-1.00)	0.99 (0.98-1.00)
Right pericalcarine cortex	0.99	0.99	0.99	0.99	0.99	0.99	0.99	0.99 (0.97-1.00)	0.99 (0.98-1.00)	0.99 (0.97-1.00)	0.98 (0.93-0.99)	0.99 (0.97-1.00)	0.99 (0.97-1.00)	0.99 (0.98-1.00)
Right postcentral gyrus	0.98	0.99	0.99	0.99	0.99	0.99	0.99	0.98 (0.95-0.99)	0.99 (0.98-1.00)	0.99 (0.97-1.00)	0.99 (0.98-1.00)	0.99 (0.96-1.00)	0.99 (0.96-1.00)	0.98 (0.96-0.99)
Right posterior cingulate cortex	0.99	0.98	0.99	0.99	0.99	0.99	0.99	0.99 (0.96-1.00)	0.98 (0.93-0.99)	0.99 (0.97-1.00)	0.99 (0.97-1.00)	0.99 (0.98-1.00)	0.99 (0.97-1.00)	0.99 (0.98-1.00)
Right precentral gyrus	0.99	0.99	0.99	1	0.99	1	0.99	0.99 (0.99-1.00)	0.99 (0.99-1.00)	0.99 (0.97-1.00)	1.00 (0.99-1.00)	0.99 (0.98-1.00)	0.99 (0.98-1.00)	0.99 (0.97-1.00)
Right precuneus	0.99	1	1	0.99	0.99	1	0.99	0.99 (0.98-1.00)	1.00 (0.99-1.00)	1.00 (0.99-1.00)	0.99 (0.98-1.00)	0.99 (0.99-1.00)	1.00 (0.99-1.00)	0.99 (0.98-1.00)
Right rostral anterior cingulate cortex	0.99	0.96	0.97	0.97	0.98	0.96	0.97	0.99 (0.97-1.00)	0.94 (0.85-0.98)	0.97 (0.91-0.99)	0.97 (0.92-0.99)	0.98 (0.94-0.99)	0.96 (0.88-0.99)	0.97 (0.92-0.99)
Right rostral middle frontal gyrus	0.99	0.99	0.99	0.99	0.99	0.99	0.99	0.99 (0.97-1.00)	0.99 (0.97-1.00)	0.99 (0.97-1.00)	0.99 (0.96-1.00)	0.99 (0.96-1.00)	0.99 (0.97-1.00)	0.99 (0.97-1.00)
Right superior frontal gyrus	1.00	0.99	0.99	1	1	1	1	1.00 (0.99-1.00)	0.99 (0.97-1.00)	0.99 (0.98-1.00)	1.00 (0.99-1.00)	1.00 (0.99-1.00)	1.00 (0.99-1.00)	1.00 (0.99-1.00)
Right superior parietal cortex	0.99	0.99	0.99	0.98	0.99	1	0.99	0.99 (0.97-1.00)	0.99 (0.97-1.00)	0.99 (0.96-1.00)	0.98 (0.94-0.99)	0.99 (0.97-1.00)	1.00 (0.99-1.00)	0.99 (0.97-1.00)
Right superior temporal gyrus	1.00	1	1	0.99	1	1	0.99	1.00 (0.99-1.00)	0.99 (0.98-1.00)	1.00 (0.99-1.00)	0.99 (0.98-1.00)	0.99 (0.99-1.00)	0.99 (0.98-1.00)	0.99 (0.98-1.00)
Right supramarginal gyrus	1.00	1	1	1	1	1	1	1.00 (0.99-1.00)	0.99 (0.98-1.00)	0.99 (0.98-1.00)	1.00 (0.99-1.00)	1.00 (1.00-1.00)	1.00 (0.99-1.00)	1.00 (0.99-1.00)
Right frontal pole	0.92	0.83	0.87	0.82	0.91	0.77	0.87	0.91 (0.77-0.97)	0.82 (0.58-0.93)	0.86 (0.64-0.95)	0.83 (0.58-0.94)	0.91 (0.76-0.97)	0.76 (0.45-0.91)	0.85 (0.63-0.95)
Right temporal pole	0.81	0.81	0.92	0.88	0.88	0.8	0.85	0.80 (0.53-0.92)	0.81 (0.54-0.93)	0.89 (0.70-0.96)	0.87 (0.67-0.95)	0.85 (0.63-0.94)	0.80 (0.52-0.92)	0.80 (0.49-0.93)
Right transverse temporal gyrus	0.95	0.96	0.96	0.97	0.97	0.94	0.97	0.95 (0.87-0.98)	0.94 (0.85-0.98)	0.96 (0.90-0.99)	0.97 (0.91-0.99)	0.97 (0.91-0.99)	0.94 (0.84-0.98)	0.97 (0.92-0.99)
Right insula	0.90	0.85	0.91	0.79	0.83	0.84	0.94	0.88 (0.70-0.96)	0.85 (0.61-0.94)	0.90 (0.75-0.97)	0.75 (0.43-0.90)	0.80 (0.53-0.93)	0.81 (0.52-0.93)	0.93 (0.80-0.97)
Local cortical thickness (mm)														
Left banks superior temporal sulcus	0.81	0.93	0.93	0.89	0.89	0.92	0.86	0.82 (0.56-0.94)	0.93 (0.81-0.98)	0.93 (0.82-0.98)	0.89 (0.73-0.96)	0.89 (0.72-0.96)	0.93 (0.81-0.97)	0.86 (0.65-0.95)
Left caudal anterior cingulate cortex	0.69	0.82	0.74	0.82	0.72	0.7	0.65	0.67 (0.28-0.87)	0.81 (0.55-0.93)	0.69 (0.33-0.88)	0.80 (0.52-0.92)	0.67 (0.30-0.87)	0.71 (0.34-0.89)	0.61 (0.18-0.84)
Left caudal middle frontal gyrus	0.84	0.8	0.8	0.85	0.77	0.8	0.77	0.83 (0.59-0.94)	0.78 (0.48-0.92)	0.78 (0.48-0.92)	0.83 (0.57-0.94)	0.74 (0.42-0.90)	0.80 (0.52-0.92)	0.77 (0.47-0.91)
Left cuneus	0.88	0.86	0.92	0.91	0.91	0.91	0.94	0.88 (0.69-0.96)	0.87 (0.66-0.95)	0.92 (0.80-0.97)	0.91 (0.77-0.97)	0.92 (0.78-0.97)	0.91 (0.78-0.97)	0.94 (0.84-0.98)
Left entorhinal cortex	0.90	0.86	0.95	0.88	0.92	0.8	0.92	0.90 (0.75-0.96)	0.86 (0.66-0.95)	0.95 (0.86-0.98)	0.87 (0.68-0.95)	0.91 (0.76-0.97)	0.80 (0.51-0.93)	0.92 (0.78-0.97)
Left fusiform gyrus	0.89	0.9	0.9	0.86	0.89	0.84	0.89	0.89 (0.73-0.96)	0.89 (0.72-0.96)	0.89 (0.73-0.96)	0.86 (0.65-0.95)	0.89 (0.71-0.96)	0.84 (0.61-0.94)	0.88 (0.70-0.96)
Left inferior parietal cortex	0.84	0.91	0.9	0.82	0.8	0.9	0.89	0.82 (0.55-0.93)	0.89 (0.72-0.96)	0.90 (0.74-0.96)	0.83 (0.57-0.94)	0.80 (0.51-0.92)	0.88 (0.67-0.96)	0.87 (0.65-0.95)
Left inferior temporal gyrus	0.88	0.91	0.9	0.85	0.89	0.89	0.92	0.86 (0.66-0.95)	0.88 (0.65-0.96)	0.89 (0.70-0.96)	0.83 (0.60-0.94)	0.88 (0.70-0.96)	0.86 (0.60-0.95)	0.89 (0.69-0.96)
Left isthmus cingulate cortex	0.88	0.94	0.95	0.97	0.98	0.94	0.91	0.89 (0.71-0.96)	0.92 (0.79-0.97)	0.94 (0.85-0.98)	0.96 (0.89-0.99)	0.97 (0.92-0.99)	0.92 (0.78-0.97)	0.91 (0.77-0.97)
Left lateral occipital cortex	0.97	0.89	0.96	0.93	0.96	0.92	0.95	0.96 (0.89-0.99)	0.87 (0.66-0.95)	0.94 (0.84-0.98)	0.92 (0.78-0.97)	0.95 (0.87-0.98)	0.92 (0.79-0.97)	0.94 (0.85-0.98)
Left lateral orbitofrontal cortex	0.86	0.86	0.85	0.8	0.81	0.85	0.85	0.85 (0.63-0.94)	0.86 (0.65-0.95)	0.86 (0.64-0.95)	0.80 (0.53-0.93)	0.80 (0.53-0.93)	0.82 (0.53-0.93)	0.82 (0.55-0.93)
Left lingual gyrus	0.84	0.91	0.86	0.94	0.89	0.9	0.85	0.82 (0.57-0.93)	0.89 (0.69-0.96)	0.84 (0.57-0.94)	0.94 (0.83-0.98)	0.87 (0.68-0.95)	0.89 (0.71-0.96)	0.83 (0.56-0.94)
Left medial orbitofrontal cortex	0.66	0.67	0.7	0.49	0.48	0.76	0.69	0.65 (0.27-0.86)	0.68 (0.29-0.88)	0.67 (0.30-0.87)	0.48 (0.01-0.78)	0.46 (0.00-0.76)	0.76 (0.44-0.91)	0.69 (0.31-0.88)
Left middle temporal gyrus	0.83	0.91	0.86	0.78	0.83	0.8	0.72	0.84 (0.60-0.94)	0.92 (0.78-0.97)	0.86 (0.65-0.95)	0.79 (0.49-0.92)	0.82 (0.57-0.93)	0.80 (0.52-0.93)	0.73 (0.38-0.90)
Left parahippocampal gyrus	0.97	0.96	0.95	0.99	0.96	0.98	0.96	0.97 (0.92-0.99)	0.96 (0.89-0.99)	0.95 (0.87-0.98)	0.98 (0.95-0.99)	0.96 (0.88-0.98)	0.98 (0.93-0.99)	0.96 (0.89-0.99)

Left paracentral lobule	0.88	0.85	0.93	0.92	0.89	0.9	0.88	0.89 (0.71-0.96)	0.86 (0.64-0.95)	0.90 (0.75-0.97)	0.91 (0.77-0.97)	0.86 (0.65-0.95)	0.90 (0.73-0.96)	0.86 (0.65-0.95)
Left pars opercularis	0.88	0.93	0.87	0.91	0.86	0.85	0.9	0.88 (0.69-0.96)	0.93 (0.82-0.98)	0.88 (0.68-0.95)	0.91 (0.77-0.97)	0.87 (0.66-0.95)	0.84 (0.61-0.94)	0.90 (0.75-0.97)
Left pars orbitalis	0.85	0.89	0.85	0.9	0.88	0.84	0.84	0.85 (0.62-0.94)	0.88 (0.69-0.95)	0.85 (0.62-0.94)	0.89 (0.72-0.96)	0.86 (0.64-0.95)	0.85 (0.61-0.94)	0.85 (0.63-0.95)
Left pars triangularis	0.91	0.96	0.96	0.92	0.91	0.95	0.93	0.91 (0.78-0.97)	0.96 (0.90-0.99)	0.96 (0.89-0.99)	0.92 (0.79-0.97)	0.91 (0.77-0.97)	0.94 (0.85-0.98)	0.93 (0.81-0.97)
Left pericalcarine cortex	0.71	0.78	0.8	0.88	0.88	0.81	0.85	0.72 (0.36-0.89)	0.79 (0.50-0.92)	0.80 (0.53-0.93)	0.87 (0.66-0.95)	0.88 (0.69-0.95)	0.81 (0.54-0.93)	0.82 (0.55-0.94)
Left postcentral gyrus	0.89	0.95	0.92	0.94	0.89	0.96	0.89	0.89 (0.71-0.96)	0.96 (0.88-0.98)	0.92 (0.80-0.97)	0.93 (0.81-0.98)	0.89 (0.71-0.96)	0.95 (0.87-0.98)	0.89 (0.72-0.96)
Left posterior cingulate cortex	0.86	0.84	0.86	0.82	0.82	0.86	0.84	0.86 (0.64-0.95)	0.85 (0.62-0.94)	0.86 (0.65-0.95)	0.82 (0.56-0.93)	0.83 (0.57-0.94)	0.86 (0.64-0.95)	0.84 (0.59-0.94)
Left precentral gyrus	0.95	0.95	0.94	0.92	0.89	0.9	0.92	0.94 (0.84-0.98)	0.94 (0.84-0.98)	0.92 (0.80-0.97)	0.92 (0.78-0.97)	0.89 (0.72-0.96)	0.88 (0.69-0.96)	0.86 (0.66-0.95)
Left precuneus	0.88	0.93	0.89	0.91	0.91	0.86	0.87	0.88 (0.69-0.96)	0.93 (0.80-0.97)	0.89 (0.71-0.96)	0.89 (0.71-0.96)	0.90 (0.73-0.96)	0.87 (0.66-0.95)	0.88 (0.68-0.96)
Left rostral anterior cingulate cortex	0.88	0.89	0.87	0.92	0.86	0.84	0.89	0.88 (0.69-0.96)	0.88 (0.71-0.96)	0.87 (0.68-0.95)	0.93 (0.81-0.97)	0.87 (0.67-0.95)	0.84 (0.61-0.94)	0.89 (0.72-0.96)
Left rostral middle frontal gyrus	0.91	0.91	0.9	0.9	0.92	0.89	0.91	0.89 (0.72-0.96)	0.90 (0.74-0.96)	0.89 (0.71-0.96)	0.88 (0.70-0.96)	0.89 (0.71-0.96)	0.88 (0.70-0.96)	0.90 (0.73-0.96)
Left superior frontal gyrus	0.75	0.79	0.79	0.82	0.74	0.72	0.69	0.71 (0.34-0.89)	0.76 (0.43-0.91)	0.75 (0.41-0.91)	0.78 (0.44-0.92)	0.69 (0.28-0.88)	0.71 (0.36-0.89)	0.68 (0.31-0.87)
Left superior parietal cortex	0.89	0.81	0.92	0.88	0.86	0.88	0.88	0.84 (0.60-0.94)	0.79 (0.51-0.92)	0.86 (0.66-0.95)	0.85 (0.62-0.94)	0.78 (0.49-0.92)	0.85 (0.64-0.95)	0.83 (0.59-0.94)
Left superior temporal gyrus	0.79	0.92	0.83	0.91	0.88	0.88	0.85	0.78 (0.47-0.92)	0.89 (0.72-0.96)	0.81 (0.54-0.93)	0.86 (0.65-0.95)	0.86 (0.64-0.95)	0.87 (0.66-0.95)	0.83 (0.59-0.94)
Left supramarginal gyrus	0.74	0.92	0.88	0.85	0.73	0.93	0.81	0.74 (0.40-0.90)	0.92 (0.80-0.97)	0.87 (0.68-0.95)	0.86 (0.64-0.95)	0.73 (0.39-0.90)	0.92 (0.80-0.97)	0.80 (0.52-0.92)
Left frontal pole	0.90	0.88	0.88	0.79	0.49	0.8	0.78	0.88 (0.69-0.96)	0.88 (0.69-0.96)	0.85 (0.63-0.95)	0.80 (0.51-0.92)	0.47 (-0.01-0.78)	0.79 (0.49-0.92)	0.76 (0.43-0.91)
Left temporal pole	0.80	0.79	0.75	0.88	0.79	0.78	0.76	0.81 (0.54-0.93)	0.80 (0.51-0.92)	0.76 (0.43-0.91)	0.87 (0.67-0.95)	0.79 (0.50-0.92)	0.77 (0.47-0.91)	0.76 (0.45-0.91)
Left transverse temporal gyrus	0.82	0.91	0.86	0.89	0.85	0.91	0.84	0.83 (0.58-0.94)	0.91 (0.77-0.97)	0.87 (0.67-0.95)	0.90 (0.73-0.96)	0.85 (0.63-0.95)	0.91 (0.76-0.97)	0.85 (0.62-0.95)
Left insula	0.71	0.55	0.23	0.53	0.55	0.44	0.6	0.69 (0.30-0.88)	0.56 (0.11-0.82)	0.24 (-0.31-0.66)	0.51 (0.06-0.79)	0.57 (0.10-0.83)	0.40 (-0.06-0.73)	0.59 (0.14-0.84)
Right banks superior temporal sulcus	0.92	0.93	0.86	0.89	0.87	0.92	0.9	0.92 (0.79-0.97)	0.93 (0.81-0.97)	0.87 (0.66-0.95)	0.90 (0.73-0.96)	0.88 (0.69-0.96)	0.90 (0.73-0.96)	0.90 (0.75-0.96)
Right caudal anterior cingulate cortex	0.82	0.8	0.81	0.79	0.82	0.79	0.82	0.80 (0.50-0.92)	0.79 (0.50-0.92)	0.80 (0.52-0.92)	0.78 (0.48-0.92)	0.81 (0.55-0.93)	0.77 (0.45-0.91)	0.77 (0.40-0.92)
Right caudal middle frontal gyrus	0.63	0.76	0.68	0.84	0.7	0.83	0.66	0.60 (0.18-0.84)	0.71 (0.36-0.89)	0.65 (0.25-0.86)	0.81 (0.55-0.93)	0.64 (0.25-0.85)	0.83 (0.59-0.94)	0.62 (0.22-0.85)
Right cuneus	0.95	0.93	0.94	0.96	0.96	0.97	0.96	0.94 (0.84-0.98)	0.92 (0.79-0.97)	0.92 (0.79-0.97)	0.95 (0.84-0.98)	0.95 (0.87-0.98)	0.96 (0.81-0.99)	0.95 (0.86-0.98)
Right entorhinal cortex	0.77	0.74	0.76	0.81	0.82	0.8	0.76	0.76 (0.44-0.91)	0.73 (0.39-0.90)	0.76 (0.42-0.91)	0.79 (0.49-0.92)	0.78 (0.47-0.92)	0.78 (0.48-0.92)	0.75 (0.42-0.91)
Right fusiform gyrus	0.83	0.88	0.87	0.79	0.83	0.88	0.87	0.83 (0.59-0.94)	0.89 (0.71-0.96)	0.88 (0.68-0.95)	0.80 (0.52-0.93)	0.84 (0.60-0.94)	0.89 (0.71-0.96)	0.87 (0.68-0.95)
Right inferior parietal cortex	0.79	0.84	0.8	0.73	0.74	0.82	0.79	0.80 (0.50-0.92)	0.84 (0.61-0.94)	0.80 (0.52-0.93)	0.73 (0.37-0.90)	0.74 (0.39-0.90)	0.80 (0.52-0.93)	0.77 (0.47-0.91)
Right inferior temporal gyrus	0.83	0.76	0.8	0.71	0.75	0.84	0.87	0.84 (0.59-0.94)	0.77 (0.46-0.91)	0.81 (0.53-0.93)	0.72 (0.37-0.89)	0.76 (0.44-0.91)	0.84 (0.61-0.94)	0.87 (0.68-0.95)
Right isthmus cingulate cortex	0.86	0.92	0.9	0.91	0.87	0.94	0.86	0.87 (0.67-0.95)	0.93 (0.81-0.97)	0.89 (0.72-0.96)	0.91 (0.76-0.97)	0.87 (0.68-0.95)	0.93 (0.81-0.97)	0.87 (0.66-0.95)
Right lateral occipital cortex	0.89	0.78	0.89	0.81	0.89	0.85	0.91	0.90 (0.73-0.96)	0.79 (0.50-0.92)	0.89 (0.72-0.96)	0.80 (0.52-0.92)	0.88 (0.71-0.96)	0.81 (0.47-0.93)	0.89 (0.69-0.96)
Right lateral orbitofrontal cortex	0.67	0.76	0.61	0.85	0.68	0.83	0.68	0.67 (0.27-0.87)	0.72 (0.36-0.89)	0.60 (0.16-0.84)	0.80 (0.51-0.93)	0.66 (0.26-0.87)	0.82 (0.58-0.93)	0.69 (0.31-0.88)
Right lingual gyrus	0.85	0.82	0.86	0.87	0.88	0.82	0.82	0.82 (0.57-0.93)	0.80 (0.52-0.92)	0.82 (0.54-0.94)	0.86 (0.65-0.95)	0.85 (0.64-0.95)	0.79 (0.51-0.92)	0.79 (0.50-0.92)
Right medial orbitofrontal cortex	0.51	0.72	0.61	0.55	0.69	0.54	0.66	0.51 (0.04-0.80)	0.69 (0.30-0.88)	0.61 (0.19-0.84)	0.55 (0.10-0.82)	0.63 (0.21-0.85)	0.53 (0.08-0.80)	0.67 (0.27-0.87)
Right middle temporal gyrus	0.91	0.87	0.87	0.82	0.78	0.87	0.81	0.90 (0.73-0.96)	0.88 (0.69-0.96)	0.87 (0.66-0.95)	0.82 (0.56-0.94)	0.79 (0.50-0.92)	0.84 (0.58-0.94)	0.81 (0.53-0.93)
Right parahippocampal gyrus	0.85	0.91	0.85	0.88	0.84	0.89	0.87	0.85 (0.64-0.95)	0.90 (0.74-0.96)	0.85 (0.63-0.94)	0.89 (0.70-0.96)	0.84 (0.60-0.94)	0.88 (0.70-0.96)	0.87 (0.66-0.95)
Right paracentral lobule	0.86	0.88	0.85	0.91	0.88	0.9	0.89	0.85 (0.63-0.94)	0.88 (0.70-0.96)	0.85 (0.63-0.94)	0.91 (0.76-0.97)	0.88 (0.69-0.96)	0.90 (0.75-0.97)	0.89 (0.72-0.96)
Right pars opercularis	0.90	0.87	0.88	0.94	0.93	0.91	0.91	0.90 (0.74-0.96)	0.88 (0.69-0.96)	0.88 (0.70-0.96)	0.94 (0.85-0.98)	0.92 (0.80-0.97)	0.91 (0.77-0.97)	0.91 (0.77-0.97)
Right pars orbitalis	0.88	0.93	0.91	0.93	0.89	0.86	0.9	0.88 (0.69-0.95)	0.91 (0.76-0.97)	0.91 (0.75-0.97)	0.93 (0.82-0.98)	0.90 (0.74-0.96)	0.85 (0.63-0.94)	0.89 (0.72-0.96)
Right pars triangularis	0.71	0.81	0.75	0.85	0.77	0.83	0.79	0.72 (0.36-0.89)	0.82 (0.55-0.93)	0.76 (0.44-0.91)	0.85 (0.63-0.95)	0.78 (0.48-0.92)	0.83 (0.58-0.94)	0.80 (0.51-0.93)
Right pericalcarine cortex	0.87	0.9	0.85	0.89	0.86	0.88	0.87	0.87 (0.68-0.95)	0.89 (0.73-0.96)	0.85 (0.62-0.94)	0.88 (0.71-0.96)	0.87 (0.66-0.95)	0.88 (0.69-0.95)	0.88 (0.68-0.95)
Right postcentral gyrus	0.91	0.95	0.93	0.94	0.92	0.94	0.92	0.91 (0.76-0.97)	0.94 (0.83-0.98)	0.92 (0.80-0.97)	0.93 (0.81-0.97)	0.91 (0.77-0.97)	0.94 (0.84-0.98)	0.92 (0.78-0.97)
Right posterior cingulate cortex	0.92	0.9	0.88	0.85	0.91	0.89	0.87	0.90 (0.73-0.96)	0.89 (0.72-0.96)	0.87 (0.67-0.95)	0.84 (0.60-0.94)	0.90 (0.73-0.96)	0.89 (0.70-0.96)	0.86 (0.66-0.95)
Right precentral gyrus	0.76	0.82	0.82	0.86	0.82	0.81	0.77	0.76 (0.44-0.91)	0.83 (0.57-0.94)	0.82 (0.56-0.93)	0.86 (0.64-0.95)	0.83 (0.57-0.94)	0.81 (0.55-0.93)	0.77 (0.46-0.91)
Right precuneus	0.85	0.87	0.84	0.9	0.89	0.94	0.88	0.84 (0.60-0.94)	0.80 (0.53-0.93)	0.83 (0.57-0.94)	0.88 (0.70-0.96)	0.88 (0.70-0.96)	0.89 (0.73-0.96)	0.87 (0.67-0.95)
Right rostral anterior cingulate cortex	0.86	0.82	0.79	0.77	0.79	0.84	0.82	0.87 (0.67-0.95)	0.83 (0.58-0.94)	0.80 (0.51-0.92)	0.78 (0.47-0.92)	0.80 (0.51-0.93)	0.84 (0.60-0.94)	0.82 (0.57-0.93)
Right rostral middle frontal gyrus	0.07	0.35	0.22	0.44	0.19	0.51	0.2	0.07 (-0.42-0.53)	0.31 (-0.18-0.68)	0.21 (-0.29-0.62)	0.42 (-0.06-0.75)	0.17 (-0.27-0.58)	0.51 (0.03-0.80)	0.19 (-0.30-0.61)

Right superior frontal gyrus	0.64	0.76	0.72	0.89	0.73	0.81	0.68	0.62 (0.22-0.85)	0.73 (0.39-0.89)	0.71 (0.36-0.89)	0.84 (0.59-0.94)	0.67 (0.28-0.87)	0.82 (0.56-0.93)	0.67 (0.30-0.87)
Right superior parietal cortex	0.84	0.86	0.87	0.89	0.91	0.87	0.79	0.84 (0.60-0.94)	0.85 (0.62-0.94)	0.86 (0.65-0.95)	0.87 (0.67-0.95)	0.88 (0.71-0.96)	0.84 (0.60-0.94)	0.79 (0.49-0.92)
Right superior temporal gyrus	0.91	0.9	0.92	0.92	0.89	0.94	0.92	0.89 (0.72-0.96)	0.90 (0.75-0.96)	0.91 (0.75-0.97)	0.91 (0.77-0.97)	0.87 (0.66-0.95)	0.94 (0.84-0.98)	0.91 (0.75-0.97)
Right supramarginal gyrus	0.83	0.92	0.91	0.86	0.84	0.85	0.81	0.83 (0.59-0.94)	0.92 (0.78-0.97)	0.91 (0.75-0.97)	0.86 (0.66-0.95)	0.83 (0.60-0.94)	0.84 (0.60-0.94)	0.82 (0.55-0.93)
Right frontal pole	0.52	0.59	0.54	0.67	-0.07	0.81	0.69	0.48 (0.03-0.78)	0.60 (0.15-0.84)	0.53 (0.07-0.81)	0.67 (0.28-0.87)	-0.06 (-0.54-0.44)	0.81 (0.54-0.93)	0.68 (0.29-0.87)
Right temporal pole	0.68	0.68	0.52	0.59	0.44	0.66	0.52	0.60 (0.19-0.84)	0.59 (0.18-0.83)	0.48 (0.02-0.77)	0.52 (0.07-0.80)	0.40 (-0.08-0.74)	0.56 (0.13-0.82)	0.45 (0.00-0.76)
Right transverse temporal gyrus	0.91	0.88	0.89	0.91	0.89	0.91	0.88	0.91 (0.77-0.97)	0.87 (0.68-0.95)	0.88 (0.69-0.96)	0.89 (0.72-0.96)	0.88 (0.70-0.96)	0.90 (0.75-0.96)	0.88 (0.69-0.95)
Right insula	0.69	0.7	0.72	0.49	0.58	0.52	0.64	0.67 (0.28-0.87)	0.70 (0.33-0.88)	0.69 (0.32-0.88)	0.43 (-0.03-0.75)	0.52 (0.06-0.80)	0.42 (-0.08-0.75)	0.64 (0.24-0.86)
Global brain measures														
Left total cortical volume (ml)	0.99	0.99	0.99	0.99	0.99	0.99	0.99	0.98 (0.96-0.99)	0.99 (0.97-1.00)	0.99 (0.96-1.00)	0.99 (0.96-1.00)	0.98 (0.96-0.99)	0.99 (0.97-1.00)	0.99 (0.96-1.00)
Right total cortical volume (ml)	0.97	0.98	0.98	0.98	0.98	0.98	0.98	0.98 (0.93-0.99)	0.98 (0.95-0.99)	0.98 (0.94-0.99)	0.98 (0.95-0.99)	0.98 (0.93-0.99)	0.98 (0.94-0.99)	0.98 (0.94-0.99)
Left total cortical white matter (ml)	1.00	1	1	1	1	1	1	1.00 (0.99-1.00)	1.00 (1.00-1.00)	1.00 (0.99-1.00)	1.00 (1.00-1.00)	1.00 (0.99-1.00)	1.00 (1.00-1.00)	1.00 (0.99-1.00)
Right total cortical white matter (ml)	1.00	1	1	1	1	1	1	1.00 (0.99-1.00)	1.00 (0.99-1.00)	1.00 (0.99-1.00)	1.00 (0.99-1.00)	1.00 (0.99-1.00)	1.00 (0.99-1.00)	1.00 (0.99-1.00)
Left total cortical surface area (cm^2)	1.00	1	1	1	1	1	1	1.00 (0.99-1.00)	1.00 (0.99-1.00)	1.00 (0.99-1.00)	1.00 (0.99-1.00)	1.00 (0.99-1.00)	1.00 (0.99-1.00)	1.00 (0.99-1.00)
Right total cortical surface area (cm^2)	1.00	1	1	1	1	1	1	1.00 (1.00-1.00)	1.00 (0.99-1.00)	1.00 (1.00-1.00)	1.00 (1.00-1.00)	1.00 (1.00-1.00)	1.00 (1.00-1.00)	1.00 (1.00-1.00)
Left mean cortical thickness (mm)	0.89	0.95	0.93	0.9	0.88	0.89	0.88	0.89 (0.72-0.96)	0.96 (0.88-0.98)	0.93 (0.81-0.97)	0.90 (0.74-0.96)	0.88 (0.69-0.95)	0.89 (0.73-0.96)	0.89 (0.71-0.96)
Right mean cortical tickness (mm)	0.74	0.86	0.81	0.85	0.78	0.85	0.79	0.74 (0.41-0.90)	0.84 (0.60-0.94)	0.80 (0.53-0.93)	0.84 (0.59-0.94)	0.77 (0.47-0.91)	0.85 (0.64-0.95)	0.79 (0.49-0.92)
Left cerebellar white matter (ml)	0.90	0.91	0.89	0.91	0.91	0.92	0.93	0.90 (0.74-0.96)	0.91 (0.76-0.97)	0.89 (0.71-0.96)	0.91 (0.76-0.97)	0.90 (0.73-0.96)	0.92 (0.79-0.97)	0.92 (0.79-0.97)
Right cerebellar white matter (ml)	0.93	0.95	0.96	0.96	0.95	0.94	0.95	0.93 (0.80-0.97)	0.95 (0.87-0.98)	0.96 (0.88-0.98)	0.96 (0.89-0.99)	0.95 (0.86-0.98)	0.94 (0.84-0.98)	0.94 (0.85-0.98)
Left cerebellar gray matter (ml)	0.99	0.99	0.99	0.99	0.99	0.99	0.99	0.99 (0.98-1.00)	0.99 (0.97-1.00)	0.99 (0.97-1.00)	0.99 (0.97-1.00)	0.99 (0.97-1.00)	0.99 (0.97-1.00)	0.99 (0.97-1.00)
Right cerebellar gray matter (ml)	1.00	0.99	1	0.99	0.99	1	0.99	1.00 (0.99-1.00)	0.99 (0.98-1.00)	1.00 (0.99-1.00)	0.99 (0.98-1.00)	0.99 (0.98-1.00)	1.00 (0.99-1.00)	0.99 (0.98-1.00)
Intracranial volume (ml)	0.99	0.99	0.99	0.99	0.97	1	0.98	0.99 (0.98-1.00)	0.99 (0.96-1.00)	0.99 (0.98-1.00)	0.98 (0.95-0.99)	0.95 (0.80-0.98)	1.00 (0.99-1.00)	0.97 (0.89-0.99)
Total brain volume without ventricles (ml)	1.00	1	1	1	1	1	1	1.00 (0.99-1.00)	1.00 (1.00-1.00)	1.00 (0.99-1.00)	1.00 (0.99-1.00)	1.00 (0.99-1.00)	1.00 (1.00-1.00)	1.00 (0.99-1.00)

Abbreviations: Orig = Original scan; FS df =FreeSurfer defacing; FSL df =FSL defacing; FM = Face Masking; Suffix 1 and 2 indicate first and second scan respectively, i.e. test-retest; ICC = intraclass correlation coefficient; CI = confidence interval.
